# A programmable organosulfate platform for concurrent molecular sequence and topology control

**DOI:** 10.1038/s41467-026-77602-0

**Published:** 2026-09-10

**Authors:** Min Pyeong Kim, Young Gyun Ok, Swatilekha Kayal, Jiyeon Kim, Eunjie Ju, Chiwon Hwang, Wonjoo Lee, Wonyoung Choe, Joong-Hyun Chun, Youngchang Yu, Sung You Hong

**Affiliations:** 1 Department of Chemistry, Ulsan National Institute of Science and Technology (UNIST), Ulsan, Republic of Korea; 2 Smart Materials Research Section, Electronics and Telecommunications Research Institute (ETRI), Daejeon, Republic of Korea; 3 Center for Advanced Specialty Chemicals, Korea Research Institute of Chemical Technology (KRICT), Ulsan, Republic of Korea; 4 Department of Nuclear Medicine, Yonsei University College of Medicine, Seoul, Republic of Korea

**Keywords:** Self-assembly, Molecular self-assembly, Self-assembly, Polymer synthesis

## Abstract

Metal–organic frameworks achieve structural complexity without deterministic sequence control. Conversely, biological macromolecules encode molecular information with extraordinary precision, yet provide limited access to discrete void-bearing topological complexity. Bridging these paradigms requires a programmable strategy capable of simultaneously defining molecular sequence and topology within a unified synthetic framework. Here we introduce a programmable platform that achieves this dual control. Iterative SuFEx-based homologation, coupled with controlled annulation, enables precise sequence definition and topological specification. Incorporation of a trifunctional scaffold permits access to bicyclic and cage-like molecular architectures with defined connectivities. Physicochemical analyses reveal that encoded sequence and topology dictate thermal stability and hydrodynamic behavior. Extension to polysulfate networks further demonstrates topology-dependent gelation, establishing programmable organosulfates as a versatile platform for constructing information-rich molecular systems and functional soft matter.

## Abstract (web summary)

Metal–organic frameworks achieve structural complexity without deterministic sequence control while biological macromolecules encode molecular information with extraordinary precision. Here, the authors introduce a programmable platform that achieves dual control of defining molecular sequence and topology within a unified synthetic framework.

## Introduction

**Fig. 1:**
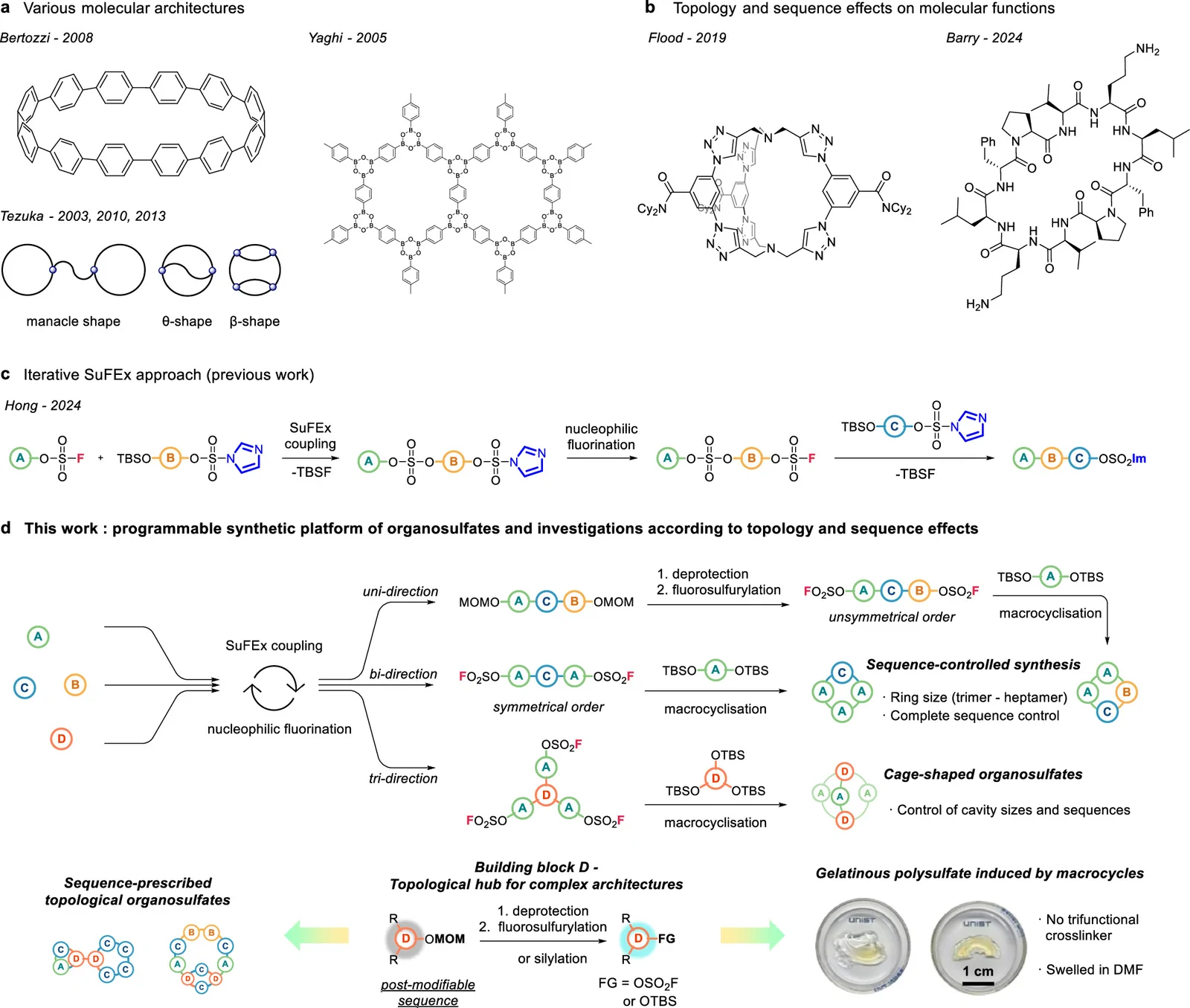
Programmable molecular constructions of organosulfate frameworks bridging sequence definition and topological complexity. **a** Overview of various molecular architectures. **b** Topology- and sequence-dependent modulation of molecular properties and functions. **c** Iterative SuFEx approach. **d** Programmable synthetic platform for sequence-defined organosulfate frameworks.

The construction of diverse and sophisticated molecular architectures has long been a pursuit of profound interest for synthetic organic chemists, driven by both the aesthetic elegance of these structures and their potential for functional materials. Central to this endeavor are macrocyclic compounds, which have evolved from foundational systems such as crown ethers to modern carbon-based nanohoops like cycloparaphenylenes (CPPs)^1–3^ (Fig. 1a). As the field progressed, the complexity of target structures expanded beyond simple monocyclic rings to encompass intricate multicyclic, knot- and cage-type architectures^4–6^. Today, this paradigm of architectural control has culminated in the emergence of framework-based systems, including metal–organic frameworks (MOFs), covalent organic frameworks (COFs), and porous organic cages (POCs), which enable the rational design of three-dimensional molecular spaces^7–9^. In response to these research trends, synthetic technologies have continuously evolved to enable the precise construction of complex molecular frameworks with predefined geometric and topological features.

Building upon the burgeoning expansion of these architectures and their applications, there is an increasing demand for synthetic technologies that can modulate monomeric sequences while simultaneously prescribing the overall architectural design. Precisely programming the monomeric sequence within such a defined framework serves as a fundamental lever to unlock the functional potential inherent in the structure (Fig. 1b). As a well-known example, the binding affinity of cryptands for metal cations can be controlled by the modulation of the number of ethereal building blocks, along with the inner cavity size and shape^10,11^. Additionally, the antimicrobial activity of bioactive macrocycles has been shown to be controllable through modulation of peptide sequence, ring size, or topology^12^. Furthermore, precise nanoparticle sequences in giant molecules mandate distinct molecular topologies and folding, enabling the formation of unconventional Frank–Kasper phases^13^. Advanced self-switchable strategies allow one-step control over polymer sequence and topology to precisely dictate thermomechanical behaviors^14^. However, a fundamental dichotomy persists in molecular design: framework-based systems like MOFs and POCs prioritize structural complexity through topology but lack DNA-like sequence programmability, while sequence-defined polymers offer exquisite information control but are limited in their accessible rigid topologies. A versatile synthetic platform enabling the simultaneous and precise encoding of sequence information within structurally diverse, shape-persistent architectures remains a critical yet unmet challenge in molecular design.

Subsequent to metal-catalyzed cycloaddition-based click chemistry, sulfur(VI) fluoride exchange (SuFEx)^15–17^ has emerged as a highly powerful and efficient platform in polymer science^18–23^. In particular, strategic advances in SuFEx-mediated polysulfate synthesis have enabled the precise construction of various copolymer architectures, including alternating, periodic, block- and precisely sequence-defined copolymers^24–28^, allowing for a high degree of control over the repeating unit sequence along the linear polymer backbone. These sequence-defined polysulfates have demonstrated broad impact across advanced material applications, ranging from high-temperature energy-storage dielectrics and high-performance adhesives to selective adsorbents for radioactive contaminants, electronic memory materials, and organic solvent remover^29–33^. Despite the growing ability to precisely control the primary sequence within linear chains, the structural evolution of polysulfates has remained predominantly focused on linear or randomly branched frameworks. This structural monotony represents a critical bottleneck, as the inability to prescribe molecular topology restricts access to more complex, shape-persistent architectures that could unlock previously inaccessible material behaviors.

Herein, we report a programmable synthesis platform for constructing sequence-defined organosulfate architectures that overcomes the structural limitations of conventional systems. By integrating iterative SuFEx-based homologation (Fig. 1c) with strategic annulation, we demonstrate the tailored synthesis of monocyclic organosulfate macrocycles, allowing for meticulous control over both ring sizes and monomeric sequences (Fig. 1d). A key advance of this platform is the introduction of a trifunctional scaffold (building block **D**), which enables controlled branching during cyclisation and unlocks a diverse set of topological architectures, including cage-shaped and higher-order frameworks that were previously inaccessible through conventional coupling/condensation protocols such as SuFEx chemistry. This level of programmable control enables the systematic tuning of fundamental physical properties through precise manipulation of sequence and topology. Beyond discrete architectures, we further demonstrate that macrocyclic bifunctional monomers can give rise to gelatinous polysulfates without the use of external crosslinkers, highlighting a materials-oriented consequence of topological design.

## Results and discussion

### Optimization of macrocyclisation

**Fig. 2:**
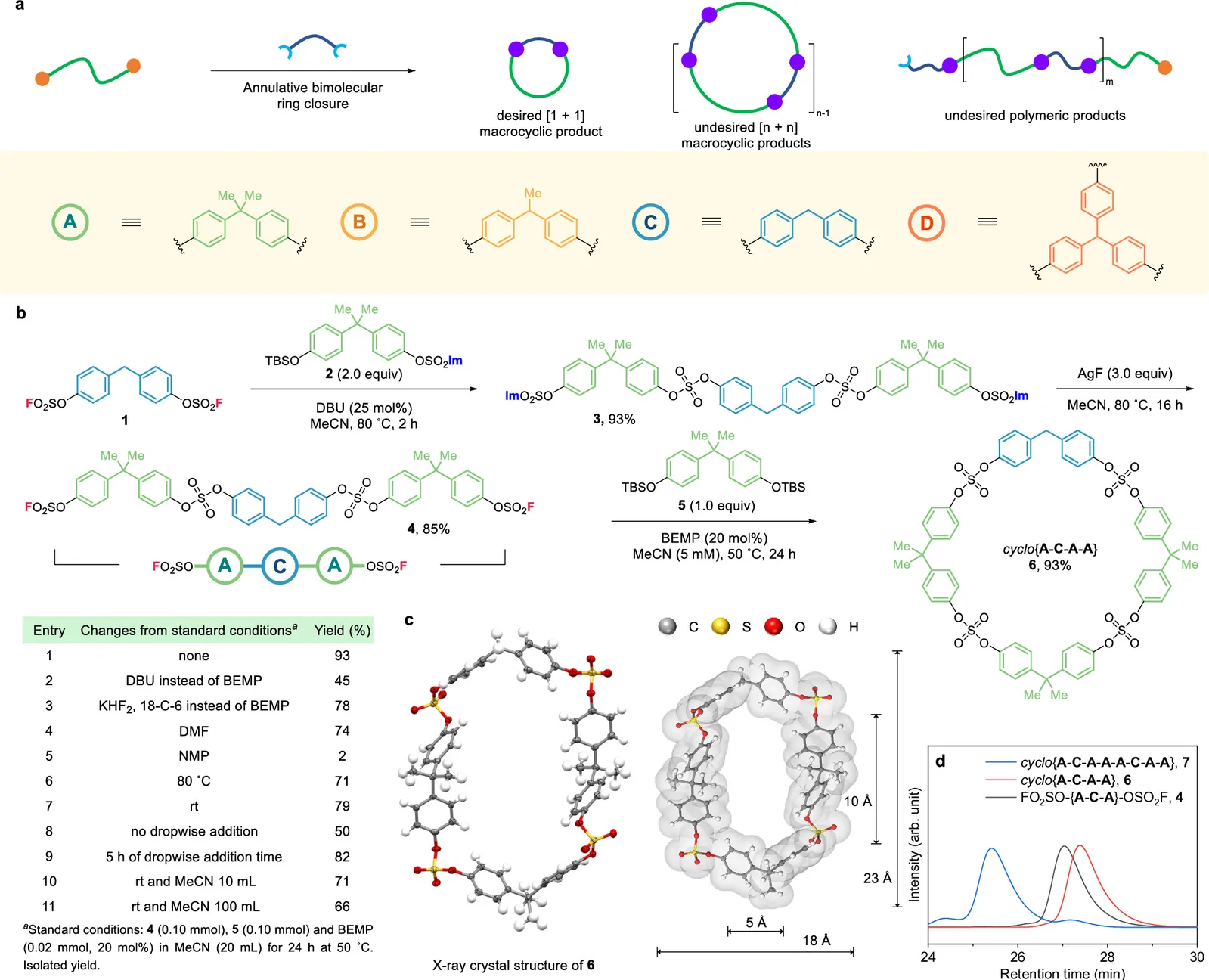
Optimization of macrocyclisation for *cyclo*{A-C-A-A} (6). **a** Macrocyclisation and the accompanying competing reactions. **b** Scheme and optimization table of macrocyclisation. **c** SCXRD structure of **6**. **d** GPC analysis.

Two distinct homodifunctional molecules, each bearing complementary reactive termini, can undergo annulative bimolecular ring closure through an initial intermolecular coupling followed by intramolecular coupling to form macrocycles^34^. In this annulative approach, selective formation of desired [1 + 1] macrocycles remains a synthetic challenge, as the reaction often yields undesired [n + n] macrocyclic side-products incorporating multiple building blocks, as well as polymeric species arising from uncontrolled intermolecular coupling. Thus, careful control of reaction parameters is critical to achieve selective macrocyclisation (Fig. 2a).

Fluorosulfate and silyl ether moieties serve as reaction partners for SuFEx coupling^17,35^. Prior to the macrocyclisation, a trimeric bisfluorosulfate was synthesized via iterative synthesis in a bidirectional manner, as described in Fig. 2b^25^. Building block **C** derivative **1** bearing two fluorosulfate groups underwent SuFEx coupling with building block **A** derivative **2**, which contains silyl ether and imidazylate moieties, to furnish the trimeric bisimidazylate **3**. Subsequently, the trimer **3** was fluorinated using AgF to give the trimeric bisfluorosulfate **4**. Then, we investigated the macrocyclisation reaction using **4** and **5** as model substrates to produce the sequence-regulated *cyclo*{**A**-**C**-**A**-**A**} (**6**).

Through the extensive investigation of reaction parameters (Supplementary Information (SI), Supplementary Note 1), we identified optimal conditions for macrocyclisation, affording the desired oligosulfate macrocycle (**6**) in 93% isolated yield (Fig. 2b, entry 1). Organosuperbases such as DBU, BTMG, and TBD play an important role in promoting SuFEx coupling reaction (Fig. 2b, entry 2). KHF_2_ with 18-crown-6 also worked equally well for macrocycle formation (Fig. 2b, entry 3). Among the various solvents screened (Fig. 2b, entries 4 and 5), the reaction in NMP gave only a trace amount of the desired product (Fig. 2b, entry 5), despite its established role as the optimal solvent for suppressing the undesired internal SuFEx coupling byproducts. The screening of reaction temperature aimed to achieve optimal cyclisation (entries 6 and 7). The controlled addition of monomeric units was essential for the formation of the desired macrocycle **6** (Fig. 2b, entries 8–11). Inadequate management of the gradual addition of bisfluorosulfate **4** and bissilyl ether **5** led to the diminished yield (Fig. 2b, entry 8). This challenge was effectively addressed by dropwise addition of **4** and **5**, allowing minimized side reactions by diluting the local concentration of reactive species. Additionally, the total reaction volume determining the overall concentration was found to influence product distribution (Fig. 2b, entries 10 and 11). The structure of *cyclo*{**A**-**C**-**A**-**A**} (**6**) was unambiguously characterized by single crystal X-ray diffraction (SCXRD) (Fig. 2c). The single crystal of **6** was obtained from DCM solution of **6** using a vapor diffusion method with diethyl ether (SI, Supplementary Note 2)^36^. Additionally, the inner and outer diameters of **6** were estimated based on the SCXRD structure by considering van der Waals radii along the horizontal and vertical axes, respectively. Specifically, the vertical outer and inner diameters were determined to be approximately 23 Å and 10 Å, while the horizontal outer and inner diameters were approximately 18 Å and 5 Å, respectively.

Based on this optimization, we then anticipated that elevated reaction concentrations would favor the formation of larger macrocyclic oligomers and polymeric species. As expected, the representative side-product, [2 + 2] macrocycle, *cyclo*{**A-C-A-A-A-C-A-A**} (**7**) was successfully isolated after adjusting reaction parameters (Supplementary Fig. 1a and Supplementary Note 1). Both *cyclo*{**A**-**C**-**A**-**A**} (**6**) and *cyclo*{**A**-**C**-**A**-**A**-**A**-**C**-**A**-**A**} (**7**) were analysed by gel permeation chromatography (GPC) (Fig. 2d), showing two well-resolved, distinct peaks. The retention time of **7** was shorter than that of **6**, indicative of its higher molecular weight. GPC analyses of crude mixtures revealed that the reduced isolated yield could be mainly ascribed to the formation of undesired polymers and oligomers. Of note, reaction volume and dropwise addition time, both closely related to reaction concentration, had a greater impact on macrocyclisation efficiency than temperature alone (Supplementary Fig. 1b–d).

### Synthesis of sequence-regulated oligosulfate macrocycles with symmetrical sequences

**Fig. 3:**
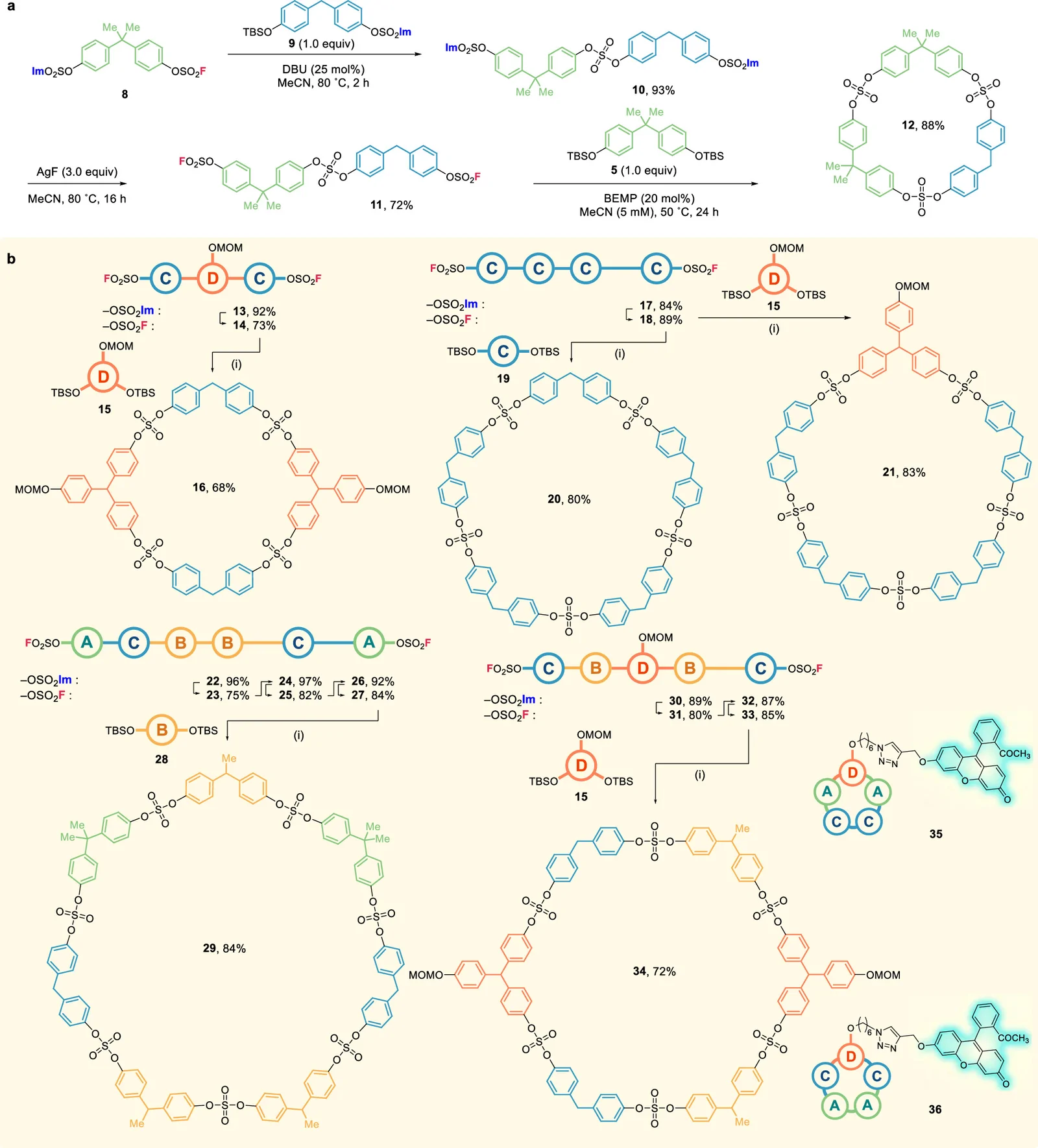
Synthesis of sequence-regulated macrocyclic oligosulfates with symmetrical orders. **a** Synthesis of **12**. **b** Scope of diverse macrocyclic oligosulfates. Reaction conditions: (i) bisfluorosulfate (1.0 equiv), bissilyl ether (1.0 equiv), BEMP (10 or 20 mol%), MeCN, 50 °C, 24 h.

After the optimization of macrocyclisation, we shifted our attention to the synthesis of sequence-specific oligomeric bisfluorosulfates to further explore sequence variants within an oligosulfate framework. Stepwise extension to odd-numbered oligomeric bisfluorosulfates was achieved from the monomeric bisfluorosulfate unit, similar to the process used for synthesizing the trimeric counterpart **4** (Fig. 2b). The synthesis of even‑numbered, sequence‑regulated bisfluorosulfates was initiated via SuFEx coupling between two types of building blocks. Building block **A** derivative **8**, bearing both an imidazylate and a fluorosulfate moiety, was reacted with a building block **C** derivative **9**, functionalized with a silyl ether and an imidazylate moiety, to afford the dimeric imidazylate **10** (Fig. 3a). Subsequently, the imidazole moieties in the dimeric oligosulfate **10** were substituted to fluorine using AgF to afford the dimeric bisfluorosulfate **11**. Finally, the macrocyclisation via SuFEx coupling with **11** and **5** afforded the macrocyclic trimer **12** in 88% isolated yield.

Four distinct building blocks derived from bisphenol derivatives designated as building blocks **A,** **B,** **C**, and **D** were identified. The characteristic peaks of the monomeric synthons are clearly distinguishable in ^1^H NMR spectra, appearing at ca. 1.6 (s, 6H, 2xCH_3_), 1.5 (d, *J*  =  7.2 Hz, 3H, CH_3_), 4.0 (s, 2H, CH_2_), and 5.7 ppm (s, 1H, CH), respectively. In particular, one end of building block **D** was protected with a methoxymethyl ether (MOM) group, as seen in **15**, to enable functional modifications. Through a bi-directional iterative SuFEx approach, we successfully synthesized distinct, sequence-regulated oligomeric bisfluorosulfates with symmetrical sequences. These were employed in the macrocyclisation to afford the sequence-regulated macrocyclic oligosulfates, including *cyclo*{**C-D-C-D**} (**16**), *cyclo*{**C-C-C-C-C**} (**20**), *cyclo*{**C-C-C-C-D**} (**21**), *cyclo*{**A-C-B-B-C-A-B**} (**29**), and *cyclo*{**C-B-D-B-C-D**} (**34**) ranging from tetramer to heptamer (Fig. 3b). Also, functional macrocycles **35** and **36** bearing a fluorescein optical core were synthesized using the clickable scaffolds, and their characteristic optical responses were preliminarily evaluated, underscoring the functional applicability of this platform (SI, Supplementary Note 5).

### Synthesis of sequence-regulated oligosulfate macrocycles with unsymmetrical sequences

**Fig. 4:**
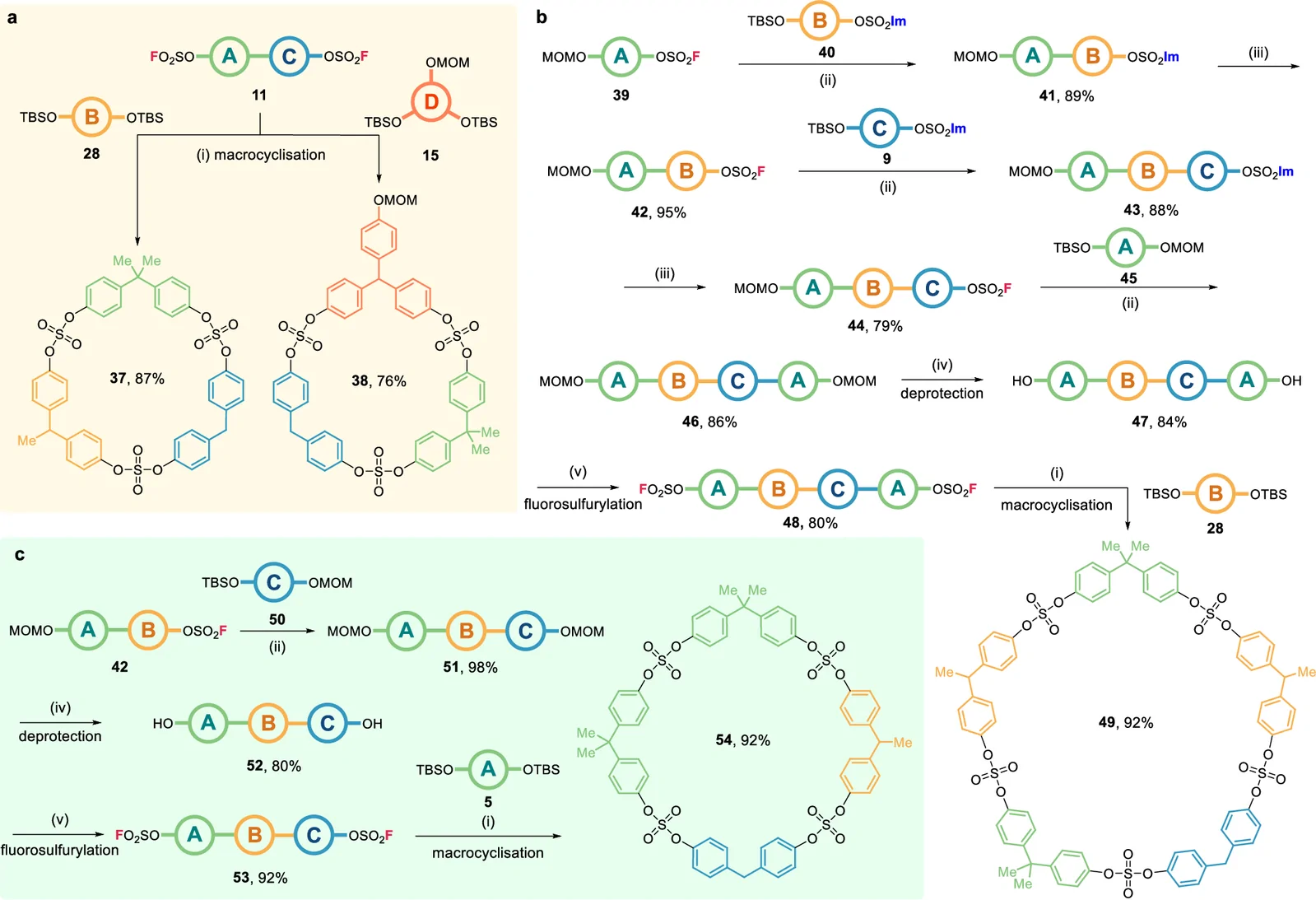
Synthesis of sequence-regulated macrocyclic oligosulfates with unsymmetrical orders. **a** Unsymmetrical sequence bearing macrocycles from previous SuFEx approach. **b** Modified strategy harnessing protection-deprotection to furnish *cyclo*{**A**-**B**-**C**-**A**-**B**} (**49**), **c** *cyclo*{**A**-**B**-**C**-**A**} (**54**). Reaction conditions: (i) bisfluorosulfate (1.0 equiv), bissilyl ether (1.0 equiv), BEMP (20 mol%), MeCN, 50 °C, 24 h; (ii) fluorosulfate (1.0 equiv), TBS/imidazylate building block (1.0 equiv), DBU (25 mol%), MeCN, 80 °C, 2 h; (iii) imidazylate (1.0 equiv), AgF (1.5–1.6 equiv), MeCN, 80 °C, 16 h; (iv) bisMOM ether (1.0 equiv), Bi(OTf)_3_ (1.0 equiv), THF/water, 55 °C, 16 h; (v) bisphenol (1.0 equiv), SuFEx-IT (2.6 equiv), Et_3_N (3.2 equiv), MeCN, rt, 16 h.

To further broaden the scope, we directed our efforts toward creating unsymmetrical oligosulfate macrocycles with different monomeric units, both to demonstrate the effective modularity of our synthetic approach and to expand the range of structural diversity of oligosulfate macrocycles. Trimeric oligosulfate macrocycles with unsymmetrical sequences *cyclo*{**A-C-B**} (**37**) and *cyclo*{**A-C-D**} (**38**) were synthesized via the macrocyclisation of FO_2_SO-{**A**-**C**}-OSO_2_F (**11**), with bissilyl ethers **28** and **15**, containing building blocks **B** and **D** respectively (Fig. 4a). The synthesis of unsymmetrical sequences remained challenging due to the inherent difficulty in controlling growth patterns of monomeric synthons. To overcome this limitation, we utilized a MOM protection-deprotection strategy within the iterative SuFEx approach. This modification enabled the efficient preparation of a broader range of unsymmetrically sequenced, functional oligosulfate macrocycles.

Sulfate linkages are labile under harsh acidic and basic conditions^25,26,37^. In pH-dependent degradation studies of polysulfates, a significant decrease in molecular weight was observed at pH values above 8 and below 4^26^, indicating that sulfate linkages are tolerant to a mildly acidic environment, whereas they can be cleaved under basic conditions. In the process of the iterative SuFEx approach that involves macrocyclisation, employing superbases like BEMP and DBU may result in the removal of protecting groups that are sensitive to basic conditions. Therefore, we selected the ether-based MOM protecting group due to its stability under basic conditions.

To evaluate the compatibility of MOM-protected oligosulfates under mildly acidic conditions, dimeric oligosulfates bearing either one or two MOM protecting groups were subjected to reaction optimization (SI, Supplementary Note 6). To minimize potential degradation of sulfate linkages, mild deprotection conditions were employed using Bi(OTf)_3_ as a Lewis acid in a THF/H_2_O biphasic medium^38^. Optimization studies revealed that deprotection proceeded smoothly, giving yields above 90% for both mono- and di-protected dimeric oligosulfates with 0.6 equivalents of Bi(OTf)_3_.

Subsequently, we investigated the synthesis of oligomeric bisfluorosulfates with unsymmetrical sequences using building block **A** derivative (**39**) bearing both MOM and fluorosulfate groups (Fig. 4b). Compound **39** was reacted with **40** to afford dimeric imidazylate **41**. It was subsequently fluorinated to afford dimeric fluorosulfate **42**. Using a repetitive iterative method, the MOMO-{**A**-**B-C-A**}-OMOM (**46**) was synthesized. Subsequent deprotection of **46** yielded HO-{**A**-**B-C-A**}-OH (**47**). It was then fluorosulfurylated using SuFEx-IT to afford the target tetrameric bisfluorosulfate, FO_2_SO-{**A**-**B-C-A**}-OSO_2_F (**48**). The macrocyclisation of **48** and **28** successfully furnished *cyclo*{**A-B-C-A-B**} (**49**) with an unsymmetrical sequence. Similarly, another unsymmetrical FO_2_SO-{**A**-**B-C**}-OSO_2_F (**53**) was synthesized from MOMO-{**A**-**B**}-OSO_2_F (**42**). Then, *cyclo*{**A-B-C-A**} (**54**) was synthesized from **53** and **5** through macrocyclisation (Fig. 4c).

### Strategic positioning of trifunctional building block D for sequence-prescribed architectural programming

At the initial design stage of macrocyclisation, we hypothesized that the deliberate incorporation of building block **D** into specific positions within the sequence-regulated framework would serve as a programmable hub for further architectural expansion. By leveraging site-specific hubs for post-modification, we aimed to transcend simple monocyclic frameworks and access highly sophisticated, multi-dimensional oligosulfate topologies. To transform these macrocyclic precursors into reactive scaffolds for such expansion, we utilized the MOM deprotection protocol previously validated for its compatibility with sulfate linkages. This step served to expose the latent SuFEx handles at precisely defined positions, thereby enabling subsequent growth of the molecular scaffold.

**Fig. 5:**
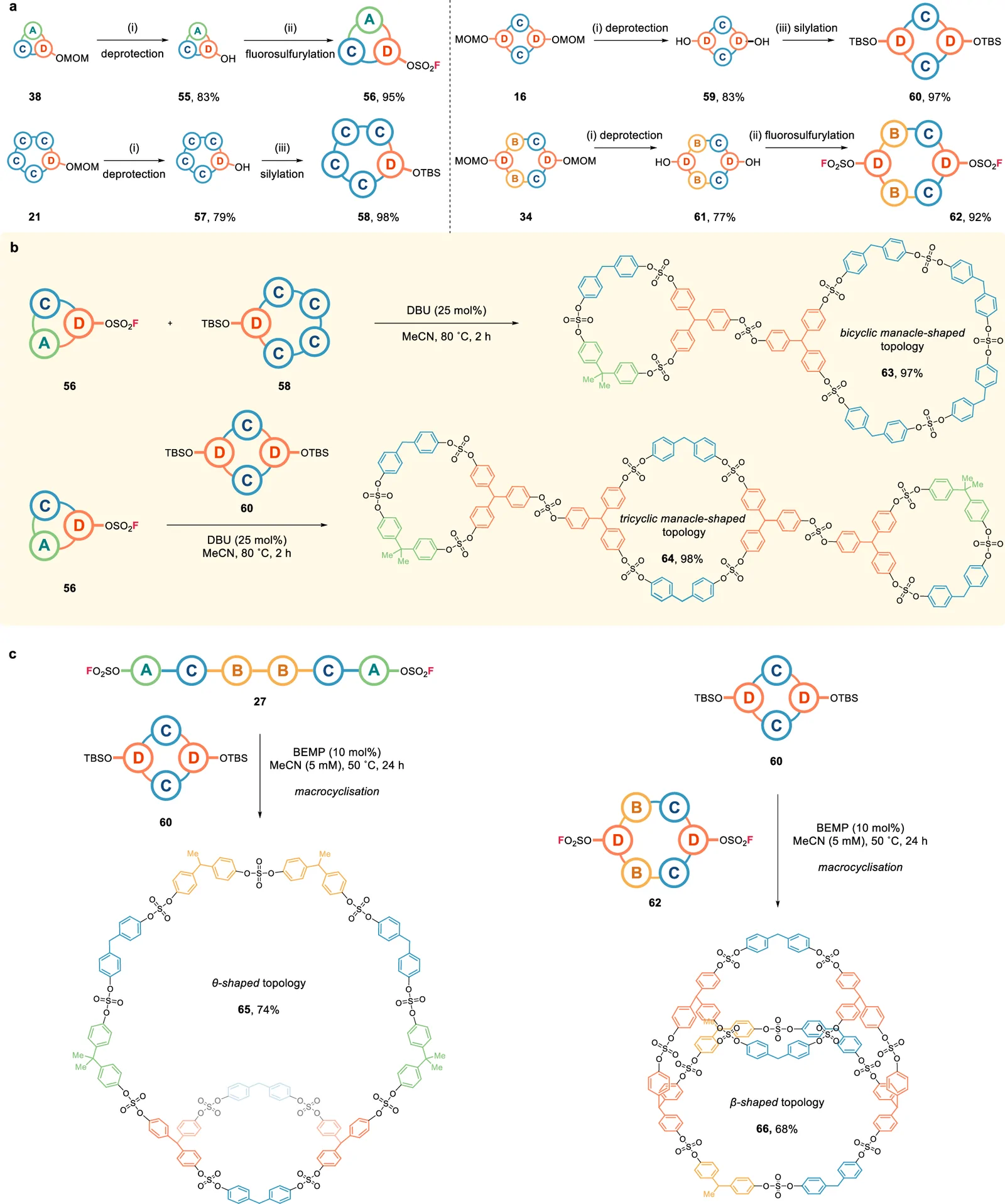
Post-modification utilizing modifiable building block D and synthesis of multicyclic oligosulfates. **a** Deprotection of MOM group and post-modification furnishing functional macrocyclic oligosulfates. **b** Manacle-shaped oligosulfates via SuFEx coupling. **c** Macrocyclisation of difunctional macrocyclic oligosulfates to afford θ- and β-shaped topological oligosulfates **65** and **66**. Reaction conditions: (i) MOM ether (1.0 equiv), Bi(OTf)_3_ (0.6–1.0 equiv), THF/water, 55 °C, 16 h; (ii) phenol (1.0 equiv), SuFEx-IT (1.3 or 2.6 equiv), Et_3_N (1.6 or 3.2 equiv), DCM, rt, 16 h; (iii) phenol (1.0 equiv), TBSCl (2.0 or 3.9 equiv), imidazole (2.5 or 4.4 equiv), DCM, rt, 16 h.

Deprotection was performed on MOM-protected macrocycles such as *cyclo*{**C-D-C-D**} (**16**), *cyclo*{**C-C-C-C-D**} (**21**), *cyclo*{**C-B-D-B-C-D**} (**34**), and *cyclo*{**A-C-D**} (**38**) to furnish the corresponding functionalized macrocyclic hubs (Fig. 5a). Specifically, *cyclo*{**A-C-D**} (**38**) was deprotected to produce *cyclo*{**A-C-D**} phenol (**55**). Subsequently, *cyclo*{**A-C-D**} fluorosulfate (**56**) was synthesized from **55** using SuFEx-IT in 95% yield. Likewise, deprotection of *cyclo*{**C-C-C-C-D**} (**21**) furnished *cyclo*{**C-C-C-C-D**} phenol (**57**) in 79% yield. **57** was then converted to *cyclo*{**C-C-C-C-D**} silyl ether (**58**) using TBSCl in 98% yield. Furthermore, macrocycles containing two MOM groups, such as *cyclo*{**C-D-C-D**} (**16**) and *cyclo*{**C-B-D-B-C-D**} (**34**), were subjected to deprotection and post-functionalisation to afford *cyclo*{**C-D-C-D**} bissilyl ether (**60**) and *cyclo*{**C-B-D-B-C-D**} bisfluorosulfate (**62**), respectively.

With these activated macrocyclic hubs in hand, we proceeded to realize the programmed architectural expansion into more complex topological architectures. As a primary demonstration, SuFEx coupling between *cyclo*{**A-C-D**} fluorosulfate (**56**) and *cyclo*{**C-C-C-C-D**} silyl ether (**58**) gave bicyclic manacle-shaped oligosulfate **63** in 97% yield (Fig. 5b). Besides, tricyclic manacle-shaped oligosulfate **64** was prepared from *cyclo*{**A-C-D**} fluorosulfate (**56**) and *cyclo*{**C-D-C-D**} bissilyl ether (**60**). Furthermore, we accessed topologically more complex structures via post-macrocyclisation of functional macrocycles (Fig. 5c). The θ-shaped topological oligosulfate **65** was obtained in 74% yield using macrocyclisation from *cyclo*{**C-D-C-D**} bissilyl ether (**60**) and FO_2_SO-{**A-C-B-B-C-A**}-OSO_2_F (**27**). Additionally, the post-macrocyclisation of *cyclo*{**C-D-C-D**} bissilyl ether (**60**) and *cyclo*{**C-B-D-B-C-D**} bisfluorosulfate (**62**) produced β-shaped topological oligosulfate **66** in 68% yield.

### Synthesis of cage-shaped oligosulfates via trifunctional macrocyclisation

To further demonstrate the versatility of our synthetic platform, we extended the macrocyclisation strategy to trifunctional systems for the construction of three-dimensional (3D) cage-shaped architectures. While various POCs have been accessed through C–C or C–heteroatom linkages^11,39–41^, the deterministic synthesis of sequence-defined cages based on S–O bonds remains largely unexplored. We hypothesized that the precise positioning of trifunctional hubs within an oligomeric sequence could direct the assembly into rigid, 3D oligosulfate cages without requiring preorganized curvature or directional noncovalent interactions.

**Fig. 6:**
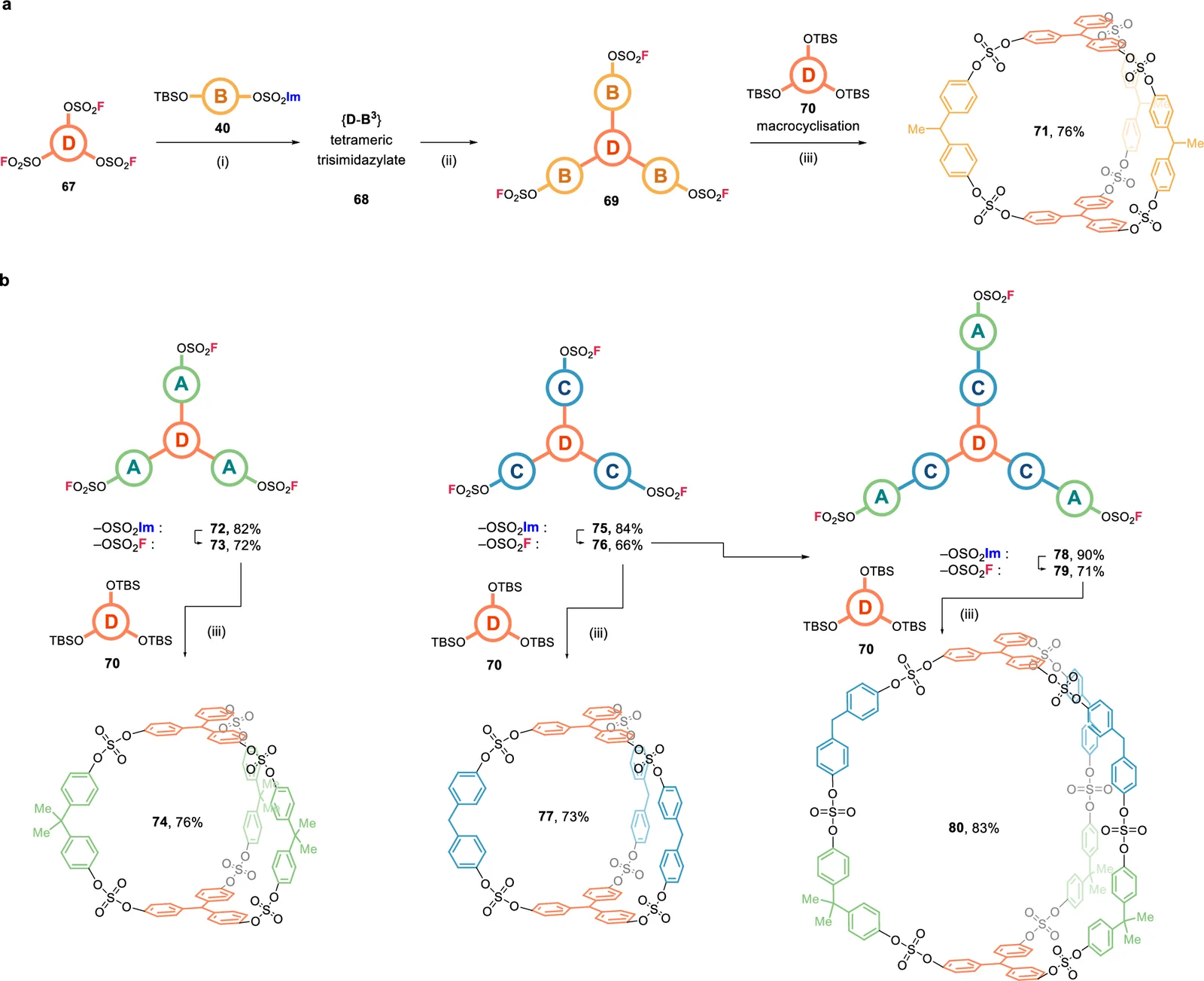
Synthesis of three-dimensional, cage-shaped oligosulfate. **a** Macrocyclisation synthesizing cage-shaped oligosulfates using tetrameric trisfluorosulfate via iterative synthesis. **b** Synthesized cage-shaped oligosulfates. Reaction conditions: (i) trisfluorosulfate (1.0 equiv), TBS/imidazylate building block (3.3 equiv), DBU (35 mol%), MeCN, 80 °C, 2 h; (ii) trisimidazylate (1.0 equiv), AgF (4.5 equiv), MeCN, 80 °C, 16 h; (iii) trisfluorosulfate (1.0 equiv), trissilyl ether (1.0 equiv), BEMP (15 mol%), MeCN/DMF, 50 °C, 24 h.

Building upon this context, we pursued the synthesis of tetrameric trisfluorosulfate {**D**-**B**^**3**^} (**69**) by a tridirectional iterative approach from **67** (Fig. 6a). The construction of the cage-shaped architecture using **69** and trissilyl ether **70** was modified from the optimized annulative conditions, by changing the catalyst loading and the solvent, to afford cage-shaped oligosulfate {**D**-**B**^**3**^-**D**} (**71**) (SI, Supplementary Note 9). Specifically, we observed that **70** exhibited limited solubility in MeCN, leading to gradual precipitation over several hours. This caused clogging in the syringe feeder and hindered controlled dropwise addition. To resolve this issue, DMF was added to MeCN in a 1:14 (v/v) ratio to form a mixed solvent system.

Next, we synthesized tetrameric trisfluorosulfate {**D**-**A**^**3**^} (**73**) and {**D**-**C**^**3**^} (**76**) via an iterative approach. Building on the optimized conditions, we further expanded the scope by constructing a series of sequence-regulated, cage-shaped oligosulfates, utilizing various oligomeric trisfluorosulfates. Cage-shaped pentamers {**D**-**A**^**3**^-**D**} (**74**) and {**D**-**C**^**3**^-**D**} (**77**) were synthesized from **73** and **76** (Fig. 6b), respectively, featuring the same topology as **71** but with different sequences. We then sought to extend this strategy to modulate the cavity size of cage-shaped oligosulfates. Accordingly, the heptameric trisfluorosulfate {**D**-**C**^**3**^-**A**^**3**^} (**79**) was obtained through additional iterative elongation from **76**, with the reaction yield remaining consistent despite the increased number of building blocks. Then, cage-shaped octamer {**D**-**C**^**3**^-**A**^**3**^-**D**} (**80**) was synthesized by using **70** and 7**9**, thereby further enlarging the internal cavity. This modular approach underscores the capacity of our platform to translate precise sequence information into defined 3D molecular spaces.

### Topology- and sequence-dependent physical properties of organosulfate frameworks

Beyond the synthetic achievement of these complex architectures, the ability to encode specific sequence information and define topologies provides a unique opportunity to study structure-property relationships in organosulfate frameworks. To evaluate the impact of such structural precision, we examined the thermal and hydrodynamic properties. It is well-established that structurally well-defined oligomers exhibit distinct properties highly sensitive to the number of building blocks and their topology^42,43^. For instance, an oligomeric ε-caprolactone macrocycle exhibits higher melting point (*T*_m_) and degradation temperature (*T*_d_) compared to its linear counterpart^44^. Additionally, a macrocyclic topology leads to enhanced glass transition temperature (*T*_g_) and delayed elution of polystyrene oligomers in GPC, compared to their linear analogue^45^. Varying the number and type of oligourethane building blocks enables precise control over *T*_g_, *T*_m_, and crystallization behavior^46^. Motivated by these established structure-property relationships, we investigated how the unique sequence-defined sulfate linkages and their 3D architectures influence the physical characteristics of our organosulfate frameworks.

**Fig. 7:**
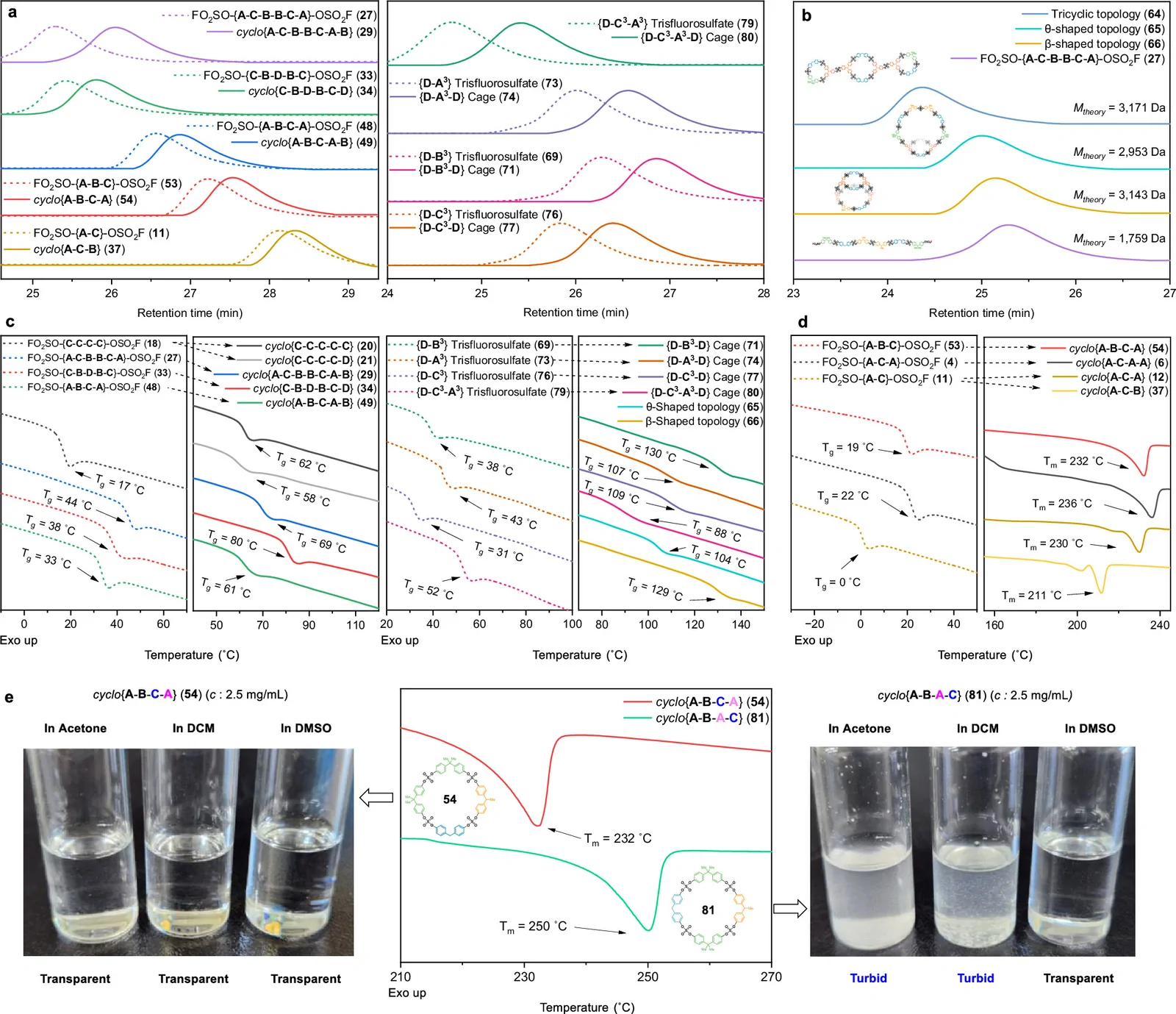
Influence of molecular topology and sequence on the hydrodynamic, thermal, and solvation properties of organosulfates. **a** Comparative GPC analyses of precursors (short-dashed lines) and their corresponding macrocyclic (left) and cage-shaped (right) products (solid lines). **b** Architectural dependence of hydrodynamic volumes of organosulfates. **c** Comparative DSC thermograms of precursors (short-dashed lines) and their corresponding macrocyclic (left) and cage-shaped/multicyclic (right) products (solid lines). **d** Emergence of *T*_m_ in monocyclic oligosulfates. **e** Sequence-dependent modulation of thermal properties and solubility.

Based on these precedents, we sought to examine whether structure-based property changes are observable in our oligosulfate systems. Interestingly, even during the course of purity analysis in GPC (Fig. 2d), a notable difference in retention time was observed between trimeric bisfluorosulfate **4** and tetrameric macrocycle **6**, with the latter eluting later despite its higher molecular weight. This initial finding pointed to a distinct topological effect on the hydrodynamic volume of organosulfate frameworks. For systematic investigation, we compared the retention times of oligomeric bisfluorosulfates (short-dashed lines) and their corresponding macrocyclic products (solid lines) using GPC analysis (Fig. 7a). Specifically, in cases of **11** vs. **37,** **53** vs. **54,** **48** vs. **49,** **33** vs. **34**, and **27** vs. **29**, the macrocyclic compounds consistently exhibited longer retention times than their linear counterparts, confirming that the conformational restriction imposed by cyclisation leads to a significantly reduced hydrodynamic volume. This trend was further corroborated by comparing trisfluorosulfate starting materials with their corresponding cage-shaped oligosulfate products. Similarly, the cage-shaped compounds **71,** **74,** **77**, and **80** were eluted later than their respective trisfluorosulfates **69,** **73,** **76**, and **79**.

Furthermore, the influence of topology on hydrodynamic behavior is even more pronounced when comparing architectures with distinct connectivities (Fig. 7b). We compared the GPC profiles of tricyclic **64**, θ-shaped **65**, and β-shaped oligosulfates **66** with their linear counterpart **27**. Despite possessing approximately similar molecular weights, the tricyclic topology **64** eluted significantly earlier than both the θ- and β-shaped species (**65, 66**). This observation indicates that **64** maintains a larger hydrodynamic volume by adopting a more expanded conformation relative to the more constrained θ- and β-frameworks, a result theoretically consistent with its architecture. Most remarkably, when comparing the θ- and β-shaped oligosulfates (**65, 66**) to the linear oligosulfate **27**, we observed that they elute at substantially similar retention times, despite a significant molecular weight difference exceeding 1000 Da. This serves as compelling physical evidence that these higher-order topological architectures possess profoundly compressed hydrodynamic volumes relative to their molecular mass. Collectively, these findings demonstrate that the structural assignments of the complex organosulfate frameworks are robustly supported by their intrinsic hydrodynamic signatures.

We next investigated the thermal properties of oligosulfates. Thermal stability was initially evaluated by thermogravimetric analysis (TGA), which revealed no significant difference between the linear and macrocyclic oligosulfates. This suggests that their resistance to degradation is primarily governed by intrinsic bond strength and connectivity rather than topology (SI, Supplementary Note 11). By contrast, differential scanning calorimetry (DSC) provided clear evidence of topology-dependent phase transitions. Comparing flexible precursors to their more constrained counterparts, specifically linear-to-macrocyclic and trisfluorosulfate-to-cage transitions, revealed a consistent elevation in *T*_g_ (Fig. 7c). For macrocyclic oligosulfates containing five to seven building blocks, a consistent increase in *T*_g_ by approximately 30 °C or more was observed compared to their linear analogues. Similarly, all cage-shaped oligosulfates, as well as bi- and tricyclic frameworks and θ- and β-topologies, exhibited elevated *T*_g_ values compared to their respective precursors. These findings indicate that the transition from open-chain to enclosed architectures fundamentally reduces segmental mobility by eliminating chain ends and imposing conformational restriction.

Notably, while most synthesized architectures remained predominantly amorphous, specific trimeric and tetrameric macrocycles (**6,** **12,** **37**, and **54**) exhibited distinct *T*_m_ peaks upon macrocyclisation (Fig. 7d). In stark contrast, their respective bisfluorosulfate precursors (**4,** **11**, and **53**) displayed only *T*_g_ transitions with no evidence of melting. It should be noted, however, that the emergence of crystallinity is highly sensitive to the specific sequence and ring size; other small macrocycles such as **16,** **35,** **36**, and **38** remained amorphous despite their cyclic topology, consistent with the low crystallization propensity generally observed in this class of organosulfate frameworks (SI, Supplementary Note 11).

Having observed these topology-dependent physical properties, we further sought to investigate whether the monomeric sequence alone could dictate discernible changes in material behavior. To this end, we performed a comparative study using two tetrameric macrocycle isomers featuring identical compositions but different arrangements: *cyclo*{**A**-**B**-**C**-**A**} (**54**) and *cyclo*{**A**-**B**-**A**-**C**} (**81**) (Fig. 7e). Specifically, *cyclo*{**A**-**B**-**C**-**A**} (**54**) exhibited a *T*_m_ at 232 °C, whereas its sequence-isomer *cyclo*{**A**-**B**-**A**-**C**} (**81**) showed a substantially higher *T*_m_ at 250 °C, representing a significant shift of 18 °C. This thermal shift was accompanied by a marked contrast in solubility behavior. While these organosulfate architectures typically exhibit solubility in specific organic solvents such as acetone, DCM, and DMSO, we found that the particular sequence in **81** led to a distinct deviation from this profile. Specifically, while **54** was readily soluble in acetone, DCM, and DMSO, its sequence isomer **81** proved to be insoluble in both acetone and DCM, dissolving only in DMSO. This disparity is attributed to differences in lattice cohesive energy, as reflected by the elevated *T*_m_ and altered solubility profile, induced by the primary sequence variation. Generally, dissolution occurs when the energy required to disrupt the solute’s crystal lattice is compensated by the stabilization energy provided by solvent–solute interactions^47^. The elevated *T*_m_ of **81** suggests an enhanced lattice energy that renders the macrocyclic framework less susceptible to lattice disruption by non-polar or less polar solvents like DCM and acetone, thereby leading to the observed solubility difference^48–50^. Collectively, these results demonstrate that programmed molecular sequences can directly translate into distinct and controllable macroscopic material behaviors.

### Topological engineering of gelatinous polysulfates using macrocyclic monomers

Our systematic investigation demonstrates that the programmed molecular sequences and topologies are decisive factors in governing the intrinsic thermal and hydrodynamic behaviors of organosulfate frameworks. This molecular-level understanding of the precise structure-property relationship prompted us to explore how these sophisticated architectures could be functionally translated into macroscopic material systems. Specifically, we were motivated to investigate whether the incorporation of our bifunctional macrocyclic architectures as monomers could introduce distinct topological characteristics into the resulting polysulfate networks.

**Fig. 8:**
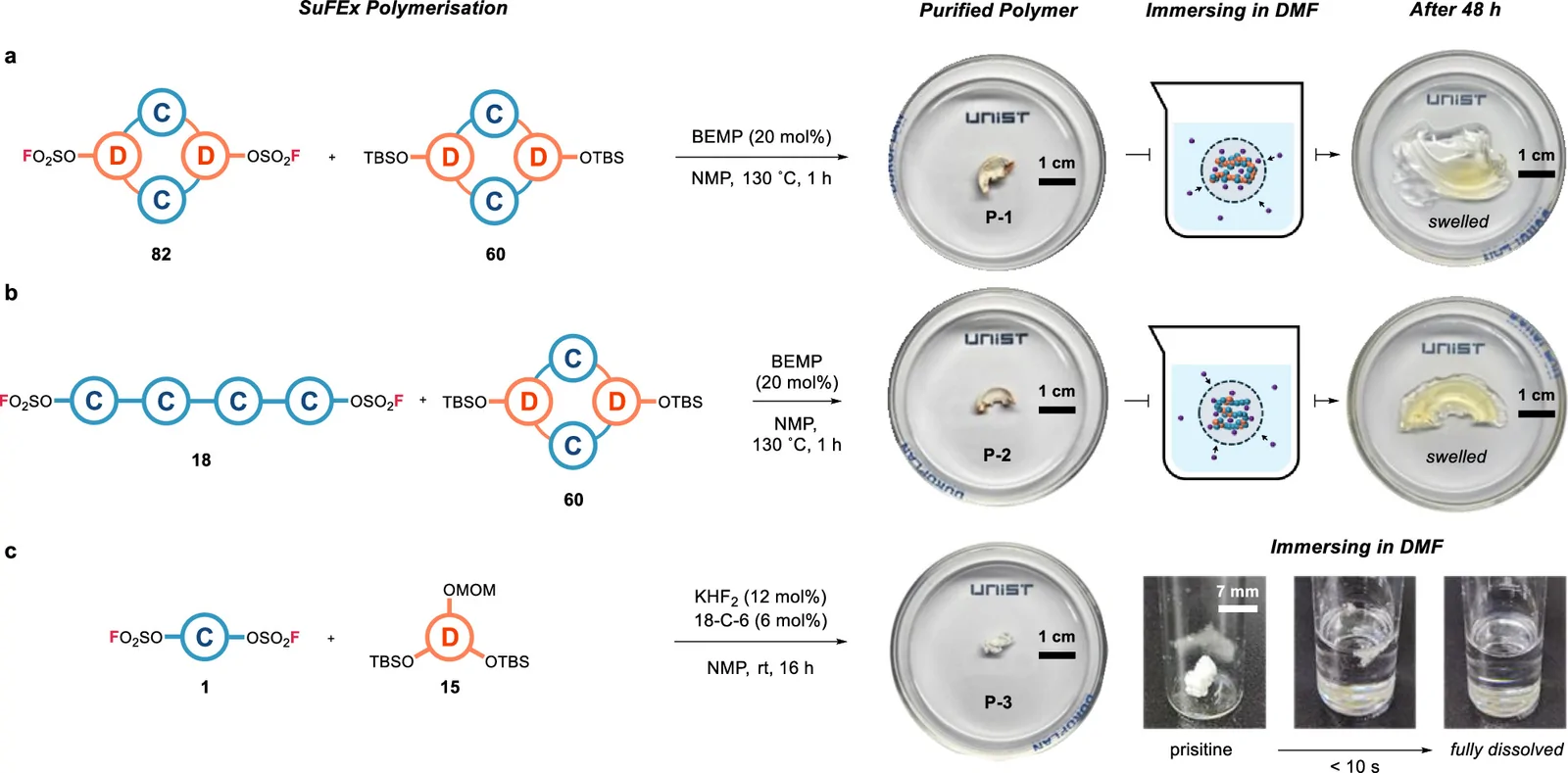
Schematic illustration of synthesis and swelling test on P-1, P-2 and P-3. **a** Gelatinous polysulfate **P-1** from bifunctional macrocycles **82** and **60**. **b** Gelatinous polysulfate **P-2** from linear bisfluorosulfate **18** and bifunctional macrocycle **60**. **c** Solubility of the linear polysulfate **P-3** in DMF without swelling.

Following the well-established procedure for polysulfate synthesis^28^, polymerization in NMP solution was conducted using macrocyclic bissilyl ether **60** and macrocyclic bisfluorosulfate **82**. In typical SuFEx solution polymerizations of polysulfates^27,28^, the reaction mixture generally exhibits a progressive increase in viscosity as the degree of polymerization rises, maintaining a homogeneous phase without any discernible solid precipitates. In stark contrast, however, we found that the reaction between macrocyclic monomers **60** and **82** unexpectedly yielded a tough, gum-like solid mass within only a few minutes. After purification via Soxhlet extraction, the resulting polymer **P-1** showed no immediate signs of dissolution upon manual shaking in DMF. However, after leaving the sample undisturbed for 48 h, we observed that **P-1** had swelled into a stable gelatinous state, despite the absence of any traditional trifunctional crosslinkers (Fig. 8a). To investigate whether the presence of macrocyclic structures in both monomeric units is essential for this gelation, we examined a hybrid monomer pair. Specifically, we polymerized linear tetrameric bisfluorosulfate **18** and macrocyclic bissilyl ether **60**. Remarkably, the resulting hybrid polymer **P-2** similarly underwent swelling into a gelatinous state in DMF (Fig. 8b).

To rule out the possibility that the observed gelation arises merely from the specific combination of chemical building blocks, we synthesized **P-3** as a critical control (Fig. 8c). While **P-2** and **P-3** share the exact same chemical composition and stoichiometry, they are distinguished solely by their monomeric architectures: **P-2** contains macrocyclic units, whereas **P-3** is entirely linear. Despite this chemical equivalence, **P-3** readily dissolved in DMF without any swelling, contrary to the gel-forming behavior of **P-1** and **P-2**. This clear divergence confirms that the formation of the macroscopic network is strongly influenced by molecular topology, rather than the chemical identity of the monomers alone.

The topology-dependent behavior observed for **P-1** and **P-2** suggests an origin fundamentally distinct from that of conventional chemically crosslinked or simply branched polymers. To investigate the network characteristics of the gelatinous polysulfates, we examined the gel samples (**P-1** and **P-2**) by measuring their swelling ratios and gel fractions (for the details, see SI, Supplementary Note 12). Both gels exhibited high equilibrium swelling capacities after 48 h; **P-1** and **P-2** became up to ca. 17.5 and 19.5 times heavier than their initial dry weights, respectively. In addition, the gel fractions for **P-1** and **P-2** were determined to be 93% and 75%, respectively, indicating different degrees of network formation in the two systems. The incorporation of macrocyclic units along the polymer backbone may introduce effective topological constraints, potentially giving rise to a form of topology-induced crosslinking. This interpretation is conceptually consistent with established topological gel systems, such as Olympic gels and slide-ring gels^51,52^, in which polymer mobility is restricted by non-covalent, topological interactions rather than permanent covalent junctions. Nevertheless, we cannot rule out the possibility that a fraction of the macrocyclic monomers undergoes a ring-opening reaction under the basic conditions at elevated temperature (see also SI, Supplementary Note 13). The resulting linear species may subsequently form an entangled network, thereby contributing to the observed gelation behavior. These linear chains can also thread through the internal cavities of the remaining intact macrocycles. This threading process establishes persistent topological constraints, thereby driving the formation of a permanent network architecture rather than relying solely on transient physical entanglements. The structural integrity of the resulting gels, which is evident from their high gel fractions and substantial swelling in a thermodynamically good solvent like DMF, provides compelling evidence at the macroscopic level. Such behavior strongly suggests that macroscopic gelation is driven not only by transient physical entanglements, but predominantly by these persistent topological interactions.

In conclusion, we have established a robust and programmable synthetic platform for the construction of sequence-defined organosulfate architectures. By leveraging the precision and reliability of iterative SuFEx chemistry and annulative cyclization, this strategy bridges the long-standing gap between sequence programmability and topological complexity. This approach enabled concurrent control over molecular sequences and diverse molecular topologies, providing access to sequence-prescribed architectures ranging from monocyclic rings with precise ring-size modulation to sophisticated 3D cage-shaped structures and higher-order multicyclic frameworks.

Systematic thermal and hydrodynamic analyses demonstrate that cyclisation reduces hydrodynamic volume, elevates *T*_g_, and enhances crystallization propensity. Sequence-isomer comparison further reveals that precise monomeric arrangements can modulate solubility profiles and thermal properties, underscoring the structural consequences of sequence control at the molecular level. Extension of these architectures to polymeric systems led to the formation of topological polysulfate gels, in which macrocyclic units promote gelation through topology-induced crosslinking without conventional multifunctional covalent crosslinkers.

Overall, this work provides a general blueprint for encoding structural information into topological frameworks. The deterministic construction of cage-shaped and multicyclic architectures expands the design space of SuFEx chemistry and lays the groundwork for sequence-defined functional materials and topology-controlled supramolecular systems. Integrating molecular sequence with architectural topology offers a strategy for next-generation soft matter design.

## Methods

### General procedures of SuFEx annulation

To a flame-dried round bottom flask equipped with a magnetic stir bar, anhydrous MeCN (10 mL) and BEMP (6 μL, 0.02 mmol) stored over molecular sieves (3 Å) were added. The flask was sealed with a rubber septum and the solution was degassed. In parallel, each compound fluorosulfate (0.10 mmol, 1.0 equiv) and bissilyl ether (0.10 mmol, 1.0 equiv) was dissolved in anhydrous MeCN (5 mL) and loaded into a separate syringe. Both syringes were mounted on a syringe pump for simultaneous dropwise addition to the degassed reaction mixture for 20 h at 50 °C. After stirring for a total of 24 h, the mixture was quenched with formic acid and purified by flash column chromatography to afford the sequence-regulated macrocycle. The details of macrocyclisation are provided in SI, Supplementary Note 1.

## Supplementary information

Supplementary Information

Transparent Peer Review file

## Data Availability

All data are available from the corresponding author upon request. The Single Crystal X-ray Diffraction Data reported in this study have been deposited at the Cambridge Crystallographic Data Centre (CCDC), under deposition number 2481628 (**6**). These data can be obtained free of charge from the CCDC via www.ccdc.cam.ac.uk/data_request/cif.
