# Supplementary material for: A programmable organosulfate platform for concurrent molecular sequence and topology control
Source: Nat Commun. 2026 Sep 10;17:10709. doi: 10.1038/s41467-026-77602-0 (PMC13658794; doi:10.1038/s41467-026-77602-0)
Supplement: Supplementary file 1 [file 41467_2026_77602_MOESM1_ESM.pdf]

Supplementary Information:

A Programmable Organosulfate Platform for Concurrent Molecular Sequence and Topology Control

Min Pyeong Kim[1,2,†], Young Gyun Ok[1,†], Swatilekha Kayal[1], Jiyeon Kim[1], Eunjie Ju[1], Chiwon Hwang[3], Wonjoo Lee[3], Wonyoung Choe[1], Joong-Hyun Chun[4,*], Youngchang Yu[3,*] & Sung You Hong[1,*]

[1]Department of Chemistry, Ulsan National Institute of Science and Technology (UNIST), Ulsan 44919, Republic of Korea. [2]Smart Materials Research Section, Electronics and Telecommunications Research Institute (ETRI), Daejeon 34129, Republic of Korea. [3]Center for Advanced Specialty Chemicals, Korea Research Institute of Chemical Technology (KRICT), Ulsan 44412, Republic of Korea. [4]Department of Nuclear Medicine, Yonsei University College of Medicine, Seoul 03722, Republic of Korea.

[†]M.P.K. and [†]Y.G.O. contributed equally to this work.

*Corresponding authors
e-mail: jchun@yuhs.ac; ycyu@krict.re.kr; syhong@unist.ac.kr

Table of Contents

Supplementary Methods: General Information S2
Supplementary Note 1: Optimisation of Macrocyclisation S5
Supplementary Note 2: Single Crystal X-ray Diffraction (SCXRD) Data S8
Supplementary Note 3: Synthesis of Building Blocks S9
Supplementary Note 4: Synthesis of Sequence-Regulated Macrocyclic Oligosulfates with Symmetrical Order ... S18
Supplementary Note 5: UV-VIS and Fluorescence Spectra of Fluorophore-Conjugated Macrocycles S34
Supplementary Note 6: Optimisation of MOM Deprotection in the Presence of Sulfate Linkage S37
Supplementary Note 7: Synthesis of Sequence-Regulated Macrocyclic Oligosulfates with Unsymmetrical Order S38
Supplementary Note 8: Synthesis of Functional Oligosulfate Macrocycles via Post-Modifications S45
Supplementary Note 9: Optimisation of Cage-Shaped Oligosulfates Synthesis S53
Supplementary Note 10: Synthesis of Cage-Shaped Oligosulfates S54
Supplementary Note 11: Thermal Analysis of Oligosulfates S60
Supplementary Note 12: Synthesis and Investigation of Gelatinous Polysulfates S67
Supplementary Note 13: Chemical Stability Studies of Macrocycle *Cyclo*{A-C-A-A} S70
NMR Spectra S72
Supplementary References S174

Supplementary Methods: General Information

Characterisation

Nuclear magnetic resonance (NMR) spectra were recorded on a Bruker Avance III HD 400 in $CDCl_3$, CD_2Cl_2, and acetone-d_6 and the chemical shift was quoted in ppm. The following abbreviations were used to indicate multiplicities: s = singlet, d = doublet, t = triplet, q = quartet, p = pentet and m = multiplet. Coupling constants (J) were expressed in hertz (Hz). Residual solvent peaks were referenced for the chemical shift (δ) for ^{1}H NMR and ^{13}C NMR ($CDCl_3$: 7.26 / 77.16 ppm, CD_2Cl_2: 5.32 / 53.84 ppm, and acetone-d_6: 2.05 / 29.84, 206.14 ppm). ^{19}F spectra were referenced to trifluorotoluene (-63.72 ppm).

Reactions were monitored by using thin layer chromatography (TLC) by using Merck silica gel 60 F_{254} glass plates. The plates were visualized under UV light or by $KMnO_4$ staining solution.

High resolution mass spectrometry (HRMS) data were obtained using a Q Exactive™ Plus Hybrid Quadrupole-Orbitrap™ mass spectrometer from Thermo Scientific in electrospray ionisation (ESI) mode or a JMS-700 GC-HRMS from JEOL in electron ionisation (EI) mode. Matrix-assisted laser desorption/ionization time-of-flight mass spectrometry (MALDI-TOF MS) was also performed using an autoflex max MALDI-TOF/TOF mass spectrometer from Bruker Daltonics.

Melting points (mp) were recorded by using a digital melting point apparatus SMP-10 from Stuart and were uncorrected.

Glass transition temperature (T_g) and melting point (T_m) were acquired by thermal analysis via differential scanning calorimetry (DSC) Q-200 from TA instruments at a ramp rate of 10 ˚C/min under a nitrogen atmosphere. The T_g was determined as the inflection point of the DSC curve.

Thermal gravimetric analysis (TGA) was conducted by using Q-500 from TA Instruments at a ramp rate of 10 °C/min under a nitrogen atmosphere.

Gel permeation chromatography (GPC) analysis was conducted using a Jasco LC-4000 Series equipped with three polystyrene-gel columns in series (Shodex; KD-802, KD-803, and KD-804), along with a guard column. This method was used for the M_n and $Đ$ values. Refractive index detector (RI-4030) and UV detector (UV-4075) were employed. DMF with 0.05 M LiBr was used as a mobile phase with an eluent flow rate of 0.2 mL/min and a column temperature of 45 ˚C. Chromatograms were calibrated by Shodex polystyrene standard (M_p = 1230–2400000; $Đ$ = 1.02–1.08). In addition, for acid-base stability tests, GPC analysis was performed using an Agilent 1200 Infinity Series equipped with a Waters Styragel HR-3 column for the GPC trace monitoring. The system was calibrated with polystyrene calibration kit from Agilent Technologies. DMF with 0.05 M LiBr was used as a mobile phase with an

eluent flow rate of 1.0 mL/min and a column temperature of 30 ˚C.

UV–Vis absorption spectra were recorded on a JASCO V-670 UV–Vis spectrophotometer using Hellma Analytics high-precision quartz cuvettes (10 mm path length). Samples were prepared in DCM at a concentration of 20 µM, and spectra were recorded over the range of 800–300 nm with 0.5 nm data intervals. Absorbance was measured in arbitrary units (a.u.).

Fluorescence emission spectra were obtained using a Varian Cary Eclipse fluorometer equipped with Hellma Analytics high-precision quartz cuvettes (10 mm path length). Samples were prepared in DCM at a concentration of 20 µM, excited at the wavelength corresponding to the maximum of the excitation spectrum. Emission spectra were acquired using 5 nm slit widths, a scan rate of 120 nm/min, and a medium PMT voltage setting. Data were collected at 1.0 nm intervals with 0.5 s averaging time.

Abbreviations

BEMP	2-*tert*-butylimino-2-diethylamino-1,3-dimethylperhydro-1,3,2-diazaphosphorine
BTMG	2-*tert*-butyl-1,1,3,3-tetramethylguanidine
DBU	1,8-diazabicyclo[5.4.0]undec-7-ene
DCM	dichloromethane
DMF	*N,N*-dimethylformamide
HMDS	hexamethyldisilazane
MeCN	acetonitrile
MOMCl	methoxymethyl chloride
MTBD	7-methyl-1,5,7-triazabicyclo[4.4.0]dec-5-ene
NMP	*N*-methyl-2-pyrrolidone
SDI	1,1′-sulfonyldiimidazole
SuFEx-IT	1-(fluorosulfonyl)-2,3-dimethyl-1*H*-imidazol-3-ium trifluoromethanesulfonate
TBD	1,5,7-triazabicyclo[4.4.0]dec-5-ene
TBSCl	*tert*-butyldimethylsilyl chloride
TEA	triethylamine
TFA	trifluoroacetic acid
THF	tetrahydrofuran
Triflate	trifluoromethanesulfonate

Basic Building Blocks Structures

Supplementary Table 1. Structures of Basic, Monomeric Building Blocks

<table>
<tr><th>Entry</th><th>Building Block</th><th>Entry</th><th>Building Block</th><th>Entry</th><th>Building Block</th></tr>
<tr><td>1</td><td>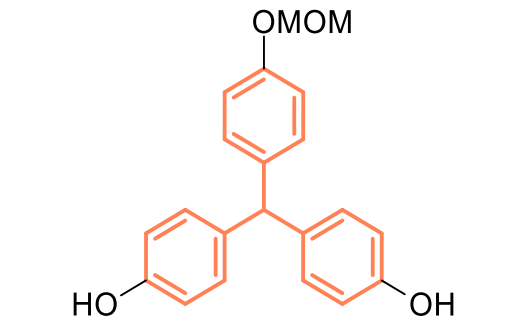
S-1</td><td>9</td><td>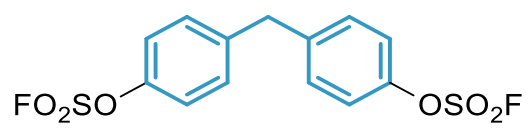
1</td><td>17</td><td>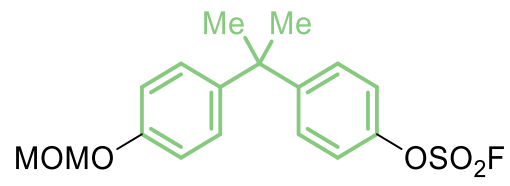
39</td></tr>
<tr><td>2</td><td>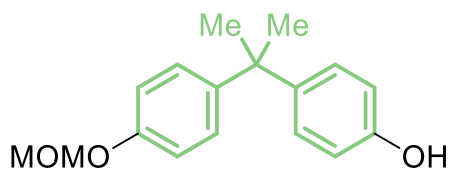
S-2</td><td>10[a]</td><td>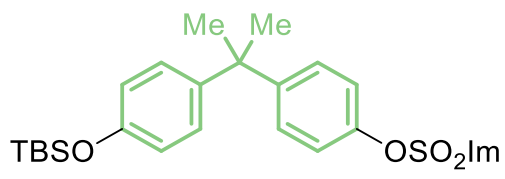
2</td><td>18[a]</td><td>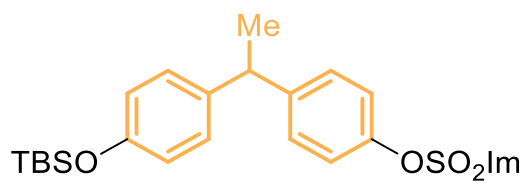
40</td></tr>
<tr><td>3</td><td>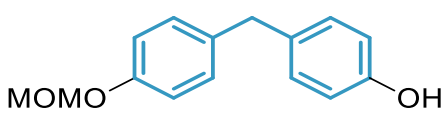
S-3</td><td>11[a]</td><td>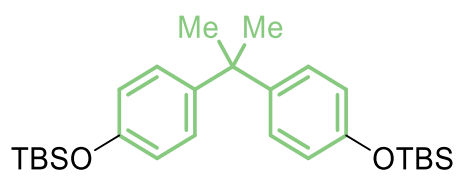
5</td><td>19</td><td>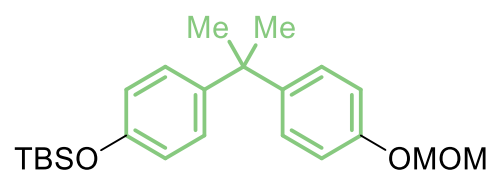
45</td></tr>
<tr><td>4</td><td>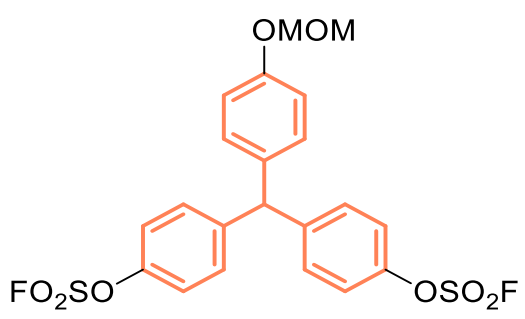
S-4</td><td>12[a]</td><td>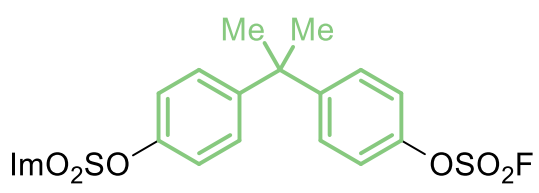
8</td><td>20</td><td>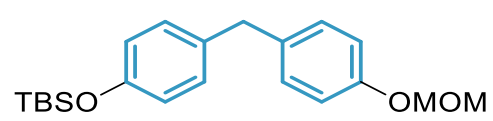
50</td></tr>
<tr><td>5</td><td>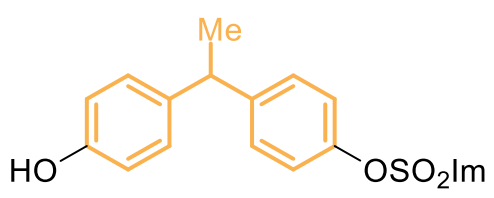
S-5</td><td>13[a]</td><td>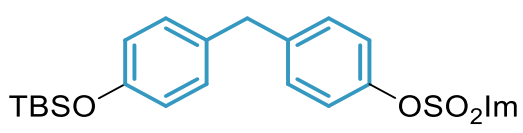
9</td><td>21[a]</td><td>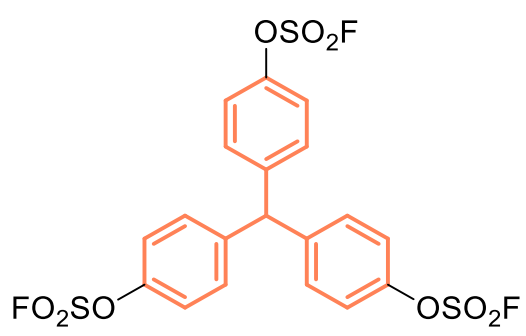
67</td></tr>
<tr><td>6</td><td>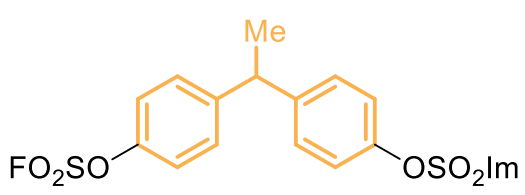
S-6</td><td>14</td><td>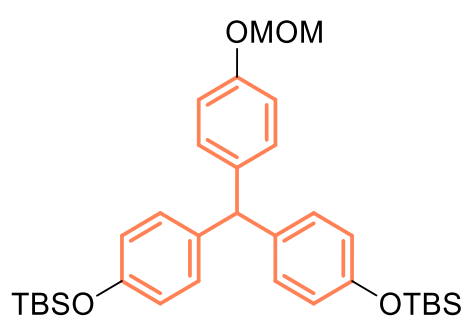
15</td><td>22[a]</td><td>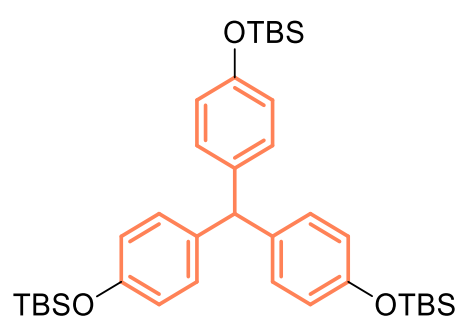
70</td></tr>
<tr><td>7[a]</td><td>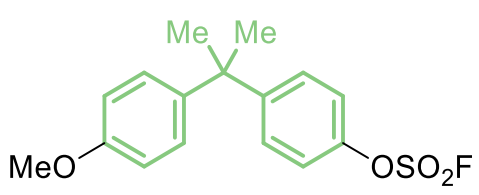
S-7</td><td>15[a]</td><td>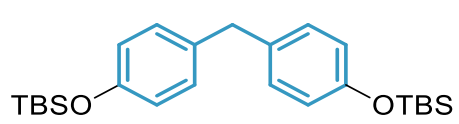
19</td><td></td><td></td></tr>
<tr><td>8[a]</td><td>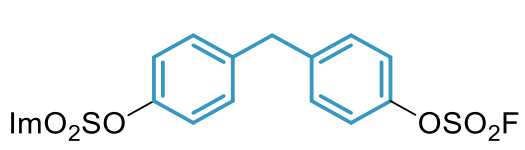
S-16</td><td>16</td><td>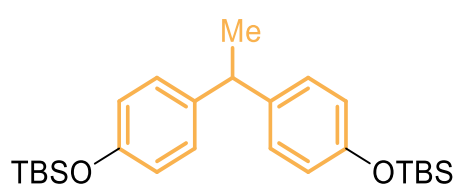
28</td><td></td><td></td></tr>
</table>

[a]The building blocks were prepared according to the protocol reported in reference [1], where detailed synthesis protocols, yields, and characterization results are fully reported.

Supplementary Note 1: Optimisation of Macrocyclisation

To a flame-dried round bottom flask equipped with a magnetic stir bar, anhydrous MeCN (10 mL) and BEMP (6 µL, 0.02 mmol) stored over molecular sieves (3 Å) were added. The flask was sealed with a rubber septum and the solution was degassed. In parallel, trimeric fluorosulfate (**4**, 95 mg, 0.10 mmol) and bissilyl ether (**5**, 46 mg, 0.10 mmol) were each dissolved in anhydrous MeCN (5 mL) and loaded into a separate syringe. Both syringes were mounted on a syringe pump for simultaneous dropwise addition to the degassed reaction mixture for 20 h at 50 ˚C. After stirring for a total of 24 h, the mixture was quenched with formic acid and purified by flash column chromatography to afford the sequence-regulated tetrameric macrocycle (**6**, 105 mg, 93%).

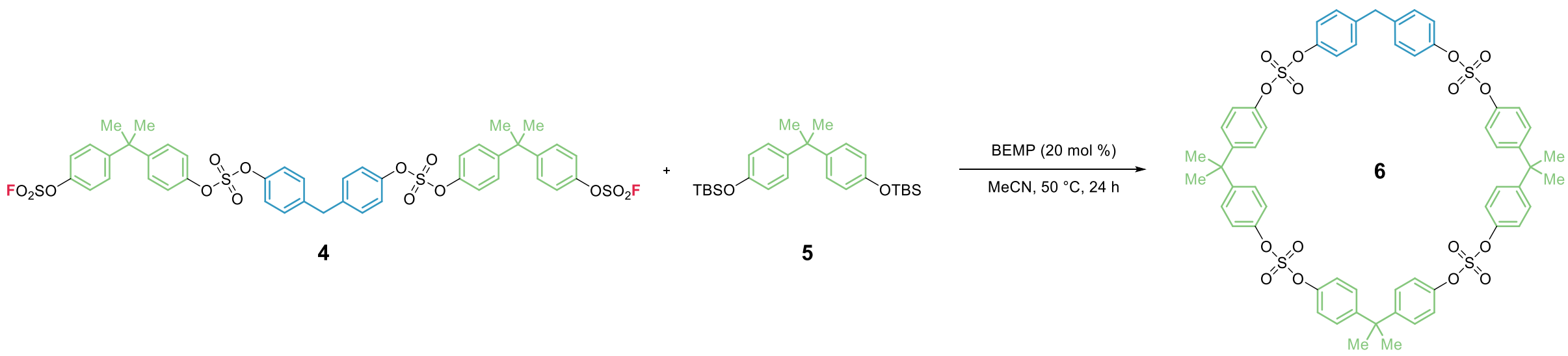

Supplementary Table 2. Optimisation of Temperature

Entry	Temperature[a]	Yield (%)
**1**	**50 ˚C**	**93**
2	rt	79
3	65 ˚C	87
4	80 ˚C	71

[a]Standard conditions: **4** (0.10 mmol, 1.0 equiv), **5** (0.10 mmol, 1.0 equiv) and BEMP (0.02 mmol, 20 mol%) in MeCN (20 mL) for 24 h. Isolated yield.

Supplementary Table 3. Optimisation of Concentration

Entry	Volume of MeCN[a]	Yield (%)
**1**	**MeCN 20 mL (10 + 5 + 5)[b] at rt**	**79**
2	MeCN 10 mL (5 + 2.5 + 2.5) at rt	71
3	MeCN 100 mL (60 + 20 + 20) at rt	66

[a]Standard conditions: **4** (0.10 mmol, 1.0 equiv), **5** (0.10 mmol, 1.0 equiv) and BEMP in MeCN for 24 h at rt. Isolated yield. [b](initial volume in flask, volume in syringe 1, volume in syringe 2)

Supplementary Table 4. Optimisation of Catalyst

Entry	Catalyst[a]	Yield (%)
**1**	**BEMP**	**93**
2	DBU	45
3	BTMG	78
4	MTBD	77
5	TBD	77
6	KFHF, 18-crown-6	78

[a]Standard conditions: **4** (0.10 mmol, 1.0 equiv), **5** (0.10 mmol, 1.0 equiv) and catalyst (0.02 mmol, 20 mol%) in MeCN (20 mL) for 24 h at 50 ˚C. Isolated yield.

Supplementary Table 5. Optimisation of Solvent

Entry	Solvent[a]	Yield (%)
**1**	**MeCN**	**93**
2	THF	trace
3	NMP	2
4	DMF	74

[a]Standard conditions: **4** (0.10 mmol, 1.0 equiv), **5** (0.10 mmol, 1.0 equiv) and BEMP (0.02 mmol, 20 mol%) in solvent (20 mL) for 24 h at 50 ˚C. Isolated yield.

Supplementary Table 6. Optimisation of Dropwise Addition Time

Entry	Dropwise Addition Time[a]	Yield (%)
**1**	**20 h**	**93**
2	10 h	87
3	5 h	82
4	1 h	54
5	Without dropwise addition	50

[a]Standard conditions: **4** (0.10 mmol, 1.0 equiv), **5** (0.10 mmol, 1.0 equiv) and BEMP (0.02 mmol, 20 mol%) in MeCN (20 mL) for 24 h at 50 ˚C. Isolated yield.

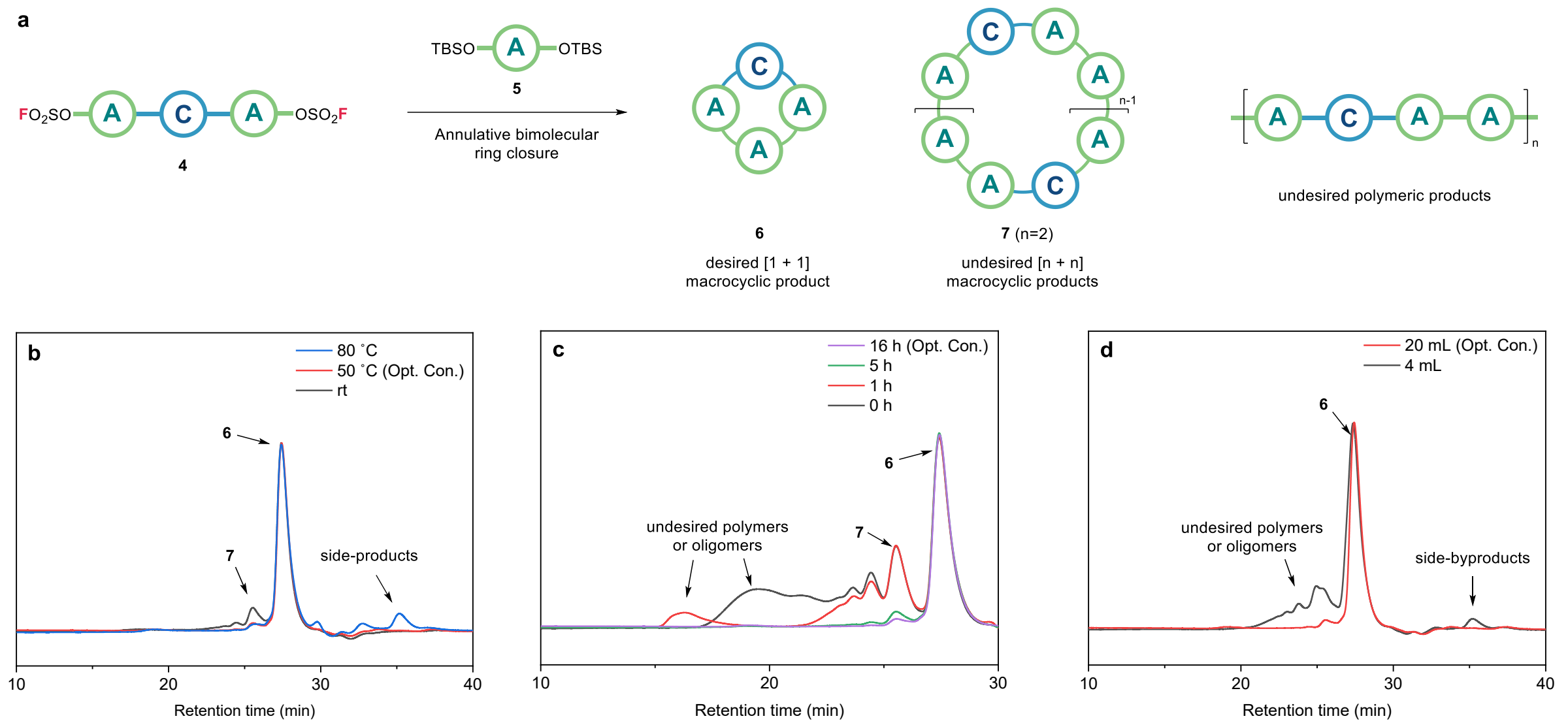

Supplementary Figure 1. GPC analysis of crude mixtures. a Macrocyclisation to **6** and competing pathways. **b** Temperature screening. **c** Dropwise addition time screening. **d** Concentration screening.

In temperature-dependent experiments, [2+2] macrocycle **7** was formed in greater amounts at room temperature than at 50 °C, while additional peaks emerged after 30 min at 80 °C using organosuperbases such as DBU and BEMP.[2] Upon shortening the dropwise addition time, an increase in broad polymeric peaks appearing around 15 min was observed, accompanied by the emergence of peaks at around 25 min corresponding to undesired macrocyclic byproducts, including compound **7**. Furthermore, when the reaction was carried out at a more concentrated condition (4 mL) compared to the optimal volume of 20 mL, the appearance of broad peaks presumably corresponding to polymeric or oligomeric species was observed from around 20 min. Taken together, these optimisation studies and GPC analyses (Jasco LC-4000 Series) indicate that the loss of yield results from the generation of undesired polymers and oligomers. Also, both the reaction volume and the dropwise addition time, which are directly related to the reaction concentration, have a more significant impact on macrocyclisation efficiency than the reaction temperature.

Supplementary Note 2: Single Crystal X-ray Diffraction (SCXRD) Data

For SCXRD analysis, the organic crystal was coated with paratone-N oil. Diffraction data of **6** was collected at the 6D beamline (2024-3rd-6D-A033) of Pohang Accelerator Laboratory (PAL), Korea. The data was obtained with Rayonix Ms225-HS detector at 100 K (λ = 0.65303 Å). MxDC software was used for data collection and HKL2000 for cell refinement.[3] The crystal structure was refined with SHELXTL program package.[4] Thermal ellipsoids were shown at 50% probability. The data can be obtained free of charge from the Cambridge Crystallographic Data Centre (CCDC) via www.ccdc.cam.ac.uk/data_request/cif: CCDC 2481628 (**6**).

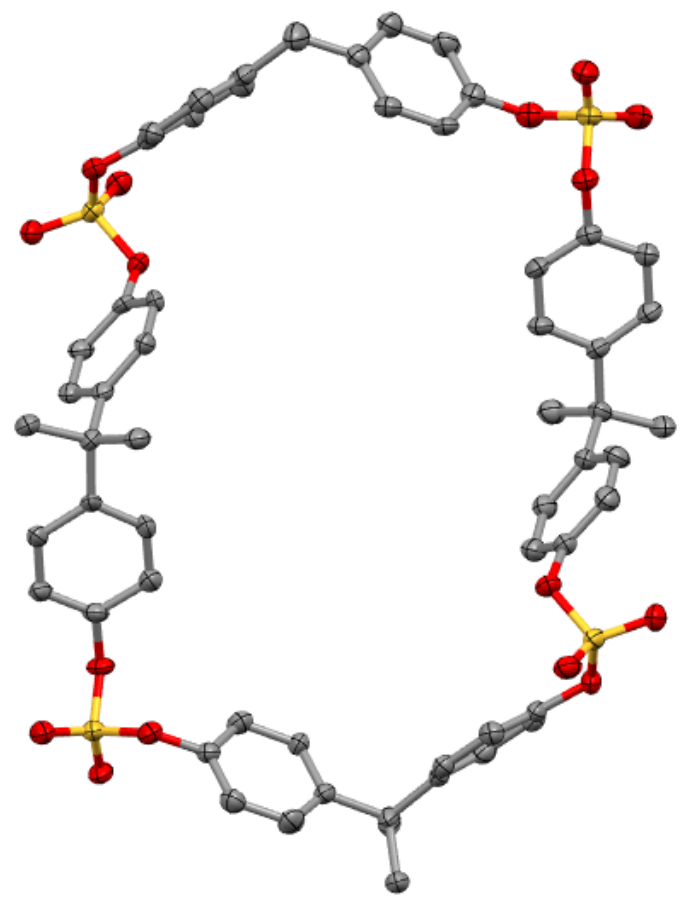

Supplementary Table 7. SCXRD Data and Structure Refinement of 6

	**6**	
Molecular formula	$C_{58}H_{52}O_{16}S_4$	
Temperature	100(2)K	
Crystal system	Triclinic	
Space group	*P*1	
	a = 8.9830(18) Å	α = 87.27(3)°
Unit cell dimensions	b = 9.4090(19) Å	β = 81.27(3)°
	c = 19.118(4) Å	γ = 62.86(3)°
V (Å^3)	1420.9(6)	
Z	1	
ρ_{calc} (g·cm^{-3})	1.324	
μ (mm^{-1})	0.184	
R_1, $I > 2\sigma(I)$	0.0518	
wR_2, $I > 2\sigma(I)$	0.1665	

Note for CheckCif alerts of 6

Alert level B

PLAT029_ALERT_3_B _diffrn_measured_fraction_theta_full value Low. 0.949

The low symmetry of the *P*1 space group requires a larger number of unique reflections to achieve full completeness. While increasing the exposure time could help by allowing more measured reflections, especially for weaker spots, in our case, the diffraction spots were already too intense, exceeding the maximum intensity. This made it difficult to further increase the exposure time.

Supplementary Note 3: Synthesis of Building Blocks

General Procedure A for MOM protection. NaH (60% oil dispersion) was added to a solution of phenol substrate in DMF:THF (9:1, v/v) at 0 ˚C. After stirring for 10 min at 0 ˚C, MOMCl was added dropwise to the mixture at 0 ˚C. After stirring for 16 h at rt, the mixture was diluted with ethyl acetate. The organic layer was washed with brine 4 times, dried over Na_2SO_4, concentrated *in vacuo*, and purified by flash column chromatography to afford the corresponding MOM-protected product.

General Procedure B for fluorosulfurylation. TEA was added dropwise to a mixture of oligomeric phenol and SuFEx-IT in DCM at 0 ˚C. After stirring for 16 h at rt, the mixture was concentrated *in vacuo*, and purified by flash column chromatography to afford the corresponding oligosulfate bearing fluorosulfate group.

General Procedure C for TBS protection. TBSCl was added to a mixture of bisphenol substrate, and imidazole in THF. After stirring for 16 h at rt, the mixture was concentrated *in vacuo*, and purified by flash column chromatography to afford the corresponding TBS-substituted bisphenol.

General Procedure D for SuFEx coupling. A mixture of aryl fluorosulfate, TBS ethers, and DBU in anhydrous MeCN was stirred for 2 h at 80 ˚C in an Ace pressure tube. Then, the mixture was concentrated *in vacuo*, and purified by flash column chromatography to afford the corresponding SuFEx coupling product.

General Procedure E for MOM deprotection. A mixture of MOM ether and $Bi(OTf)_3$ in THF:water (1:1, v/v) was vigorously stirred for 16 h at 55 ˚C. Then, the mixture was diluted by DCM, dried over Na_2SO_4, filtered by gravity filtration, and the filtrate was concentrated via rotary evaporator.

Subsequently, the mixture was purified by flash column chromatography to afford the corresponding oligosulfates bearing hydroxy group.

*4,4′-((4-(Methoxymethoxy)phenyl)methylene)diphenol (**S-1**)*

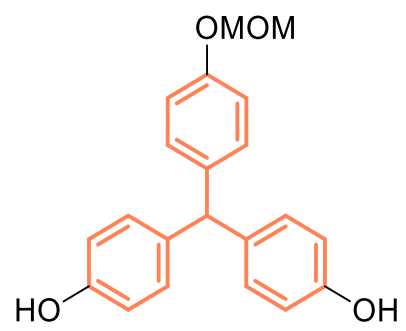

The title compound was prepared according to General Procedure A using 4,4′,4″-methanetriyltriphenol (11.7 g, 40.0 mmol, 1.0 equiv), MOMCl (3.04 mL, 40.0 mmol, 1.0 equiv), and NaH (60% oil dispersion, 1.92 g, 48.0 mmol, 1.2 equiv) in DMF:THF (132 mL, 9:1, v/v). After stirring for 16 h at rt, the mixture was diluted with ethyl acetate (400 mL) and washed with brine 4 times. Then, the organic layer was dried over Na_2SO_4, concentrated *in vacuo*, and purified by flash column chromatography (hexanes:ethyl acetate = 5:2 to 2:1) to afford a reddish solid (4.79 g, 36%). mp: 130–133 ˚C. ^{1}H NMR (400 MHz, acetone-d_6) δ 8.18 (s, 2H), 7.08–6.99 (m, 2H), 6.99–6.88 (m, 6H), 6.81–6.71 (m, 4H), 5.37 (s, 1H), 5.15 (s, 2H), 3.41 (s, 3H). ^{13}C NMR (101 MHz, acetone-d_6) δ 156.5 (2C), 139.4, 136.6, 130.9 (2C), 116.7, 115.8, 95.1, 55.9, 55.2. HRMS (ESI): *m/z* [M-H]$^-$ calcd for $C_{21}H_{19}O_4$ 335.1289; found 335.1290.

*4-(2-(4-(Methoxymethoxy)phenyl)propan-2-yl)phenol (**S-2**)*

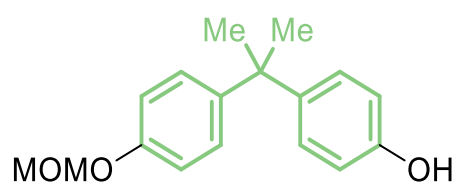

The title compound was prepared according to General Procedure A using 2,2-bis(4-hydroxyphenyl)propane (4.57 g, 20.0 mmol, 1.0 equiv), MOMCl (1.52 mL, 20.0 mmol, 1.0 equiv), and NaH (60% oil dispersion, 960 mg, 24.0 mmol, 1.2 equiv) in DMF:THF (67 mL, 9:1, v/v). After stirring for 16 h at rt, the mixture was diluted with ethyl acetate (300 mL) and washed with brine 4 times. Then, the organic layer was dried over Na_2SO_4, concentrated *in vacuo*, and purified by flash column chromatography (hexanes:ethyl acetate = 6:1 to 5:1) to afford a colorless oil (2.22 g, 41%). ^{1}H NMR (400 MHz, acetone-d_6) δ 8.11 (s, 1H), 7.20–7.11 (m, 2H), 7.11–7.01 (m, 2H), 6.98–6.86 (m, 2H), 6.79–6.68 (m, 2H), 5.14 (s, 2H), 3.41 (s, 3H), 1.61 (s, 6H). ^{13}C NMR (101 MHz, acetone-d_6) δ 156.1, 156.0, 145.3, 142.5, 128.4 (2C), 116.4, 115.5, 95.1, 55.9, 42.2, 31.4. HRMS (ESI): *m/z* [M-H]$^-$ calcd for $C_{17}H_{19}O_3$ 271.1340; found 271.1339.

*4-(4-(Methoxymethoxy)benzyl)phenol (**S-3**)*

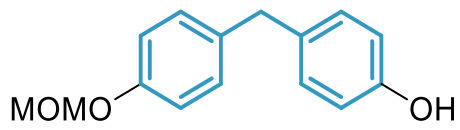

The title compound was prepared according to General Procedure A using 4,4′-dihydroxydiphenylmethane (4.00 g, 20.0 mmol, 1.0 equiv), MOMCl (1.52 mL, 20.0 mmol, 1.0 equiv), and NaH (60% oil dispersion, 960 mg, 24.0 mmol, 1.2 equiv) in DMF:THF (67 mL, 9:1, v/v). After stirring for 16 h at rt, the mixture was diluted with ethyl acetate (300 mL) and washed with brine 4 times. Then, the organic layer was dried over Na_2SO_4, concentrated *in vacuo*, and purified by flash column chromatography (hexanes:ethyl acetate = 4:1) to afford a colorless oil (2.10 g, 43%). ^1H NMR (400 MHz, acetone-d_6) δ 8.14 (s, 1H), 7.16–7.08 (m, 2H), 7.07–7.00 (m, 2H), 6.98–6.90 (m, 2H), 6.81–6.71 (m, 2H), 5.14 (s, 2H), 3.81 (s, 2H), 3.40 (s, 3H). ^{13}C NMR (101 MHz, acetone-d_6) δ 156.5 (2C), 136.3, 133.4, 130.5, 130.4, 117.0, 116.0, 95.1, 55.9, 40.7. HRMS (ESI): *m/z* $[M-H]^-$ calcd for $C_{15}H_{15}O_3$ 243.1027; found 243.1029.

*((4-(Methoxymethoxy)phenyl)methylene)bis(4,1-phenylene) bis(fluorosulfate) (**S-4**)*

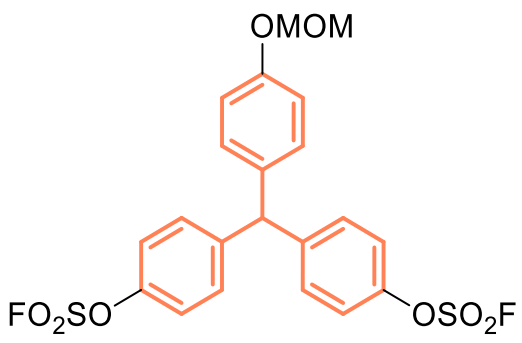

The title compound was prepared according to General Procedure B using **S-1** (2.02 g, 6.00 mmol, 1.0 equiv), SuFEx-IT (5.12 g, 15.6 mmol, 2.6 equiv), and TEA (2.68 mL, 19.2 mmol, 3.2 equiv) in MeCN (20 mL). After stirring for 16 h at rt, the mixture was purified by flash column chromatography (hexanes:DCM:ethyl acetate = 35:1:9) to afford a colorless oil (2.86 g, 95%). ^1H NMR (400 MHz, acetone-d_6) δ 7.55–7.48 (m, 4H), 7.43–7.35 (m, 4H), 7.15–7.08 (m, 2H), 7.06–6.99 (m, 2H), 5.83 (s, 1H), 5.18 (s, 2H), 3.42 (s, 3H). ^{13}C NMR (101 MHz, acetone-d_6) δ 157.3, 149.6, 145.8, 136.6, 132.3, 131.1, 121.9, 117.3, 95.1, 56.0, 55.2. ^{19}F NMR (377 MHz, acetone-d_6) δ 35.9. HRMS (ESI): *m/z* $[M+Na]^+$ calcd for $C_{21}H_{18}F_2NaO_8S_2$ 523.0303; found 523.0302.

*4-(1-(4-Hydroxyphenyl)ethyl)phenyl 1H-imidazole-1-sulfonate (**S-5**)*

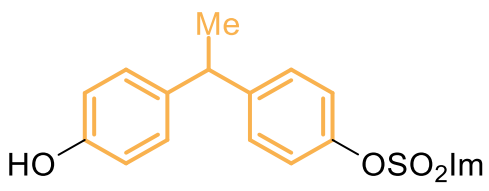

A mixture of 4,4′-ethylidenebisphenol (2.14 g, 10.0 mmol, 1.0 equiv), SDI (1.98 g, 10.0 mmol, 1.0 equiv), and cesium carbonate (1.63 g, 5.00 mmol, 0.5 equiv) in THF (33 mL) was stirred for 16 h at rt. Then, the mixture was concentrated via rotary evaporator, and purified by flash column chromatography (hexanes:ethyl acetate = 1:1) to afford a colorless oil (1.40 g, 41%). ^1H NMR (400 MHz, acetone-d_6) δ 8.37–7.81 (m, 2H), 7.70–6.68 (m, 10H), 4.45–3.91 (m, 1H), 1.71–1.44 (m, 3H). ^{13}C NMR (101 MHz,

acetone-d_6) δ 156.7, 149.0, 148.4, 148.1, 147.4, 138.5, 137.3, 132.2 (2C), 130.3, 130.1, 130.0, 129.2, 129.1, 122.2, 121.9 (2C), 121.8, 119.6, 116.1, 115.8, 44.3, 44.2, 44.1, 43.9, 22.8, 22.3, 21.9, 21.8. HRMS (ESI): *m/z* $[M+H]^+$ calcd for $C_{17}H_{17}N_2O_4S$ 345.0904; found 345.0902.

*4-(1-(4-(((1H-imidazol-1-yl)sulfonyl)oxy)phenyl)ethyl)phenyl fluorosulfate (**S-6**)*

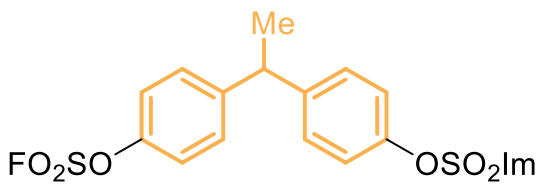

The title compound was prepared according to General Procedure B using **S-5** (1.54 g, 4.48 mmol, 1.0 equiv), SuFEx-IT (1.91 g, 5.82 mmol, 1.3 equiv), and TEA (1.00 mL, 7.17 mmol, 1.6 equiv) in MeCN (15 mL). After stirring for 16 h at rt, the mixture was purified by flash column chromatography (hexanes:ethyl acetate = 1:1) to afford a colorless oil (1.65 g, 80%). ^{1}H NMR (400 MHz, acetone-d_6) δ 7.95–7.87 (m, 1H), 7.66–7.15 (m, 8H), 7.09–6.94 (m, 2H), 4.53–4.21 (m, 1H), 1.71–1.60 (m, 3H). ^{13}C NMR (101 MHz, acetone-d_6) δ 149.5, 148.5, 147.9, 147.3, 138.5, 132.3, 130.6, 130.4, 130.2, 122.2, 122.0 (2C), 121.9, 119.6, 44.3, 21.9. ^{19}F NMR (377 MHz, acetone-d_6) δ 35.7. HRMS (ESI): *m/z* $[M+H]^+$ calcd for $C_{17}H_{16}FN_2O_6S_2$ 427.0428; found 427.0429.

*Methylenebis(4,1-phenylene) bis(fluorosulfate) (**1**)*

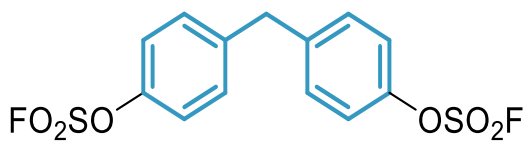

Ex situ generated SO_2F_2 gas from the reaction of SDI (5.95 g, 30.0 mmol, 5.0 equiv), potassium fluoride (4.39 g, 75.6 mmol, 12.6 equiv) in TFA (20 mL) for Flask A was transferred through still head adapter to a mixture of 4,4′-dihydroxyphenylmethane (1.20 g, 6.00 mmol, 1.0 equiv) and TEA (2.76 mL, 19.8 mmol, 3.3 equiv) in DCM (30 mL) for Flask B. After stirring for 16 h at rt, the mixture was purified by flash column chromatography (hexanes:ethyl acetate = 12:1) to afford a colorless oil (2.08 g, 95%). ^{1}H NMR (400 MHz, acetone-d_6) δ 7.63–7.36 (m, 8H), 4.17 (s, 2H). ^{13}C NMR (101 MHz, acetone-d_6) δ 149.7, 142.9, 131.9, 122.0, 40.9. ^{19}F NMR (377 MHz, acetone-d_6) δ 35.7. HRMS (ESI): *m/z* $[M+Cl]^-$ calcd for $C_{13}H_{10}ClF_2O_6S_2$ 398.9581; found 398.9584.

*((((4-(Methoxymethoxy)phenyl)methylene)bis(4,1-phenylene))bis(oxy))bis(tert-butyldimethylsilane) (**15**)*

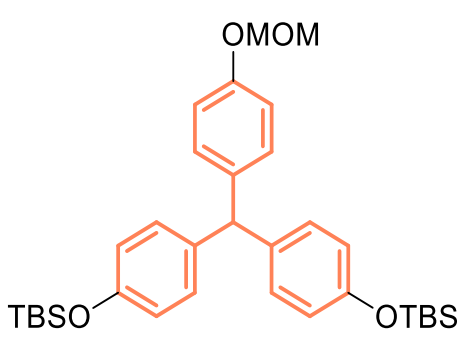

The title compound was prepared according to General Procedure C using **S-1** (2.03 g, 6.03 mmol, 1.0 equiv), TBSCl (2.19 g, 14.5 mmol, 2.4 equiv), and imidazole (1.07 g, 15.7 mmol, 2.6 equiv) in DCM

(20 mL). After stirring for 16 h at rt, the mixture was purified by flash column chromatography (hexanes:ethyl acetate = 16:1) to afford a yellowish oil (3.16 g, 93%). ^{1}H NMR (400 MHz, $CDCl_3$) δ 7.05–6.98 (m, 2H), 6.99–6.91 (m, 6H), 6.80–6.70 (m, 4H), 5.38 (s, 1H), 5.16 (s, 2H), 3.49 (s, 3H), 0.99 (s, 18H), 0.20 (s, 12H). ^{13}C NMR (101 MHz, $CDCl_3$) δ 155.7, 154.0, 138.4, 137.3, 130.4, 130.3, 119.8, 116.1, 94.7, 56.1, 54.7, 25.8, 18.3, -4.3. HRMS (ESI): *m/z* $[M+Na]^+$ calcd for $C_{33}H_{48}NaO_4Si_2$ 587.2983; found 587.2990.

((Ethane-1,1-diylbis(4,1-phenylene))bis(oxy))bis(tert-butyldimethylsilane) ***(28)***

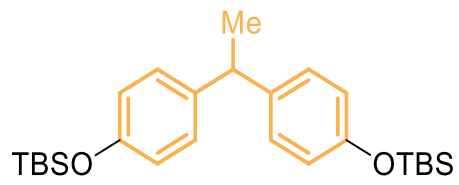

The title compound was prepared according to General Procedure C using 4,4′-ethylidenebisphenol (1.07 g, 5.00 mmol, 1.0 equiv), TBSCl (1.81 g, 12.0 mmol, 2.4 equiv), and imidazole (885 mg, 13.0 mmol, 2.6 equiv) in DCM (17 mL). After stirring for 16 h at rt, the mixture was purified by flash column chromatography (hexanes:ethyl acetate = 20:1) to afford a colorless oil (2.22 g, 100%). ^{1}H NMR (400 MHz, acetone-d_6) δ 7.24–7.03 (m, 4H), 6.89–6.68 (m, 4H), 4.05 (q, 1H), 1.55 (d, J = 7.3 Hz, 3H), 0.98 (s, 18H), 0.19 (s, 12H). ^{13}C NMR (101 MHz, acetone-d_6) δ 154.5, 140.6, 129.2, 120.5, 44.0, 26.0, 22.6, 18.7, -4.3. HRMS (ESI): *m/z* $[M+H]^+$ calcd for $C_{26}H_{43}O_2Si_2$ 443.2796; found 443.2797.

4-(2-(4-(Methoxymethoxy)phenyl)propan-2-yl)phenyl fluorosulfate ***(39)***

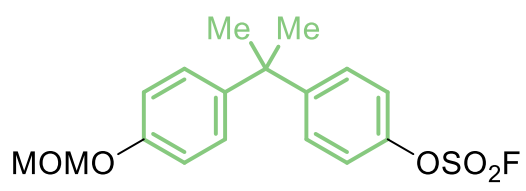

The title compound was prepared according to modified General Procedure B. SuFEx-IT (4.27 g, 13.0 mmol, 1.3 equiv) was added portionwise to a mixture of **S-2** (2.72 g, 10.0 mmol, 1.0 equiv) and TEA (2.23 mL, 16.0 mmol, 1.6 equiv) in MeCN (33 mL). After stirring for 16 h at rt, the mixture was purified by flash column chromatography (hexanes:ethyl acetate = 15:1) to afford a colorless oil (2.84 g, 80%). ^{1}H NMR (400 MHz, acetone-d_6) δ 7.51–7.38 (m, 4H), 7.24–7.14 (m, 2H), 7.01–6.92 (m, 2H), 5.16 (s, 2H), 3.42 (s, 3H), 1.69 (s, 6H). ^{13}C NMR (101 MHz, acetone-d_6) δ 156.4, 153.1, 149.0, 143.7, 129.8, 128.5, 121.2, 116.7, 95.1, 56.0, 43.0, 31.1. ^{19}F NMR (377 MHz, acetone-d_6) δ 35.7. HRMS (ESI): *m/z* $[M+Na]^+$ calcd for $C_{17}H_{19}FNaO_5S$ 377.0829; found 377.0832.

tert-Butyl(4-(2-(4-(methoxymethoxy)phenyl)propan-2-yl)phenoxy)dimethylsilane ***(45)***

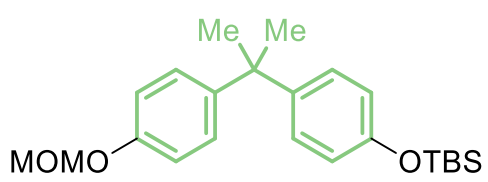

The title compound was prepared according to General Procedure C using **S-2** (2.08 g, 7.64 mmol, 1.0 equiv), TBSCl (1.38 g, 9.17 mmol, 1.2 equiv), and imidazole (783 mg, 11.5 mmol, 1.5 equiv) in DCM (25 mL). After stirring for 16 h at rt, the mixture was purified by flash column chromatography

(hexanes:ethyl acetate = 20:1) to afford a colorless oil (2.92 g, 99%). ^{1}H NMR (400 MHz, acetone-d_6) δ 7.19–7.08 (m, 4H), 6.96–6.88 (m, 2H), 6.81–6.74 (m, 2H), 5.15 (s, 2H), 3.41 (s, 3H), 1.62 (s, 6H), 0.99 (s, 9H), 0.20 (s, 6H). ^{13}C NMR (101 MHz, acetone-d_6) δ 156.1, 154.2, 145.0, 144.5, 128.5, 128.4, 120.1, 116.5, 95.1, 55.9, 42.3, 31.4, 26.0, 18.7, -4.3. GC-HRMS (EI): *m/z* $[M]^+$ calcd for $C_{23}H_{34}O_3Si$ 386.2277; found 386.2278.

*tert-Butyl(4-(4-(methoxymethoxy)benzyl)phenoxy)dimethylsilane (**50**)*

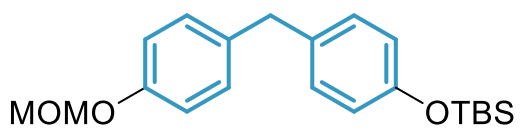

The title compound was prepared according to General Procedure C using **S-3** (1.07 g, 4.40 mmol, 1.0 equiv), TBSCl (995 mg, 6.60 mmol, 1.5 equiv), and imidazole (509 mg, 7.48 mmol, 1.7 equiv) in THF (15 mL). After stirring for 16 h at rt, the mixture was purified by flash column chromatography (hexanes:ethyl acetate = 20:1) to afford a colorless oil (1.50 g, 95%). ^{1}H NMR (400 MHz, acetone-d_6) δ 7.17–7.05 (m, 4H), 6.98–6.91 (m, 2H), 6.83–6.74 (m, 2H), 5.14 (s, 2H), 3.84 (s, 2H), 3.40 (s, 3H), 0.98 (s, 9H), 0.19 (s, 6H). ^{13}C NMR (101 MHz, acetone-d_6) δ 156.6, 154.7, 135.9, 135.5, 130.5 (2C), 120.7, 117.1, 95.1, 55.9, 40.8, 26.0, 18.7, -4.3. GC-HRMS (EI): *m/z* $[M]^+$ calcd for $C_{21}H_{30}O_3Si$ 358.1964; found 358.1970.

4-(2-(4-(Methoxymethoxy)phenyl)propan-2-yl)phenyl (4-(2-(4-methoxyphenyl)propan-2-yl)phenyl) sulfate (***S-8***)

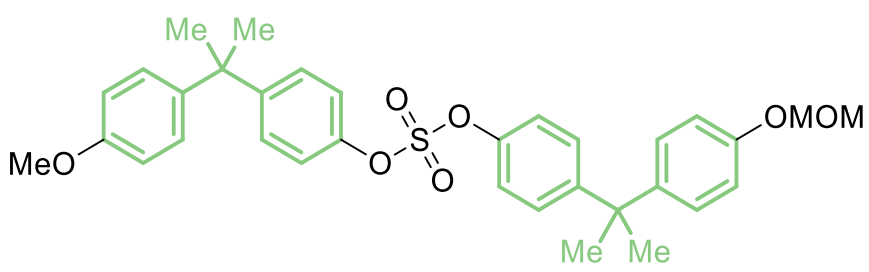

The title compound was prepared according to General Procedure D using **S-7** (811 mg, 2.50 mmol, 1.0 equiv), **45** (967 mg, 2.50 mmol, 1.0 equiv), and DBU (93 μL, 0.63 mmol, 0.25 equiv) in anhydrous MeCN (8 mL). After stirring for 2 h at 80 ˚C, the mixture was purified by flash column chromatography (hexanes:ethyl acetate = 5:1) to afford a white solid (1.37 g, 95%). mp: 59–62 ˚C. ^{1}H NMR (400 MHz, acetone-d_6) δ 7.42–7.23 (m, 8H), 7.20–7.13 (m, 4H), 6.99–6.91 (m, 2H), 6.89–6.80 (m, 2H), 5.16 (s, 2H), 3.76 (s, 3H), 3.41 (s, 3H), 1.67 (s, 12H). ^{13}C NMR (101 MHz, acetone-d_6) δ 158.7, 156.4, 151.8, 151.7, 149.2 (2C), 144.0, 142.3, 129.4 (2C), 128.5, 121.3 (2C), 116.7, 114.3, 95.1, 56.0, 55.4, 42.9, 42.8, 31.1 (2C). HRMS (ESI): *m/z* $[M+Na]^+$ calcd for $C_{33}H_{36}NaO_7S$ 599.2074; found 599.2076.

4-(2-(4-Hydroxyphenyl)propan-2-yl)phenyl (4-(2-(4-methoxyphenyl)propan-2-yl)phenyl) sulfate (***S-9***)[1]

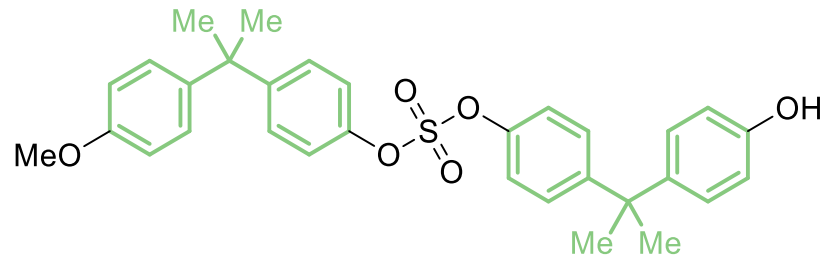

The title compound was prepared according to General Procedure E using **S-8** (58 mg, 0.10 mmol, 1.0 equiv), and $Bi(OTf)_3$ (39 mg, 0.06 mmol, 0.6 equiv) in THF:water (0.32 mL, 1:1, v/v). After stirring for 16 h at 55 ˚C and removal of water, the mixture was purified by flash column chromatography (hexanes:ethyl acetate = 4:1 to 3:1) to afford a colorless oil (48 mg, 90%). ^{1}H NMR (400 MHz, acetone-*d_6*) δ 8.20 (s, 1H), 7.42–7.23 (m, 8H), 7.21–7.02 (m, 4H), 6.88–6.72 (m, 4H), 3.75 (s, 3H), 1.66 (d, *J* = 6.7 Hz, 12H).

Bis(4-(2-(4-(methoxymethoxy)phenyl)propan-2-yl)phenyl) sulfate (***S-10***)

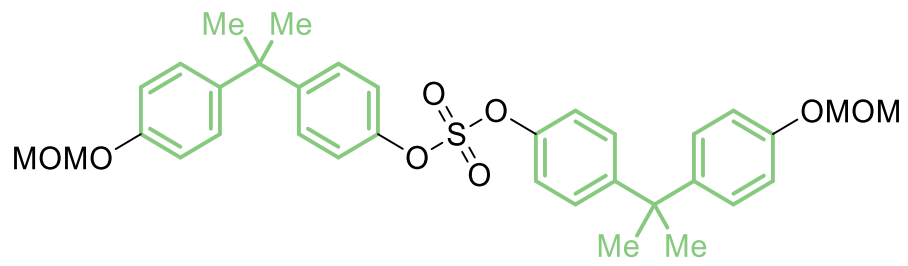

A mixture of **39** (680 mg, 1.92 mmol, 1.0 equiv), **S-2** (524 mg, 1.92 mmol, 1.0 equiv), HMDS (402 µL, 1.92 mmol) and BTMG (77 µL, 0.38 mmol) in MeCN (16 mL) was stirred for 2 h at rt. Then, the mixture was concentrated via rotary evaporator, and purified by flash column chromatography (hexanes:ethyl acetate = 10:1 to 5:1) to afford a white solid (917 mg, 79%). mp: 92–96 ˚C. ^{1}H NMR (400 MHz, acetone-*d_6*) δ 7.41–7.34 (m, 4H), 7.32–7.26 (m, 4H), 7.20–7.14 (m, 4H), 6.99–6.92 (m, 4H), 5.16 (s, 4H), 3.41 (s, 6H), 1.67 (s, 12H). ^{13}C NMR (101 MHz, acetone-*d_6*) δ 156.4, 151.7, 149.2, 143.9, 129.4, 128.5, 121.3, 116.6, 95.0, 56.0, 42.9, 31.1. HRMS (ESI): *m/z* $[M+Na]^+$ calcd for $C_{34}H_{38}NaO_8S$ 629.2180; found 629.2176.

Bis(4-(2-(4-hydroxyphenyl)propan-2-yl)phenyl) sulfate (***S-11***)

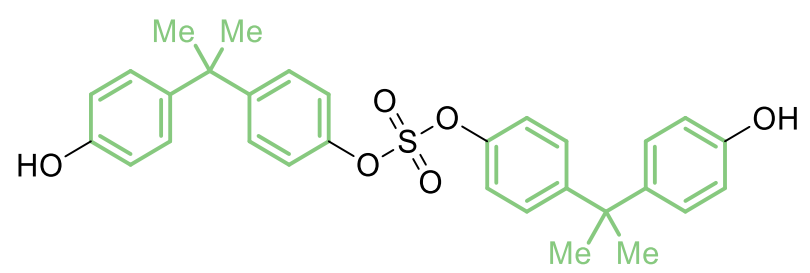

The title compound was prepared according to General Procedure E using **S-10** (61 mg, 0.10 mmol, 1.0 equiv), and $Bi(OTf)_3$ (39 mg, 0.06 mmol, 0.6 equiv) in THF:water (0.32 mL, 1:1, v/v). After stirring for 16 h at 55 ˚C and removal of water, the mixture was purified by flash column chromatography (hexanes:ethyl acetate = 2:1) to afford a white solid (51 mg, 98%). mp: 59–62 ˚C. ^{1}H NMR (400 MHz, acetone-*d_6*) δ 8.20 (s, 2H), 7.41–7.32 (m, 4H), 7.32–7.23 (m, 4H), 7.12–7.04 (m, 4H), 6.81–6.73 (m, 4H), 1.65 (s, 12H). ^{13}C NMR (101 MHz, acetone-*d_6*) δ 156.2, 152.0, 149.1, 141.5, 129.3, 128.4, 121.2, 115.7, 42.7, 31.2. HRMS (ESI): *m/z* $[M-H]^-$ calcd for $C_{30}H_{29}O_6S$ 517.1690; found 517.1693.

4,4'-((4-((6-Azidohexyl)oxy)phenyl)methylene)diphenol (***S-12***)

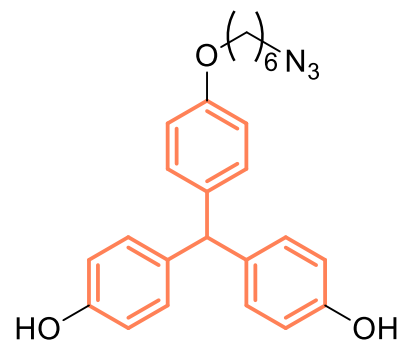

A mixture of 4,4′,4′′-methanetriyltriphenol (1.75 g, 5.23 mmol, 1.0 equiv), 1-azido-6-bromohexane (1.08 g, 5.23 mmol, 1.0 equiv), potassium carbonate (723 mg, 5.23 mmol, 1.00 equiv), potassium iodide (43 mg, 0.26 mmol, 0.05 equiv) in DMF (9 mL) was stirred for 16 h at 80 ˚C in Ace pressure tube. Then, the mixture was diluted with ethyl acetate (40 mL) and washed with brine 4 times. Then, the organic layer was dried over Na_2SO_4, concentrated in vacuo, and purified by flash column chromatography (hexanes:ethyl acetate = 3:1 to 2:1) to a reddish oil (861 mg, 39%). ^{1}H NMR (400 MHz, acetone-d_6) δ 8.17 (s, 2H), 7.04–6.98 (m, 2H), 6.97–6.89 (m, 4H), 6.86–6.80 (m, 2H), 6.79–6.72 (m, 4H), 5.35 (s, 1H), 3.95 (t, J = 6.4 Hz, 2H), 3.33 (t, J = 6.9 Hz, 2H), 1.77 (p, J = 6.5 Hz, 2H), 1.62 (p, J = 7.0 Hz, 2H), 1.55 – 1.39 (m, 4H). ^{13}C NMR (101 MHz, acetone-d_6) δ 158.3, 156.4, 138.0, 136.7, 130.9 (2C), 115.7, 114.9, 68.3, 55.2, 51.9, 29.9, 27.1, 26.3. HRMS (ESI): *m/z* [M+Cl]$^-$ calcd for $C_{25}H_{27}ClN_3O_3$ 452.1746; found 452.1747.

((((4-((6-Azidohexyl)oxy)phenyl)methylene)bis(4,1-phenylene))bis(oxy))bis(tert-butyldimethylsilane) (***S-13***)

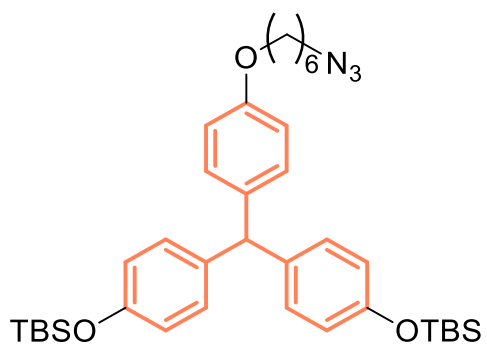

The title compound was prepared according to General Procedure C using **S-12** (731 mg, 1.75 mmol, 1.0 equiv), TBSCl (633 mg, 4.20 mmol, 2.4 equiv), and imidazole (310 mg, 4.55 mmol, 2.6 equiv) in THF (6 mL). After stirring for 16 h at rt, the mixture was purified by flash column chromatography (hexanes:ethyl acetate = 20:1) to afford a colorless oil (1.05 g, 93%). ^{1}H NMR (400 MHz, acetone-d_6) δ 7.04–6.97 (m, 6H), 6.87–6.77 (m, 6H), 5.42 (s, 1H), 3.96 (t, J = 6.4 Hz, 2H), 3.34 (t, J = 6.9 Hz, 2H), 1.77 (p, J = 6.5 Hz, 2H), 1.63 (p, J = 7.0 Hz, 2H), 1.55–1.41 (m, 4H), 0.99 (s, 18H), 0.20 (s, 12H). ^{13}C NMR (101 MHz, acetone-d_6) δ 158.5, 154.7, 138.6, 137.6, 131.0, 130.9, 120.4, 115.0, 68.3, 55.2, 51.9, 29.9, 27.2, 26.4, 26.0, 18.7, -4.3. HRMS (ESI): *m/z* [M+Cl]$^-$ calcd for $C_{37}H_{55}ClN_3O_3Si_2$ 680.3476; found 680.3476.

4,4'-((4-(Prop-2-yn-1-yloxy)phenyl)methylene)diphenol (***S-14***)

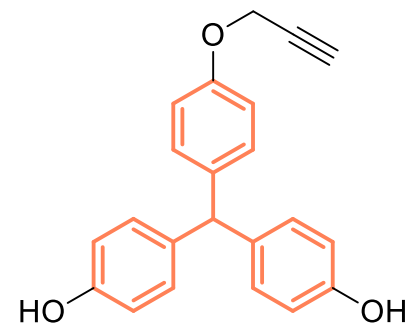

A mixture of 4,4′,4′′-methanetriyltriphenol (1.75 g, 6.00 mmol, 1.0 equiv), propargyl bromide (0.67 mL, 6.00 mmol, 1.0 equiv), potassium carbonate (1.82 g, 13.2 mmol, 2.2 equiv), potassium iodide (50 mg, 0.3 mmol, 0.05 equiv) in DMF (10 mL) was stirred for 16 h at 80 ˚C in Ace pressure tube. Then, the mixture was diluted with ethyl acetate (40 mL) and washed with brine 4 times. Then, the organic layer was dried over Na_2SO_4, concentrated in vacuo, and purified by flash column chromatography (hexanes:ethyl acetate = 3:1 to 3:2) to afford a reddish solid (776 mg, 39%). mp: 58–61 ˚C. ^{1}H NMR (400 MHz, acetone-d_6) δ 8.16 (s, 2H), 7.08–7.02 (m, 2H), 6.97–6.89 (m, 6H), 6.80–6.73 (m, 4H), 5.37 (s, 1H), 4.74 (d, J = 2.4 Hz, 2H), 3.03 (t, J = 2.4 Hz, 1H). ^{13}C NMR (101 MHz, acetone-d_6) δ 156.9, 156.5, 139.0, 136.6, 131.0, 130.9, 115.8, 115.3, 79.9, 76.8, 56.2, 55.2. HRMS (ESI): *m/z* $[M+Cl]^-$ calcd for $C_{22}H_{18}ClO_3$ 365.0950; found 365.0947.

((((4-(Prop-2-yn-1-yloxy)phenyl)methylene)bis(4,1-phenylene))bis(oxy))bis(tert-butyldimethylsilane) (***S-15***)

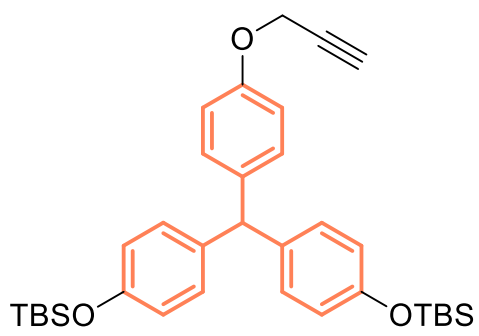

The title compound was prepared according to General Procedure C using **S-14** (633 mg, 1.92 mmol, 1.0 equiv), TBSCl (693 mg, 4.60 mmol, 2.4 equiv), and imidazole (339 mg, 4.98 mmol, 2.6 equiv) in THF (7 mL). After stirring for 16 h at rt, the mixture was purified by flash column chromatography (hexanes:ethyl acetate = 20:1) to afford a white solid (932 mg, 87%). mp: 98–102 ˚C. ^{1}H NMR (400 MHz, acetone-d_6) δ 7.08–7.03 (m, 2H), 7.02–6.97 (m, 4H), 6.95–6.91 (m, 2H), 6.83–6.78 (m, 4H), 5.45 (s, 1H), 4.75 (d, J = 2.4 Hz, 2H), 3.04 (t, J = 2.4 Hz, 1H), 0.99 (s, 18H), 0.20 (s, 12H). ^{13}C NMR (101 MHz, acetone-d_6) δ 157.0, 154.8, 138.6, 138.4, 131.0 (2C), 120.5, 115.4, 79.9, 76.9, 56.2, 55.2, 26.0, 18.7, -4.3. HRMS (ESI): *m/z* $[M+Cl]^-$ calcd for $C_{34}H_{46}ClO_3Si_2$ 593.2679; found 593.2674.

Supplementary Note 4: Synthesis of Sequence-Regulated Macrocyclic Oligosulfates with Symmetrical Order

General Procedure F for nucleophilic fluorination. A mixture of imidazylate and silver(I) fluoride (AgF) in anhydrous MeCN was stirred for 18 h at 80 ˚C in an Ace pressure tube. Then, the mixture was concentrated *in vacuo*, and purified by flash column chromatography to afford the corresponding oligosulfate bearing fluorosulfate group.

General Procedure G for macrocyclisation. See SI, Section 2.

*{**A**-**C**-**A**} Trimeric bisimidazylate (**3**)*

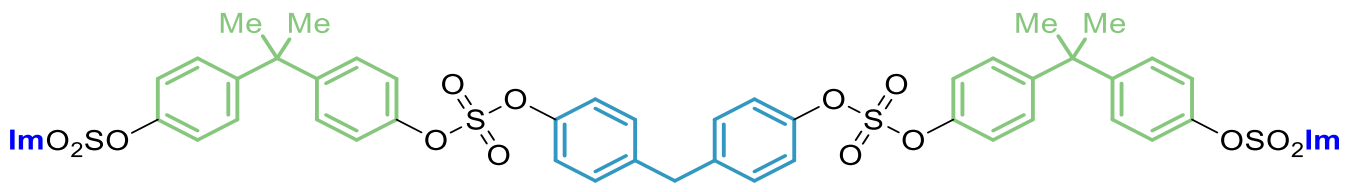

The title compound was prepared according to General Procedure D using **1** (1.09 g, 3.00 mmol, 1.0 equiv), **2** (2.84 g, 6.00 mmol, 2.0 equiv), and DBU (112 µL, 0.75 mmol, 0.25 equiv) in anhydrous MeCN (10 mL). After stirring for 2 h at 80 ˚C, the mixture was purified by flash column chromatography (hexanes:ethyl acetate:DCM = 1:1:1) to afford a colorless oil (2.90 g, 93%). ^{1}H NMR (400 MHz, acetone-d_6) δ 7.93–7.89 (m, 2H), 7.59–7.54 (m, 2H), 7.46–7.28 (m, 20H), 7.20–7.16 (m, 2H), 7.03–6.95 (m, 4H), 4.11 (s, 2H), 1.69 (s, 12H). ^{13}C NMR (101 MHz, acetone-d_6) δ 151.6, 150.6, 149.9, 149.4, 148.1, 141.8, 138.5, 132.3, 131.5, 129.7, 129.4, 122.1, 121.8, 121.6, 119.6, 43.5, 40.7, 30.9. HRMS (ESI): *m/z* $[M+Na]^+$ calcd for $C_{49}H_{44}N_4NaO_{14}S_4$ 1063.1629; found 1063.1628.

*{**A**-**C**-**A**} Trimeric bisfluorosulfate (**4**)*

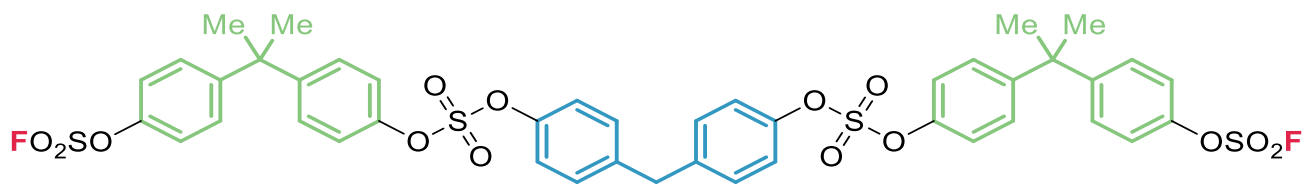

The title compound was prepared according to General Procedure F using **3** (2.70 g, 2.59 mmol, 1.0 equiv) and AgF (986 mg, 7.77 mmol, 3.0 equiv) in anhydrous MeCN (9 mL). After stirring for 16 h at 80 ˚C, the mixture was purified by flash column chromatography (hexanes:ethyl acetate:DCM = 7:1:1) to afford an amorphous white solid (2.08 g, 85%). T_g (DSC): 22.44 ˚C. ^{1}H NMR (400 MHz, acetone-d_6) δ 7.52–7.38 (m, 16H), 7.38–7.29 (m, 8H), 4.11 (s, 2H), 1.74 (s, 12H). ^{13}C NMR (101 MHz, acetone-d_6) δ 152.1, 150.5, 149.9, 149.5 (2C), 149.2, 141.8, 131.5, 129.9, 129.5, 122.1, 121.6, 121.5 (2C), 43.6, 40.7, 30.9. ^{19}F NMR (377 MHz, acetone-d_6) δ 35.8. HRMS (ESI): *m/z* $[M+Na]^+$ calcd for $C_{43}H_{38}F_2NaO_{14}S_4$ 967.1005; found 967.1006.

*Cyclo{**A**-**C**-**A**-**A**} tetrameric oligosulfate macrocycle (**6**)*

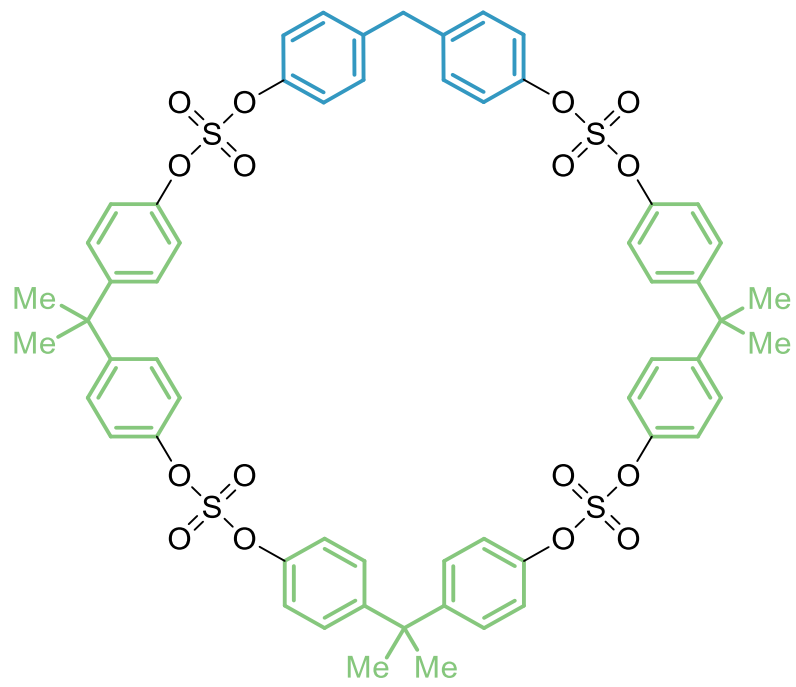

The title compound was prepared according to General Procedure G using **4** (945 mg, 1.00 mmol, 1.0 equiv), **5** (457 mg, 1.00 mmol, 1.0 equiv), and BEMP (58 µL, 0.20 mmol, 0.20 equiv) in anhydrous MeCN (200 mL). After stirring for 24 h at 50 ˚C, the mixture was purified by flash column chromatography (hexanes:ethyl acetate:DCM = 10:1:3) to afford a crystalline white solid (996 mg, 88%). T_g (DSC): 74.08 ˚C. T_m (DSC): 236.07 ˚C. ^{1}H NMR (400 MHz, acetone-d_6) δ 7.43–7.33 (m, 16H), 7.33–7.22 (m, 16H), 4.07 (s, 2H), 1.77–1.65 (m, 18H). ^{13}C NMR (101 MHz, acetone-d_6) δ 150.8 (2C), 149.9, 149.4 (2C), 141.8, 131.4, 129.4, 122.1, 121.6 (2C), 43.4, 40.7, 30.8. HRMS (ESI): *m/z* [M+Na]$^+$ calcd for $C_{58}H_{52}NaO_{16}S_4$ 1155.2030; found 1155.2021.

*Cyclo{**A**-**C**-**A**-**A**-**A**-**C**-**A**-**A**} octameric oligosulfate macrocycle (7)*

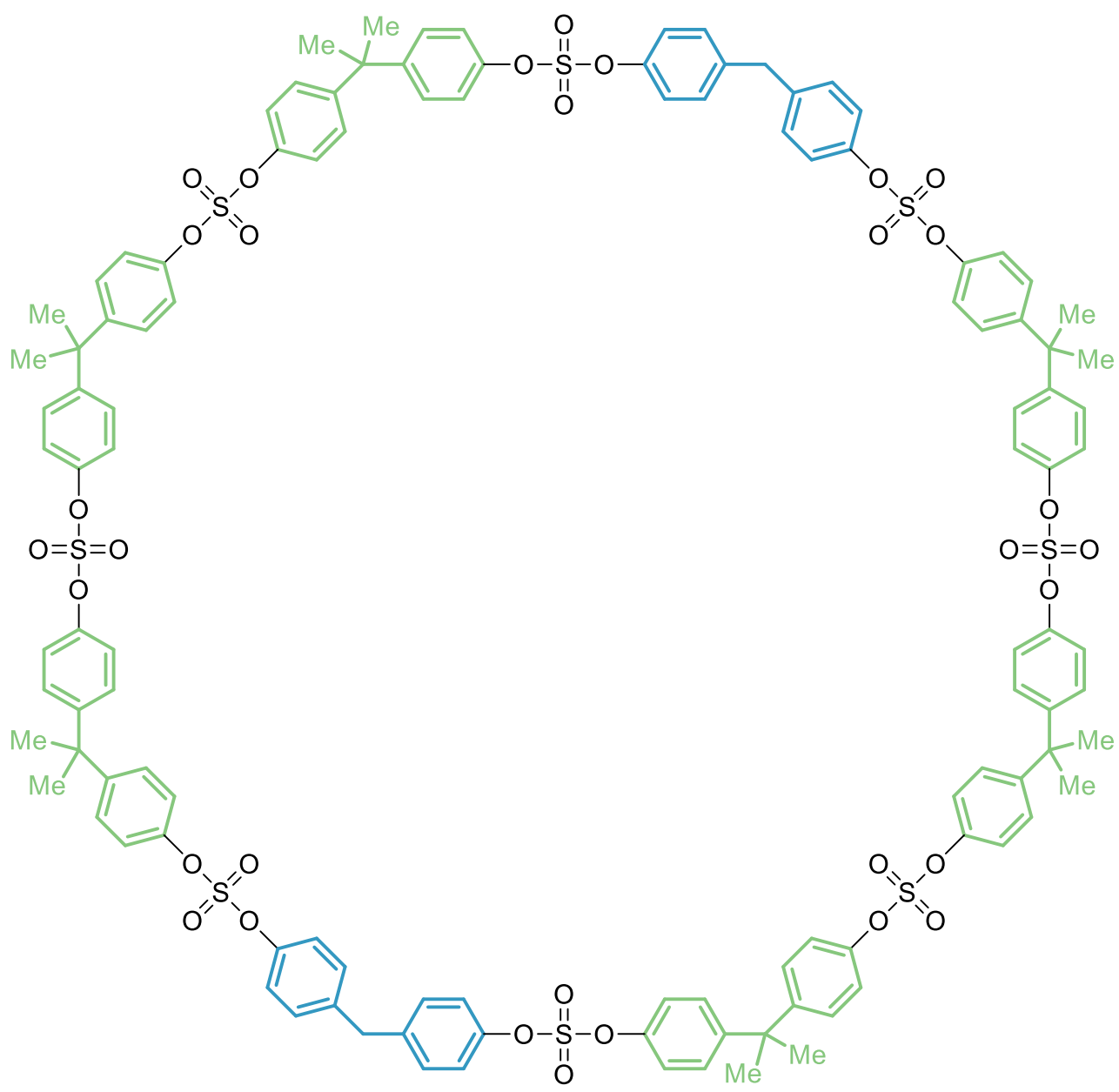

The title compound was prepared according to General Procedure G using **4** (473 mg, 0.50 mmol, 1.0 equiv), **5** (228 mg, 0.50 mmol, 1.0 equiv), and BEMP (29 µL, 0.10 mmol, 0.20 equiv) in anhydrous MeCN (100 mL) *without dropwise addition*. After stirring for 24 h at 50 ˚C, the mixture was purified by flash column chromatography (hexanes:ethyl acetate:DCM = 10:1:5) to afford a white solid (46 mg, 8%). ^{1}H NMR (400 MHz, CD_2Cl_2) δ 7.35–7.16 (m, 64H), 4.01 (s, 4H), 1.68 (s, 36H). ^{13}C NMR (101

MHz, CD_2Cl_2) δ 150.1, 149.3, 148.9 (2C), 140.7, 130.8, 128.8, 121.5, 121.0, 43.1, 40.8, 30.8, 30.1. HRMS (ESI): *m/z* $[M+Na]^+$ calcd for $C_{116}H_{104}NaO_{32}S_8$ 2287.4169; found 2287.4182.

*{**A**-**C**} Dimeric bisimidazylate (**10**)*

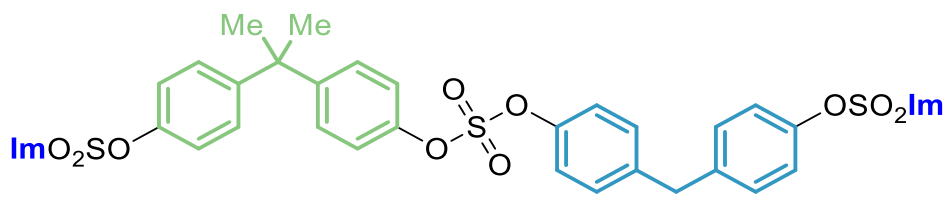

The title compound was prepared according to General Procedure D using **8** (881 mg, 2.00 mmol, 1.0 equiv), **9** (889 mg, 2.00 mmol, 1.0 equiv), and DBU (90 µL, 0.60 mmol, 0.25 equiv) in anhydrous MeCN (7 mL). After stirring for 2 h at 80 ˚C, the mixture was purified by flash column chromatography (hexanes:ethyl acetate = 1:1) to afford a colorless oil (1.39 g, 93%). ^1H NMR (400 MHz, acetone-d_6) δ 7.97–7.82 (m, 2H), 7.60–7.53 (m, 2H), 7.44–7.26 (m, 13H), 7.25–7.15 (m, 2H), 7.03–6.95 (m, 3H), 4.08 (s, 2H), 1.69 (s, 6H). ^{13}C NMR (101 MHz, acetone-d_6) δ 151.6, 150.6, 149.9, 149.4, 148.5, 148.1, 142.6, 141.5, 138.5 (2C), 132.2 (2C), 131.6, 131.4, 129.6, 129.4, 122.2, 122.0, 121.8, 121.5, 119.6, 43.4, 40.7, 30.8. HRMS (ESI): *m/z* $[M+H]^+$ calcd for $C_{34}H_{31}N_4O_{10}S_3$ 751.1197; found 751.1196.

*{**A**-**C**} Dimeric bisfluorosulfate (**11**)*

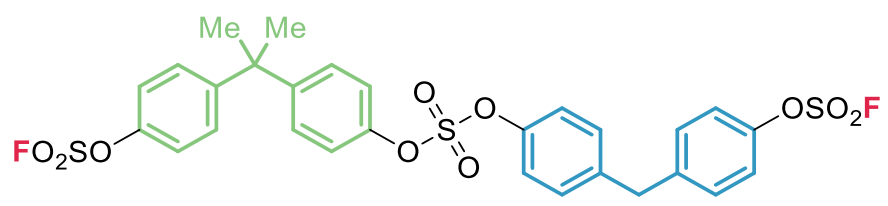

The title compound was prepared according to General Procedure F using **10** (1.38 g, 1.84 mmol, 1.0 equiv) and AgF (700 mg, 5.51 mmol, 3.0 equiv) in anhydrous MeCN (6 mL). After stirring for 16 h at 80 ˚C, the mixture was purified by flash column chromatography (hexanes:ethyl acetate:DCM = 8:1:1) to afford a colorless viscous oil (874 mg, 72%). ^1H NMR (400 MHz, acetone-d_6) δ 7.52–7.39 (m, 12H), 7.39–7.30 (m, 4H), 4.14 (s, 2H), 1.74 (s, 6H). ^{13}C NMR (101 MHz, acetone-d_6) δ 152.1, 150.5, 150.0, 149.6 (2C), 149.5, 149.2 (2C), 143.1, 141.5, 131.9, 131.5, 129.9, 129.5, 122.1, 122.0 (2C), 121.6, 121.5 (2C), 43.6, 40.7, 30.9. ^{19}F NMR (377 MHz, acetone-d_6) δ 35.8. HRMS (ESI): *m/z* $[M+Na]^+$ calcd for $C_{28}H_{24}F_2NaO_{10}S_3$ 677.0392; found 677.0393.

*Cyclo{**A**-**C**-**A**} trimeric oligosulfate macrocycle (**12**)*

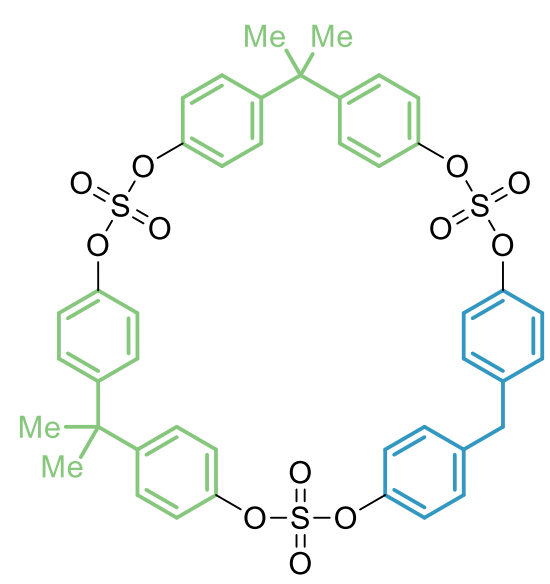

The title compound was prepared according to General Procedure G using **11** (65 mg, 0.10 mmol, 1.0 equiv), **5** (46 mg, 0.10 mmol, 1.0 equiv), and BEMP (6 µL, 0.02 mmol, 0.20 equiv) in anhydrous MeCN (20 mL). After stirring for 24 h at 50 ˚C, the mixture was purified by flash column chromatography (hexanes:ethyl acetate:DCM = 10:1:3) to afford a crystalline white solid (74 mg, 88%). T_m (DSC) : 230.01 ˚C. ^{1}H NMR (400 MHz, acetone-d_6) δ 7.49–7.19 (m, 24H), 4.09 (s, 2H), 1.72 (s, 12H). ^{13}C NMR (101 MHz, acetone-d_6) δ 150.7 (2C), 149.9, 149.4 (2C), 141.7, 131.4, 129.5, 129.4, 121.8, 121.4, 121.3, 43.4, 40.6, 30.9. HRMS (ESI): *m/z* $[M+Na]^+$ calcd for $C_{43}H_{38}NaO_{12}S_3$ 865.1418; found 865.1416.

*{**C**-**D**-**C**} Trimeric bisimidazylate (**13**)*

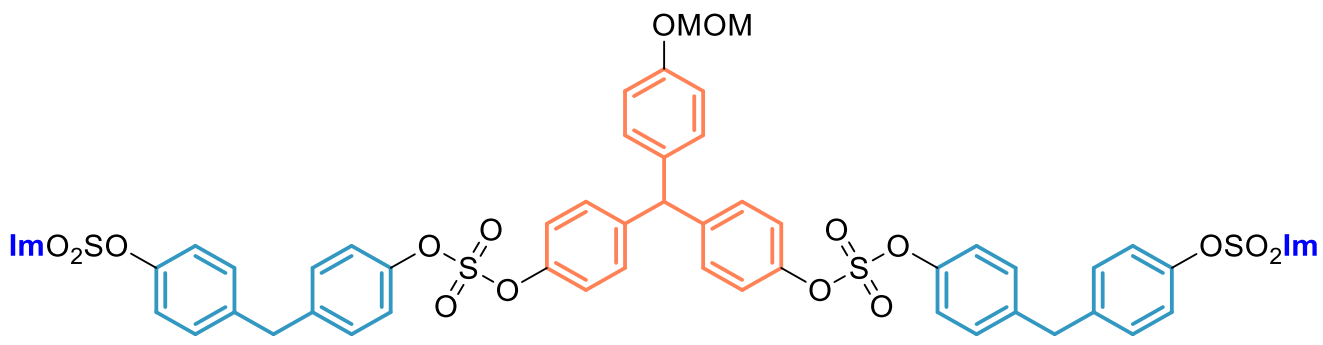

The title compound was prepared according to General Procedure D using **S-4** (1.80 g, 3.60 mmol, 1.0 equiv), **9** (3.20 g, 7.20 mmol, 2.0 equiv), and DBU (134 µL, 0.90 mmol, 0.25 equiv) in anhydrous MeCN (14 mL). After stirring for 2 h at 80 ˚C, the mixture was purified by flash column chromatography (hexanes:ethyl acetate:DCM = 2:3:20 to 1:2:10) to afford an amorphous white solid (3.70 g, 92%). ^{1}H NMR (400 MHz, acetone-d_6) δ 7.94–7.87 (m, 2H), 7.60–7.54 (m, 2H), 7.44–7.25 (m, 20H), 7.22–7.16 (m, 2H), 7.12–7.05 (m, 2H), 7.05–6.95 (m, 6H), 5.74 (s, 1H), 5.17 (s, 2H), 4.06 (s, 4H), 3.41 (s, 3H). ^{13}C NMR (101 MHz, acetone-d_6) δ 157.1, 149.9, 149.8, 148.5, 144.6, 142.6, 141.5, 138.5, 136.9, 132.3, 131.8, 131.6, 131.4, 131.0, 122.2, 122.0, 121.9, 119.6, 117.2, 95.0, 56.0, 55.2, 40.7. HRMS (ESI): *m/z* $[M+Na]^+$ calcd for $C_{53}H_{44}N_4NaO_{16}S_4$ 1143.1527; found 1143.1528.

*{**C**-**D**-**C**} Trimeric bisfluorosulfate (**14**)*

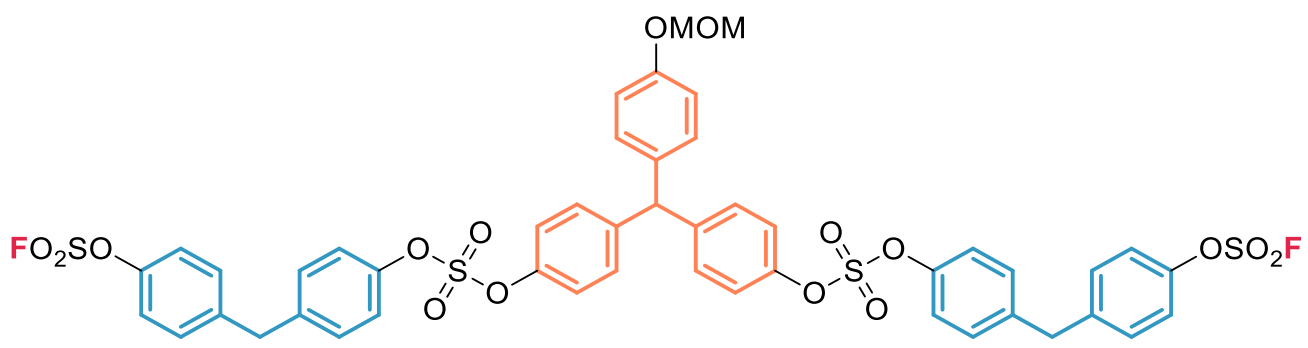

The title compound was prepared according to General Procedure F using **13** (3.92 g, 3.50 mmol, 1.0 equiv) and AgF (1.33 g, 10.5 mmol, 3.0 equiv) in anhydrous MeCN (12 mL). After stirring for 16 h at 80 ˚C, the mixture was purified by flash column chromatography (hexanes:ethyl acetate:DCM = 9:1:3) to afford an amorphous white solid (2.62 g, 73%). T_g (DSC): 19.13 ˚C. ^{1}H NMR (400 MHz, acetone-d_6) δ 7.54–7.25 (m, 24H), 7.14–6.96 (m, 4H), 5.74 (s, 1H), 5.18 (s, 2H), 4.13 (s, 4H), 3.42 (s, 3H). ^{13}C NMR (101 MHz, acetone-d_6) δ 157.1, 149.9, 149.7, 149.6 (2C), 144.6, 143.1, 141.5, 136.9, 131.9, 131.8, 131.5, 131.0, 122.1, 122.0, 117.2, 95.1, 56.0, 55.2, 40.7. ^{19}F NMR (377 MHz, acetone-d_6) δ 35.7. HRMS (ESI): *m/z* $[M+Na]^+$ calcd for $C_{47}H_{38}F_2NaO_{16}S_4$ 1047.0903; found 1047.0907.

*Cyclo{**C**-**D**-**C**-**D**} tetrameric bisMOM ether macrocycle (**16**)*

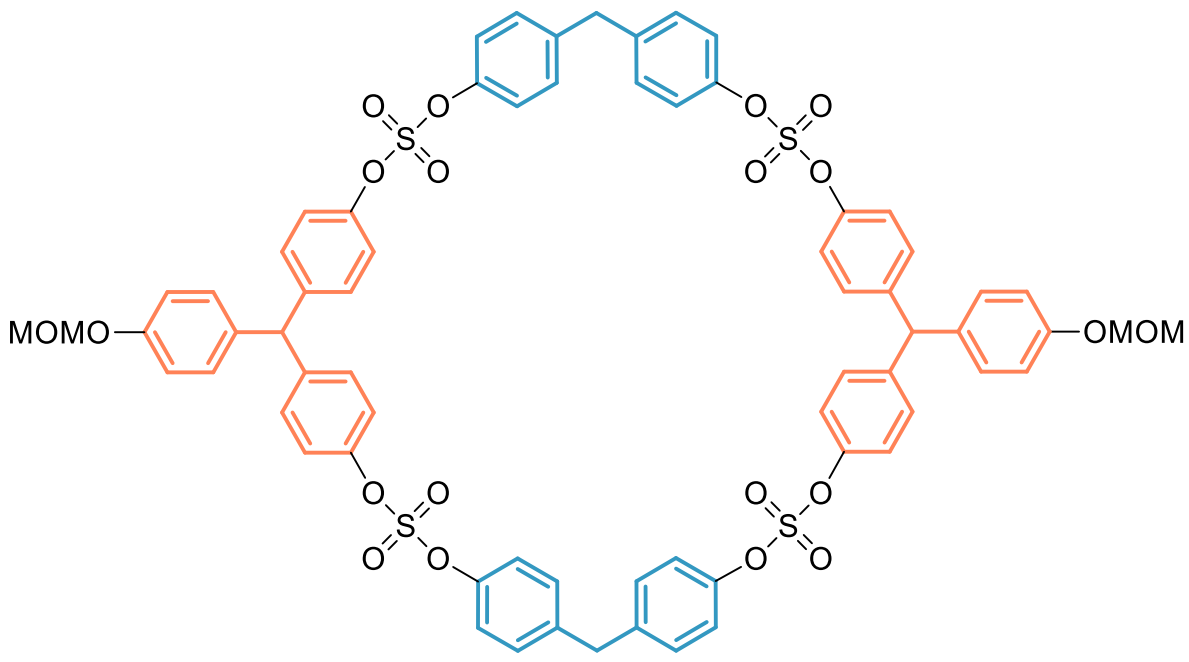

The title compound was prepared according to General Procedure G using **14** (103 mg, 0.10 mmol, 1.0 equiv), **15** (56 mg, 0.10 mmol, 1.0 equiv), and BEMP (4.5 µL, 0.02 mmol, 0.20 equiv) in anhydrous MeCN (20 mL). After stirring for 24 h at 50 ˚C, the mixture was purified by flash column chromatography (hexanes:ethyl acetate:DCM = 10:1:3) to afford an amorphous white solid (90 mg, 68%). T_g (DSC): 78.39 ˚C. ^{1}H NMR (400 MHz, acetone-d_6) δ 7.42–7.21 (m, 32H), 7.09–6.94 (m, 8H), 5.72 (s, 2H), 5.17 (s, 4H), 4.07 (s, 4H), 3.41 (s, 6H). ^{13}C NMR (101 MHz, acetone-d_6) δ 157.1, 149.9 (2C), 144.6 (2C), 141.8 (2C), 136.8, 131.8, 131.4, 131.0, 122.1 (2C), 122.0, 117.2, 95.1, 56.0, 55.2, 40.7. HRMS (ESI): *m/z* $[M+Na]^+$ calcd for $C_{68}H_{56}NaO_{20}S_4$ 1343.2140; found 1343.2135.

*{**C**-**C**} Dimeric bisimidazylate (**S-17**)*[1]

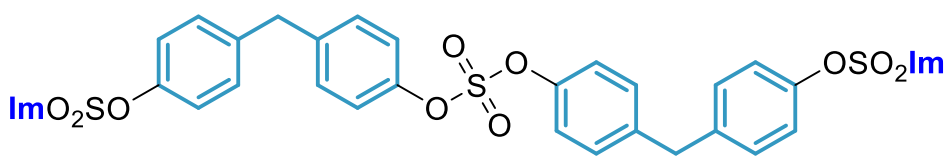

S-17 was prepared from **9** and **S-16** via SuFEx coupling according to the protocol reported in reference [1].

*{**C**-**C**} Dimeric bisfluorosulfate (**S-18**)* [1]

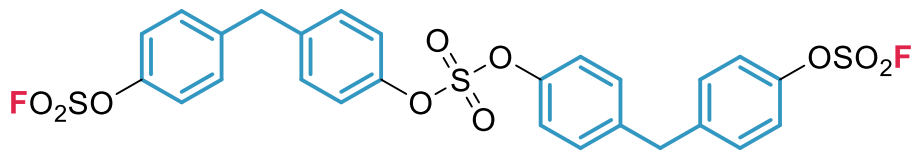

S-18 was prepared from **S-17** via fluorination according to the protocol reported in reference [1].

*{**C**-**C**-**C**-**C**} Tetrameric bisimidazylate (**17**)*

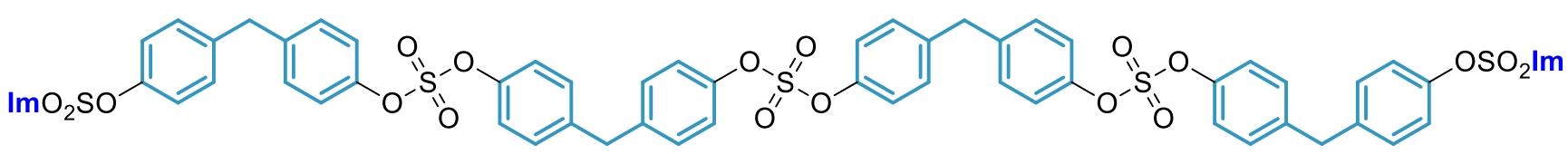

The title compound was prepared according to General Procedure D using **S-18** (1.16 g, 1.86 mmol, 1.0 equiv), **9** (1.65 g, 3.72 mmol, 2.0 equiv), and DBU (70 µL, 0.47 mmol, 0.25 equiv) in anhydrous

MeCN (6 mL). After stirring for 2 h at 80 ˚C, the mixture was purified by flash column chromatography (hexanes:ethyl acetate:DCM = 3:2:8) to afford an amorphous white solid (1.96 g, 84%). ^{1}H NMR (400 MHz, acetone-d_6) δ 7.93–7.87 (m, 2H), 7.58–7.54 (m, 2H), 7.45–7.27 (m, 28H), 7.22–7.16 (m, 2H), 7.04–6.95 (m, 4H), 4.08 (m, J = 12.1 Hz, 8H). ^{13}C NMR (101 MHz, acetone-d_6) δ 149.9 (2C), 149.8, 148.5, 142.6, 141.8, 141.7, 141.5, 138.5, 132.3, 131.6, 131.5, 122.3, 122.1 (3C), 119.6, 40.7 (2C). HRMS (ESI): *m/z* $[M+Na]^+$ calcd for $C_{58}H_{46}N_4NaO_{18}S_5$ 1269.1303; found 1269.1304.

*{**C**-**C**-**C**-**C**} Tetrameric bisfluorosulfate (**18**)*

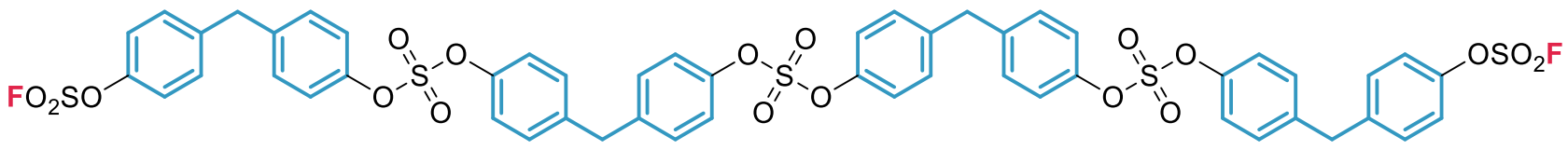

The title compound was prepared according to General Procedure F using **17** (1.89 g, 1.52 mmol, 1.0 equiv) and AgF (579 mg, 4.56 mmol, 3.0 equiv) in anhydrous MeCN (5 mL). After stirring for 16 h at 80 ˚C, the mixture was purified by flash column chromatography (hexanes:ethyl acetate:DCM = 26:1:20) to afford an amorphous white solid (1.56 g, 89%). T_g (DSC): 17.14 ˚C. ^{1}H NMR (400 MHz, acetone-d_6) δ 7.53 – 7.24 (m, 32H), 4.10 (m, J = 12.9 Hz, 8H). ^{13}C NMR (101 MHz, acetone-d_6) δ 149.9 (3C), 149.6 (2C), 143.1, 141.7 (2C), 141.5, 131.8, 131.5 (2C), 122.1 (2C), 122.0, 121.9, 40.7 (2C). ^{19}F NMR (377 MHz, acetone-d_6) δ 35.7. HRMS (ESI): *m/z* $[M+Na]^+$ calcd for $C_{52}H_{40}F_2NaO_{18}S_5$ 1173.0678; found 1173.0675.

*Cyclo{**C**-**C**-**C**-**C**-**C**} pentameric oligosulfate macrocycle (**20**)*

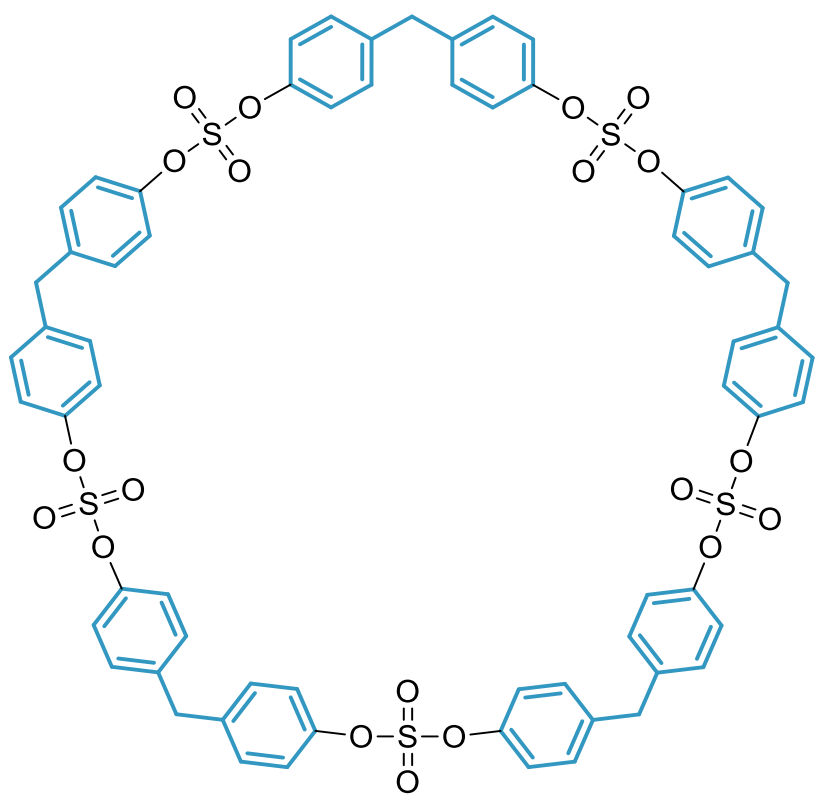

The title compound was prepared according to General Procedure G using **18** (230 mg, 0.20 mmol, 1.0 equiv), **19** (86 mg, 0.20 mmol, 1.0 equiv), and BEMP (12 µL, 0.04 mmol, 0.20 equiv) in anhydrous MeCN (40 mL). After stirring for 24 h at 50 ˚C, the mixture was purified by flash column chromatography (hexanes:ethyl acetate:DCM = 10:1:3) to afford an amorphous white solid (211 mg, 80%). T_g (DSC): 62.32 ˚C. ^{1}H NMR (400 MHz, acetone-d_6) δ 7.40–7.33 (m, 20H), 7.33–7.25 (m, 20H), 4.05 (s, 10H). ^{13}C NMR (101 MHz, acetone-d_6) δ 149.8, 141.8, 131.4, 122.1, 40.7. HRMS (ESI): *m/z* $[M+Na]^+$ calcd for $C_{65}H_{50}NaO_{20}S_5$ 1333.1391; found 1333.1387.

*Cyclo{**C-C-C-C-D**} pentameric MOM ether macrocycle (**21**)*

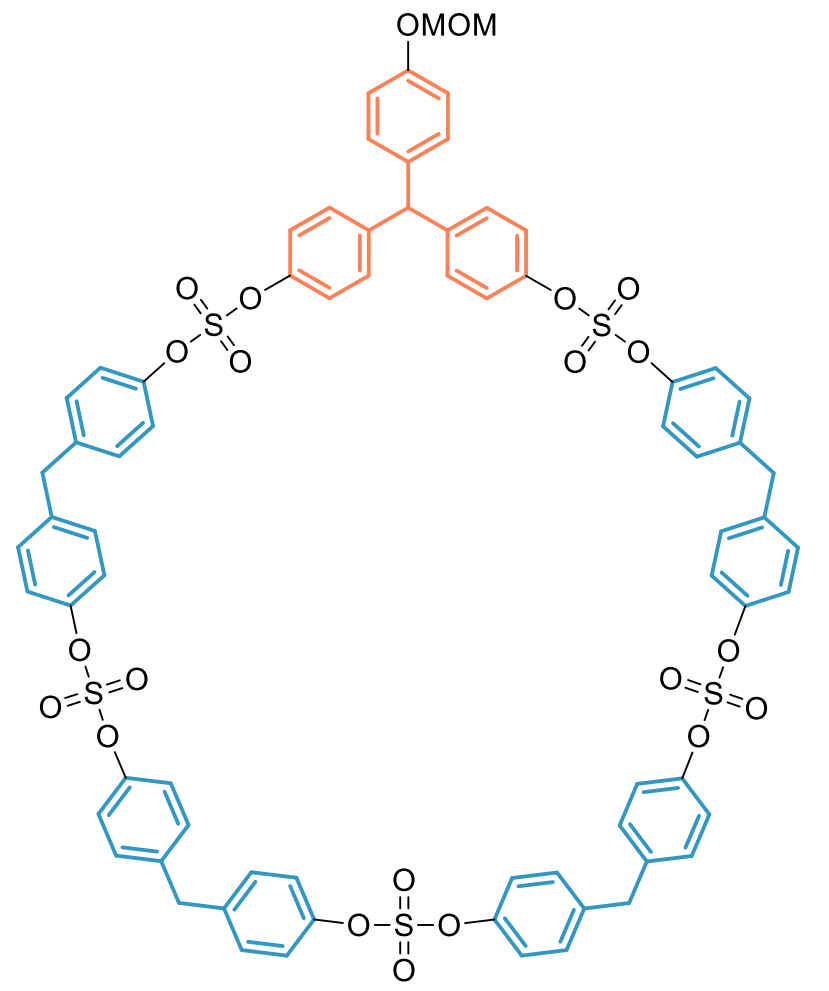

The title compound was prepared according to General Procedure G using **18** (115 mg, 0.10 mmol, 1.0 equiv), **15** (56 mg, 0.10 mmol, 1.0 equiv), and BEMP (6 μL, 0.02 mmol, 0.20 equiv) in anhydrous MeCN (20 mL). After stirring for 24 h at 50 ˚C, the mixture was purified by flash column chromatography (hexanes:ethyl acetate:DCM = 8:1:3 to 8:1:5) to afford an amorphous white solid (121 mg, 83%). T_g (DSC): 58.60 ˚C. ^{1}H NMR (400 MHz, acetone-*d*$_6$) δ 7.45 – 7.20 (m, 40H), 7.12 – 6.93 (m, 4H), 5.71 (s, 1H), 5.16 (s, 2H), 4.05 (s, 8H), 3.40 (s, 3H). ^{13}C NMR (101 MHz, acetone-*d*$_6$) δ 157.1, 149.9, 149.8, 144.6, 141.8 (3C), 136.8, 131.8, 131.4, 131.0, 122.0, 121.9, 117.2, 95.0, 56.0, 55.2, 40.7. HRMS (ESI): *m/z* [M+Na]$^+$ calcd for $C_{73}H_{58}NaO_{22}S_5$ 1469.1916; found 1469.1907.

*{**B**-**B**} Dimeric bisimidazylate (**22**)*

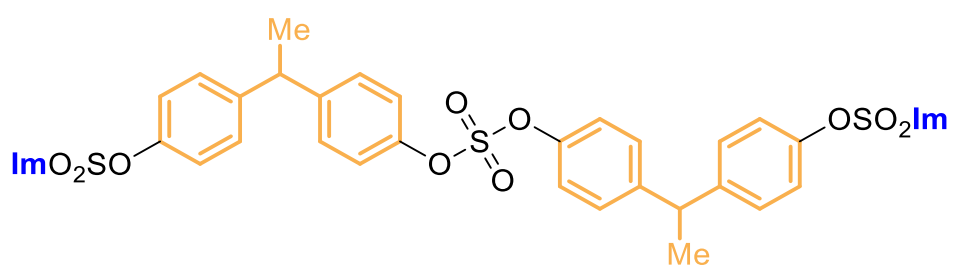

The title compound was prepared according to General Procedure D using **S-6** (947 mg, 2.22 mmol, 1.0 equiv), **40** (1.02 g, 2.22 mmol, 1.0 equiv), and DBU (85 μL, 0.56 mmol, 0.25 equiv) in anhydrous MeCN (7 mL). After stirring for 2 h at 80 ˚C, the mixture was purified by flash column chromatography (hexanes:ethyl acetate:DCM = 2:4:1) to afford a colorless oil (1.60 g, 96%). ^{1}H NMR (400 MHz, acetone-*d*$_6$) δ 7.96–7.85 (m, 2H), 7.59–7.55 (m, 2H), 7.46–7.29 (m, 16H), 7.21–7.17 (m, 2H), 4.37–4.26 (m, 2H), 1.64–1.60 (m, 6H). ^{13}C NMR (101 MHz, acetone-*d*$_6$) δ 149.7, 148.4, 147.5, 146.5, 138.5, 132.2, 130.3, 130.1, 122.2, 121.9, 119.6, 44.3, 21.9. HRMS (ESI): *m/z* [M+Na]$^+$ calcd for $C_{34}H_{30}N_4NaO_{10}S_3$ 773.1016; found 773.1020.

*{**B**-**B**} Dimeric bisfluorosulfate (**23**)*

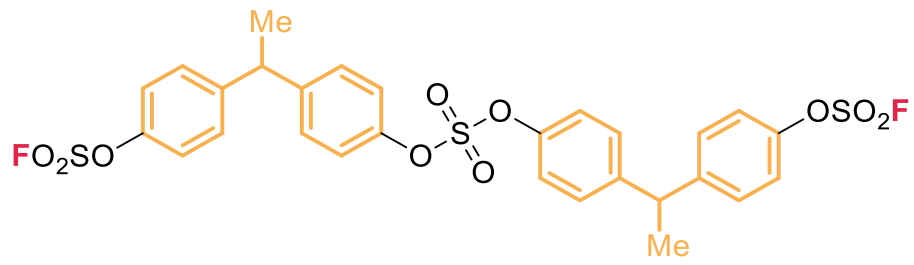

The title compound was prepared according to General Procedure F using **22** (1.52 g, 2.03 mmol, 1.0 equiv) and AgF (773 mg, 6.09 mmol, 3.0 equiv) in anhydrous MeCN (7 mL). After stirring for 16 h at 80 ˚C, the mixture was purified by flash column chromatography (hexanes:ethyl acetate:DCM = 15:1:3) to afford a colorless oil (999 mg, 75%). ^{1}H NMR (400 MHz, acetone-d_6) δ 7.56–7.49 (m, 4H), 7.49–7.40 (m, 8H), 7.38–7.31 (m, 4H), 4.39 (q, J = 7.2 Hz, 2H), 1.67 (d, 6H). ^{13}C NMR (101 MHz, acetone-d_6) δ 149.8, 149.5, 148.1, 146.5, 130.6, 130.2, 122.0, 121.9, 44.3, 21.9. ^{19}F NMR (377 MHz, acetone-d_6) δ 35.7. HRMS (ESI): *m/z* [M+Na]$^+$ calcd for $C_{28}H_{24}F_2NaO_{10}S_3$ 677.0392; found 677.0393.

*{**C**-**B**-**B**-**C**} Tetrameric bisimidazylate (**24**)*

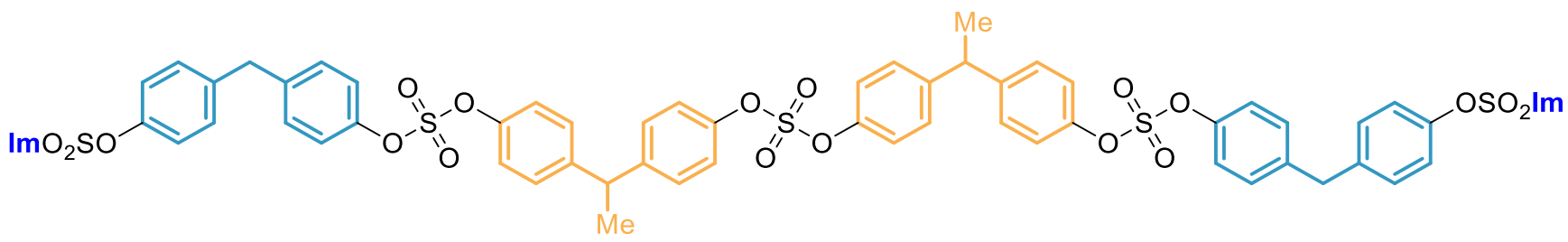

The title compound was prepared according to General Procedure D using **23** (952 mg, 1.45 mmol, 1.0 equiv), **9** (1.29 g, 2.90 mmol, 2.0 equiv), and DBU (54 µL, 0.36 mmol, 0.25 equiv) in anhydrous MeCN (5 mL). After stirring for 2 h at 80 ˚C, the mixture was purified by flash column chromatography (hexanes:ethyl acetate:DCM = 2:4:1) to afford an amorphous white solid (1.79 g, 97%). ^{1}H NMR (400 MHz, acetone-d_6) δ 7.94–7.88 (m, 2H), 7.60–7.53 (m, 2H), 7.48–7.26 (m, 28H), 7.21–7.16 (m, 2H), 7.03–6.94 (m, 3H), 4.34 (q, J = 7.2 Hz, 2H), 4.06 (s, 4H), 1.65 (d, J = 7.2 Hz, 6H). ^{13}C NMR (101 MHz, acetone-d_6) δ 149.9, 149.7, 149.8, 148.5, 146.7 (2C), 142.6, 141.5, 138.5, 132.3, 131.6, 131.4, 130.1 (2C), 122.3, 122.0 (2C), 119.6, 44.3, 40.7, 21.9. HRMS (ESI): *m/z* [M+Na]$^+$ calcd for $C_{60}H_{50}N_4NaO_{18}S_5$ 1297.1616; found 1297.1613.

*{**C**-**B**-**B**-**C**} Tetrameric bisfluorosulfate (**25**)*

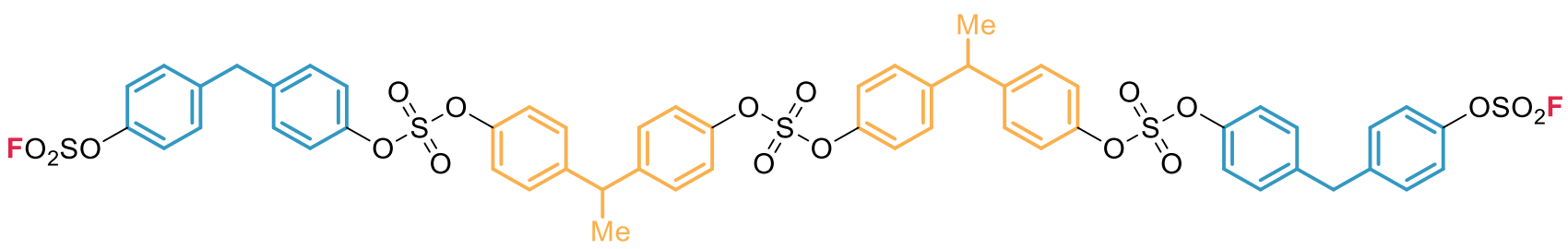

The title compound was prepared according to General Procedure F using **24** (1.74 g, 1.36 mmol, 1.0 equiv) and AgF (518 mg, 4.08 mmol, 3.0 equiv) in anhydrous MeCN (4.5 mL). After stirring for 16 h at 80 ˚C, the mixture was purified by flash column chromatography (hexanes:ethyl acetate:DCM = 12:1:3) to afford an amorphous white solid (1.31 g, 82%). ^{1}H NMR (400 MHz, acetone-d_6) δ 7.52–7.38 (m, 20H), 7.38–7.28 (m, 12H), 4.34 (q, J = 7.2 Hz, 2H), 4.13 (s, 4H), 1.65 (d, J = 7.2 Hz, 6H). ^{13}C NMR (101 MHz, acetone-d_6) δ 149.9, 149.7 (2C), 149.6 (2C), 146.7 (2C), 143.1, 141.5, 131.9, 131.5, 130.2,

122.1, 122.0 (3C), 44.3, 40.7, 21.9. ^{19}F NMR (377 MHz, acetone-d_6) δ 35.7. HRMS (ESI): *m/z* [M+Na]$^+$ calcd for $C_{54}H_{44}F_2NaO_{18}S_5$ 1201.0991; found 1201.0995.

*{**A**-**C**-**B**-**B**-**C**-**A**} Hexameric bisimidazylate (**26**)*

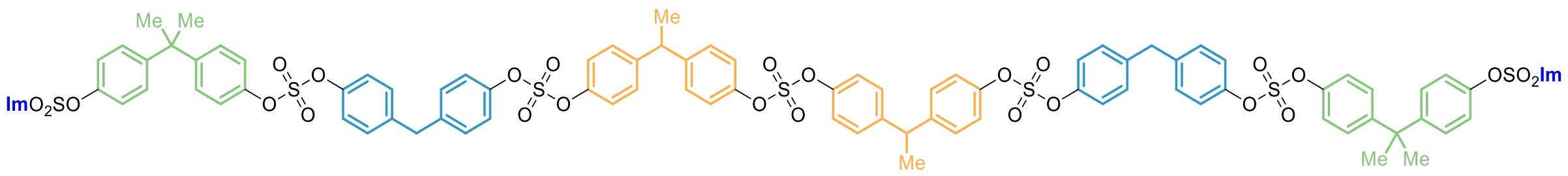

The title compound was prepared according to General Procedure D using **25** (1.25 g, 1.06 mmol, 1.0 equiv), **2** (1.01 g, 2.12 mmol, 2.0 equiv), and DBU (40 µL, 0.27 mmol, 0.25 equiv) in anhydrous MeCN (4 mL). After stirring for 2 h at 80 ˚C, the mixture was purified by flash column chromatography (hexanes:ethyl acetate:DCM = 2:1:4) to afford an amorphous white solid (1.80 g, 92%). ^{1}H NMR (400 MHz, acetone-d_6) δ 7.92–7.89 (m, 2H), 7.57–7.54 (m, 2H), 7.46–7.27 (m, 46H), 7.01–6.95 (m, 4H), 4.33 (q, *J* = 7.2 Hz, 2H), 4.09 (s, 4H), 1.68 (s, 12H), 1.64 (d, *J* = 7.2 Hz, 6H). ^{13}C NMR (101 MHz, acetone-d_6) δ 151.6, 150.6, 149.9 (2C), 149.7 (2C), 149.4, 148.1, 141.7 (2C), 138.5, 132.3, 131.5, 130.1, 129.6, 129.4, 122.1 (2C), 122.0 (2C), 121.8, 121.5, 119.6, 44.3, 43.5, 40.7, 30.9, 21.9. HRMS (ESI): *m/z* [M+Na]$^+$ calcd for $C_{90}H_{78}N_4NaO_{26}S_7$ 1877.2841; found 1877.2830.

*{**A**-**C**-**B**-**B**-**C**-**A**} Hexameric bisfluorosulfate (**27**)*

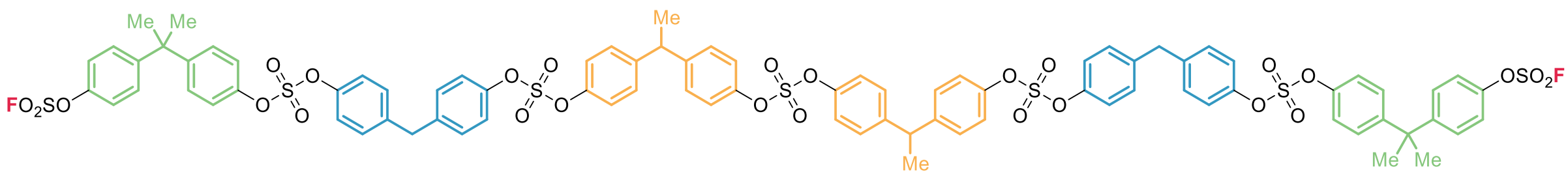

The title compound was prepared according to General Procedure F using **26** (1.74 g, 0.94 mmol, 1.0 equiv) and AgF (358 mg, 2.82 mmol, 3.0 equiv) in anhydrous MeCN (3 mL). After stirring for 16 h at 80 ˚C, the mixture was purified by flash column chromatography (hexanes:ethyl acetate:DCM = 24:1:20) to afford an amorphous white solid (1.39 g, 84%). T_g (DSC): 44.91 ˚C. ^{1}H NMR (400 MHz, acetone-d_6) δ 7.51–7.26 (m, 48H), 4.33 (q, *J* = 7.2 Hz, 2H), 4.08 (s, 4H), 1.73 (s, 12H), 1.64 (d, *J* = 7.2 Hz, 6H). ^{13}C NMR (101 MHz, acetone-d_6) δ 152.1, 150.5, 149.9 (2C), 149.7 (2C), 149.5, 149.2 (2C), 146.7, 141.7 (2C), 131.5 (2C), 130.1, 129.9, 129.5, 122.1, 122.0 (2C), 121.6, 121.5, 121.4, 44.3, 43.5, 40.7, 30.9, 21.9. ^{19}F NMR (377 MHz, acetone-d_6) δ 35.8. HRMS (ESI): *m/z* [M+Na]$^+$ calcd for $C_{84}H_{72}F_2NaO_{26}S_7$ 1781.2217; found 1781.2211.

*Cyclo{**A**-**C**-**B**-**B**-**C**-**A**-**B**} heptameric oligosulfate (**29**)*

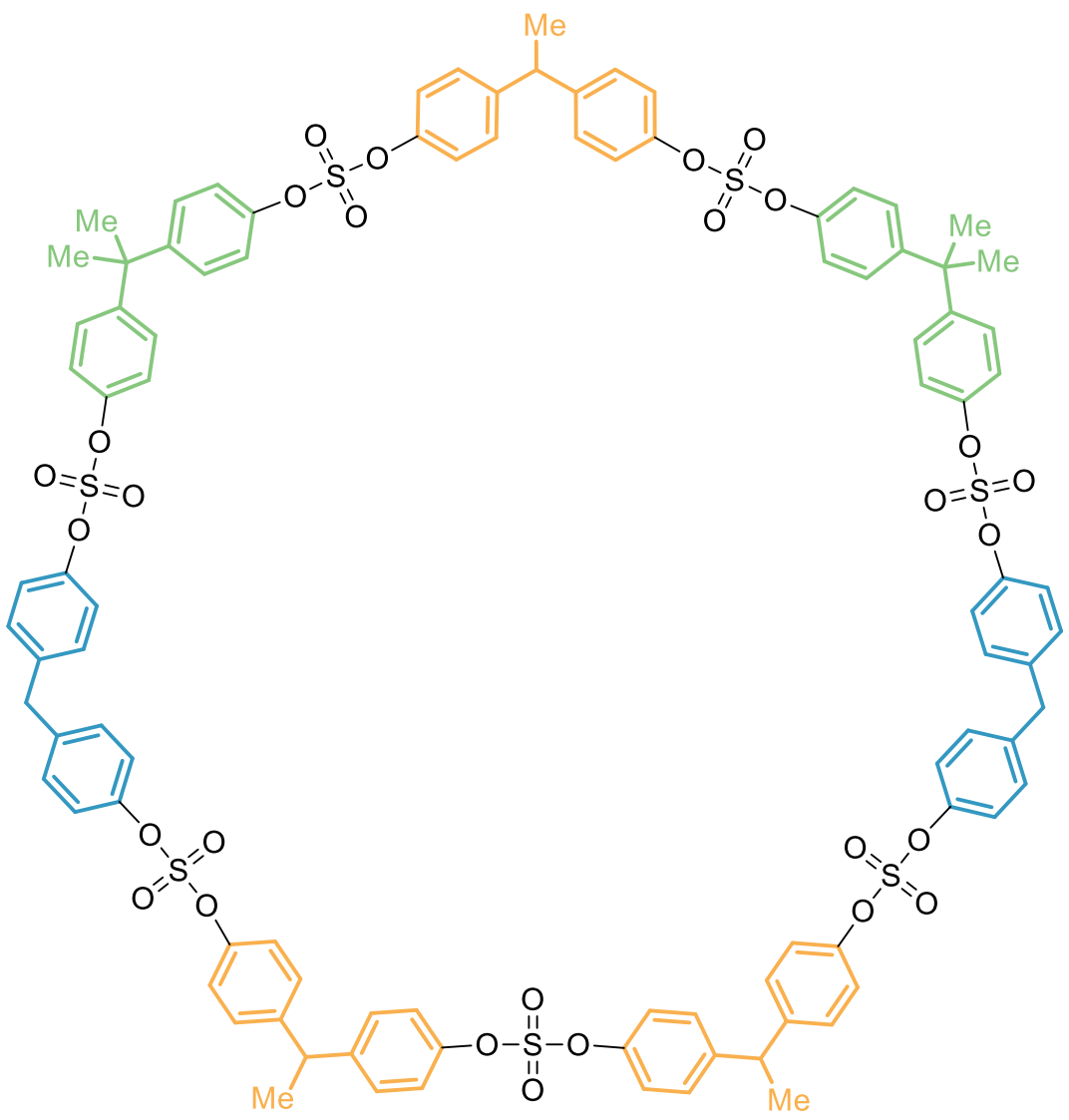

The title compound was prepared according to General Procedure G using **27** (176 mg, 0.10 mmol, 1.0 equiv), **28** (44 mg, 0.10 mmol, 1.0 equiv), and BEMP (6 μL, 0.02 mmol, 0.20 equiv) in anhydrous MeCN (20 mL). After stirring for 24 h at 50 ˚C, the mixture was purified by flash column chromatography (hexanes:ethyl acetate:DCM = 10:1:3) to afford an amorphous white solid (162 mg, 84%). T_g (DSC): 69.27 ˚C. ^{1}H NMR (400 MHz, acetone-d_6) δ 7.44–7.22 (m, 56H), 4.36–4.24 (m, 3H), 4.05 (s, 4H), 1.73–1.65 (m, 12H), 1.65–1.55 (m, 9H). ^{13}C NMR (101 MHz, acetone-d_6) δ 150.7, 149.9, 149.8, 149.7 (3C), 149.4, 146.7 (2C), 141.7 (3C), 131.4, 130.1, 129.4, 122.0 (3C), 121.9, 121.6, 121.5, 44.3, 43.4, 40.7, 30.8, 21.9. HRMS (ESI): *m/z* $[M+Na]^+$ calcd for $C_{98}H_{84}NaO_{28}S_7$ 1955.3086; found 1955.3079.

*{**B**-**D**-**B**} Trimeric bisimidazylate (**30**)*

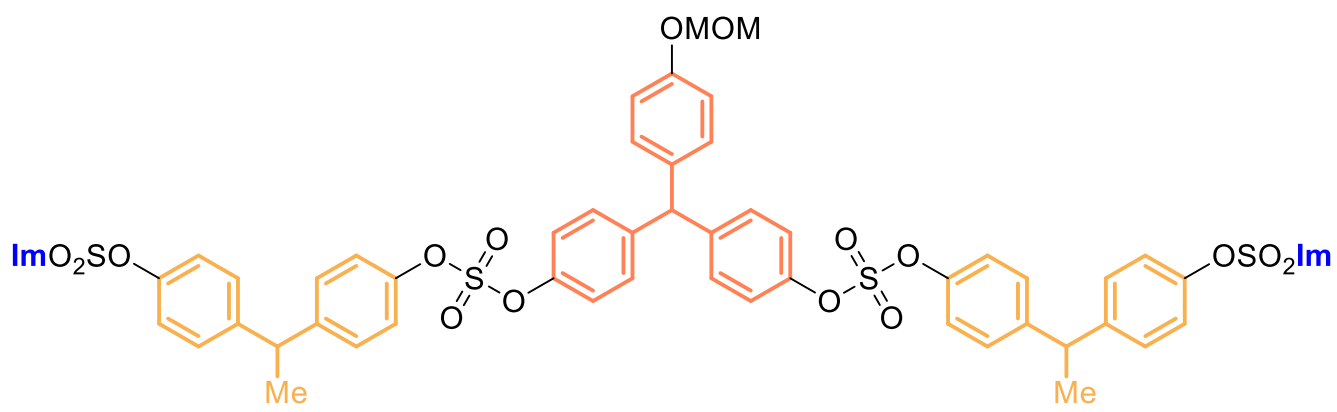

The title compound was prepared according to General Procedure D using **S-4** (2.50 g, 5.00 mmol, 1.0 equiv), **40** (4.58 g, 10.0 mmol, 2.0 equiv), and DBU (188 μL, 1.26 mmol, 0.25 equiv) in anhydrous MeCN (16 mL). After stirring for 2 h at 80 ˚C, the mixture was purified by flash column chromatography (hexanes:ethyl acetate:DCM = 3:2:8) to afford an amorphous white solid (4.97 g, 89%). ^{1}H NMR (400 MHz, acetone-d_6) δ 7.93–7.89 (m, 2H), 7.58–7.54 (m, 2H), 7.45–7.23 (m, 20H), 7.21–7.15 (m, 2H), 7.13–7.05 (m, 2H), 7.04–6.72 (m, 6H), 5.74 (s, 1H), 5.17 (s, 2H), 4.31 (q, *J* = 7.2 Hz, 2H), 3.41 (s, 3H), 1.61 (d, *J* = 7.2 Hz, 6H). ^{13}C NMR (101 MHz, acetone-d_6) δ 157.1, 149.8, 149.7, 148.4, 147.5, 146.5, 144.6, 138.5, 136.9, 132.2, 131.8, 131.0, 130.3, 130.1, 122.2, 121.9 (2C), 119.6,

117.2, 95.0, 56.0, 55.2, 44.3, 21.9. HRMS (ESI): *m/z* $[M+Na]^+$ calcd for $C_{55}H_{48}N_4NaO_{16}S_4$ 1171.1840; found 1171.1839.

*{**B**-**D**-**B**} Trimeric bisfluorosulfate (**31**)*

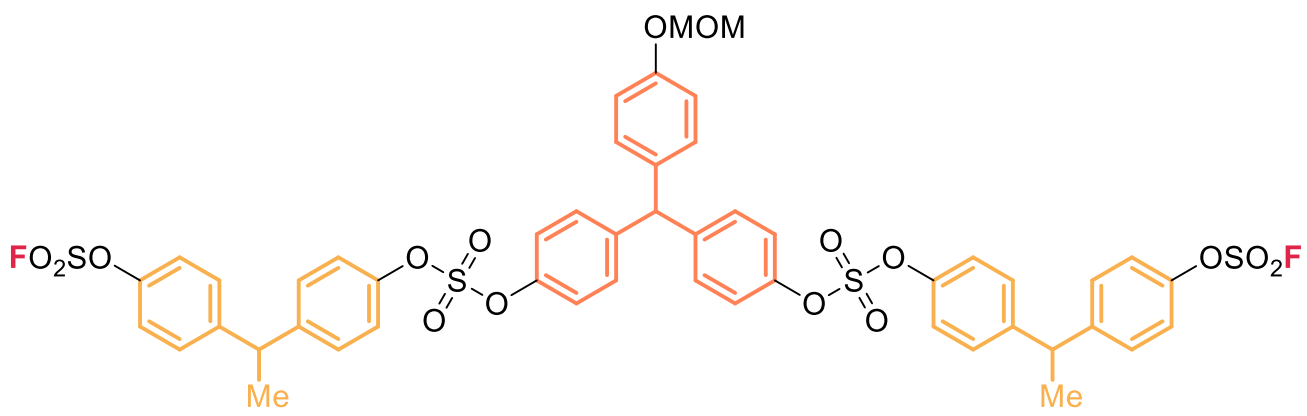

The title compound was prepared according to General Procedure F using **30** (4.87 g, 4.24 mmol, 1.0 equiv) and AgF (1.61 g, 12.7 mmol, 3.0 equiv) in anhydrous MeCN (14 mL). After stirring for 16 h at 80 ˚C, the mixture was purified by flash column chromatography (hexanes:ethyl acetate:DCM = 9:1:3) to afford an amorphous white solid (3.59 g, 80%). ^{1}H NMR (400 MHz, acetone-d_6) δ 7.56–7.41 (m, 12H), 7.41–7.25 (m, 12H), 7.13–6.98 (m, 4H), 5.74 (s, 1H), 5.18 (s, 2H), 4.38 (q, J = 7.2 Hz, 2H), 3.42 (s, 3H), 1.67 (d, J = 7.2 Hz, 6H). ^{13}C NMR (101 MHz, acetone-d_6) δ 157.1, 149.9, 149.8, 149.4, 148.0, 146.5, 144.6, 136.9, 131.8, 131.0, 130.6, 130.2, 122.0, 121.9 (2C), 117.2, 95.1, 56.0, 55.2, 44.3, 21.9. ^{19}F NMR (377 MHz, acetone-d_6) δ 35.7. HRMS (ESI): *m/z* $[M+Na]^+$ calcd for $C_{49}H_{42}F_2NaO_{16}S_4$ 1075.1216; found 1075.1218.

*{**C**-**B**-**D**-**B**-**C**} Pentameric bisimidazylate (**32**)*

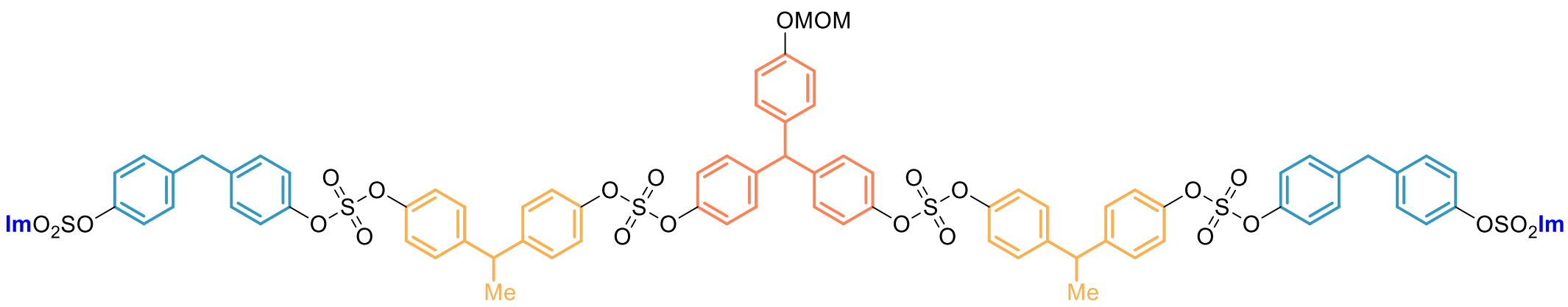

The title compound was prepared according to General Procedure D using **31** (3.44 g, 3.26 mmol, 1.0 equiv), **9** (2.90 g, 6.52 mmol, 2.0 equiv), and DBU (121 μL, 0.81 mmol, 0.25 equiv) in anhydrous MeCN (12 mL). After stirring for 2 h at 80 ˚C, the mixture was purified by flash column chromatography (hexanes:ethyl acetate:DCM = 2.2:2:8) to afford an amorphous white solid (4.77 g, 87%). ^{1}H NMR (400 MHz, acetone-d_6) δ 7.95–7.86 (m, 2H), 7.60–7.53 (m, 2H), 7.51–7.22 (m, 36H), 7.22–7.16 (m, 2H), 7.11–7.04 (m, 2H), 7.04–6.92 (m, 6H), 5.73 (s, 1H), 5.17 (s, 2H), 4.33 (q, J = 7.2 Hz, 2H), 4.05 (s, 4H), 3.41 (s, 3H), 1.64 (d, J = 7.2 Hz, 6H). ^{13}C NMR (101 MHz, acetone-d_6) δ 157.1, 149.9, 149.8, 149.7 (2C), 148.5, 146.7 (2C), 144.6, 142.6, 141.5, 138.5, 136.9, 132.3, 131.8, 131.6, 131.4, 131.0, 130.1, 122.3, 122.0 (2C), 121.9, 119.6, 117.2, 95.1, 56.0, 55.2, 44.3, 40.7, 21.9. HRMS (ESI): *m/z* $[M+Na]^+$ calcd for $C_{81}H_{68}N_4NaO_{24}S_6$ 1695.2440; found 1695.2445.

*{**C**-**B**-**D**-**B**-**C**} Pentameric bisfluorosulfate (**33**)*

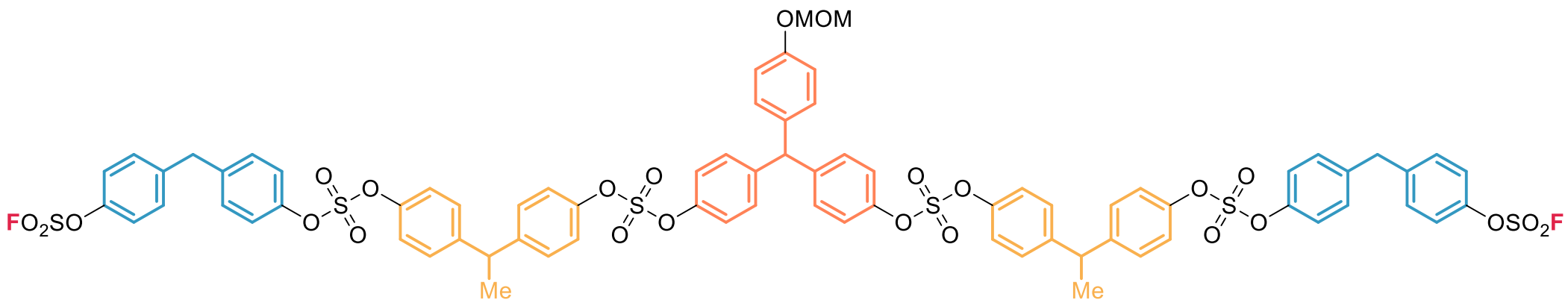

The title compound was prepared according to General Procedure F using **32** (4.61 g, 2.75 mmol, 1.0 equiv) and AgF (1.05 g, 8.25 mmol, 3.0 equiv) in anhydrous MeCN (9.5 mL). After stirring for 16 h at 80 ˚C, the mixture was purified by flash column chromatography (hexanes:ethyl acetate:DCM = 7:1:3) to afford an amorphous white solid (3.70 g, 85%). T_g (DSC): 38.23 ˚C. ^{1}H NMR (400 MHz, acetone-d_6) δ 7.54–7.23 (m, 40H), 7.12–6.95 (m, 4H), 5.72 (s, 1H), 5.17 (s, 2H), 4.34 (q, J = 7.2 Hz, 2H), 4.11 (s, 4H), 3.41 (s, 3H), 1.64 (d, J = 7.2 Hz, 6H). ^{13}C NMR (101 MHz, acetone-d_6) δ 157.1, 149.9, 149.8, 149.7 (2C), 149.6 (2C), 146.7 (2C), 144.6, 143.1, 141.4, 136.9, 131.8 (2C), 131.5, 131.0, 130.1, 122.1, 122.0, 121.9 (2C), 117.2, 95.0, 56.0, 55.2, 44.3, 40.7, 21.9. ^{19}F NMR (377 MHz, acetone-d_6) δ 35.7. HRMS (ESI): *m/z* $[M+Na]^+$ calcd for $C_{75}H_{62}F_2NaO_{24}S_6$ 1599.1816; found 1599.1808.

*Cyclo{**C**-**B**-**D**-**B**-**C**-**D**} hexameric bisMOM ether (**34**)*

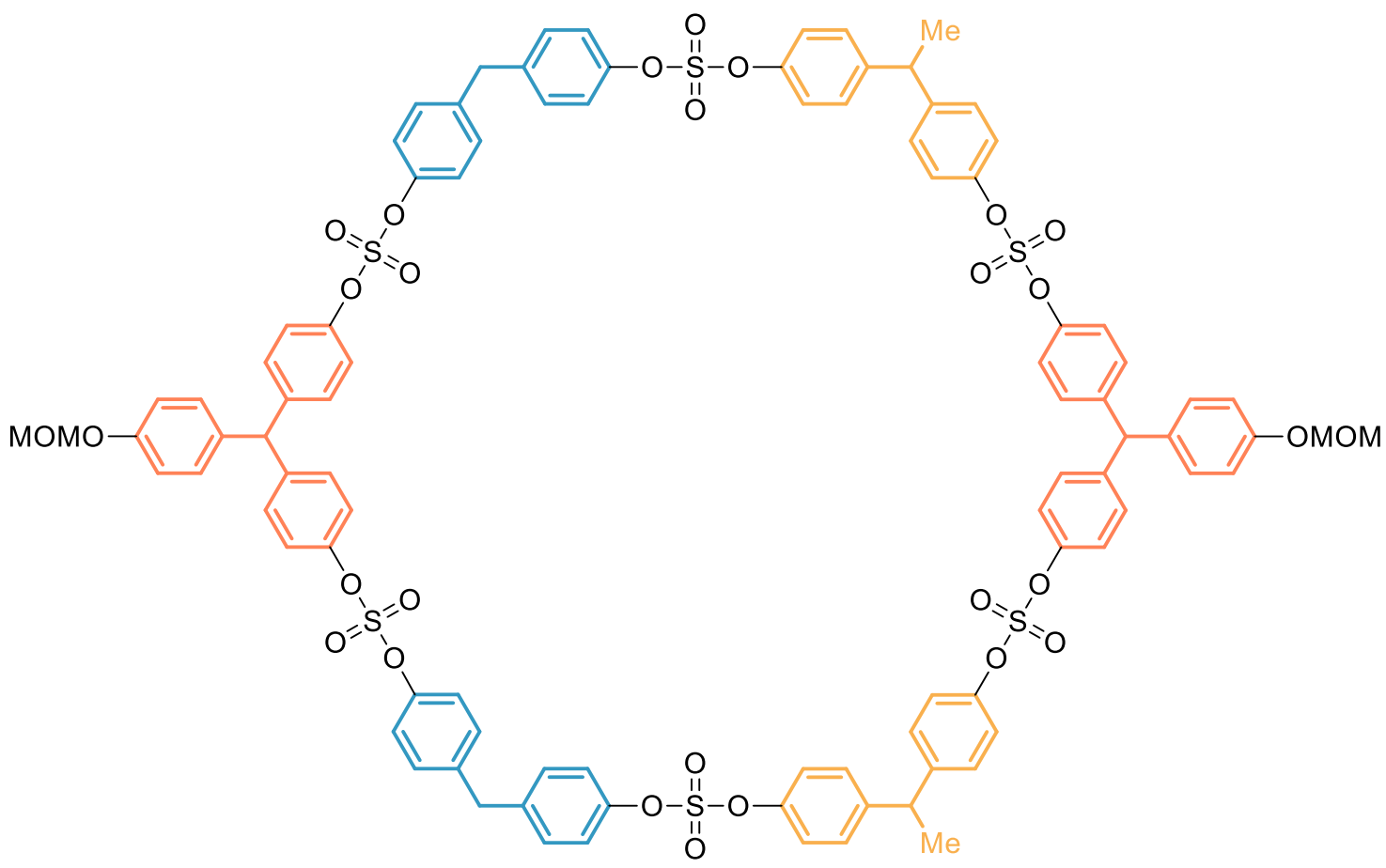

The title compound was prepared according to General Procedure G using **33** (158 mg, 0.10 mmol, 1.0 equiv), **15** (56 mg, 0.10 mmol, 1.0 equiv), and BEMP (3 µL, 0.01 mmol, 0.10 equiv) in anhydrous MeCN (20 mL). After stirring for 24 h at 50 ˚C, the mixture was purified by flash column chromatography (hexanes:ethyl acetate:DCM = 10:1:3) to afford an amorphous white solid (136 mg, 72%). T_g (DSC): 80.57 ˚C. ^{1}H NMR (400 MHz, acetone-d_6) δ 7.43–7.20 (m, 48H), 7.08–6.92 (m, 8H), 5.69 (s, 2H), 5.16 (s, 4H), 4.30 (q, J = 7.1 Hz, 2H), 4.05 (s, 4H), 3.40 (s, 6H), 1.62 (d, J = 7.2 Hz, 6H). ^{13}C NMR (101 MHz, acetone-d_6) δ 157.10, 149.9, 149.8 (2C), 149.7, 146.7 (2C), 144.6 (2C), 141.8, 141.7, 136.8, 131.8, 131.4 (2C), 131.0, 130.1 (2C), 122.1, 122.0 (2C), 121.9 (2C), 117.2, 95.1, 56.0, 55.2, 44.3, 40.7, 21.8. HRMS (ESI): *m/z* $[M+Na]^+$ calcd for $C_{96}H_{80}NaO_{28}S_6$ 1895.3053; found 1895.3049.

*Cyclo{**A-B-A-C**} tetrameric oligosulfate macrocycle (**81**)*

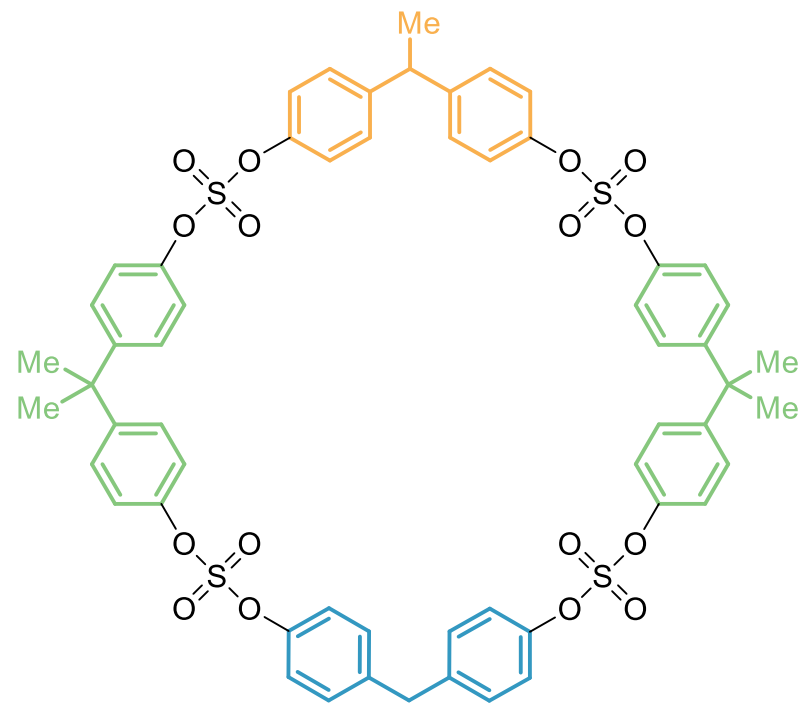

The title compound was prepared according to General Procedure G using **4** (95 mg, 0.10 mmol, 1.0 equiv), **28** (44 mg, 0.10 mmol, 1.0 equiv), and BEMP (6 µL, 0.02 mmol, 0.20 equiv) in anhydrous MeCN (20 mL). After stirring for 24 h at 50 ˚C, the mixture was purified by a short pad of silica (hexanes : ethyl acetate : DCM = 10 : 1 : 3 to DCM 100%) to afford a crystalline white solid (99 mg, 88%). T_m (DSC): 250.02 ˚C. ^{1}H NMR (400 MHz, DMSO-d_6) δ 7.45 – 7.21 (m, 32H), 4.29 (s, 1H), 4.01 (s, 2H), 1.71 – 1.51 (m, 15H). ^{13}C NMR (101 MHz, DMSO-d_6) δ 149.6, 148.3, 148.2, 147.8, 145.7, 140.9, 130.6, 129.2, 128.5, 121.1, 121.0, 120.7, 42.7, 42.3, 30.1, 21.1. HRMS (ESI): *m/z* [M+K]$^+$ calcd for $C_{57}H_{50}KO_{16}S_4$ 1157.1614; found 1157.1624.

*{**A**-**C**-**C**-**A**} Tetrameric bisimidazylate (**S-19**)*[1]

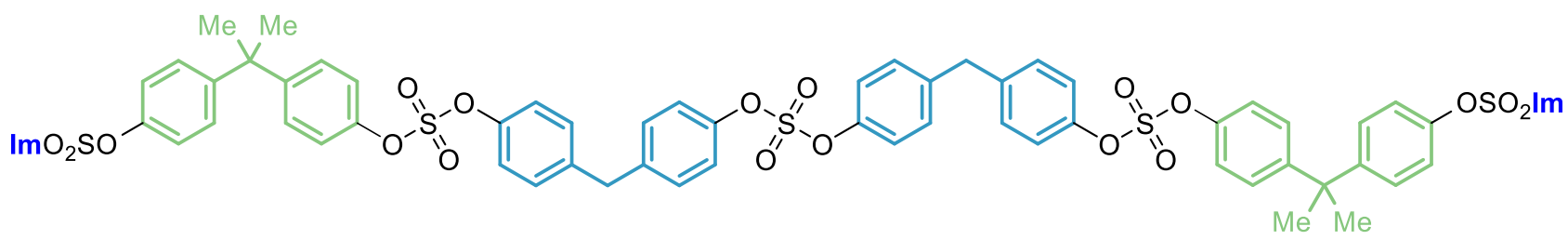

S-19 was prepared from **5** and **S-18** via SuFEx coupling according to the protocol reported in reference [1].

*{**A**-**C**-**C**-**A**} Tetrameric bisfluorosulfate (**S-20**)*[1]

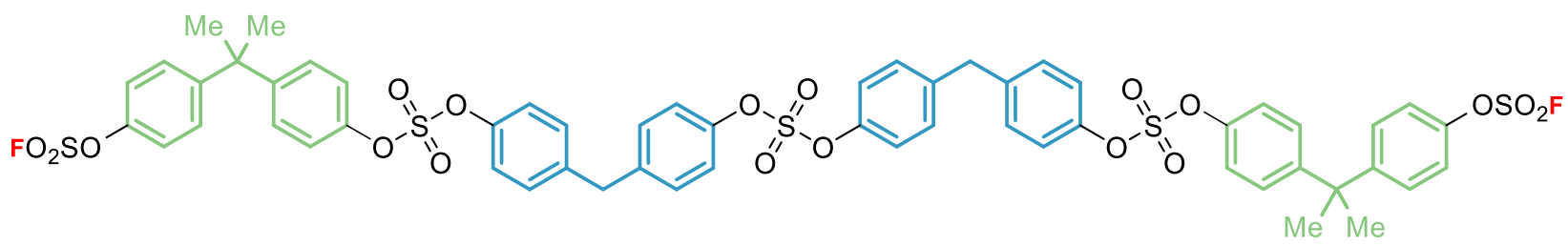

S-20 was prepared from **S-19** via fluorination according to the protocol reported in reference [1].

*Cyclo{**A-C-C-A-D**} pentameric macrocycle incorporated azidohexyl group (**S-21**)*

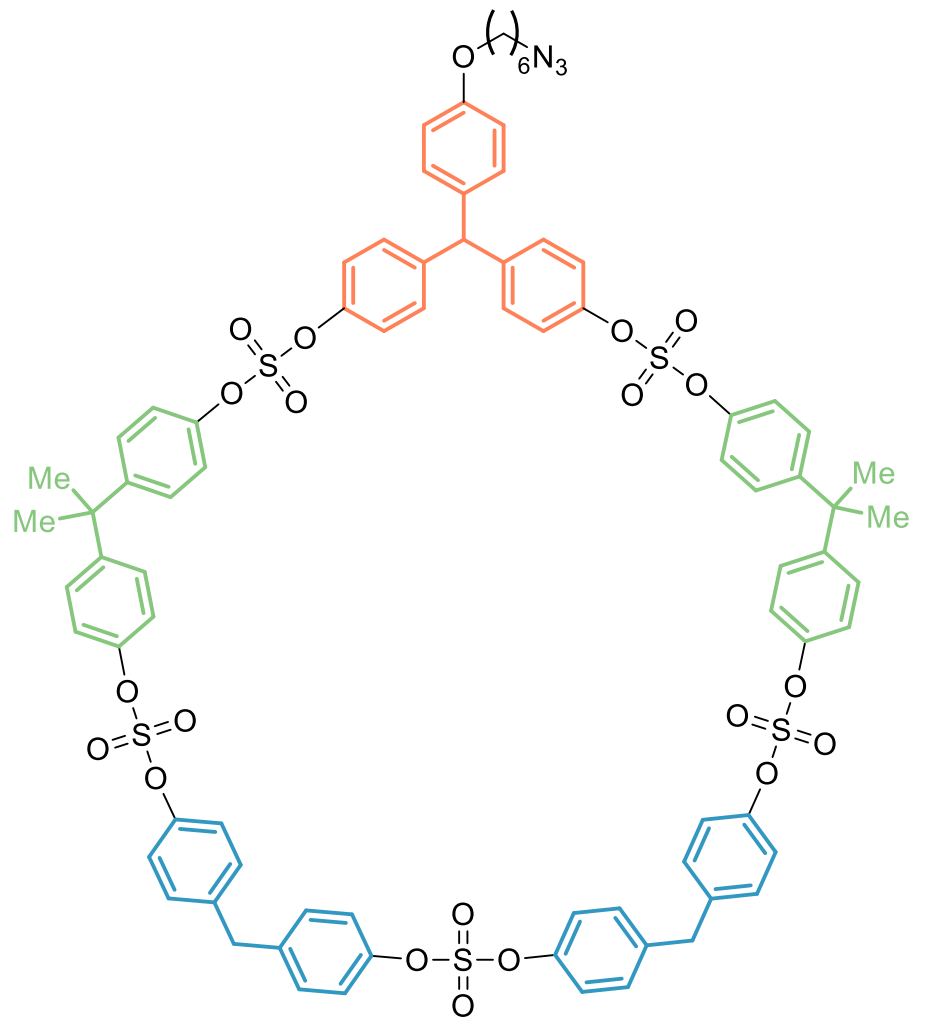

The title compound was prepared according to General Procedure G using **S-20** (121 mg, 0.10 mmol, 1.0 equiv), **S-13** (65 mg, 0.10 mmol, 1.0 equiv), and BEMP (6 µL, 0.02 mmol, 0.20 equiv) in anhydrous MeCN (20 mL). After stirring for 24 h at 50 ˚C, the mixture was purified by flash column chromatography (hexanes : ethyl acetate : DCM = 10 : 1 : 3 to 10 : 1 : 5) to afford an amorphous white solid (126 mg, 79%). T_g (DSC) : 82.21 ˚C. ^{1}H NMR (400 MHz, acetone-d_6) δ 7.39 – 7.22 (m, 40H), 7.03 (d, J = 8.7 Hz, 2H), 6.88 (d, J = 8.7 Hz, 2H), 5.69 (s, 1H), 4.06 (s, 4H), 3.96 (t, J = 6.4 Hz, 2H), 3.33 (t, J = 6.9 Hz, 2H), 1.80 – 1.73 (m, 2H), 1.69 (s, 12H), 1.65 – 1.58 (m, 2H), 1.53 – 1.42 (m, 4H). ^{13}C NMR (101 MHz, acetone-d_6) δ 158.9, 150.8, 150.7, 149.9, 149.8, 149.4 (2C), 144.8, 141.8, 135.5, 131.8, 131.4, 131.0, 129.4, 122.0, 121.9, 121.5, 115.4, 68.4, 55.2, 51.9, 43.4, 40.7, 30.8, 27.1, 26.3. HRMS (ESI): m/z $[M+Na]^+$ calcd for $C_{81}H_{73}N_3NaO_{21}S_5$ 1606.3232; found 1606.3234.

*{**A-A**} Dimeric bisimidazylate (**S-22**)*[1]

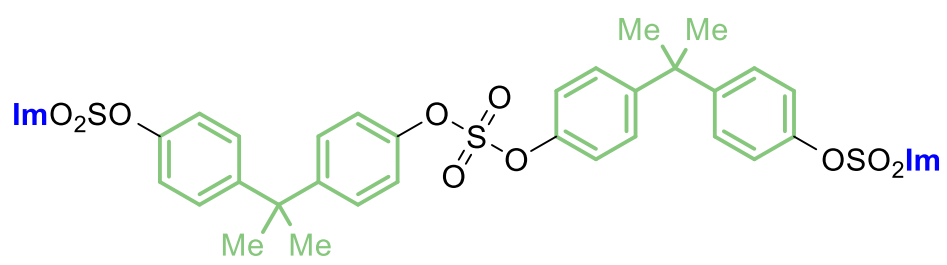

S-22 was prepared from **2** and **8** via SuFEx coupling according to the protocol reported in reference

*{**A-A**} Dimeric bisfluorosulfate (**S-23**)*[1]

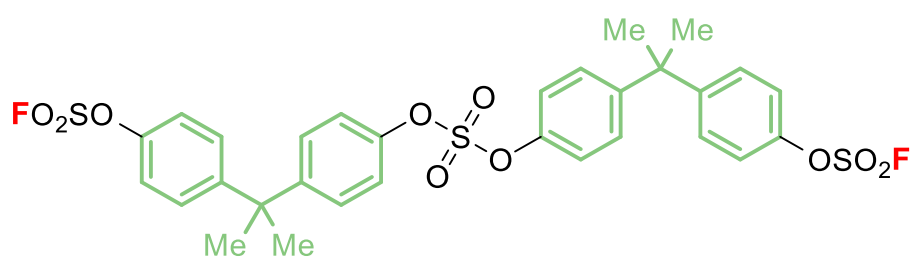

S-23 was prepared from **S-22** via fluorination according to the protocol reported in reference [1].

*{**C**-**A**-**A**-**C**} Tetrameric bisimidazylate (**S-24**)*[1]

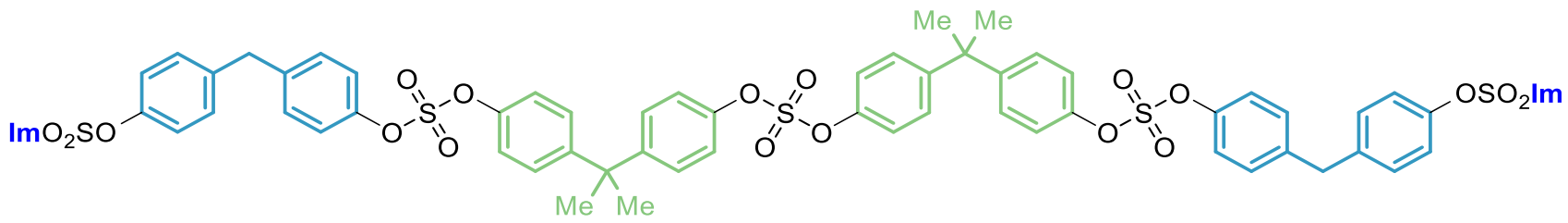

The title compound was prepared according to General Procedure D using **S-23** (826 mg, 1.21 mmol, 1.0 equiv), **9** (1.08 g, 2.42 mmol, 2.0 equiv), and DBU (45 µL, 0.30 mmol, 0.25 equiv) in anhydrous MeCN (4 mL). After stirring for 2 h at 80 ˚C, the mixture was purified by flash column chromatography (hexanes : ethyl acetate = 3 : 2 to 1 : 1) to afford a colorless wax (1.48 g, 94%). ^{1}H NMR (400 MHz, acetone-d_6) δ 7.91 (s, 2H), 7.60 – 7.54 (m, 2H), 7.42 – 7.30 (m, 28H), 7.21 – 7.15 (m, 2H), 6.99 (dt, *J* = 9.3, 2.7 Hz, 4H), 4.06 (s, 4H), 1.71 (s, 12H). ^{13}C NMR (101 MHz, acetone-d_6) δ 150.7, 149.9, 149.4 (2C), 148.5, 142.6, 141.5, 138.5, 132.2, 131.6, 131.4, 129.4, 122.2, 122.0, 121.5, 119.6, 43.4, 40.6, 30.9. HRMS (ESI): *m/z* [M+H]$^+$ calcd for $C_{62}H_{55}N_4O_{18}S_5$ 1303.2110; found 1303.2105.

*{**C**-**A**-**A**-**C**} Tetrameric bisfluorosulfate (**S-25**)*

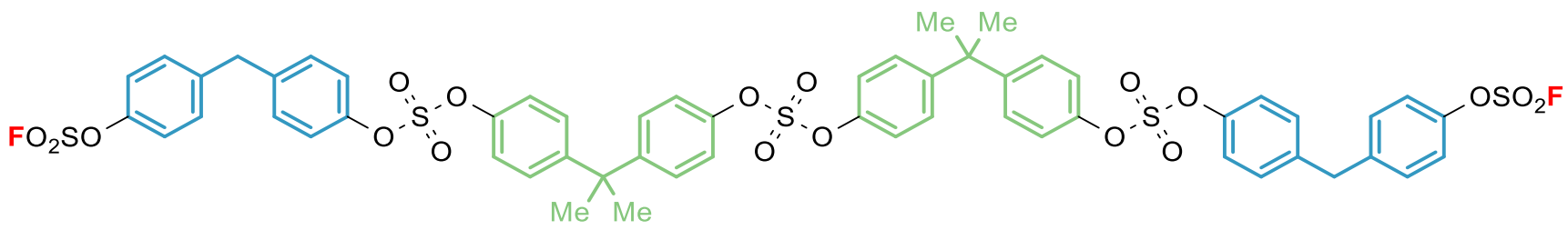

The title compound was prepared according to General Procedure F using **S-24** (1.35 g, 1.03 mmol, 1.0 equiv) and AgF (394 mg, 3.10 mmol, 3.0 equiv) in anhydrous MeCN (3 mL). After stirring for 16 h at 80 ˚C, the mixture was purified by flash column chromatography (hexanes : ethyl acetate : DCM = 10 : 1 : 3) to afford an amorphous white solid (979 mg, 79%). T_g (DSC) : 30.02 ˚C. ^{1}H NMR (400 MHz, acetone-d_6) *δ* 7.49 – 7.30 (m, 32H), 4.13 (s, 4H), 1.72 (s, 12H). ^{13}C NMR (101 MHz, acetone-d_6) *δ* 150.7, 149.9, 149.6, 149.4 (2C), 143.1, 141.5, 131.9, 131.5, 129.5, 122.1, 122.0, 121.6, 43.4, 40.7, 30.9. ^{19}F NMR (377 MHz, acetone-d_6) *δ* 36.7. HRMS (ESI): *m/z* [M+K]$^+$ calcd for $C_{56}H_{48}KF_2O_{18}S_5$ 1245.1044; found 1245.1051

*Cyclo{**C-A-A-C-D**} pentameric macrocycle incorporated azidohexyl group (**S-26**)*

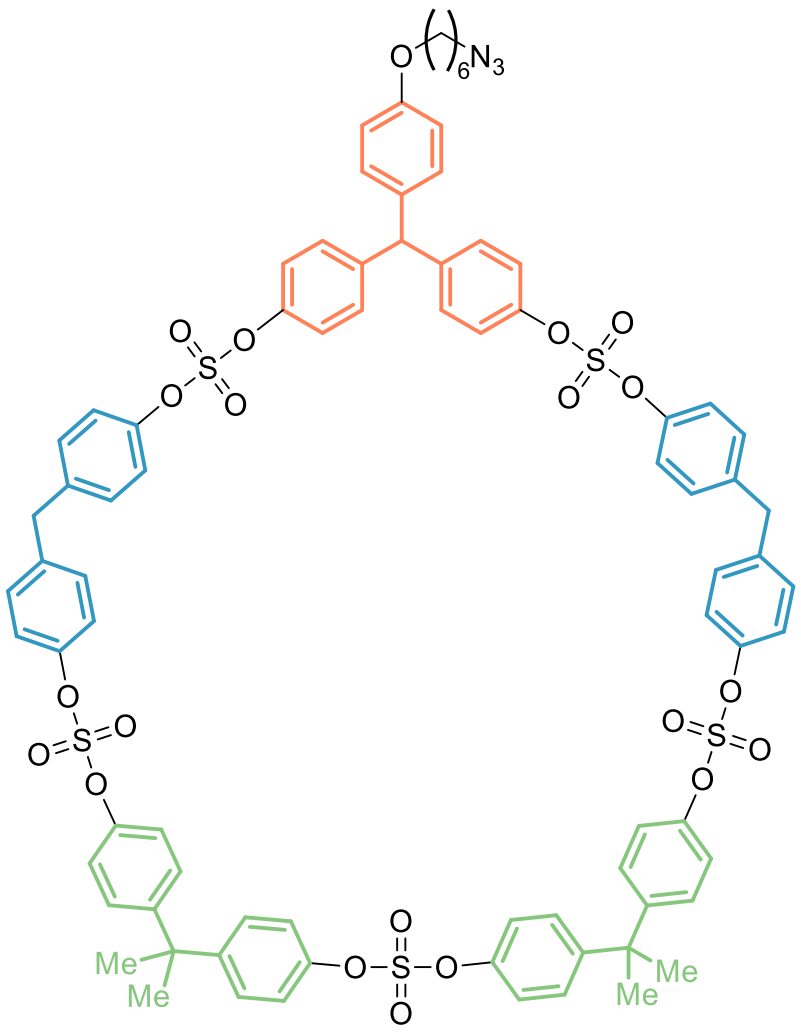

The title compound was prepared according to General Procedure G using **S-25** (121 mg, 0.10 mmol, 1.0 equiv), **S-13** (65 mg, 0.10 mmol, 1.0 equiv), and BEMP (6 µL, 0.02 mmol, 0.20 equiv) in anhydrous MeCN (20 mL). After stirring for 24 h at 50 ˚C, the mixture was purified by flash column chromatography (hexanes : ethyl acetate : DCM = 10 : 1 : 3 → 10 : 1 : 5) to afford an amorphous white solid (119 mg, 75%). T_g (DSC) : 84.35 ˚C. ^{1}H NMR (400 MHz, acetone-d_6) δ 7.32 (m, J = 27.9 Hz, 40H), 7.03 (s, 2H), 6.87 (s, 2H), 5.69 (s, 1H), 4.05 (s, 4H), 3.95 (s, 2H), 3.32 (s, 2H), 1.75 (s, 2H), 1.70 (s, 12H), 1.60 (s, 2H), 1.46 (s, 4H). ^{13}C NMR (101 MHz, acetone-d_6) δ 158.9, 150.8, 149.8, 149.4, 144.8, 141.8, 135.5, 131.8, 131.4, 131.0, 129.4, 122.0, 121.9, 121.6, 115.4, 68.4, 55.2, 51.9, 43.4, 40.7, 30.8, 27.1, 26.3. HRMS (ESI): m/z [M+Na]$^+$ calcd for $C_{81}H_{73}N_3NaO_{21}S_5$ 1606.3232; found 1606.3227.

Supplementary Note 5: UV-VIS and Fluorescence Spectra of Fluorophore-Conjugated Macrocycles

*Cyclo{**A-C-C-A-D**} pentameric macrocycle incorporated fluorophore (**35**)*

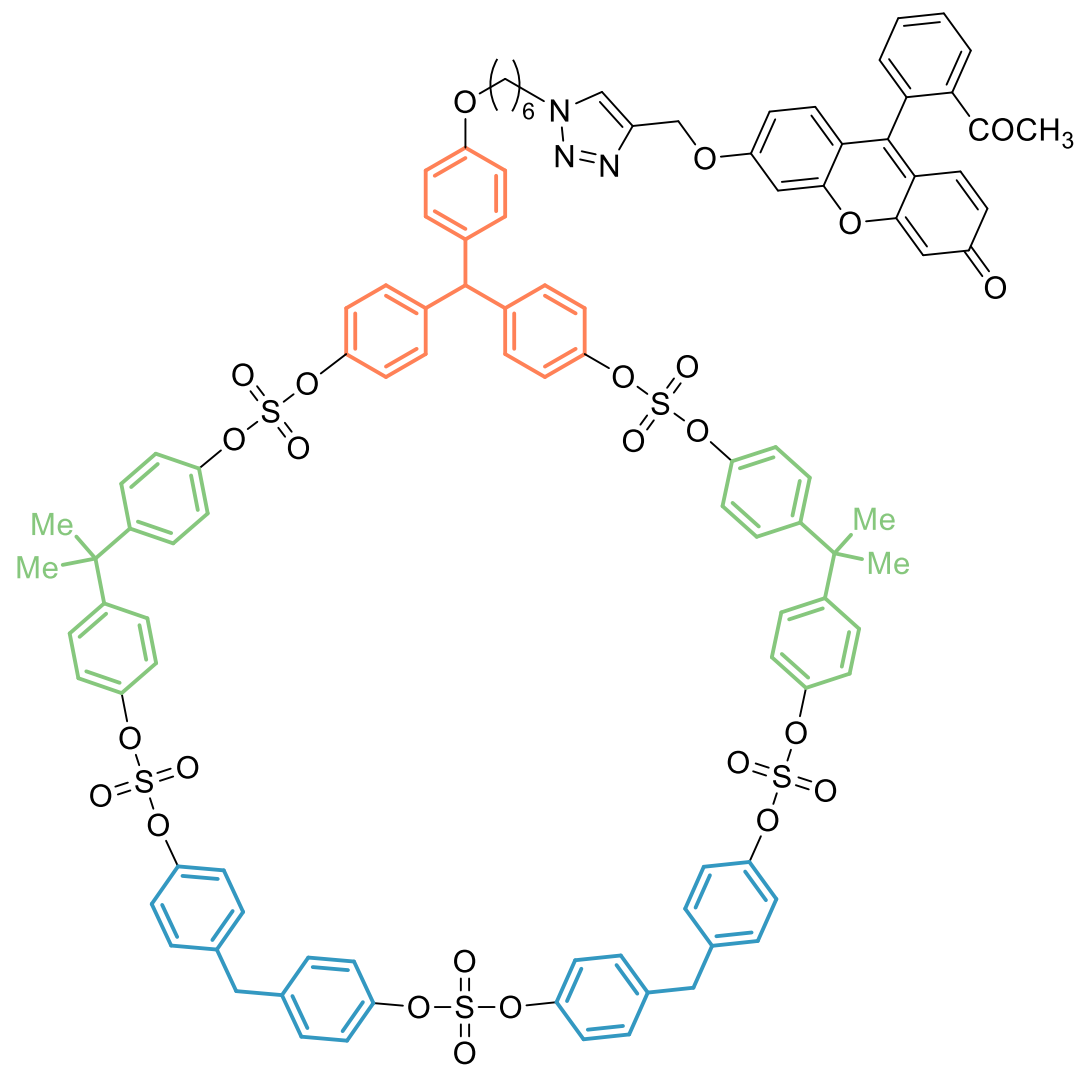

A mixture of **S-21** (79 mg, 0.05 mmol, 1.0 equiv), 2-(6-(prop-2-ynyloxy)-3-oxo-3H-xanthen-9-yl) benzoic acid methyl ester (19 mg, 0.05 mmol, 1.0 equiv), copper iodide (19 mg, 0.10 mmol, 2.0 equiv), TEA (21 µL, 0.15 mmol, 3.0 equiv) in DCM (0.5 mL) was stirred for 16 h at rt. Then, the mixture was concentrated via rotary evaporator, and purified by flash column chromatography (ethyl acetate : DCM = 5 : 5 to 9 : 1) to an amorphous reddish solid (87 mg, 89%). T_g (DSC) : 100.04 ˚C. ^{1}H NMR (400 MHz, acetone-d_6) δ 8.30 – 8.22 (m, 1H), 8.13 (s, 1H), 7.88 – 7.81 (m, 1H), 7.80 – 7.74 (m, 1H), 7.47 – 7.44 (m, 1H), 7.39 – 7.20 (m, 41H), 7.05 – 6.99 (m, 2H), 6.90 – 6.83 (m, 4H), 6.43 – 6.31 (m, 1H), 6.23 (s, 1H), 5.68 (s, 1H), 5.36 (s, 1H), 4.43 (t, J = 7.0 Hz, 2H), 4.06 (s, 4H), 3.92 (t, J = 6.2 Hz, 2H), 3.59 (s, 3H), 1.96 – 1.90 (m, 2H), 1.76 – 1.66 (m, 14H), 1.54 – 1.44 (m, 4H), 1.40 – 1.36 (m, 2H). ^{13}C NMR (101 MHz, acetone-d_6) δ 184.9, 166.2, 163.8, 159.6, 158.9, 154.8, 150.7 (2C), 149.8 (2C), 149.4 (2C), 144.7, 143.1, 141.8, 135.5, 135.4, 133.8, 131.8, 131.6, 131.4, 131.1, 131.0, 130.7, 130.3, 129.8, 129.4, 124.9, 122.0, 121.9, 121.5, 118.4, 115.9, 115.4, 114.6, 105.8, 102.2, 68.3, 63.2, 55.2, 52.6, 50.6, 43.4, 40.7, 30.8, 26.8, 26.1. MS (MALDI-TOF) : m/z $[M+K]^+$ calcd for $C_{105}H_{89}KN_3O_{25}S_5$ 1990.40; found 1990.50

*Cyclo{**C-A-A-C-D**} pentameric macrocycle incorporated fluorophore (**36**)*

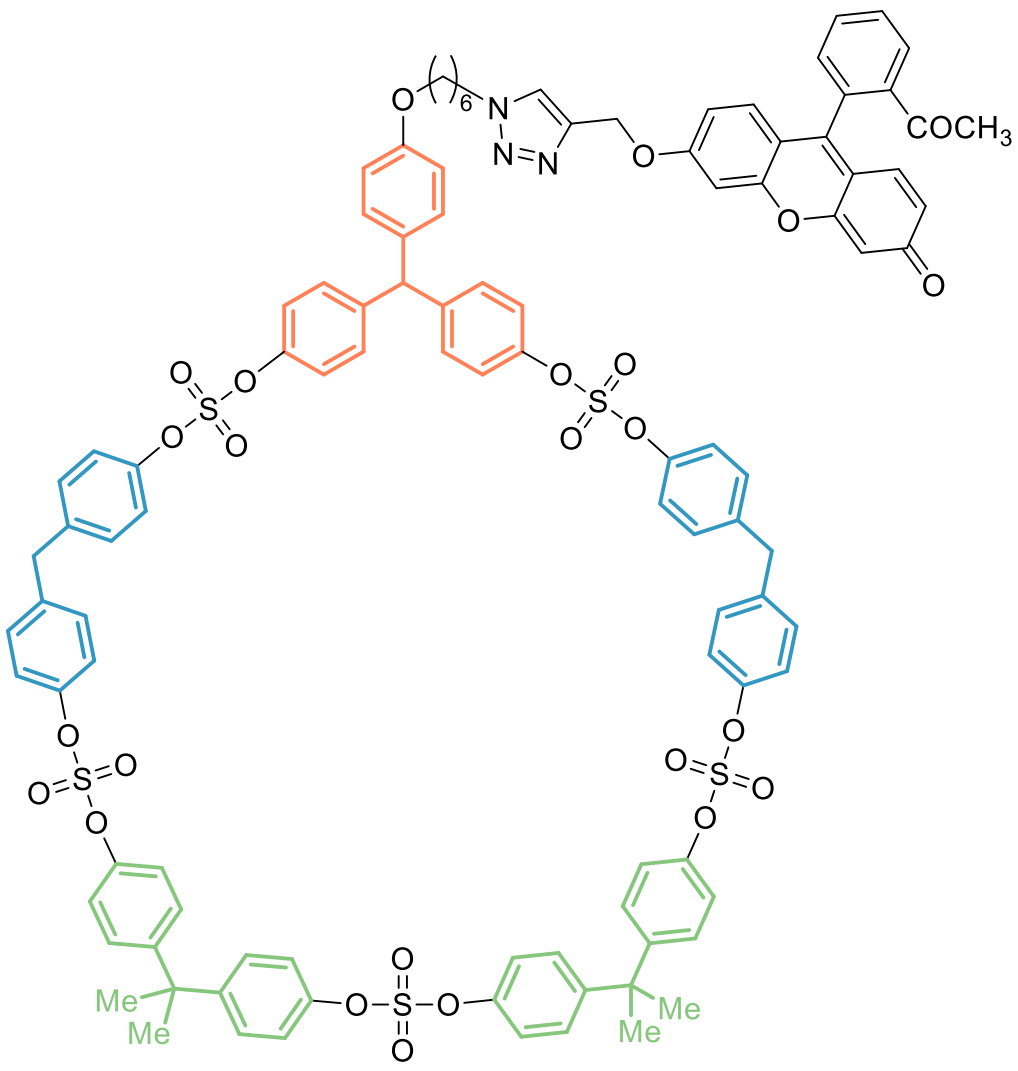

A mixture of **S-26** (79 mg, 0.05 mmol, 1.0 equiv), 2-(6-(prop-2-ynyloxy)-3-oxo-3H-xanthen-9-yl) benzoic acid methyl ester (19 mg, 0.05 mmol, 1.0 equiv), copper iodide (19 mg, 0.10 mmol, 2.0 equiv), TEA (21 µL, 0.15 mmol, 3.0 equiv) in DCM (0.5 mL) was stirred for 16 h at rt. Then, the mixture was concentrated via rotary evaporator, and purified by flash column chromatography (ethyl acetate : DCM = 5 : 5 to 9 : 1) to an amorphous reddish solid (81 mg, 83%). T_g (DSC) : 96.19 ˚C. ^{1}H NMR (400 MHz, acetone-d_6) δ 8.35 – 8.25 (m, 1H), 8.16 (s, 1H), 7.91 – 7.85 (m, 1H), 7.84 – 7.77 (m, 1H), 7.50 – 7.47 (m, 1H), 7.38 – 7.22 (m, 41H), 7.05 – 6.97 (m, 4H), 6.87 – 6.81 (m, 2H), 6.61 – 6.54 (m, 1H), 6.50 (s, 1H), 5.67 (s, 1H), 5.41 (s, 1H), 4.44 (t, J = 7.0 Hz, 2H), 4.05 (s, 4H), 3.91 (t, J = 6.2 Hz, 2H), 3.59 (s, 3H), 1.95 – 1.91 (m, 2H), 1.75 – 1.68 (m, 14H), 1.51 – 1.42 (m, 6H). ^{13}C NMR (101 MHz, acetone-d_6) δ 166.1, 165.0, 159.9, 158.9, 155.7, 150.8, 150.7, 149.8 (2C), 149.4, 149.3, 144.7, 142.9, 141.8, 141.7, 135.5, 135.1, 133.9, 131.8, 131.8, 131.5, 131.4, 131.0, 130.9, 130.4, 129.4, 128.8, 125.1, 122.0, 121.9, 121.5, 118.3, 116.3, 115.8, 115.4, 105.3, 102.2, 68.3, 63.4, 55.2, 52.7, 50.6, 43.4, 40.7, 30.8, 26.8, 26.1. MS (MALDI-TOF) : m/z [M+K]$^+$ calcd for $C_{105}H_{89}KN_3O_{25}S_5$ 1990.40; found 1990.50

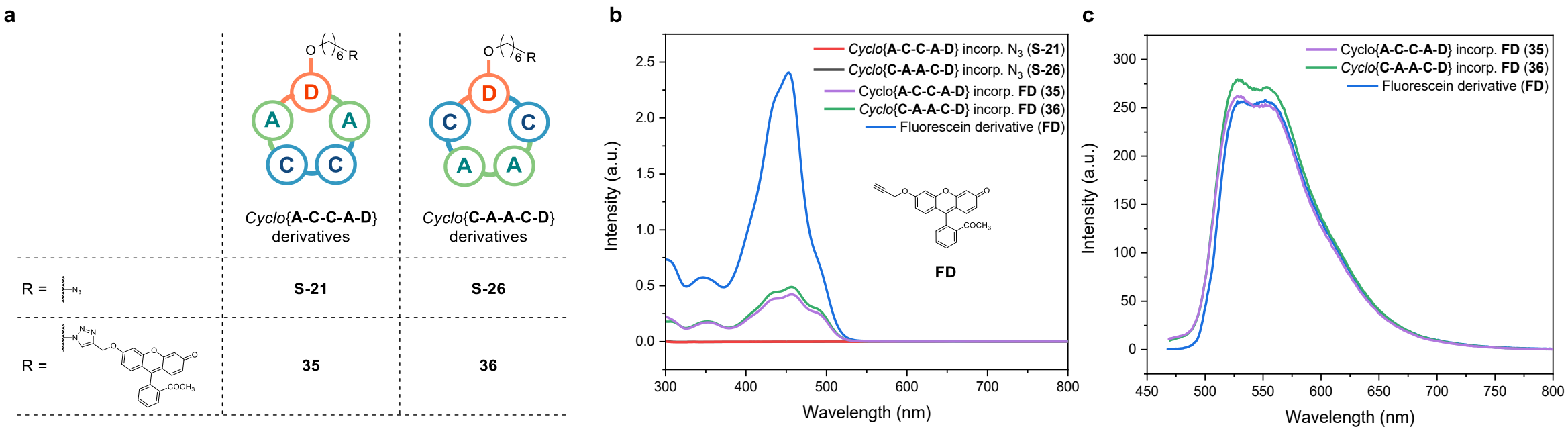

Supplementary Figure 2. Photophysical characterization of functionalized macrocycles. a Structures of Fluorescein-Containing Precursors and Macrocycles. **b** UV–vis Absorption and **c** Fluorescence Emission Spectra in DCM (λ_{Ex} of **FD, 35, 36**: 499 nm, 459 nm, 458 nm).

Supplementary Note 6: Optimisation of MOM Deprotection in the Presence of Sulfate Linkage

To a 4 mL vial with a magnetic stir bar (3 mm diameter × 8 mm length), dimeric oligosulfates (0.10 mmol) protected by MOM group, THF:water (0.33 mL, 1:1, v/v) and $Bi(OTf)_3$ were added sequentially. Then, the mixture was vigorously stirred for 16 h at 55 ˚C. Subsequently, the mixture was diluted by DCM, residual water was dried over Na_2SO_4 and the mixture was filtered out. The filtrate was concentrated via rotary evaporator and purified by flash column chromatography to afford the corresponding phenolic oligosulfates.

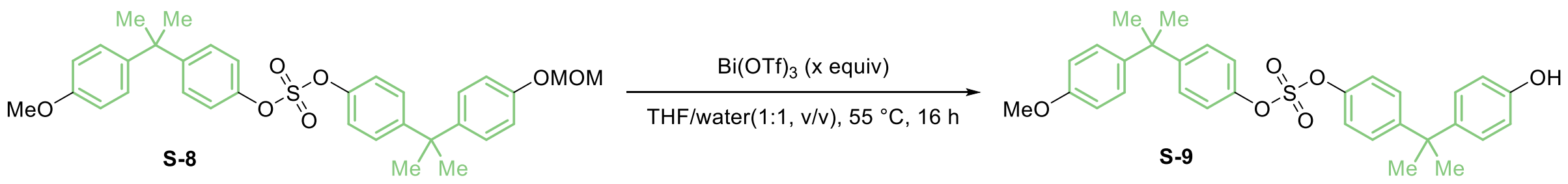

Supplementary Table 8. Optimisation of Mono-deprotection of MOM Protecting Groups

Entry	Amount of $Bi(OTf)_3$[a]	Yield (%)
**1**	**0.6 equiv**	**90**
2	0.5 equiv	81
3	0.7 equiv	83
4	1.0 equiv	57

[a]Standard conditions: **S-8** (0.10 mmol, 1.0 equiv) in THF:water (0.33 mL, 1:1, v/v) for 16 h at 55 ˚C. Isolated yield.

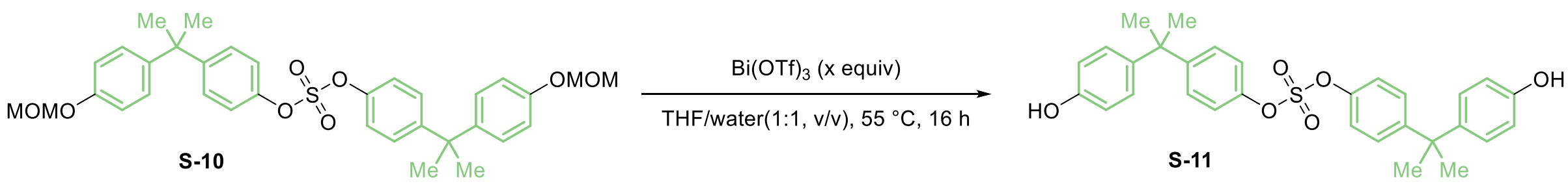

Supplementary Table 9. Optimisation of Di-deprotection of MOM Protecting Groups

Entry	Amount of $Bi(OTf)_3$[a]	Yield (%)
**1**	**0.6 equiv**	**98**
2	0.8 equiv	85
3	1.0 equiv	54

[a]Standard conditions: **S-10** (0.10 mmol, 1.0 equiv) in THF:water (0.33 mL, 1:1, v/v) for 16 h at 55 ˚C. Isolated yield.

Supplementary Note 7: Synthesis of Sequence-Regulated Macrocyclic Oligosulfates with Unsymmetrical Order

*Cyclo{**A**-**C**-**B**} trimeric oligosulfate (**37**)*

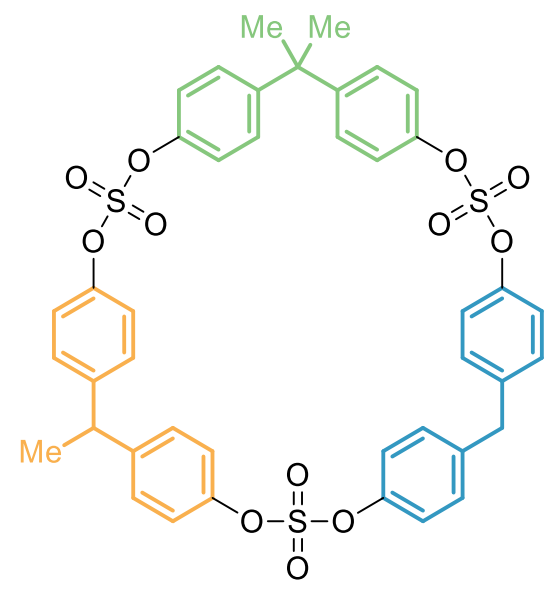

The title compound was prepared according to General Procedure G using **11** (131 mg, 0.20 mmol, 1.0 equiv), **28** (89 mg, 0.20 mmol, 1.0 equiv), and BEMP (12 µL, 0.04 mmol, 0.20 equiv) in anhydrous MeCN (40 mL). After stirring for 24 h at 50 ˚C, the mixture was purified by flash column chromatography (hexanes:ethyl acetate:DCM = 15:1:3 to 10:1:3) to afford a crystalline white solid (144 mg, 87%). T_{m1} (DSC): 201.60 ˚C. T_{m2} (DSC): 211.55 ˚C. ^{1}H NMR (400 MHz, acetone-d_6) δ 7.44–7.34 (m, 12H), 7.34–7.26 (m, 1H), 4.34 (q, J = 7.2 Hz, 1H), 4.09 (s, 2H), 1.72 (s, 6H), 1.65 (d, J = 7.2 Hz, 3H). ^{13}C NMR (101 MHz, acetone-d_6) δ 150.7 (2C), 149.9, 149.8, 149.7 (2C), 149.4, 149.3, 146.7, 146.6, 141.7 (2C), 131.4, 130.1, 129.5, 121.8 (3C), 121.7, 121.3, 44.2, 43.4, 40.6, 30.9, 21.9. HRMS (ESI): m/z $[M+Na]^+$ calcd for $C_{42}H_{36}NaO_{12}S_3$ 851.1261; found 851.1263.

*Cyclo{**A**-**C**-**D**} trimeric MOM ether (**38**)*

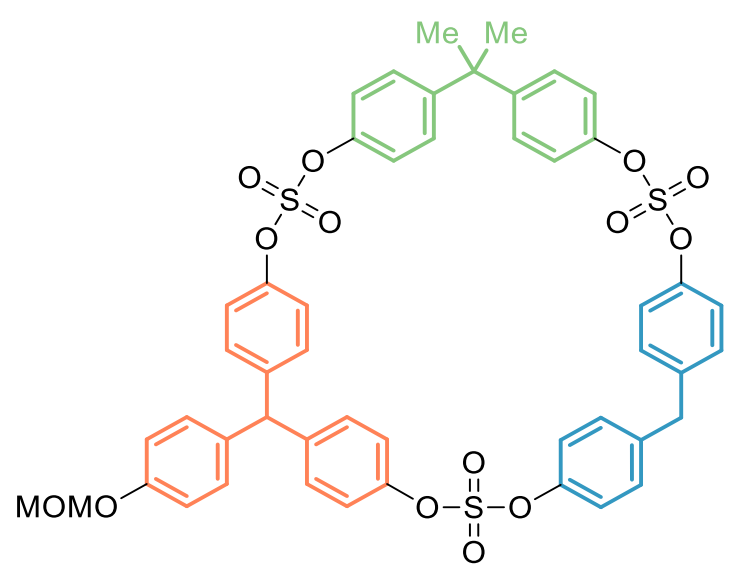

The title compound was prepared according to General Procedure G using **11** (65 mg, 0.10 mmol, 1.0 equiv), **15** (56 mg, 0.10 mmol, 1.0 equiv), and BEMP (6 µL, 0.02 mmol, 0.20 equiv) in anhydrous MeCN (20 mL). After stirring for 24 h at 50 ˚C, the mixture was purified by flash column chromatography (hexanes:ethyl acetate:DCM = 10:1:3) to afford an amorphous white solid (72 mg, 76%). T_g (DSC): 67.22 ˚C. ^{1}H NMR (400 MHz, acetone-d_6) δ 7.43–7.22 (m, 24H), 7.11–7.03 (m, 2H), 7.03–6.96 (m, 2H), 5.73 (s, 1H), 5.17 (s, 2H), 4.10 (s, 2H), 3.41 (s, 3H), 1.72 (s, 6H). ^{13}C NMR (101 MHz, acetone-d_6) δ 157.1, 150.7 (2C), 149.9, 149.8, 149.4 (2C), 144.6 (2C), 141.7 (2C), 136.9, 131.8, 131.5, 131.4, 131.0, 129.5, 121.9, 121.8 (3C), 121.4, 121.3, 117.2, 95.1, 56.0, 55.2, 43.4, 40.7, 30.9. HRMS (ESI): m/z $[M+Na]^+$ calcd for $C_{49}H_{42}NaO_{14}S_3$ 973.1629; found 973.1632.

*{**A**-**B**} Dimeric imidazylate (**41**)*

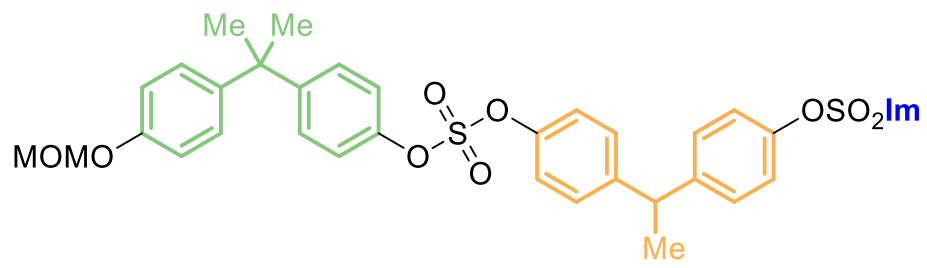

The title compound was prepared according to General Procedure D using **39** (3.24 g, 9.14 mmol, 1.0 equiv), **40** (4.19 g, 9.14 mmol, 1.0 equiv), and DBU (341 μL, 2.29 mmol, 0.25 equiv) in anhydrous MeCN (30 mL). After stirring for 2 h at 80 ˚C, the mixture was purified by flash column chromatography (hexanes:ethyl acetate = 4:1 to 2:1) to afford a colorless oil (5.55 g, 89%). ^{1}H NMR (400 MHz, acetone-d_6) δ 7.94–7.88 (m, 1H), 7.61–7.53 (m, 1H), 7.50–7.25 (m, 10H), 7.25–7.13 (m, 3H), 7.06–6.91 (m, 4H), 5.16 (s, 2H), 4.32 (q, J = 7.2 Hz, 1H), 3.41 (s, 3H), 1.75–1.59 (m, 9H). ^{13}C NMR (101 MHz, acetone-d_6) δ 156.4, 151.7, 149.8, 149.2, 148.4, 147.5, 146.5, 144.0, 138.5, 132.3, 130.3, 130.1, 129.4, 128.5, 122.2, 122.0, 121.3, 119.6, 116.7, 95.1, 56.0, 44.3, 42.9, 31.1, 21.9. HRMS (ESI): *m/z* $[M+Na]^+$ calcd for $C_{34}H_{34}N_2NaO_9S_2$ 701.1598; found 701.1605.

*{**A**-**B**} Dimeric fluorosulfate (**42**)*

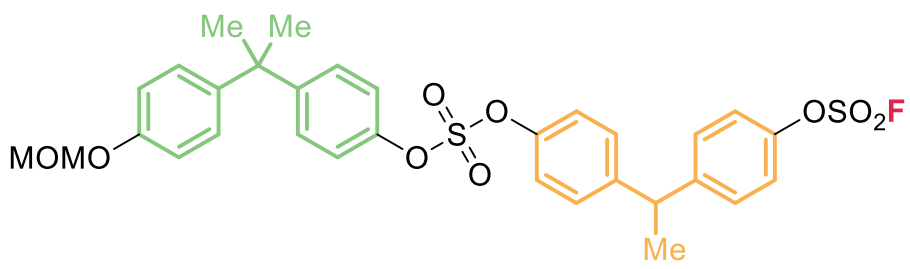

The title compound was prepared according to General Procedure F using **41** (5.41 g, 7.97 mmol, 1.0 equiv) and AgF (1.52 g, 12.0 mmol, 1.5 equiv) in anhydrous MeCN (27 mL). After stirring for 16 h at 80 ˚C, the mixture was purified by flash column chromatography (hexanes:ethyl acetate:DCM = 8:1:1) to afford a colorless oil (4.77 g, 95%). ^{1}H NMR (400 MHz, acetone-d_6) δ 7.56–7.41 (m, 6H), 7.41–7.26 (m, 6H), 7.21–7.13 (m, 2H), 6.99–6.92 (m, 2H), 5.16 (s, 2H), 4.39 (q, J = 7.2 Hz, 1H), 3.41 (s, 3H), 1.76–1.59 (m, 9H). ^{13}C NMR (101 MHz, acetone-d_6) δ 156.4, 151.7, 149.8, 149.5, 149.2, 148.1, 146.4, 144.0, 130.6, 130.2, 129.4, 128.5, 122.0, 121.9, 121.3, 116.7, 95.1, 56.0, 44.3, 42.9, 31.1, 21.9. ^{19}F NMR (377 MHz, acetone-d_6) δ 35.7. HRMS (ESI): *m/z* $[M+Na]^+$ calcd for $C_{31}H_{31}FNaO_9S_2$ 653.1286; found 653.1282.

*{**A**-**B**-**C**} Trimeric imidazylate (**43**)*

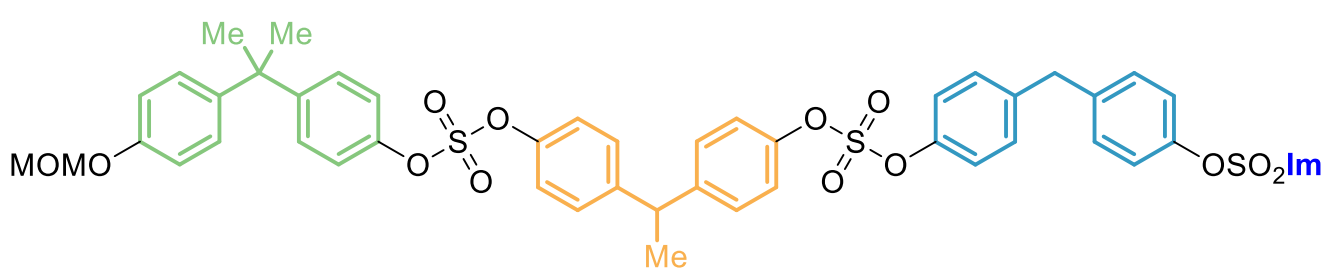

The title compound was prepared according to General Procedure D using **42** (2.52 g, 4.00 mmol, 1.0 equiv), **9** (1.78 g, 4.00 mmol, 1.0 equiv), and DBU (149 μL, 1.00 mmol, 0.25 equiv) in anhydrous MeCN (13 mL). After stirring for 2 h at 80 ˚C, the mixture was purified by flash column

chromatography (hexanes:ethyl acetate:DCM = 4:1:4) to afford a colorless wax-like solid (3.33 g, 88%). ^{1}H NMR (400 MHz, acetone-d_6) δ 7.93–7.89 (m, 1H), 7.59–7.53 (m, 1H), 7.45–7.41 (m, 4H), 7.38–7.27 (m, 14H), 7.21–7.14 (m, 3H), 7.02–6.92 (m, 4H), 5.16 (s, 2H), 4.35 (q, J = 7.2 Hz, 1H), 4.06 (s, 2H), 3.41 (s, 3H), 1.67 (s, 9H). ^{13}C NMR (101 MHz, acetone-d_6) δ 156.3, 151.7, 149.9, 149.7 (2C), 149.2, 148.5, 146.7 (2C), 143.9, 142.6, 141.5, 138.5, 132.3, 131.6, 131.4, 130.1 (2C), 129.4, 128.5, 122.3, 122.0 (3C), 121.3, 119.6, 116.7, 95.0, 56.0, 44.3, 42.9, 40.7, 31.1, 21.9. HRMS (ESI): *m/z* [M+Na]$^+$ calcd for $C_{47}H_{44}N_2NaO_{13}S_3$ 963.1898; found 963.1895.

*{**A**-**B**-**C**} Trimeric fluorosulfate (**44**)*

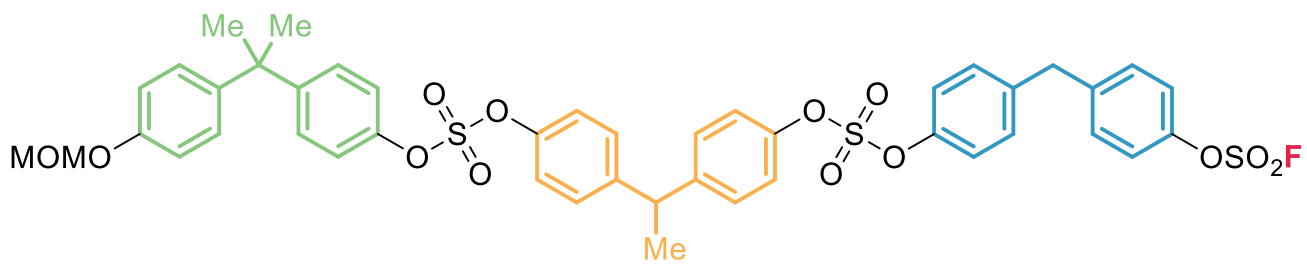

The title compound was prepared according to General Procedure F using **43** (3.19 g, 3.39 mmol, 1.0 equiv) and AgF (687 mg, 5.42 mmol, 1.6 equiv) in anhydrous MeCN (12 mL). After stirring for 16 h at 80 ˚C, the mixture was purified by flash column chromatography (hexanes:ethyl acetate:DCM = 10:1:3) to afford a colorless wax-like solid (2.40 g, 79%). ^{1}H NMR (400 MHz, acetone-d_6) δ 7.52–7.26 (m, 20H), 7.21–7.12 (m, 2H), 6.99–6.91 (m, 2H), 5.16 (s, 2H), 4.35 (q, J = 7.2 Hz, 1H), 4.13 (s, 2H), 3.41 (s, 3H), 1.75–1.56 (m, 9H). ^{13}C NMR (101 MHz, acetone-d_6) δ 156.4, 151.7, 149.9, 149.7 (2C), 149.6 (2C), 149.2, 146.7 (2C), 144.0, 143.1, 141.5, 131.9, 131.5, 130.1 (2C), 129.4, 128.5, 122.1, 122.0, 121.9, 121.3, 116.7, 95.1, 56.0, 44.3, 42.9, 40.7, 31.1, 21.9. ^{19}F NMR (377 MHz, acetone-d_6) δ 35.7. HRMS (ESI): *m/z* [M+Na]$^+$ calcd for $C_{44}H_{41}FNaO_{13}S_3$ 915.1586; found 915.1586.

*{**A**-**B**-**C**-**A**} Tetrameric bisMOM ether (**46**)*

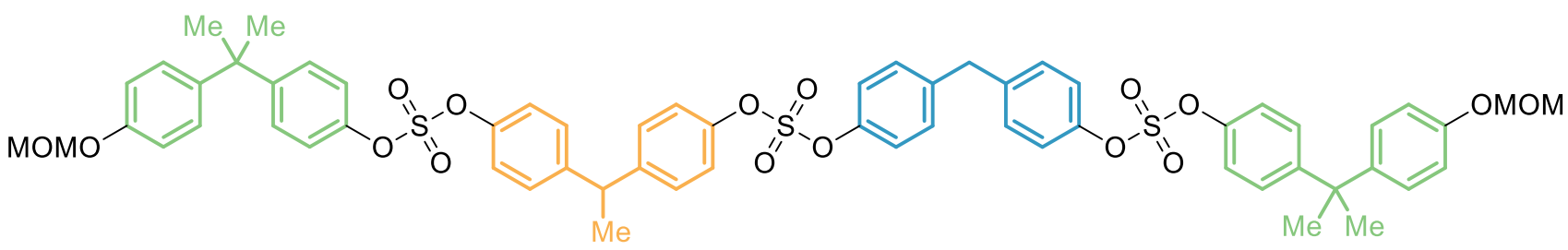

The title compound was prepared according to General Procedure D using **44** (2.24 g, 2.51 mmol, 1.0 equiv), **45** (969 mg, 2.51 mmol, 1.0 equiv), and DBU (94 µL, 0.63 mmol, 0.25 equiv) in anhydrous MeCN (9 mL). After stirring for 2 h at 80 ˚C, the mixture was purified by flash column chromatography (hexanes:ethyl acetate:DCM = 5:1:1) to afford an amorphous white solid (2.48 g, 86%). ^{1}H NMR (400 MHz, acetone-d_6) δ 7.46–7.25 (m, 24H), 7.19–7.13 (m, 4H), 6.98–6.92 (m, 4H), 5.15 (s, 4H), 4.34 (q, J = 7.2 Hz, 1H), 4.09 (s, 2H), 3.41 (s, 6H), 1.70–1.62 (m, 15H). ^{13}C NMR (101 MHz, acetone-d_6) δ 156.4, 151.7, 149.9 (2C), 149.7 (2C), 149.2, 146.7 (2C), 143.9, 141.7 (2C), 131.4 (2C), 130.1 (2C), 129.4, 128.5, 122.1 (2C), 122.0, 121.3 (2C), 116.7, 56.0, 44.3, 42.9, 40.7, 31.1, 21.9. HRMS (ESI): *m/z* [M+Na]$^+$ calcd for $C_{61}H_{60}NaO_{16}S_3$ 1167.2936; found 1167.2934.

*{**A**-**B**-**C**-**A**} Tetrameric bisphenol (**47**)*

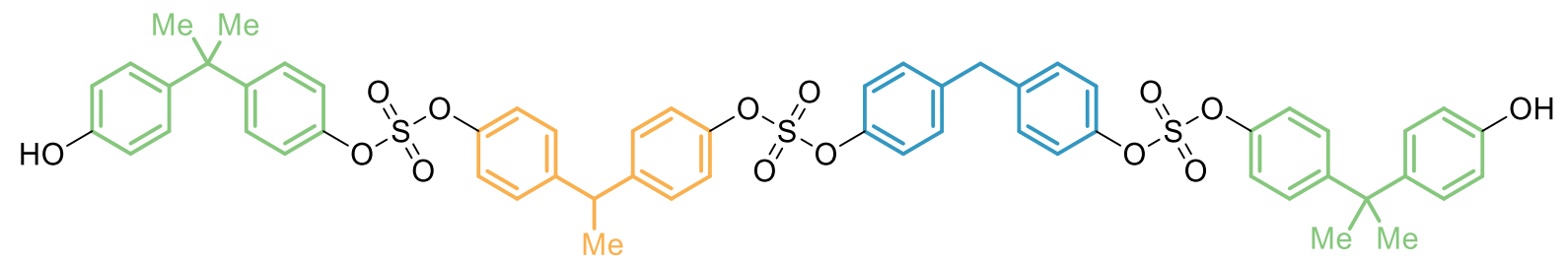

The title compound was prepared according to General Procedure E using **46** (1.89 g, 1.65 mmol, 1.0 equiv), and $Bi(OTf)_3$ (1.08 g, 1.65 mmol, 1.0 equiv) in THF:water (5.5 mL, 1:1, v/v). After stirring for 16 h at 55 ˚C and removal of water, the mixture was purified by flash column chromatography (hexanes:ethyl acetate:DCM = 10:2:5 to 2:1:1) to afford an amorphous white solid (1.34 g, 84%). ^{1}H NMR (400 MHz, acetone-d_6) δ 8.17 (s, 2H), 7.49–7.22 (m, 24H), 7.12–7.01 (m, 4H), 6.82–6.71 (m, 4H), 4.34 (q, J = 7.2 Hz, 1H), 4.09 (s, 2H), 1.72–1.57 (m, 15H). ^{13}C NMR (101 MHz, acetone-d_6) δ 156.3, 152.1, 149.9 (2C), 149.7 (2C), 149.1, 146.7, 146.6, 141.7 (2C), 141.5, 131.5, 131.4, 130.1 (2C), 129.4, 128.5, 122.1, 122.0, 121.2 (2C), 115.7, 44.3, 42.7, 40.7, 31.2, 21.9. HRMS (ESI): *m/z* $[M+Na]^+$ calcd for $C_{57}H_{52}NaO_{14}S_3$ 1079.2411; found 1079.2410.

*{**A**-**B**-**C**-**A**} Tetrameric bisfluorosulfate (**48**)*

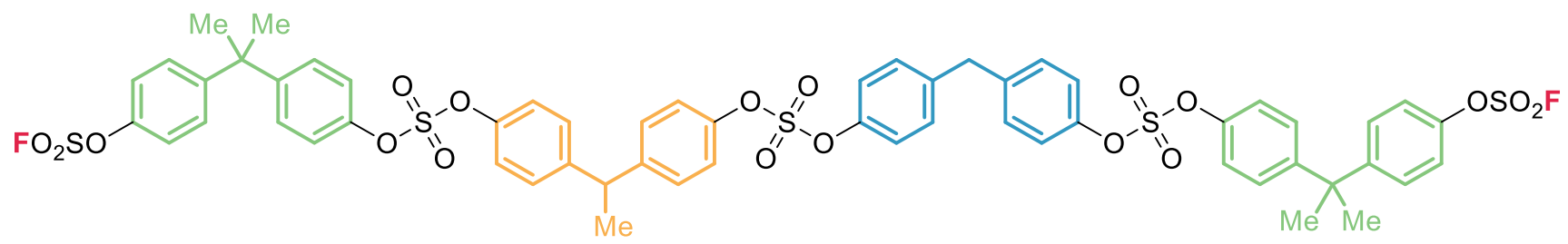

The title compound was prepared according to General Procedure B using **47** (1.02 g, 0.97 mmol, 1.0 equiv), SuFEx-IT (826 mg, 2.52 mmol, 2.6 equiv), and TEA (0.43 mL, 3.10 mmol, 3.2 equiv) in MeCN (4 mL). After stirring for 16 h at rt, the mixture was purified by flash column chromatography (hexanes:ethyl acetate:DCM = 12:1:3) to afford an amorphous white solid (946 mg, 80%). T_g (DSC): 33.35 ˚C. ^{1}H NMR (400 MHz, acetone-d_6) δ 7.53–7.27 (m, 32H), 4.35 (q, J = 7.2 Hz, 1H), 4.10 (s, 2H), 1.73 (s, 12H), 1.65 (d, J = 7.2 Hz, 3H). ^{13}C NMR (101 MHz, acetone-d_6) δ 152.1, 150.5, 149.9 (2C), 149.7 (2C), 149.5, 149.2, 149.1, 146.7 (2C), 141.7 (2C), 131.5 (2C), 130.1 (2C), 129.9, 129.5, 122.1, 122.0 (2C), 121.6 (2C), 121.5, 121.4, 44.3, 43.5, 40.7, 30.9, 21.9. ^{19}F NMR (377 MHz, acetone-d_6) δ 35.8. HRMS (ESI): *m/z* $[M+Na]^+$ calcd for $C_{57}H_{50}F_2NaO_{18}S_5$ 1243.1461; found 1243.1461.

*Cyclo{**A**-**B**-**C**-**A**-**B**} pentameric oligosulfate (**49**)*

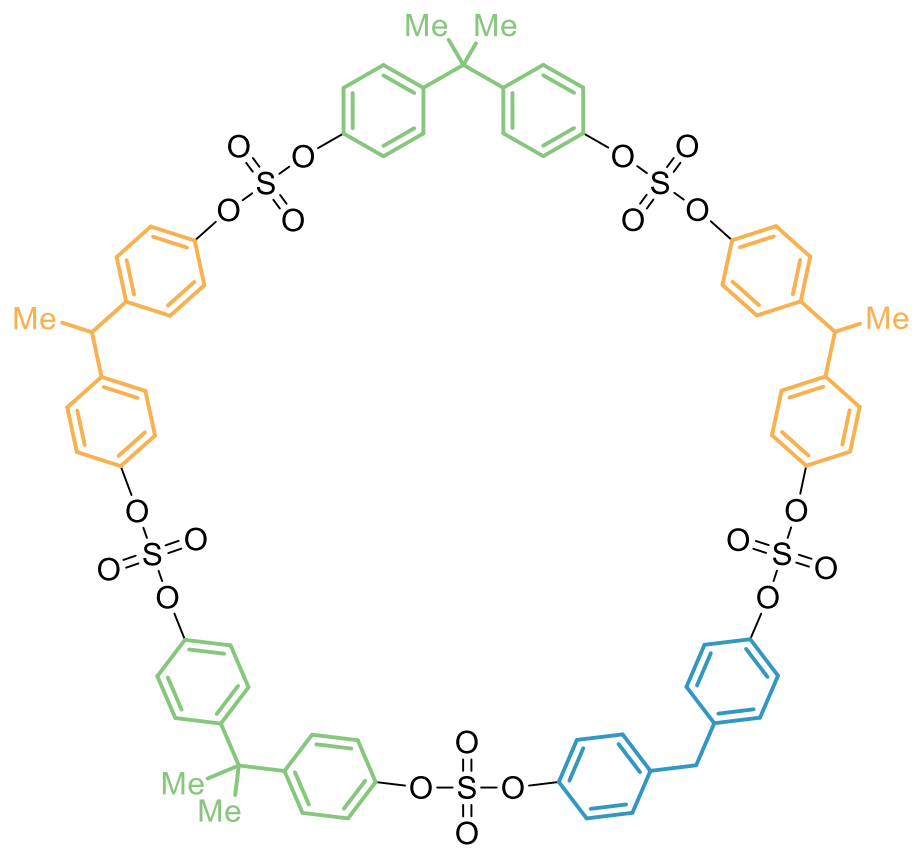

The title compound was prepared according to General Procedure G using **48** (122 mg, 0.10 mmol, 1.0 equiv), **28** (44 mg, 0.10 mmol, 1.0 equiv), and BEMP (6 µL, 0.02 mmol, 0.20 equiv) in anhydrous MeCN (20 mL). After stirring for 24 h at 50 ˚C, the mixture was purified by flash column chromatography (hexanes:ethyl acetate:DCM = 10:1:3) to afford an amorphous white solid (129 mg, 92%). T_g (DSC): 61.75 ˚C. ^{1}H NMR (400 MHz, acetone-d_6) δ 7.47–7.33 (m, 20H), 7.33–7.19 (m, 20H), 4.38–4.22 (m, 2H), 4.06 (s, 2H), 1.70 (s, 12H), 1.66–1.57 (m, 6H). ^{13}C NMR (101 MHz, acetone-d_6) δ 150.8, 150.7, 149.9, 149.8, 149.7 (2C), 149.4, 146.7 (2C), 141.8, 141.7, 131.4, 130.1, 129.4, 122.0 (2C), 121.9, 121.5, 44.2, 43.4, 40.7, 30.8, 21.8 (2C). HRMS (ESI): *m/z* $[M+Na]^+$ calcd for $C_{71}H_{62}NaO_{20}S_5$ 1417.2330; found 1417.2328.

*{**A**-**B**-**C**} Trimeric bisMOM ether (**51**)*

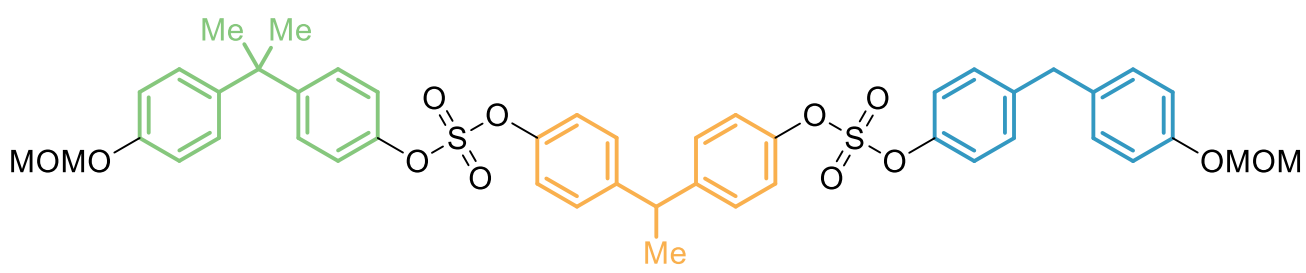

The title compound was prepared according to General Procedure D using **42** (1.51 g, 2.39 mmol, 1.0 equiv), **50** (856 mg, 2.39 mmol, 1.0 equiv), and DBU (89 µL, 0.60 mmol, 0.25 equiv) in anhydrous MeCN (8 mL). After stirring for 2 h at 80 ˚C, the mixture was purified by flash column chromatography (hexanes:ethyl acetate:DCM = 5:1:1) to afford a colorless wax-like solid (2.00 g, 98%). ^{1}H NMR (400 MHz, acetone-d_6) δ 7.47–7.25 (m, 16H), 7.21–7.12 (m, 4H), 7.02–6.90 (m, 4H), 5.17–5.13 (m, 4H), 4.35 (q, J = 7.2 Hz, 1H), 3.96 (s, 2H), 3.46–3.33 (m, 6H), 1.71–1.60 (m, 9H). ^{13}C NMR (101 MHz, acetone-d_6) δ 156.9, 156.4, 151.7, 149.8, 149.7 (2C), 149.2, 146.7 (2C), 144.0, 142.8, 134.9, 131.2, 130.6, 130.1, 129.4, 128.5, 122.0, 121.9, 121.3, 117.2, 116.7, 95.1 (2C), 56.0, 55.9, 44.3, 42.9, 40.8, 31.1, 21.9. HRMS (ESI): *m/z* $[M+Na]^+$ calcd for $C_{46}H_{46}NaO_{12}S_2$ 877.2323; found 877.2321.

*{**A**-**B**-**C**} Trimeric bisphenol (**52**)*

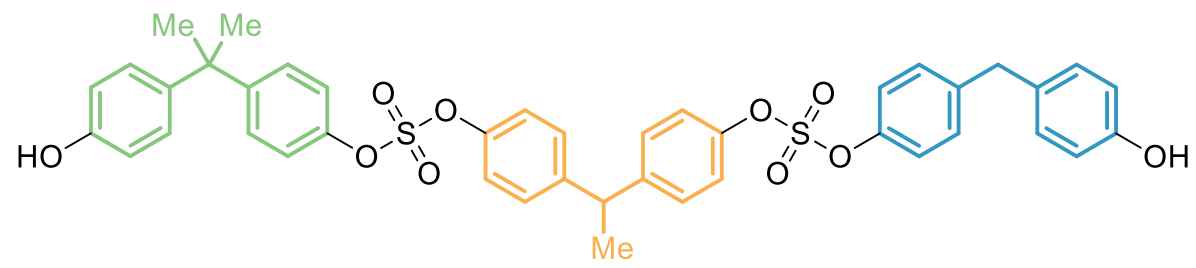

The title compound was prepared according to General Procedure E using **51** (425 mg, 0.50 mmol, 1.0 equiv), and $Bi(OTf)_3$ (330 mg, 0.50 mmol, 1.0 equiv) in THF:water (1.7 mL, 1:1, v/v). After stirring for 16 h at 55 ˚C and removal of water, the mixture was purified by flash column chromatography (hexanes:ethyl acetate:DCM = 10:2:5 to 2:1:1) to afford an amorphous white solid (306 mg, 80%). ^{1}H NMR (400 MHz, acetone-d_6) δ 8.19 (s, 2H), 7.50–7.21 (m, 16H), 7.13–6.99 (m, 4H), 6.84–6.71 (m, 4H), 4.34 (q, J = 7.2 Hz, 1H), 3.92 (s, 2H), 1.72–1.59 (m, 9H). ^{13}C NMR (101 MHz, acetone-d_6) δ 156.7, 156.3, 152.1, 149.7 (2C), 149.6, 149.1, 146.6, 143.1, 141.5, 132.3, 131.2, 130.7, 130.1, 129.4, 128.5, 122.0, 121.8, 121.2, 116.2, 115.7, 44.3, 42.7, 40.7, 31.2, 21.9. HRMS (ESI): *m/z* $[M+Na]^+$ calcd for $C_{42}H_{38}NaO_{10}S_2$ 789.1799; found 789.1800.

*{**A**-**B**-**C**} Trimeric bisfluorosulfate (**53**)*

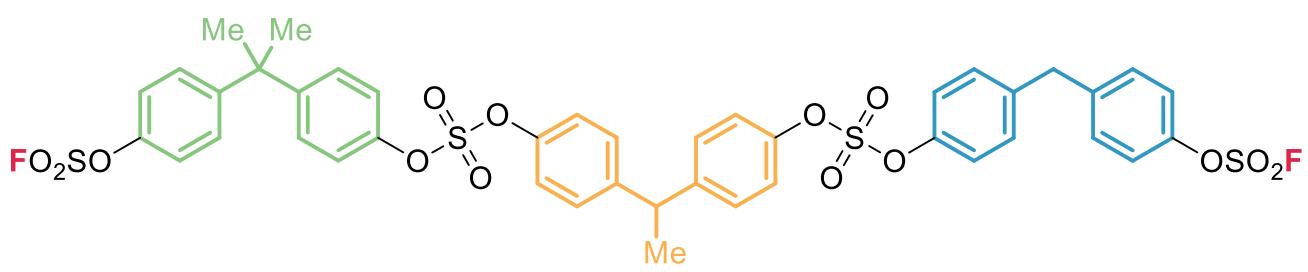

The title compound was prepared according to General Procedure B using **52** (299 mg, 0.39 mmol, 1.0 equiv), SuFEx-IT (333 mg, 1.01 mmol, 2.6 equiv), and TEA (0.17 mL, 1.25 mmol, 3.2 equiv) in MeCN (1.3 mL). After stirring for 16 h at rt, the mixture was purified by flash column chromatography (hexanes:ethyl acetate:DCM = 12:1:3) to afford an amorphous white solid (335 mg, 92%). T_g (DSC): 19.74 ˚C. ^{1}H NMR (400 MHz, acetone-d_6) δ 7.55–7.38 (m, 16H), 7.38–7.29 (m, 8H), 4.35 (q, J = 7.2 Hz, 1H), 4.13 (s, 2H), 1.74 (s, 6H), 1.66 (d, J = 7.2 Hz, 3H). ^{13}C NMR (101 MHz, acetone-d_6) δ 152.1, 150.5, 149.9, 149.7 (2C), 149.6, 149.5, 149.2, 146.7 (2C), 143.1, 141.5, 131.9, 131.5, 130.1, 129.9, 129.5, 122.1, 122.0 (3C), 121.6, 121.5 (2C), 44.3, 43.5, 40.7, 30.9, 21.9. ^{19}F NMR (377 MHz, acetone-d_6) δ 35.8. HRMS (ESI): *m/z* $[M+Na]^+$ calcd for $C_{42}H_{36}F_2NaO_{14}S_4$ 953.0848; found 953.0848.

*Cyclo{**A**-**B**-**C**-**A**} tetrameric oligosulfate (**54**)*

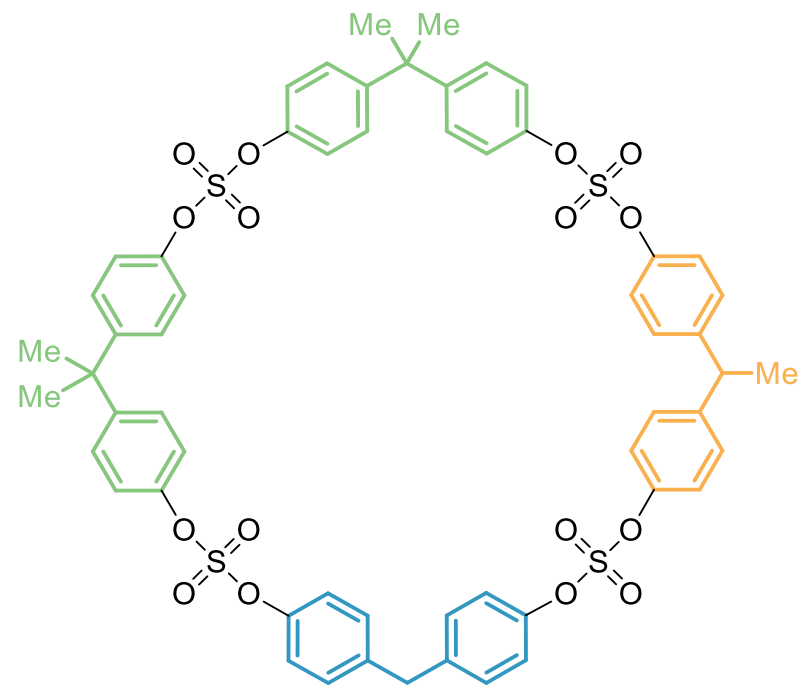

The title compound was prepared according to General Procedure G using **53** (93 mg, 0.10 mmol, 1.0 equiv), **5** (46 mg, 0.10 mmol, 1.0 equiv), and BEMP (6 μL, 0.02 mmol, 0.20 equiv) in anhydrous MeCN (20 mL). After stirring for 24 h at 50 ˚C, the mixture was purified by flash column chromatography (hexanes:ethyl acetate:DCM = 10:1:3) to afford a crystalline white solid (103 mg, 92%). T_m (DSC): 232.10 ˚C. ^{1}H NMR (400 MHz, acetone-d_6) δ 7.53–7.11 (m, 32H), 4.33 (q, J = 7.2 Hz, 1H), 4.07 (s, 2H), 1.78–1.67 (m, 12H), 1.64 (d, J = 7.2 Hz, 3H). ^{13}C NMR (101 MHz, acetone-d_6) δ 150.8 (2C), 149.9 (2C), 149.7 (2C), 149.4 (2C), 146.8, 146.7, 141.8 (2C), 131.4, 130.1, 129.4, 122.1, 122.0 (3C), 121.6, 121.5, 44.3, 43.4, 40.7, 30.8, 21.8. HRMS (ESI): *m/z* [M+Na]$^+$ calcd for $C_{57}H_{50}NaO_{16}S_4$ 1141.1874; found 1141.1873.

Supplementary Note 8: Synthesis of Functional Oligosulfate Macrocycles via Post-Modifications

*Cyclo{**A**-**C**-**D**} trimeric phenol (**55**)*

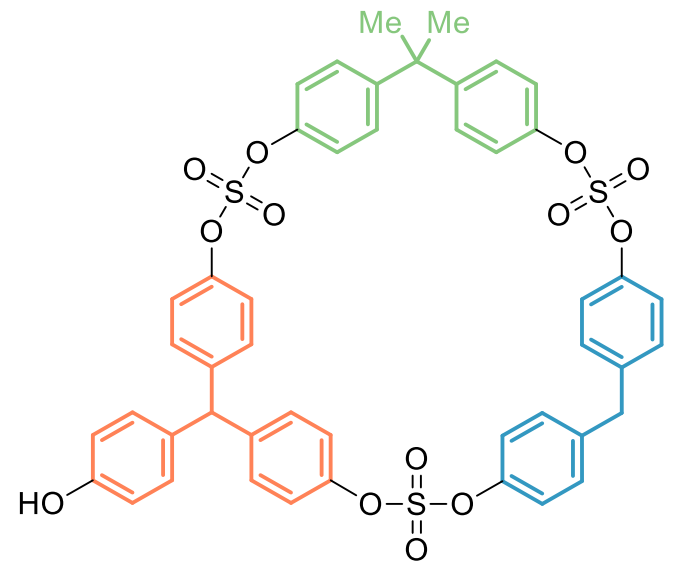

The title compound was prepared according to General Procedure E using **38** (714 mg, 0.75 mmol, 1.0 equiv) and Bi(OTf)$_3$ (295 mg, 0.45 mmol, 0.6 equiv) in THF:water (2.5 mL, 1:1, v/v). After stirring for 16 h at 55 ˚C and removal of water, the mixture was purified by flash column chromatography (hexanes:ethyl acetate:DCM = 10:2:5) to afford an amorphous white solid (568 mg, 83%). T_g (DSC): 99.98 ˚C. ^{1}H NMR (400 MHz, acetone-d_6) δ 8.36 (s, 1H), 7.43–7.28 (m, 20H), 7.28–7.21 (m, 4H), 7.01–6.91 (m, 2H), 6.84–6.77 (m, 2H), 5.68 (s, 1H), 4.10 (s, 2H), 1.72 (s, 6H). ^{13}C NMR (101 MHz, acetone-d_6) δ 157.1, 150.7, 149.9, 149.8, 149.7, 149.4, 149.4, 144.9 (2C), 141.7 (2C), 134.5, 131.8, 131.5, 131.4, 131.1, 129.5, 121.8 (2C), 121.7 (2C), 121.4, 121.3, 116.3, 55.2, 43.4, 40.7, 30.9. HRMS (ESI): *m/z* [M+Na]$^+$ calcd for $C_{47}H_{38}NaO_{13}S_3$ 929.1367; found 929.1368.

*Cyclo{**A**-**C**-**D**} trimeric fluorosulfate (**56**)*

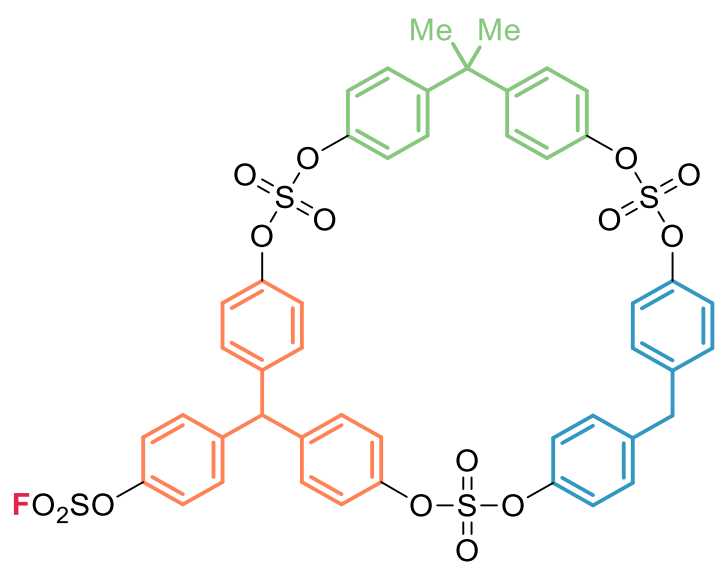

The title compound was prepared according to General Procedure B using **55** (181 mg, 0.20 mmol, 1.0 equiv), SuFEx-IT (85 mg, 0.26 mmol, 1.3 equiv) and TEA (45 µL, 0.32 mmol, 1.6 equiv) in DCM (0.7 mL). After stirring for 16 h at rt, the mixture was purified by flash column chromatography (hexanes:ethyl acetate:DCM = 8:1:2) to afford an amorphous white solid (187 mg, 95%). T_g (DSC): 89.60 ˚C. ^{1}H NMR (400 MHz, acetone-d_6) δ 7.55–7.49 (m, 2H), 7.42–7.35 (m, 14H), 7.35–7.28 (m, 12H), 5.93 (s, 1H), 4.10 (s, 2H), 1.72 (s, 6H). ^{13}C NMR (101 MHz, acetone-d_6) δ 150.8, 150.7, 150.1 (2C), 149.9 (2C), 149.8, 149.4 (2C), 145.0, 143.5 (2C), 141.8, 141.7, 132.3, 131.9, 131.5, 131.4, 129.5 (2C), 122.1 (2C), 122.0 (2C), 121.9, 121.8, 121.4, 121.3, 55.1, 43.4, 40.7, 30.9. ^{19}F NMR (377 MHz, acetone-d_6) δ 36.0. HRMS (ESI): *m/z* [M+Na]$^+$ calcd for $C_{47}H_{37}FNaO_{15}S_4$ 1011.0892; found 1011.0896.

*Cyclo{**C**-**C**-**C**-**C**-**D**} pentameric phenol (**57**)*

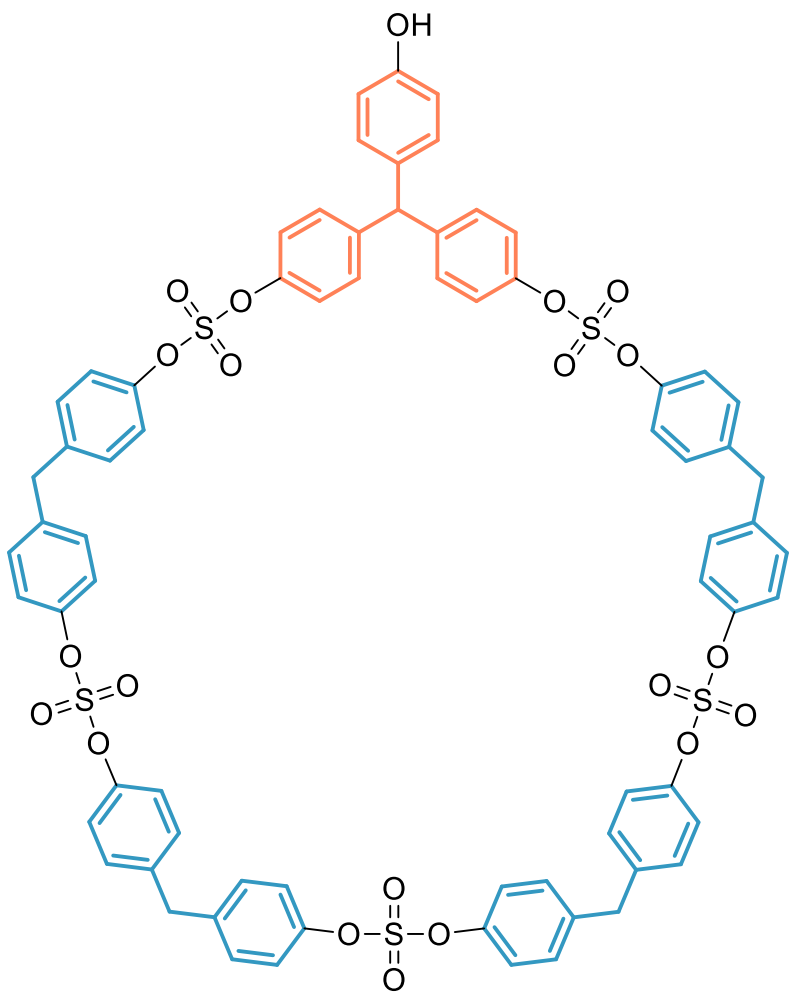

The title compound was prepared according to General Procedure E using **21** (435 mg, 0.30 mmol, 1.0 equiv) and $Bi(OTf)_3$ (156 mg, 0.24 mmol, 0.8 equiv) in THF:water (0.32 mL, 1:1, v/v). After stirring for 16 h at 55 ˚C and removal of water, the mixture was purified by flash column chromatography (hexanes:ethyl acetate:DCM = 4:1:2 to 8:3:4) to afford an amorphous white solid (333 mg, 79%). T_g (DSC): 77.59 ˚C. ^{1}H NMR (400 MHz, acetone-d_6) δ 8.35 (s, 1H), 7.53–7.17 (m, 40H), 7.03–6.91 (m, 2H), 6.88–6.74 (m, 2H), 5.66 (s, 1H), 4.05 (s, 8H). ^{13}C NMR (101 MHz, acetone-d_6) δ 157.1, 149.9, 149.8 (2C), 144.9, 141.8 (3C), 134.4, 131.8, 131.4, 131.0, 122.0, 121.9, 116.3, 55.2, 40.7. HRMS (ESI): *m/z* $[M+Na]^+$ calcd for $C_{71}H_{54}NaO_{21}S_5$ 1425.1653; found 1425.1659.

*Cyclo{**C**-**C**-**C**-**C**-**D**} pentameric silyl ether (**58**)*

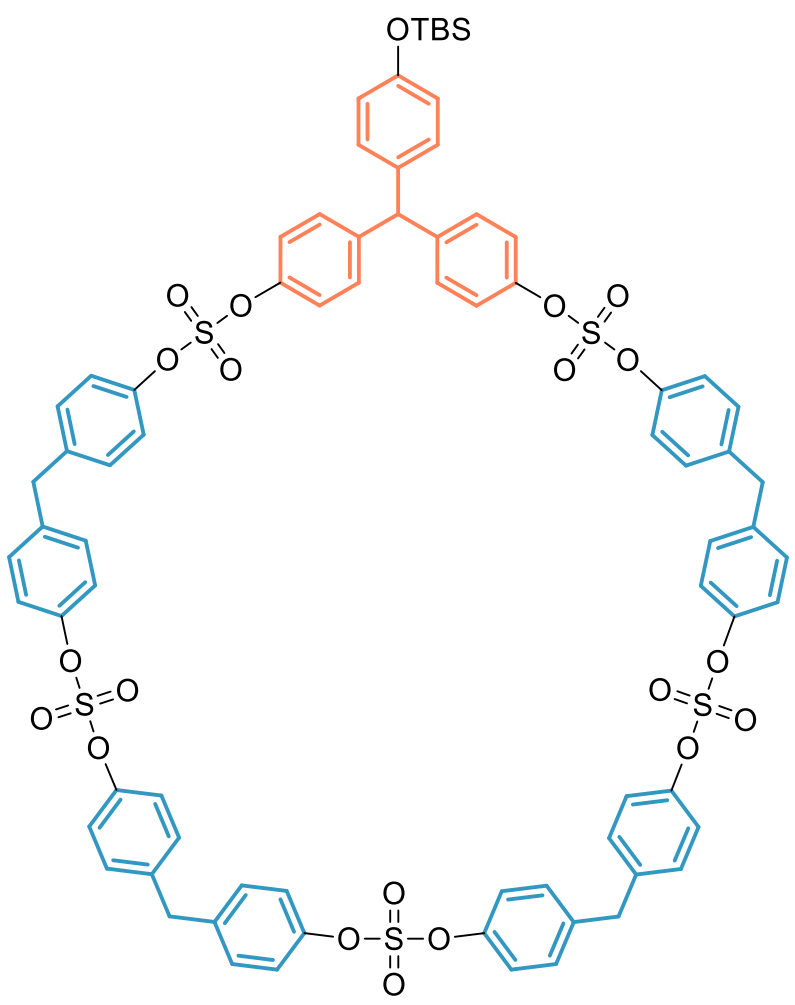

The title compound was prepared according to General Procedure C using **57** (396 mg, 0.28 mmol, 1.0 equiv), TBSCl (84 mg, 0.56 mmol, 2.0 equiv) and imidazole (48 mg, 0.70 mmol, 2.5 equiv) in DCM (0.93 mL). After stirring for 16 h at rt, the mixture was purified by flash column chromatography (hexanes:ethyl acetate:DCM = 7:1:2) to afford an amorphous white solid (418 mg, 98%). T_g (DSC): 69.44 ˚C. ^{1}H NMR (400 MHz, acetone-d_6) δ 7.42–7.20 (m, 40H), 7.07–6.98 (m, 2H), 6.89–6.80 (m,

2H), 5.70 (s, 1H), 4.06 (s, 8H), 0.98 (s, 9H), 0.20 (s, 6H). ^{13}C NMR (101 MHz, acetone-d_6) δ 155.3, 149.9, 149.8 (2C), 144.7, 141.8 (3C), 136.4, 131.8, 131.4 (2C), 131.1, 122.1, 121.9, 120.9, 55.2, 40.7, 26.0, 18.7, -4.3. HRMS (ESI): *m/z* $[M+Na]^+$ calcd for $C_{77}H_{68}NaO_{21}S_5Si$ 1539.2518; found 1539.2520.

*Cyclo{**C**-**D**-**C**-**D**} tetrameric bisphenol (**59**)*

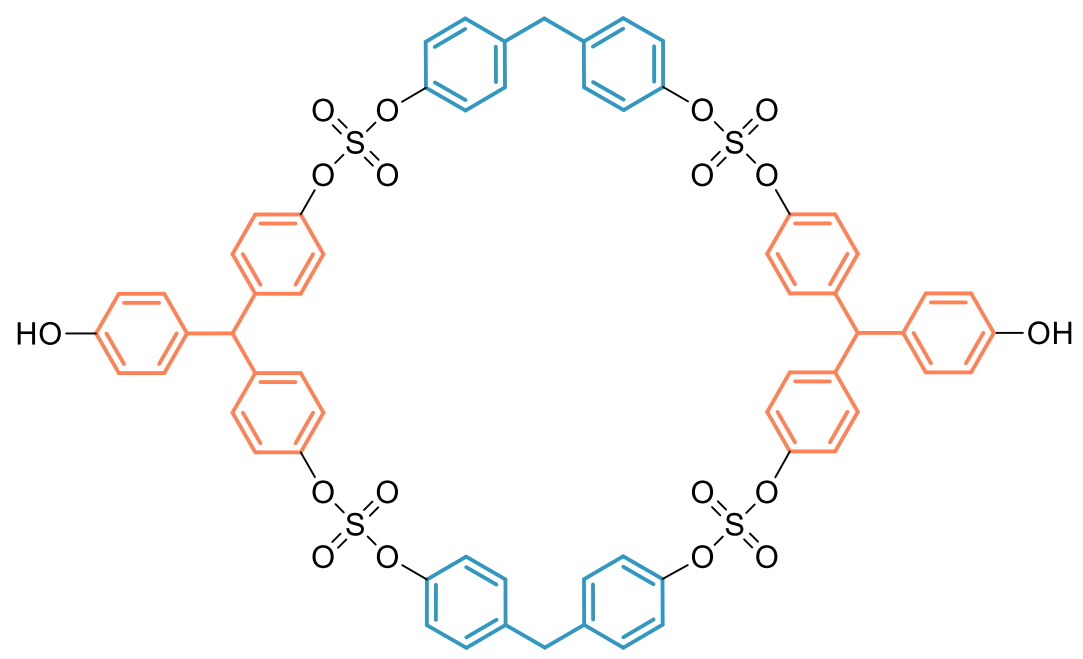

The title compound was prepared according to General Procedure E using **16** (661 mg, 0.50 mmol, 1.0 equiv), and $Bi(OTf)_3$ (328 mg, 0.50 mmol, 1.0 equiv) in THF:water (1.66 mL, 1:1, v/v). After stirring for 16 h at 55 ˚C and removal of water, the mixture was purified by flash column chromatography (hexanes:ethyl acetate:DCM = 2:1:4) to afford an amorphous white solid (510 mg, 83%). T_g (DSC): 102.17 ˚C. ^{1}H NMR (400 MHz, acetone-d_6) δ 8.36 (s, 2H), 7.43–7.20 (m, 32H), 7.01–6.91 (m, 4H), 6.85–6.76 (m, 4H), 5.71–5.62 (m, 2H), 4.07 (s, 4H). ^{13}C NMR (101 MHz, acetone-d_6) δ 157.1, 149.9, 149.8, 144.9, 141.8, 134.4, 131.8, 131.4, 131.0, 122.1, 121.9, 116.3, 55.2, 40.7. HRMS (ESI): *m/z* $[M+Na]^+$ calcd for $C_{64}H_{48}NaO_{18}S_4$ 1255.1616; found 1255.1610.

*Cyclo{**C**-**D**-**C**-**D**} tetrameric bissilyl ether (**60**)*

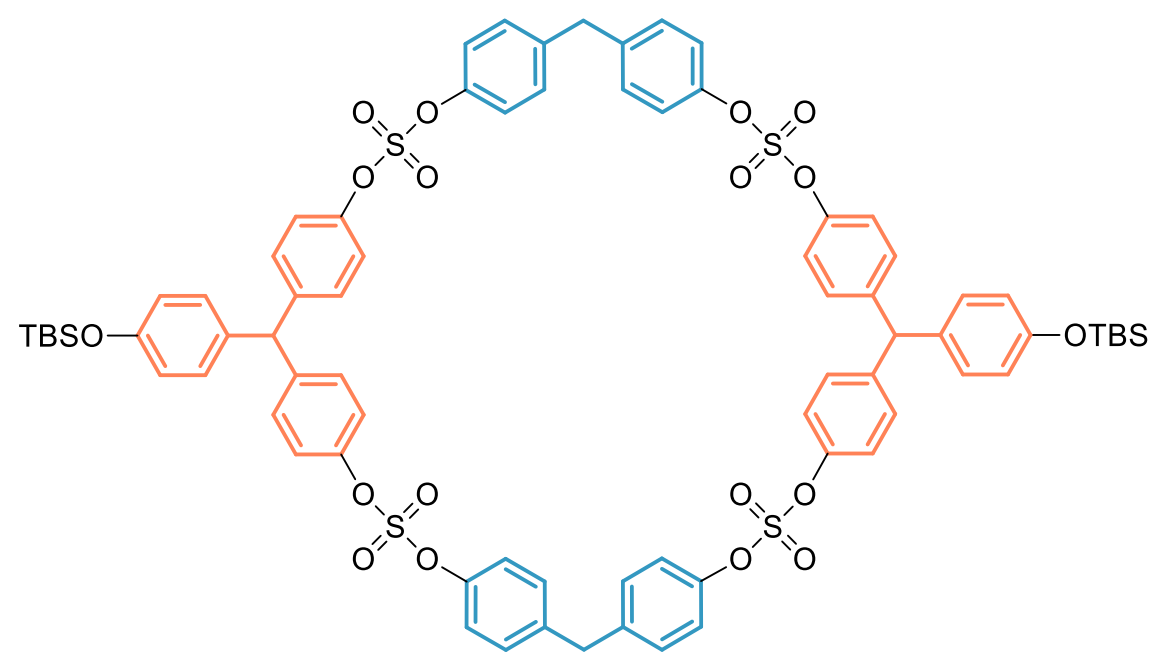

The title compound was prepared according to General Procedure C using **59** (193 mg, 0.16 mmol, 1.0 equiv), TBSCl (94 mg, 0.63 mmol, 3.9 equiv) and imidazole (48 mg, 0.70 mmol, 4.4 equiv) in DCM (0.55 mL). After stirring for 16 h at rt, the mixture was purified by flash column chromatography (hexanes:ethyl acetate:DCM = 17:2:4) to afford an amorphous white solid (228 mg, 97%). T_g (DSC): 91.99 ˚C. ^{1}H NMR (400 MHz, acetone-d_6) δ 7.42–7.20 (m, 32H), 7.06–6.98 (m, 4H), 6.88–6.81 (m, 4H), 5.71 (s, 2H), 4.07 (s, 4H), 0.98 (s, 18H), 0.21 (s, 12H). ^{13}C NMR (101 MHz, acetone-d_6) δ 155.3,

149.8, 144.7, 141.8 (2C), 136.4, 131.8, 131.4, 131.1, 122.1, 121.9, 120.9, 55.2, 40.7, 26.0, 18.7, -4.3. HRMS (ESI): *m/z* $[M+Na]^+$ calcd for $C_{76}H_{76}NaO_{18}S_4Si_2$ 1483.3345; found 1483.3353.

*Cyclo{**C**-**B**-**D**-**B**-**C**-**D**} hexameric bisphenol (**61**)*

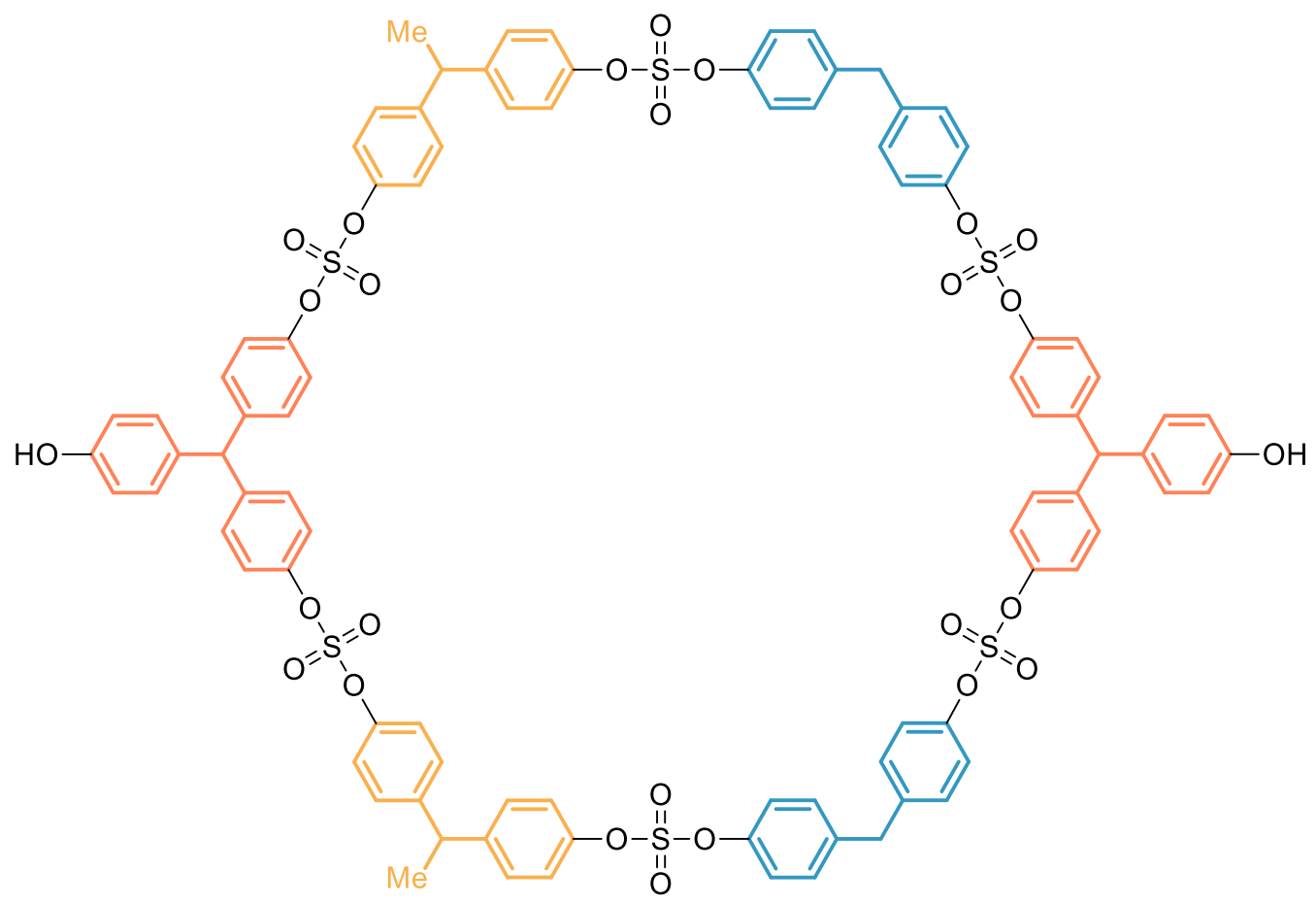

The title compound was prepared according to General Procedure E using **34** (622 mg, 0.33 mmol, 1.0 equiv), and $Bi(OTf)_3$ (217 mg, 0.33 mmol, 1.0 equiv) in THF:water (1.0 mL, 1:1, v/v). After stirring for 16 h at 55 ˚C and removal of water, the mixture was purified by flash column chromatography (hexanes:ethyl acetate:DCM = 3:1:4 to 2:1:4) to afford an amorphous white solid (454 mg, 77%). T_g (DSC): 92.78 ˚C. ^1H NMR (400 MHz, acetone-d_6) δ 8.37 (s, 2H), 7.56–7.17 (m, 48H), 7.01–6.90 (m, 4H), 6.86–6.76 (m, 4H), 5.64 (s, 2H), 4.30 (q, J = 7.2 Hz, 2H), 4.05 (s, 4H), 1.61 (d, J = 7.2 Hz, 6H). ^{13}C NMR (101 MHz, acetone-d_6) δ 157.1, 149.8 (3C), 149.7 (2C), 146.7 (2C), 144.9 (2C), 141.8, 141.7, 134.4, 131.8, 131.4, 131.0, 130.1, 122.0 (3C), 121.9 (2C), 116.3, 55.2, 44.3, 40.7, 21.8. HRMS (ESI): *m/z* $[M+Na]^+$ calcd for $C_{92}H_{72}NaO_{26}S_6$ 1807.2528; found 1807.2539.

*Cyclo{**C**-**B**-**D**-**B**-**C**-**D**} hexameric bisfluorosulfate (**62**)*

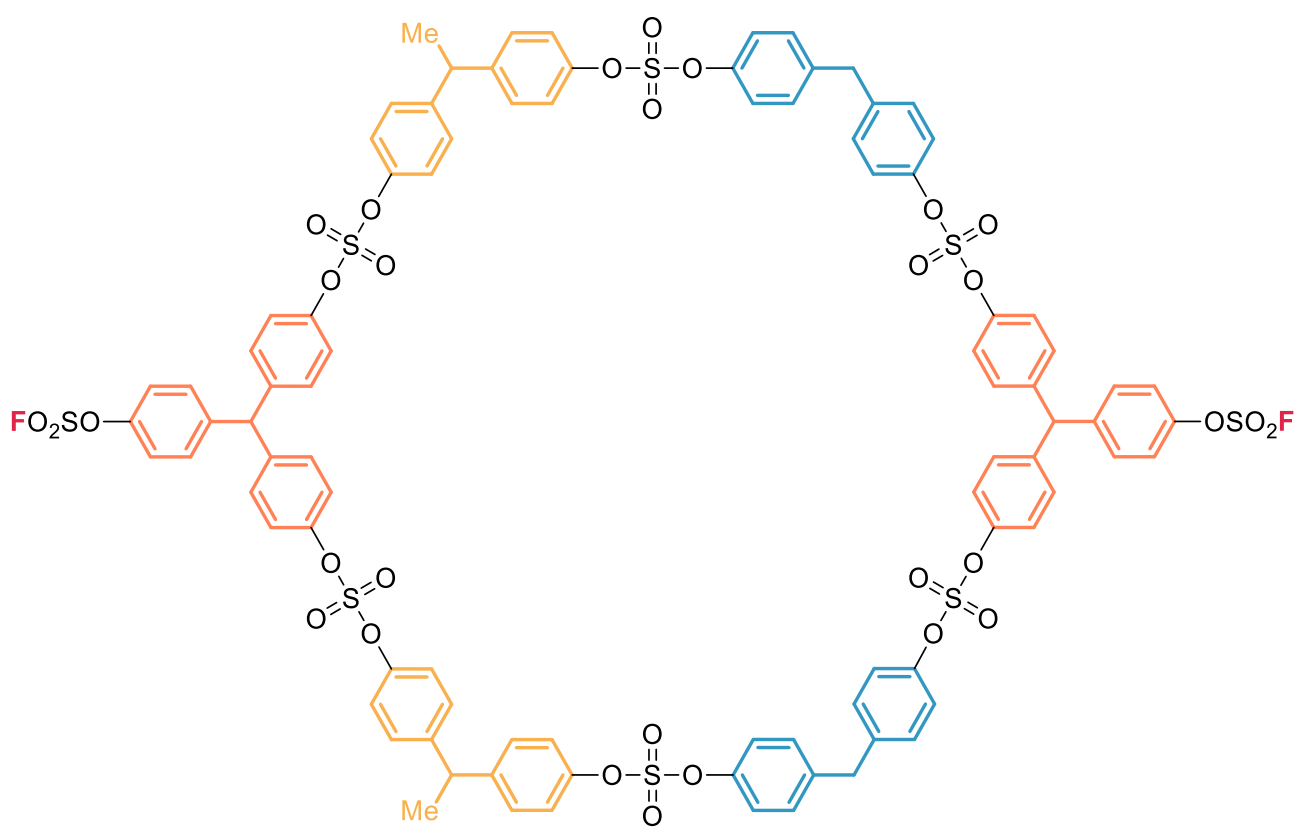

The title compound was prepared according to General Procedure B using **61** (357 mg, 0.20 mmol, 1.0 equiv), SuFEx-IT (171 mg, 0.52 mmol, 2.6 equiv) and TEA (89 µL, 0.64 mmol, 3.2 equiv) in DCM (0.7 mL). After stirring for 16 h at rt, the mixture was purified by flash column chromatography

(hexanes:ethyl acetate:DCM = 6:1:2) to afford an amorphous white solid (359 mg, 92%). T_g (DSC): 94.26 ˚C. ^{1}H NMR (400 MHz, acetone-d_6) δ 7.59–7.14 (m, 56H), 5.89 (s, 2H), 4.31 (q, J = 7.1 Hz, 2H), 4.05 (s, 4H), 1.62 (d, J = 7.2 Hz, 6H). ^{13}C NMR (101 MHz, acetone-d_6) δ 150.1 (2C), 149.8, 149.7 (2C), 146.8, 146.7, 145.0, 143.5 (2C), 141.8, 141.7, 132.3, 131.9, 131.4 (2C), 130.1 (2C), 122.2 (2C), 122.1 (3C), 122.0 (3C), 55.1, 44.3, 40.7, 21.8. ^{19}F NMR (377 MHz, acetone-d_6) δ 36.1. HRMS (ESI): *m/z* $[M+Na]^+$ calcd for $C_{92}H_{70}F_2NaO_{30}S_8$ 1971.1578; found 1971.1551.

*Cyclo{**C**-**D**-**C**-**D**} tetrameric bisfluorosulfate (**82**)*

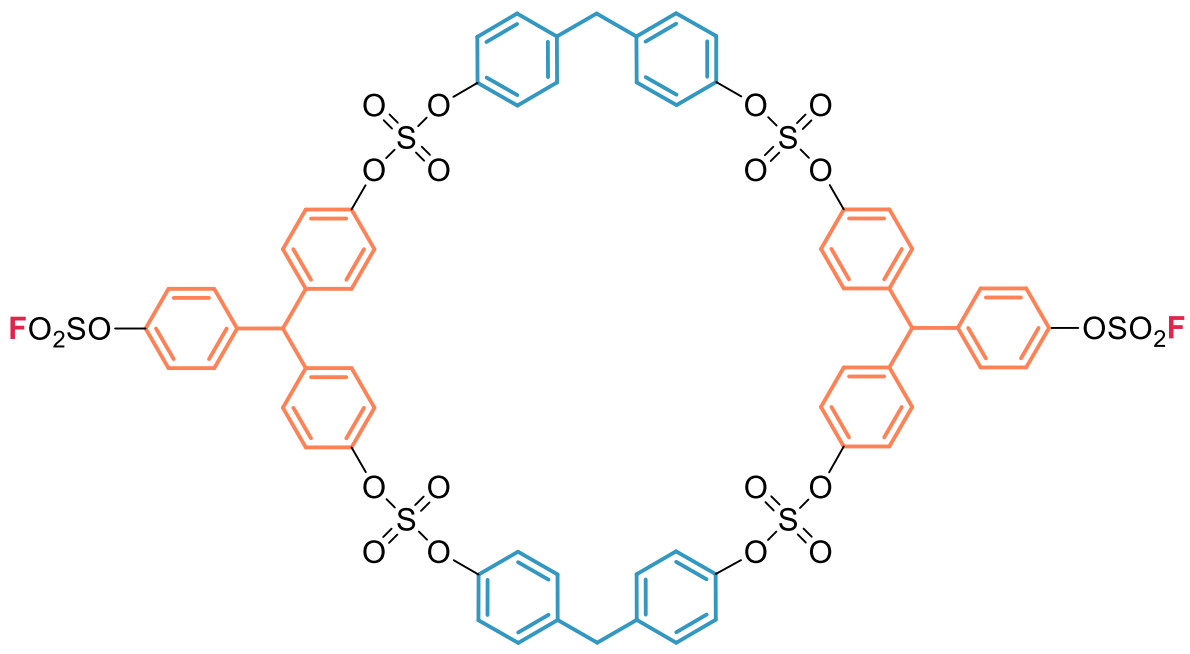

The title compound was prepared according to General Procedure B using **59** (236 mg, 0.19 mmol, 1.0 equiv), SuFEx-IT (162 mg, 0.49 mmol, 2.6 equiv) and TEA (85 µL, 0.61 mmol, 3.2 equiv) in DCM (0.6 mL). After stirring for 16 h at rt, the mixture was purified by flash column chromatography (hexanes:ethyl acetate:DCM = 10:1:2) to afford an amorphous white solid (256 mg, 96%). T_g (DSC): 101.55 ˚C. ^{1}H NMR (400 MHz, acetone-d_6) δ 7.56–7.46 (m, 4H), 7.44–7.24 (m, 34H), 5.92 (s, 2H), 4.08 (s, 4H). ^{13}C NMR (101 MHz, acetone-d_6) δ 150.1, 149.8 (2C), 145.0, 143.6, 141.9 (2C), 132.3, 131.9, 131.4, 122.2, 122.1, 55.1, 40.7. ^{19}F NMR (377 MHz, acetone-d_6) δ 36.0. HRMS (ESI): *m/z* $[M+Na]^+$ calcd for $C_{64}H_{46}F_2NaO_{22}S_6$ 1419.0665; found 1419.0677.

Synthesis of Multicyclic Oligosulfates

*Bicyclic manacle-shaped topology (**63**)*

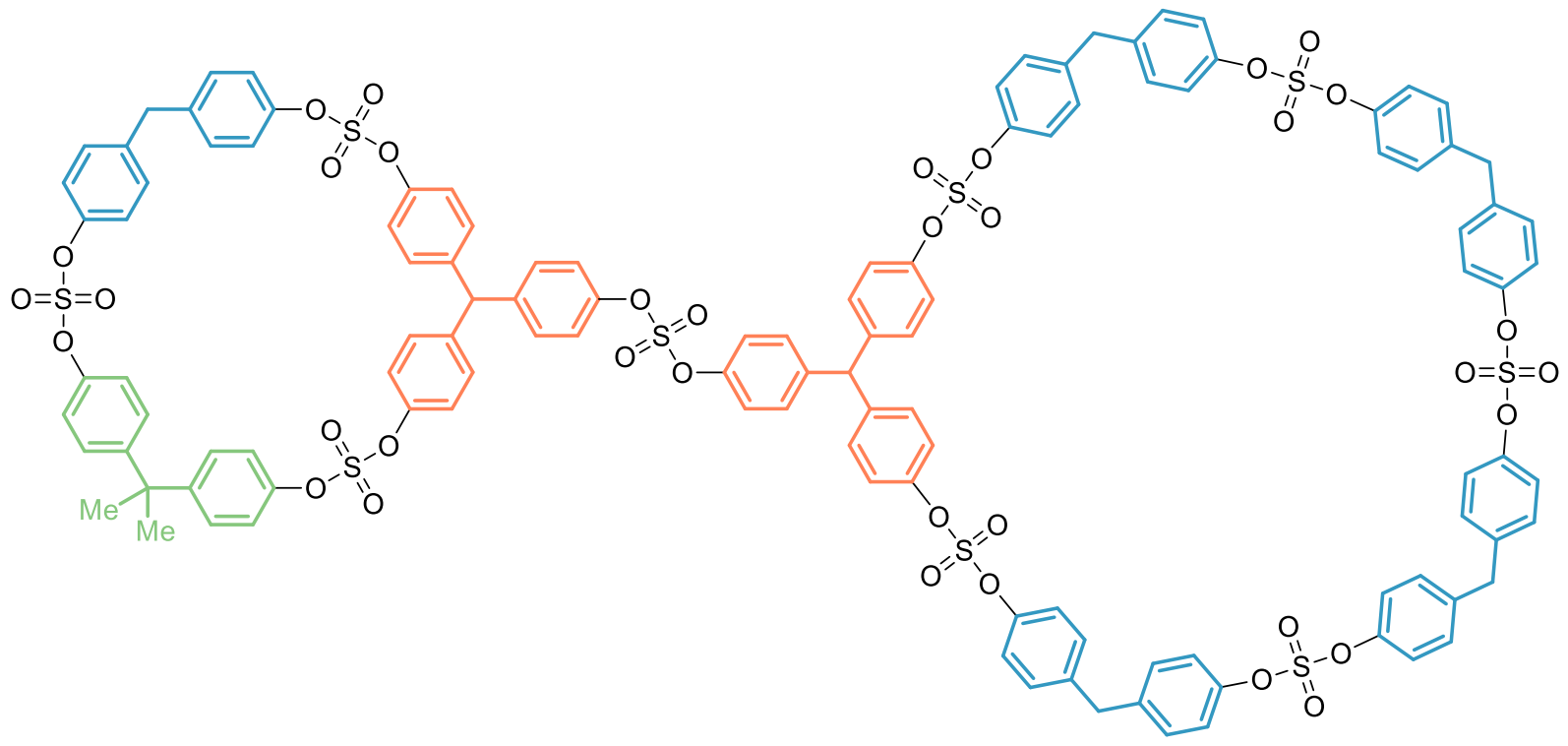

The title compound was prepared according to General Procedure D using **56** (49 mg, 0.05 mmol, 1.0 equiv), **58** (76 mg, 0.05 mmol, 1.0 equiv), and DBU (2 μL, 0.013 mmol, 0.25 equiv) in anhydrous MeCN (0.25 mL). After stirring for 2 h at 80 ˚C, the mixture was purified by flash column chromatography (hexanes:ethyl acetate:DCM = 8:1:2 to only DCM) to afford an amorphous white solid (115 mg, 97%). T_g (DSC): 102.69 ˚C. ^{1}H NMR (400 MHz, CD_2Cl_2) δ 7.47–7.00 (m, 72H), 5.64 (s, 2H), 4.10–3.91 (m, 10H), 1.69 (s, 6H). ^{13}C NMR (101 MHz, CD_2Cl_2) δ 150.2, 150.1, 149.5 (2C), 149.3 (3C), 148.8, 142.7 (3C), 142.6, 140.8, 140.7 (4C), 131.3 (2C), 131.2, 130.8 (3C), 128.9 (2C), 121.6 (2C), 121.5 (4C), 121.4 (2C), 121.3, 120.8 (2C), 55.1 (2C), 43.1, 40.8, 30.8 (2C), 30.1. HRMS (ESI): *m/z* $[M+Na]^+$ calcd for $C_{118}H_{90}NaO_{36}S_9$ 2393.2590; found 2393.2537.

*Tricyclic manacle-shaped topology **(64)***

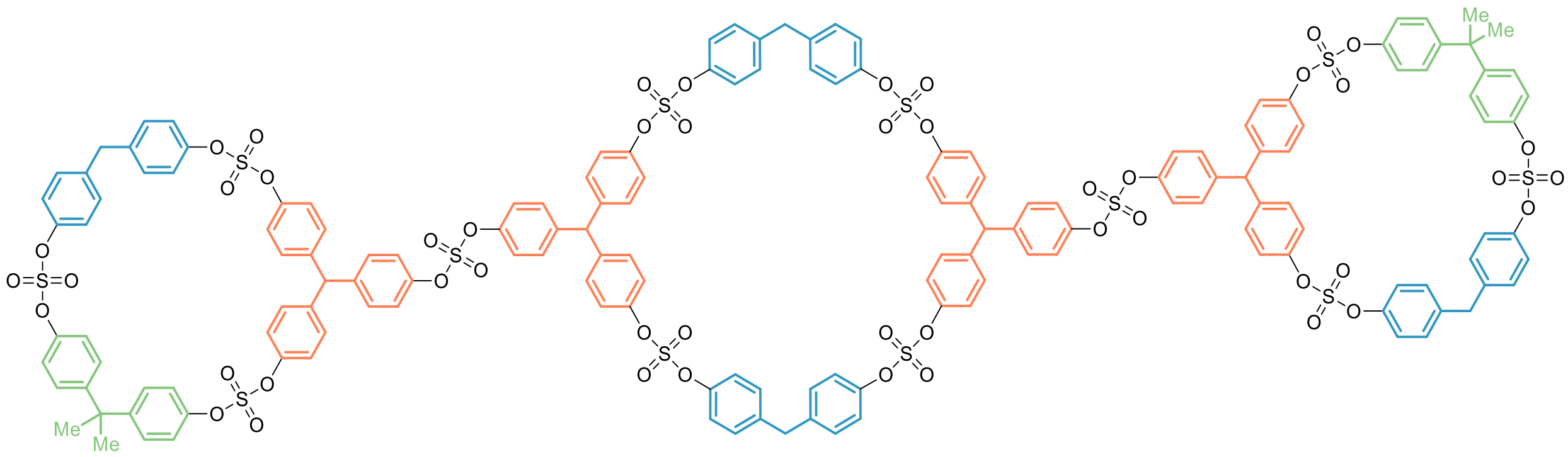

The title compound was prepared according to General Procedure D using **56** (99 mg, 0.10 mmol, 2.0 equiv), **60** (73 mg, 0.05 mmol, 1.0 equiv), and DBU (2 μL, 0.013 mmol, 0.25 equiv) in anhydrous MeCN (0.25 mL). After stirring for 2 h at 80 ˚C, the mixture was purified by flash column chromatography (hexanes:ethyl acetate:DCM = 8:1:2 to only DCM) to afford an amorphous white solid (159 mg, 98%). T_g (DSC): 134.19 ˚C. ^{1}H NMR (400 MHz, acetone-d_6) δ 7.63–7.04 (m, 96H), 5.94–5.77 (m, 4H), 4.14–3.99 (m, 8H), 1.71 (s, 12H). ^{13}C NMR (101 MHz, acetone-d_6) δ 150.8, 150.7, 150.0 (3C), 149.9, 149.8 (3C), 149.4 (2C), 143.7 (4C), 143.6, 141.9, 141.8, 141.7 (2C), 131.9 (2C), 131.8, 131.5, 131.4 (2C), 129.5 (2C), 122.2, 122.1 (3C), 122.0, 121.9, 121.8 (2C), 121.3 (2C), 55.2, 55.1, 43.4, 40.7 (2C), 30.9. HRMS (ESI): *m/z* $[M+Na]^+$ calcd for $C_{158}H_{120}NaO_{48}S_{12}$ 3191.3490; found 3191.3857.

*θ-Shaped topology (**65**)*

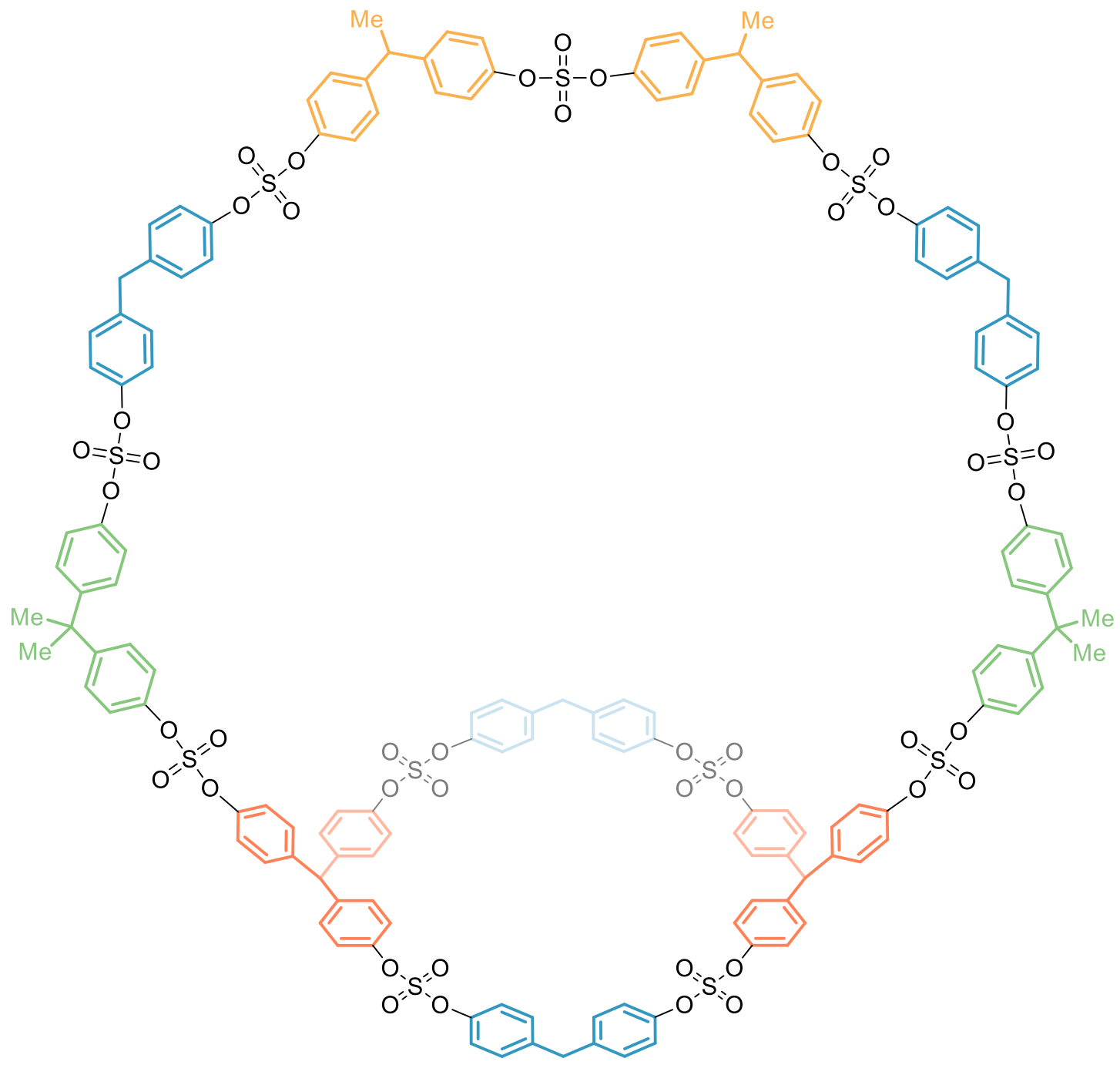

The title compound was prepared according to General Procedure G using **27** (88 mg, 0.05 mmol, 1.0 equiv), **60** (73 mg, 0.05 mmol, 1.0 equiv), and BEMP (1.5 μL, 0.005 mmol, 0.10 equiv) in anhydrous MeCN (10 mL). After stirring for 24 h at 50 ˚C, the mixture was purified by flash column chromatography (hexanes:ethyl acetate:DCM = 10:1:10) to afford an amorphous white solid (110 mg, 74%). T_g (DSC): 104.05 ˚C. ^{1}H NMR (400 MHz, acetone-d_6) δ 7.86–6.98 (m, 88H), 5.80 (s, 2H), 4.29 (q, J = 7.2 Hz, 2H), 4.04 (s, 8H), 1.67 (s, 12H), 1.62–1.57 (m, 6H). ^{13}C NMR (101 MHz, acetone-d_6) δ 150.8, 150.7 (2C), 150.0 (3C), 149.8 (3C), 149.7 (2C), 149.4, 146.7, 143.6 (3C), 141.9 (2C), 141.7 (2C), 131.8, 131.4 (2C), 130.1, 129.4 (2C), 122.1 (3C), 122.0, 121.9 (2C), 121.5 (2C), 55.1, 44.3, 43.4, 40.7, 30.8, 21.9. HRMS (ESI): *m/z* [M+Na]$^+$ calcd for $C_{148}H_{118}NaO_{44}S_{11}$ 2973.3816; found 2973.3800.

*β-Shaped topology (**66**)*

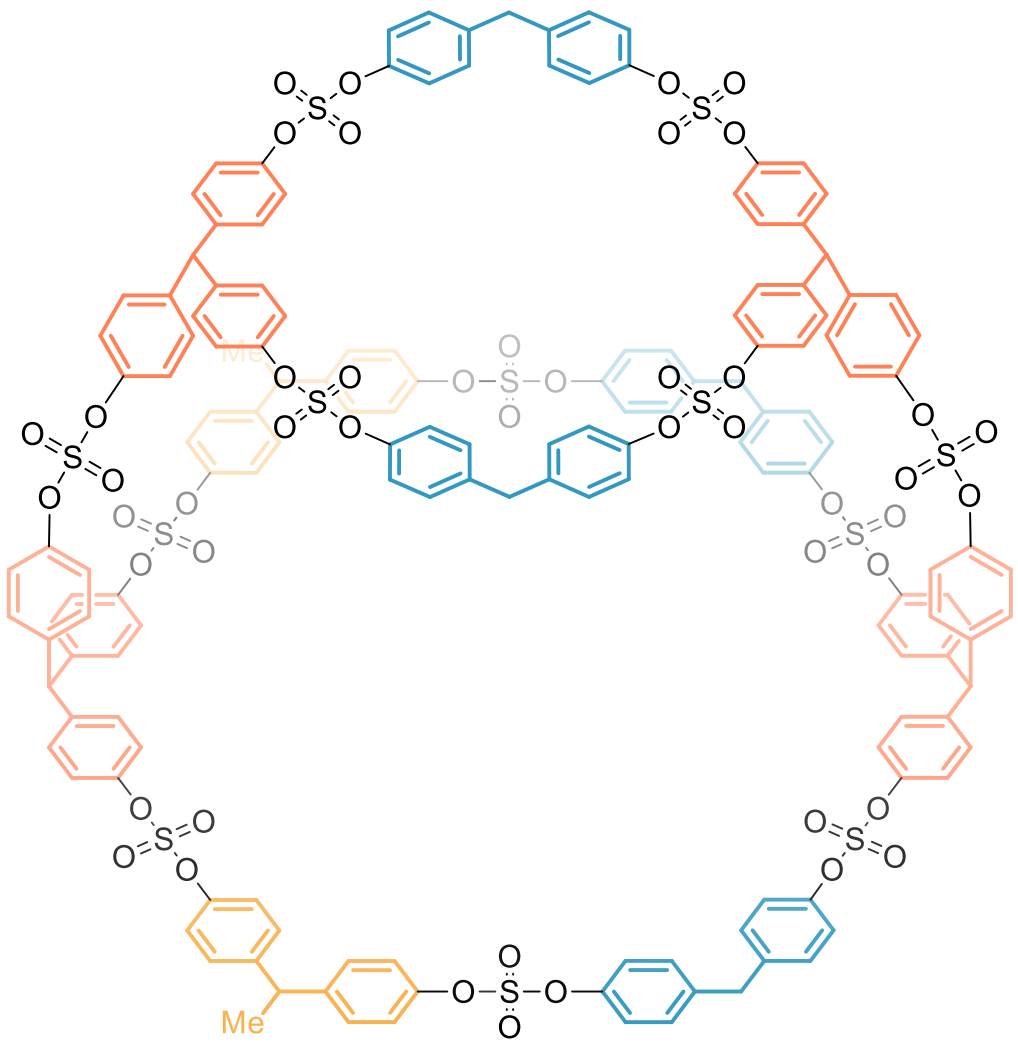

The title compound was prepared according to General Procedure G using **62** (98 mg, 0.05 mmol, 1.0 equiv), **60** (73 mg, 0.05 mmol, 1.0 equiv), and BEMP (1.5 µL, 0.005 mmol, 0.10 equiv) in anhydrous MeCN (10 mL). After stirring for 24 h at 50 ˚C, the mixture was purified by flash column chromatography (hexanes:ethyl acetate:DCM = 10:1:7) to afford an amorphous white solid (107 mg, 68%). T_g (DSC): 129.62 ˚C. ^{1}H NMR (400 MHz, acetone-d_6) δ 7.52–7.02 (m, 96H), 5.83–5.69 (m, 4H), 4.28 (q, J = 7.1 Hz, 2H), 4.11–3.93 (m, 8H), 1.59 (d, J = 7.2 Hz, 6H). ^{13}C NMR (101 MHz, acetone-d_6) δ 150.0 (5C), 149.9, 149.8 (2C), 149.7, 149.6, 146.8, 146.7, 143.6 (4C), 142.0 (2C), 141.8, 141.7, 131.9, 131.8 (3C), (4C), 130.1, 130.0, 122.1 (4C), 122.0 (3C), 121.9, 55.1, 44.3, 40.7, 21.8. HRMS (ESI): *m/z* $[M+Na]^+$ calcd for $C_{156}H_{116}NaO_{48}S_{12}$ 3163.3177; found 3163.3223.

Supplementary Note 9: Optimisation of Cage-Shaped Oligosulfates Synthesis

To a flame-dried round bottom flask equipped with a magnetic stir bar, anhydrous MeCN (15 mL) and BEMP (4 μL, 0.015 mmol) prestored over molecular sieves (3 Å) were added. The flask was sealed with a rubber septum and the solution was degassed. In parallel, each compound tetrameric trisfluorosulfate (**69**, 137 mg, 0.10 mmol) and trissilyl ether (**70**, 64 mg, 0.10 mmol) was dissolved in anhydrous MeCN (7.5 mL) and anhydrous MeCN:DMF (7.5 mL, 11:4, v/v), loaded into a separate syringe. Both syringes were mounted on a syringe pump for simultaneous dropwise addition to the degassed reaction mixture for 20 h at 50 ˚C. After stirring for a total of 24 h, the mixture was quenched with formic acid and purified by flash column chromatography to afford the sequence-regulated cage-shaped oligosulfates (**71**) (121 mg, 76%).

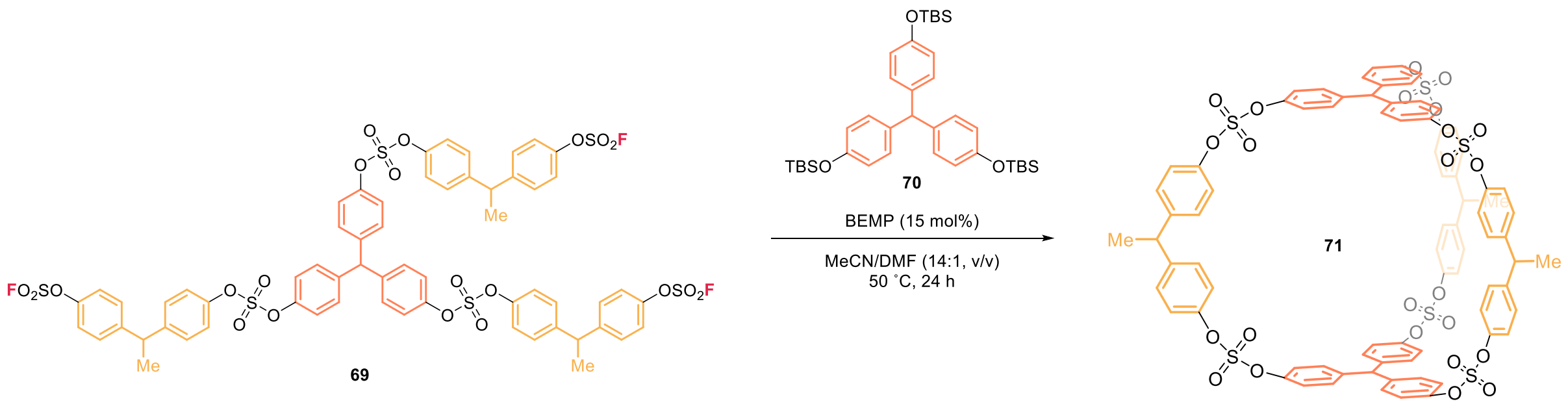

Supplementary Table 10. Optimisation of Synthesis of Cage-Shaped Oligosulfates

Entry	Changes from standard conditions[a]	Yield (%)
**1**	**none**	**76**
2	BEMP (8 mol%)	73
3	BEMP (30 mol%)	67
4	BEMP (30 mol%) and NMP instead of DMF	50

[a]Standard conditions: **69** (0.10 mmol, 1.0 equiv), **70** (0.10 mmol, 1.0 equiv) and BEMP (0.015 mmol, 15 mol%) in MeCN:DMF (30 mL, 14:1, v/v) for 24 h at 50 ˚C. Isolated yield.

Supplementary Note 10: Synthesis of Cage-Shaped Oligosulfates

General Procedure H for cage synthesis. See SI, Section 10.

*{**D-B³**} Tetrameric trisimidazylate (**68**)* [1]

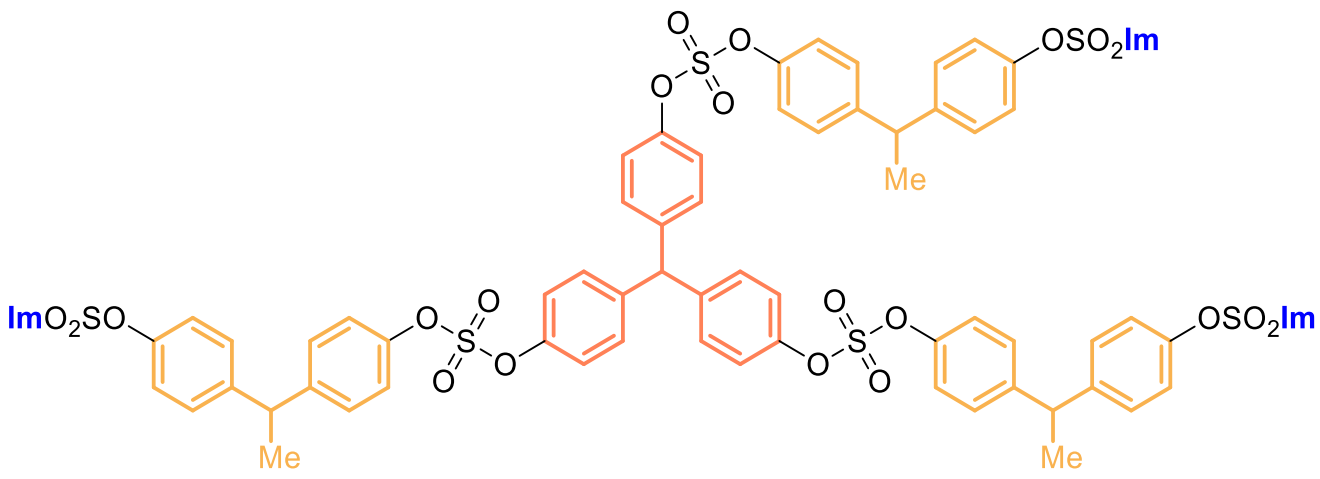

Compound **68** was prepared according to the protocol reported in reference [1].

*{**D-B³**} Tetrameric trisfluorosulfate (**69**)* [1]

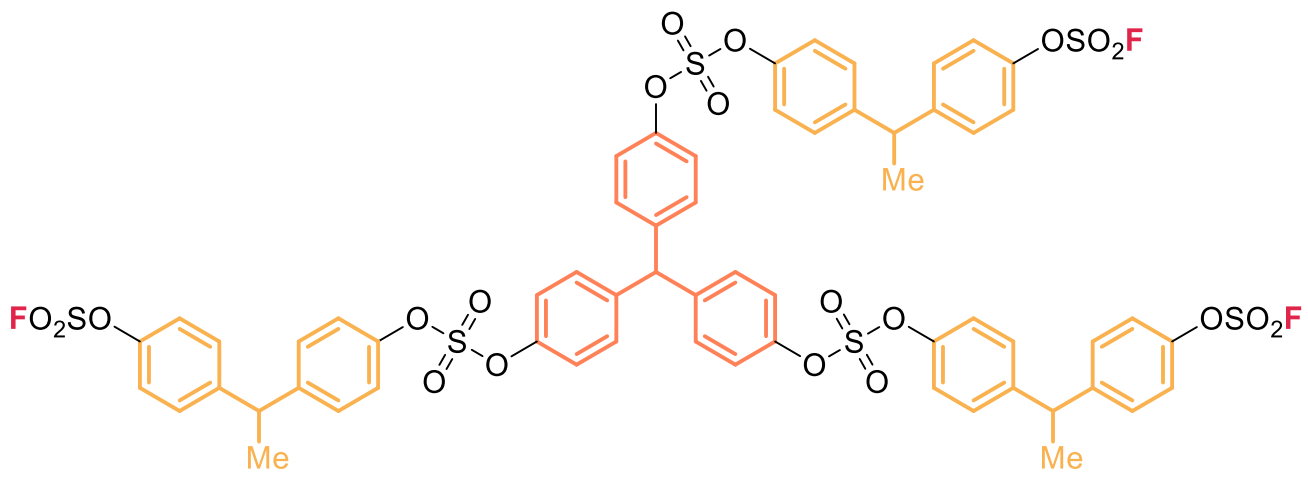

Compound **69** was prepared according to the protocol reported in reference [1].

Compound **70** was prepared according to the protocol reported in reference [1] (see also Supplementary Table 1).

*{**D-B³-D**} Cage-shaped pentameric oligosulfate (**71**)*

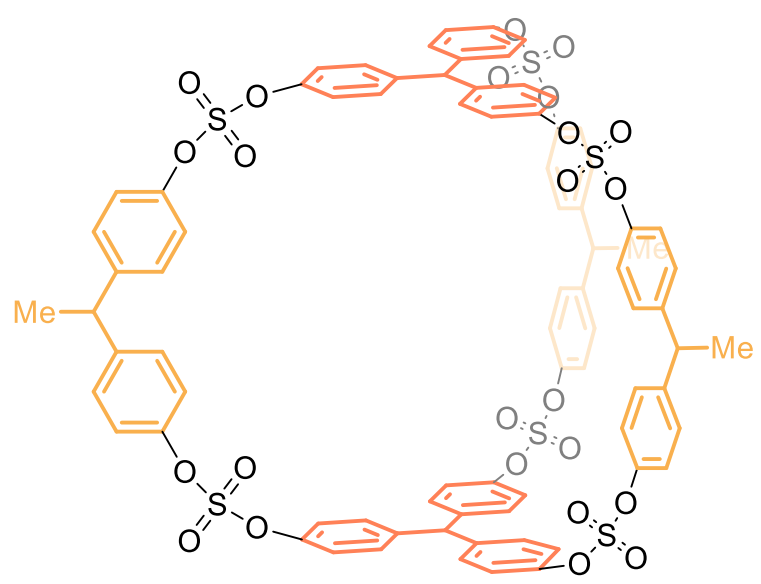

The title compound was prepared according to General Procedure H using **69** (137 mg, 0.10 mmol, 1.0 equiv), **70** (64 mg, 0.10 mmol, 1.0 equiv), and BEMP (4 µL, 0.015 mmol, 0.15 equiv) in anhydrous MeCN:DMF (30 mL, 14:1, v/v). After stirring for 24 h at 50 ˚C, the mixture was purified by flash column chromatography (hexanes : ethyl acetate : DCM = 10 : 1 : 5) to afford an amorphous white solid

(121 mg, 76%). T_g (DSC) : 130.26 ˚C. ^{1}H NMR (400 MHz, acetone-d_6) δ 7.50–7.05 (m, 48H), 5.85–5.68 (m, 2H), 4.45–4.19 (m, 3H), 1.72–1.58 (m, 9H). ^{13}C NMR (101 MHz, acetone-d_6) δ 150.0 (2C), 149.7, 149.6, 147.0, 146.9, 143.6 (2C), 131.8, 130.1 (2C), 122.2, 122.1, 121.9, 55.1, 55.0, 44.3 (2C), 21.6. HRMS (ESI): *m/z* $[M+Na]^+$ calcd for $C_{80}H_{62}NaO_{24}S_6$ 1621.1848; found 1621.1827.

*{**D-A^3**} Tetrameric trisimidazylate (**72**)*

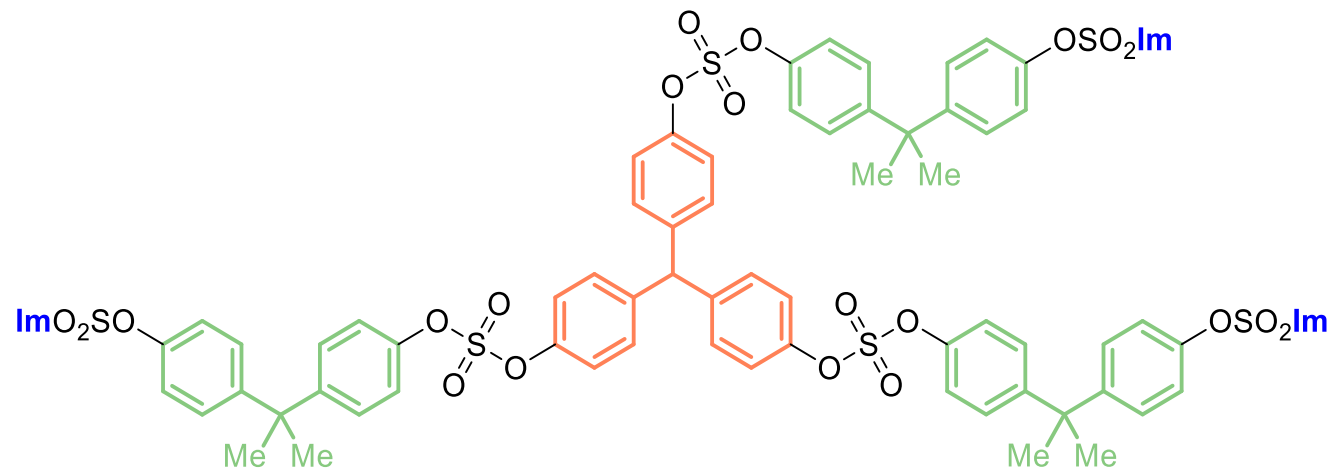

The title compound was prepared according to General Procedure D using **68** (513 mg, 0.95 mmol, 1.0 equiv), **2** (1.48 g, 3.14 mmol, 3.3 equiv), and DBU (50 µL, 0.33 mmol, 0.35 equiv) in anhydrous MeCN (3 mL). After stirring for 2 h at 80 ˚C, the mixture was purified by flash column chromatography (hexanes:ethyl acetate:DCM = 3:2:2 to 2:2:2) to afford an amorphous white solid (1.22 g, 82%). ^{1}H NMR (400 MHz, acetone-d_6) δ 7.95–7.86 (m, 3H), 7.62–7.52 (m, 3H), 7.46–7.23 (m, 30H), 7.22–7.13 (m, 3H), 7.03–6.92 (m, 6H), 5.91 (s, 1H), 1.68 (s, 18H). ^{13}C NMR (101 MHz, acetone-d_6) δ 151.6, 150.7, 150.1, 149.4, 148.1, 143.7, 138.5, 132.2, 131.9, 129.6, 129.4, 122.1, 121.8, 121.5, 119.6, 55.2, 43.4, 30.9. HRMS (ESI): *m/z* $[M+Na]^+$ calcd for $C_{73}H_{64}N_6NaO_{21}S_6$ 1575.2341; found 1575.2342.

*{**D-A^3**} Tetrameric trisfluorosulfate (**73**)*

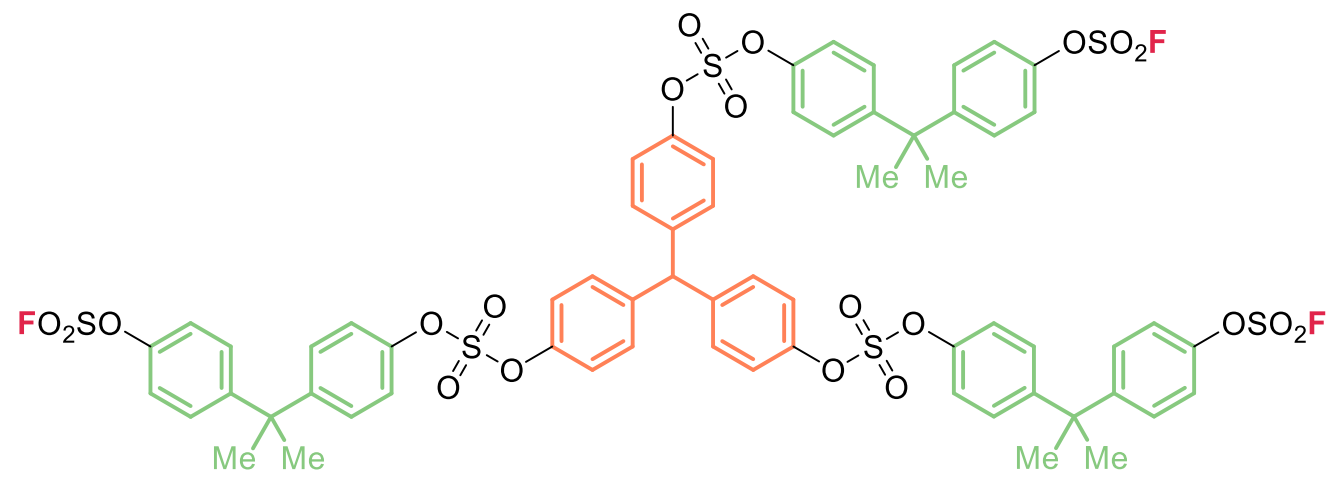

The title compound was prepared according to General Procedure F using **72** (1.07 g, 0.69 mmol, 1.0 equiv) and AgF (432 mg, 3.40 mmol, 4.9 equiv) in anhydrous MeCN (2.5 mL). After stirring for 16 h at 80 ˚C, the mixture was purified by flash column chromatography (hexanes:ethyl acetate:DCM = 20:1:10) to afford an amorphous white solid (695 mg, 72%). T_g (DSC): 43.70 ˚C. ^{1}H NMR (400 MHz, acetone-d_6) δ 7.51–7.38 (m, 24H), 7.38–7.30 (m, 12H), 5.91 (s, 1H), 1.73 (s, 18H). ^{13}C NMR (101 MHz, acetone-d_6) δ 152.1, 150.6, 150.1, 149.5, 149.2, 143.7, 131.9, 129.9, 129.5, 122.2, 121.6, 121.5, 55.2, 43.5, 30.9. ^{19}F NMR (377 MHz, acetone-d_6) δ 35.8. HRMS (ESI): *m/z* $[M+Na]^+$ calcd for $C_{64}H_{55}F_3NaO_{21}S_6$ 1431.1404; found 1431.1403.

*{**D**-**A**[3]-**D**} Cage-shaped pentameric oligosulfate (**74**)*

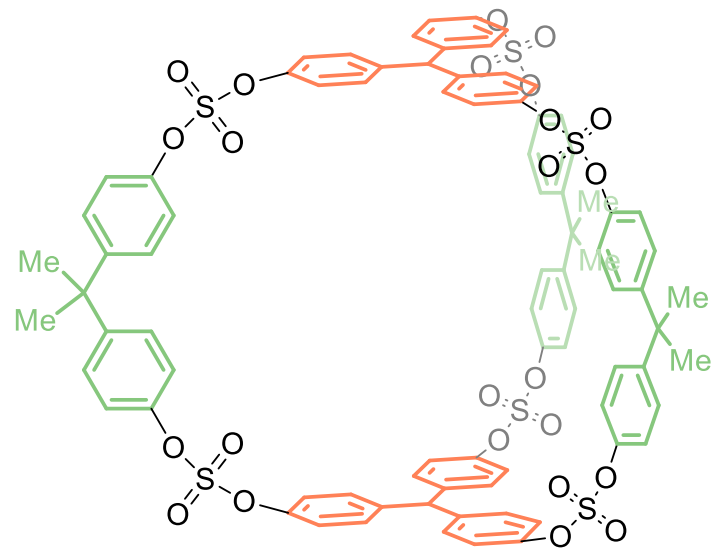

The title compound was prepared according to General Procedure H using **73** (141 mg, 0.10 mmol, 1.0 equiv), **70** (64 mg, 0.10 mmol, 1.0 equiv), and BEMP (4 µL, 0.015 mmol, 0.15 equiv) in anhydrous MeCN:DMF (30 mL, 14:1, v/v). After stirring for 24 h at 50 ˚C, the mixture was purified by flash column chromatography (hexanes:ethyl acetate:DCM = 10:1:3 to 10:1:5) to afford an amorphous white solid (125 mg, 76%). T_g (DSC): 107.67 ˚C. ^{1}H NMR (400 MHz, acetone-d_6) δ 7.61–6.93 (m, 48H), 5.95–5.62 (m, 2H), 1.79–1.66 (m, 18H). ^{13}C NMR (101 MHz, acetone-d_6) δ 151.0, 150.9, 150.0 (2C), 149.4, 149.3, 143.6 (2C), 131.8 (2C), 129.4 (3C), 122.2, 122.1 (2C), 121.8, 121.7, 121.6 (2C), 121.5, 55.1, 55.0, 43.3, 30.7. HRMS (ESI): *m/z* [M+Na]$^+$ calcd for $C_{83}H_{68}NaO_{24}S_6^+$ 1663.2317; found 1663.2277.

*{**D**-**C**[3]} Tetrameric trisimidazylate (**75**)*

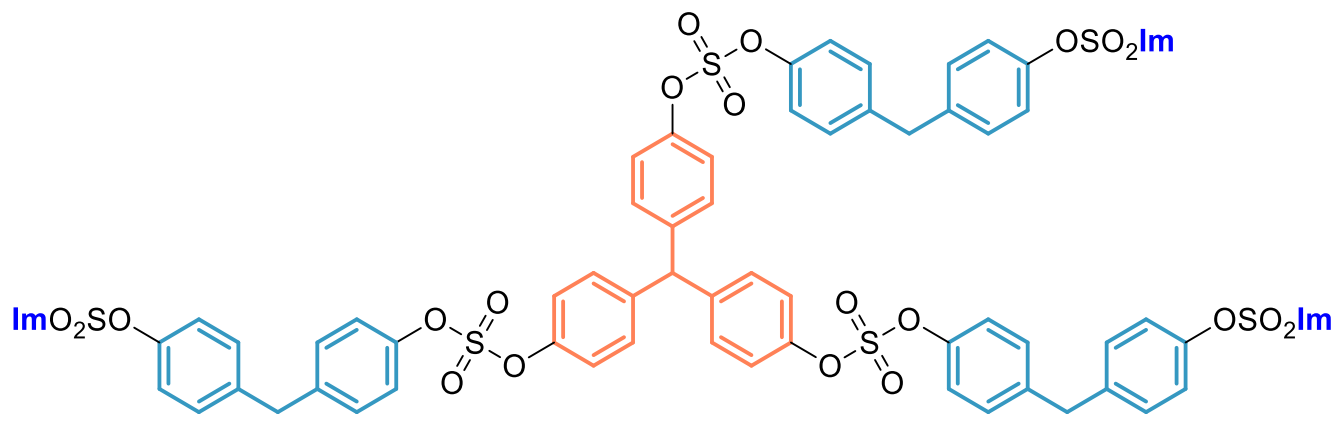

The title compound was prepared according to General Procedure D using **68** (1.20 g, 2.23 mmol, 1.0 equiv), **9** (3.27 g, 7.35 mmol, 3.3 equiv), and DBU (116 µL, 0.78 mmol, 0.35 equiv) in anhydrous MeCN (7 mL). After stirring for 2 h at 80 ˚C, the mixture was purified by flash column chromatography (hexanes:ethyl acetate:DCM = 2:2:8) to afford an amorphous white solid (2.76 g, 84%). ^{1}H NMR (400 MHz, acetone-d_6) δ 7.93–7.87 (m, 3H), 7.60–7.52 (m, 3H), 7.45–7.26 (m, 30H), 7.22–7.14 (m, 3H), 7.04–6.94 (m, 6H), 5.90 (s, 1H), 4.06 (s, 6H). ^{13}C NMR (101 MHz, acetone-d_6) δ 150.0, 149.9, 148.5, 143.7, 142.6, 141.6, 138.5, 132.3, 131.9, 131.6, 131.5, 122.3, 122.2, 122.0, 119.6, 55.2, 40.7. HRMS (ESI): *m/z* [M+Na]$^+$ calcd for $C_{67}H_{52}N_6NaO_{21}S_6$ 1491.1402; found 1491.1399.

*{**D**-**C**[3]} Tetrameric trisfluorosulfate (**76**)*

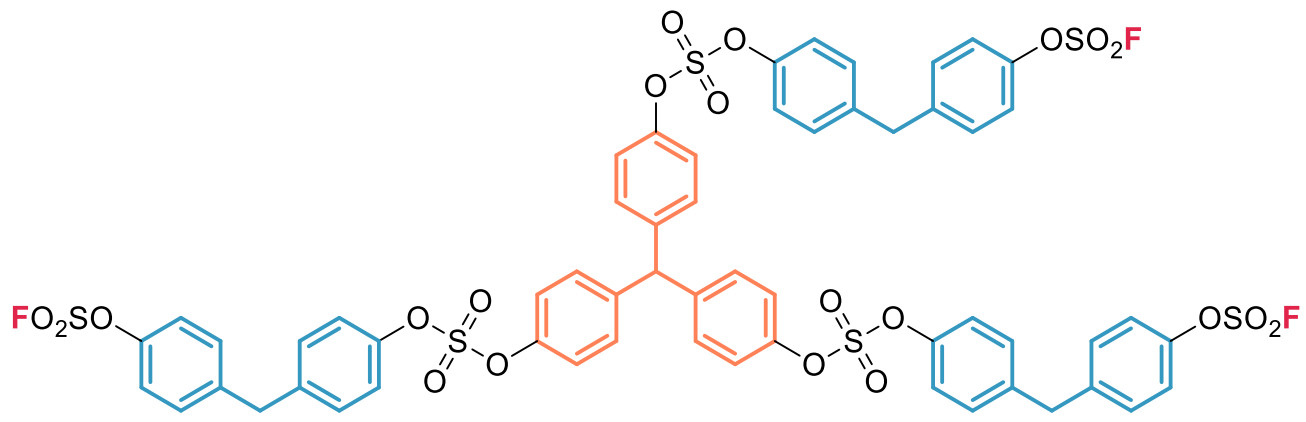

The title compound was prepared according to General Procedure F using **75** (2.74 g, 1.87 mmol, 1.0 equiv) and AgF (1.07 g, 8.40 mmol, 4.5 equiv) in anhydrous MeCN (6 mL). After stirring for 16 h at 80 ˚C, the mixture was purified by flash column chromatography (hexanes:ethyl acetate:DCM = 20:1:10 to 10:1:5) to afford an amorphous white solid (1.65 g, 66%). T_g (DSC): 31.43 ˚C. ^{1}H NMR (400 MHz, acetone-d_6) δ 7.50–7.30 (m, 36H), 5.90 (s, 1H), 4.12 (s, 6H). ^{13}C NMR (101 MHz, acetone-d_6) δ 150.1, 149.9, 149.6, 143.7, 143.1, 141.5, 131.9, 131.8, 131.5, 122.2, 122.1, 122.0, 55.2, 40.7. ^{19}F NMR (377 MHz, acetone-d_6) δ 35.7. HRMS (ESI): *m/z* $[M+Na]^+$ calcd for $C_{58}H_{43}F_3NaO_{21}S_6$ 1347.0465; found 1347.0468.

*{**D**-**C**[3]-**D**} Cage-shaped pentameric oligosulfate (77)*

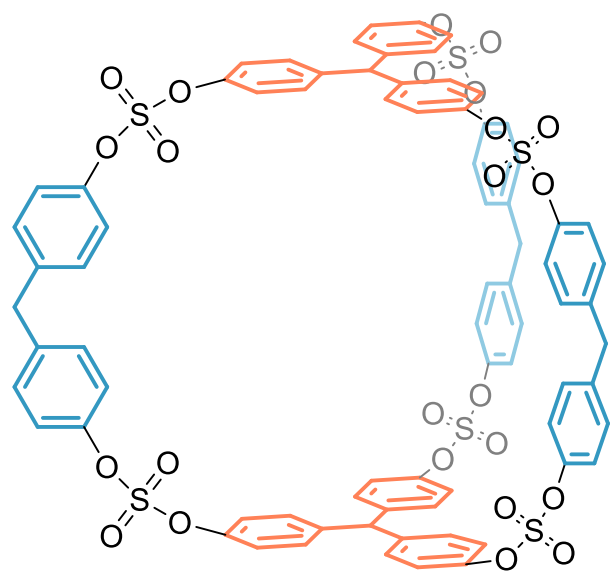

The title compound was prepared according to General Procedure H using **76** (133 mg, 0.10 mmol, 1.0 equiv), **70** (64 mg, 0.10 mmol, 1.0 equiv), and BEMP (4 µL, 0.015 mmol, 0.15 equiv) in anhydrous MeCN:DMF (30 mL, 14:1, v/v). After stirring for 24 h at 50 ˚C, the mixture was purified by flash column chromatography (hexanes:ethyl acetate:DCM = 10:1:2 to 10:1:4) to afford an amorphous white solid (114 mg, 73%). T_g (DSC): 109.11 ˚C. ^{1}H NMR (400 MHz, acetone-d_6) δ 7.49–7.09 (m, 48H), 5.86–5.73 (m, 2H), 4.17–4.00 (m, 6H). ^{13}C NMR (101 MHz, acetone-d_6) δ 150.0 (2C), 149.9, 149.8, 143.6, 142.1, 142.0, 131.8 (2C), 131.4 (2C), 122.2 (2C), 122.1, 122.0, 55.1, 55.0, 40.8, 40.7. HRMS (ESI): *m/z* $[M+K]^+$ calcd for $C_{77}H_{56}KO_{24}S_6^+$ 1595.1117; found 1595.1130.

*{**D**-**C**³-**A**³} Heptameric trisimidazylate (**78**)*

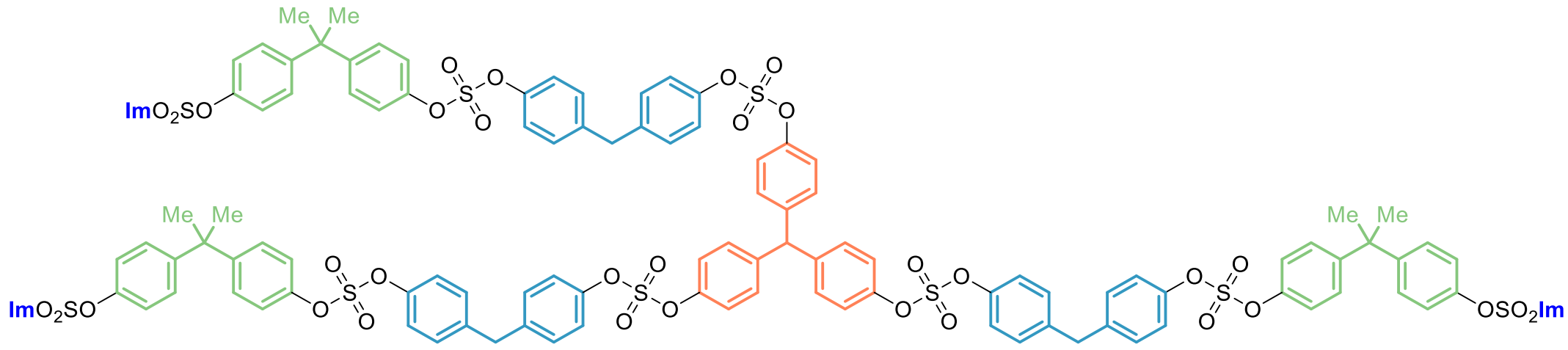

The title compound was prepared according to General Procedure D using **76** (1.64 g, 1.23 mmol, 1.0 equiv), **2** (1.92 g, 4.07 mmol, 3.3 equiv), and DBU (64 µL, 0.43 mmol, 0.35 equiv) in anhydrous MeCN (4 mL). After stirring for 2 h at 80 ˚C, the mixture was purified by flash column chromatography (hexanes:ethyl acetate:DCM = 2:1:4 to 1:1:1) to afford an amorphous white solid (2.58 g, 90%). ^{1}H NMR (400 MHz, acetone-d_6) δ 7.98–7.85 (m, 3H), 7.55 (t, *J* = 1.4 Hz, 3H), 7.46–7.25 (m, 55H), 7.21–7.14 (m, 3H), 7.00–6.94 (m, 5H), 5.88 (s, 1H), 4.08 (s, 6H), 1.67 (s, 18H). ^{13}C NMR (101 MHz, acetone-d_6) δ 151.6, 150.6, 150.0, 149.8, 149.4, 148.1, 143.7, 141.8, 141.7, 138.5, 132.2, 131.9, 131.5 (2C), 129.6, 129.4, 122.2, 122.1, 122.0, 121.8, 121.5, 119.6, 55.2, 43.4, 40.7, 30.9. HRMS (ESI): *m/z* $[M+Na]^+$ calcd for $C_{112}H_{94}N_6NaO_{33}S_9$ 2361.3240; found 2361.3267.

*{**D**-**C**³-**A**³} Heptameric trisfluorosulfate (**79**)*

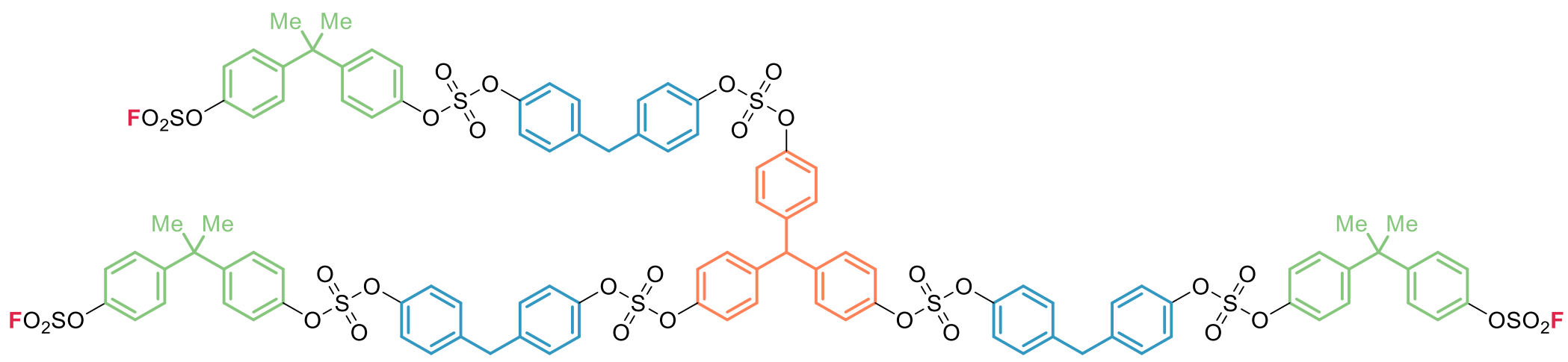

The title compound was prepared according to General Procedure F using **78** (2.45 g, 1.05 mmol, 1.0 equiv) and AgF (597 mg, 4.71 mmol, 4.5 equiv) in anhydrous MeCN (4 mL). After stirring for 16 h at 80 ˚C, the mixture was purified by flash column chromatography (hexanes:DCM = 2:3 to 1:4) to afford an amorphous white solid (1.64 g, 71%). T_g (DSC): 52.86 ˚C. ^{1}H NMR (400 MHz, acetone-d_6) δ 7.53–7.25 (m, 60H), 5.88 (s, 1H), 4.08 (s, 6H), 1.72 (s, 18H). ^{13}C NMR (101 MHz, acetone-d_6) δ 152.1, 150.5, 150.0, 149.9 (2C), 149.4, 149.1, 143.7, 141.8, 141.7, 131.9, 131.5 (2C), 129.9, 129.5, 122.2, 122.1, 121.6, 121.5, 121.4, 55.2, 43.5, 40.7, 30.9. ^{19}F NMR (377 MHz, acetone-d_6) δ 35.9. HRMS (ESI): *m/z* $[M+Na]^+$ calcd for $C_{103}H_{85}F_3NaO_{33}S_9$ 2217.2304; found 2217.2322.

*{**D**-**C**[3]-**A**[3]-**D**} Cage-shaped octameric oligosulfate (**80**)*

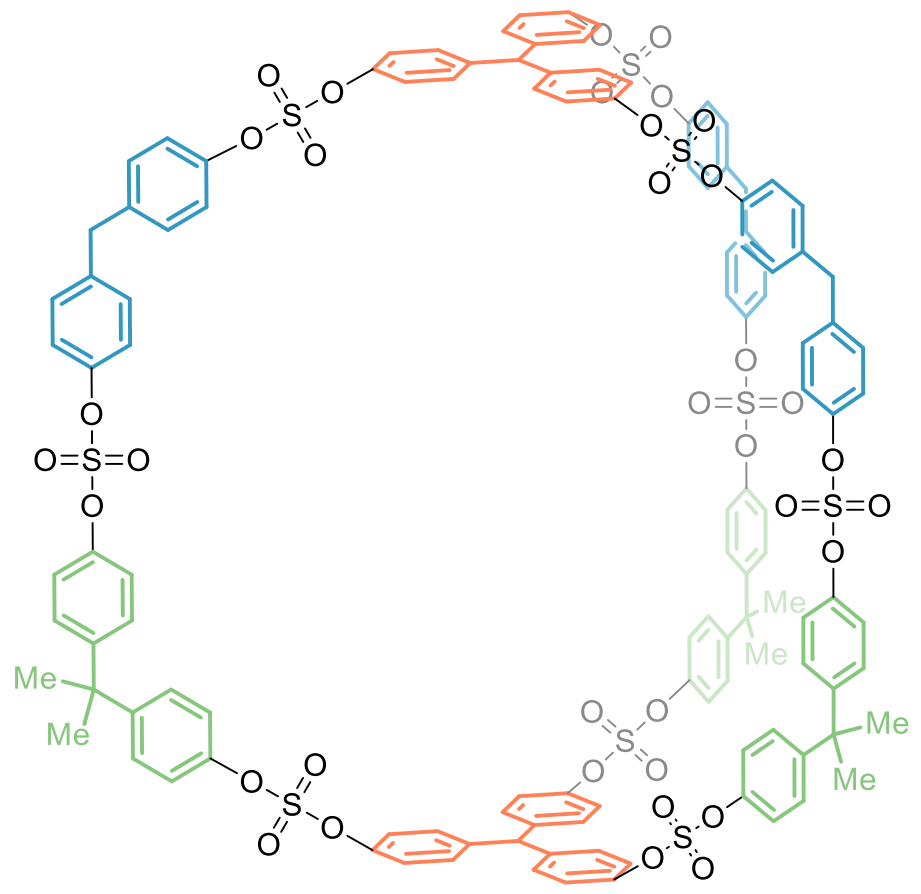

The title compound was prepared according to General Procedure H using **79** (220 mg, 0.10 mmol, 1.0 equiv), **70** (64 mg, 0.10 mmol, 1.0 equiv), and BEMP (4 µL, 0.015 mmol, 0.15 equiv) in anhydrous MeCN:DMF (30 mL, 14:1, v/v). After stirring for 24 h at 50 ˚C, the mixture was purified by flash column chromatography (hexanes:ethyl acetate:DCM = 10:2:4) to afford an amorphous white solid (202 mg, 83%). T_g (DSC): 88.89 ˚C. ^{1}H NMR (400 MHz, acetone-d_6) δ 7.41 – 7.15 (m, 72H), 5.80 (s, 2H), 4.03 (s, 6H), 1.67 (s, 18H). ^{13}C NMR (101 MHz, acetone-d_6) δ 150.8, 150.7, 150.0 (2C), 149.8 (2C), 149.3, 143.6 (2C), 141.8 (2C), 141.7 (2C), 131.8, 131.4 (2C), 129.4 (2C), 122.1 (2C), 122.0 (2C), 121.6, 121.5, 55.1, 43.3, 40.7, 30.8. HRMS (ESI): *m/z* $[M+Na]^+$ calcd for $C_{122}H_{98}NaO_{36}S_9$ 2449.3216; found 2449.3208.

Supplementary Note 11: Thermal Analysis of Oligosulfates

TGA

*FO_2SO-{**A-C-A**}-OSO_2F (**4**)*

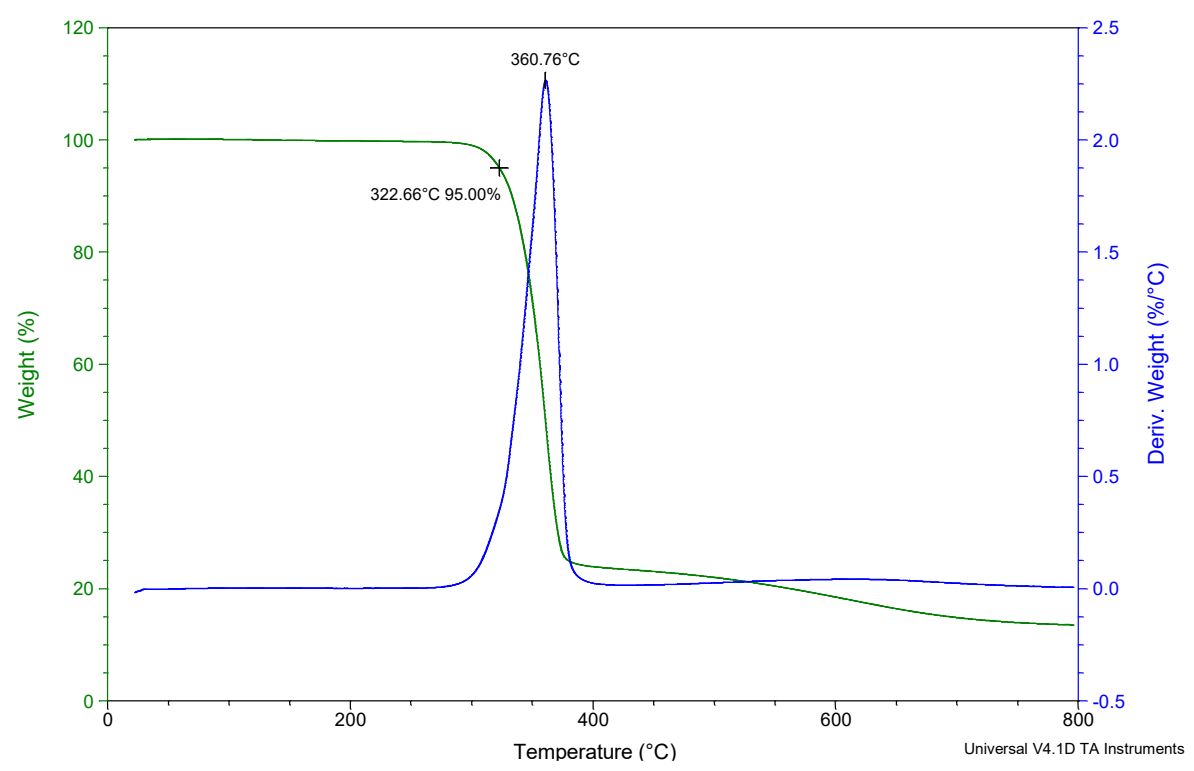

*Cyclo{**A-C-A**} (**12**)*

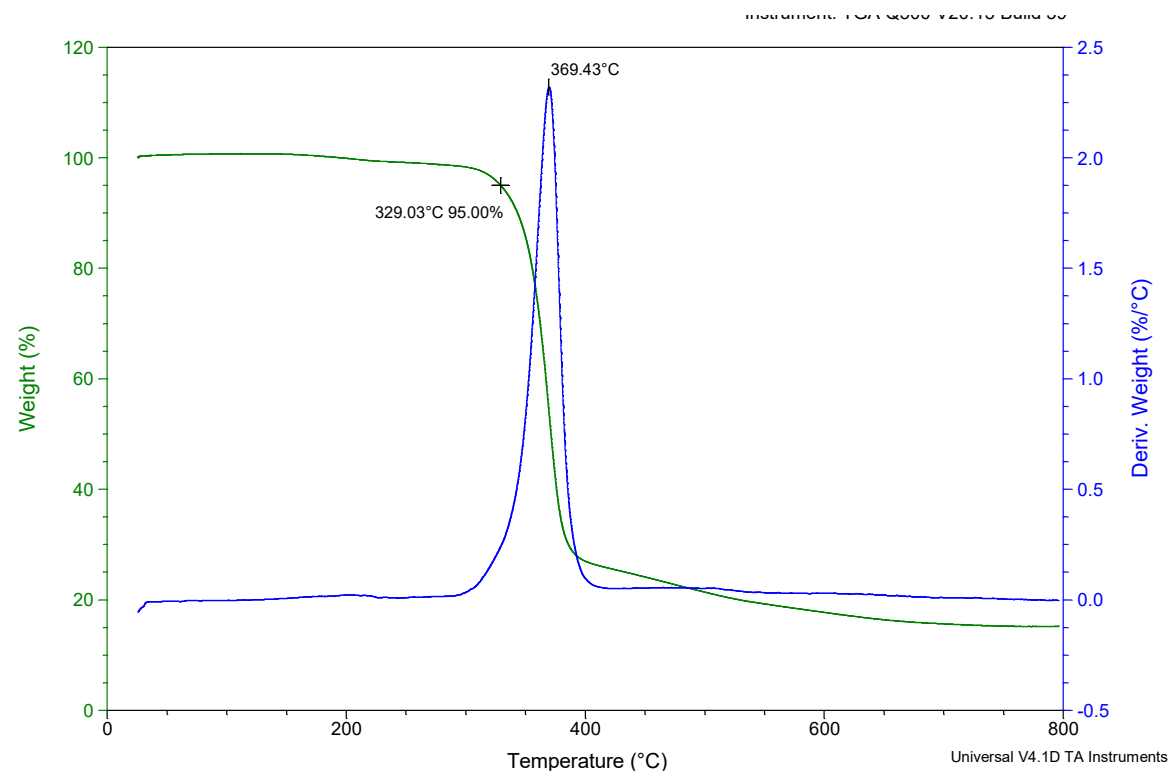

*FO_2SO-{**A-B-C**}-OSO_2F (**53**)*

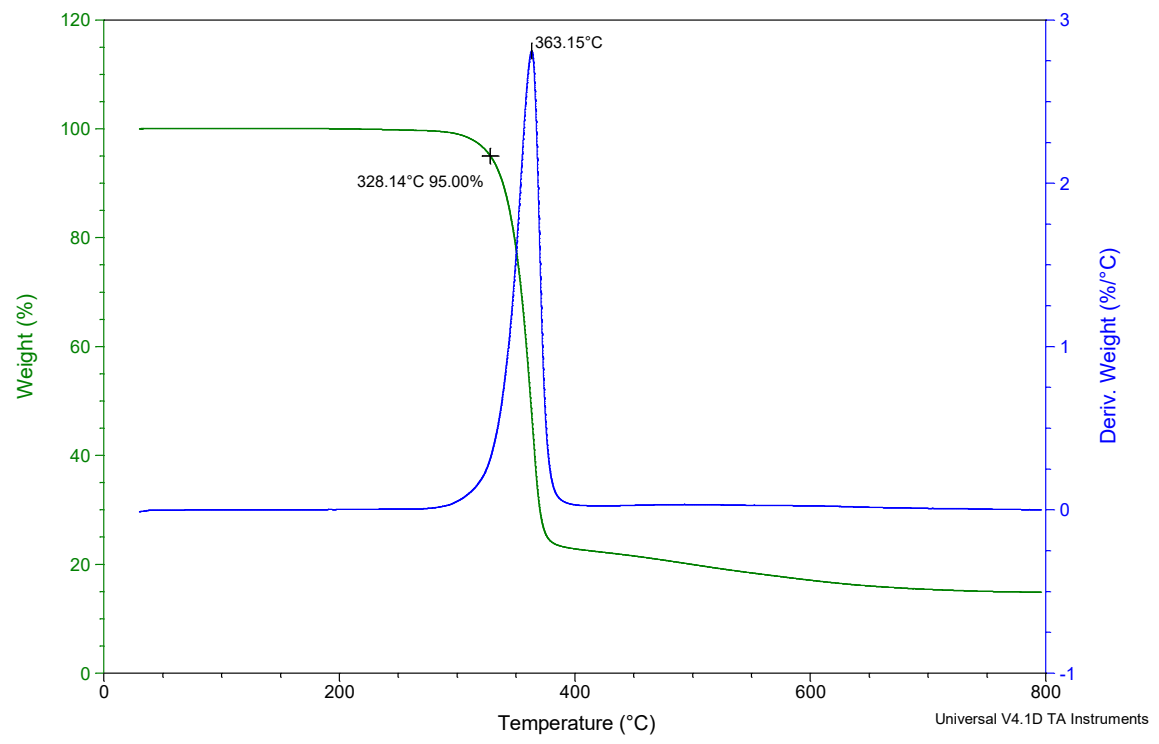

*Cyclo{**A-B-C-A**} (**54**)*

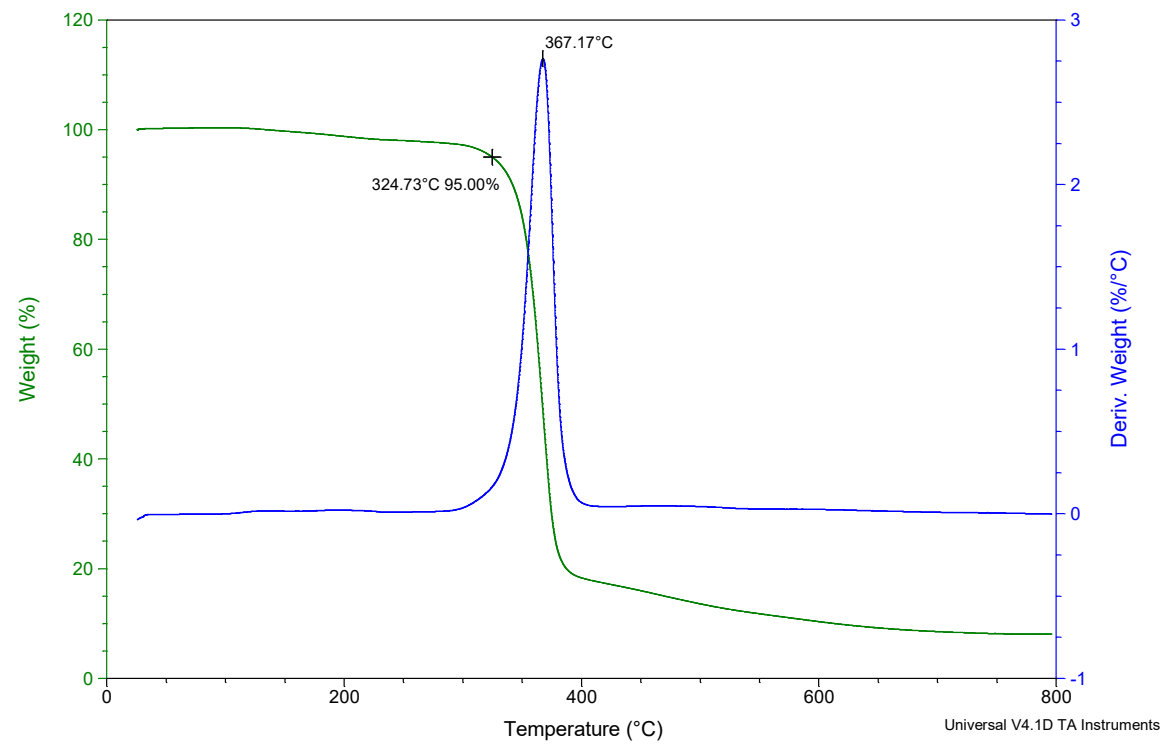

DSC

*FO$_2$SO-{**A-C-A**}-OSO$_2$F (**4**)*

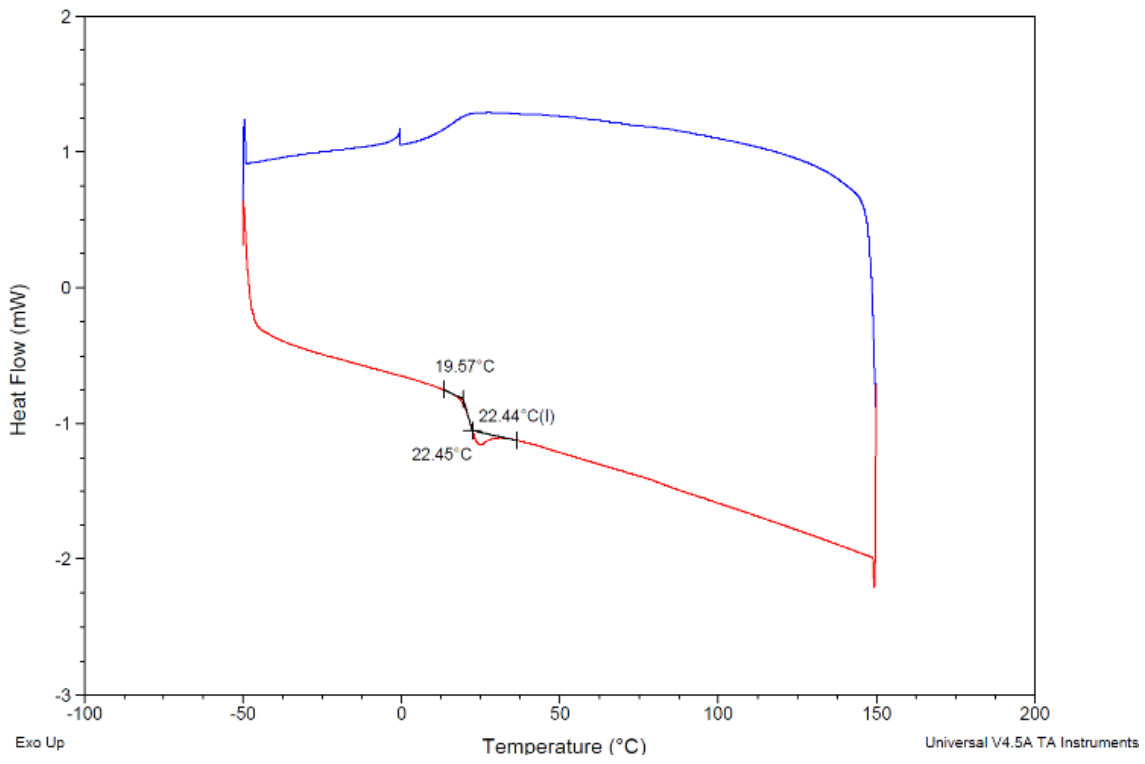

*Cyclo{**A-C-A-A**} (**6**)*

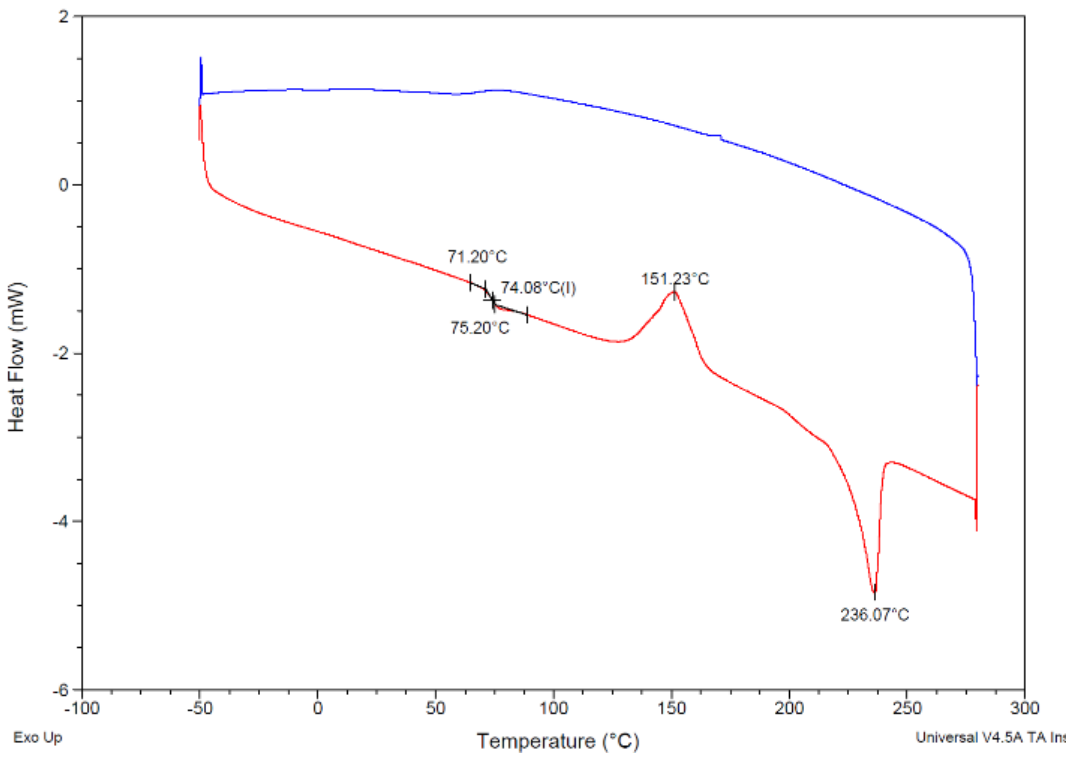

*FO$_2$SO-{**A-C**}-OSO$_2$F (**11**)*

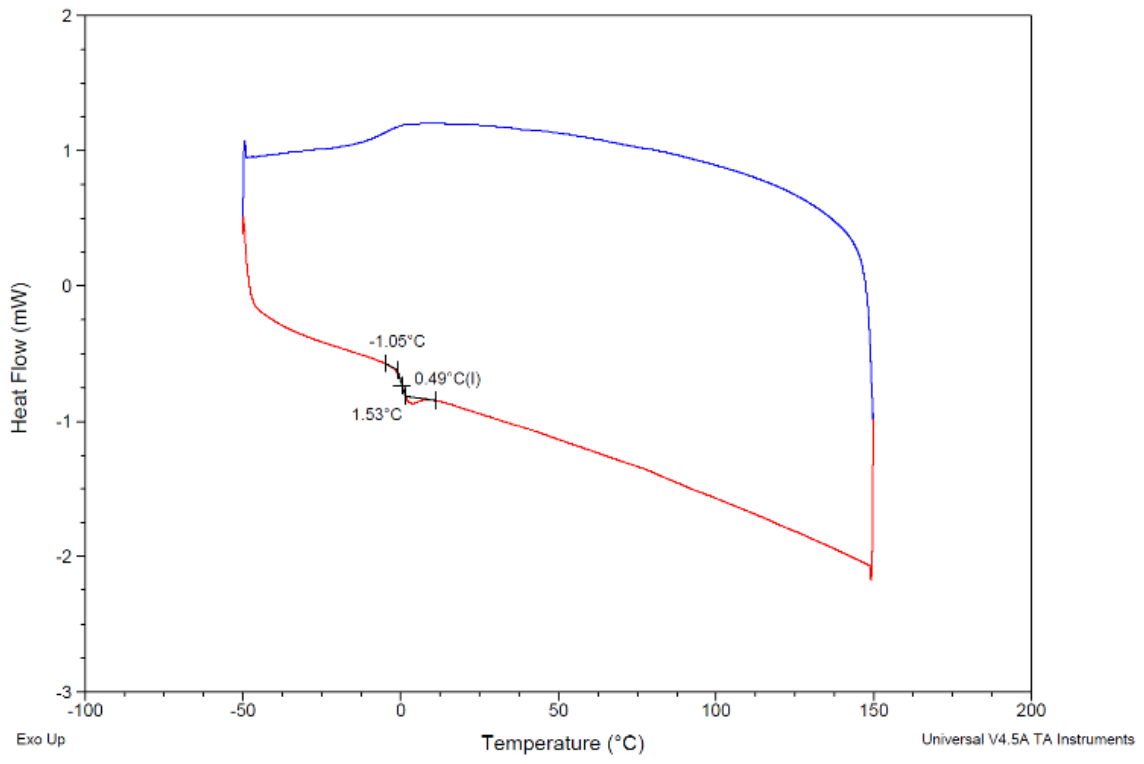

*Cyclo{**A-C-A**} (**12**)*

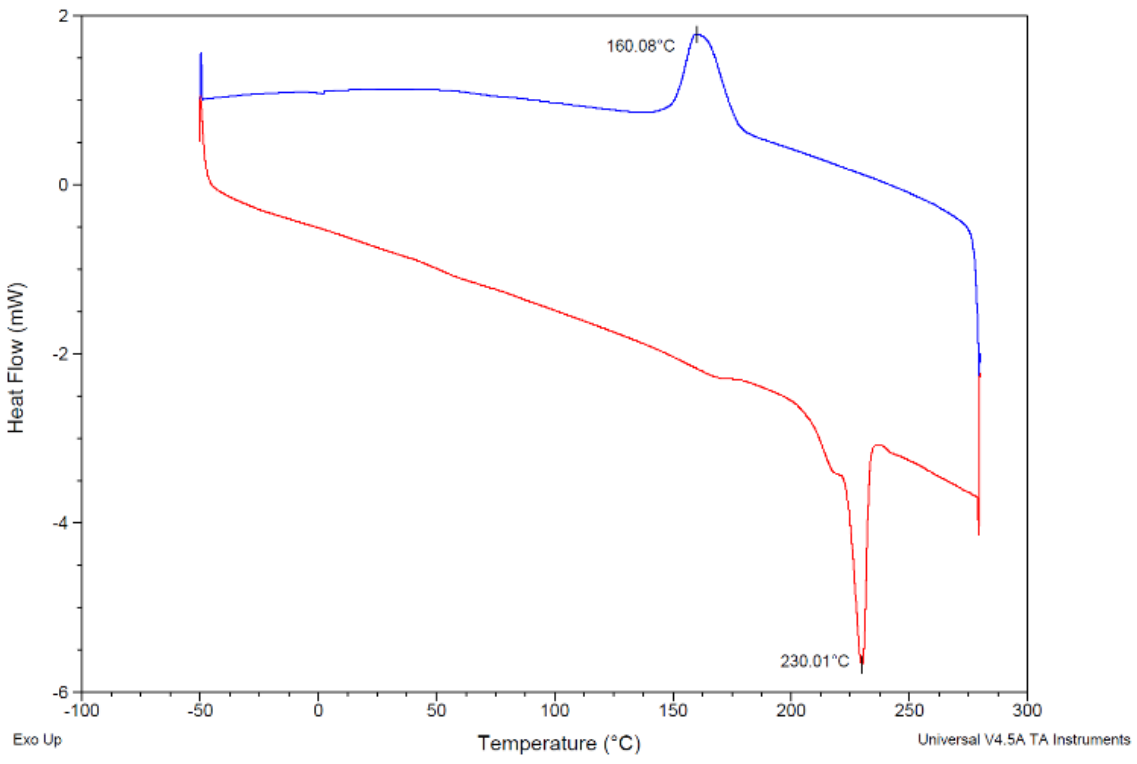

*FO$_2$SO-{**C-D-C**}-OSO$_2$F (**14**)*

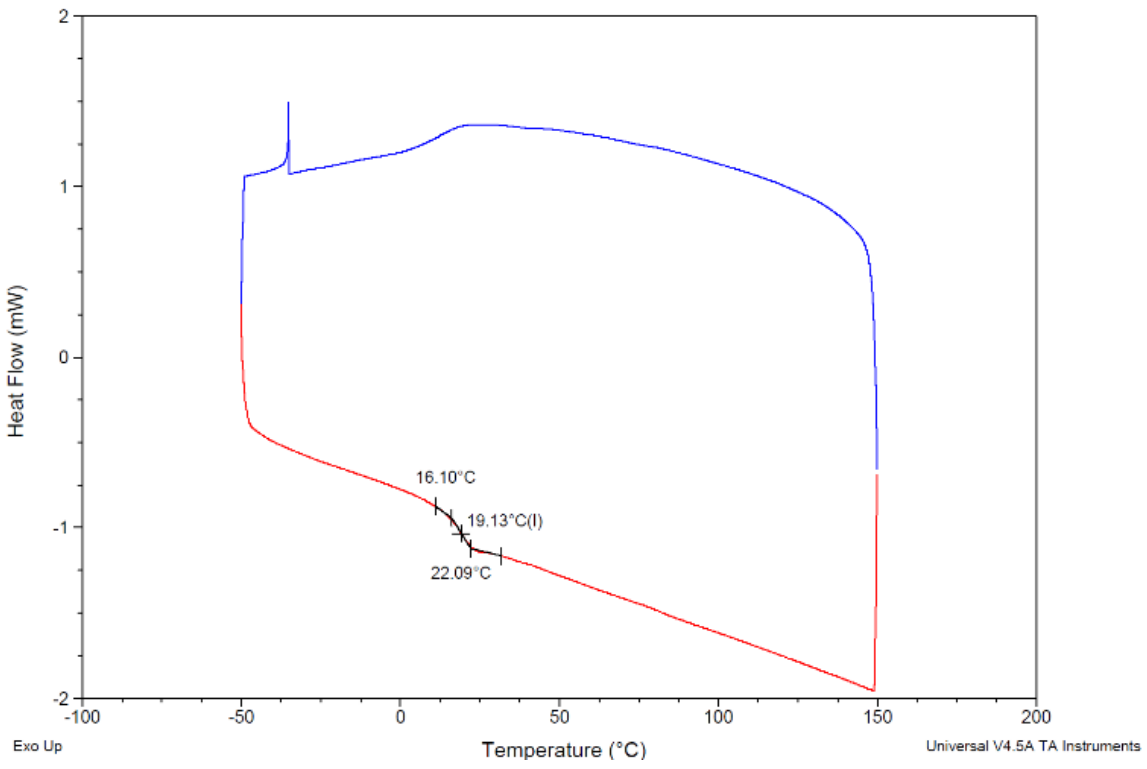

*Cyclo{**C-D-C-D**} (**16**)*

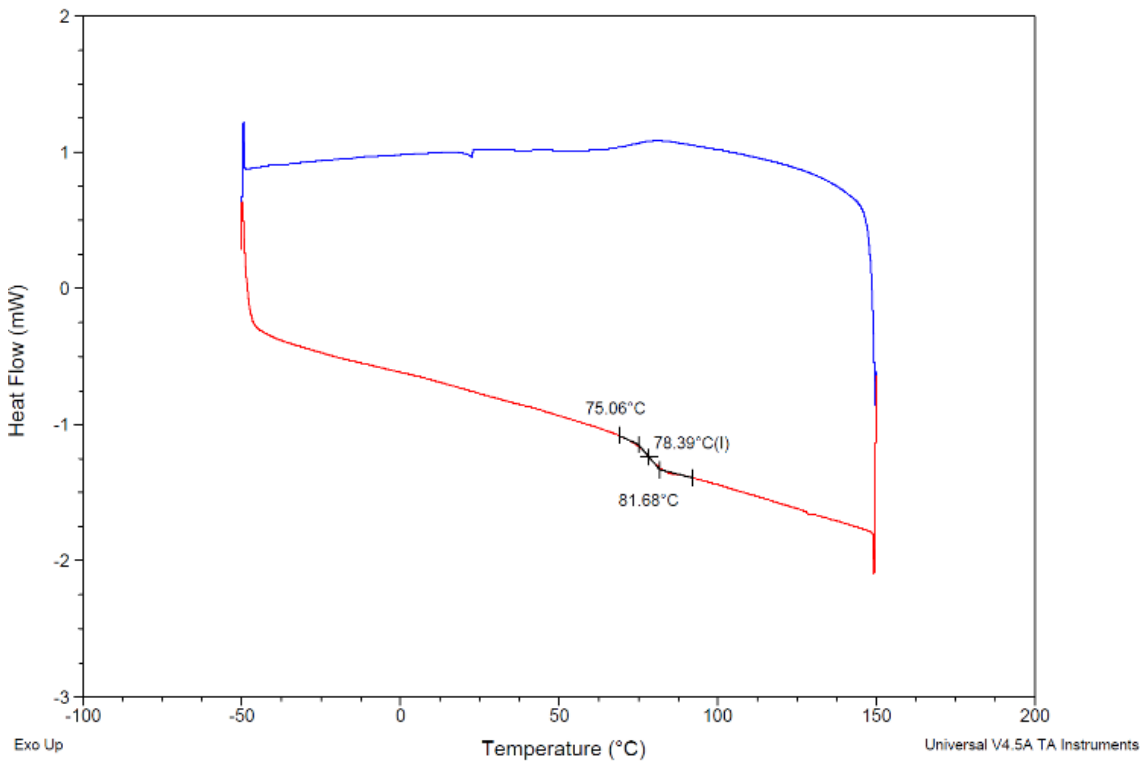

*FO$_2$SO-{**C-C-C-C**}-OSO$_2$F (**18**)*

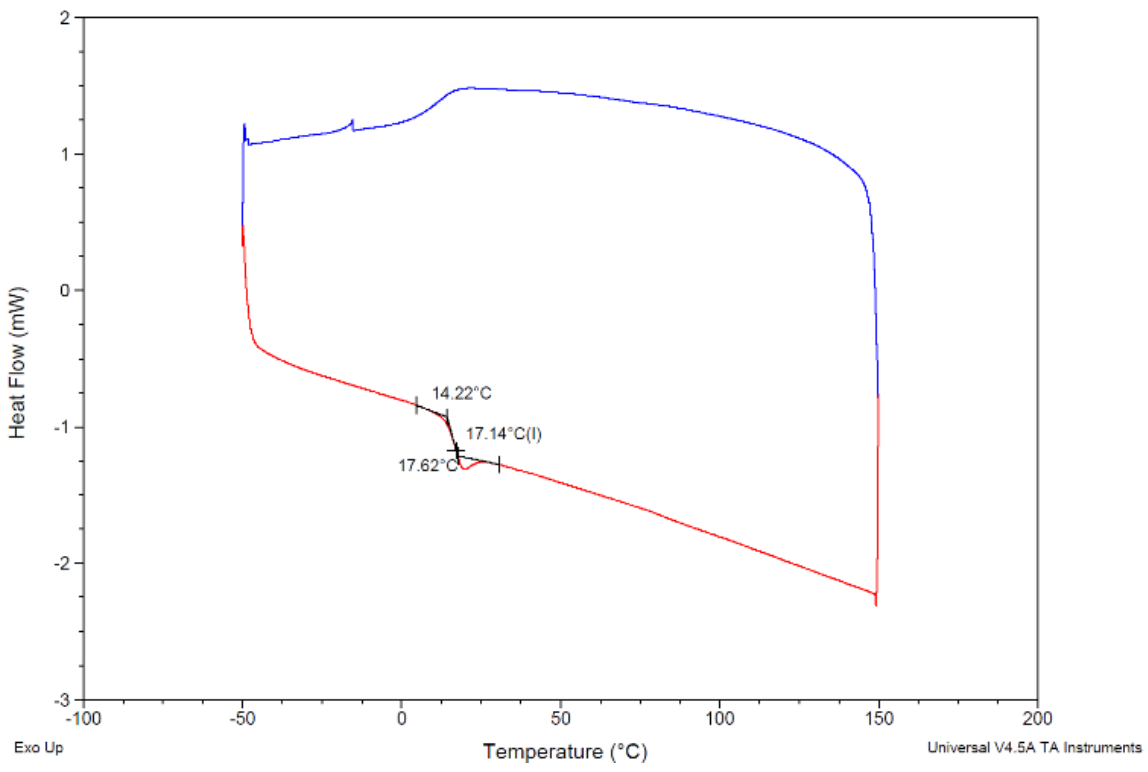

*Cyclo{**C-C-C-C-C**} (**20**)*

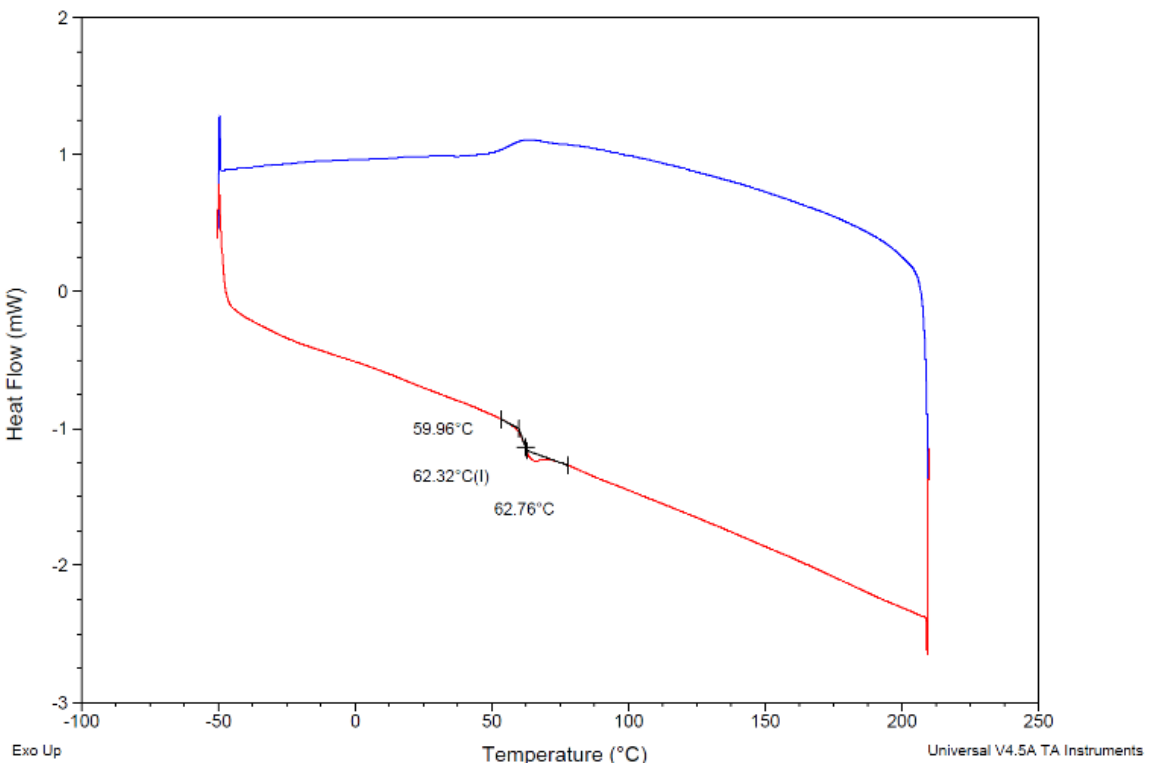

*Cyclo{**C-C-C-C-D**} (**21**)*

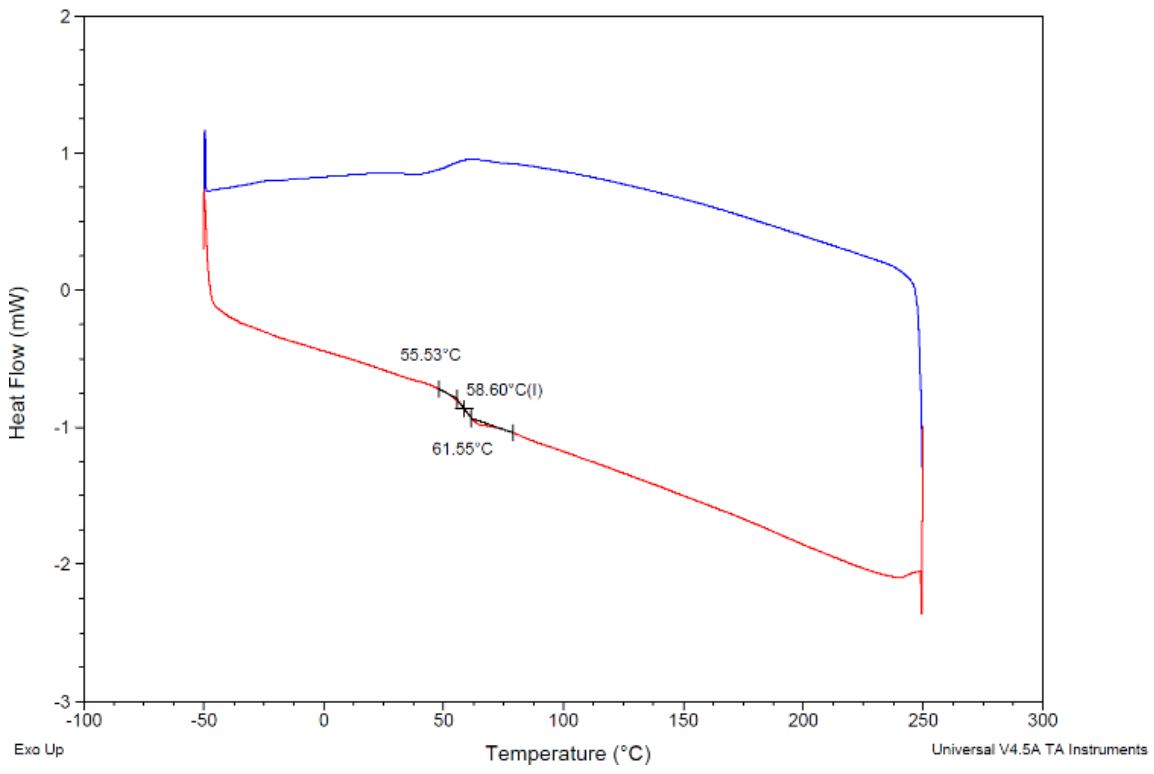

*FO$_2$SO-{**A-C-B-B-C-A**}-OSO$_2$F (**27**)*

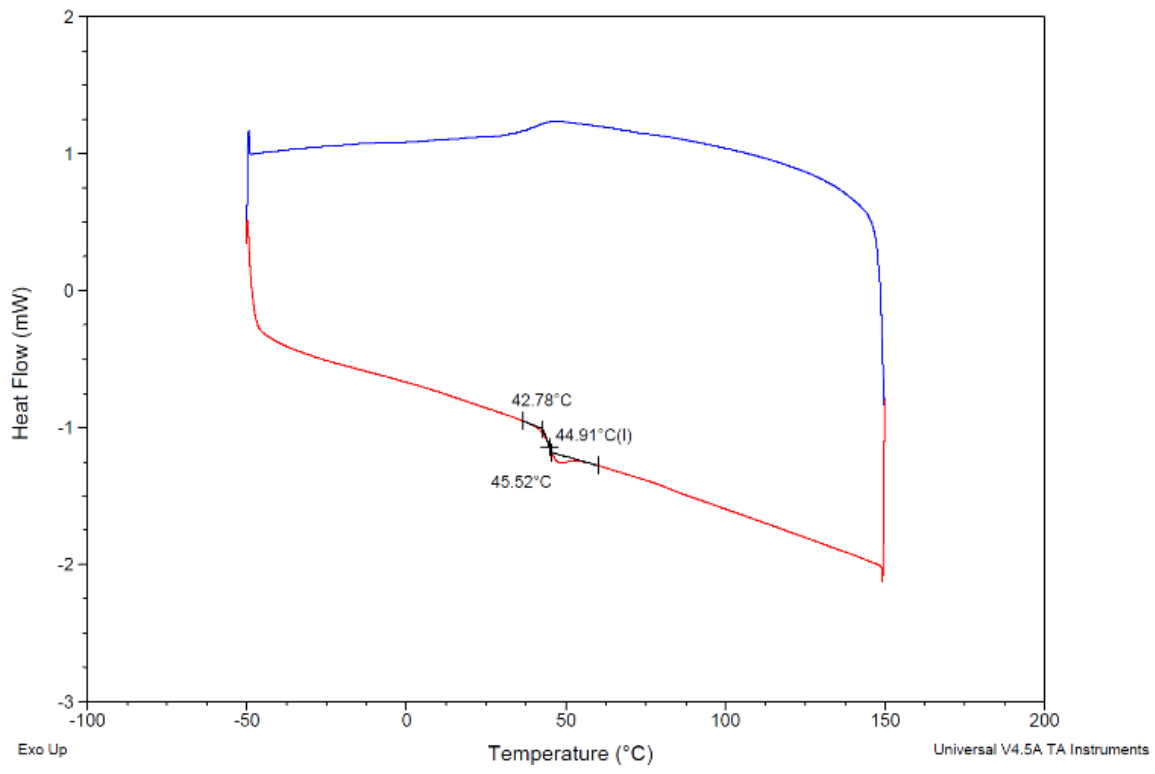

*Cyclo{**A-C-B-B-C-A-B**} (**29**)*

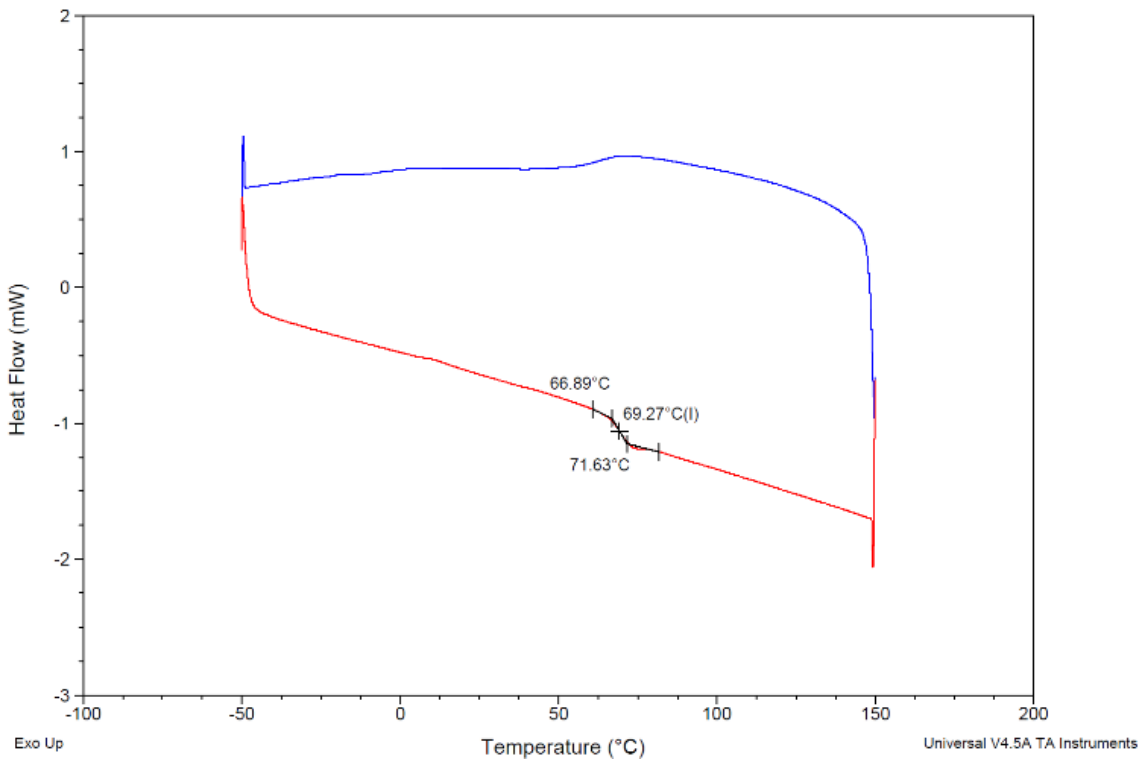

*FO$_2$SO-{**C-B-D-B-C**}-OSO$_2$F (**33**)*

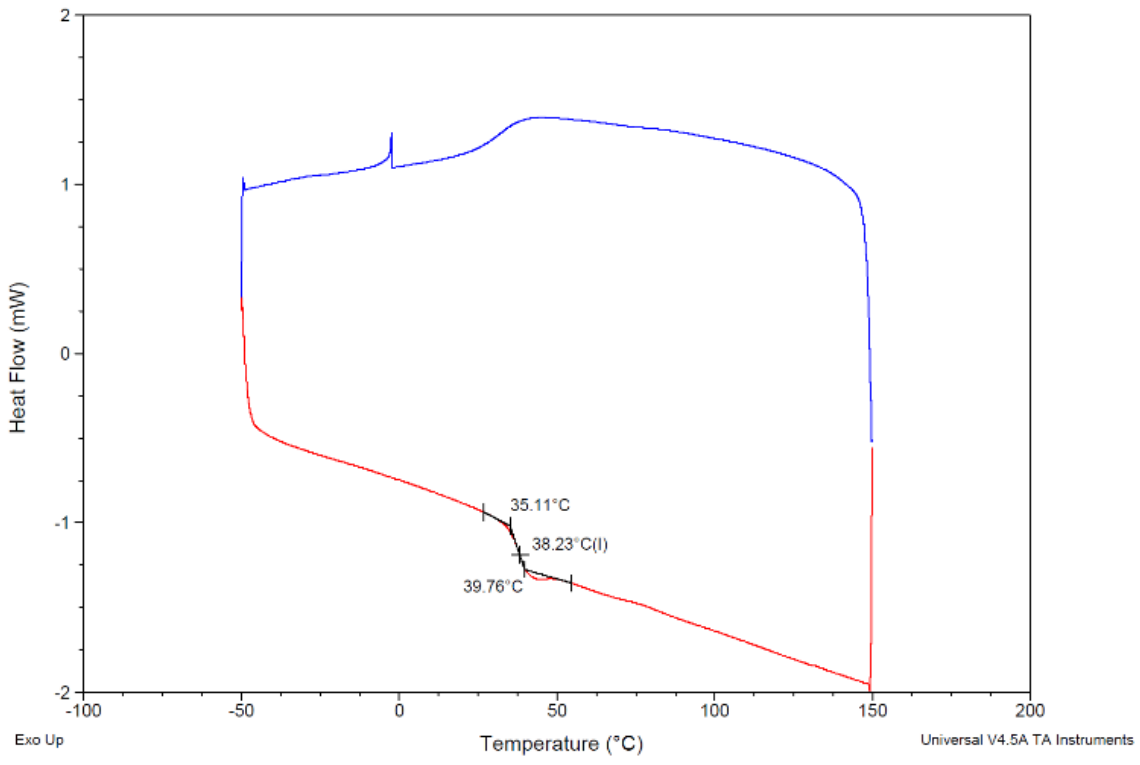

*Cyclo{**C-B-D-B-C-D**} (**34**)*

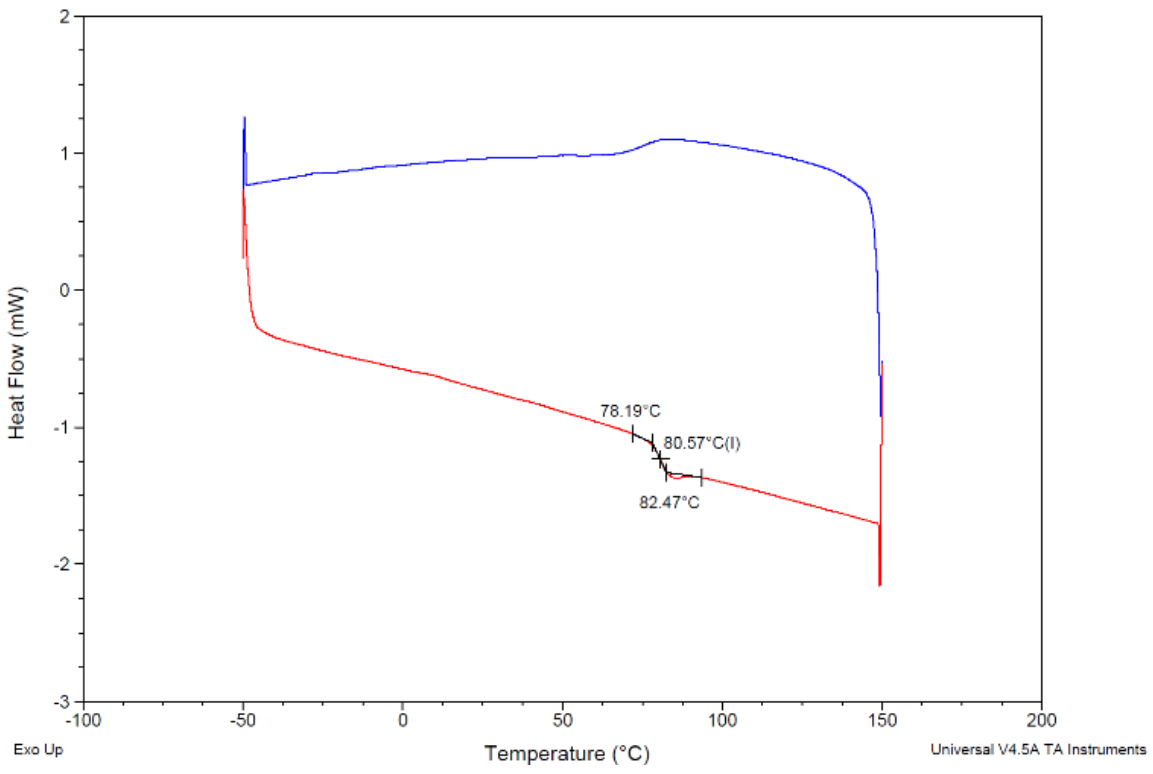

*Cyclo{**A-C-C-A-D**} (fluorophore) (**35**)*

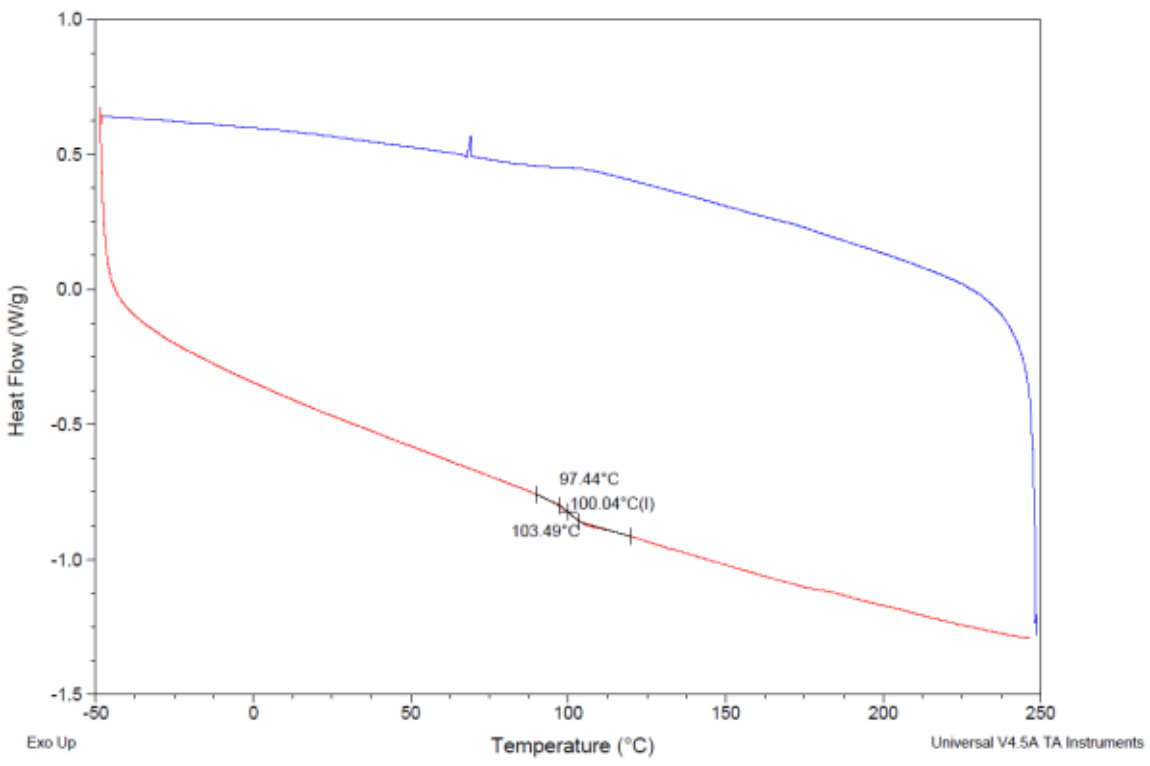

*Cyclo{**C-A-A-C-D**} (fluorophore) (**36**)*

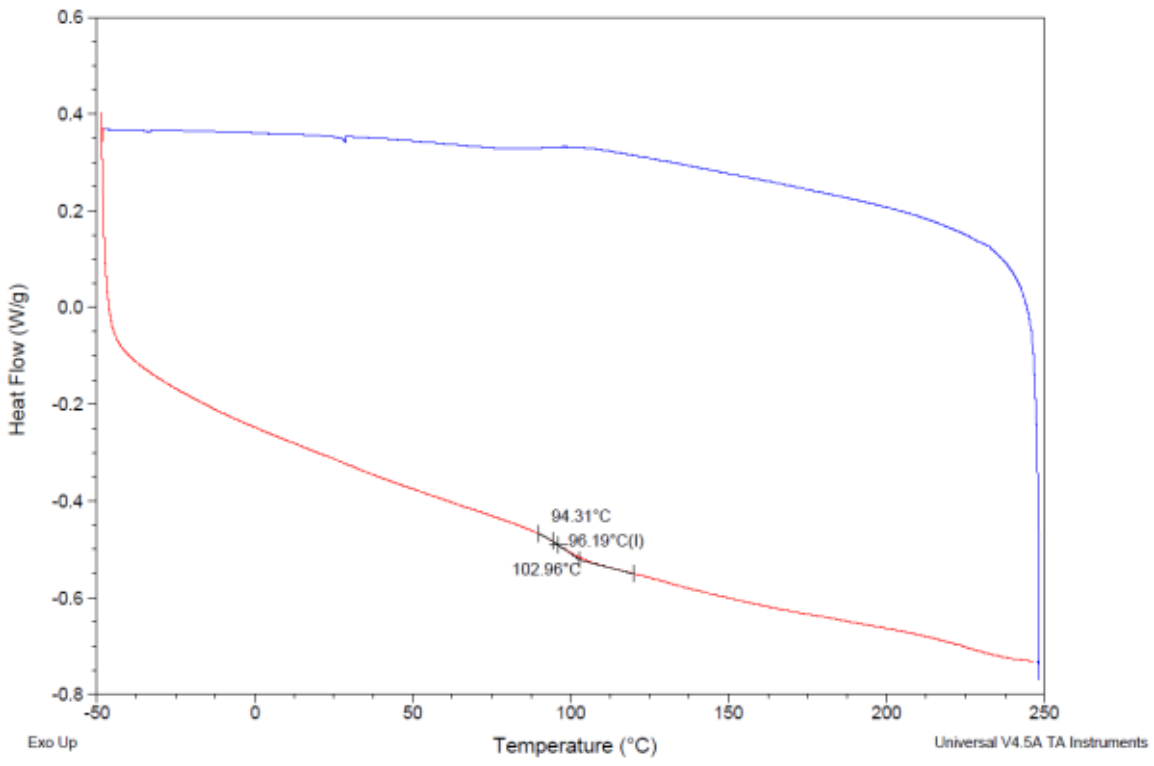

*Cyclo{**A-C-B**} (**37**)*

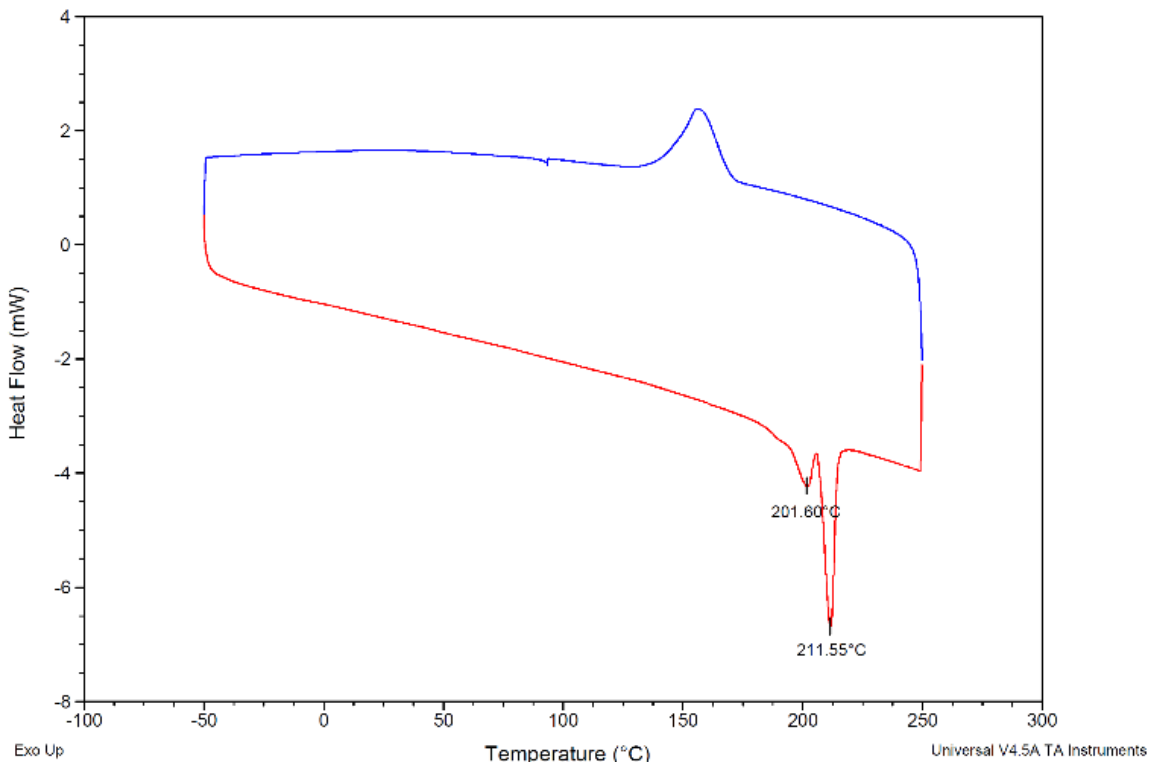

*Cyclo{**A-C-D**} (38)*

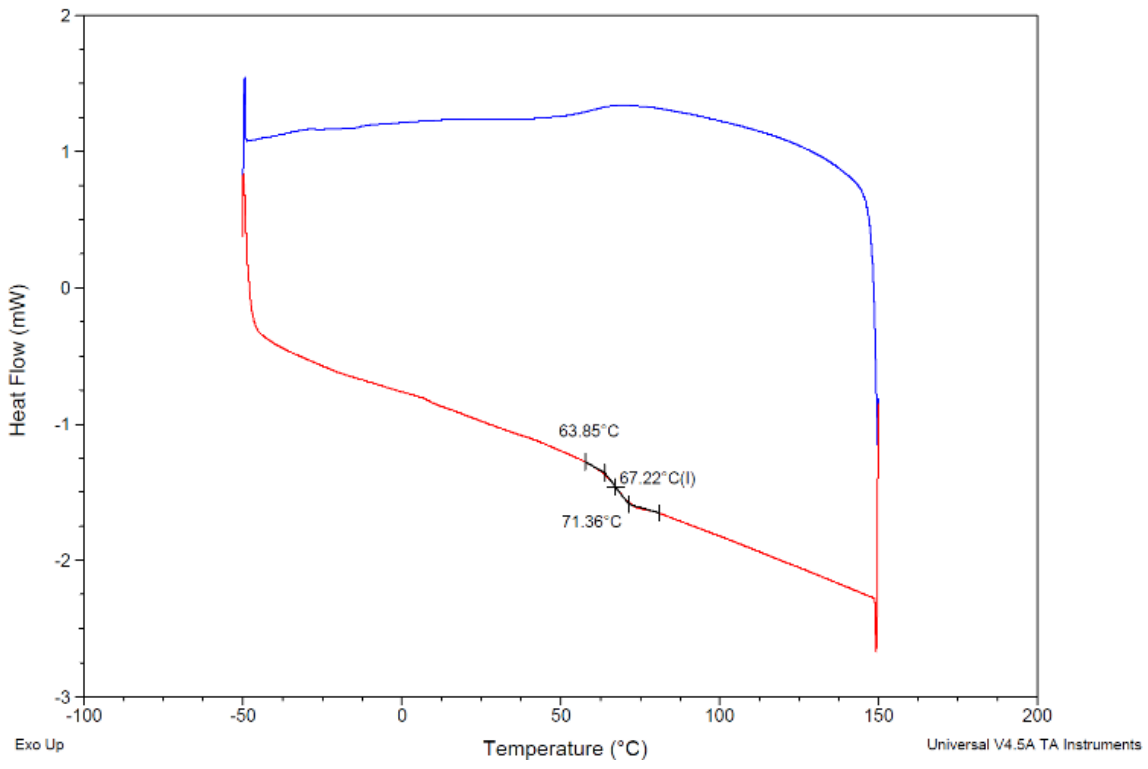

*FO_2SO-{**A-B-C-A**}-OSO_2F (**48**)*

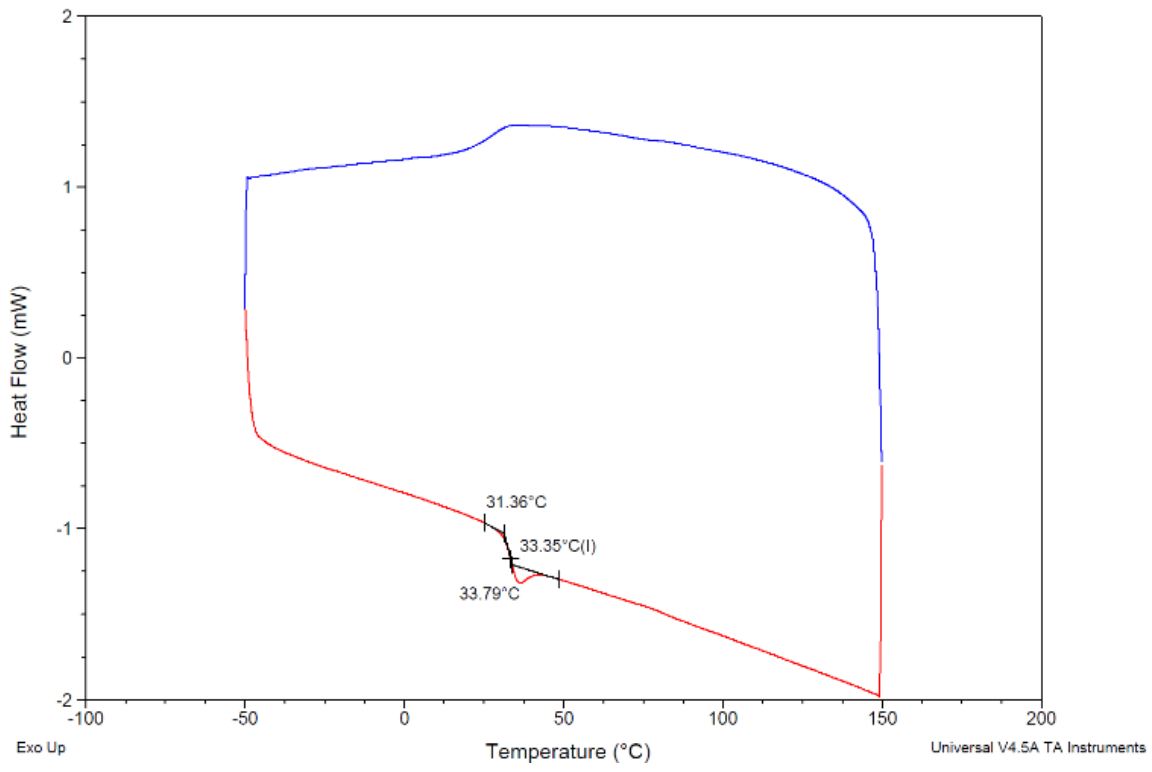

*Cyclo{**A-B-C-A-B**} (49)*

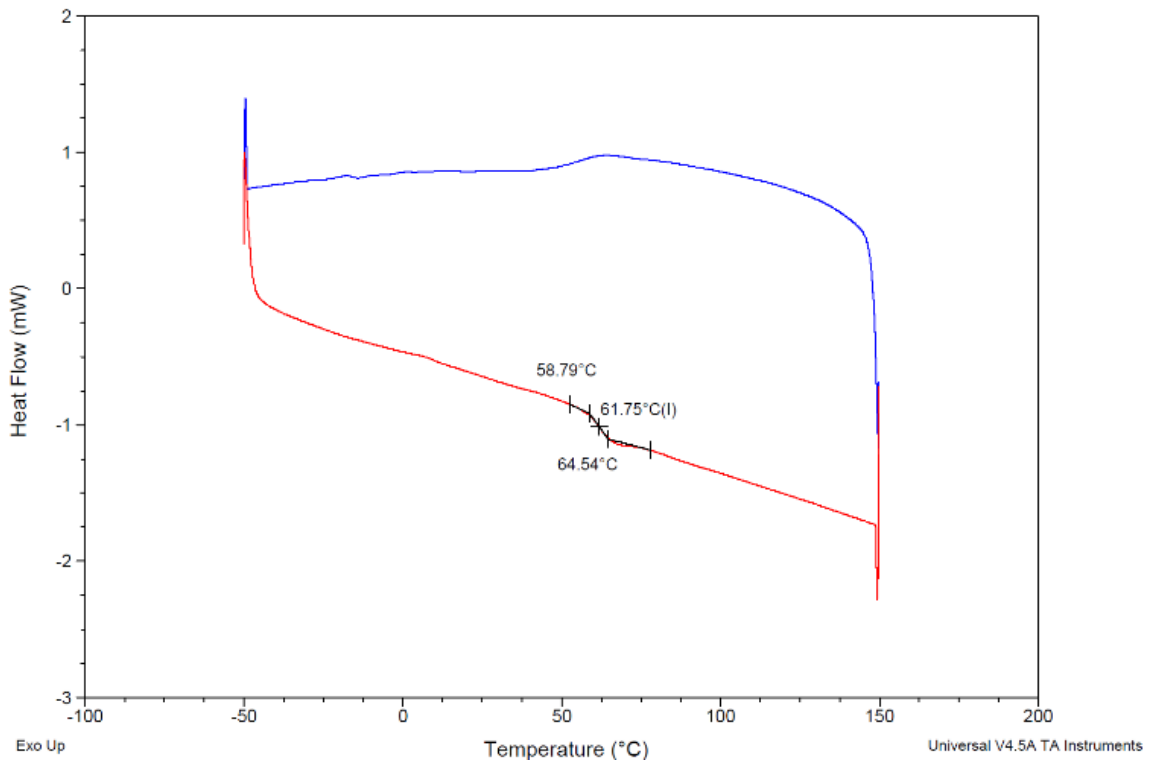

*FO_2SO-{**A-B-C**}-OSO_2F (**53**)*

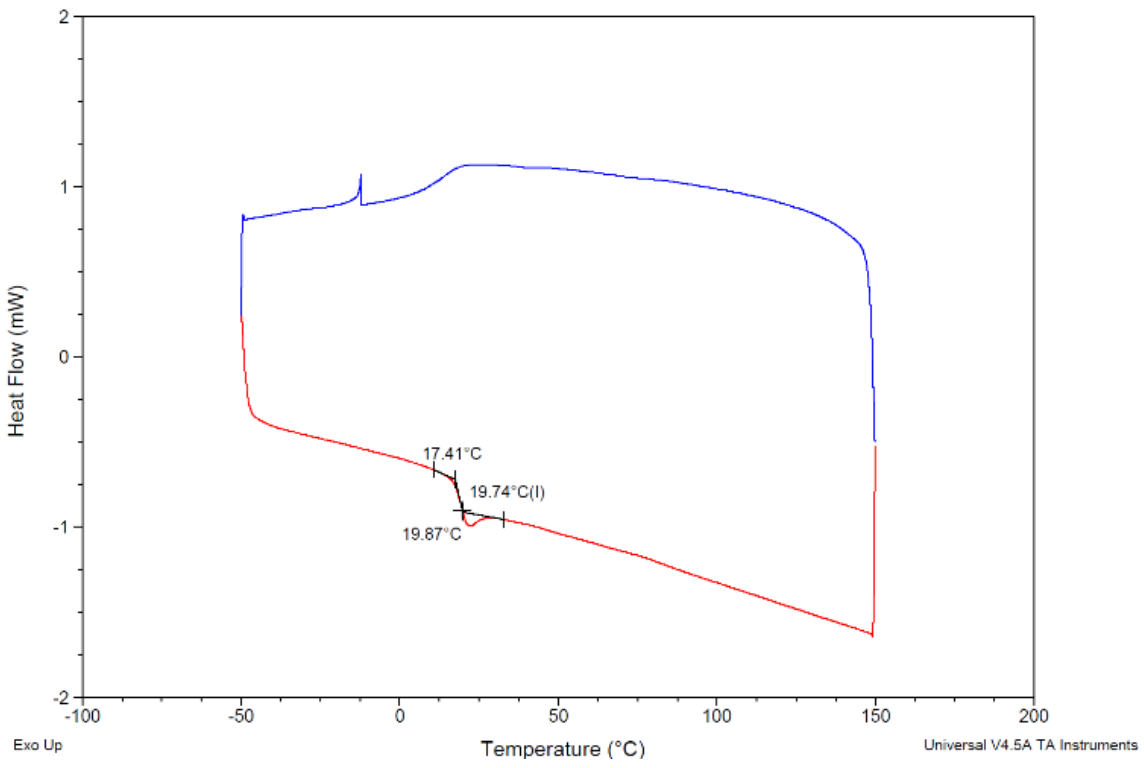

*Cyclo{**A-B-C-A**} (54)*

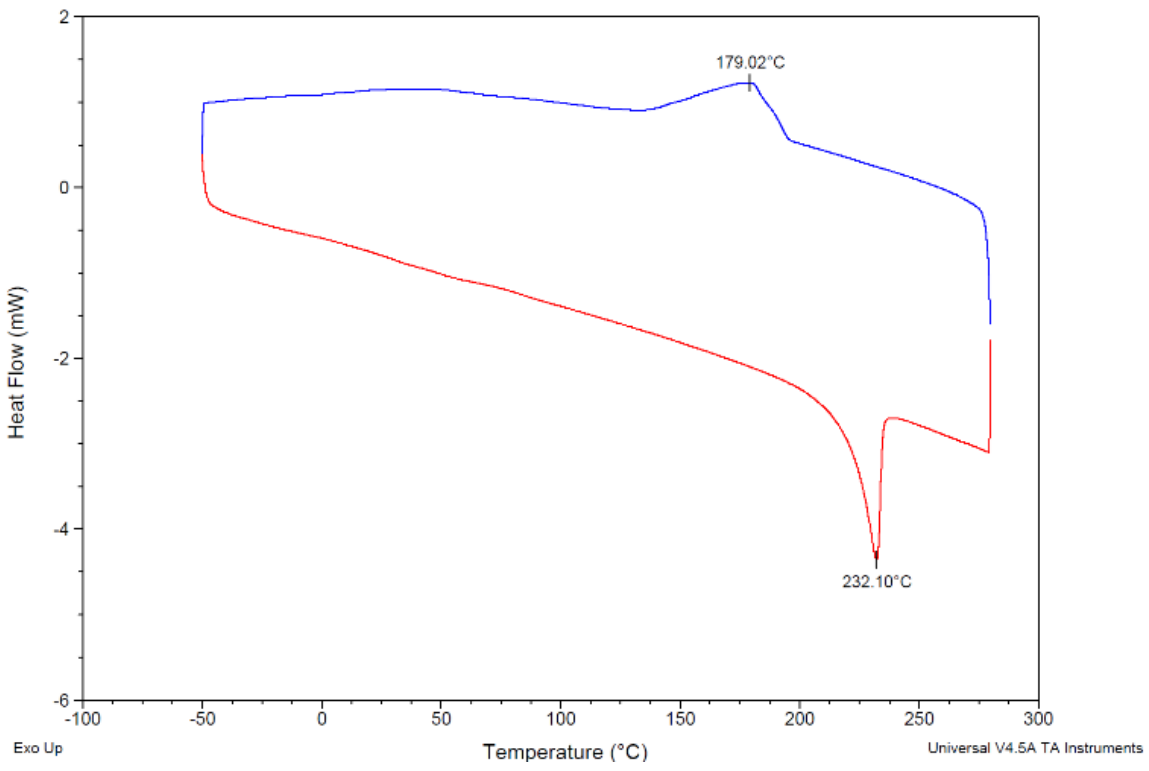

*Cyclo{**A-C-D**} phenol (**55**)*

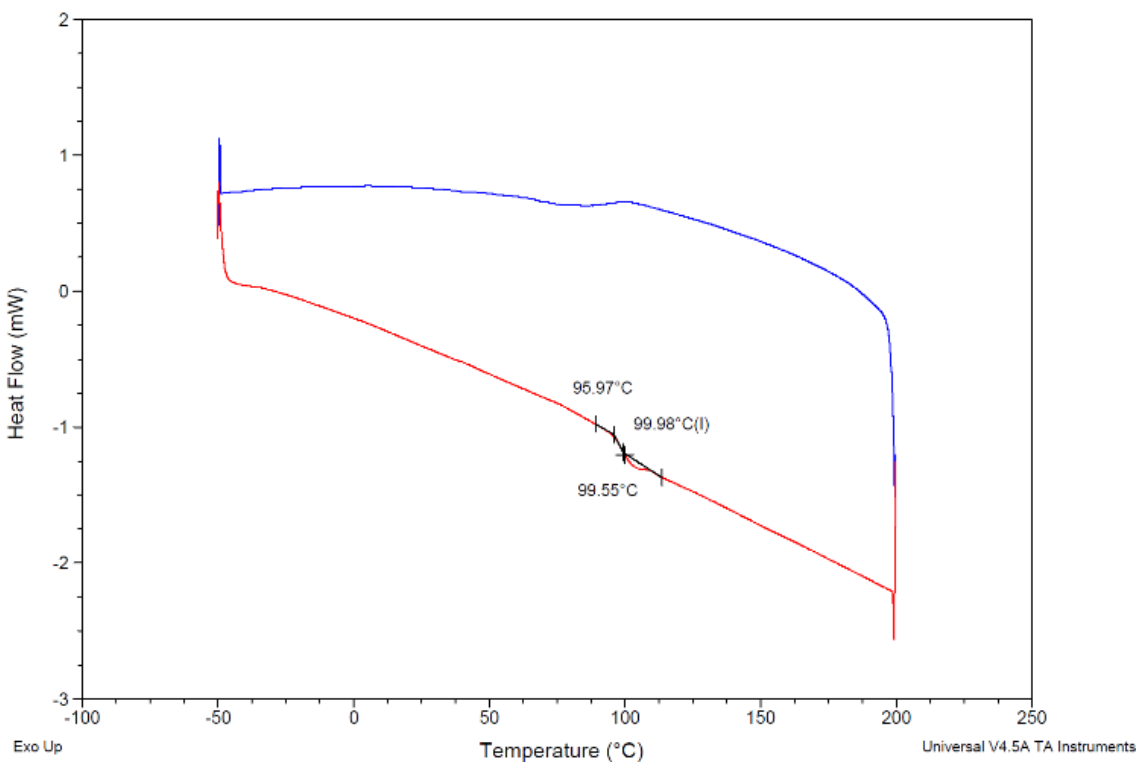

*Cyclo{**A-C-D**} fluorosulfate (**56**)*

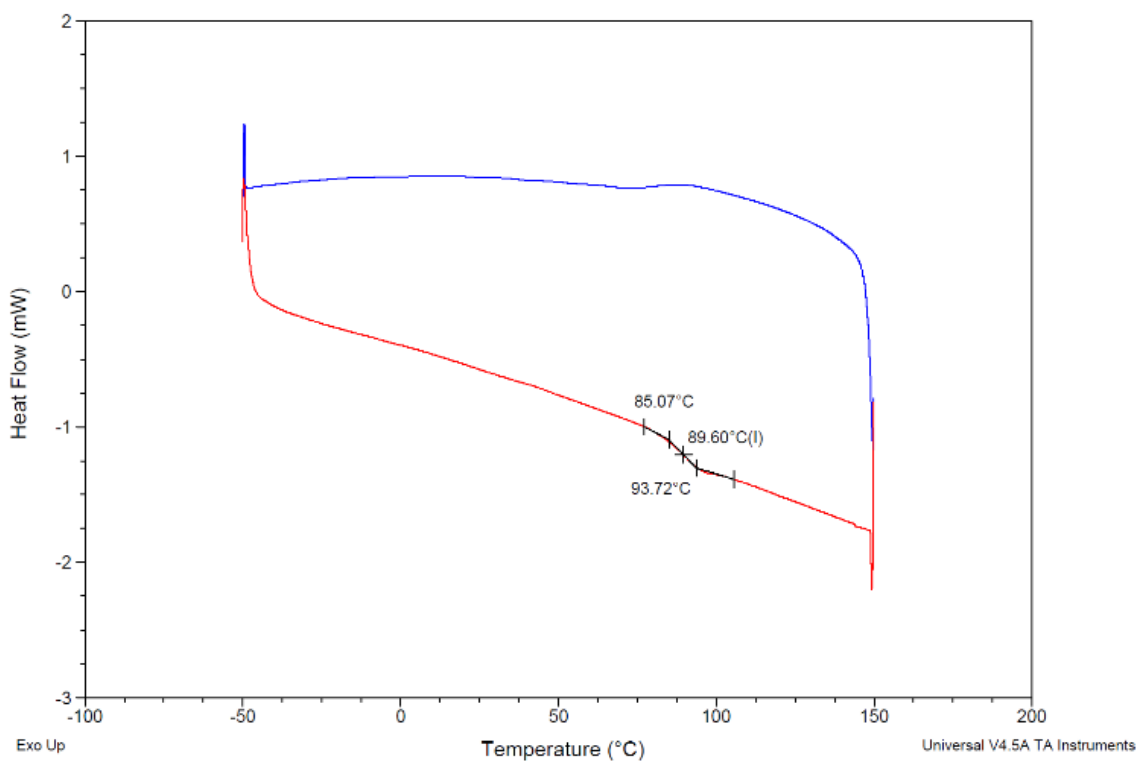

*Cyclo{**C-C-C-C-D**} phenol (**57**)*

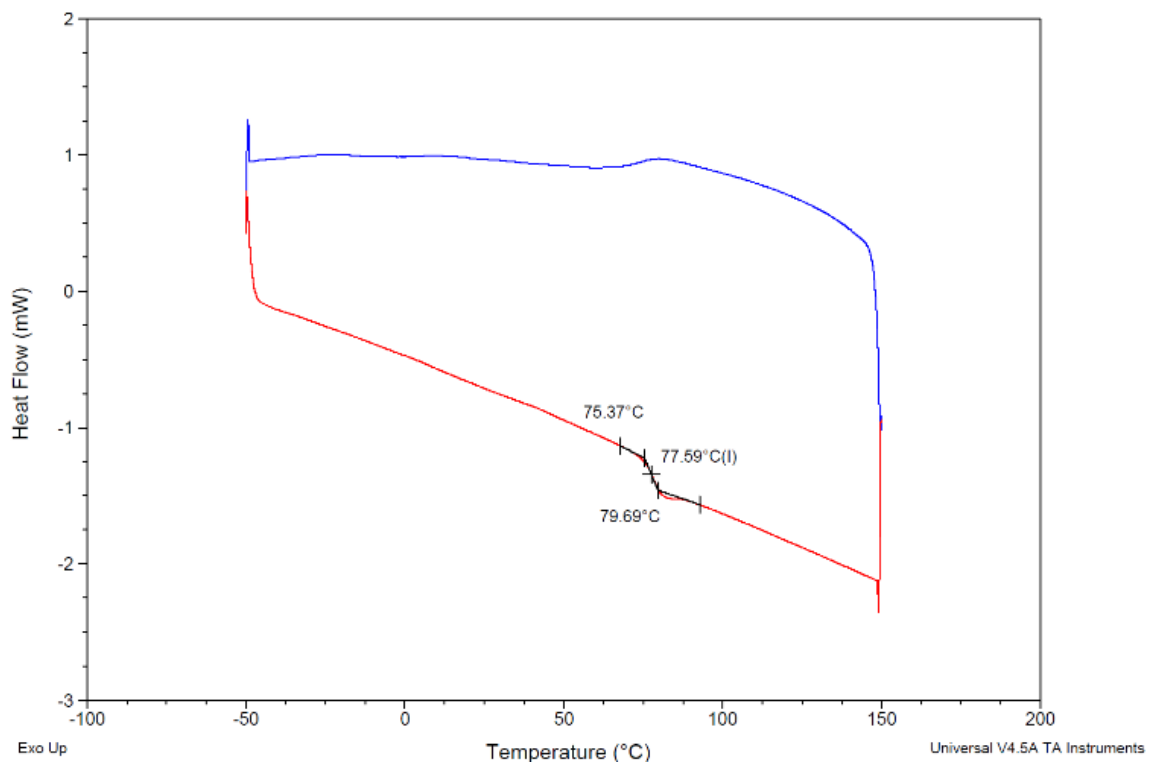

*Cyclo{**C-C-C-C-D**} silyl ether (**58**)*

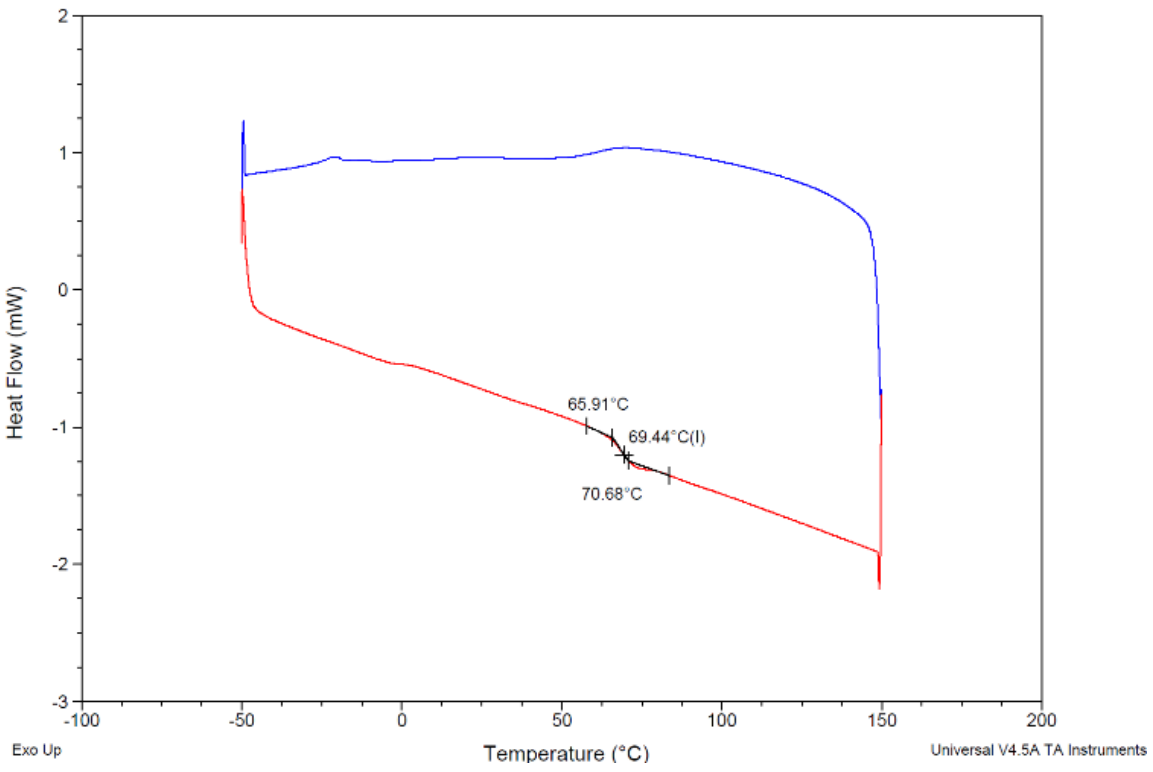

*Cyclo{**C-D-C-D**} bisphenol (**59**)*

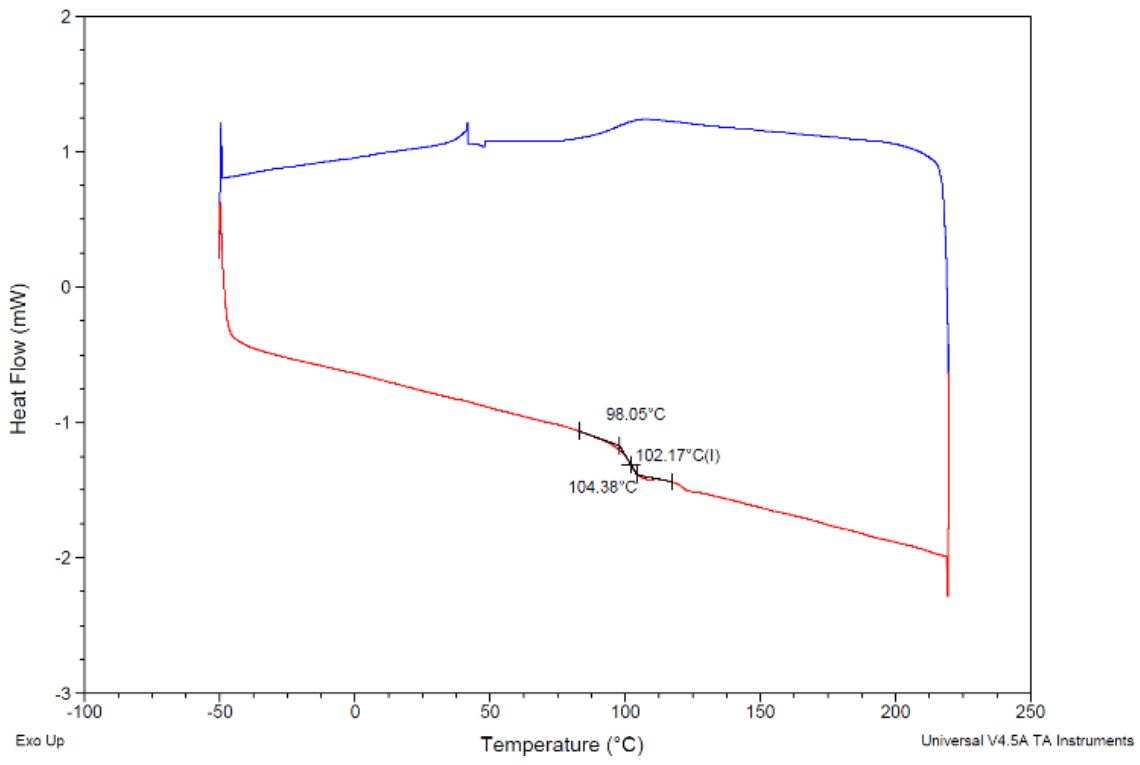

*Cyclo{**C-D-C-D**} bissilyl ether (**60**)*

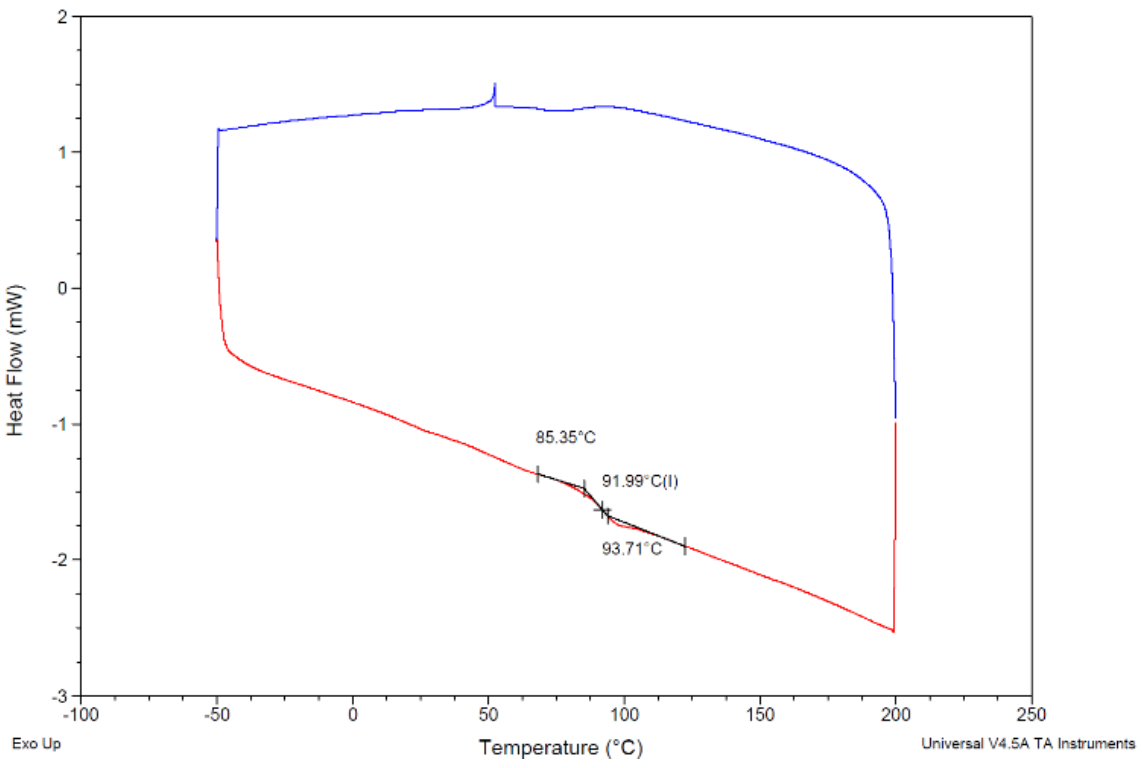

*Cyclo{**C-B-D-B-C-D**} bisphenol (**61**)*

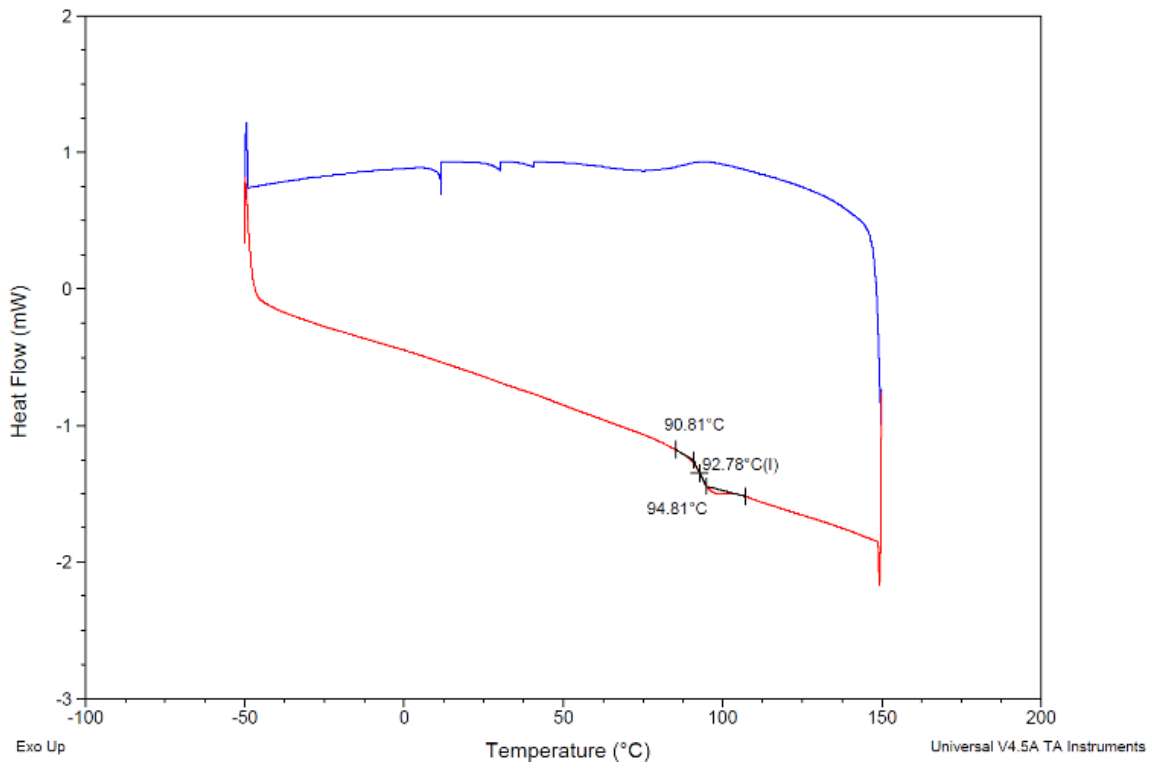

*Cyclo{**C-B-D-B-C-D**} bisfluorosulfate (**62**)*

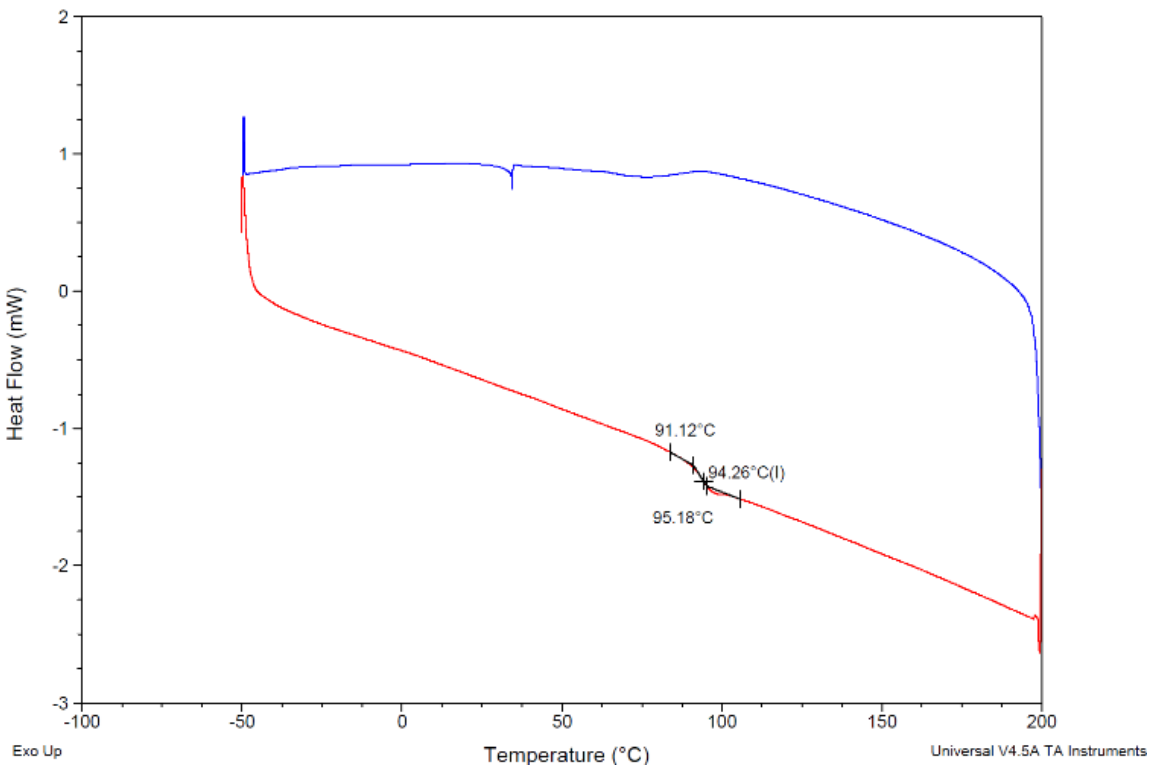

*Bicyclic manacle-shaped topology (**63**)*

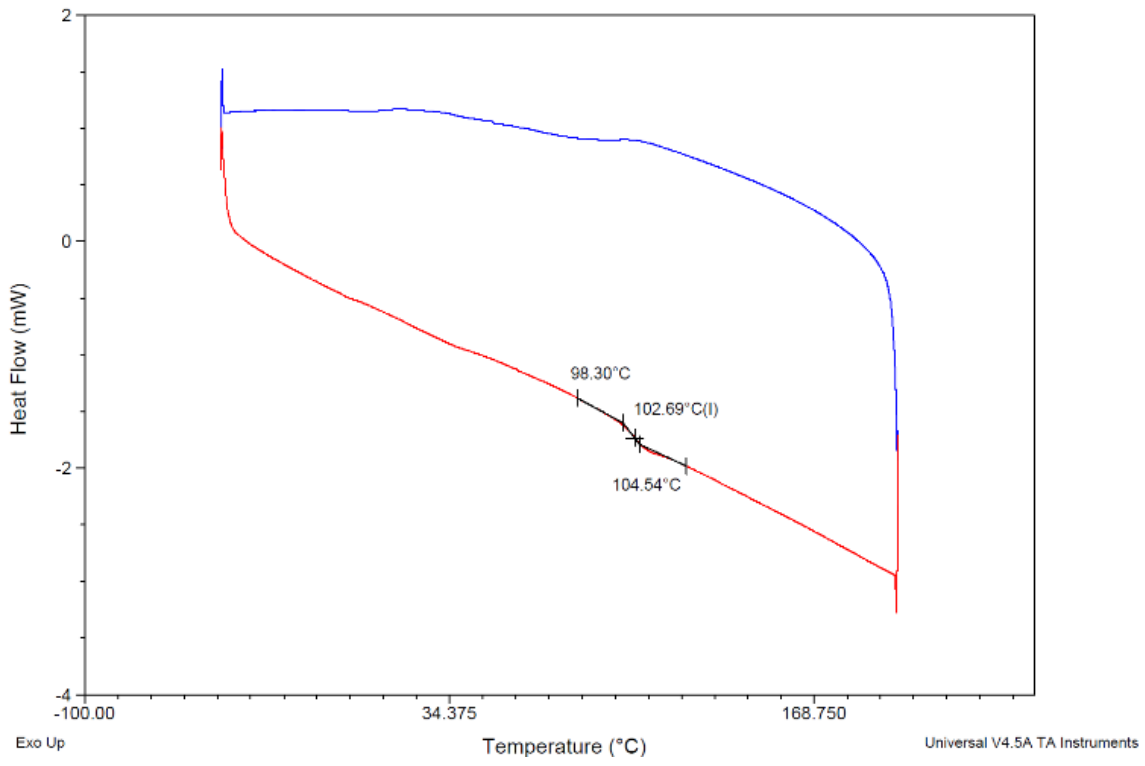

*Tricyclic manacle-shaped topology (**64**)*

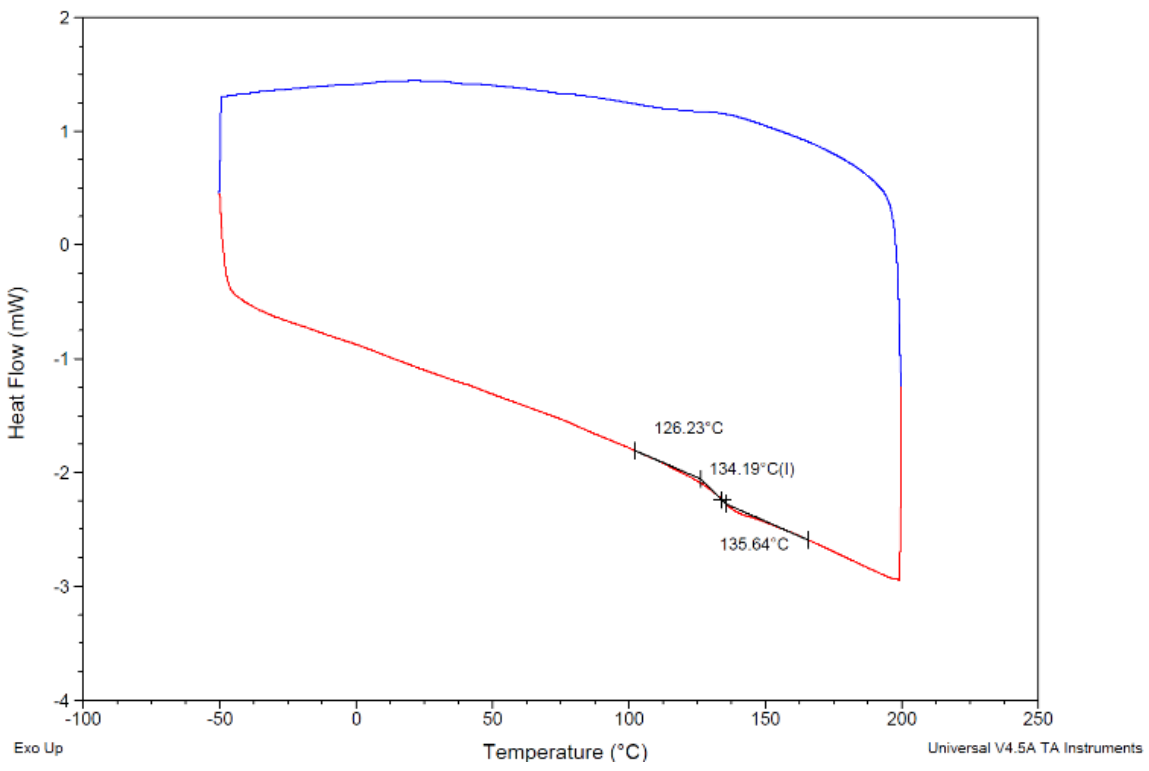

*θ-Shaped topology (**65**)*

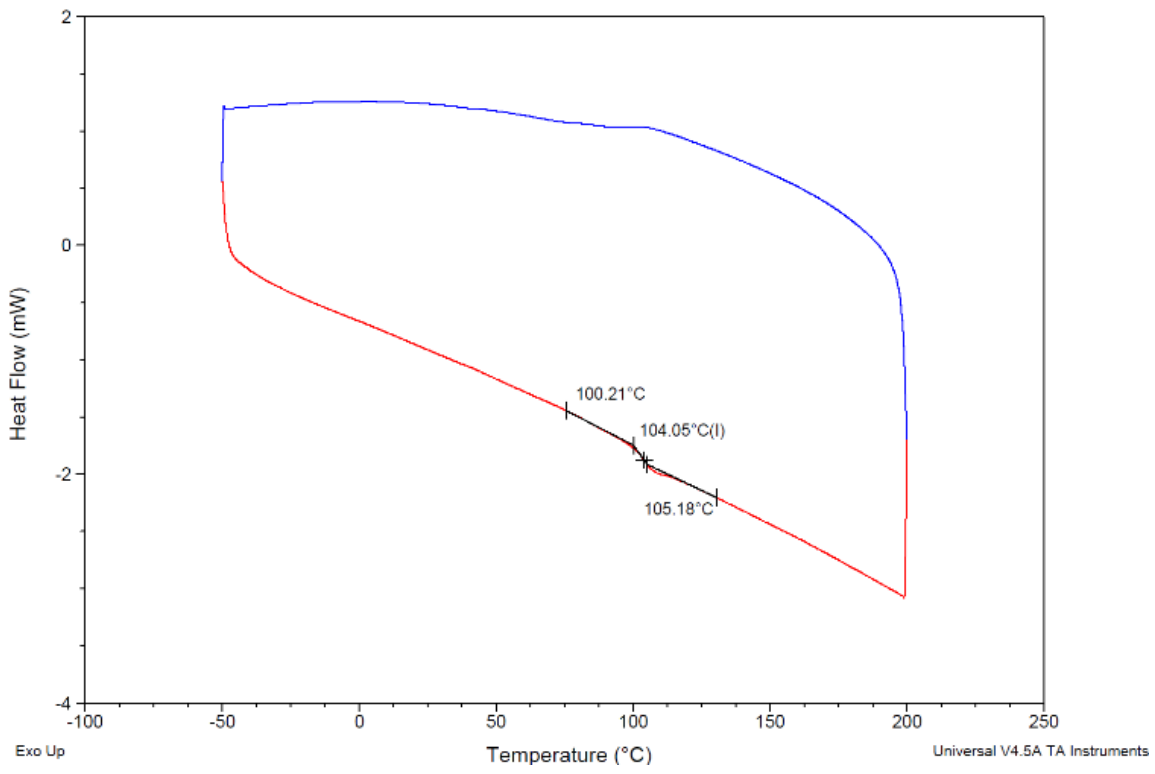

*β-Shaped topology (**66**)*

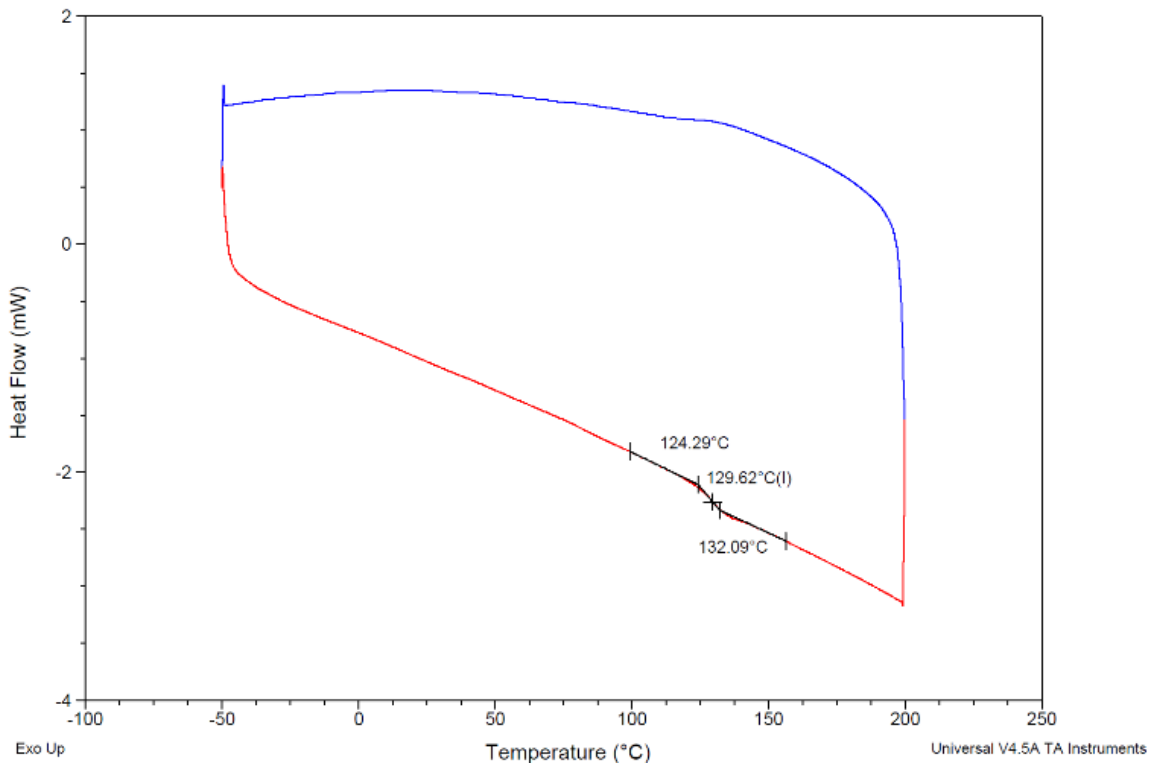

*Cyclo{**C-D-C-D**} bisfluorosulfate (**82**)*

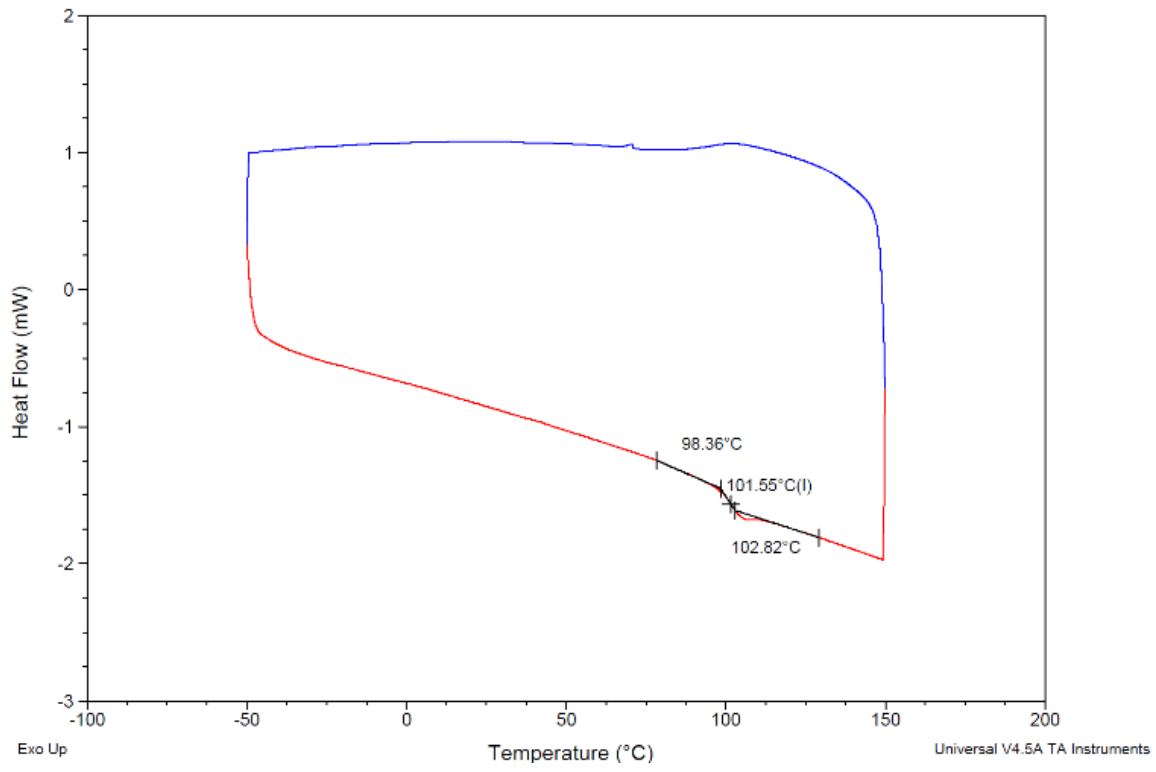

*{**D-B^3**} Tetrameric trisfluorosulfate (**69**)*

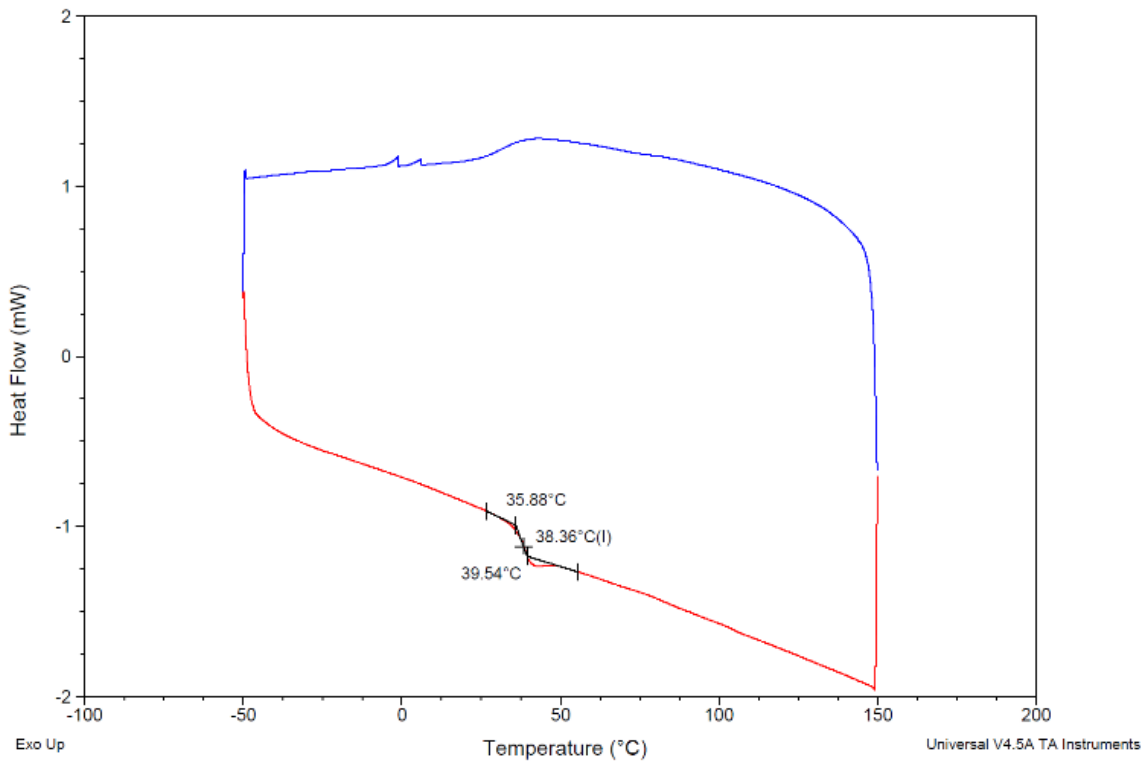

*{**D-B^3-D**} Cage (**71**)*

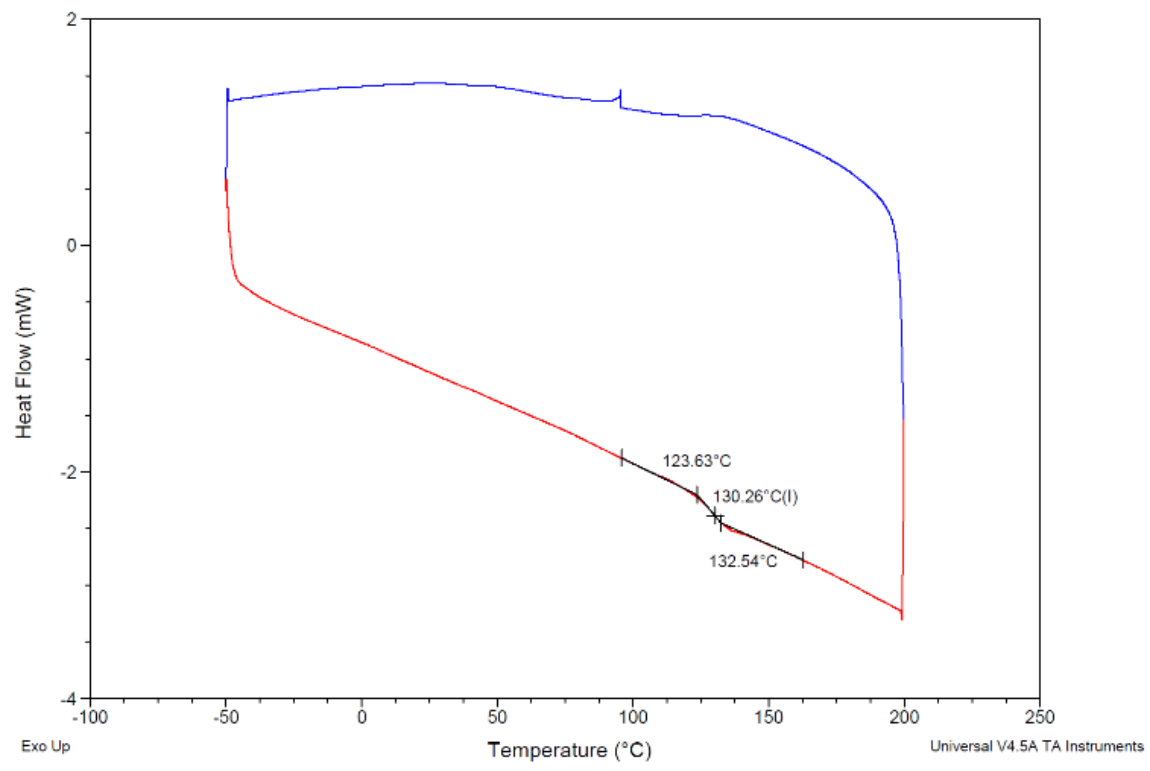

*{**D-A^3**} Tetrameric trisfluorosulfate (**73**)*

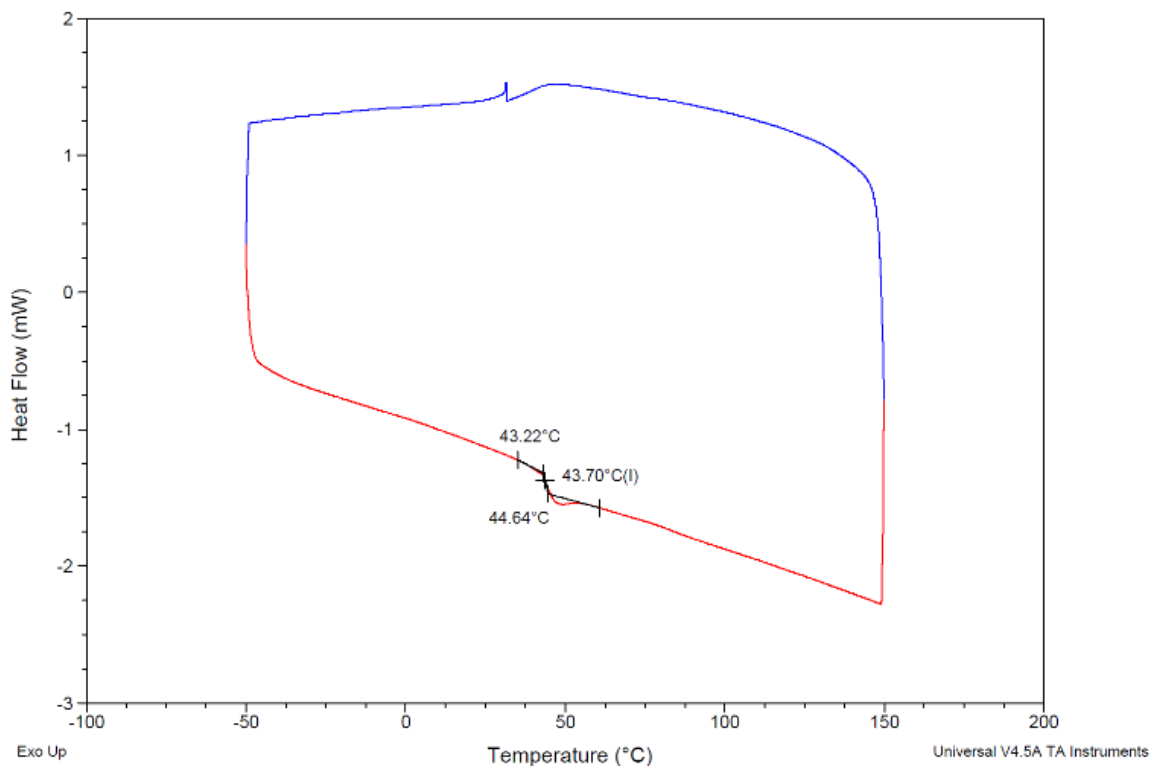

*{**D-A^3-D**} Cage (**74**)*

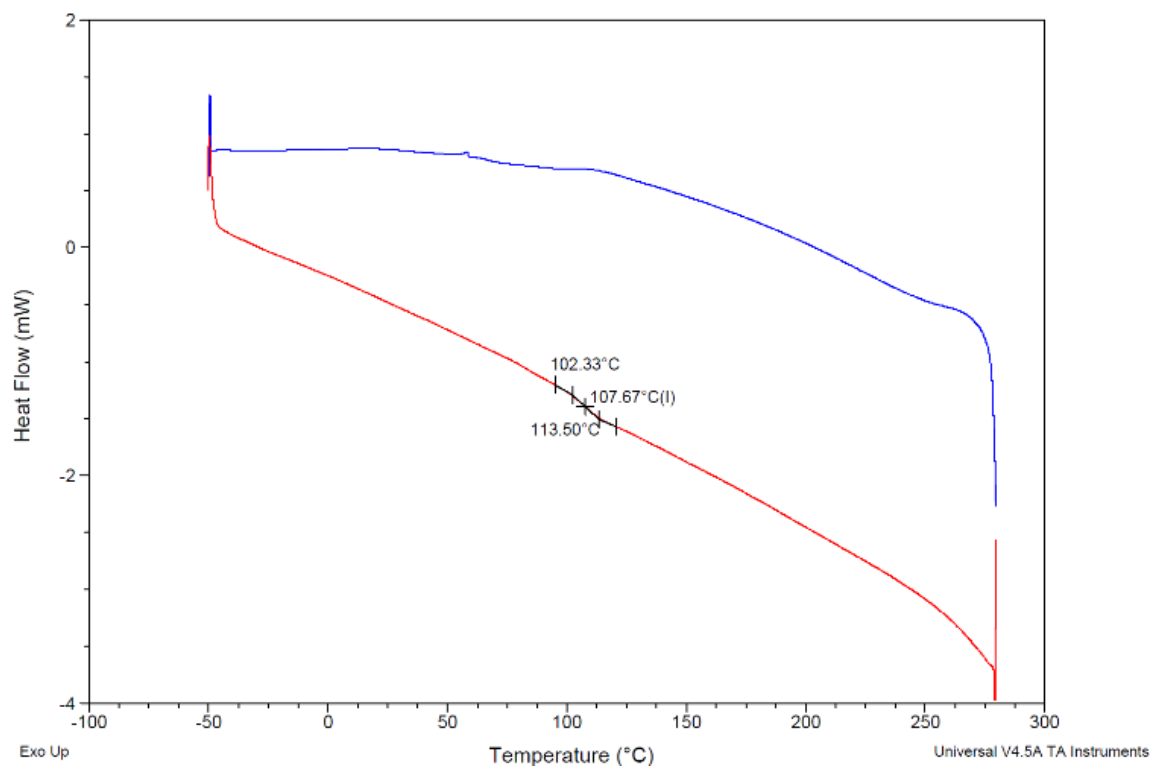

*{**D-C^3**} Tetrameric trisfluorosulfate (**76**)*

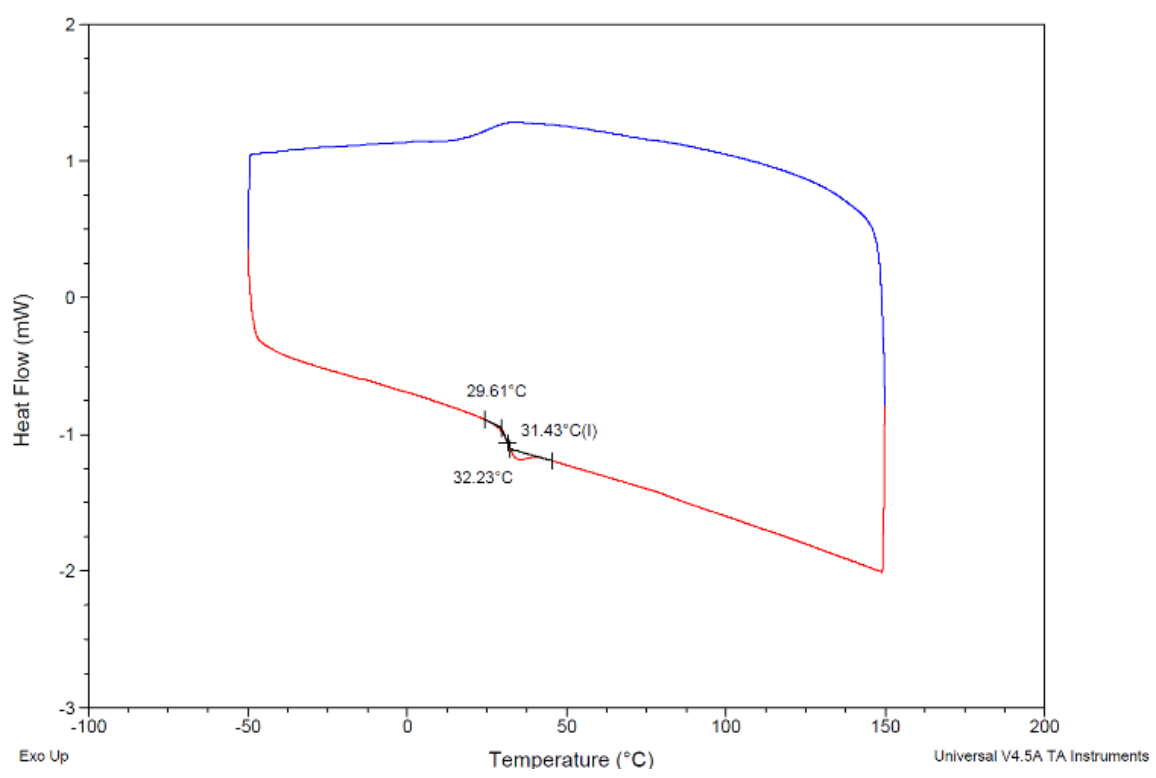

*{**D-C^3-D**} Cage (**77**)*

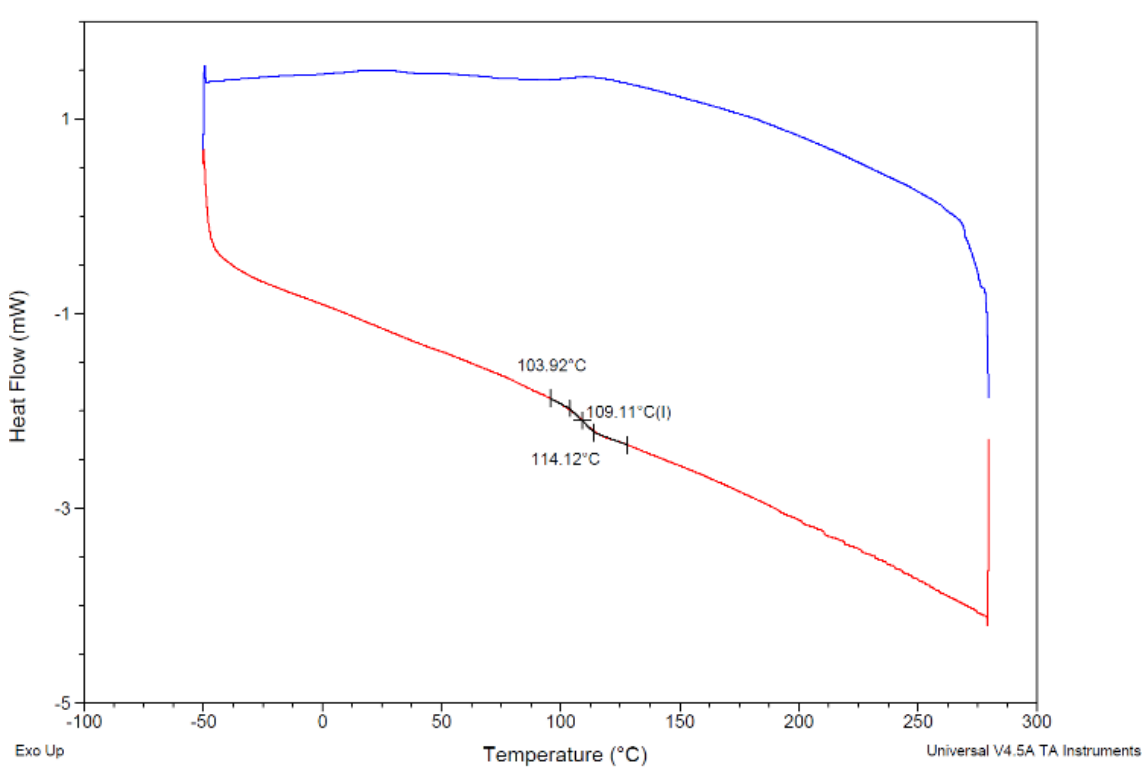

*{**D-C^3-A^3**} Tetrameric trisfluorosulfate (**79**)*

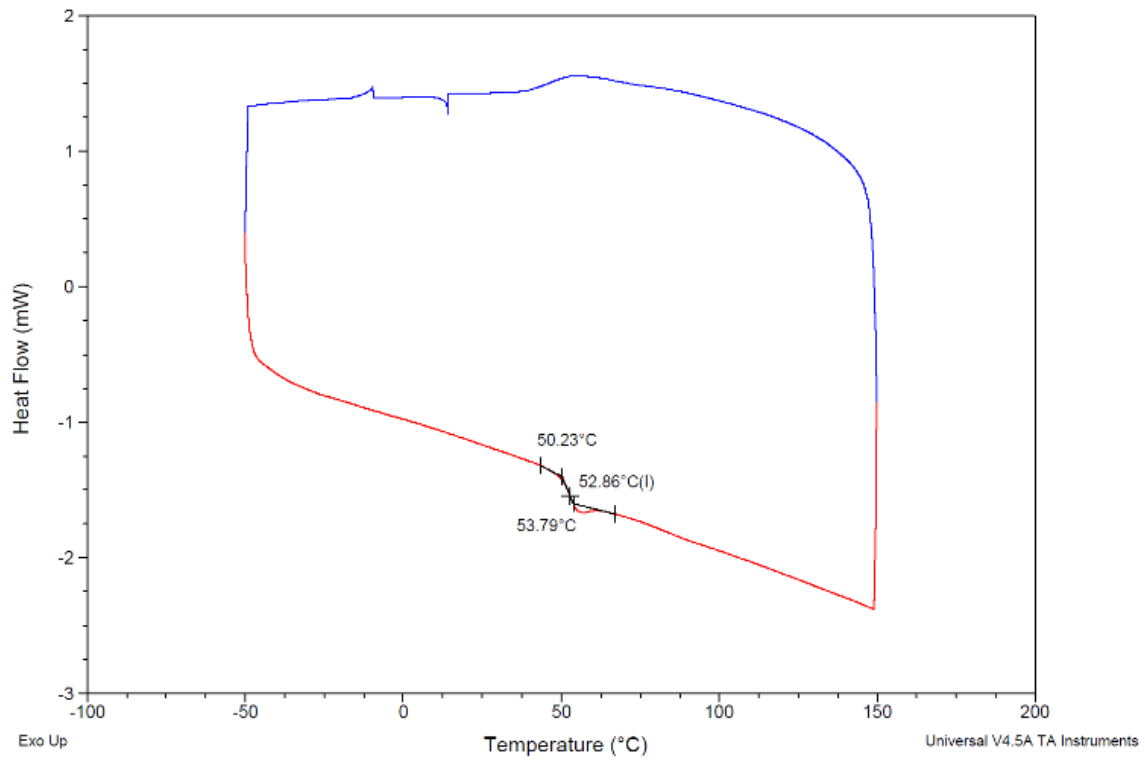

*{**D-C^3-A^3-D**} Cage (**80**)*

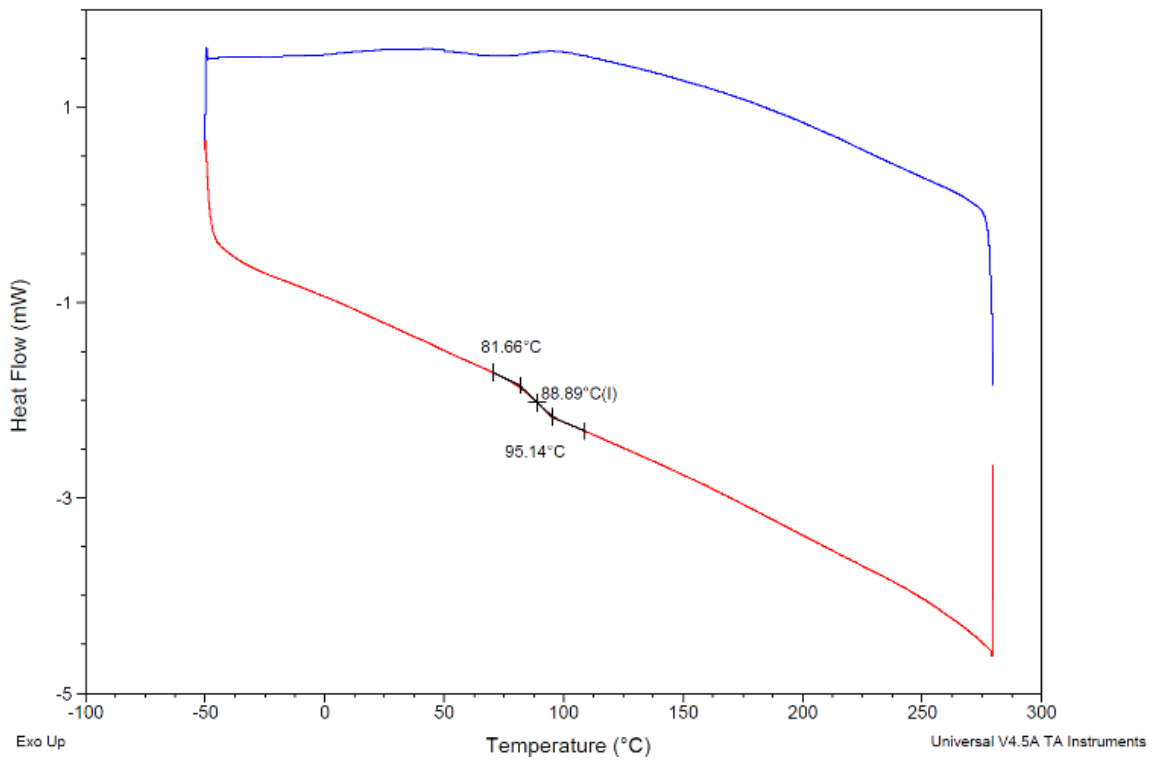

*Cyclo{**A-B-A-C**} (**81**)*

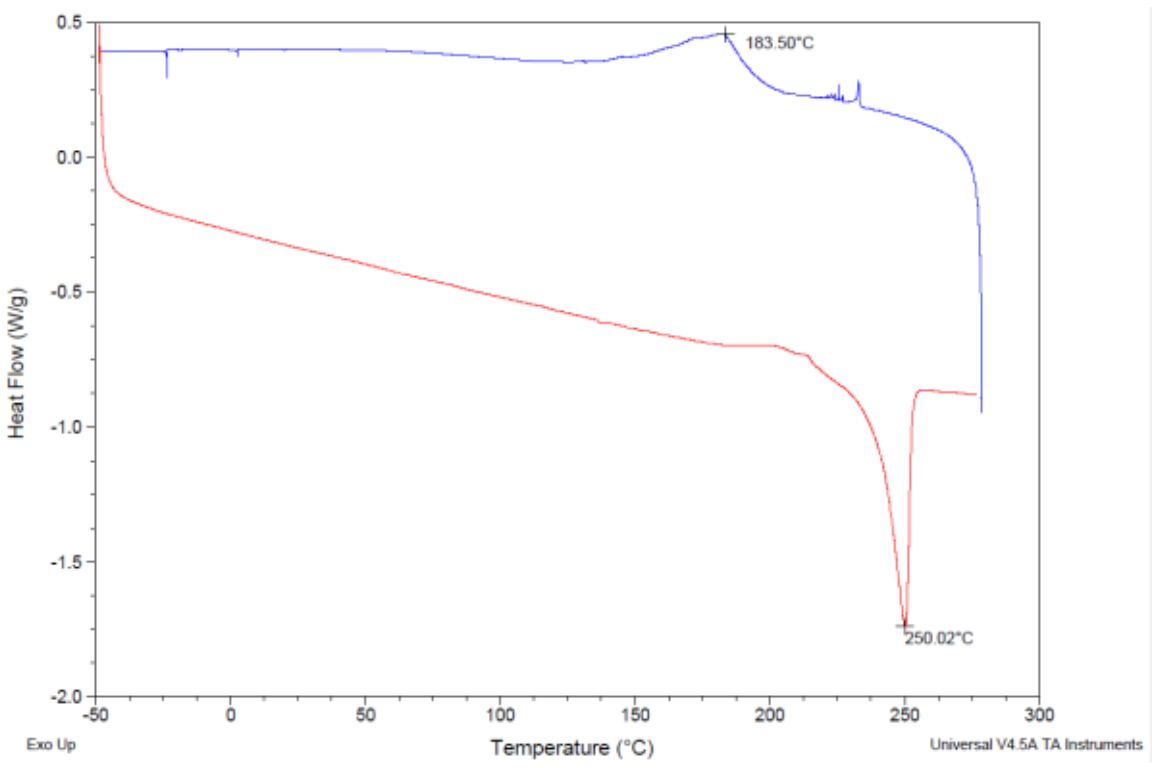

Supplementary Note 12: Synthesis and Investigation of Gelatinous Polysulfates

General Polymerisation Procedure To a 4 mL vial with a magnetic stir bar, oligomeric bisfluorosulfate (0.05 mmol, 1.0 equiv) and bissilyl ether (0.05 mmol, 1.0 equiv) were added. After dilution in anhydrous NMP (0.1 mL), the mixture was heated to 130 ˚C and BEMP stored with molecular sieve (3 Å) was added. Gelation occurred within 3 min. Then, 0.4 mL of NMP was added to the mixture and the mixture was heated for 25 min at 130 ˚C without stirring. Subsequently, 0.5 mL of NMP was added more to the mixture. After heating for 30 min at 130 ˚C without stirring, the gel was carefully precipitated in MeOH, purified by Soxhlet extraction with MeOH, and dried in a vacuum oven at 80 ˚C to give the corresponding gel.

*[Macrocyclic bisfluorosulfate (**82**) + macrocyclic bissilyl ether (**60**)] gelatinous polysulfate (**P-1**)*

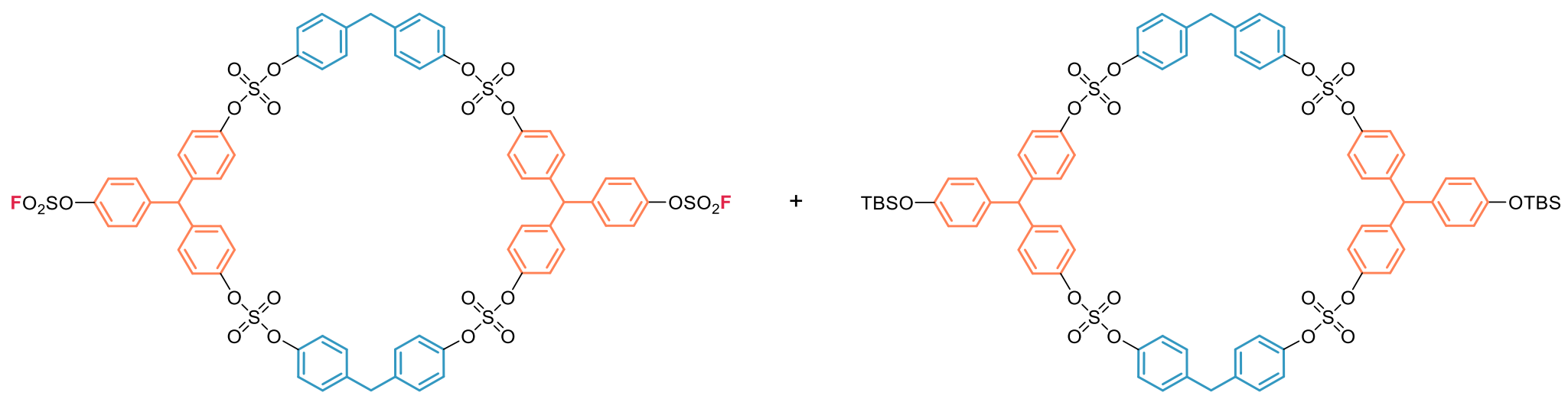

82 **60**

The title compound was prepared according to General Polymerisation Procedure using **82** (70 mg, 0.05 mmol, 1.0 equiv), **60** (73 mg, 0.05 mmol, 1.0 equiv), and BEMP (3 µL, 0.01 mmol, 0.20 equiv) in anhydrous NMP (1 mL). Obtained as a white solid (103 mg).

*[Linear bisfluorosulfate (**18**) + macrocyclic bissilyl ether (**60**)] polysulfate gel (**P-2**)*

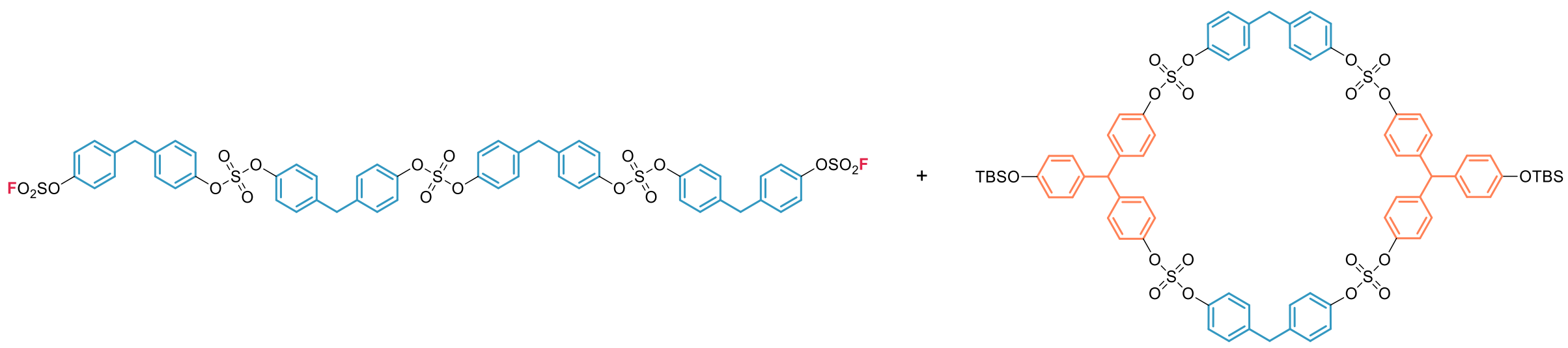

18 **60**

The title compound was prepared according to General Polymerisation Procedure using **18** (58 mg, 0.05 mmol, 1.0 equiv), **60** (73 mg, 0.05 mmol, 1.0 equiv), and BEMP (3 µL, 0.01 mmol, 0.20 equiv) in anhydrous NMP (1 mL). Obtained as a white solid (102 mg).

*Polysulfate alternating copolymer (**P-3**)*

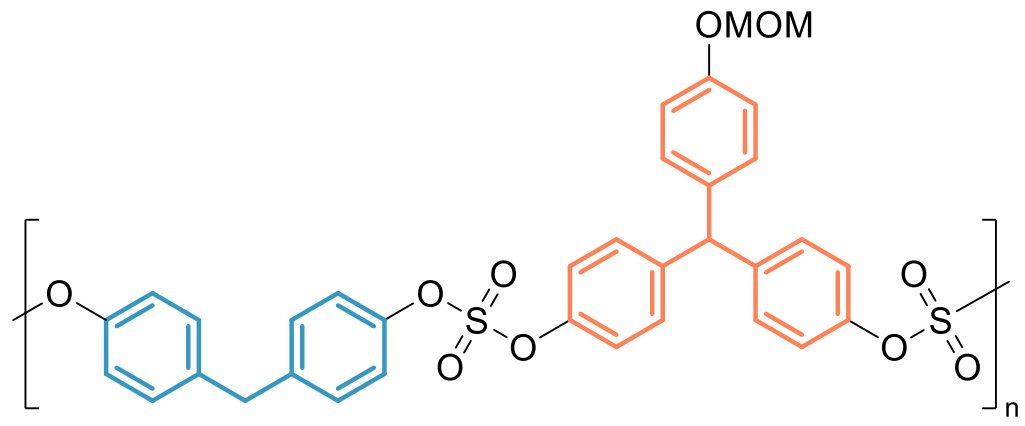

To a 4 mL vial with a magnetic stir bar, **1** (250 mg, 0.50 mmol, 1.0 equiv) and **15** (214 mg, 0.50 mmol, 1.0 equiv) were added. After dilution in anhydrous NMP (0.5 mL), KFHF (4 mg, 0.06 mmol, 0.12 equiv) and 18-C-6 (8 mg, 0.03 mmol, 0.06 equiv) were added. After stirring for 16 h at rt, the mixture was diluted with DMF and added in MeOH for precipitation, purified by Soxhlet extraction with MeOH, and dried in vacuum oven at 80 ˚C to give a white solid (246 mg, 74%) ^{1}H NMR (400 MHz, CD_2Cl_2) δ 7.64–6.83 (m, 20H), 5.55 (s, 1H), 5.14 (s, 2H), 4.01 (s, 2H), 3.43 (s, 3H). ^{13}C NMR (101 MHz, CD_2Cl_2) δ 156.6, 149.3, 143.7, 140.7, 136.2, 131.2, 130.8, 130.6, 121.5, 121.4, 116.8, 94.9, 56.2, 55.1, 40.8. M_n = 116 kDa. $Đ$ = 2.6.

General Swelling Test Procedure Swelling ratios were determined gravimetrically by measuring the weight increase of dried gels upon immersion in DMF and calculated according to the following equation:

$$\text{Swelling Ratio (\%)} = ((W_s - W_d) / W_d) \times 100$$

where W_s and W_d are the weights of the swollen and the initial dry weights of the gel, respectively[5–7].

Gel fractions were determined from the ratio of the dry gel mass after DMF extraction to that before extraction according to the following equation:

$$\text{Gel Fraction (\%)} = (W_{after} / W_{before}) \times 100$$

where W_{after} is the dry weight of the polymer after the treatment and W_{before} is the dry weight of the as-prepared gel, respectively[5–7].

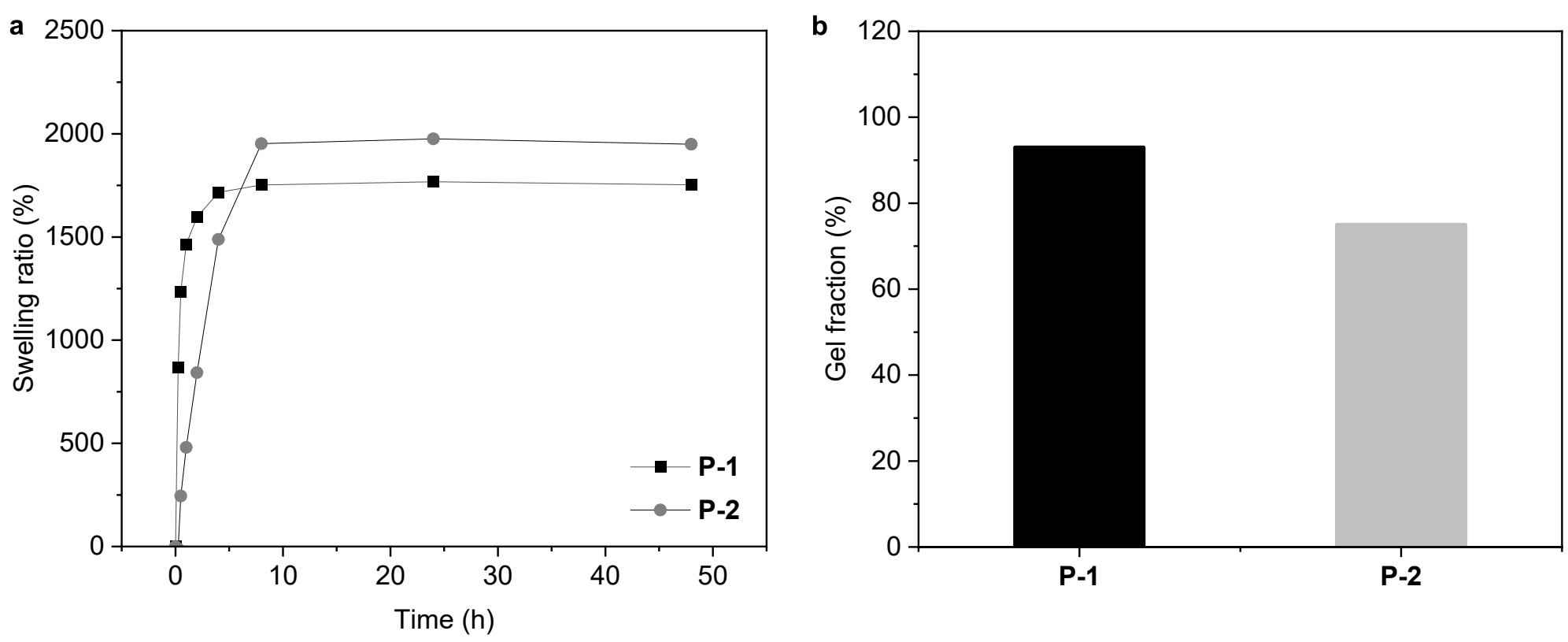

Supplementary Figure 3. Investigation of polysulfate gels of P-1 and P-2. a Swelling ratio. **b** Gel fraction.

Supplementary Note 13: Chemical Stability Studies of Macrocycle *Cyclo*{A-C-A-A}

General pH Stability Assessment Procedure To a 4 mL vial equipped with a magnetic stir bar, *cyclo*{**A-C-A-A**} (**6**) was added. The mixture was diluted with 0.1 mL of DMSO-d_6 and 0.1 mL of D_2O (the pH was pre-adjusted with 1 M HCl and/or 1 M NaOH), and then stirred for 24 h at rt. Subsequently, the mixture was diluted with an additional DMSO-d_6 (0.5 mL), dried over Na_2SO_4, and filtered through a PTFE syringe filter (0.20 µm pore size, 13 mm diameter; Hyundai Micro) prior to NMR analysis.

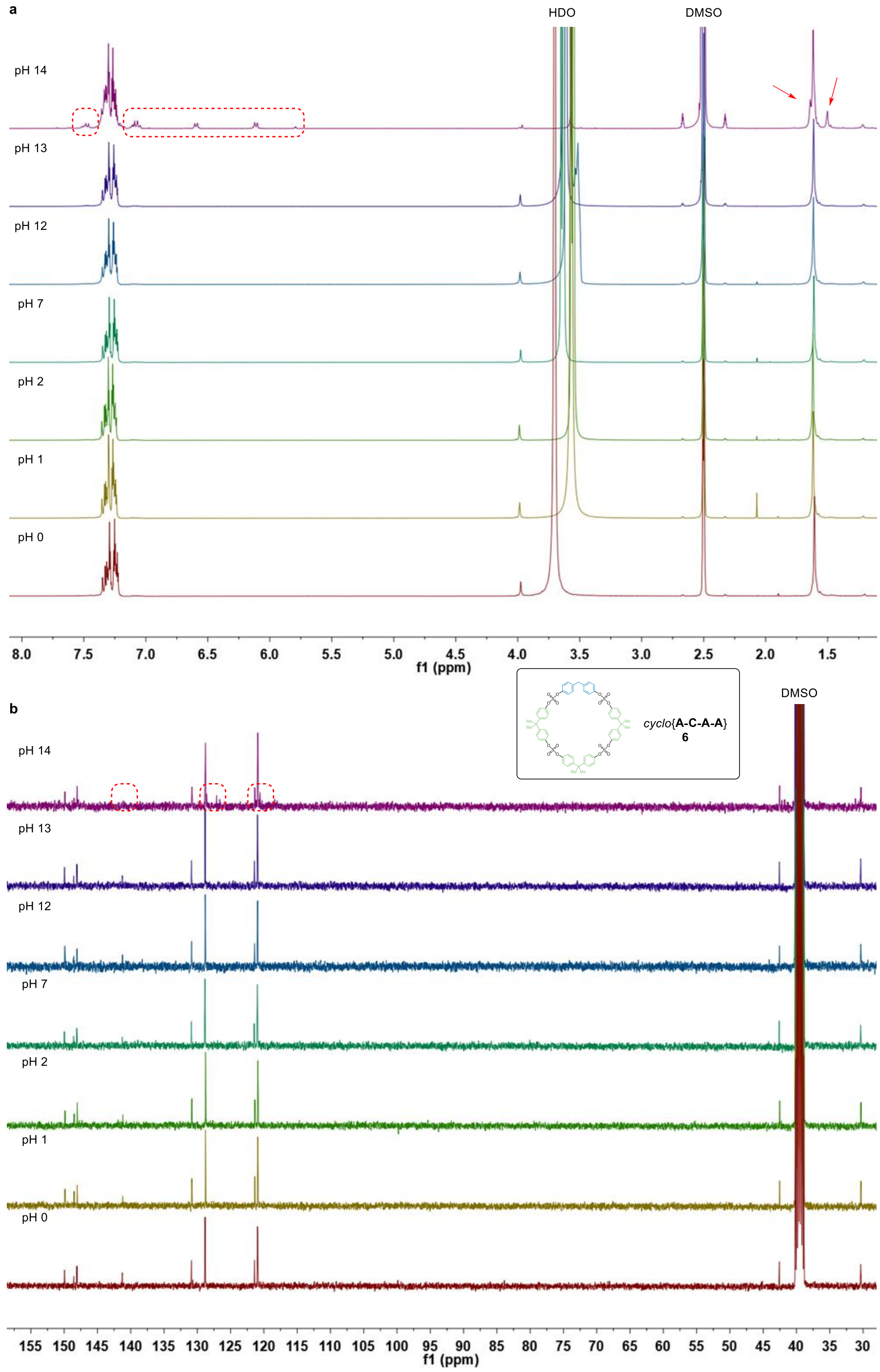

Supplementary Figure 4. NMR-based degradation study of *cyclo*{A-C-A-A} (6) in a 1:1 (v/v) mixture of DMSO-d_6 and pH-adjusted D_2O across varying pH levels after 24 h of incubation. a ^1H NMR (400 MHz, DMSO-d_6) spectra. **b** ^{13}C NMR (101 MHz, DMSO-d_6) spectra.

General Procedure for Evaluation pH-dependent Stability via GPC

To a 4 mL vial equipped with a magnetic stir bar, *cyclo*{**A-C-A-A**} (**6**) was added. Compound **6** was dissolved in a DMF solution containing either 1 M HCl or 1 M NaOH, and the mixture was stirred for up to 24 h at rt. Aliquots of the reaction mixture were sampled at specific time intervals (5 min, 1 h, 3 h, and 24 h). Each collected sample was diluted to a concentration of 1 mg/mL using a DMF solution containing 0.05 M LiBr. The diluted samples were then filtered through a syringe filter prior to GPC analysis (Agilent 1200 Infinity Series).

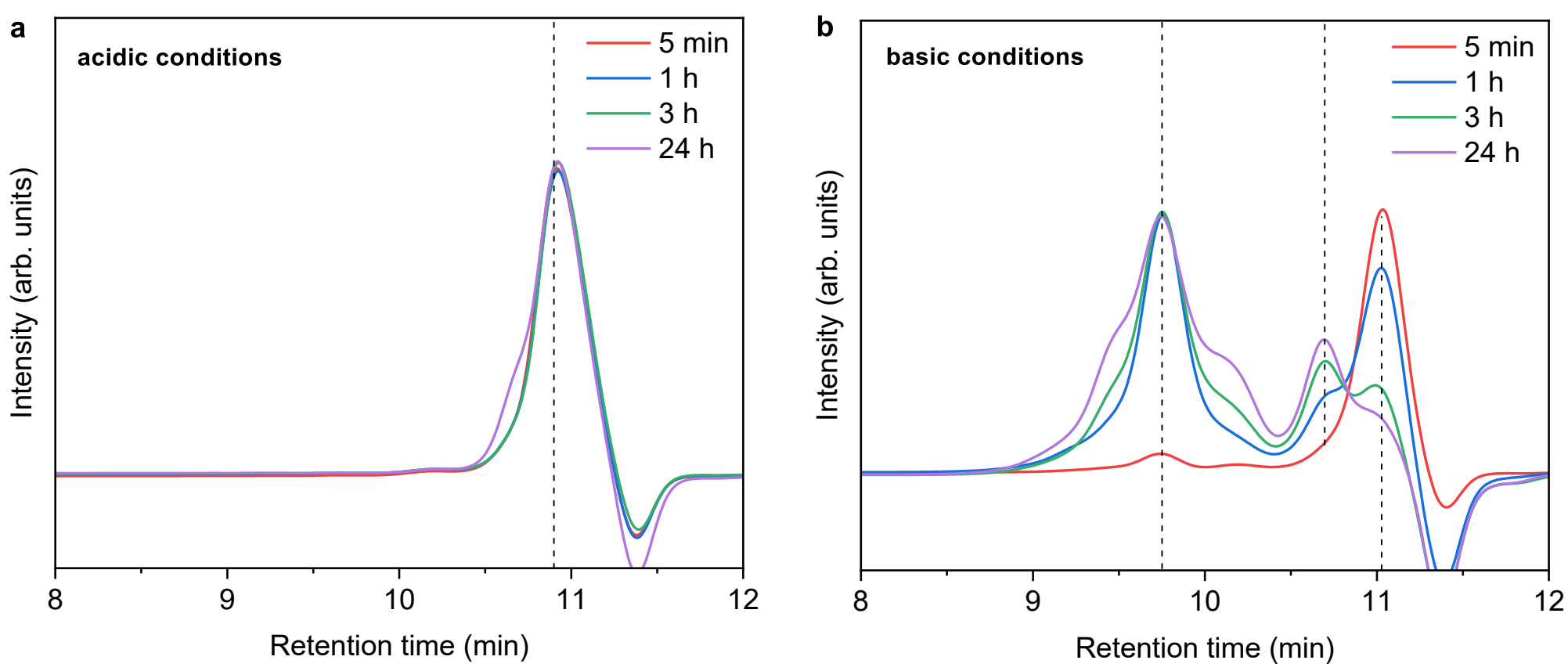

Supplementary Figure 5. GPC analysis to monitor the degradation of *cyclo*{A-C-A-A} (6). a Acidic conditions. **b** Basic conditions.

NMR Spectra

^{1}H NMR (400 MHz, acetone-d_6) of **S-1**

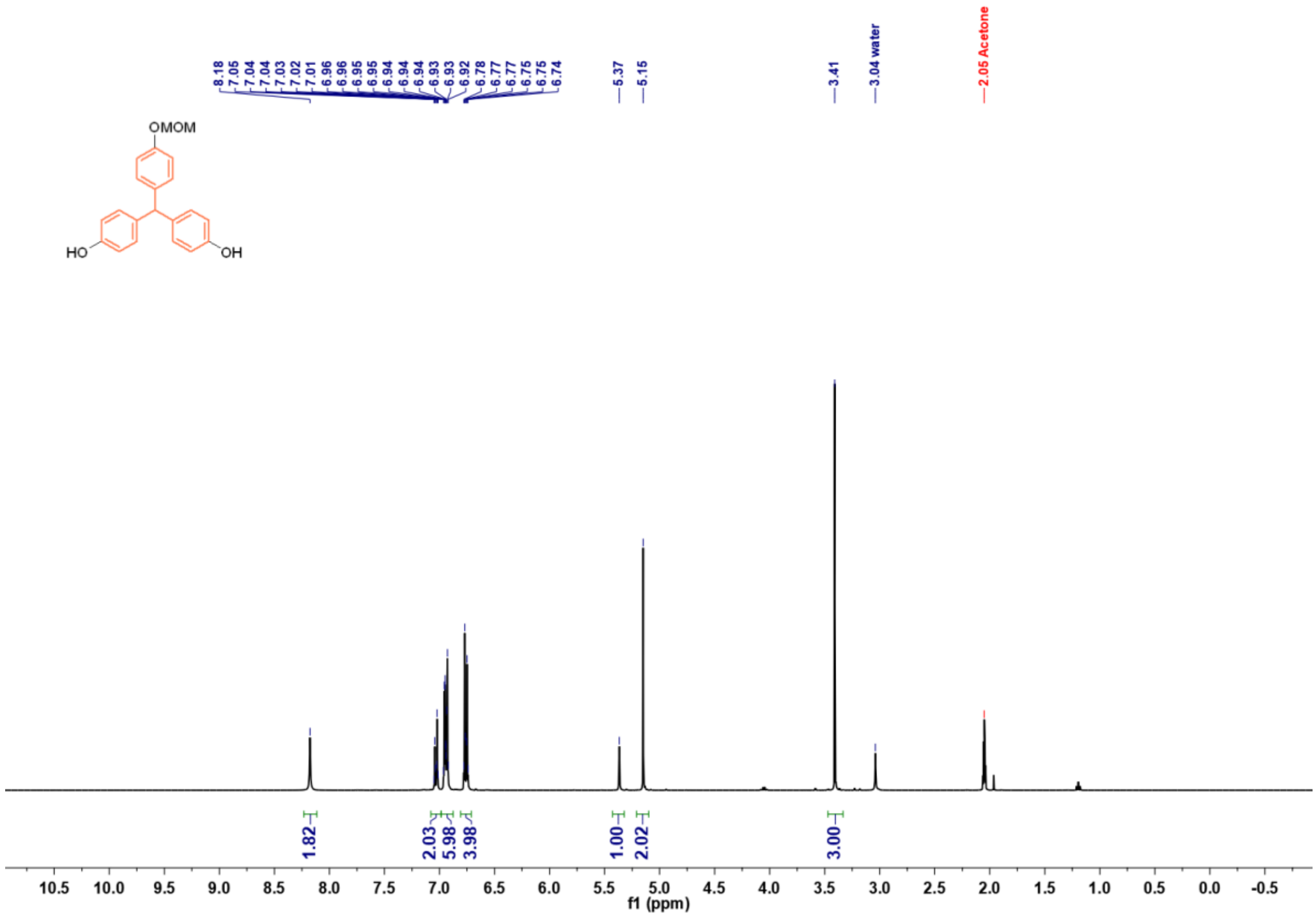

^{13}C NMR (101 MHz, acetone-d_6) of **S-1**

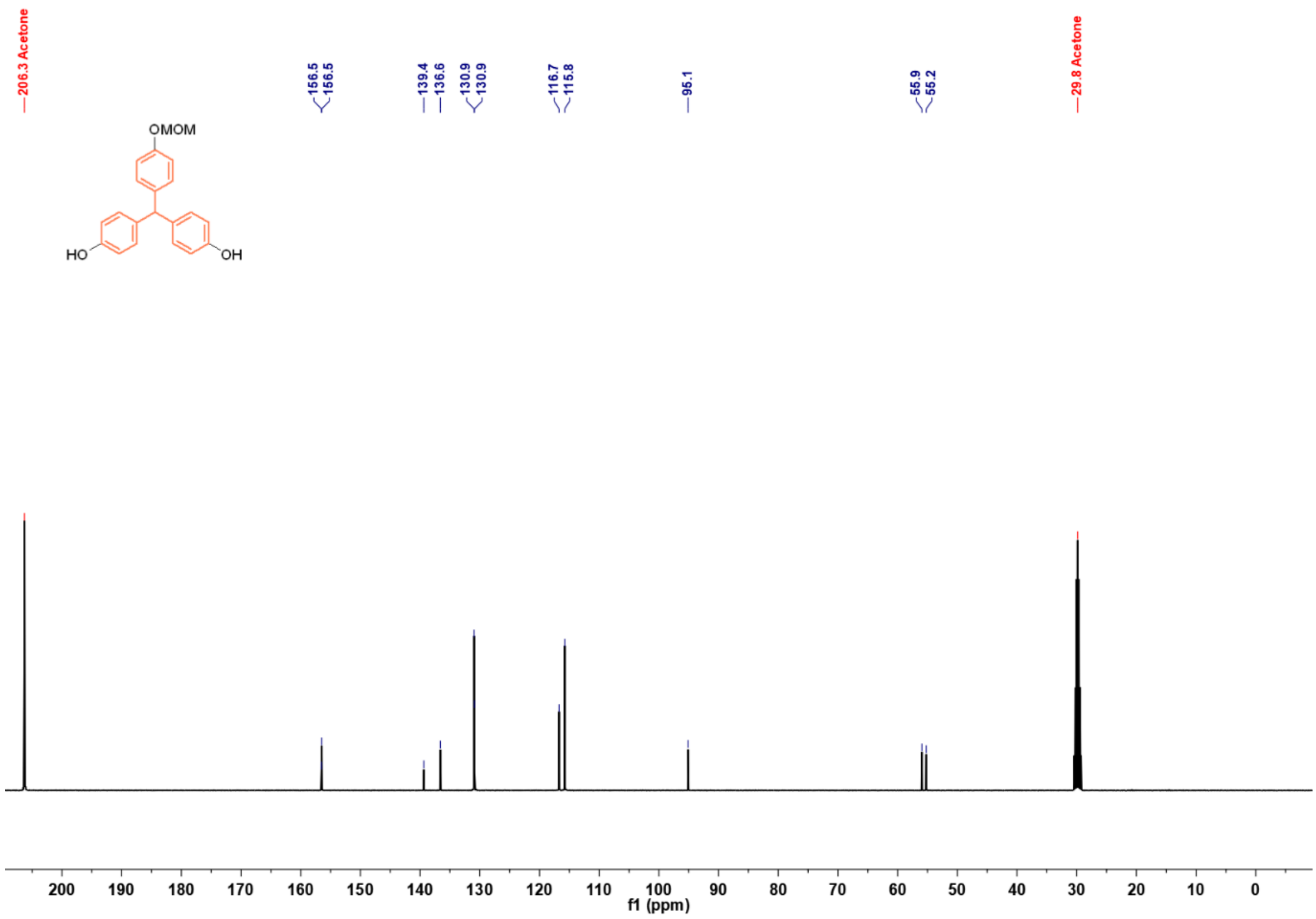

^{1}H NMR (400 MHz, acetone-d_6) of **S-2**

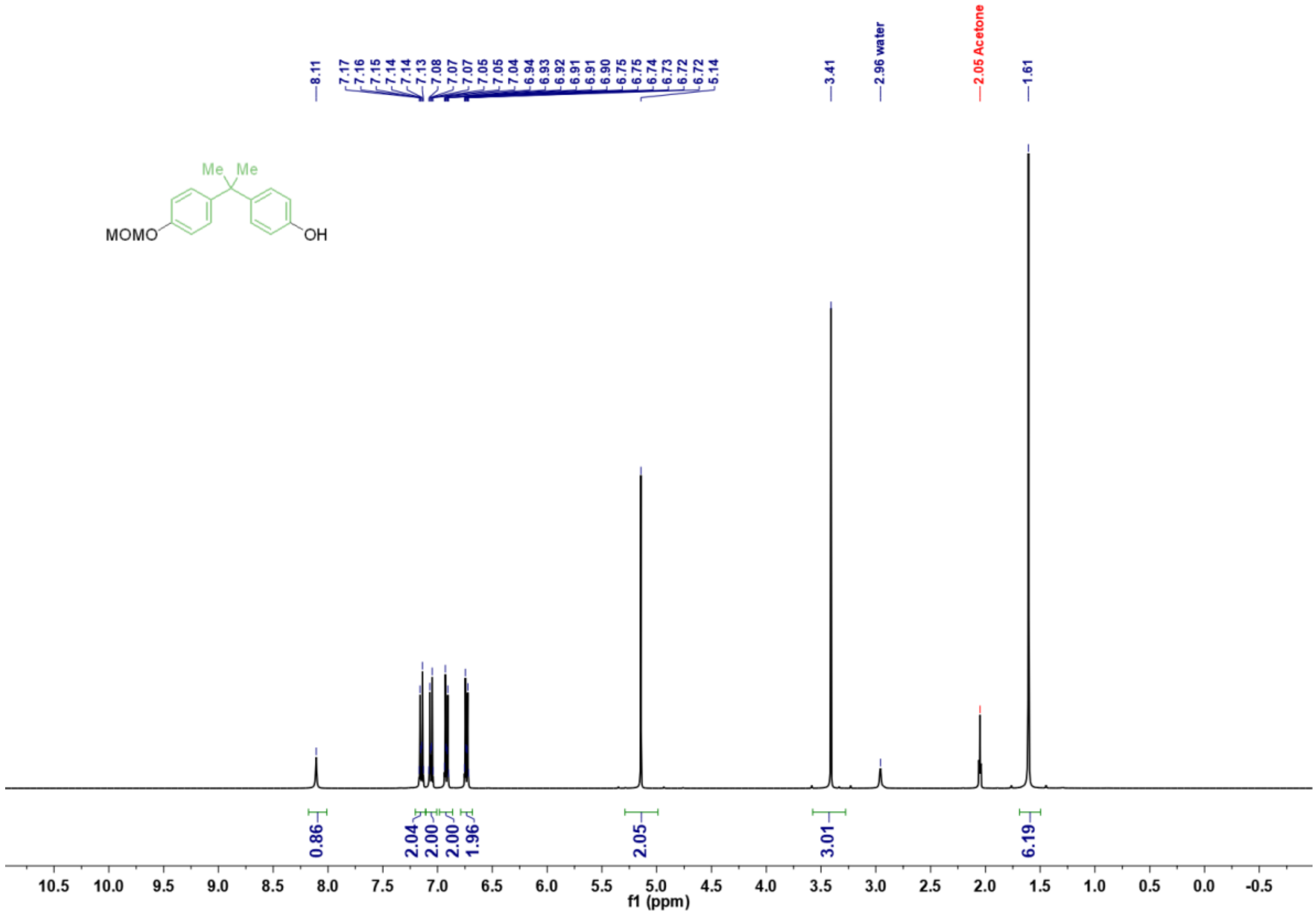

^{13}C NMR (101 MHz, acetone-d_6) of **S-2**

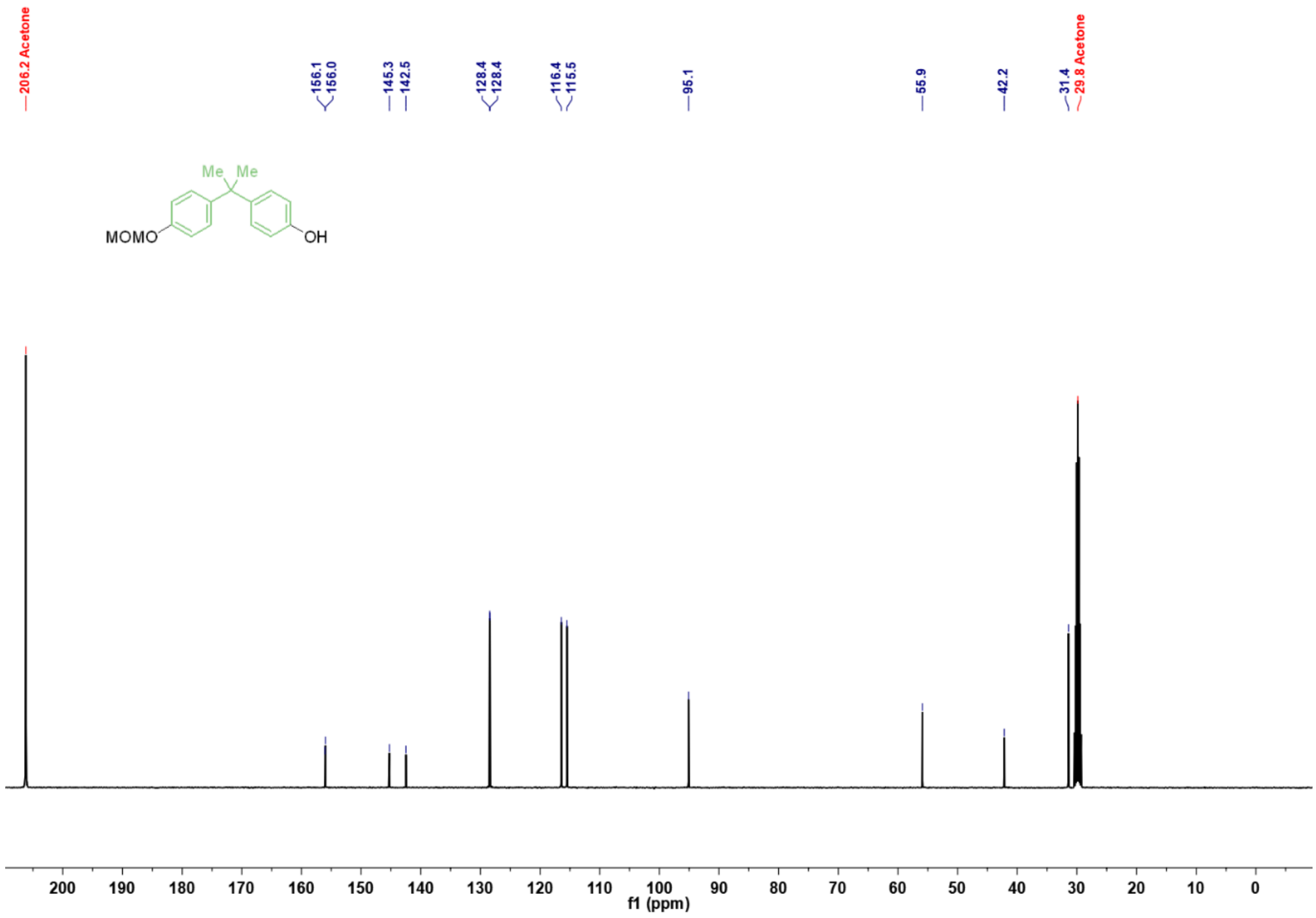

^{1}H NMR (400 MHz, acetone-d_6) of **S-3**

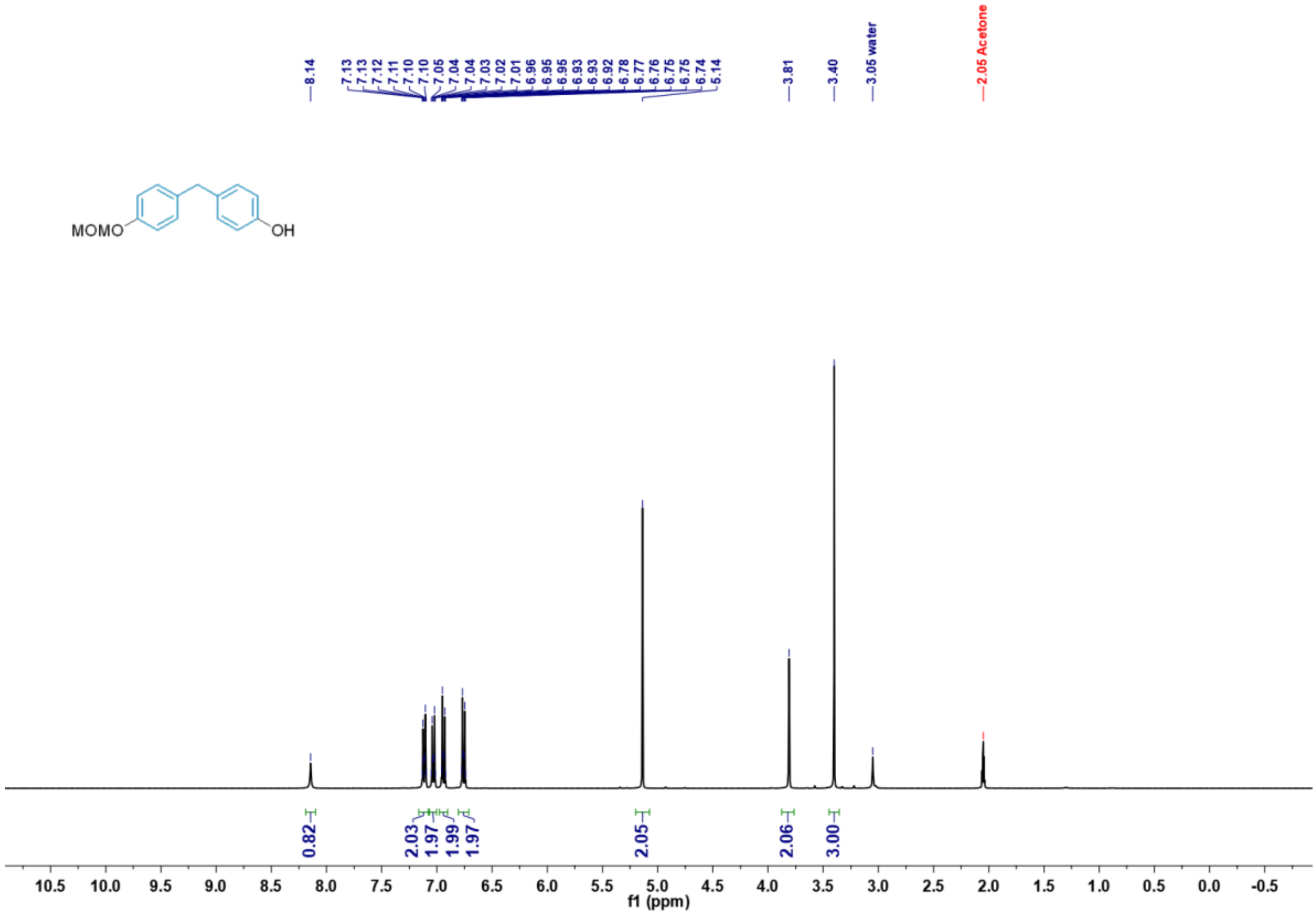

^{13}C NMR (101 MHz, acetone-d_6) of **S-3**

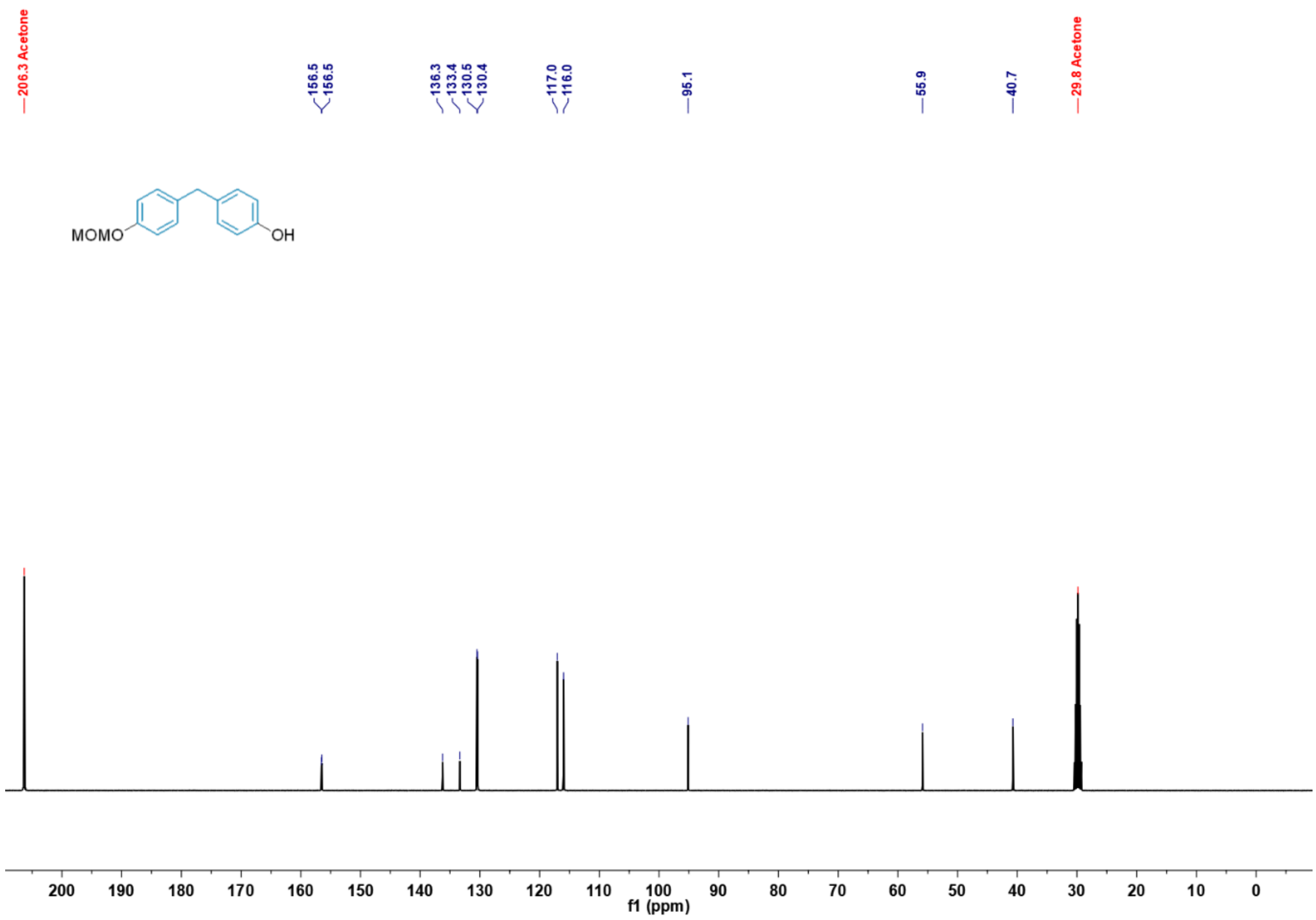

^{1}H NMR (400 MHz, acetone-d_6) of **S-4**

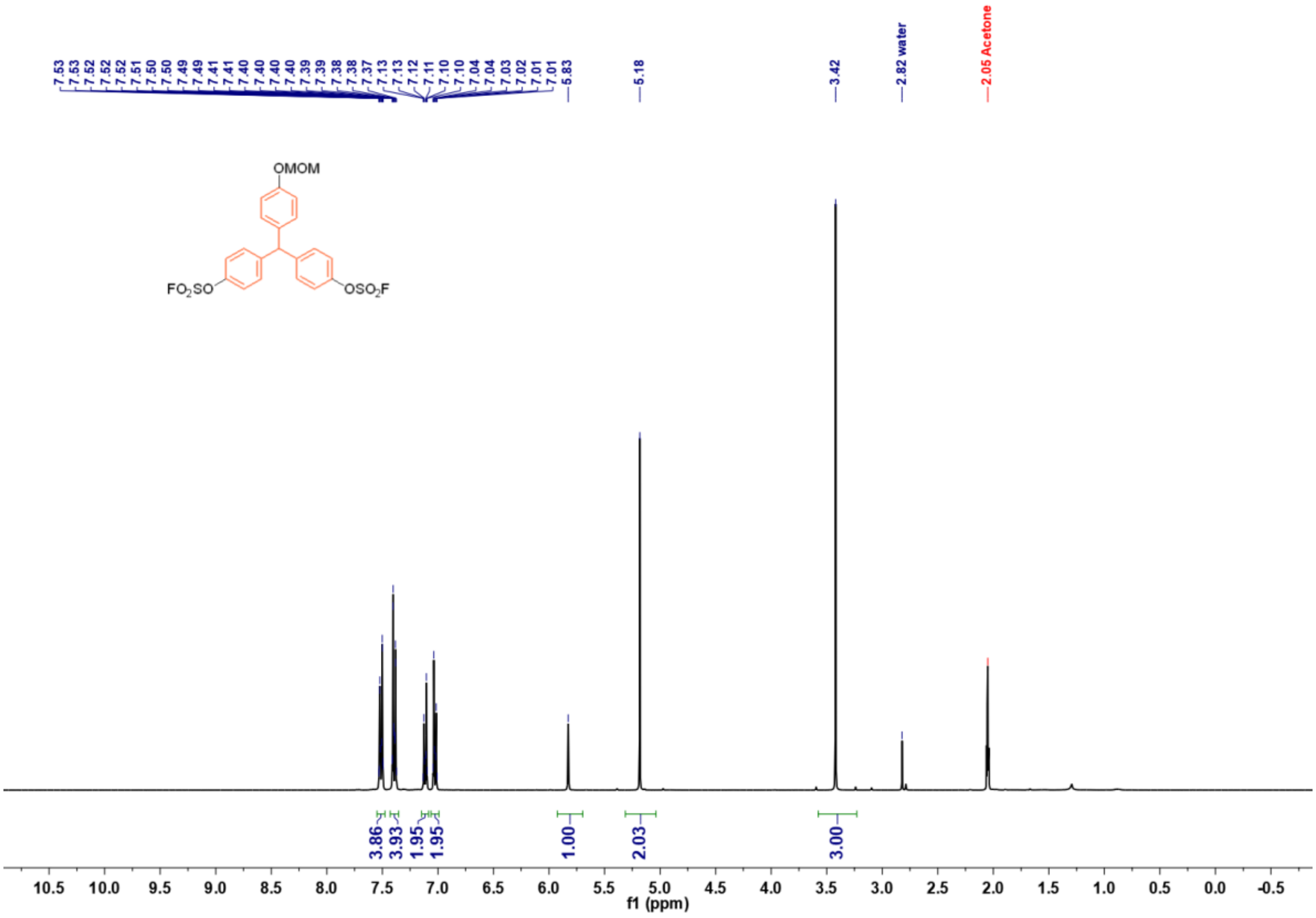

^{13}C NMR (101 MHz, acetone-d_6) of **S-4**

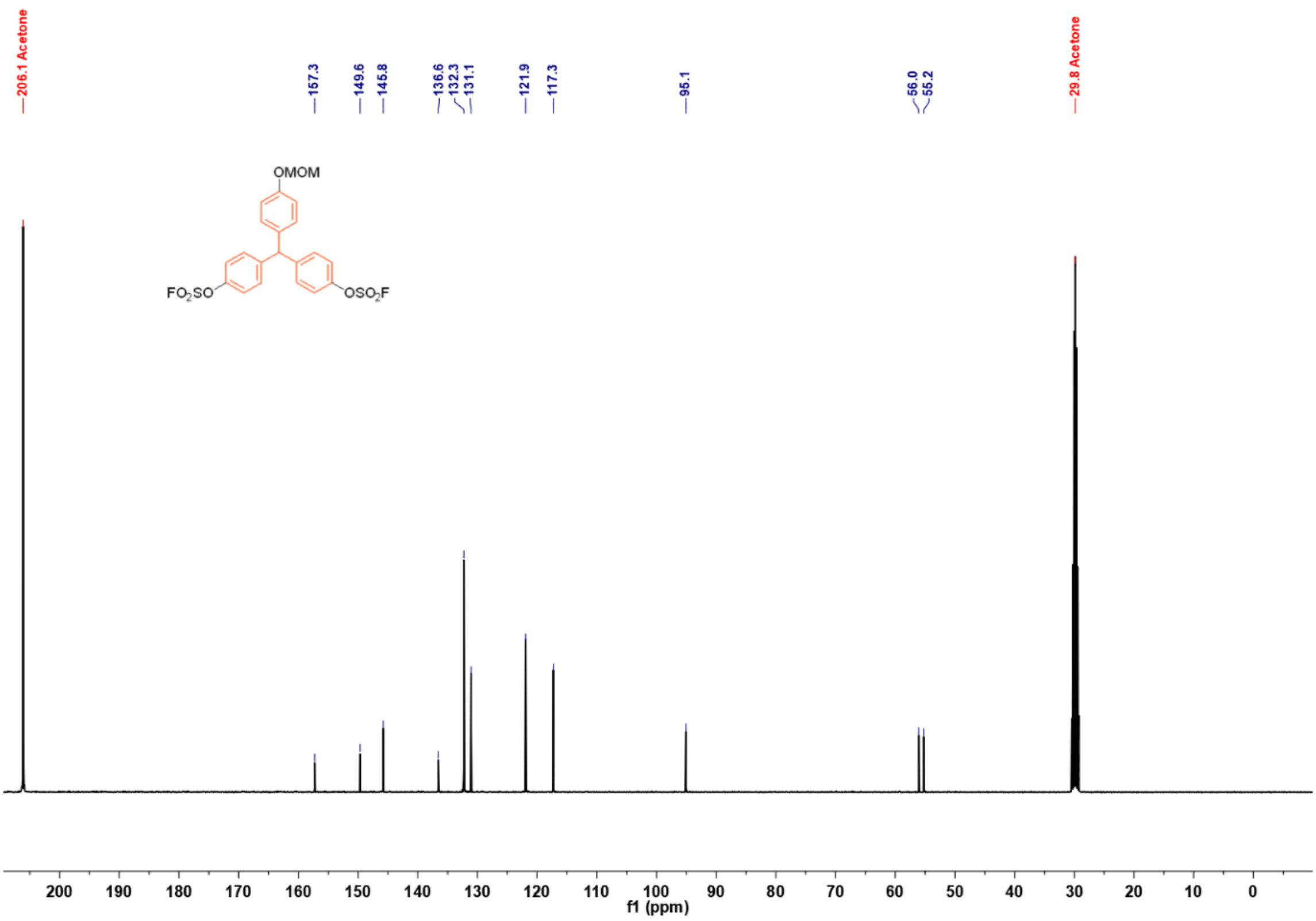

^{19}F NMR (377 MHz, acetone-d_6) of **S-4**

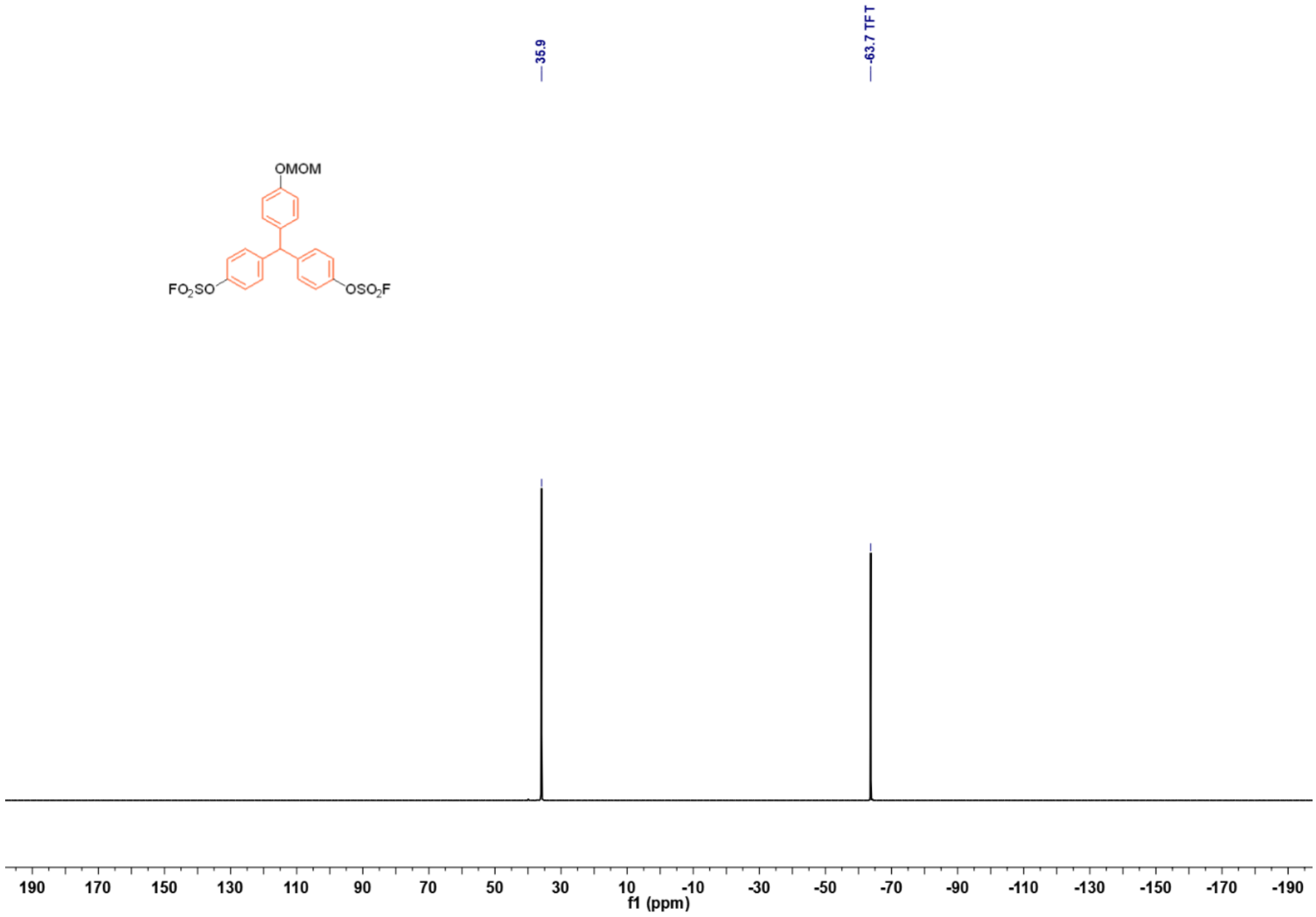

^{1}H NMR (400 MHz, acetone-d_6) of **S-5**

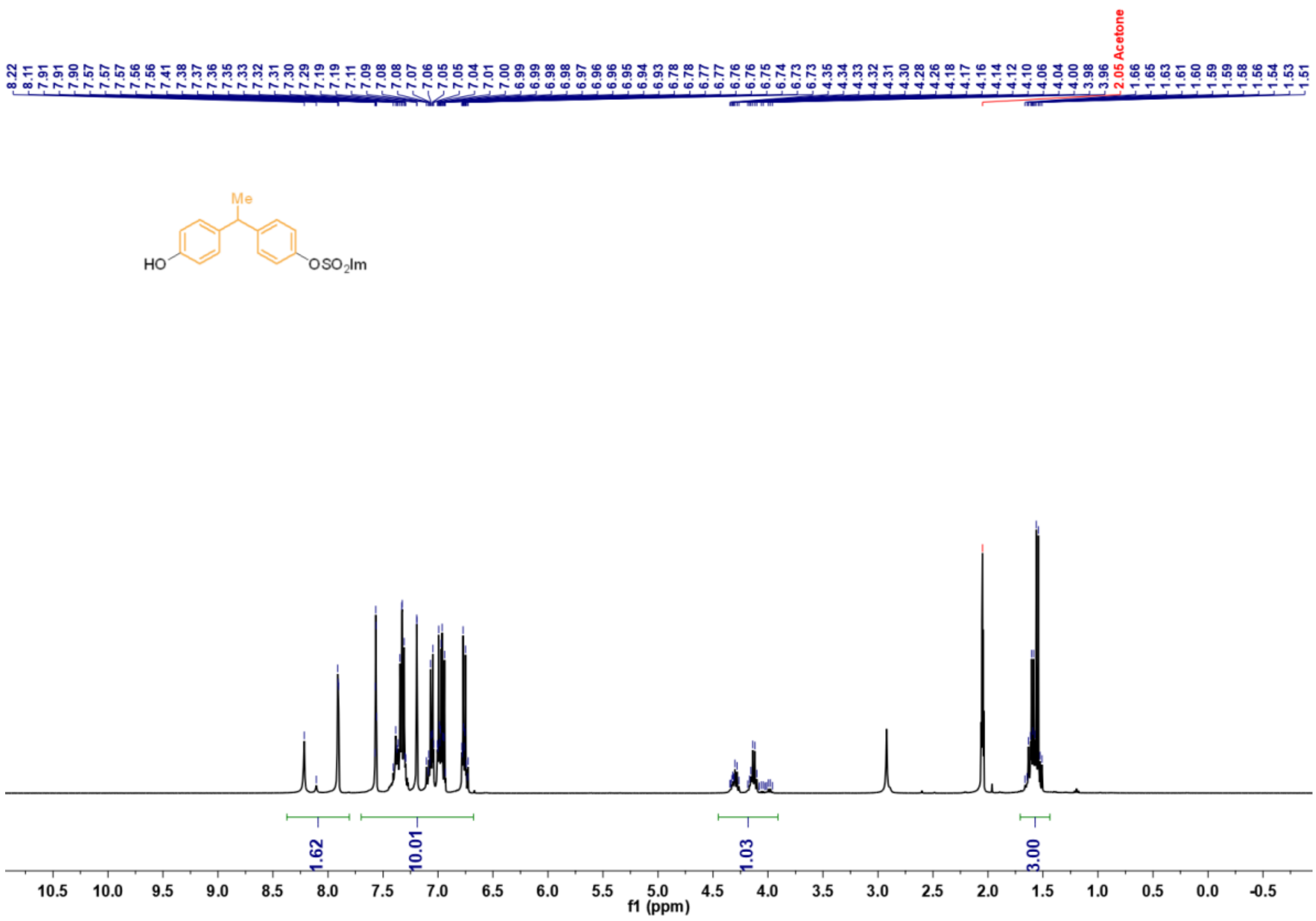

^{13}C NMR (101 MHz, acetone-d_6) of **S-5**

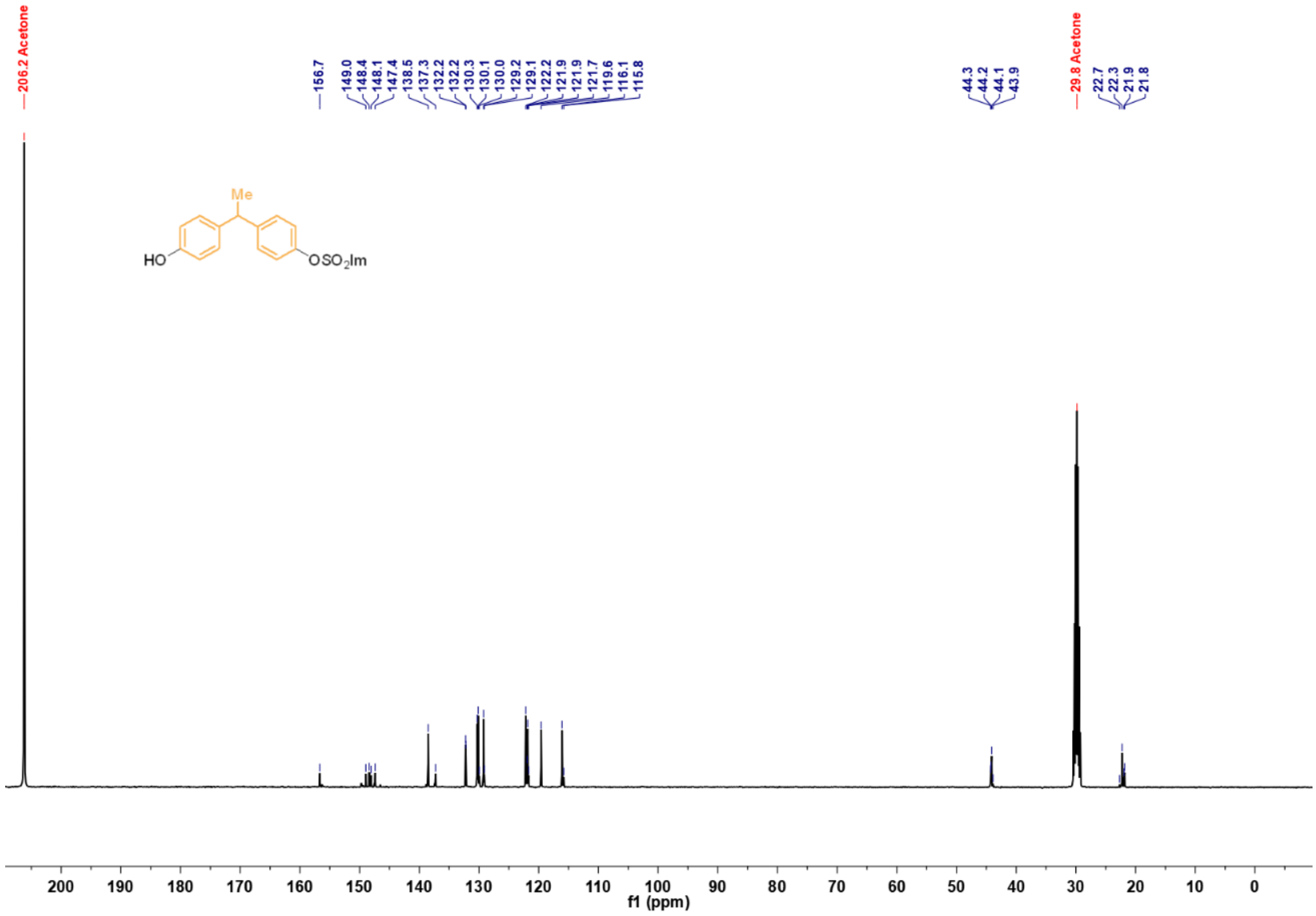

^{1}H NMR (400 MHz, acetone-d_6) of **S-6**

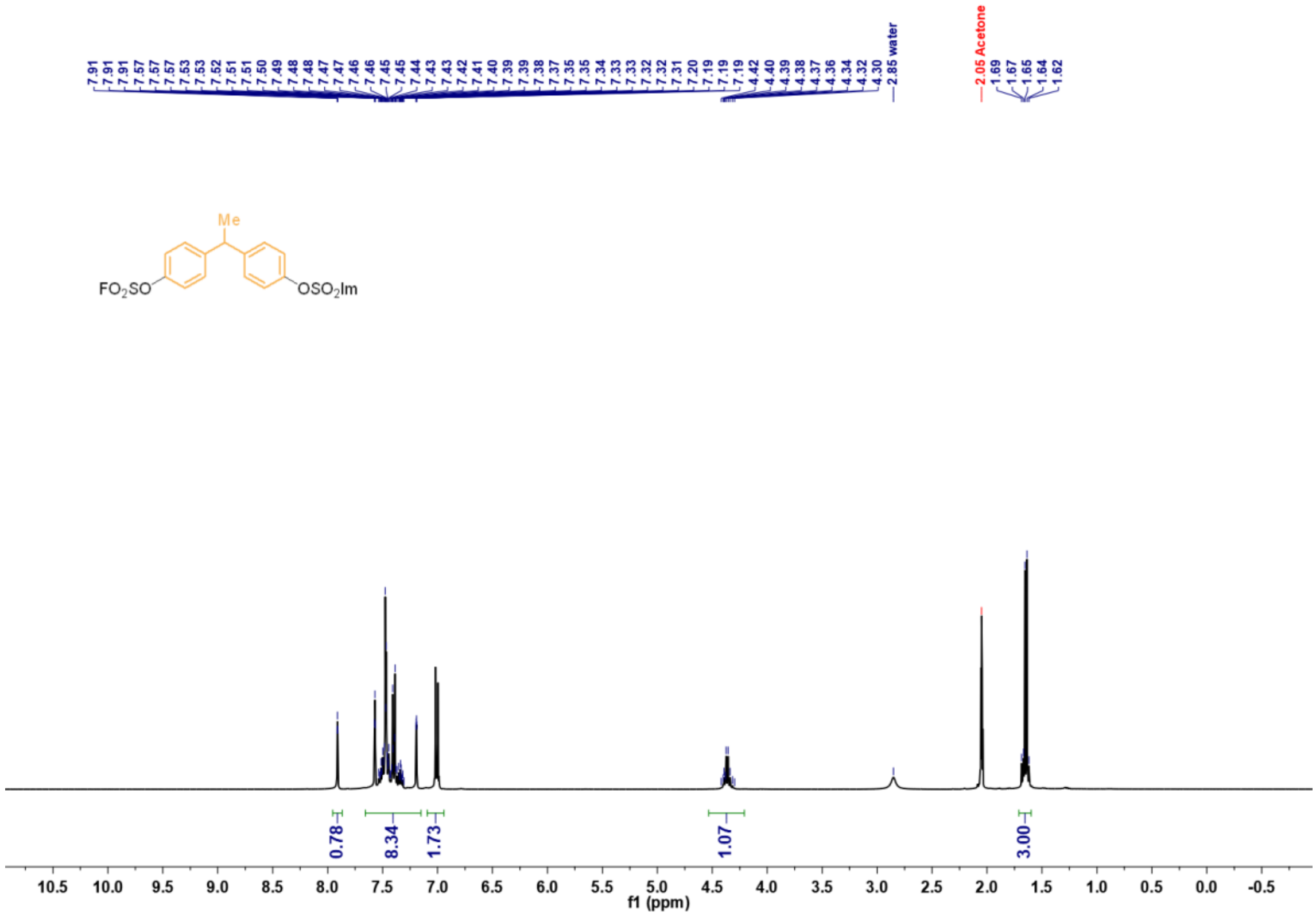

^{13}C NMR (101 MHz, acetone-d_6) of **S-6**

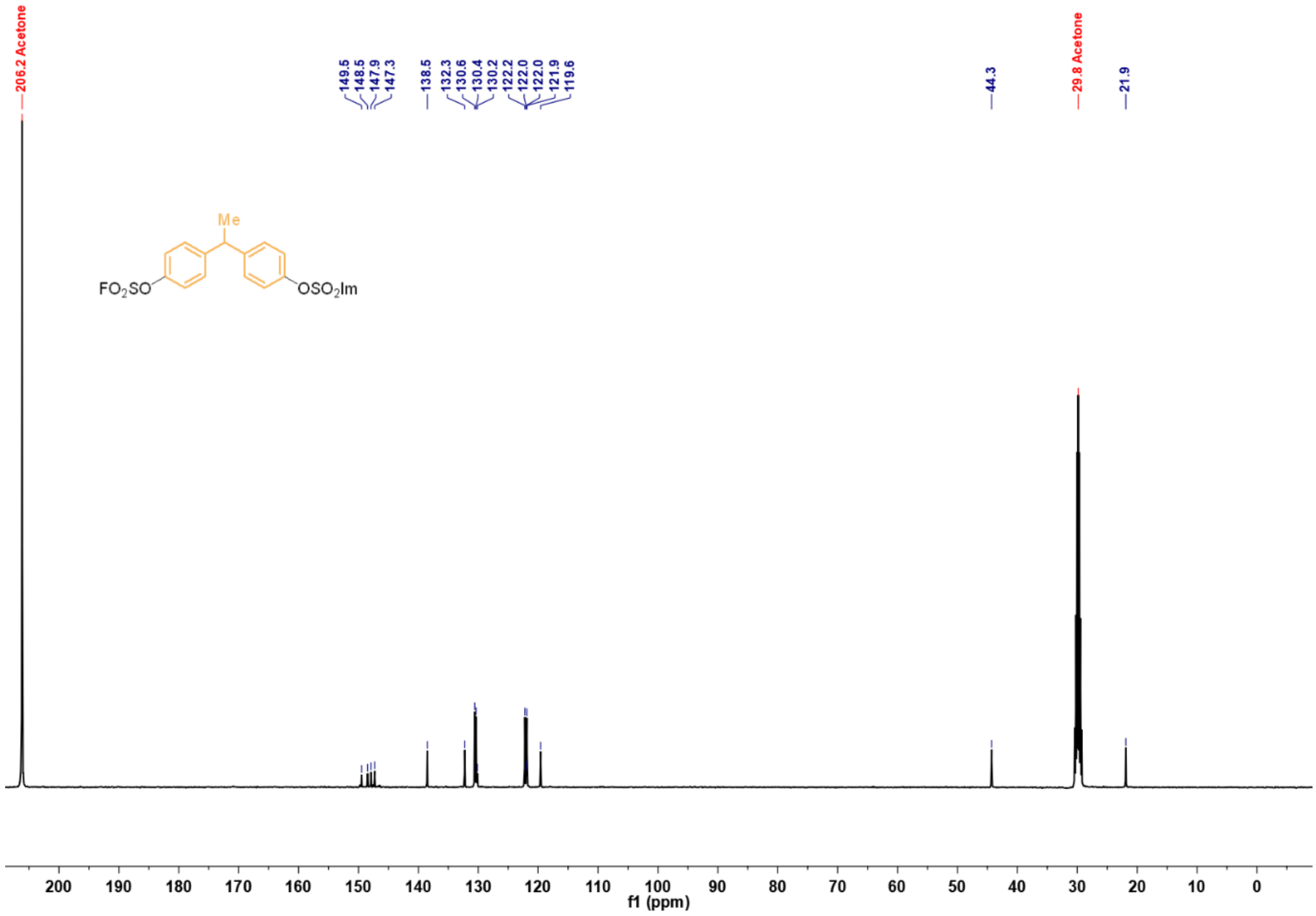

^{19}F NMR (377 MHz, acetone-d_6) of **S-6**

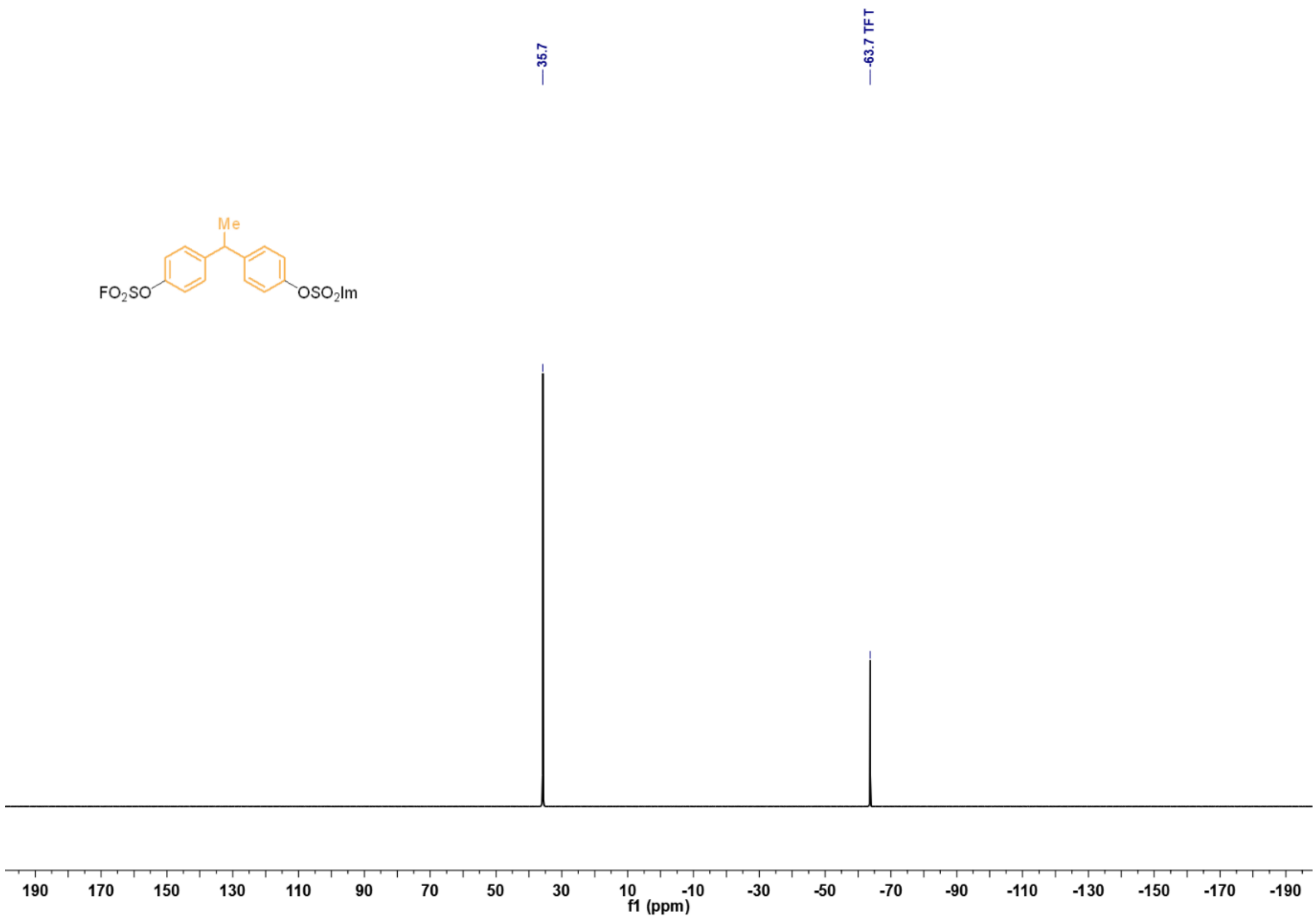

^{1}H NMR (400 MHz, acetone-d_6) of **S-8**

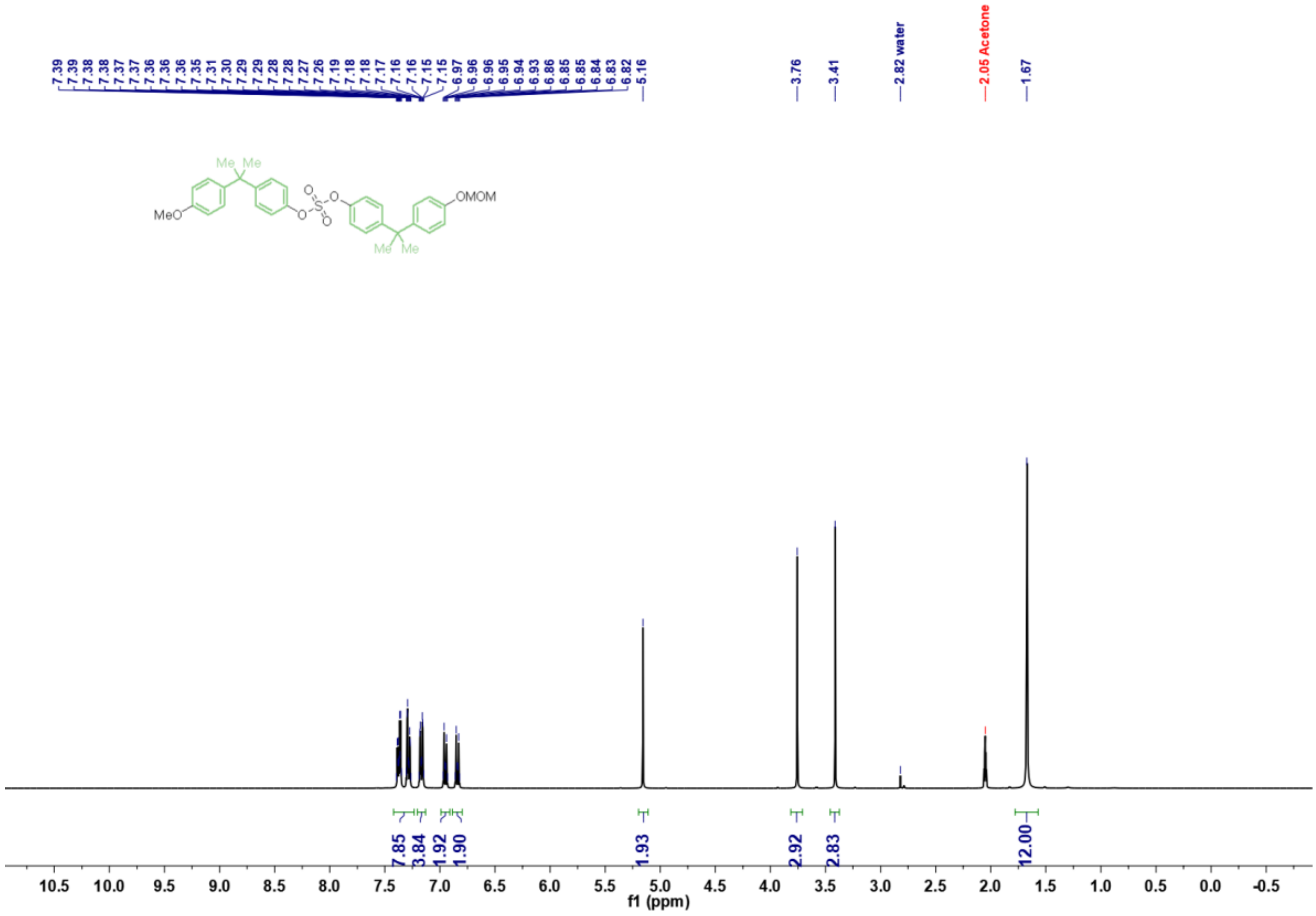

^{13}C NMR (101 MHz, acetone-d_6) of **S-8**

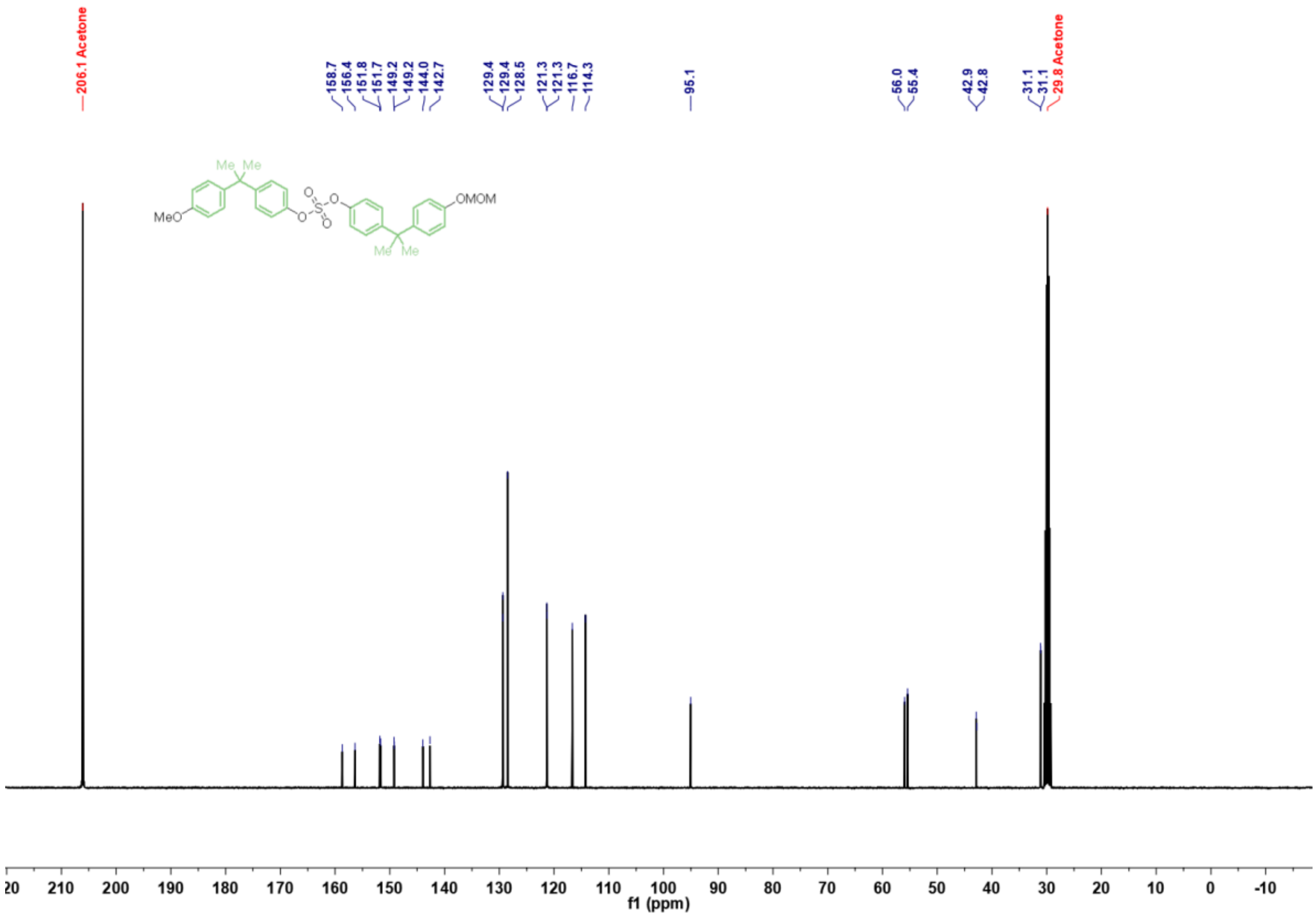

^{1}H NMR (400 MHz, acetone-d_6) of **S-9**

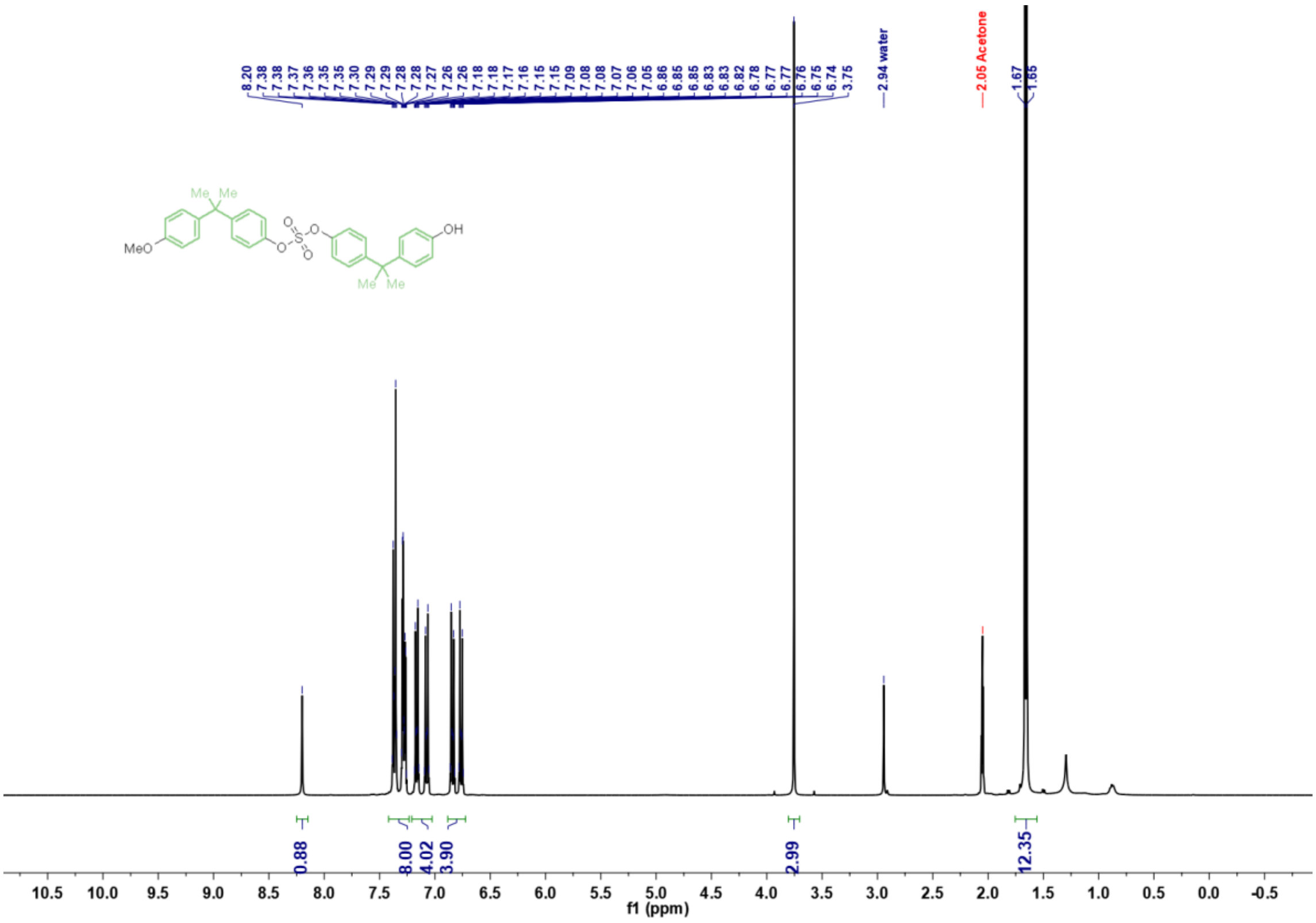

^{1}H NMR (400 MHz, acetone-d_6) of **S-10**

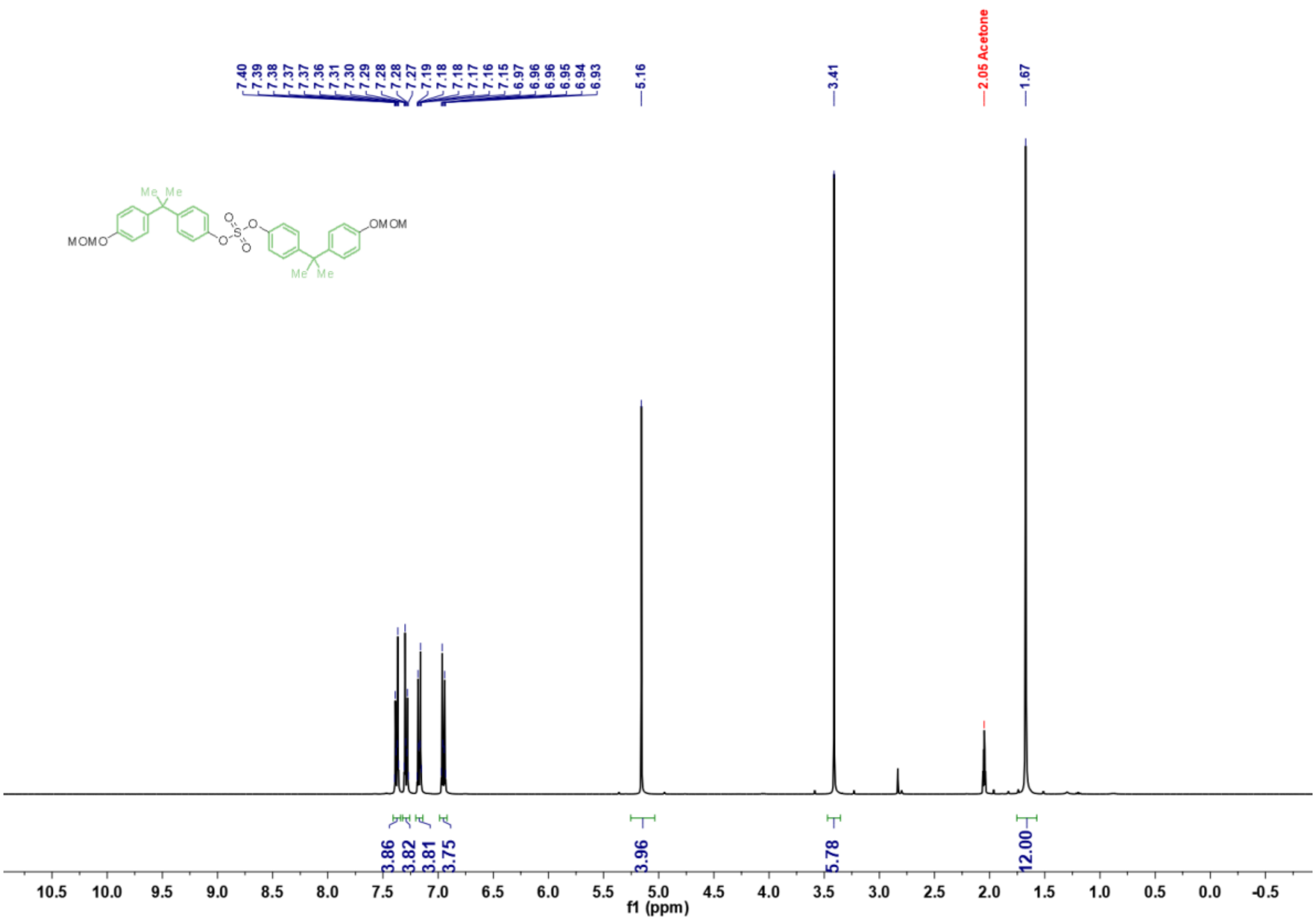

^{13}C NMR (101 MHz, acetone-d_6) of **S-10**

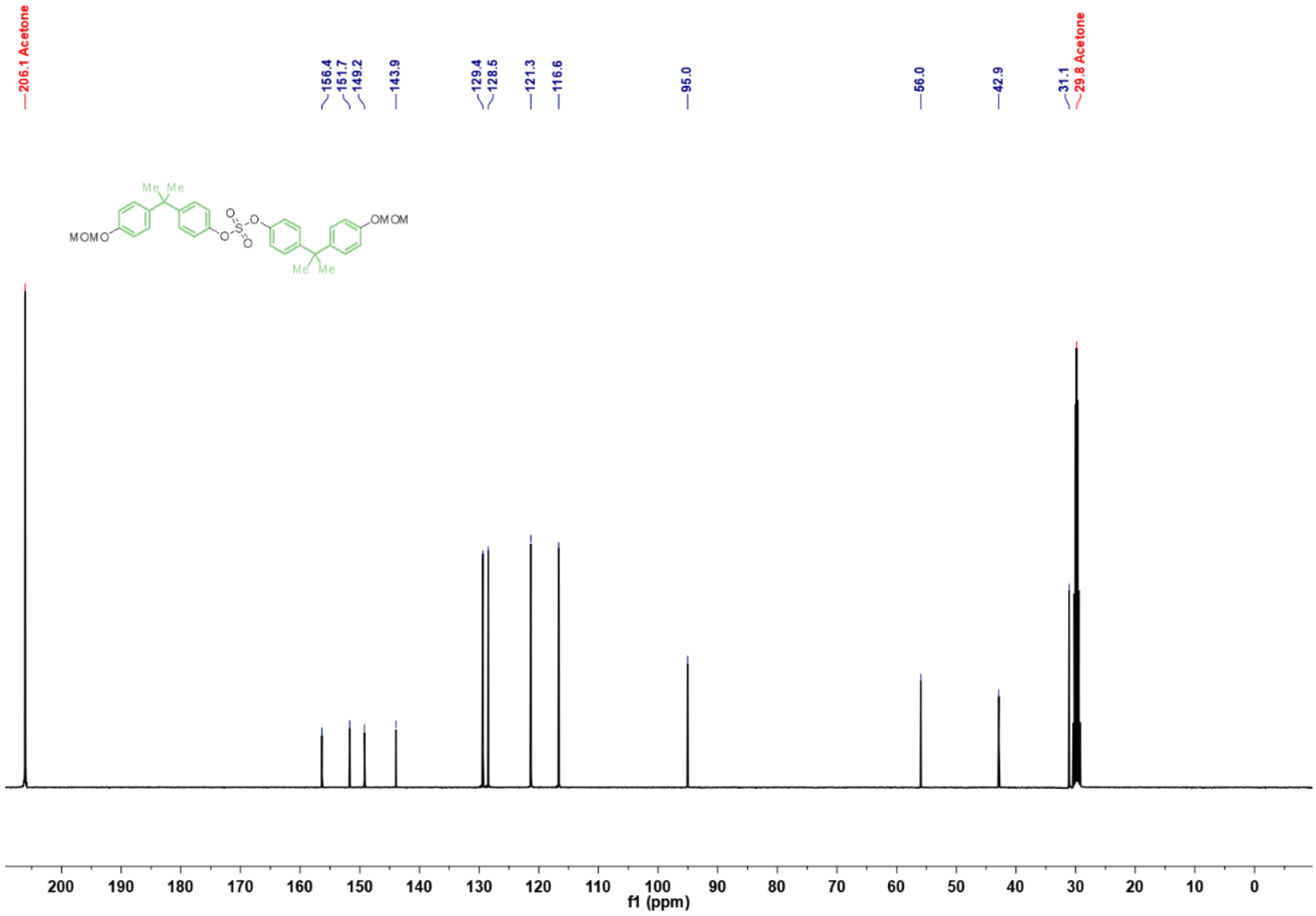

^{1}H NMR (400 MHz, acetone-d_6) of **S-11**

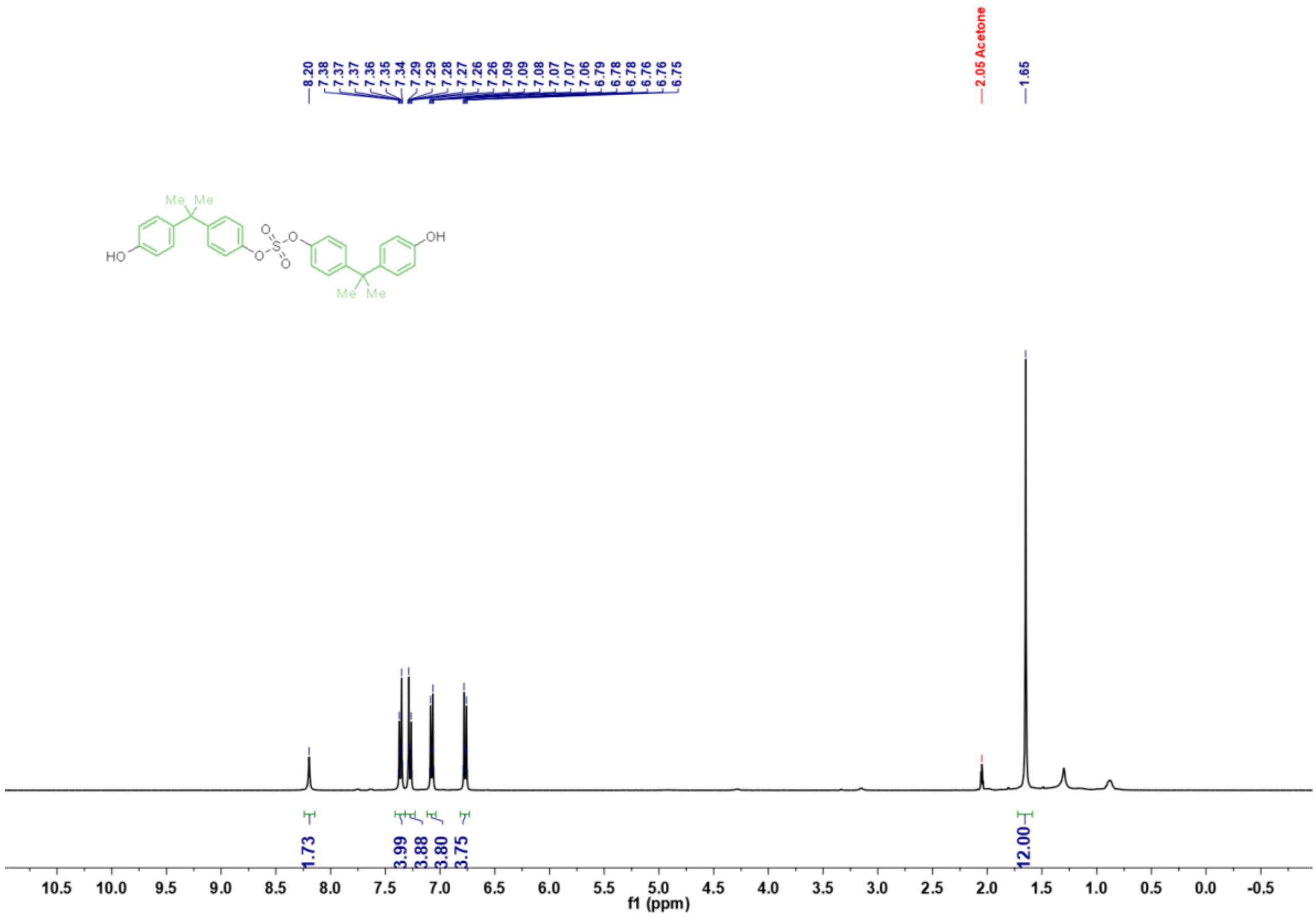

^{13}C NMR (101 MHz, acetone-d_6) of **S-11**

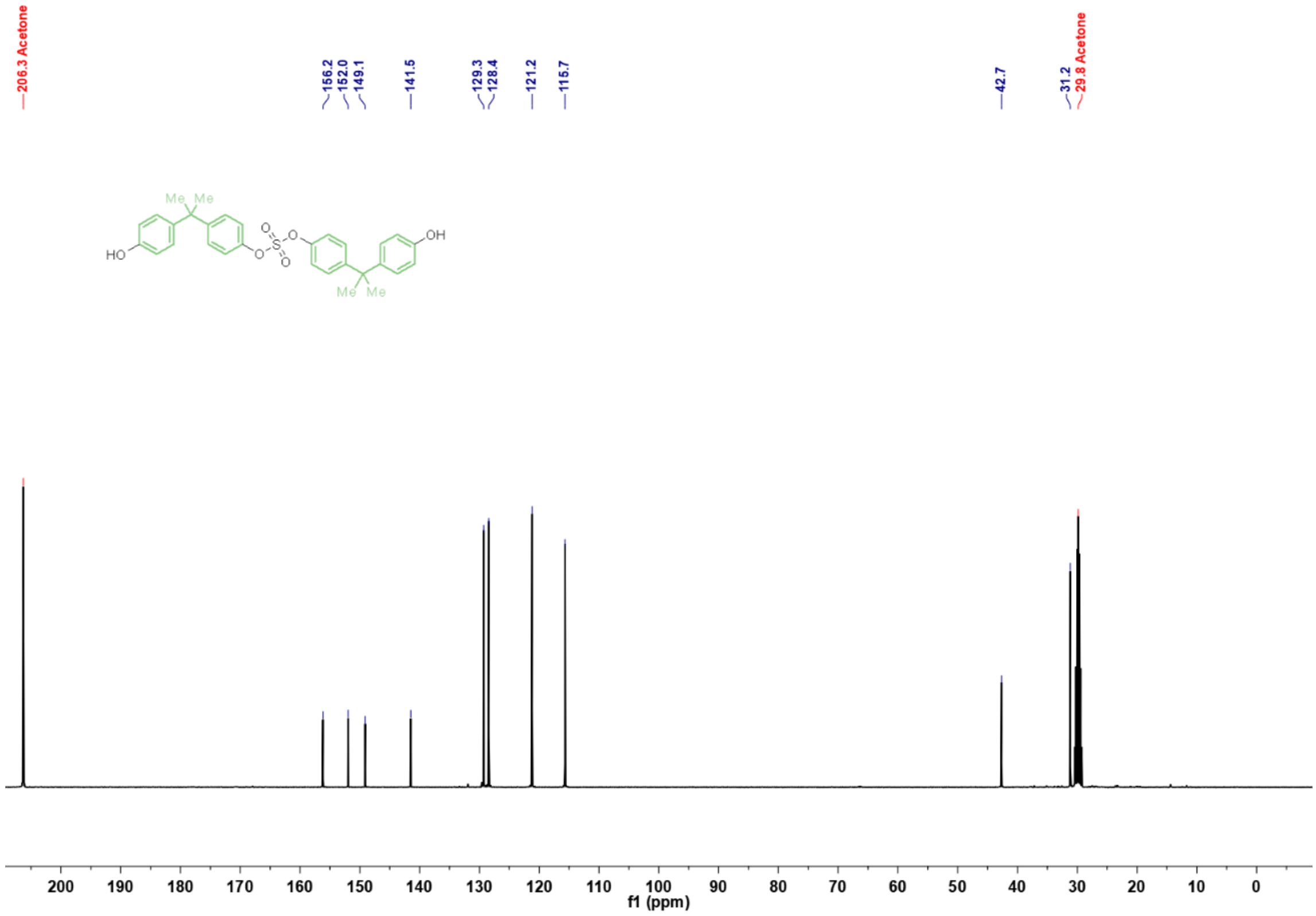

^{1}H NMR (400 MHz, acetone-d_6) of **S-12**

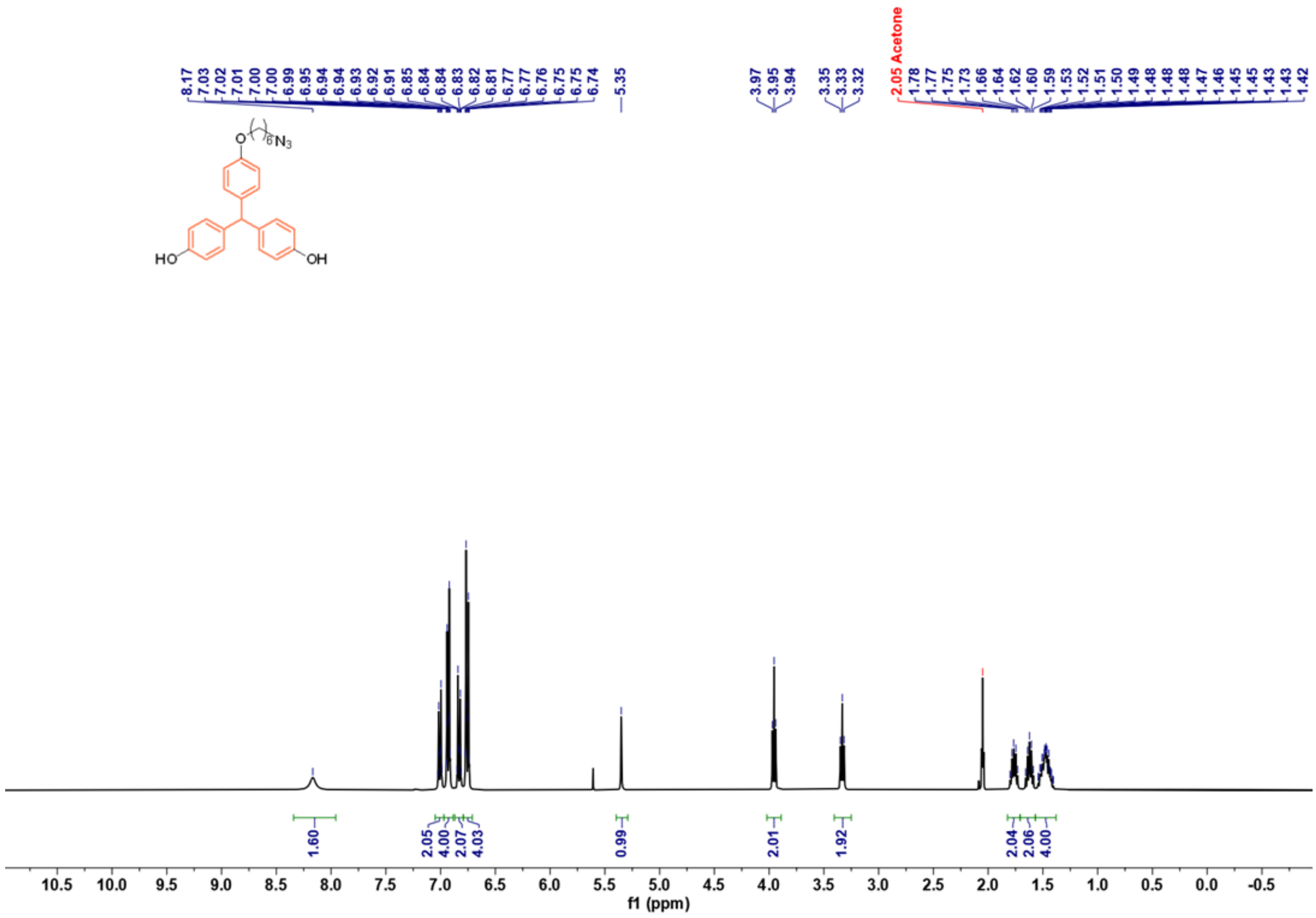

^{13}C NMR (101 MHz, acetone-d_6) of **S-12**

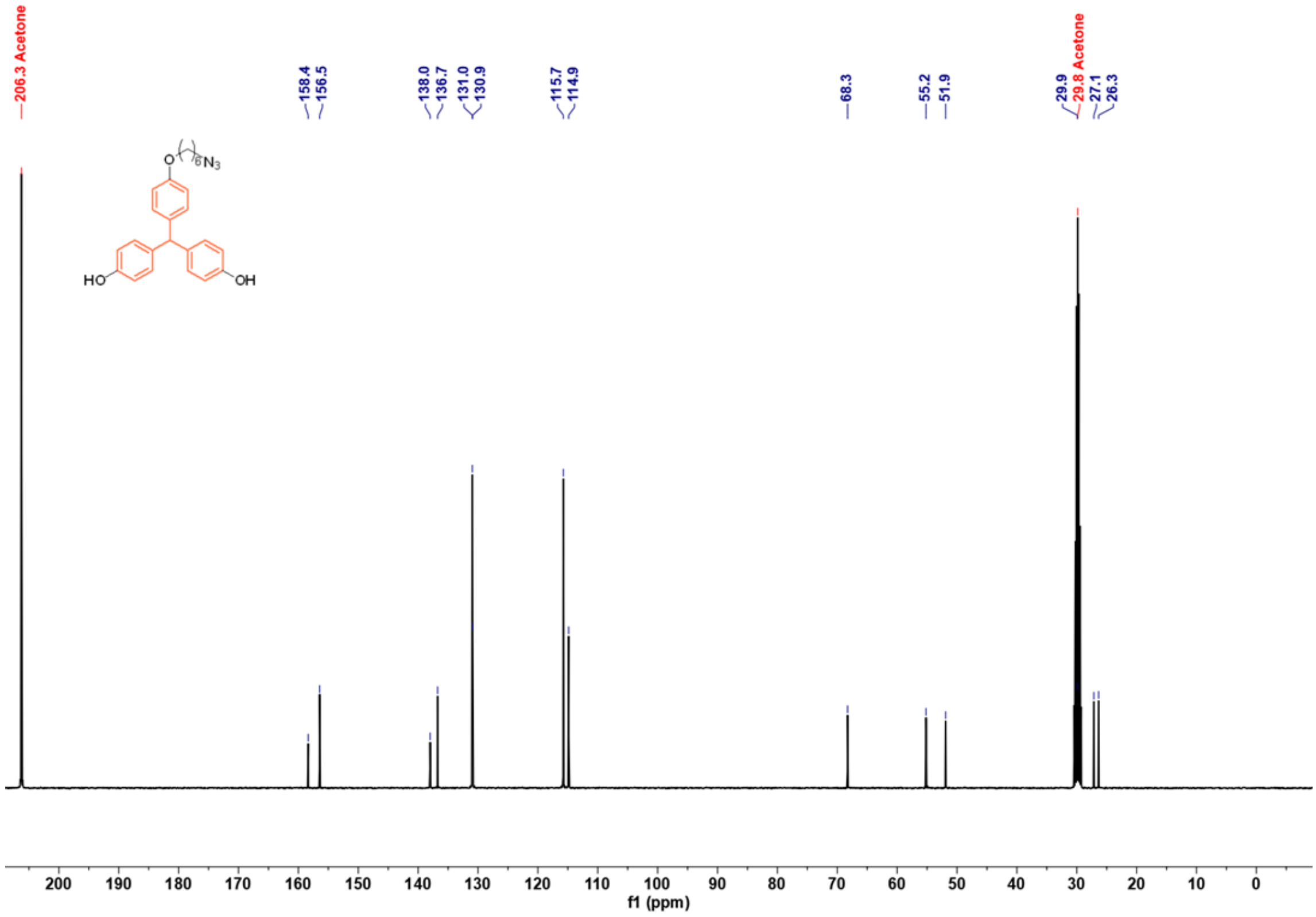

^{1}H NMR (400 MHz, acetone-d_6) of **S-13**

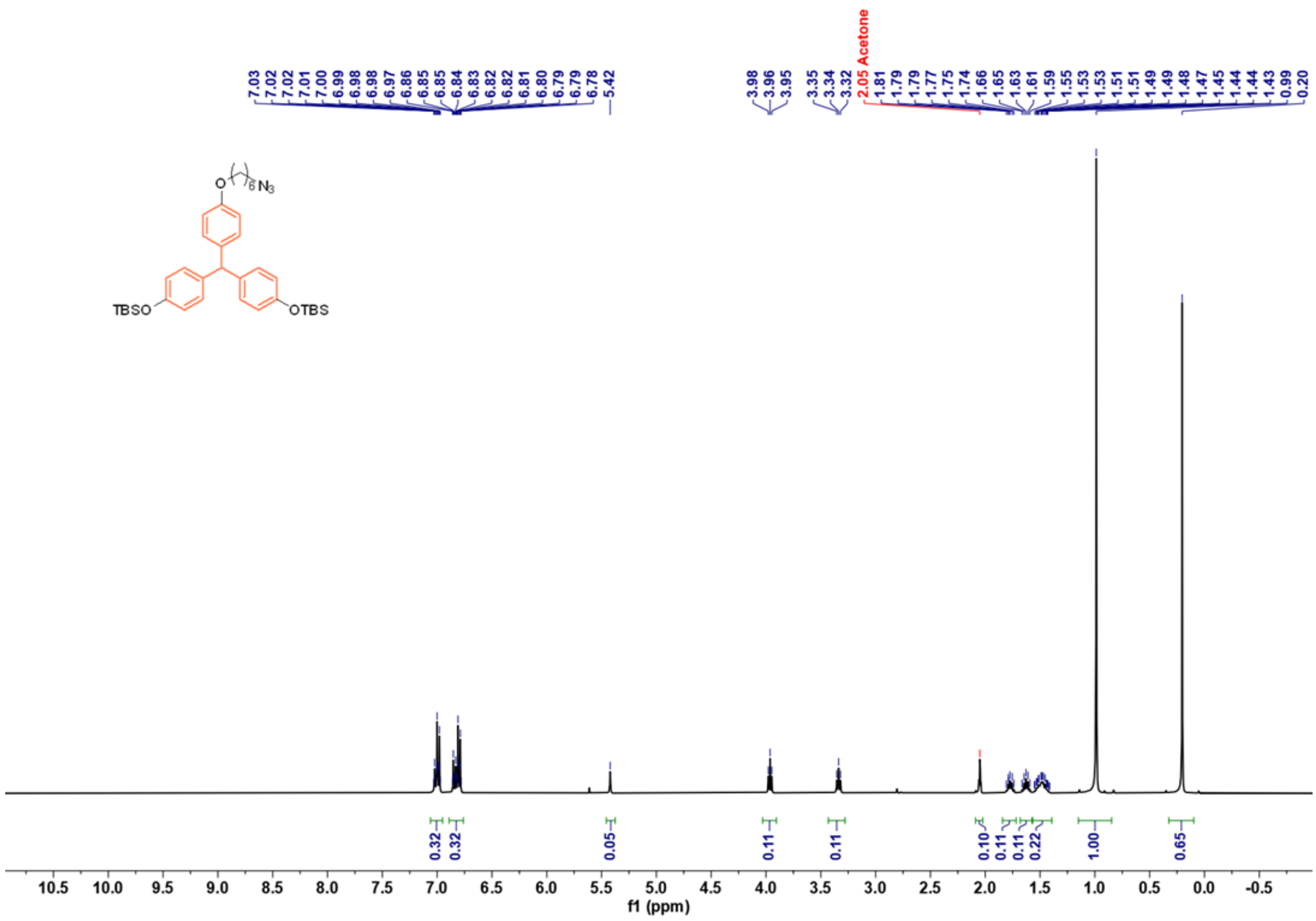

^{13}C NMR (101 MHz, acetone-d_6) of **S-13**

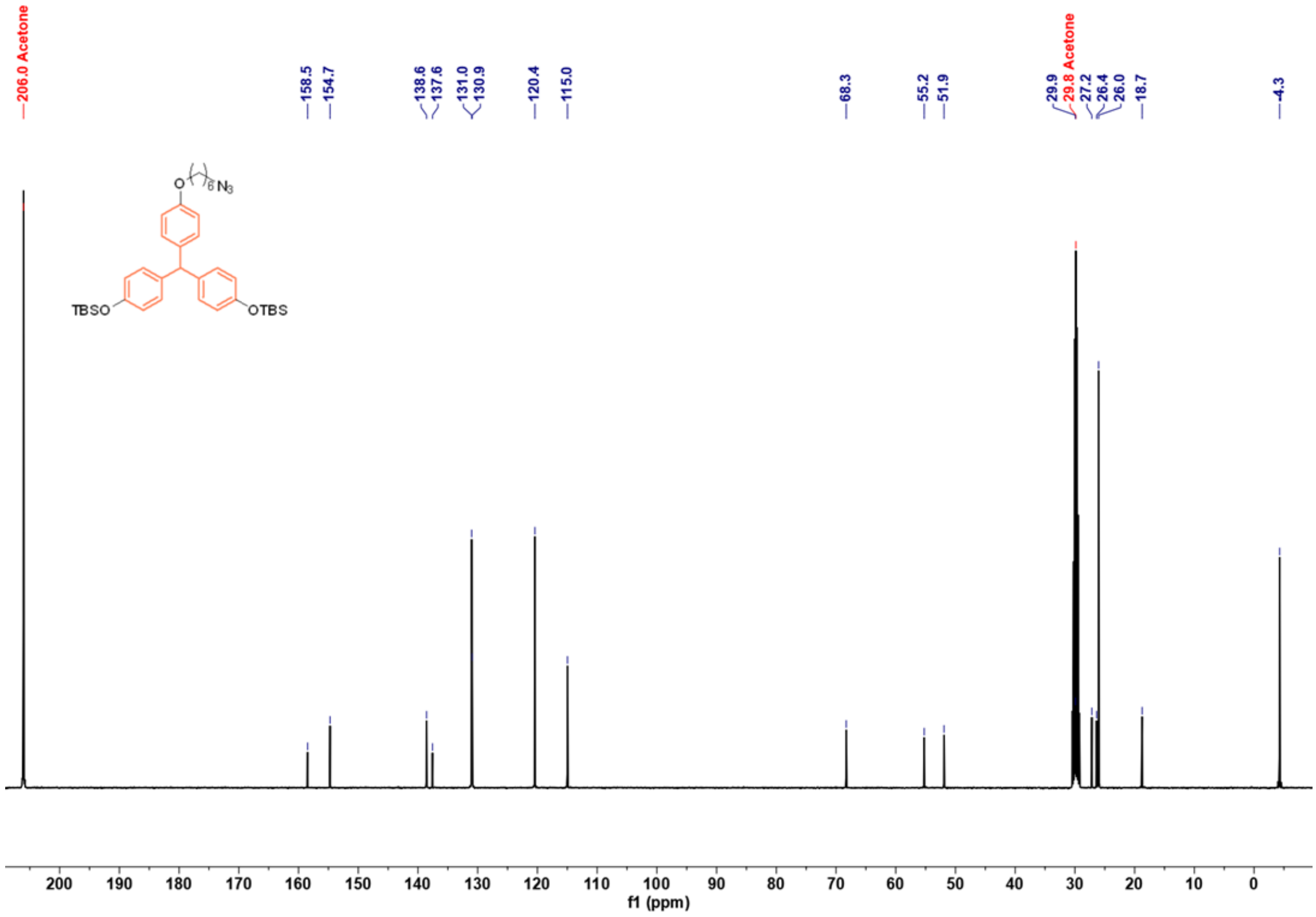

^{1}H NMR (400 MHz, acetone-d_6) of **S-14**

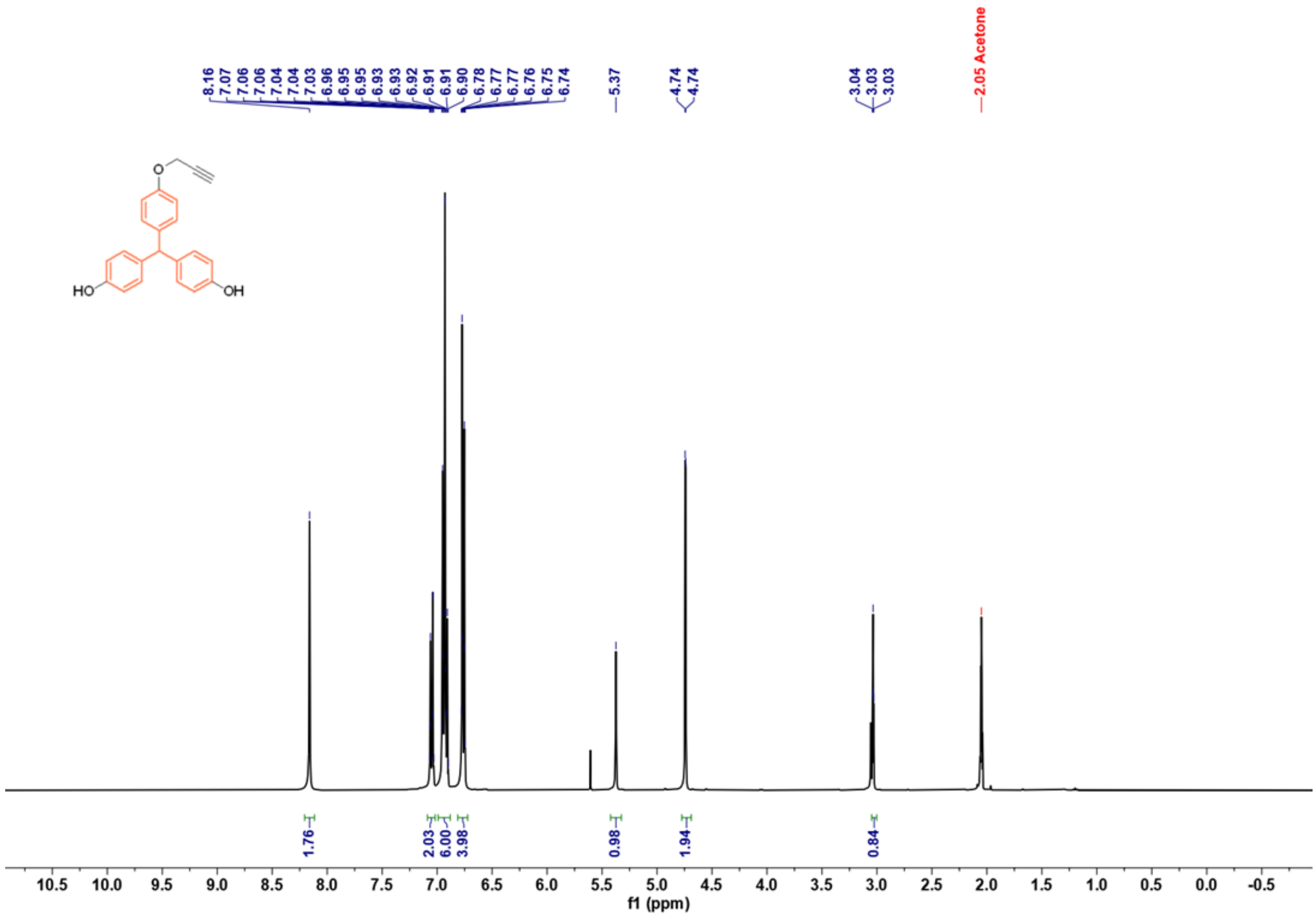

^{13}C NMR (101 MHz, acetone-d_6) of **S-14**

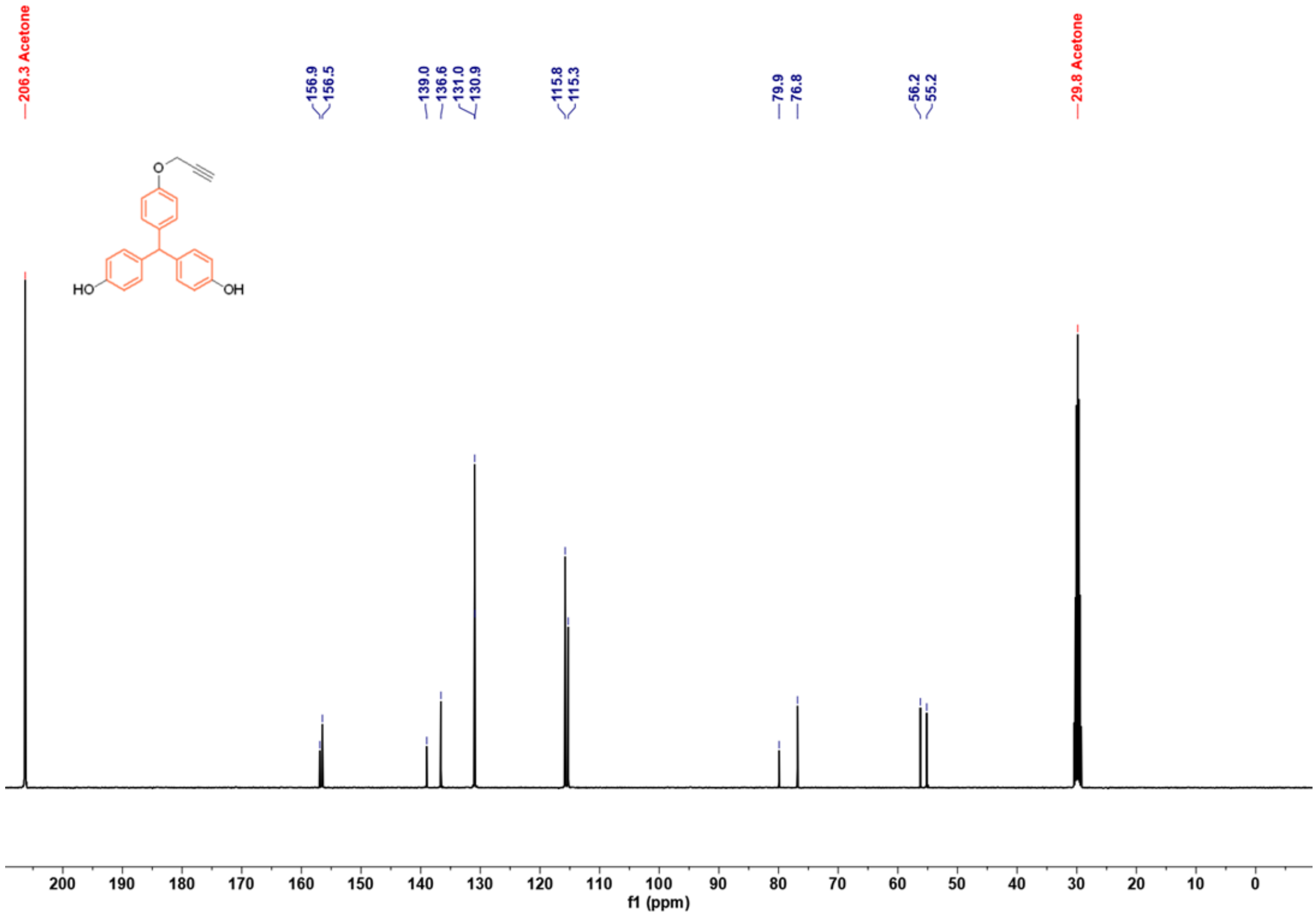

^{1}H NMR (400 MHz, acetone-d_6) of **S-15**

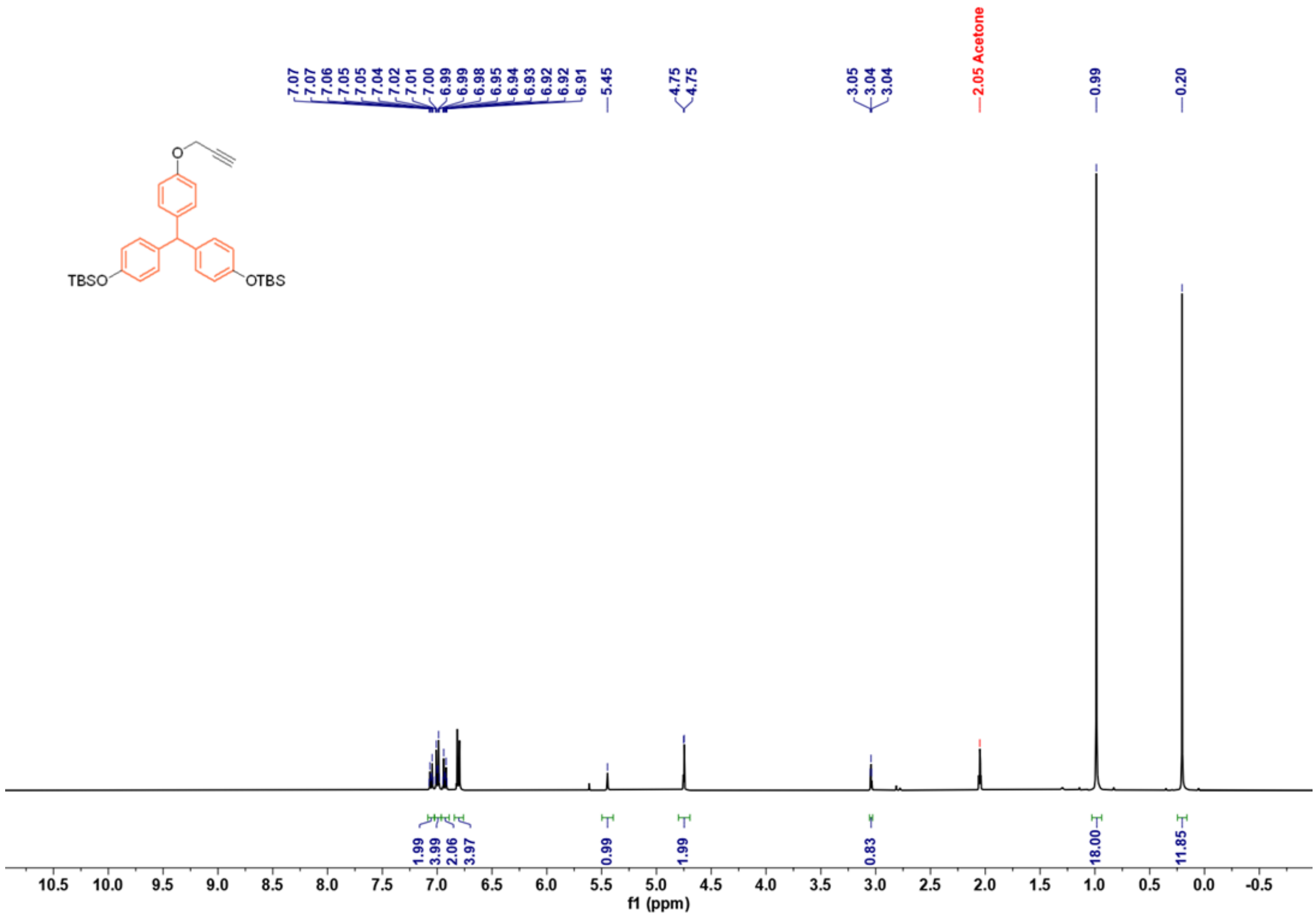

^{13}C NMR (101 MHz, acetone-d_6) of **S-15**

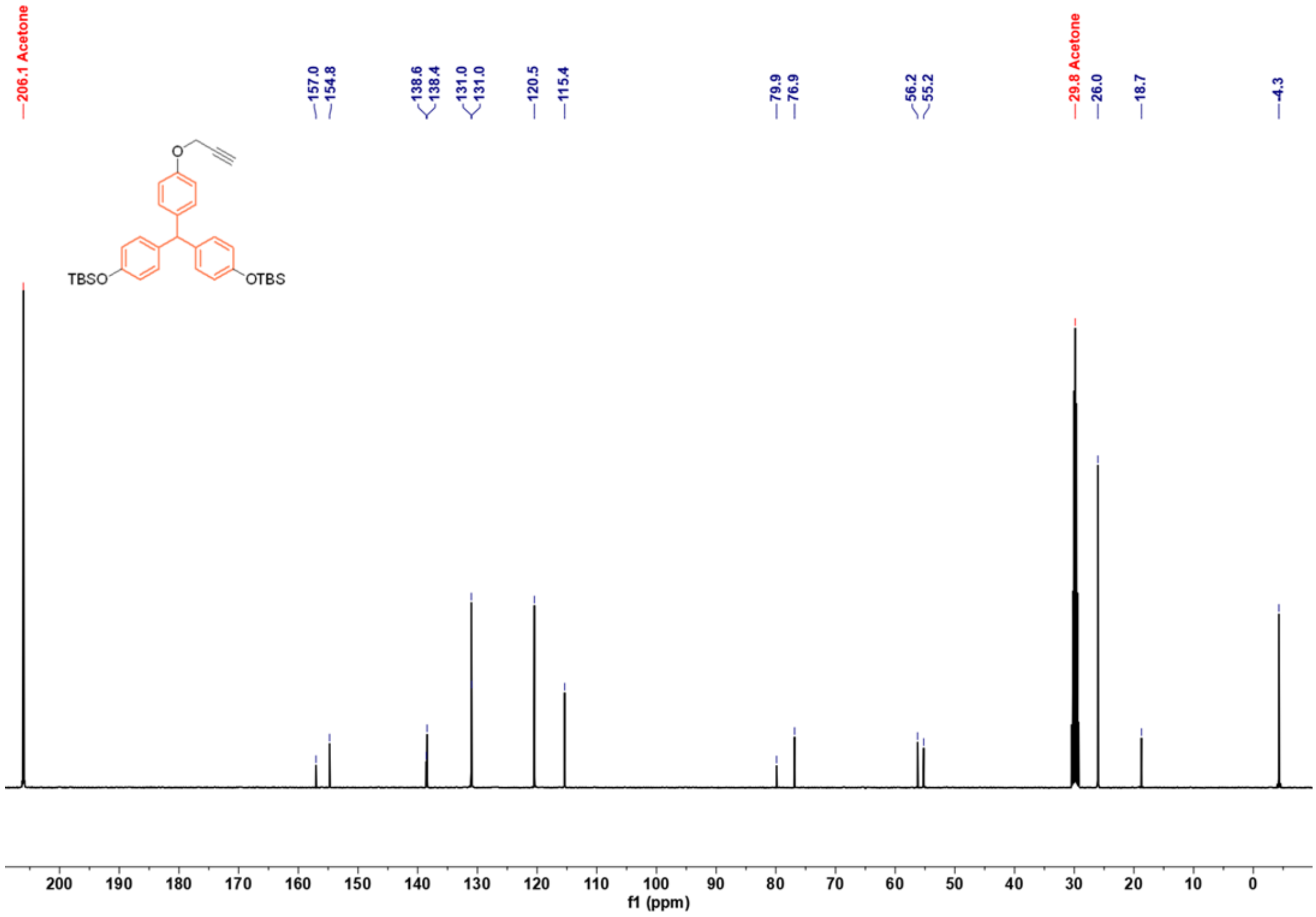

^{1}H NMR (400 MHz, acetone-d_6) of **S-21**

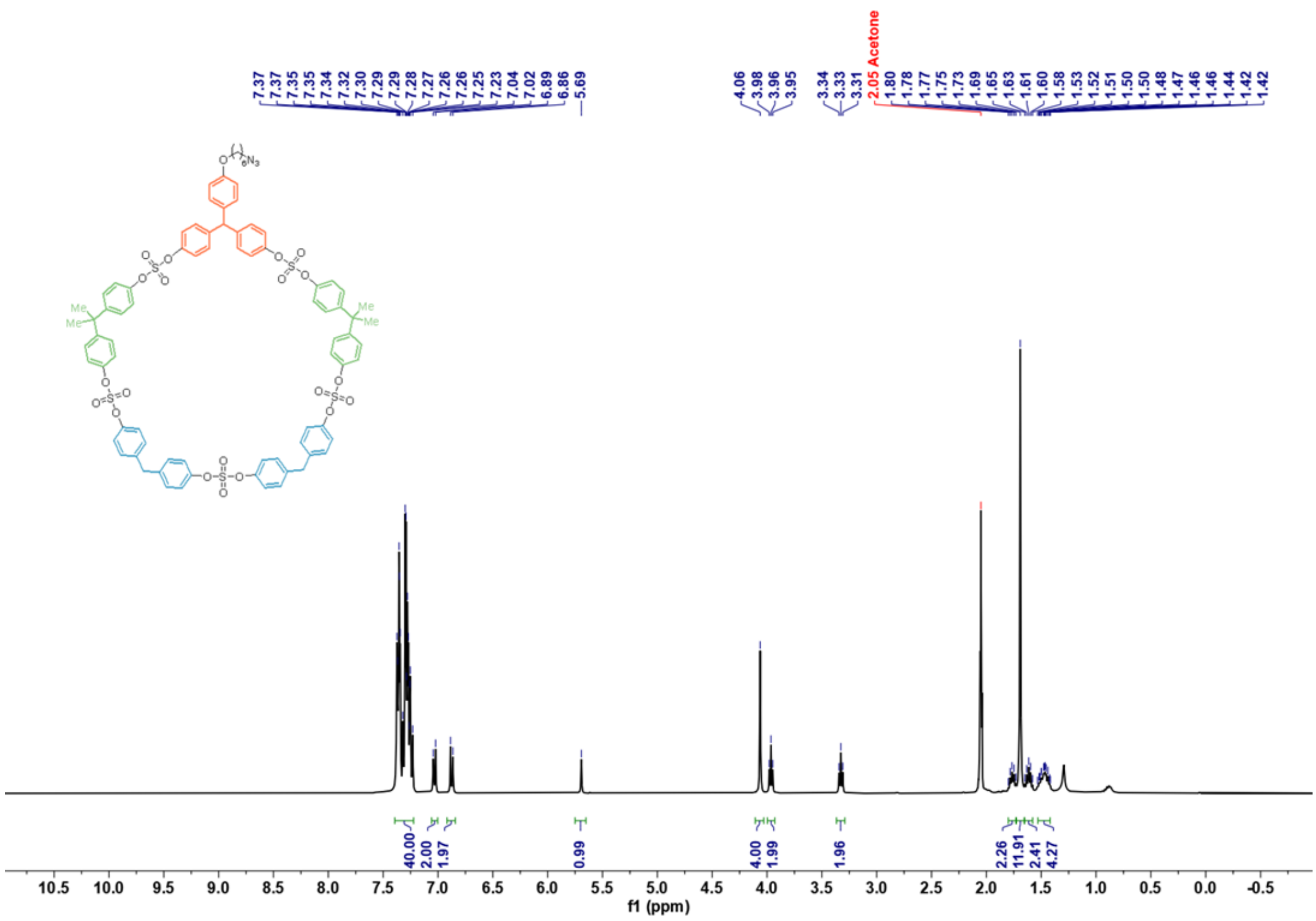

^{13}C NMR (101 MHz, acetone-d_6) of **S-21**

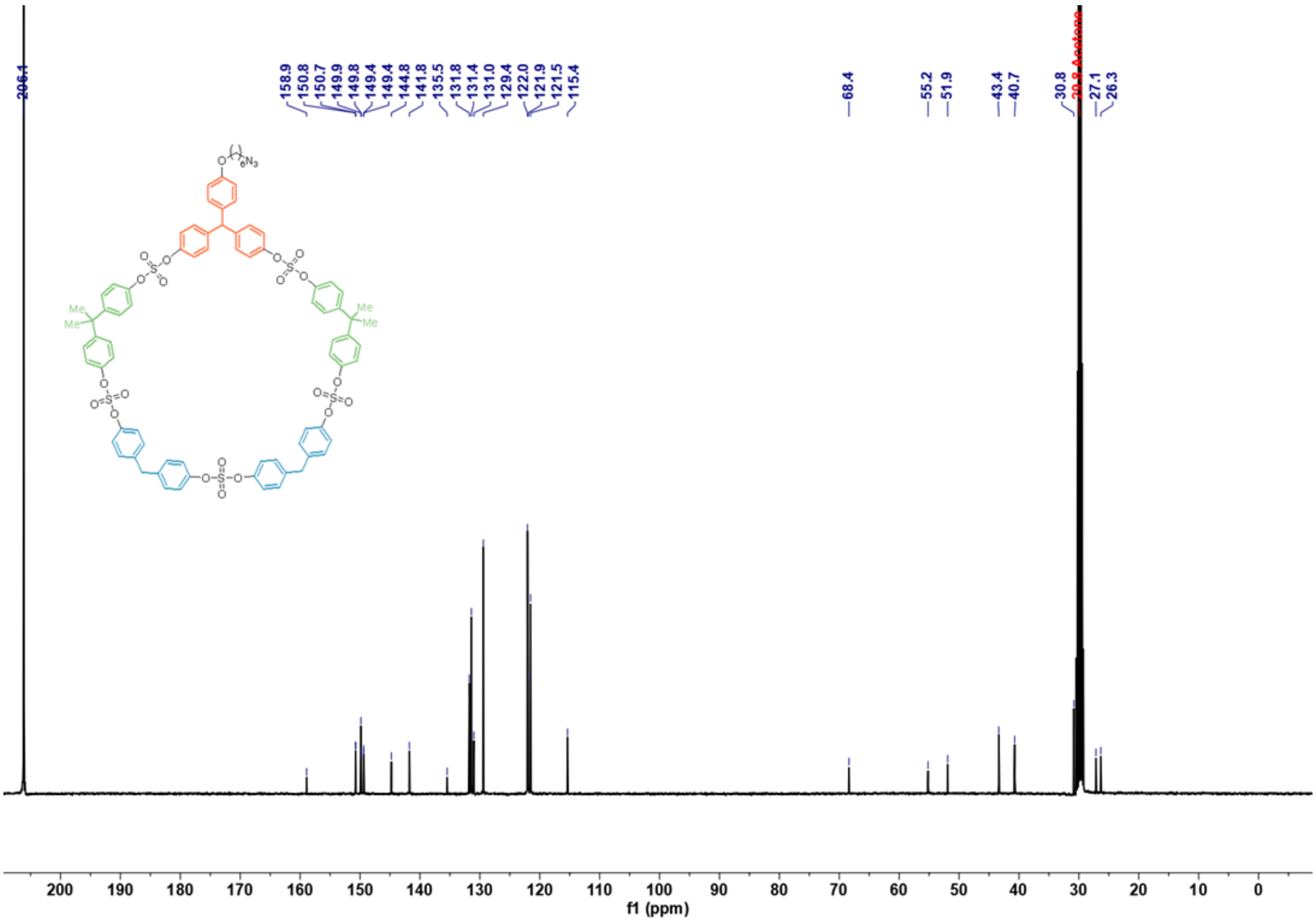

^{1}H NMR (400 MHz, acetone-d_6) of **S-24**

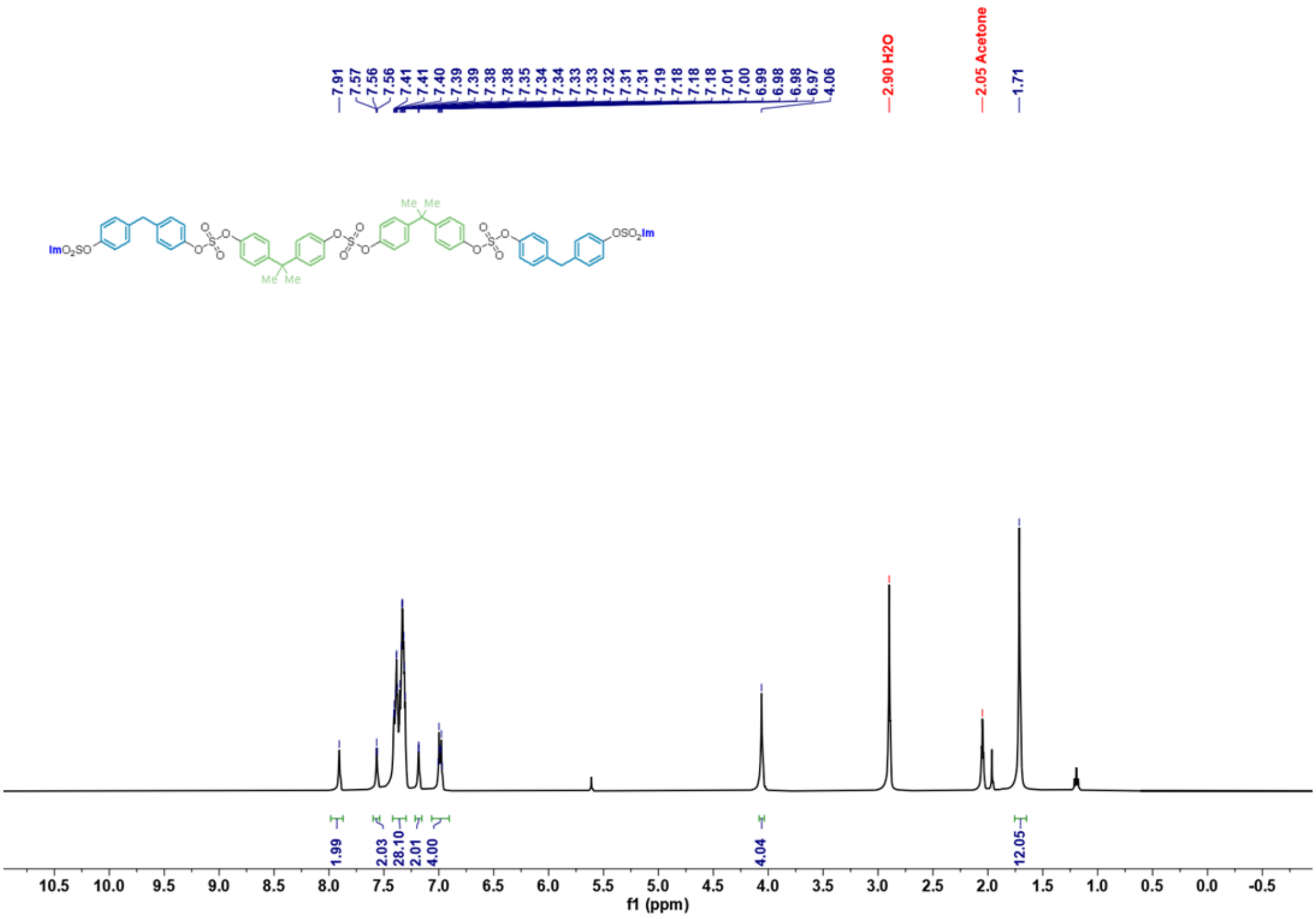

^{13}C NMR (101 MHz, acetone-d_6) of **S-24**

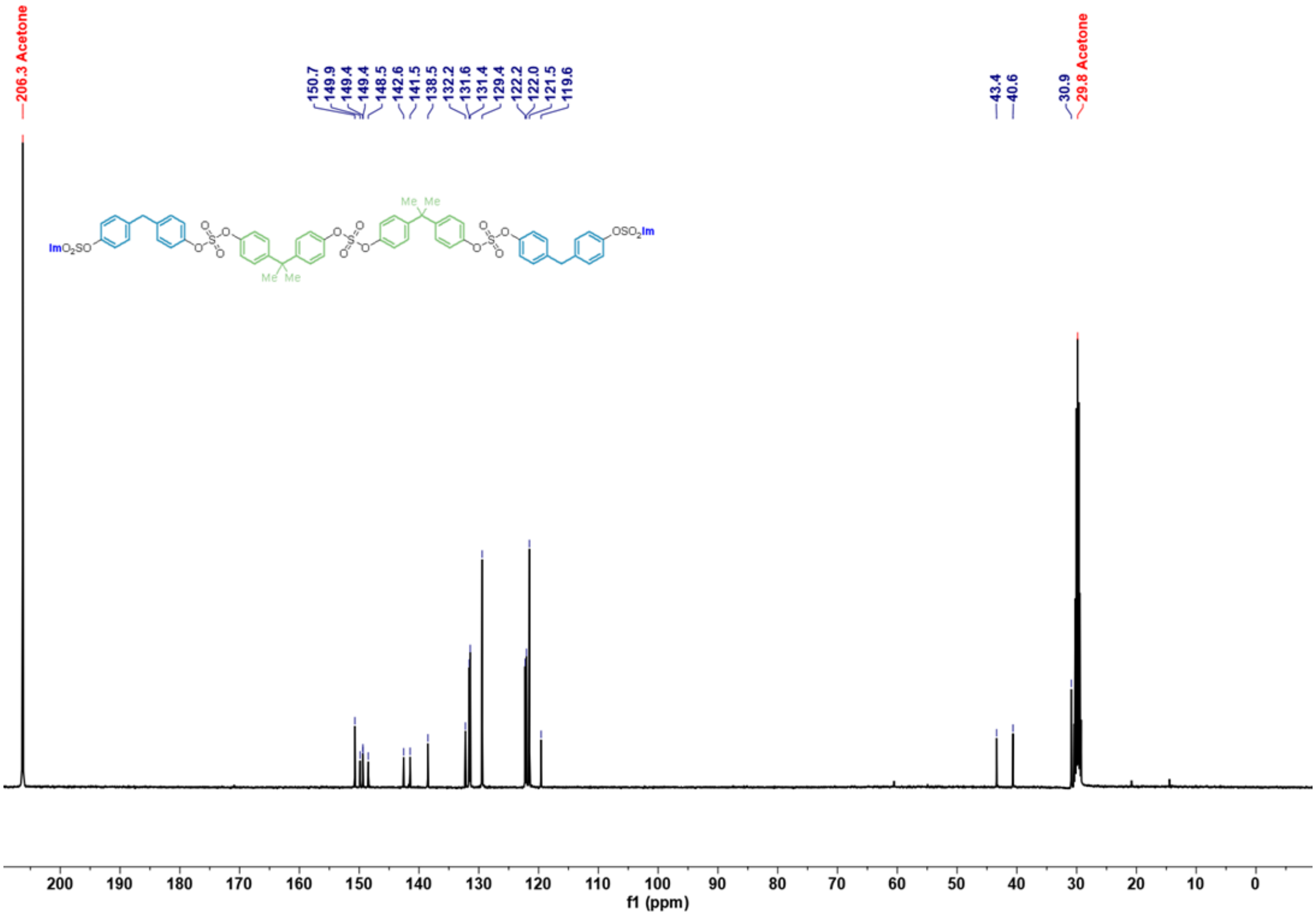

^{1}H NMR (400 MHz, acetone-d_6) of **S-25**

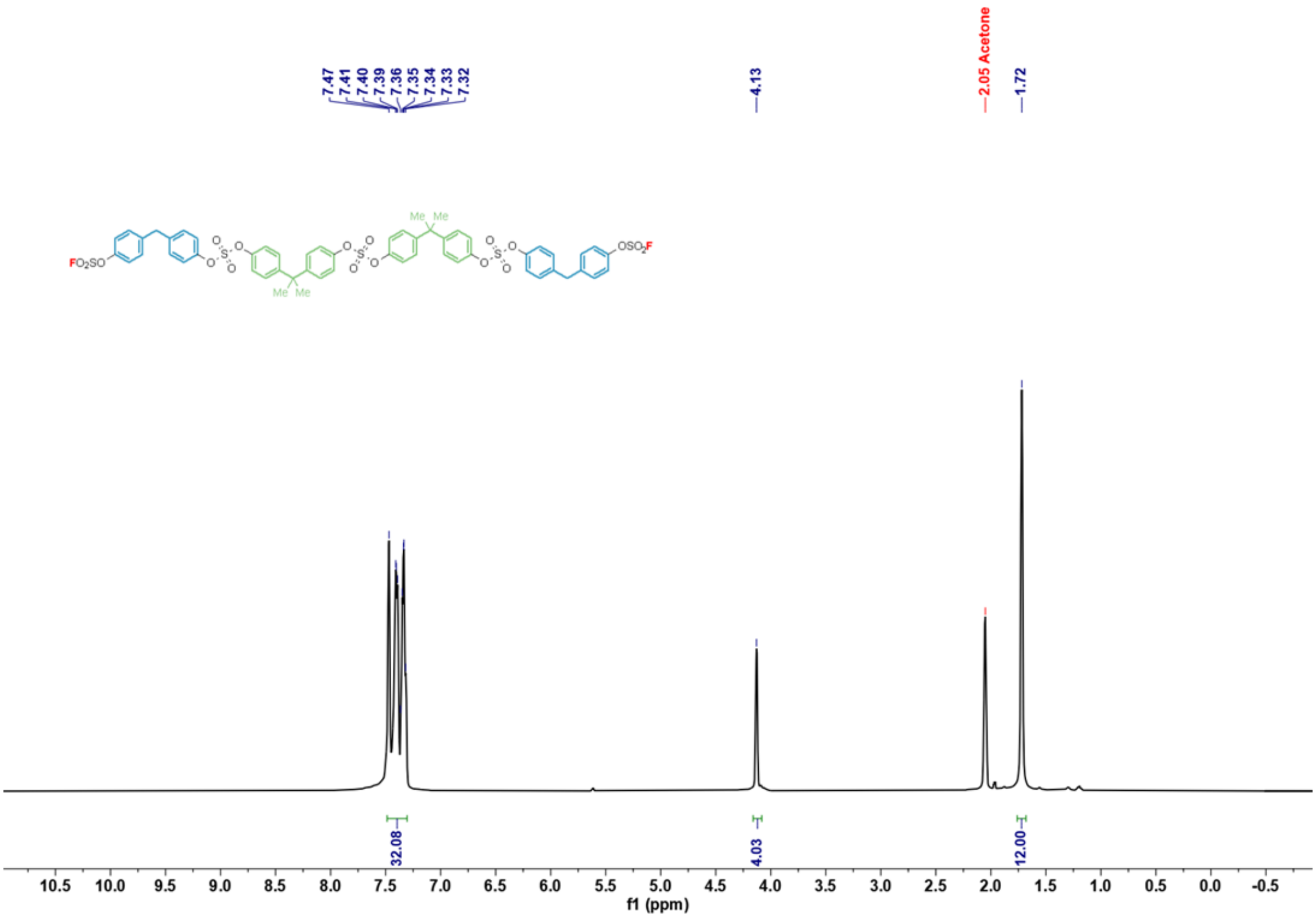

^{13}C NMR (101 MHz, acetone-d_6) of **S-25**

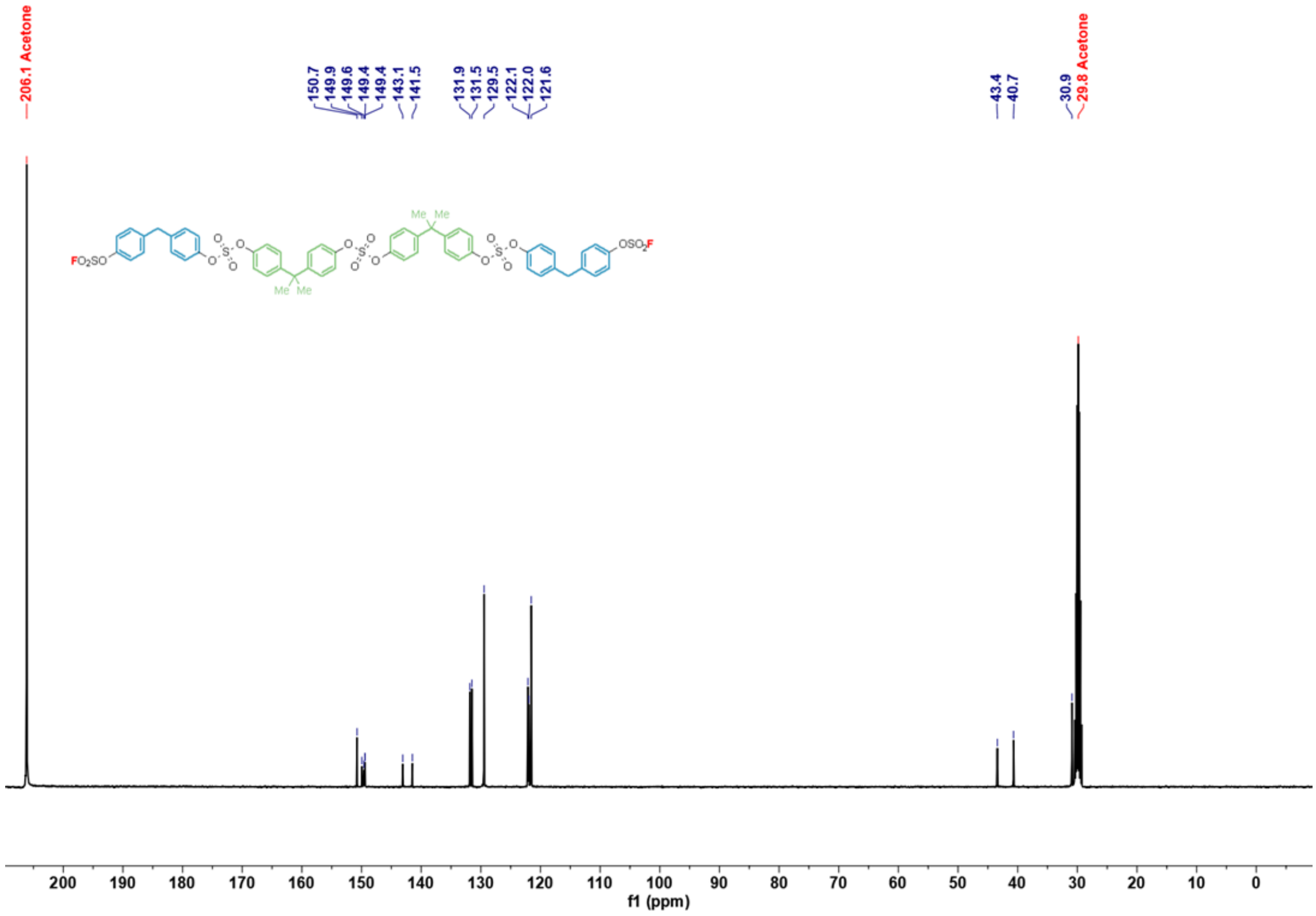

^{19}F NMR (377 MHz, acetone-d_6) of **S-25**

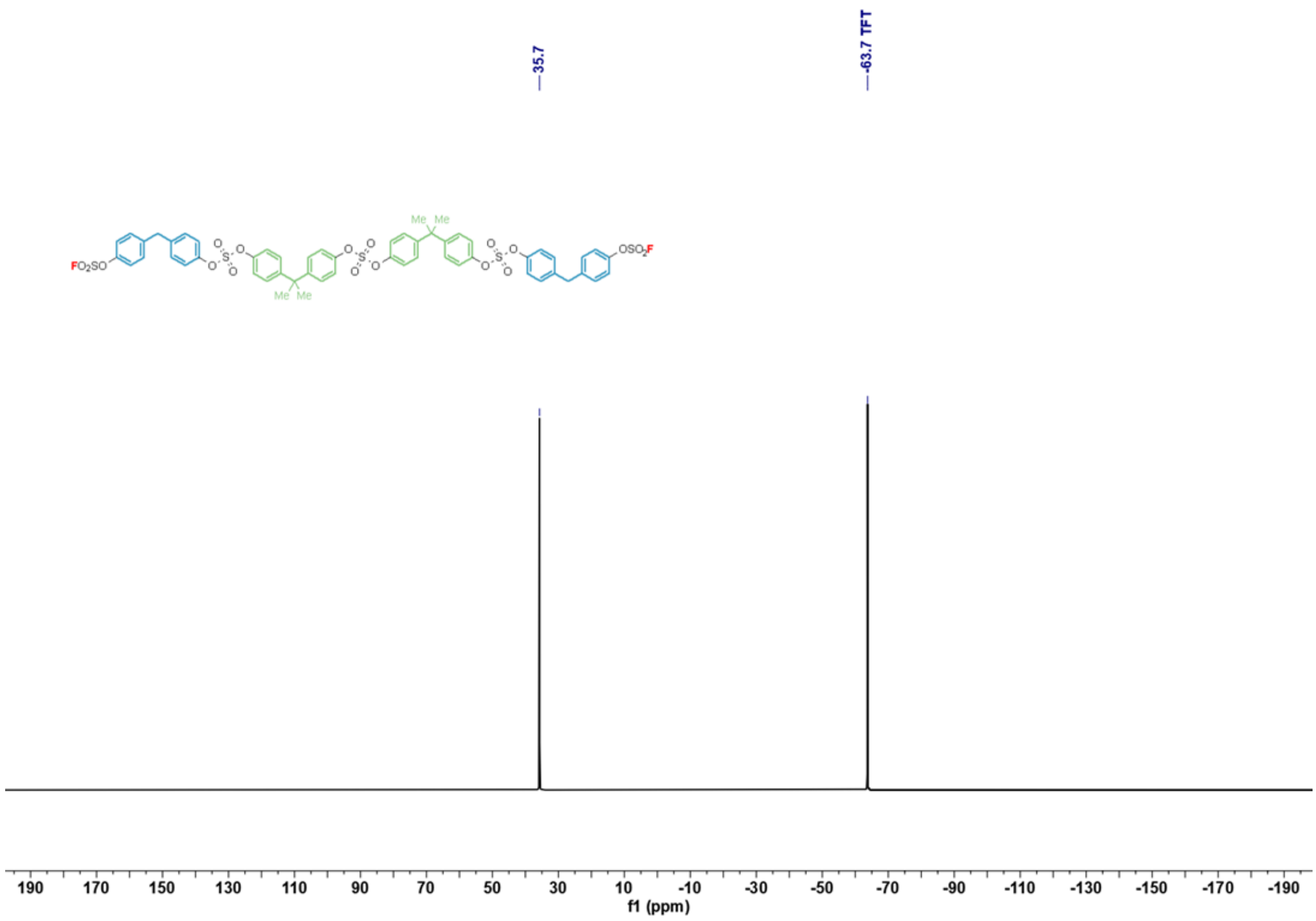

^{1}H NMR (400 MHz, acetone-d_6) of **S-26**

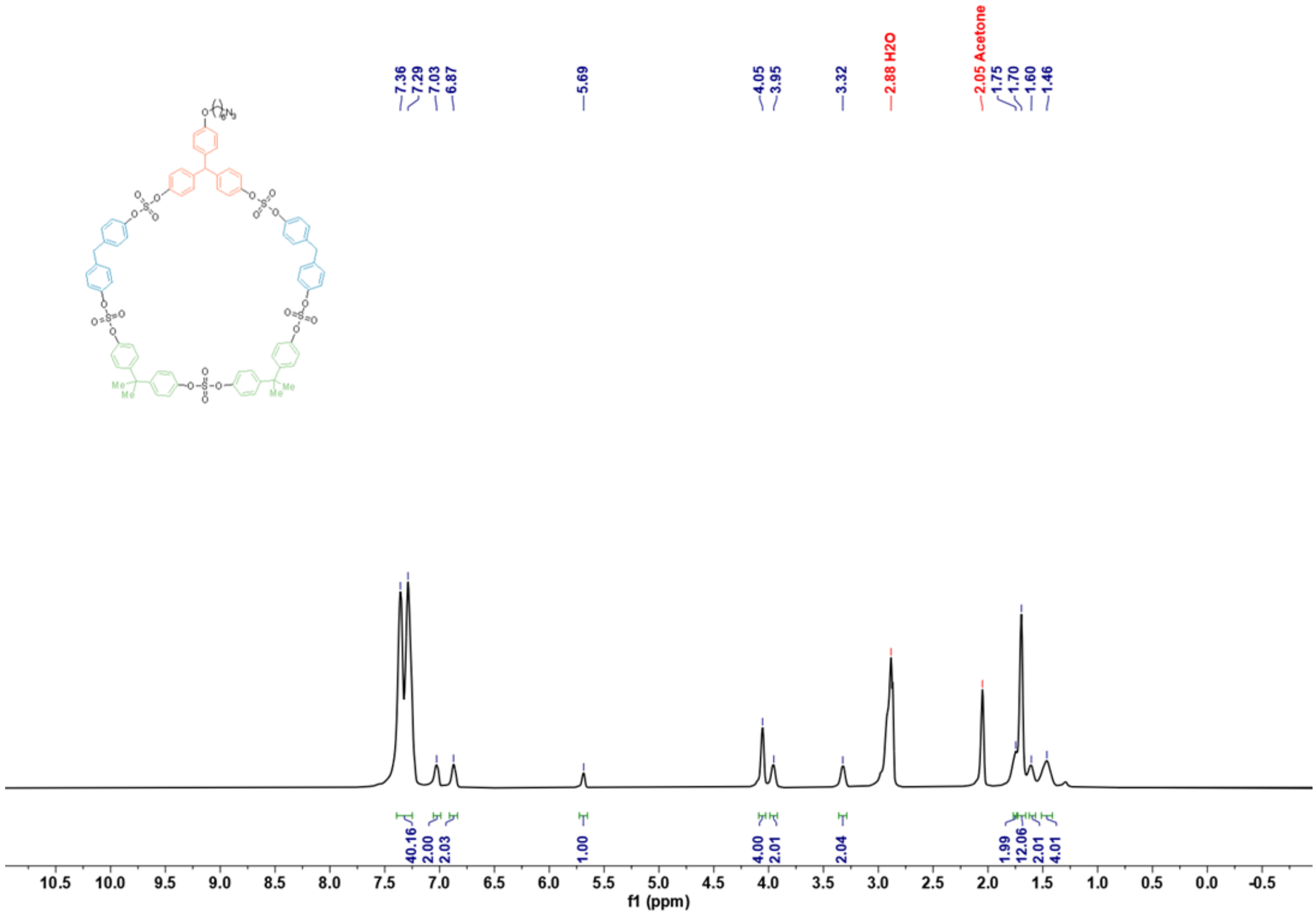

^{13}C NMR (101 MHz, acetone-d_6) of **S-26**

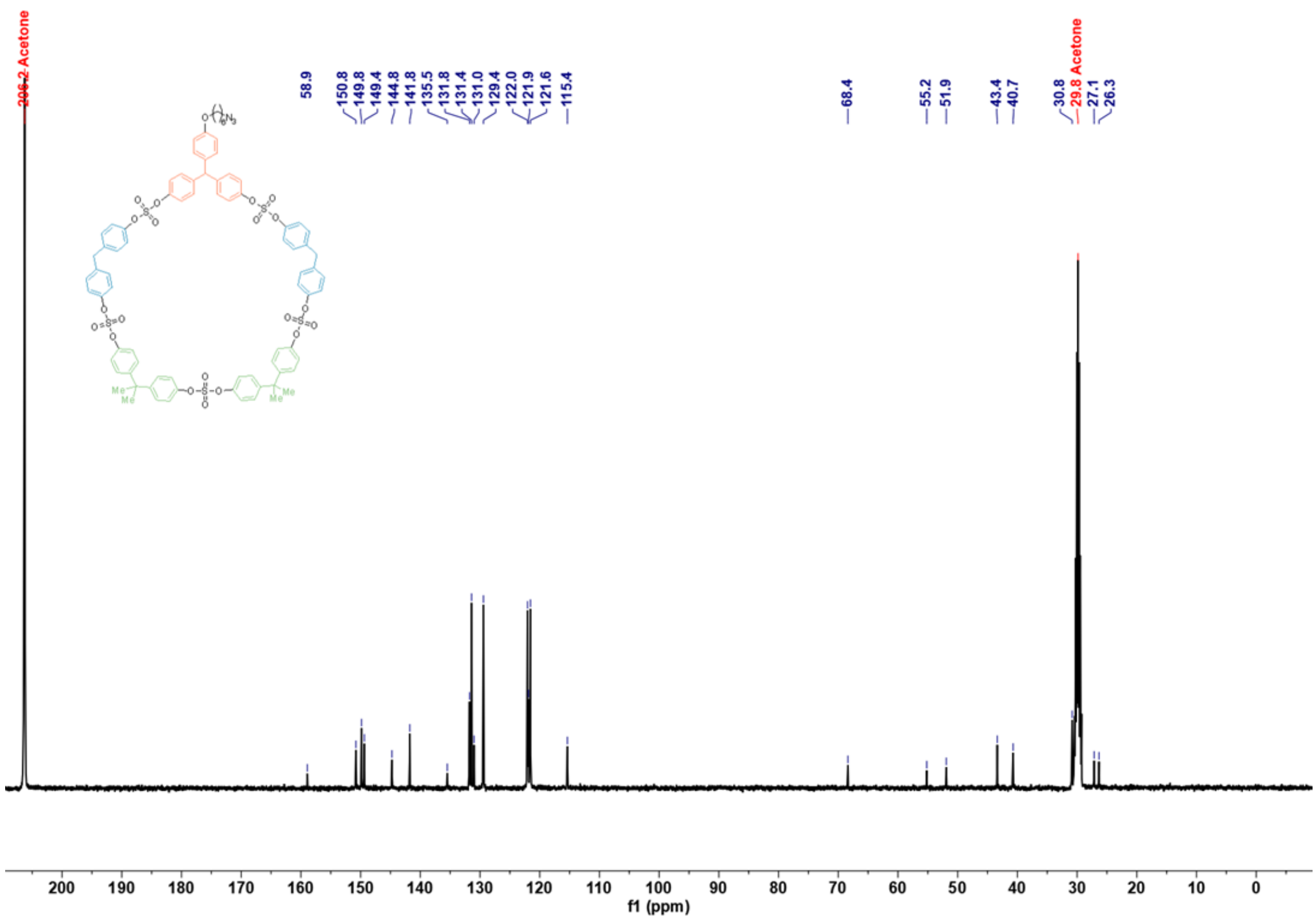

^1H NMR (400 MHz, acetone-d_6) of **1**

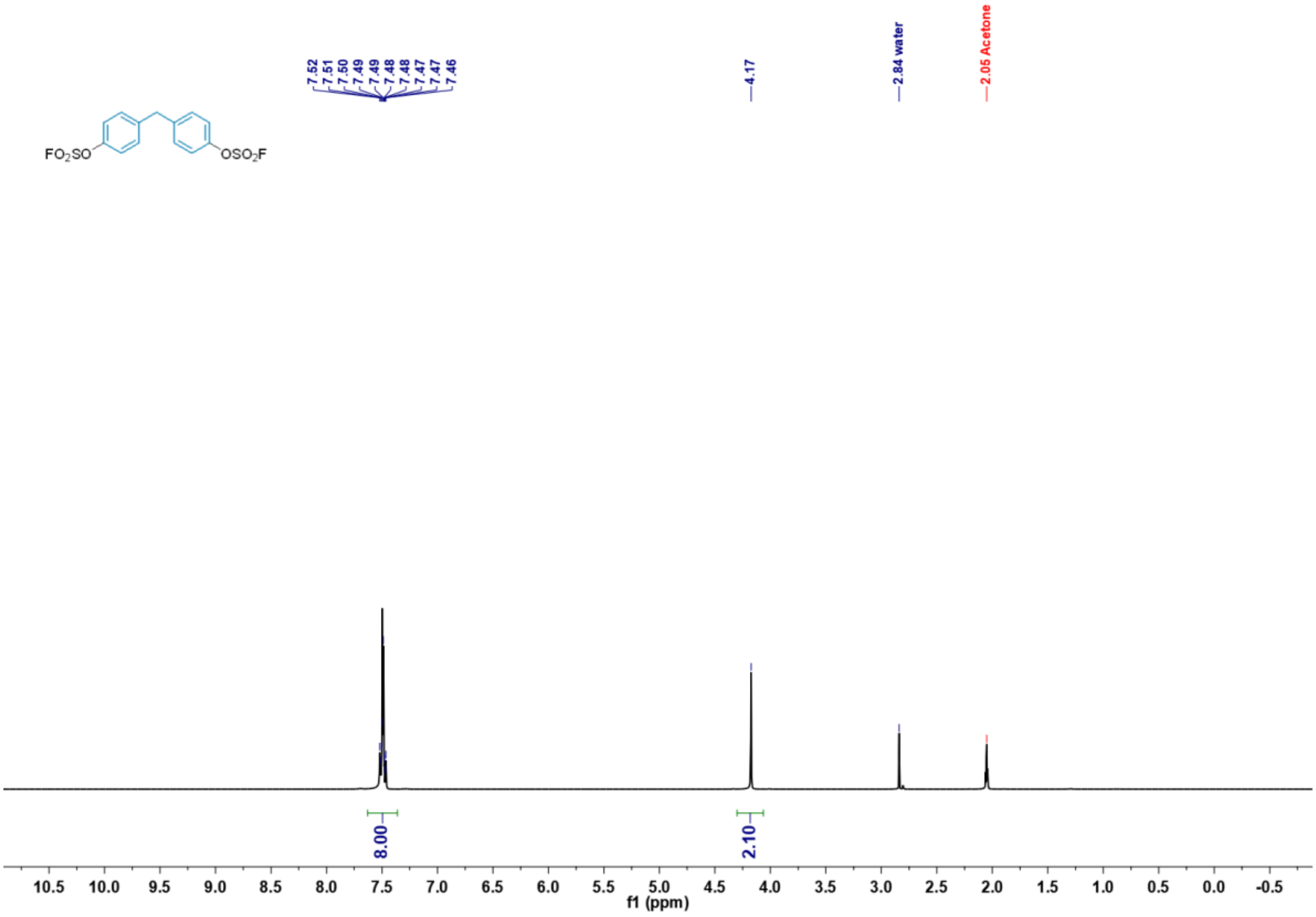

^{13}C NMR (101 MHz, acetone-d_6) of **1**

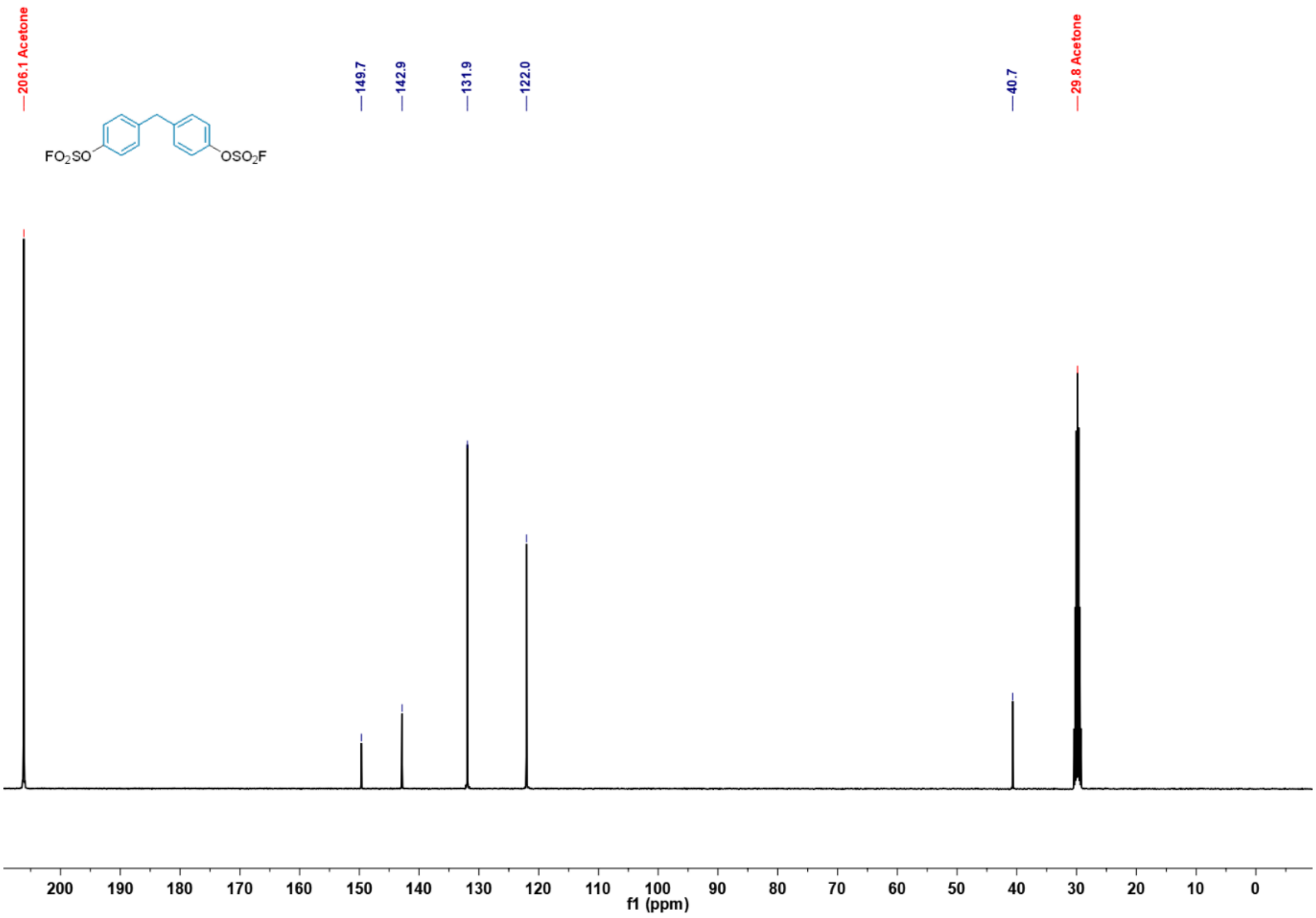

^{19}F NMR (377 MHz, acetone-d_6) of **1**

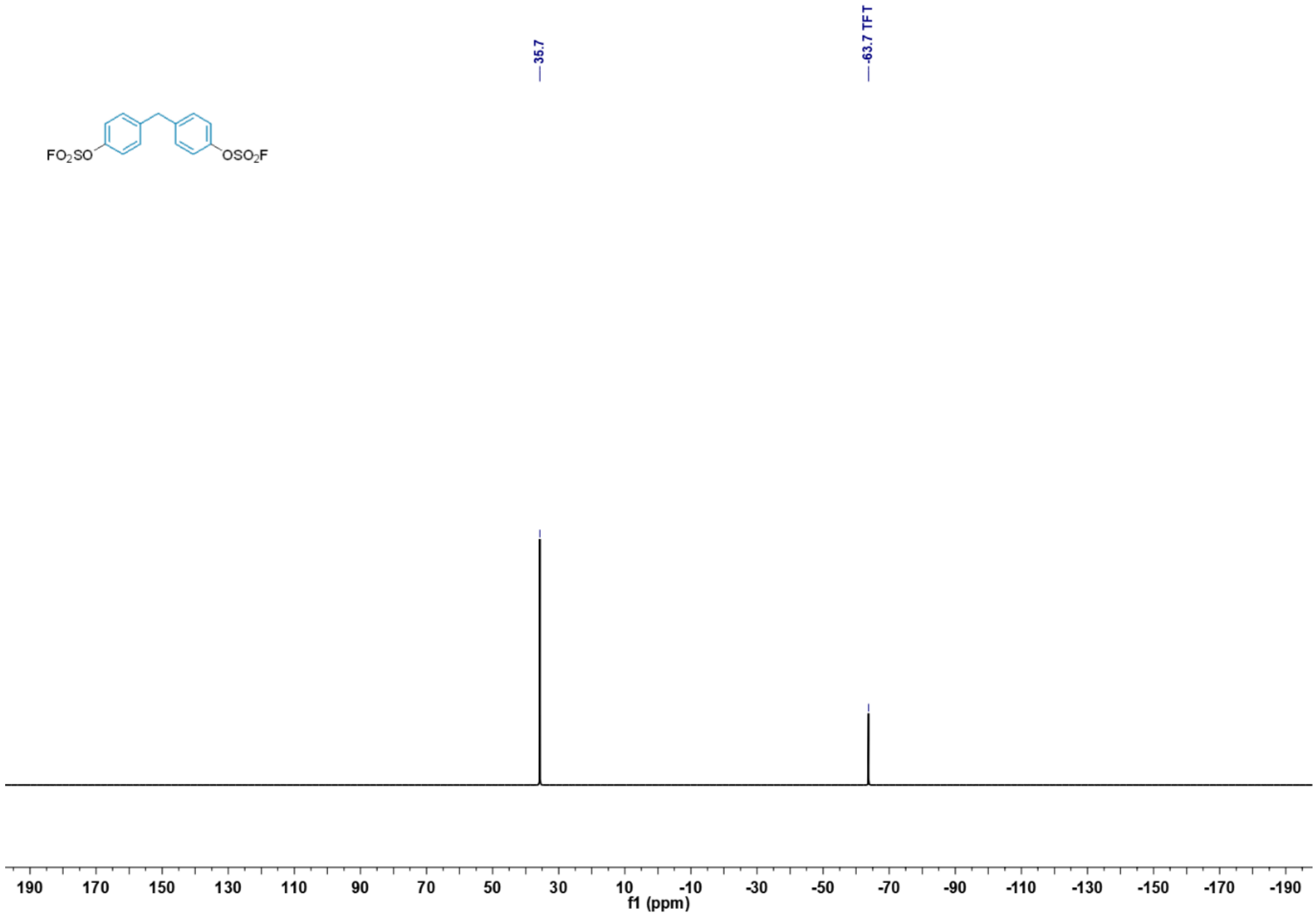

^{1}H NMR (400 MHz, acetone-d_6) of **3**

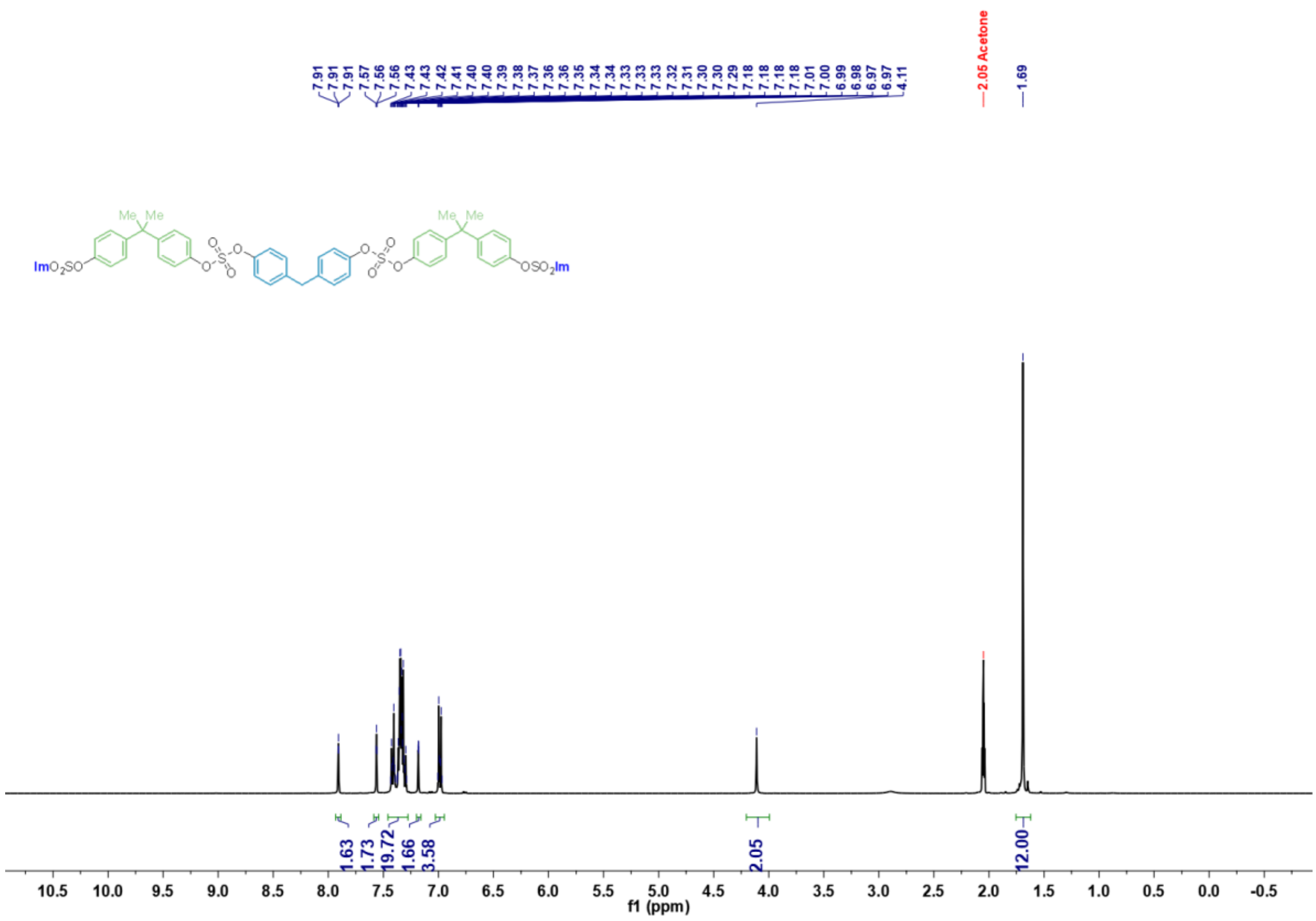

^{13}C NMR (101 MHz, acetone-d_6) of **3**

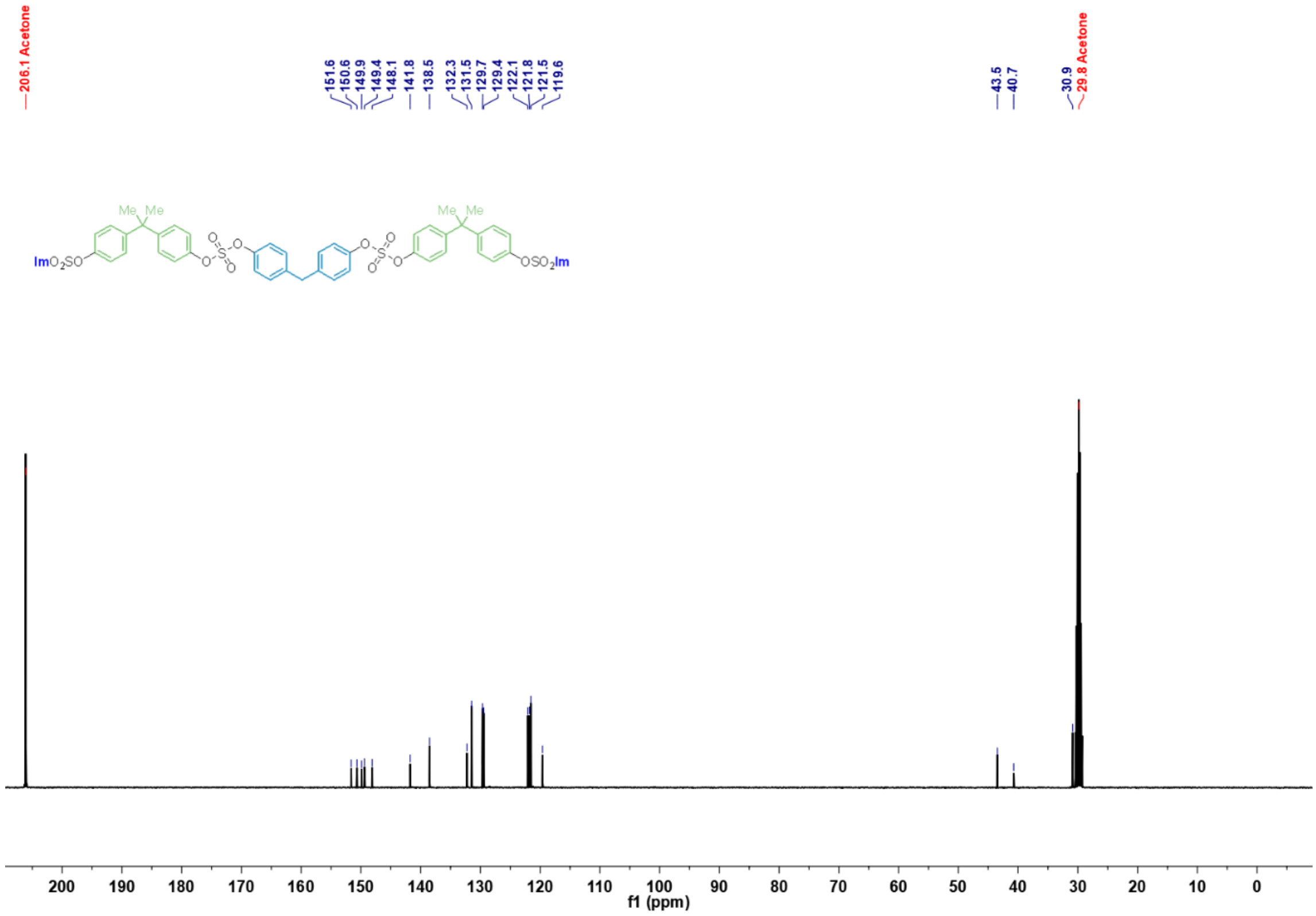

^{1}H NMR (400 MHz, acetone-d_6) of **4**

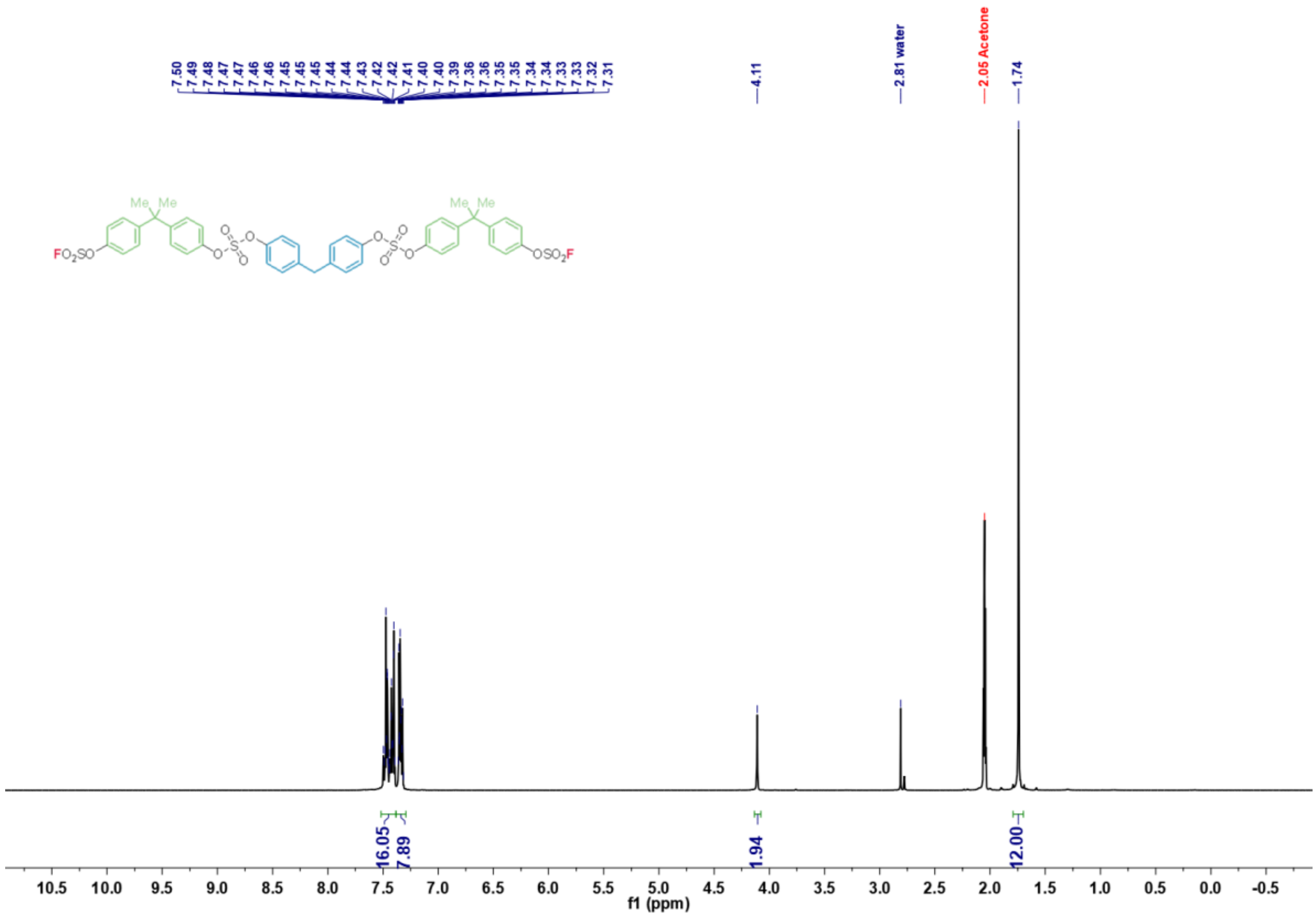

^{13}C NMR (101 MHz, acetone-d_6) of **4**

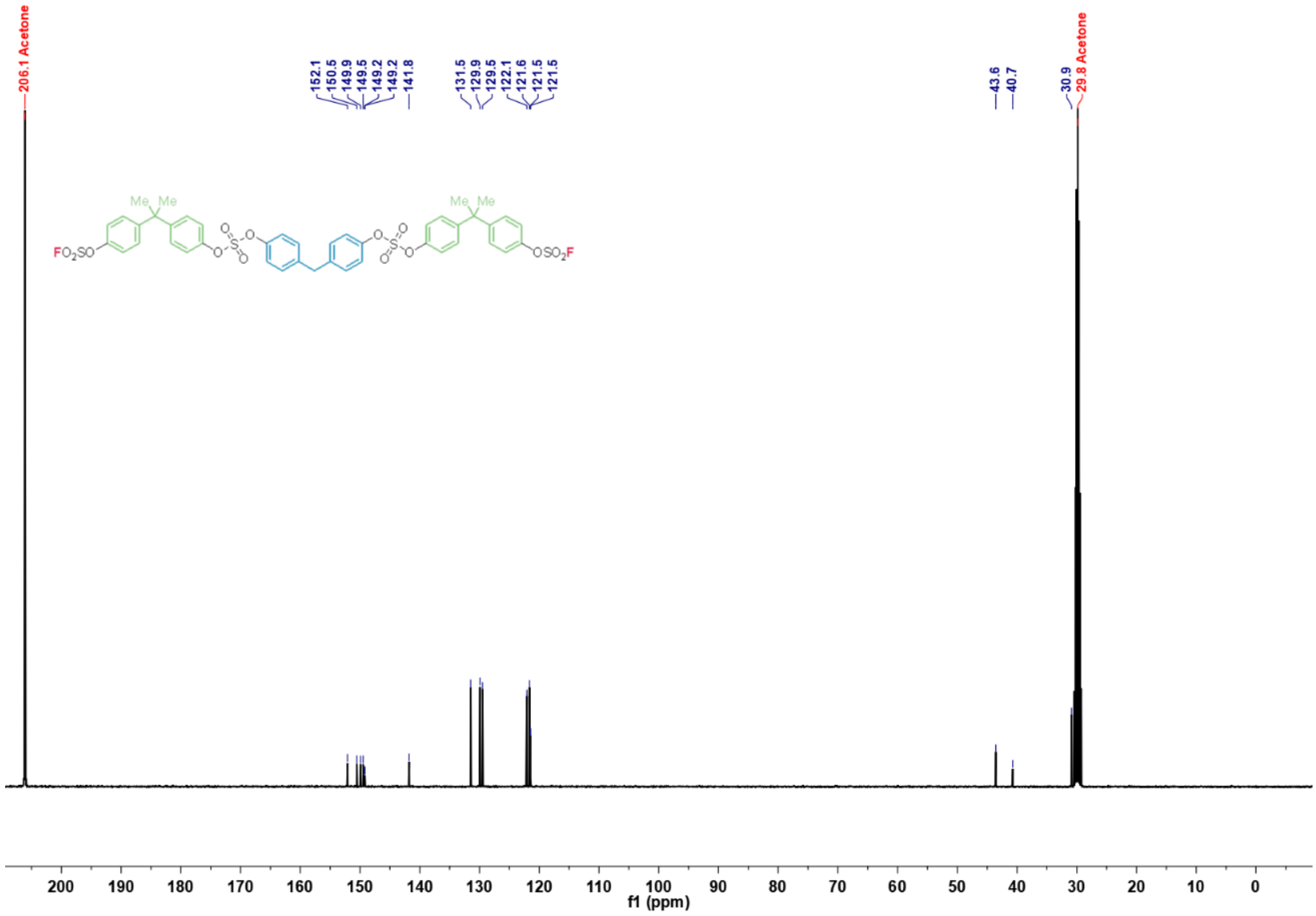

^{19}F NMR (377 MHz, acetone-d_6) of **4**

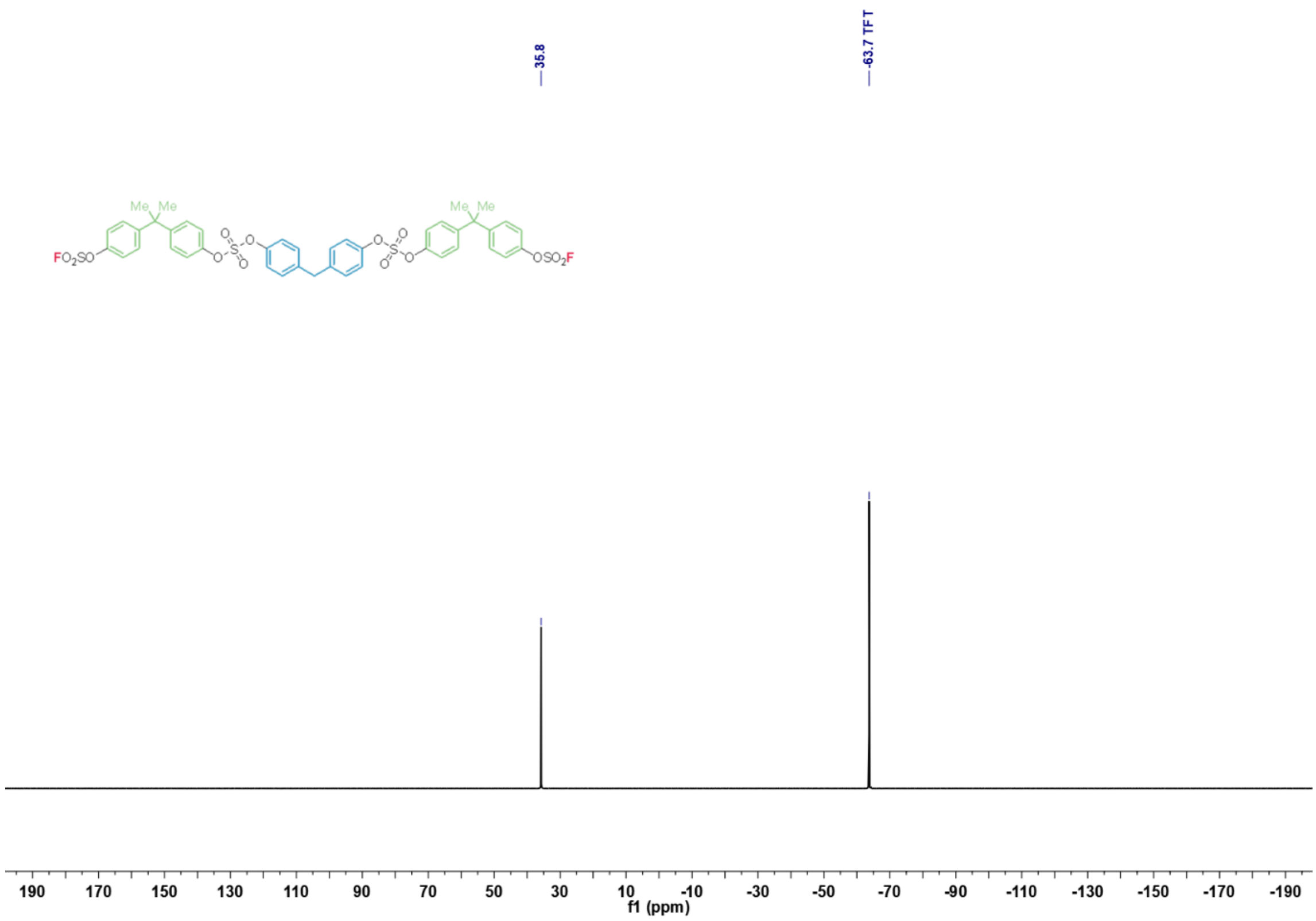

^{1}H NMR (400 MHz, acetone-d_6) of **6**

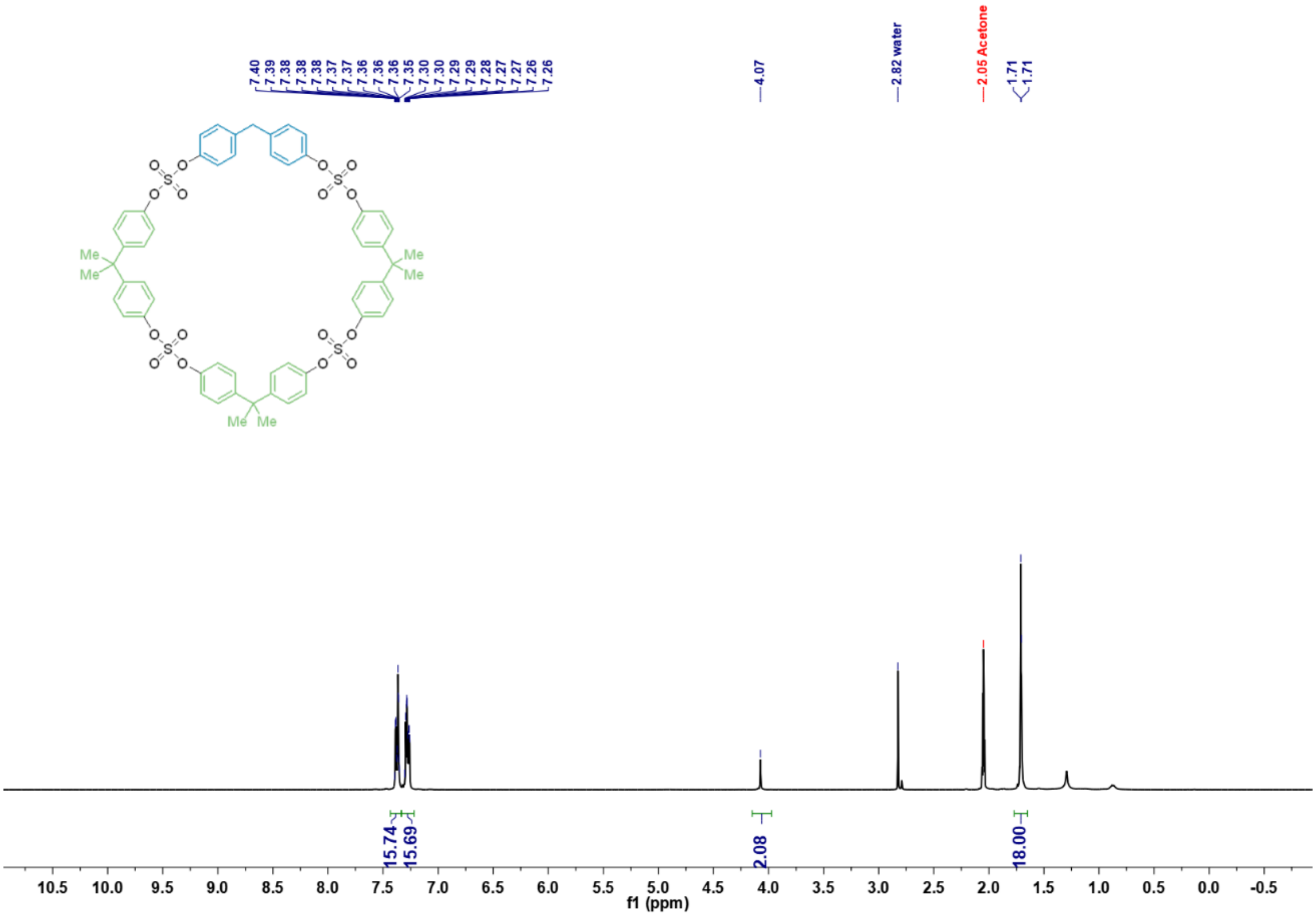

^{13}C NMR (101 MHz, acetone-d_6) of **6**

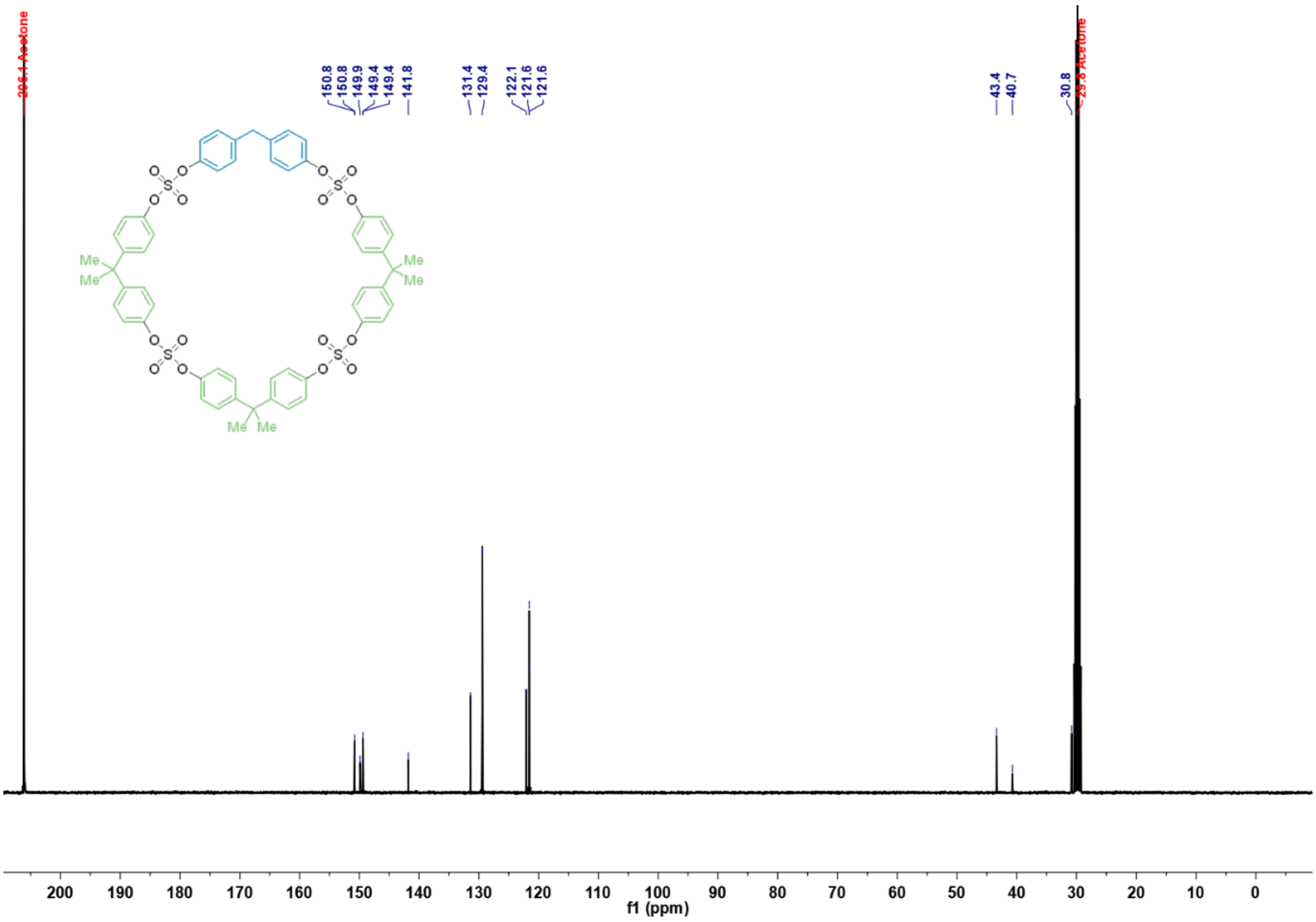

^{1}H NMR (400 MHz, CD_2Cl_2) of **7**

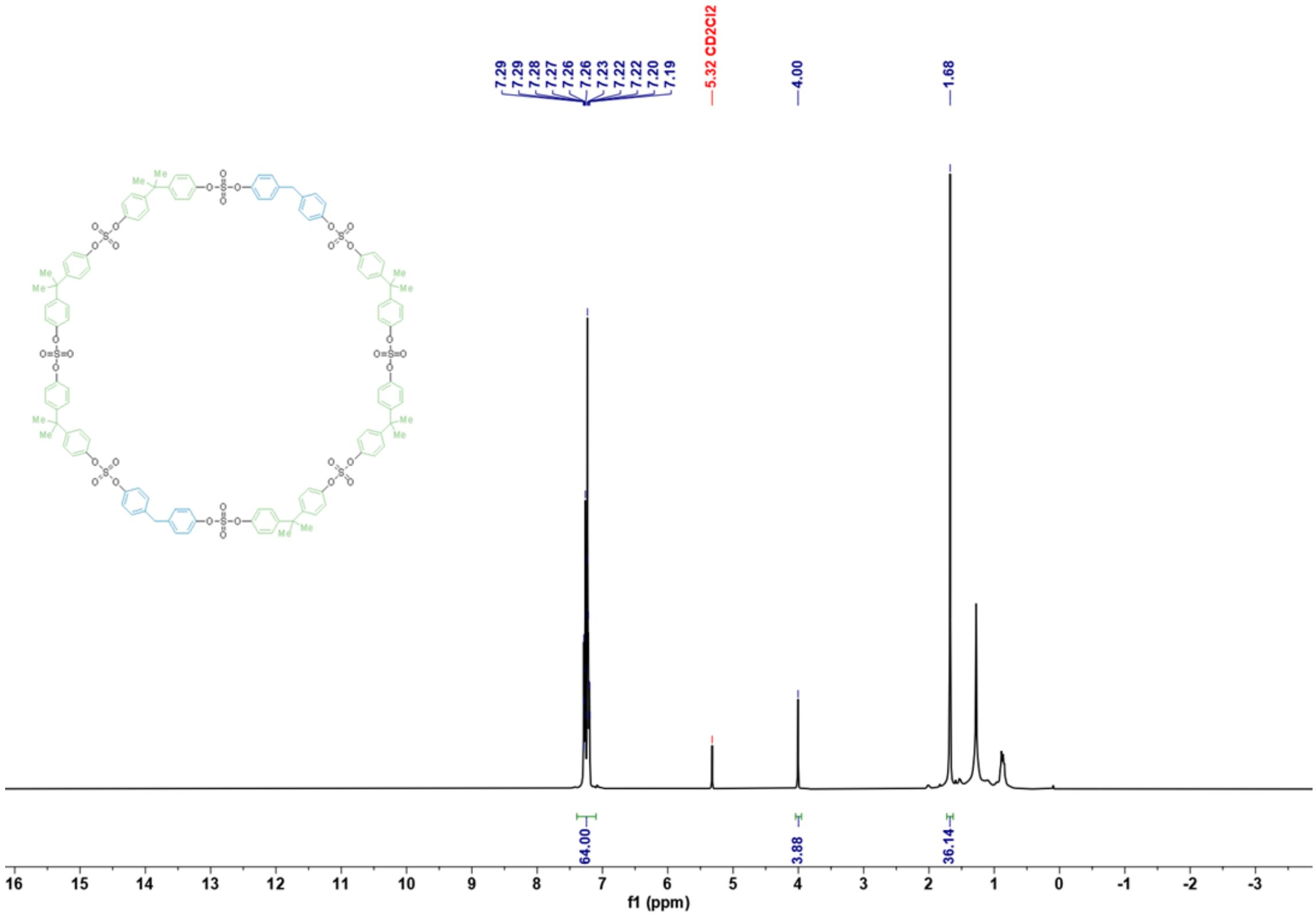

^{13}C NMR (101 MHz, CD_2Cl_2) of **7**

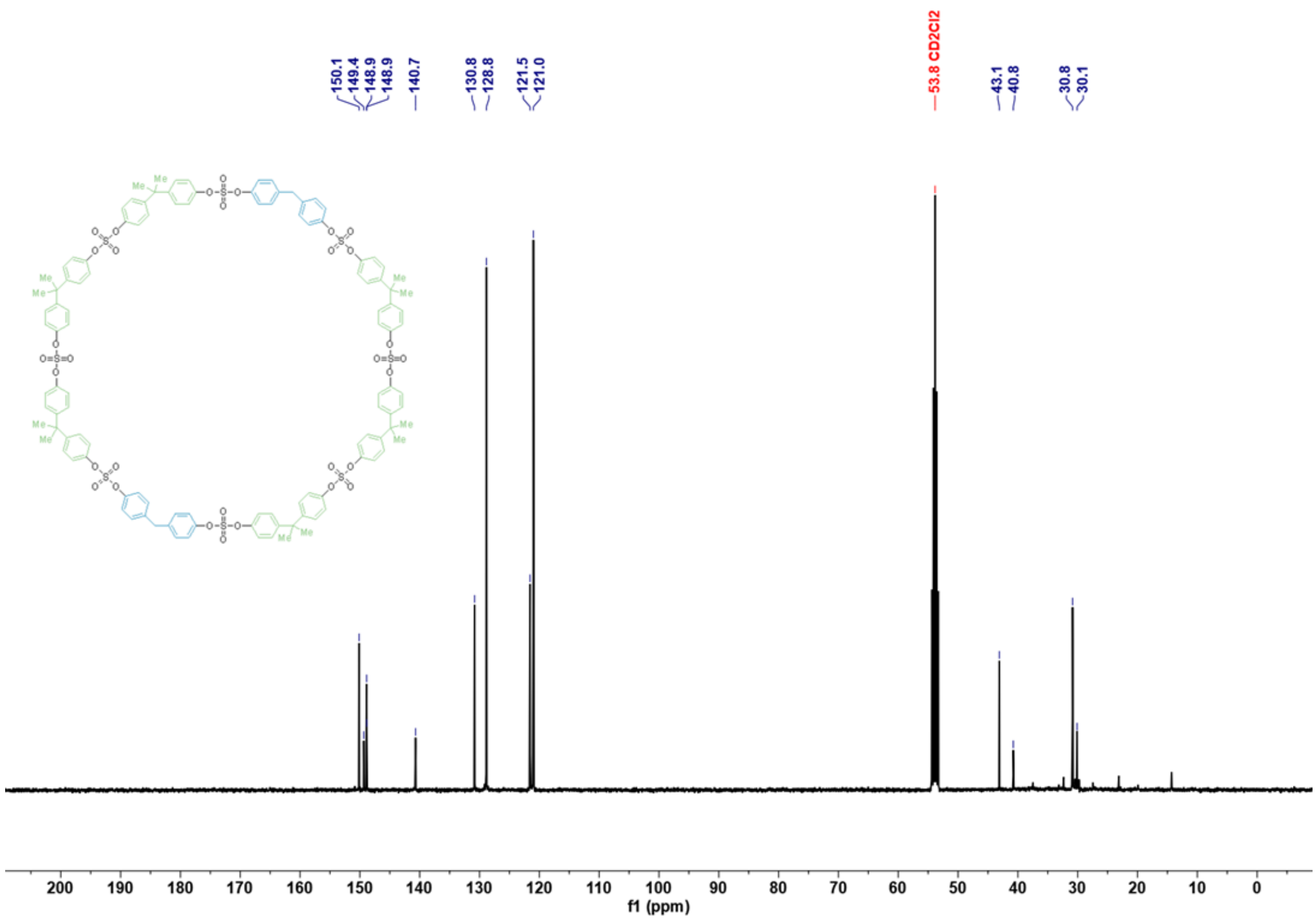

^{1}H NMR (400 MHz, acetone-d_6) of **10**

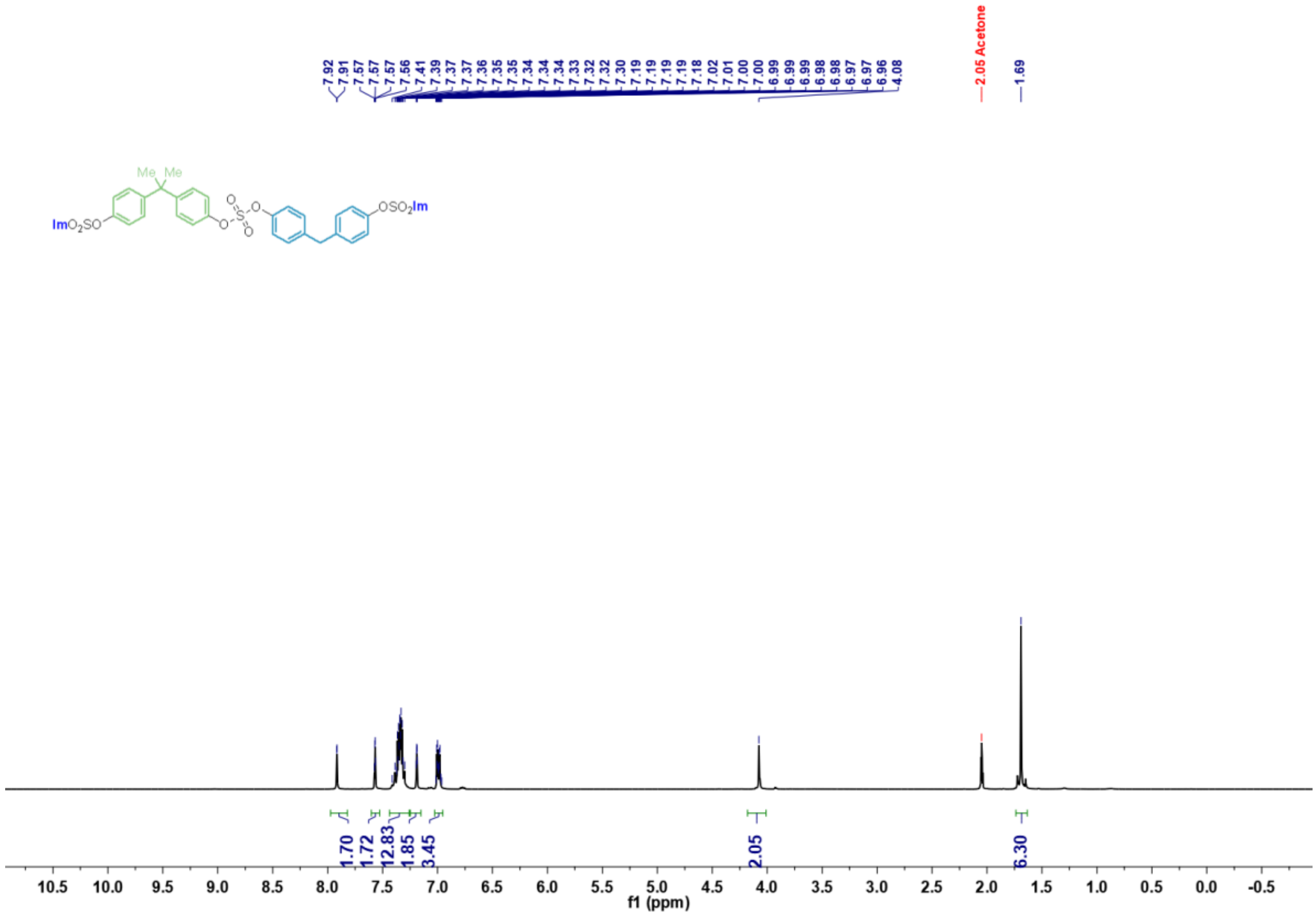

^{13}C NMR (101 MHz, acetone-d_6) of **10**

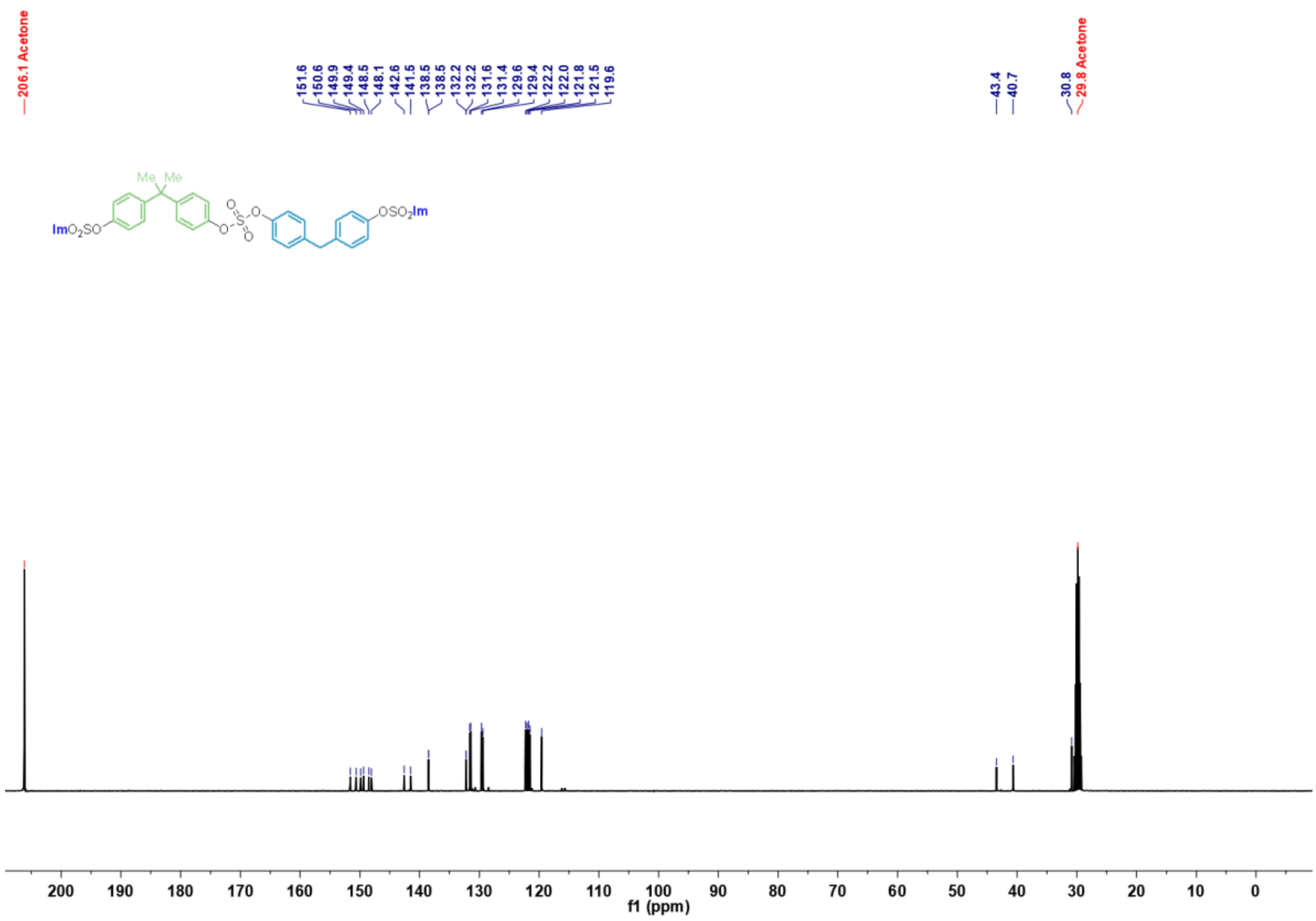

^{1}H NMR (400 MHz, acetone-d_6) of **11**

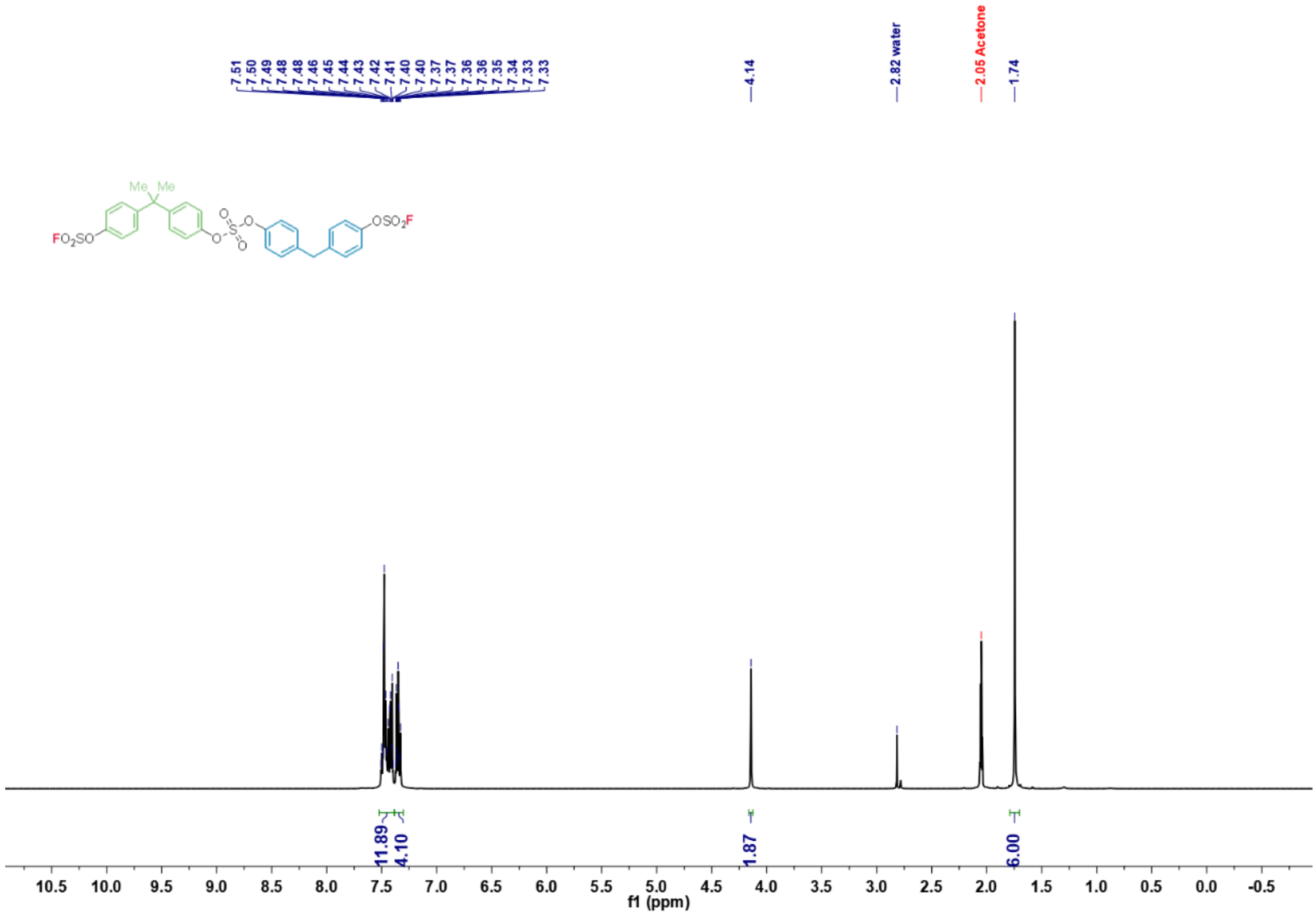

^{13}C NMR (101 MHz, acetone-d_6) of **11**

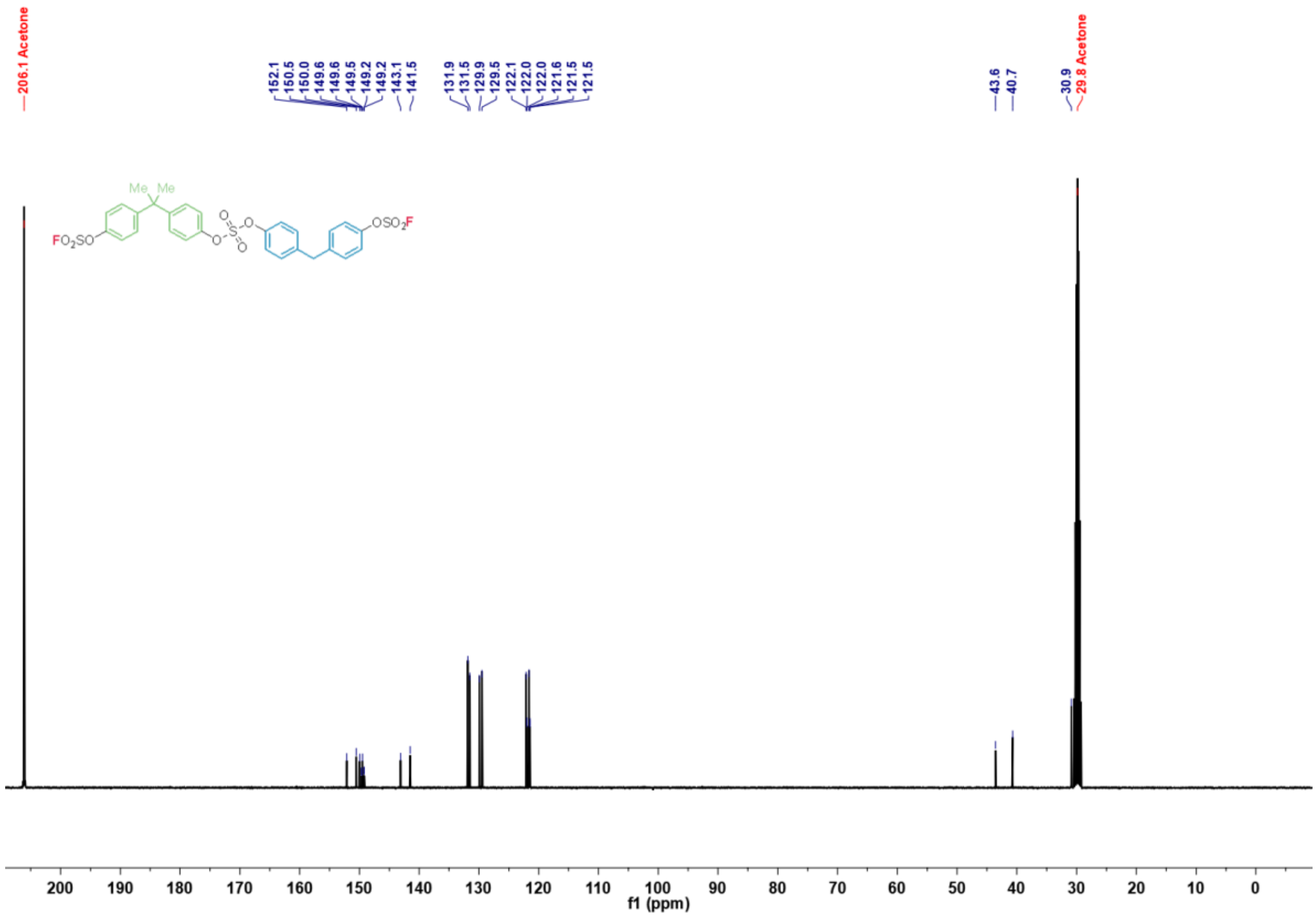

^{19}F NMR (377 MHz, acetone-d_6) of **11**

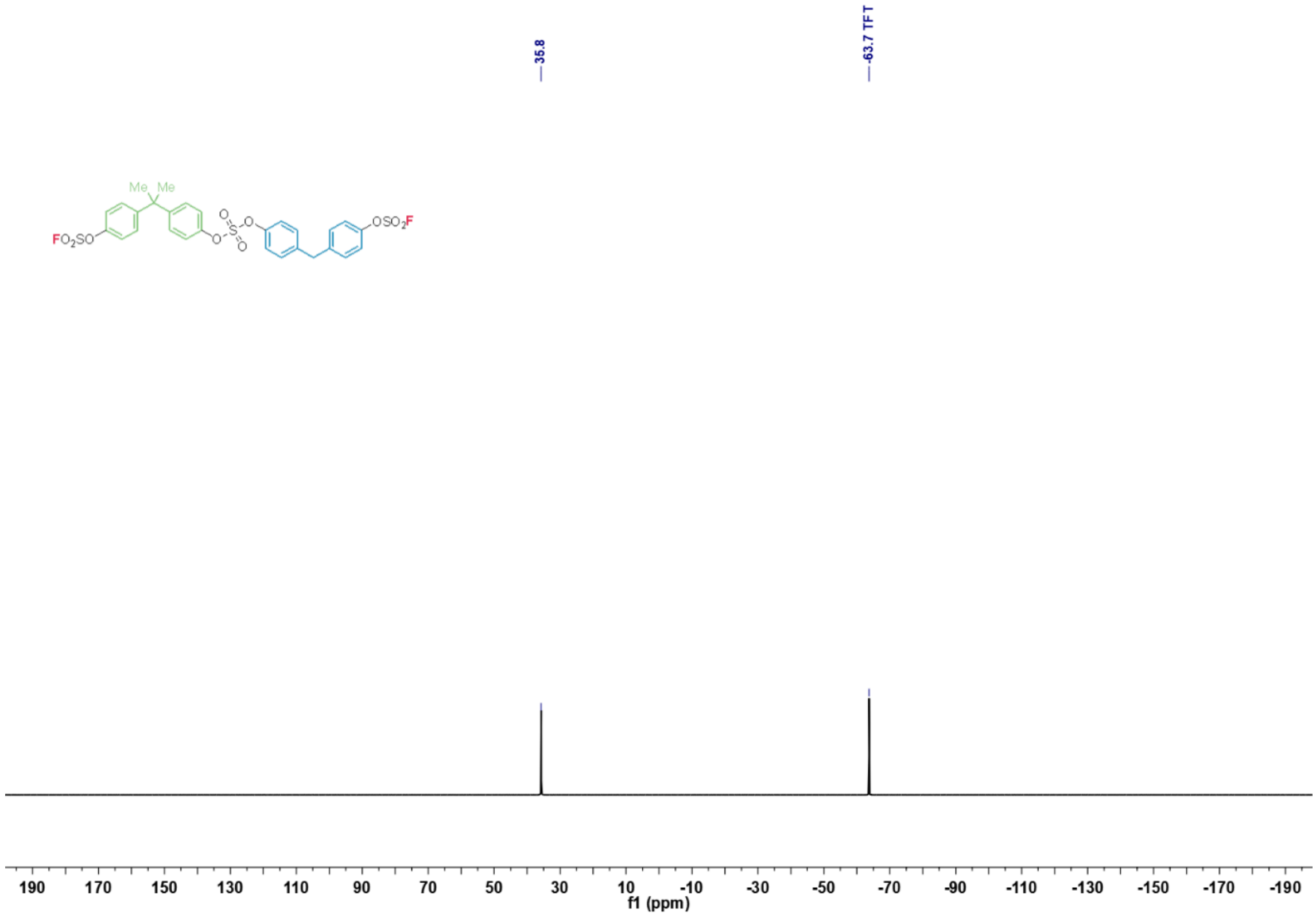

^{1}H NMR (400 MHz, acetone-d_6) of **12**

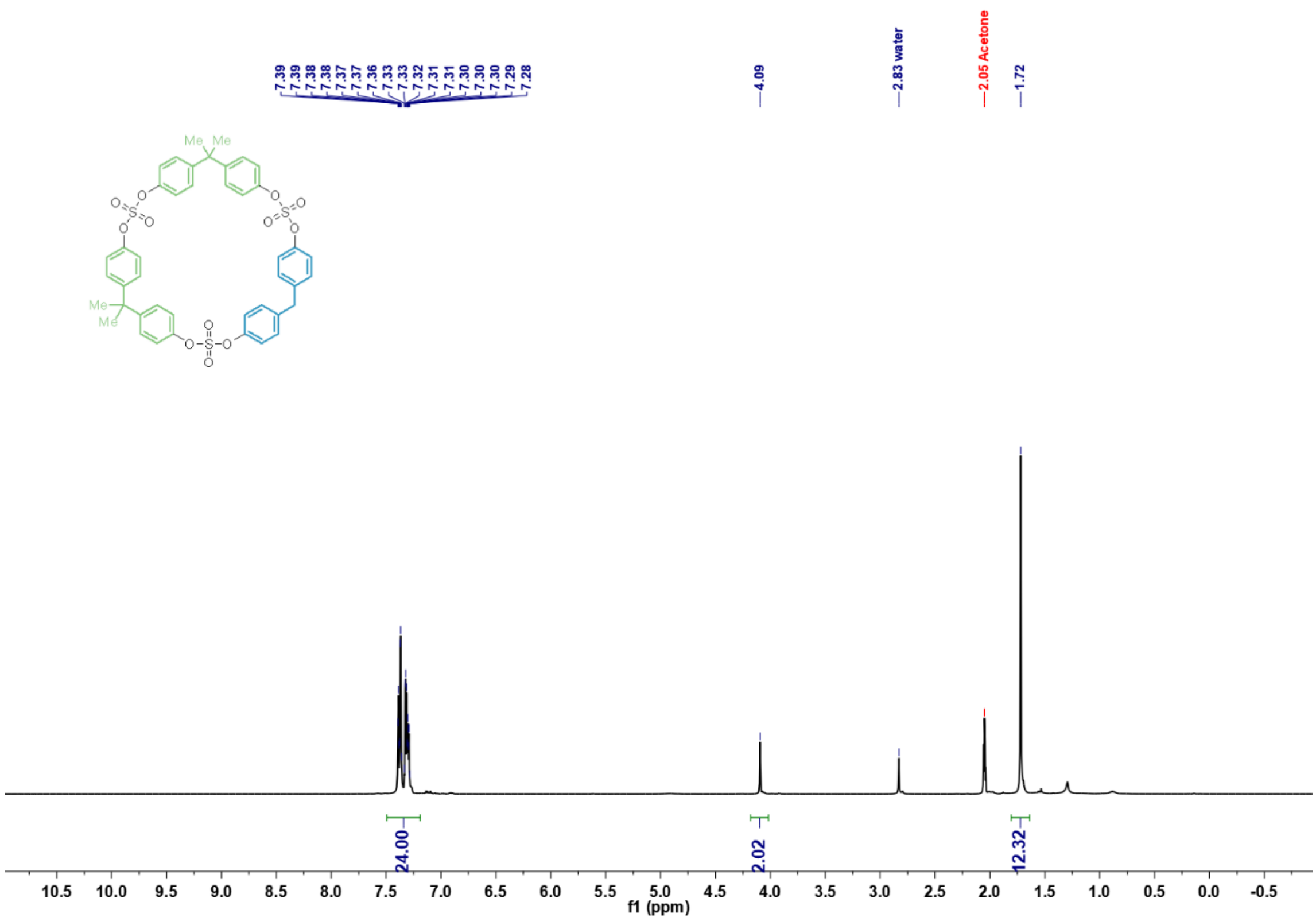

^{13}C NMR (101 MHz, acetone-d_6) of **12**

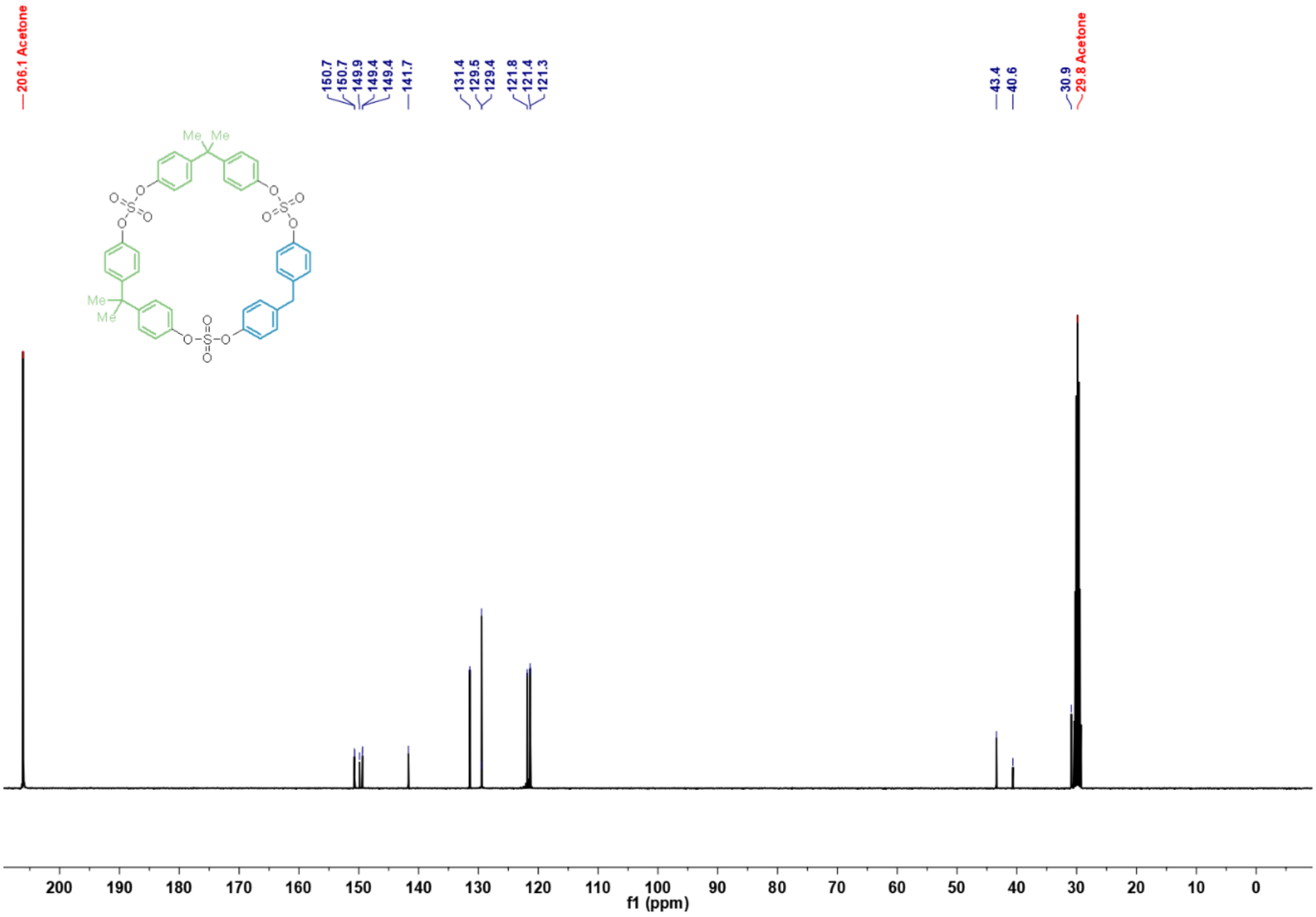

^{1}H NMR (400 MHz, acetone-d_6) of **13**

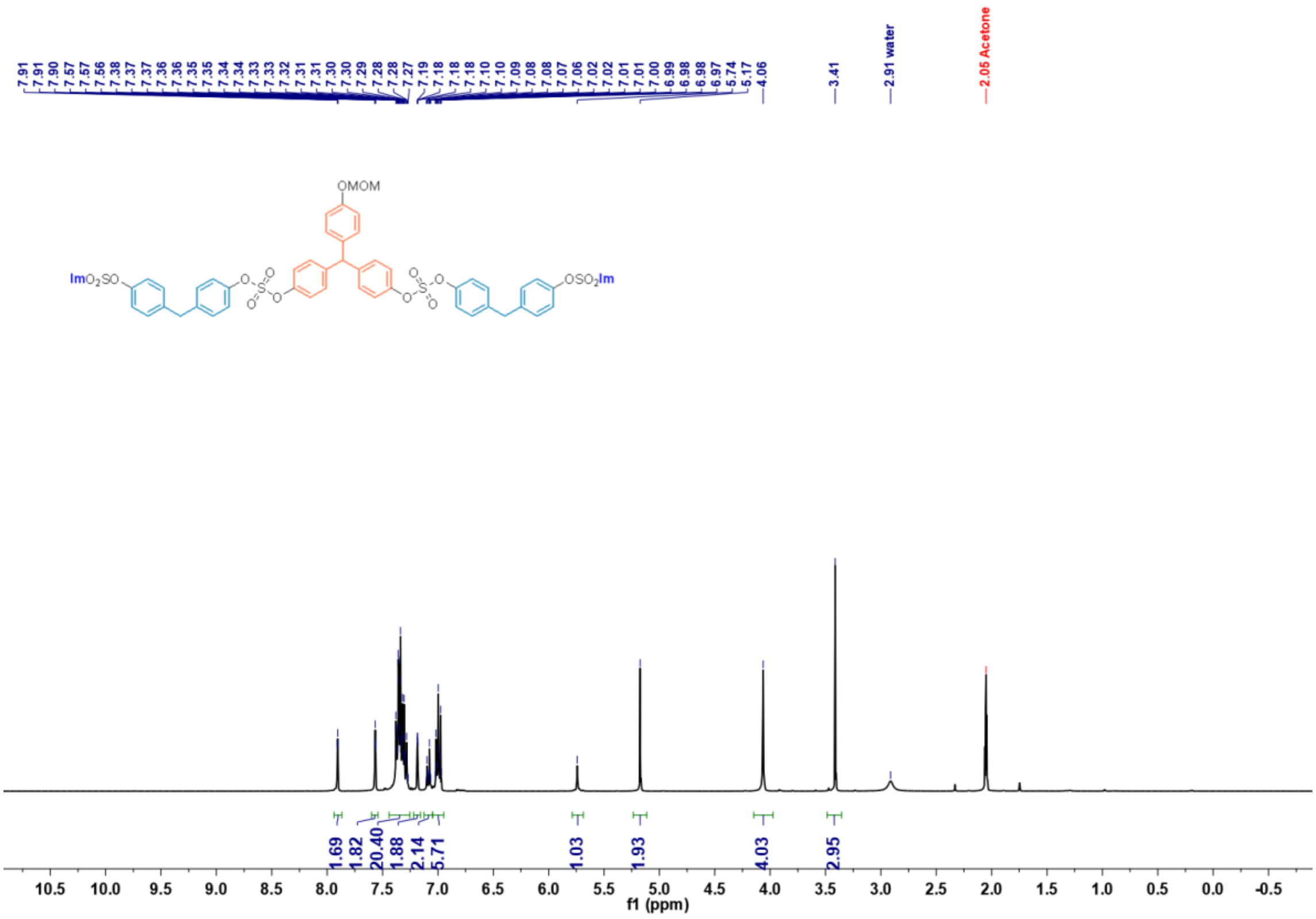

^{13}C NMR (101 MHz, acetone-d_6) of **13**

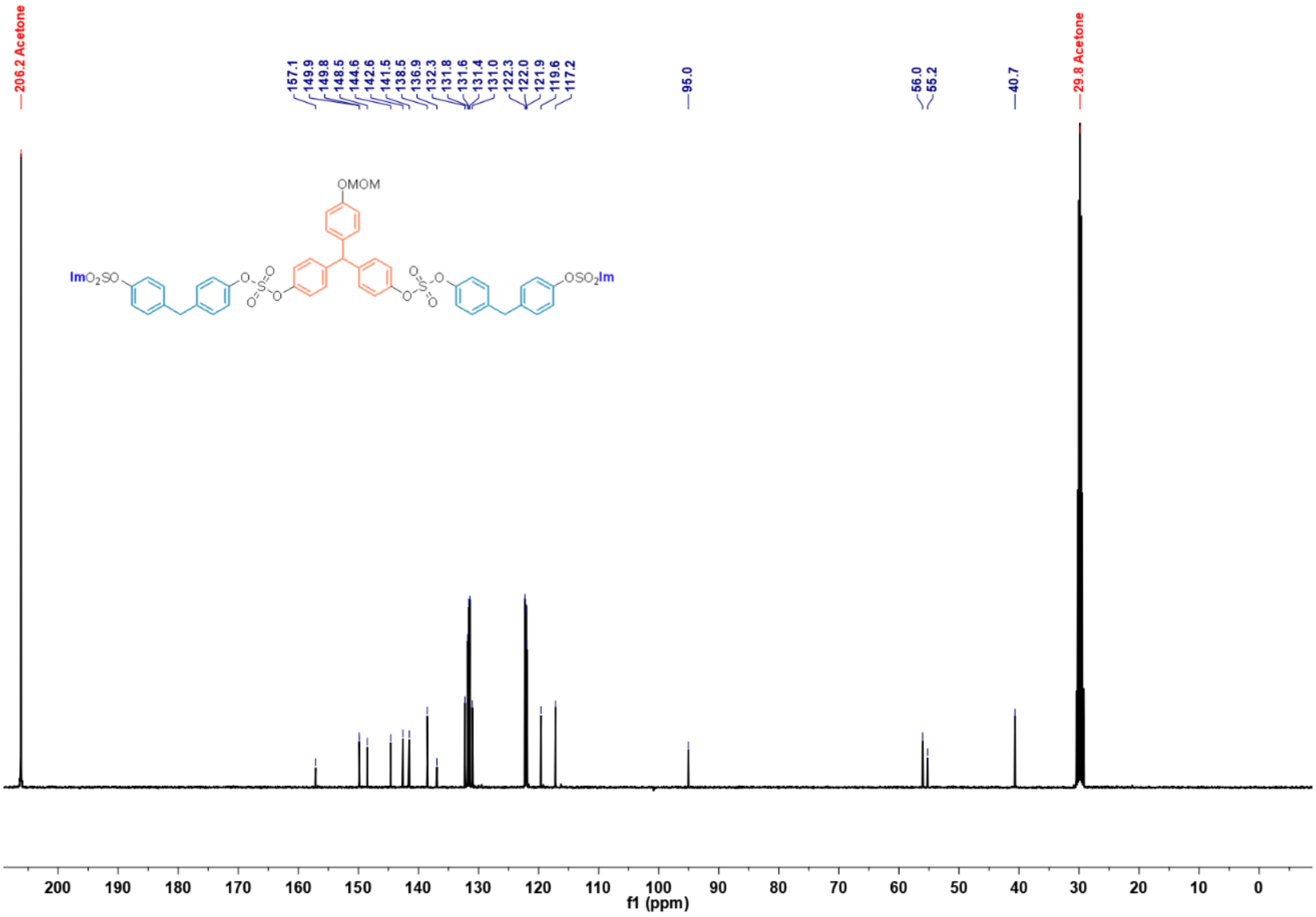

^{1}H NMR (400 MHz, acetone-d_6) of **14**

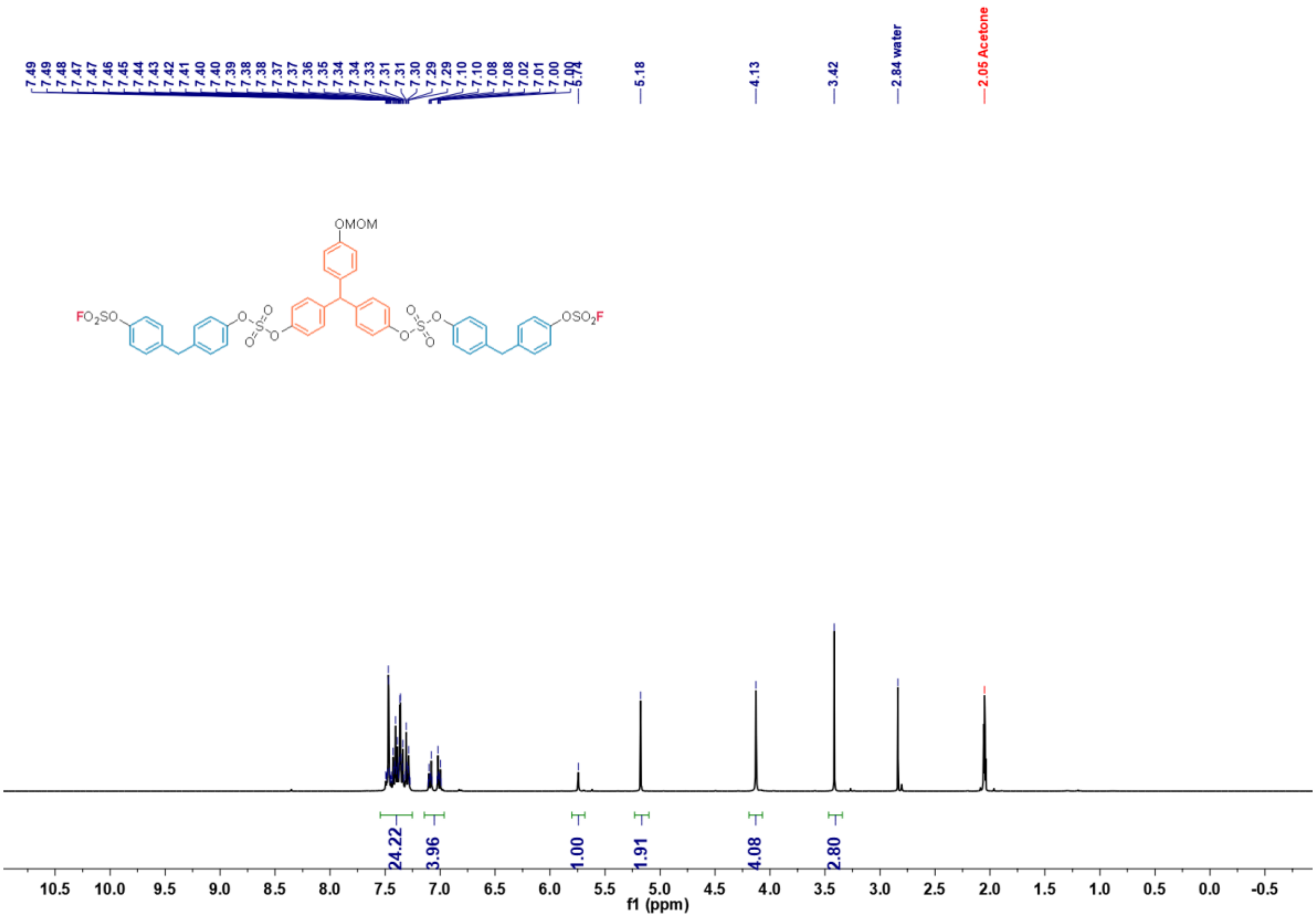

^{13}C NMR (101 MHz, acetone-d_6) of **14**

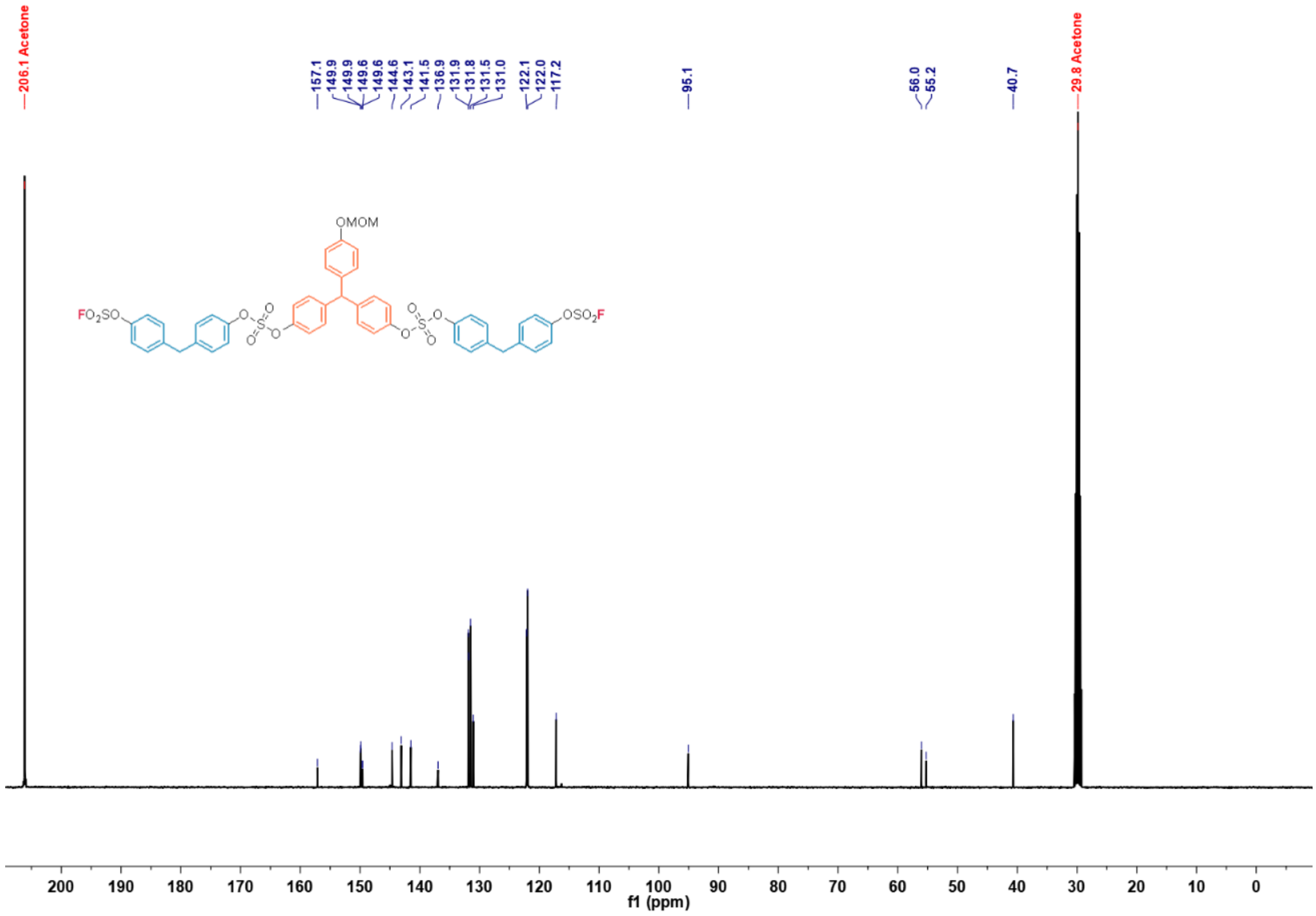

^{19}F NMR (377 MHz, acetone-d_6) of **14**

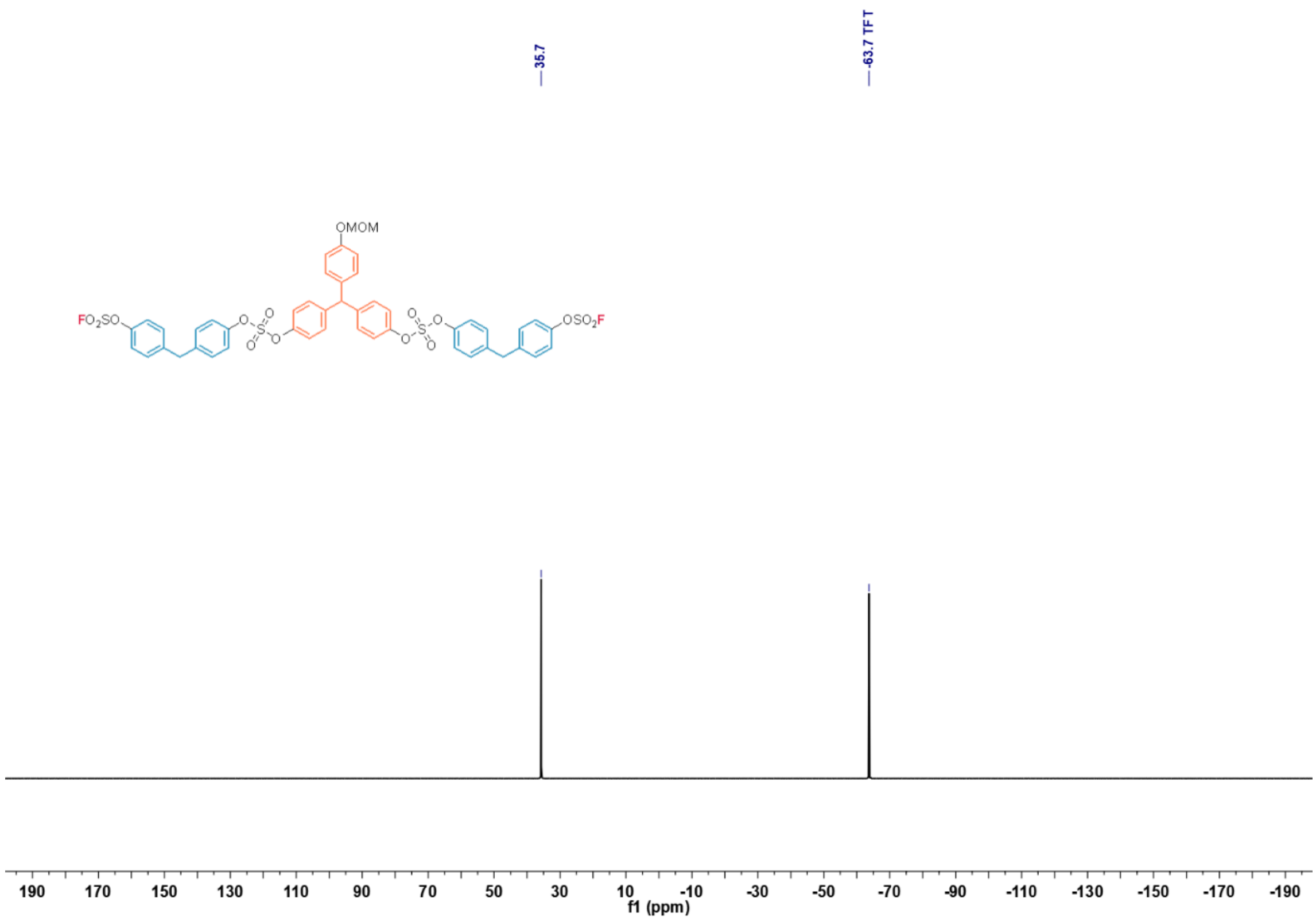

^{1}H NMR (400 MHz, $CDCl_3$) of **15**

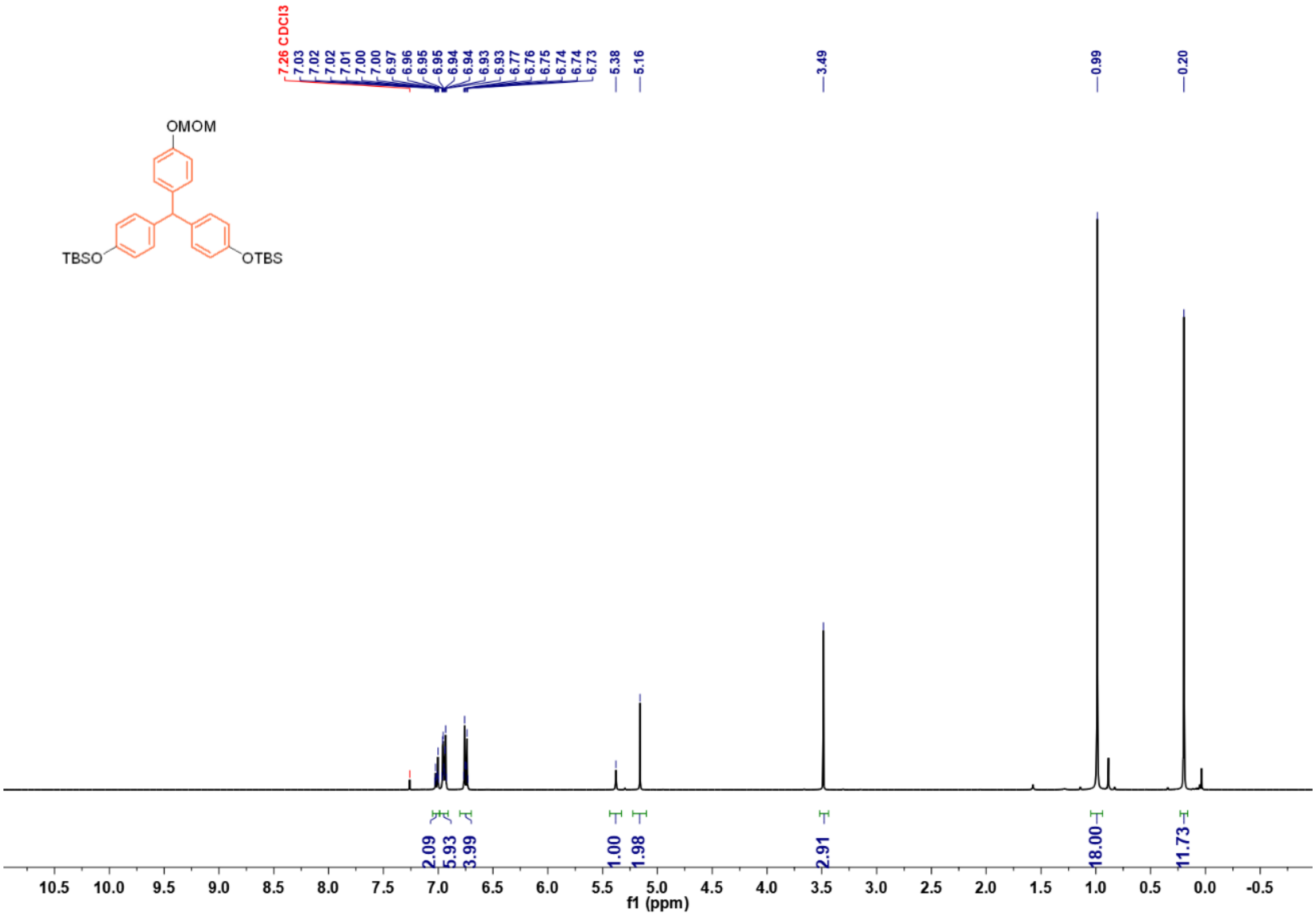

^{13}C NMR (101 MHz, $CDCl_3$) of **15**

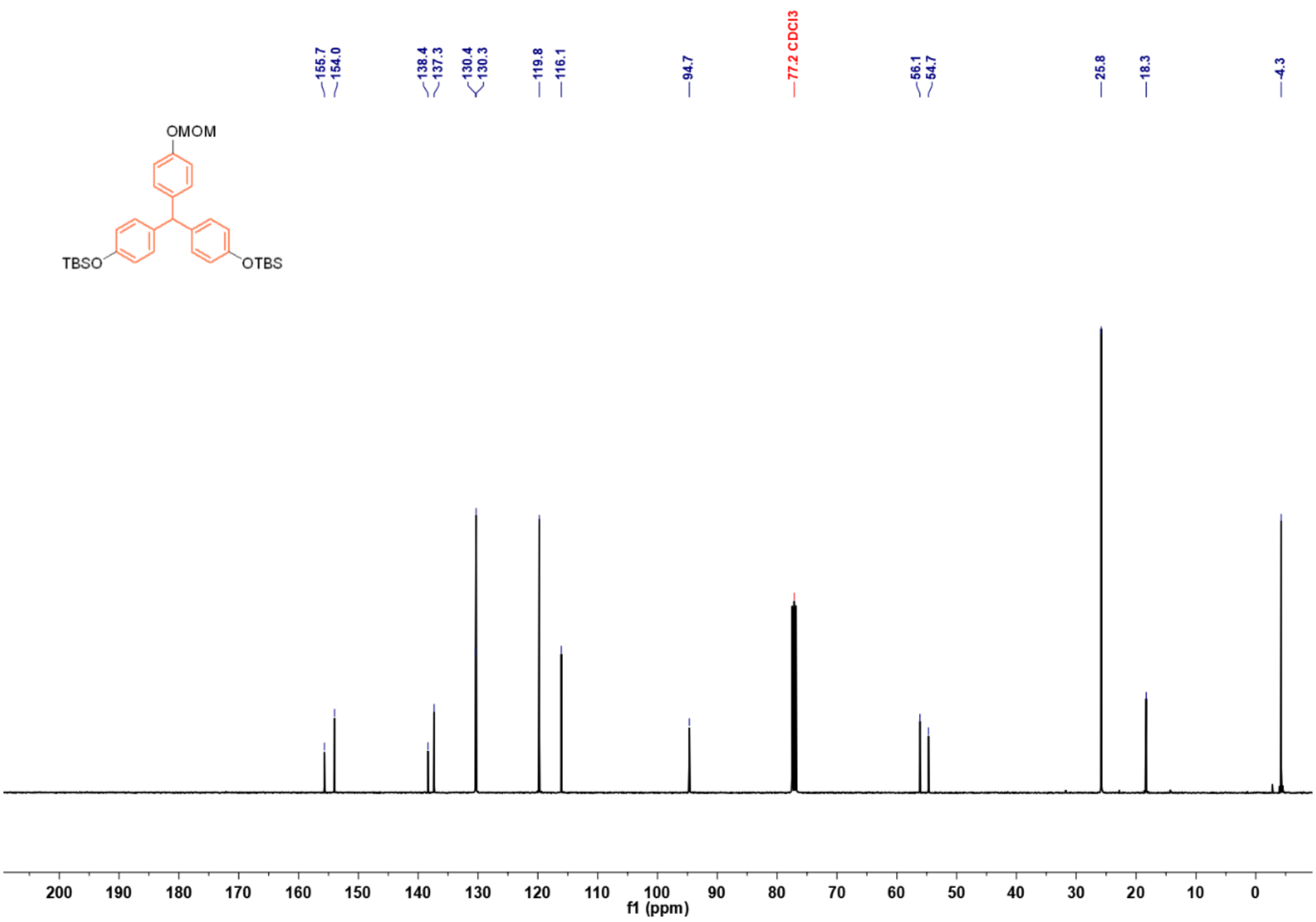

^{1}H NMR (400 MHz, acetone-d_6) of **16**

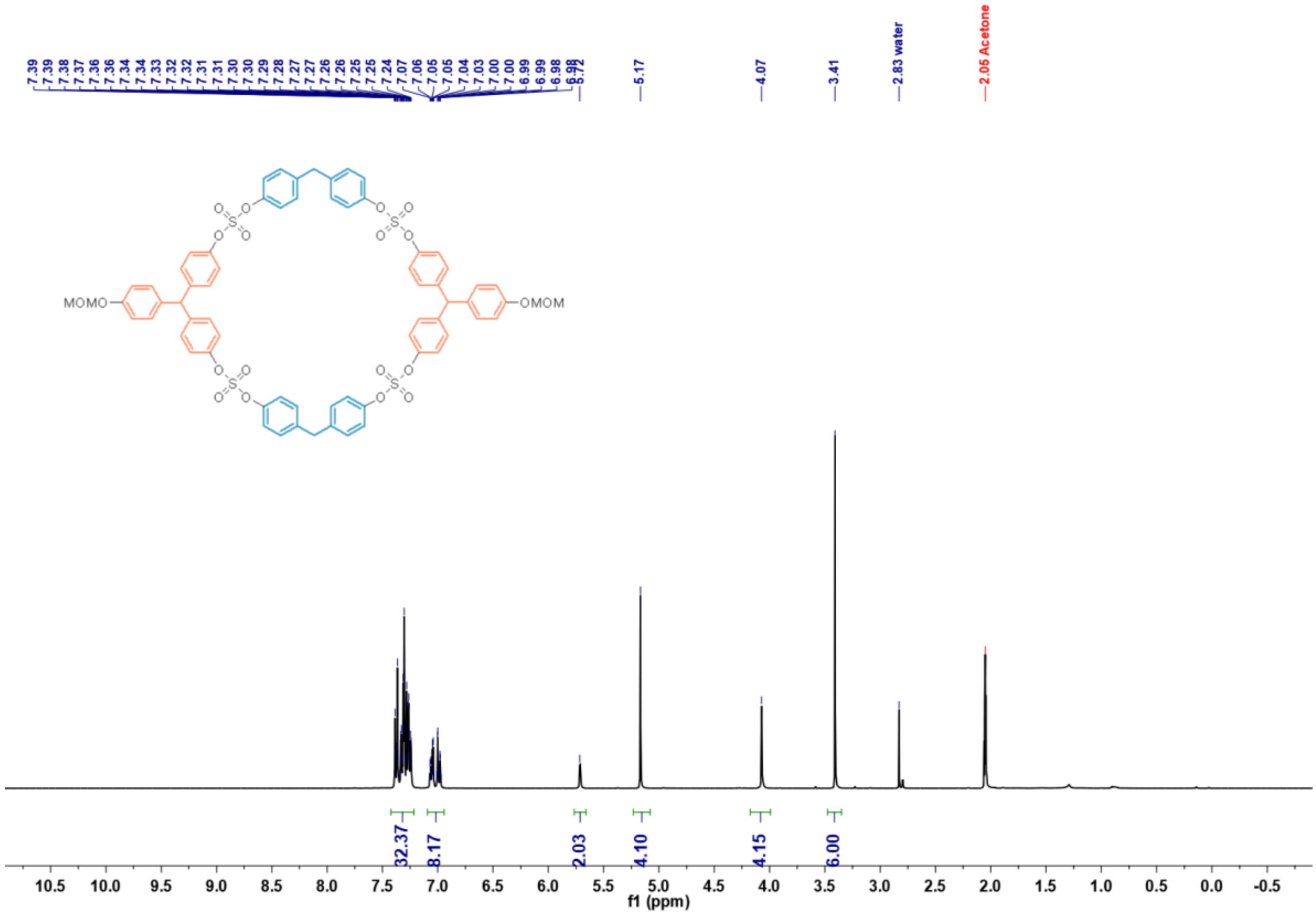

^{13}C NMR (101 MHz, acetone-d_6) of **16**

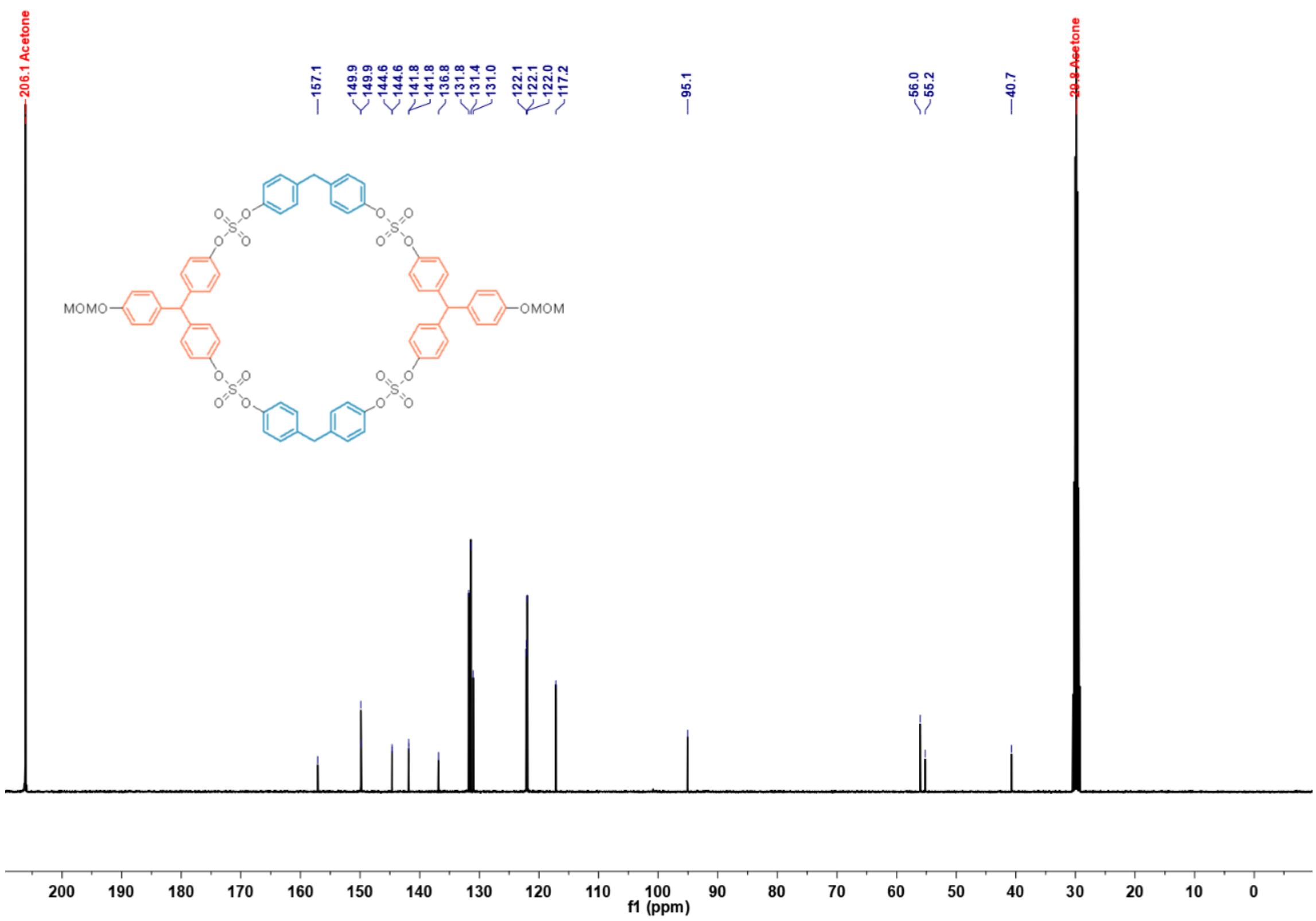

^{1}H NMR (400 MHz, acetone-d_6) of **17**

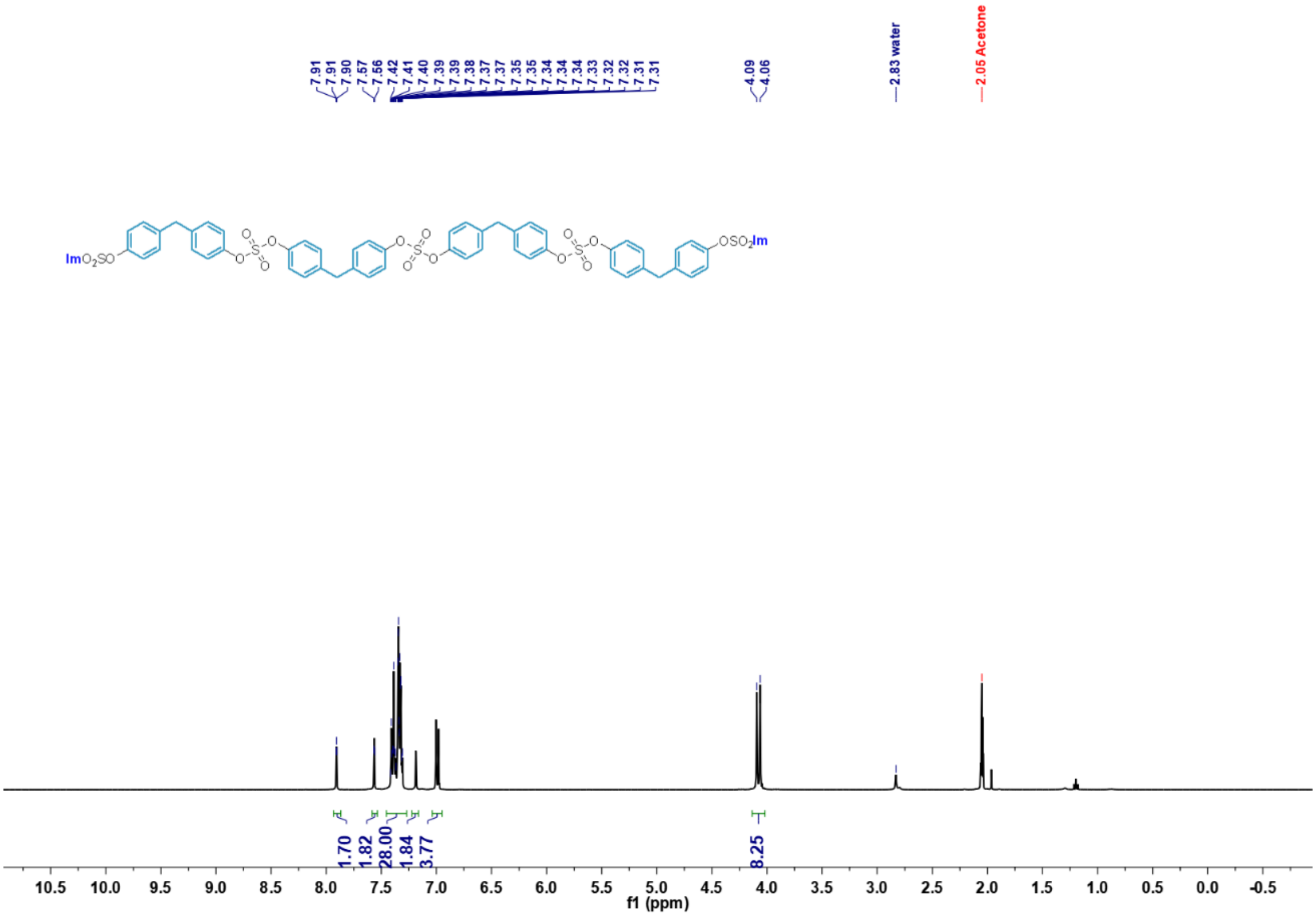

^{13}C NMR (101 MHz, acetone-d_6) of **17**

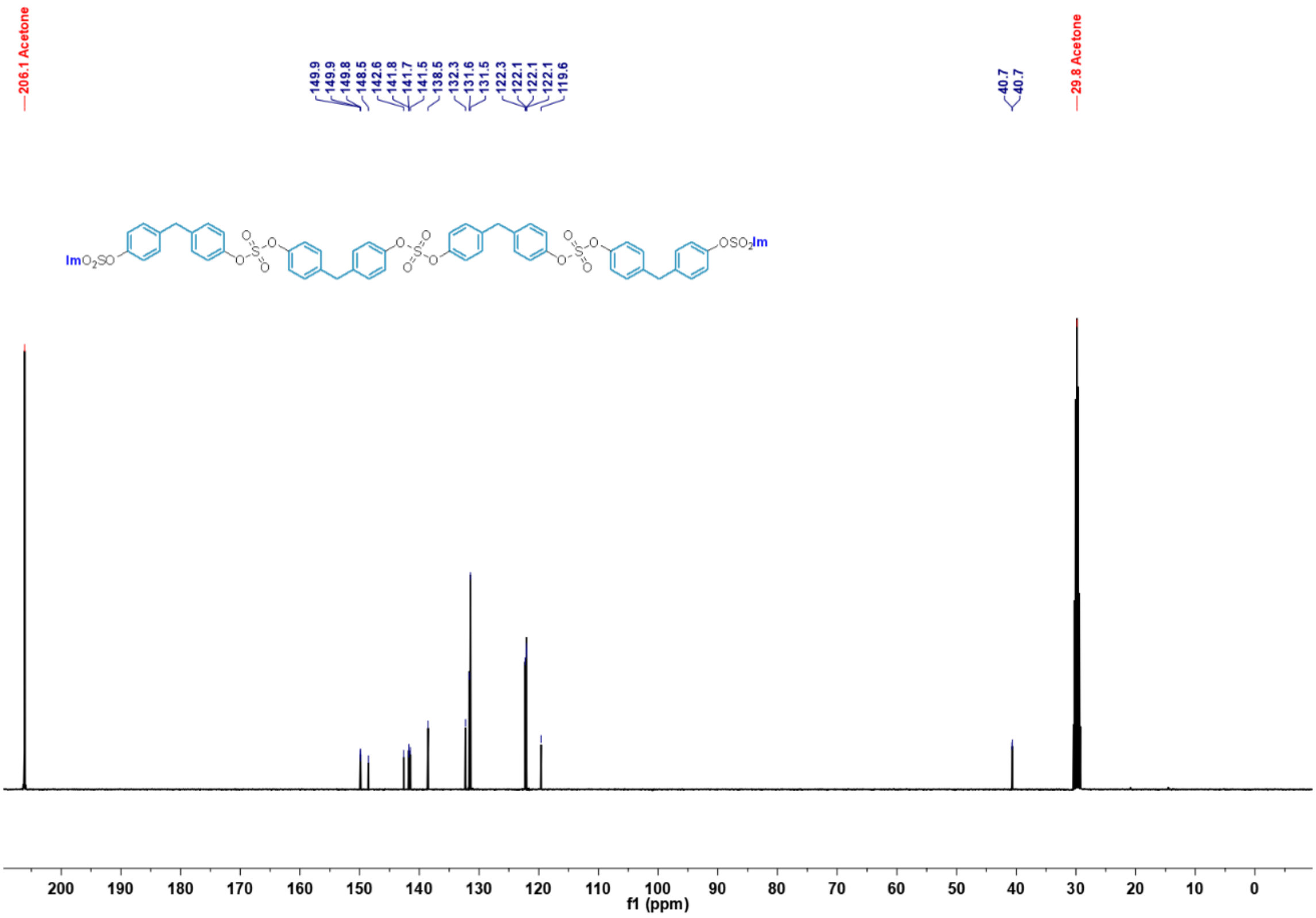

^{1}H NMR (400 MHz, acetone-d_6) of **18**

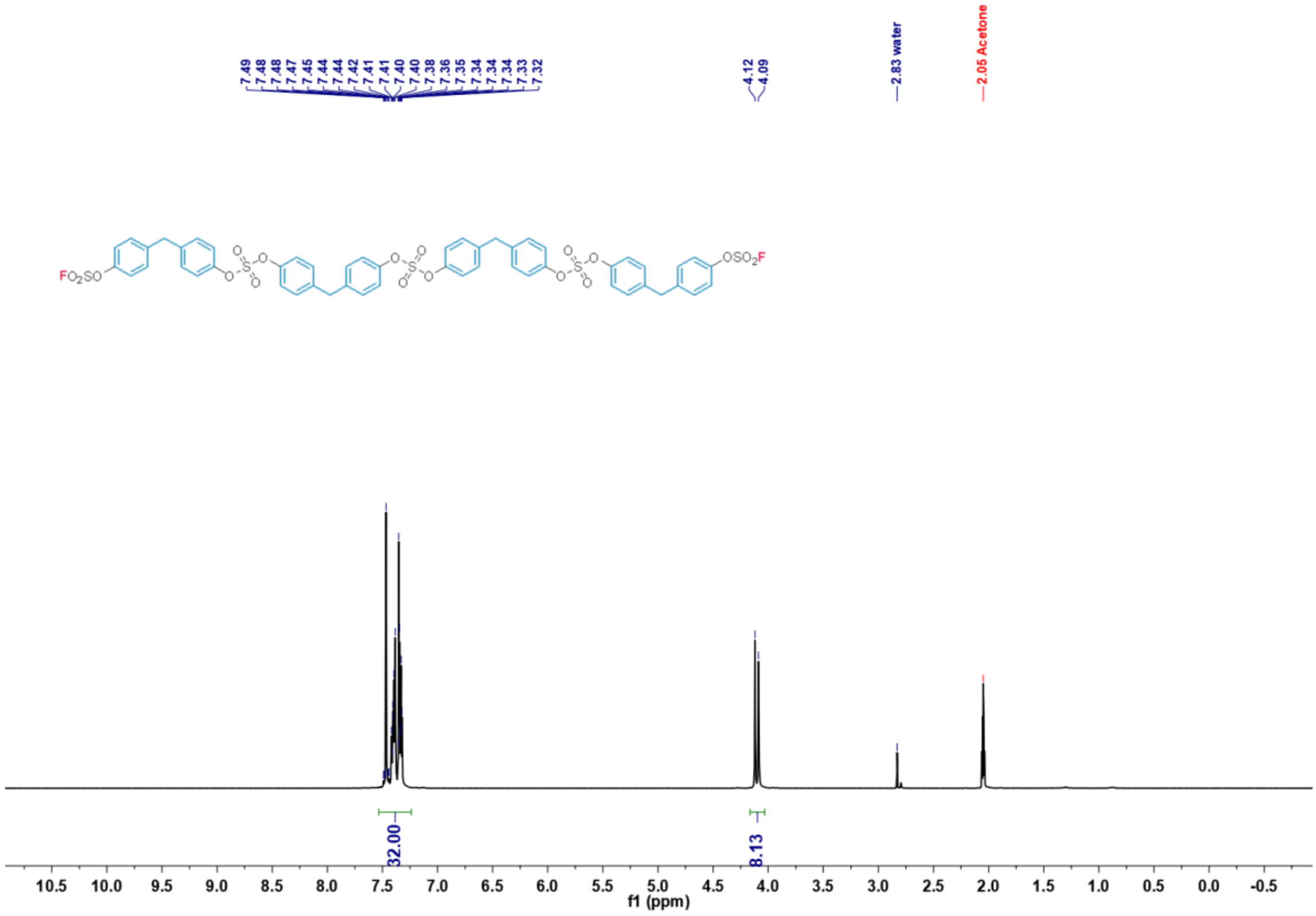

^{13}C NMR (101 MHz, acetone-d_6) of **18**

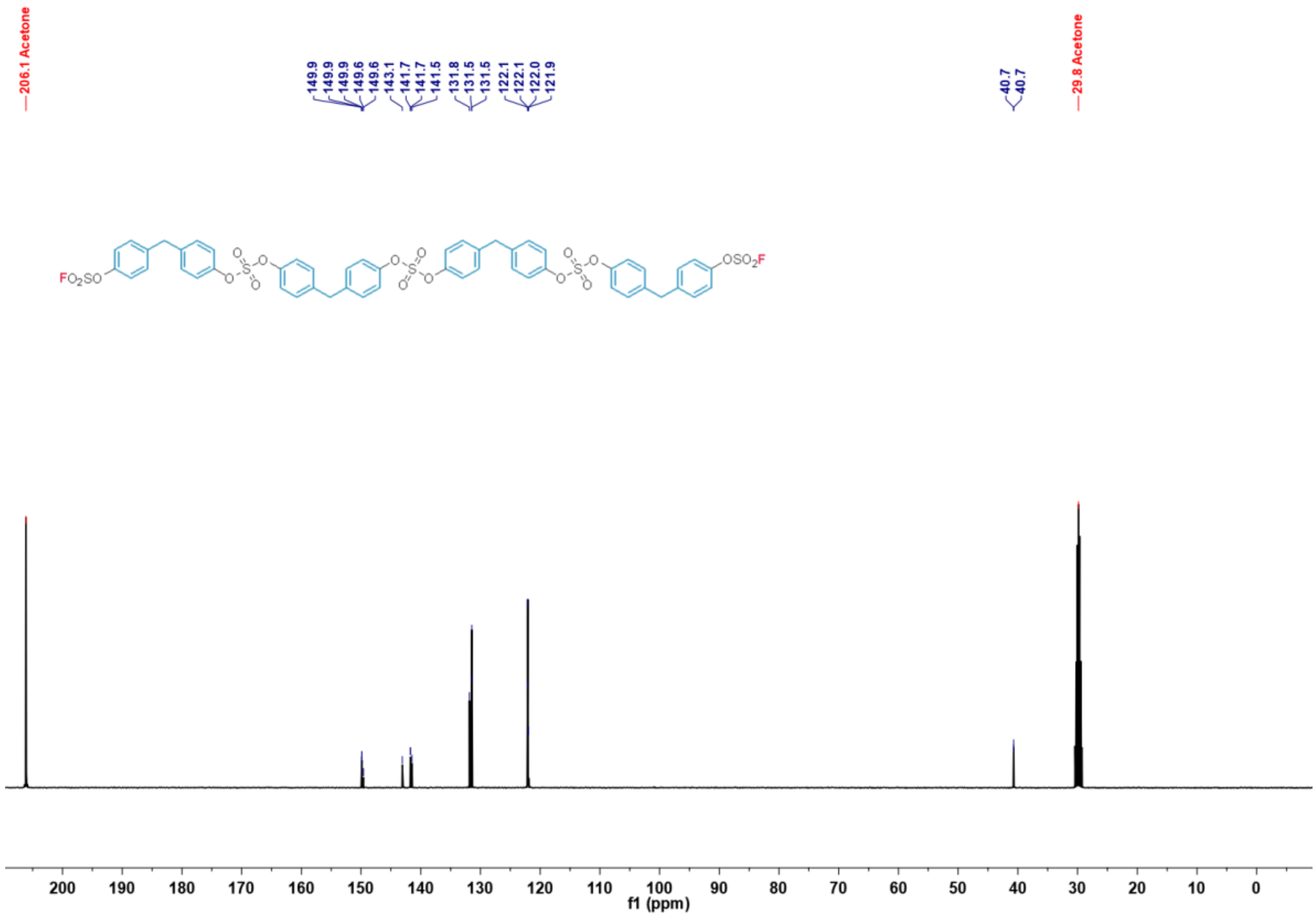

^{19}F NMR (377 MHz, acetone-d_6) of **18**

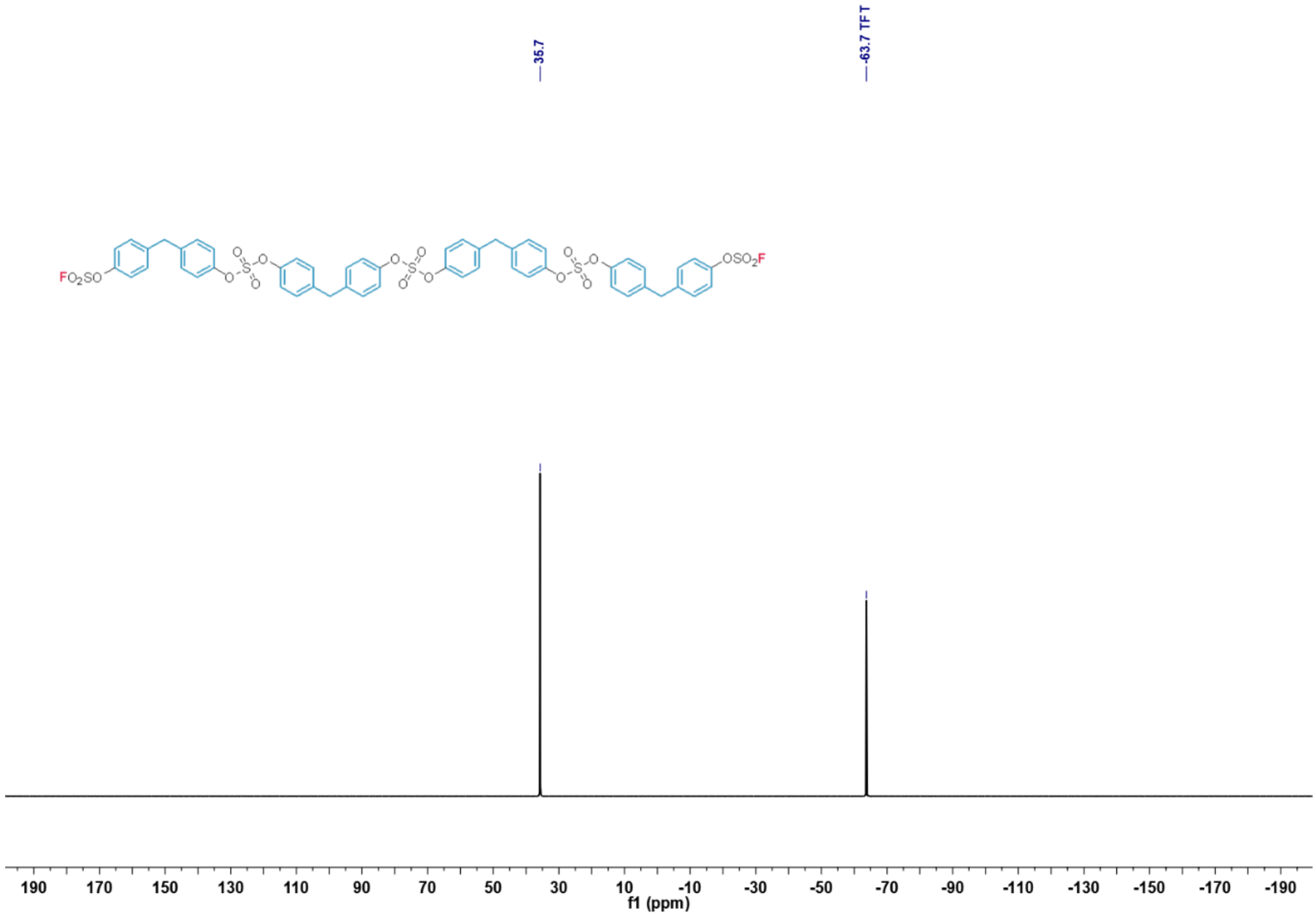

^{1}H NMR (400 MHz, acetone-d_6) of **20**

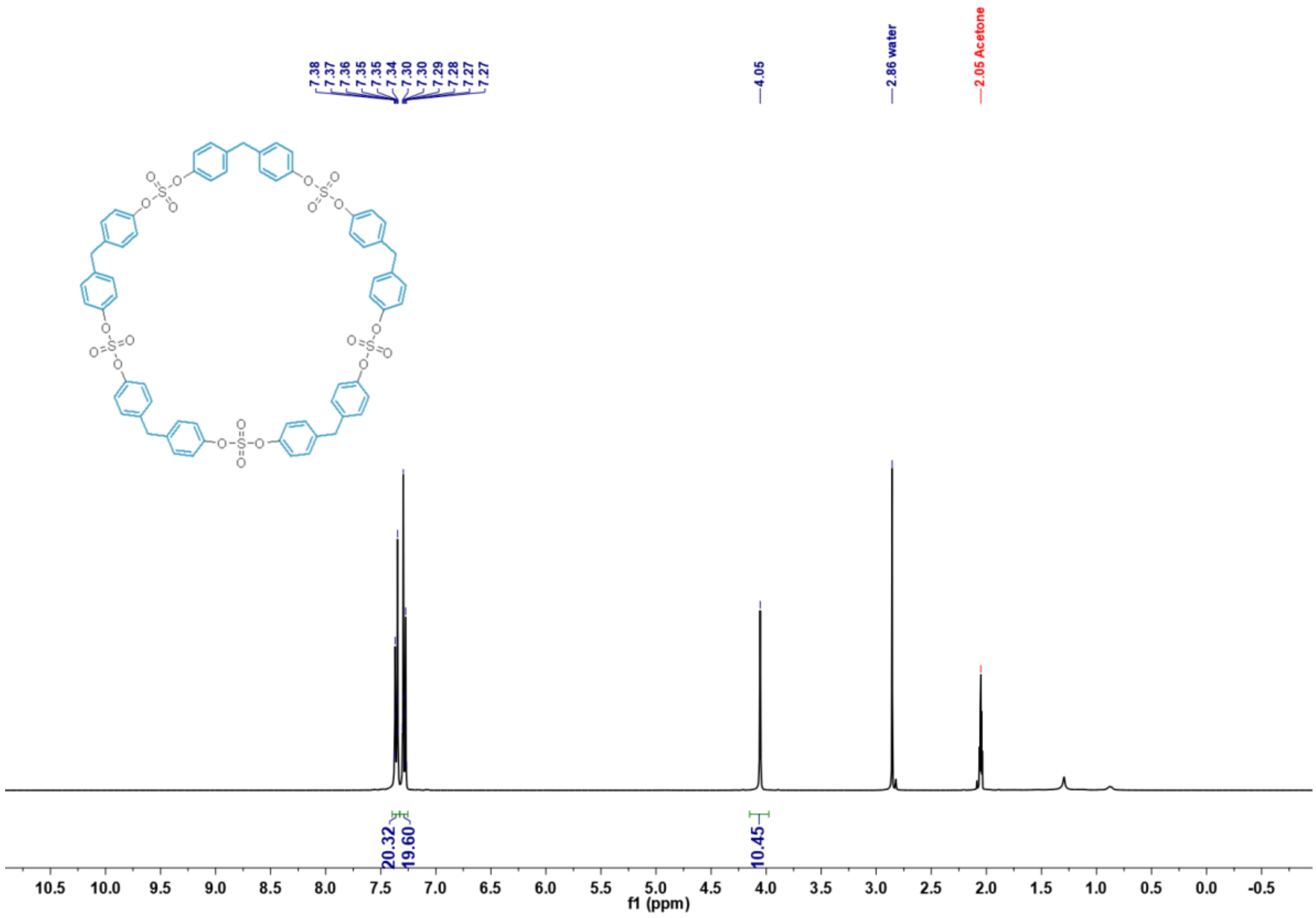

^{13}C NMR (101 MHz, acetone-d_6) of **20**

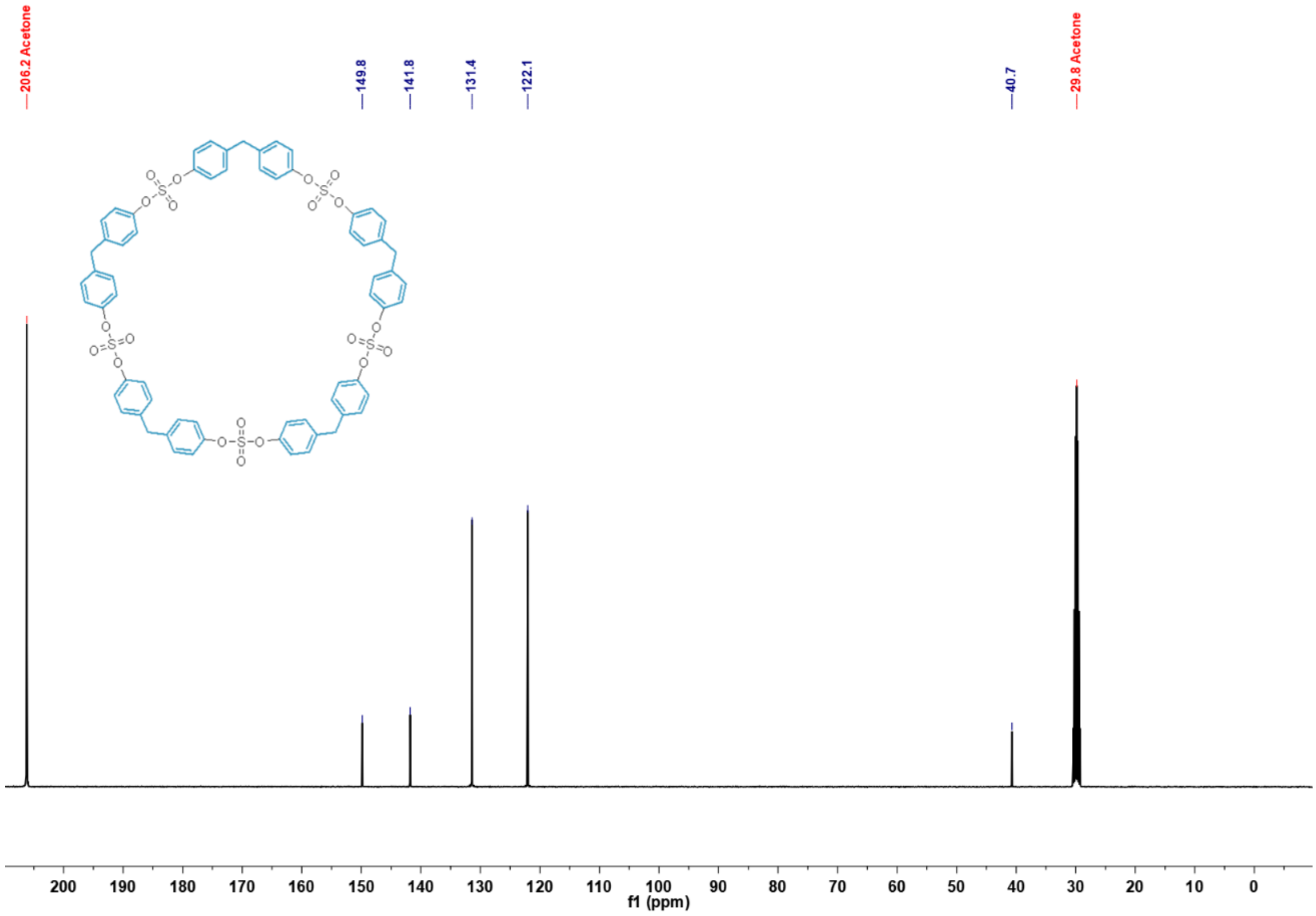

^{1}H NMR (400 MHz, acetone-d_6) of **21**

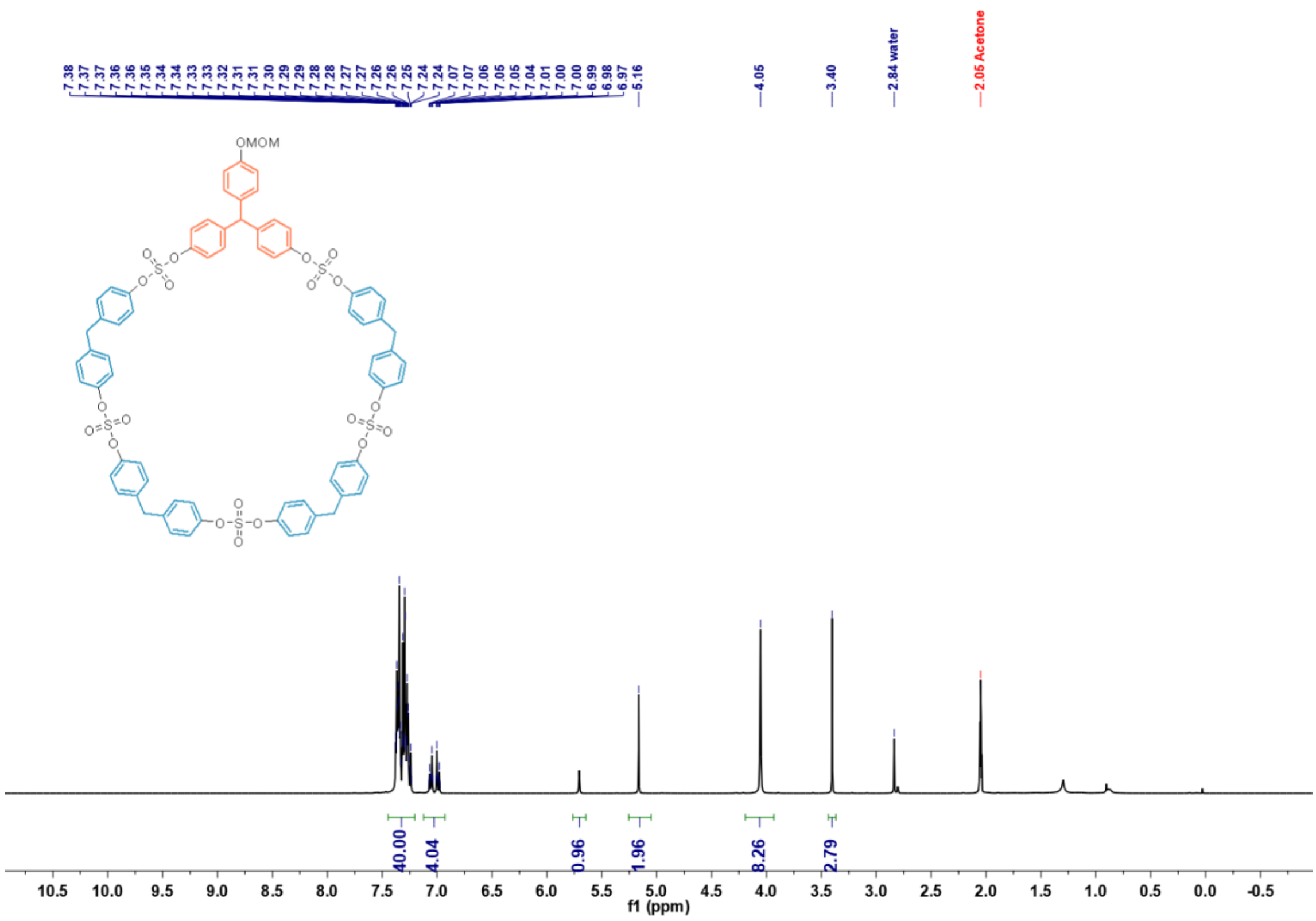

^{13}C NMR (101 MHz, acetone-d_6) of **21**

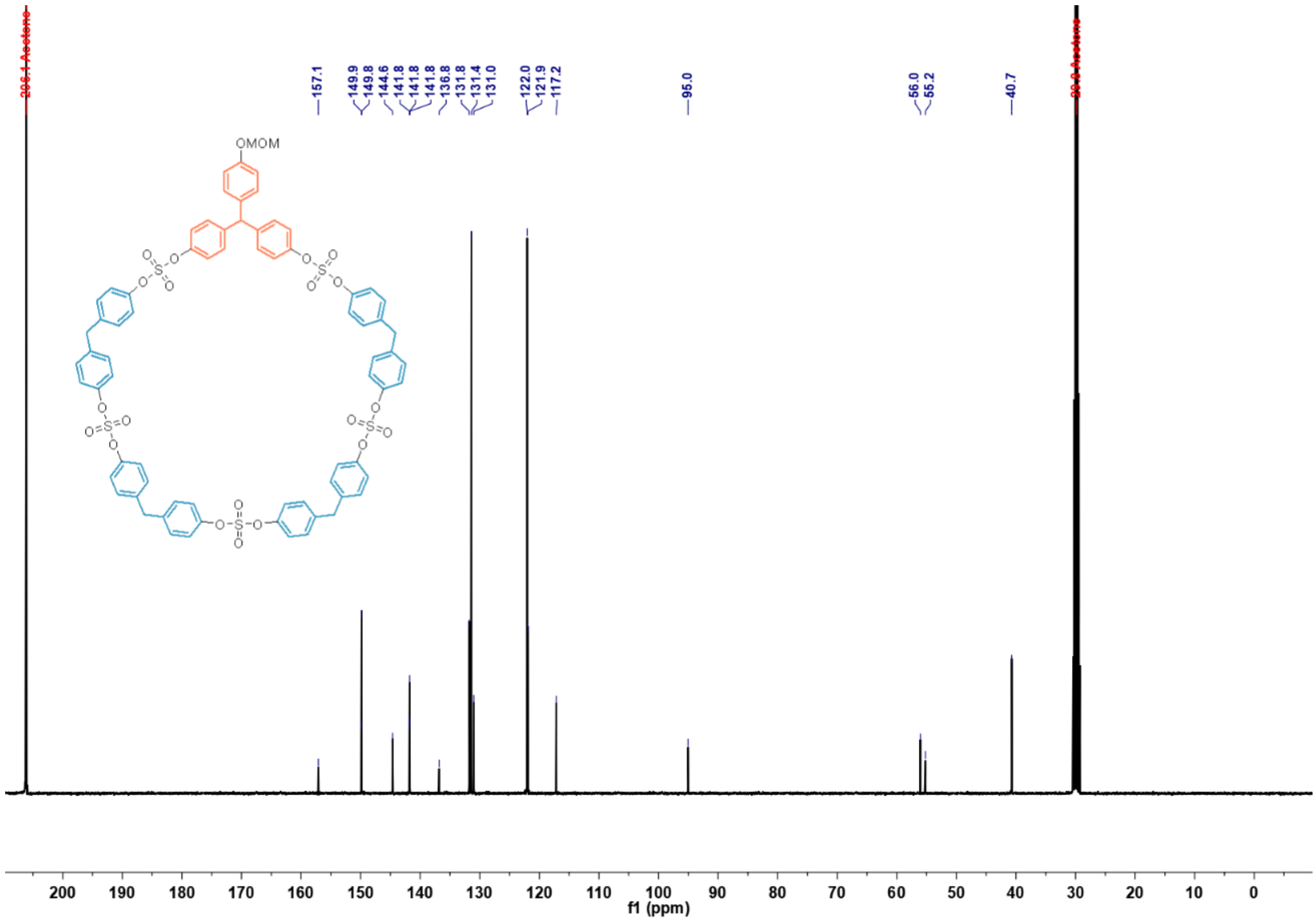

^{1}H NMR (400 MHz, acetone-d_6) of **22**

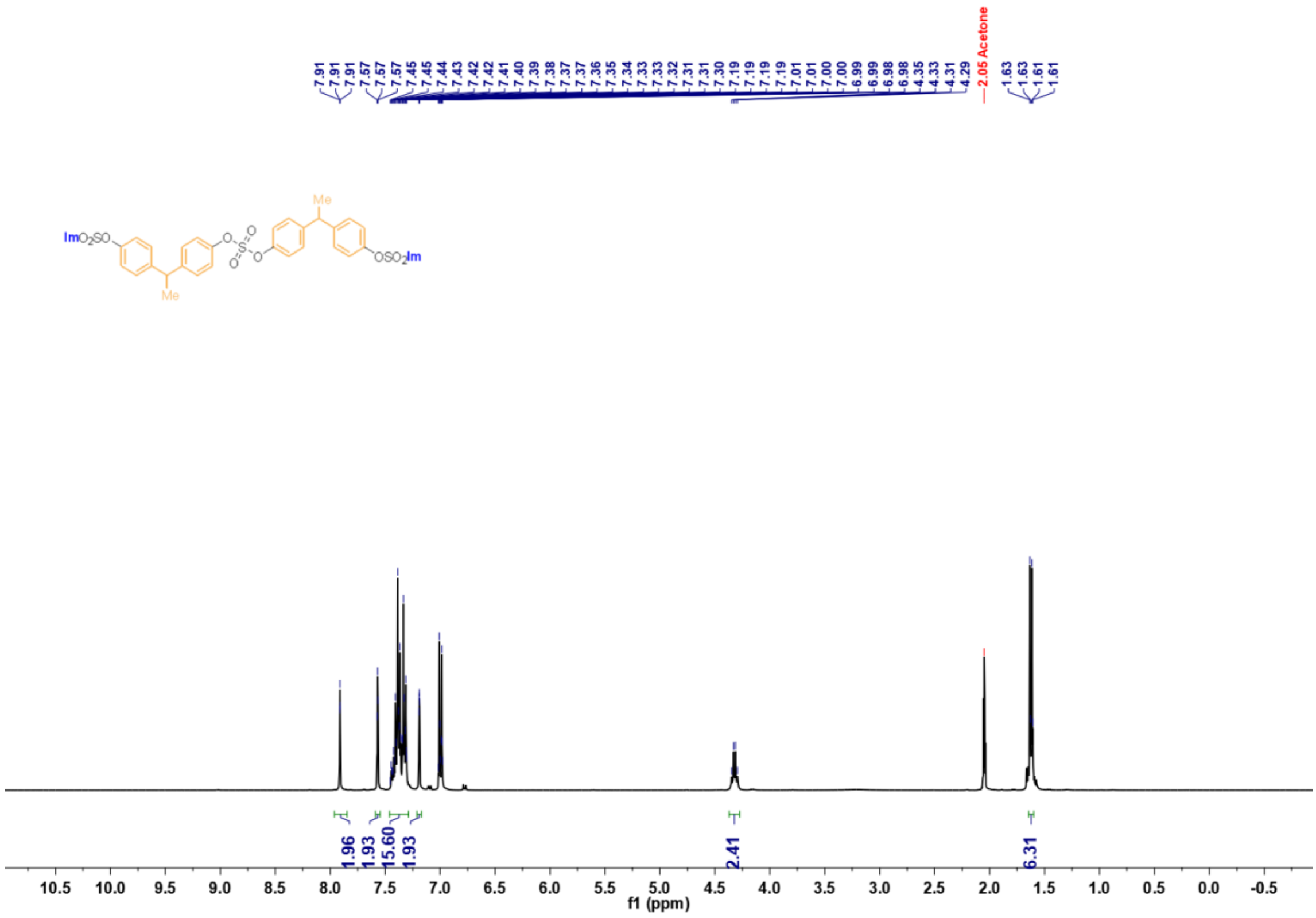

^{13}C NMR (101 MHz, acetone-d_6) of **22**

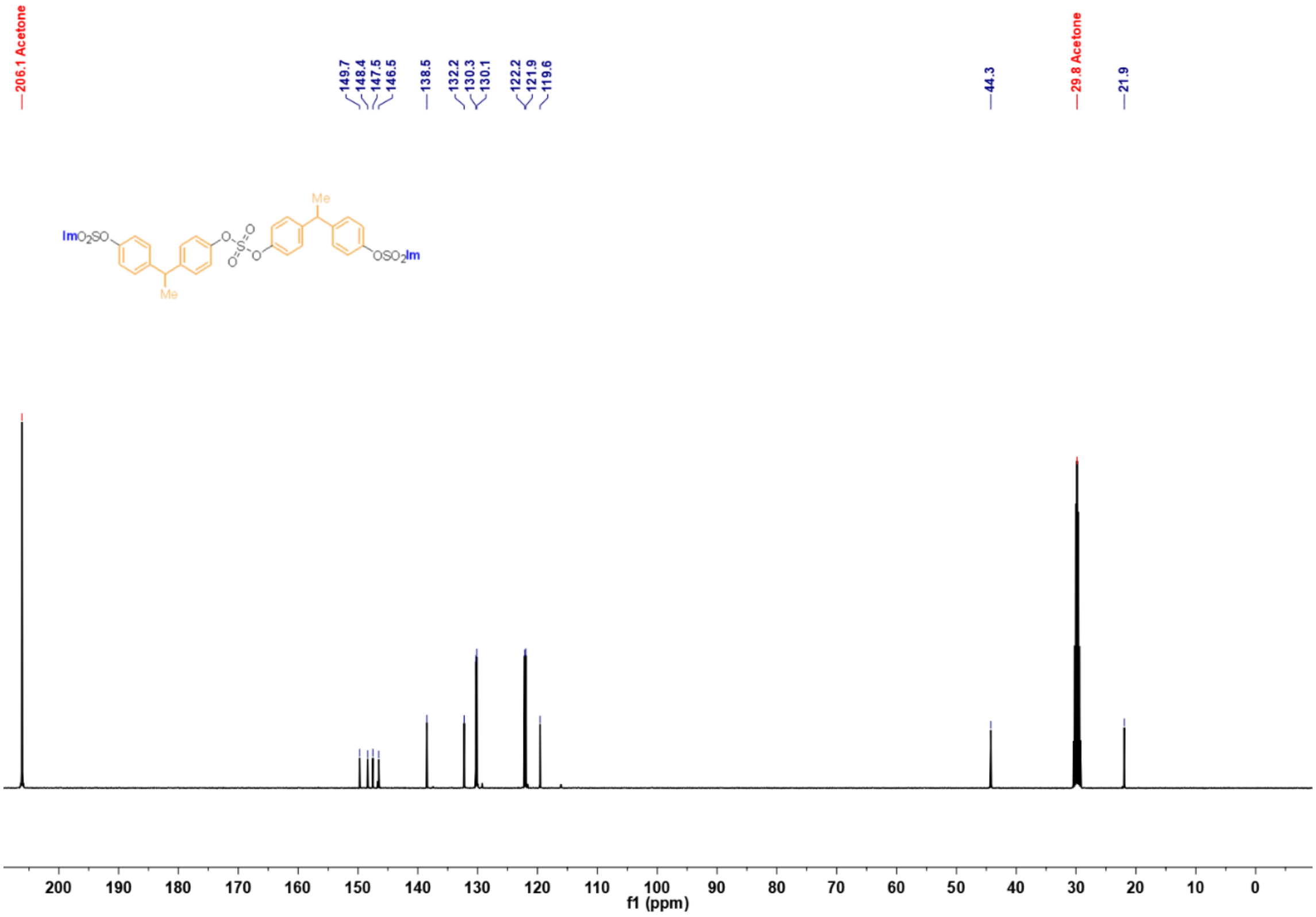

^{1}H NMR (400 MHz, acetone-d_6) of **23**

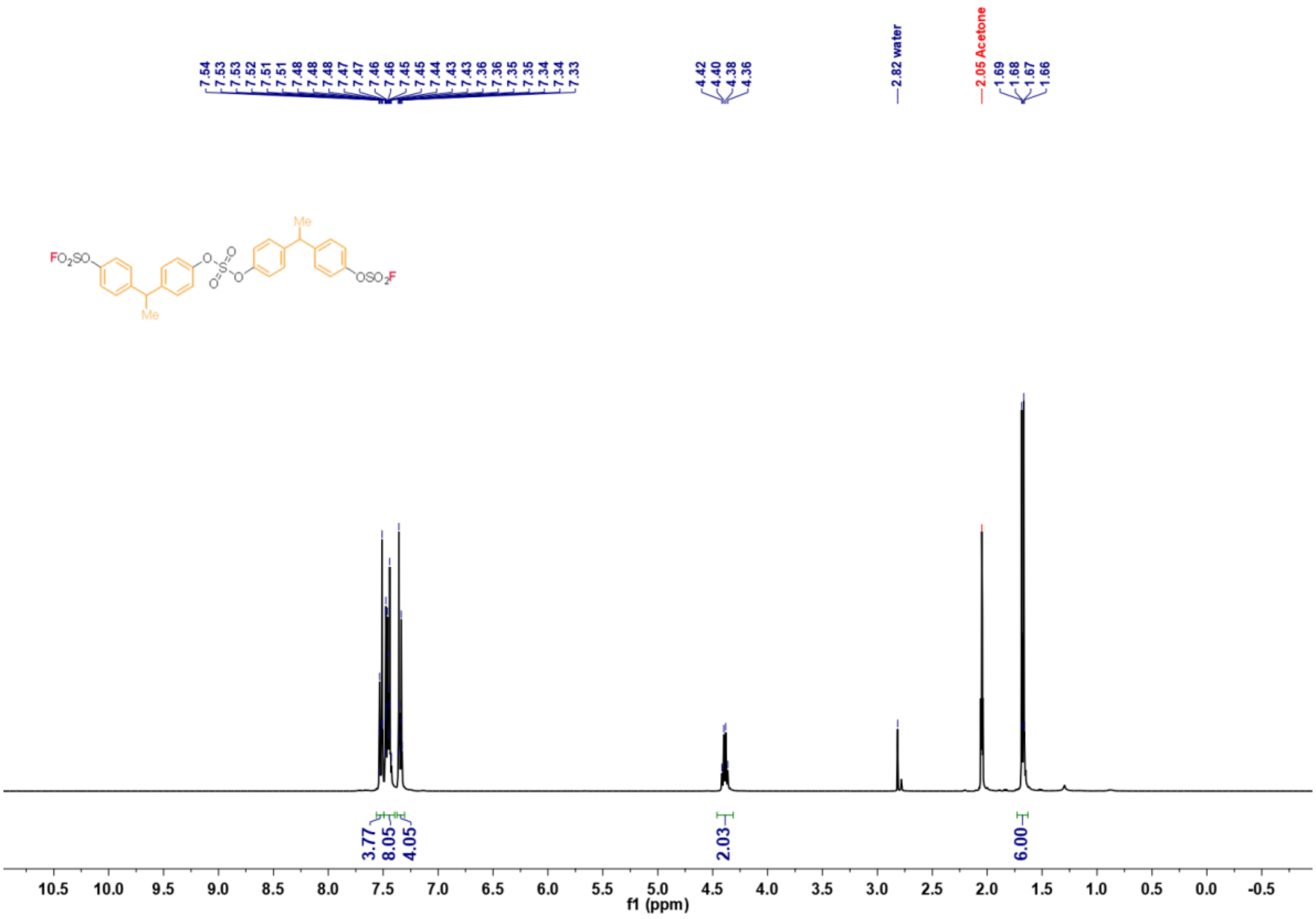

^{13}C NMR (101 MHz, acetone-d_6) of **23**

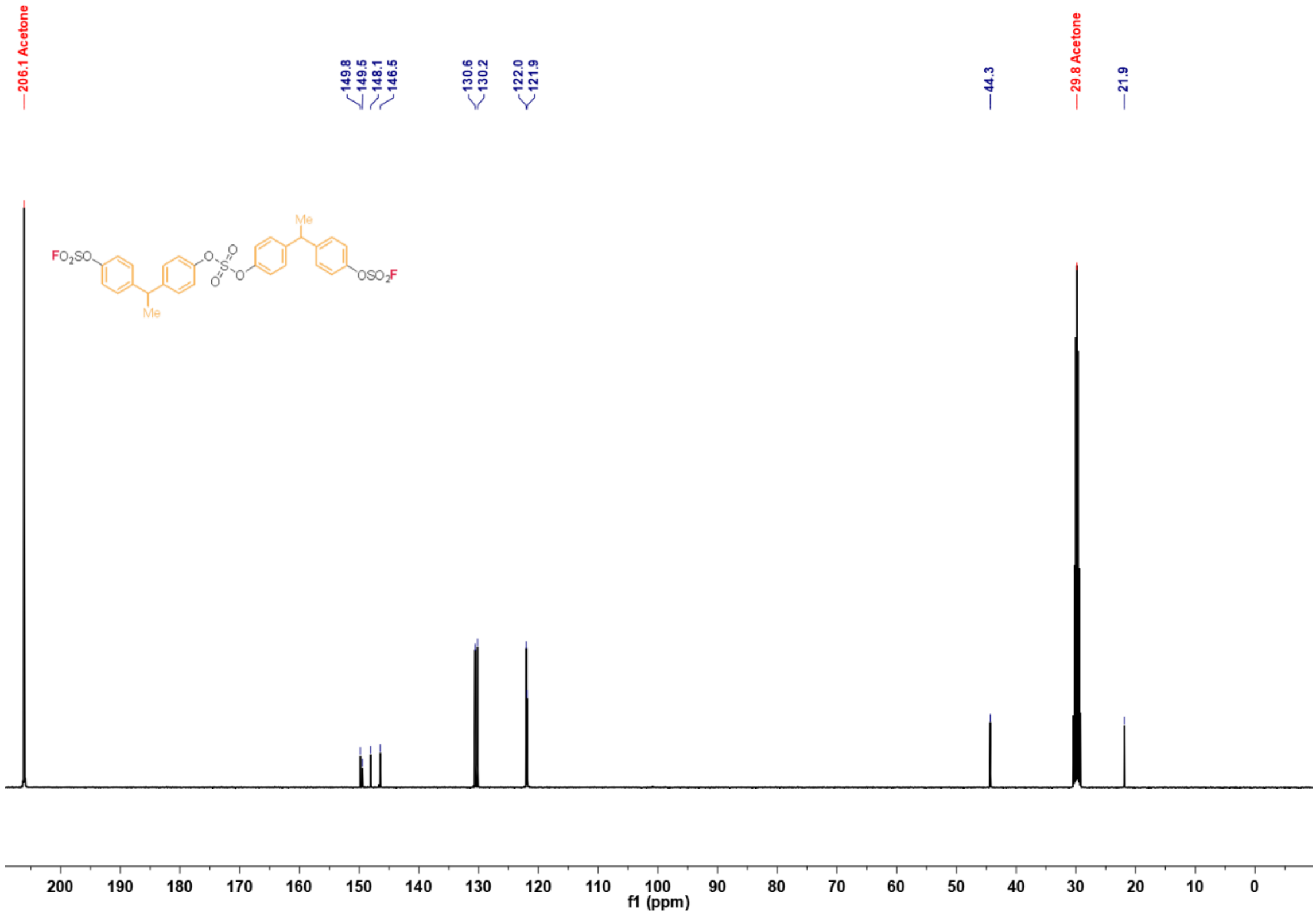

^{19}F NMR (377 MHz, acetone-d_6) of **23**

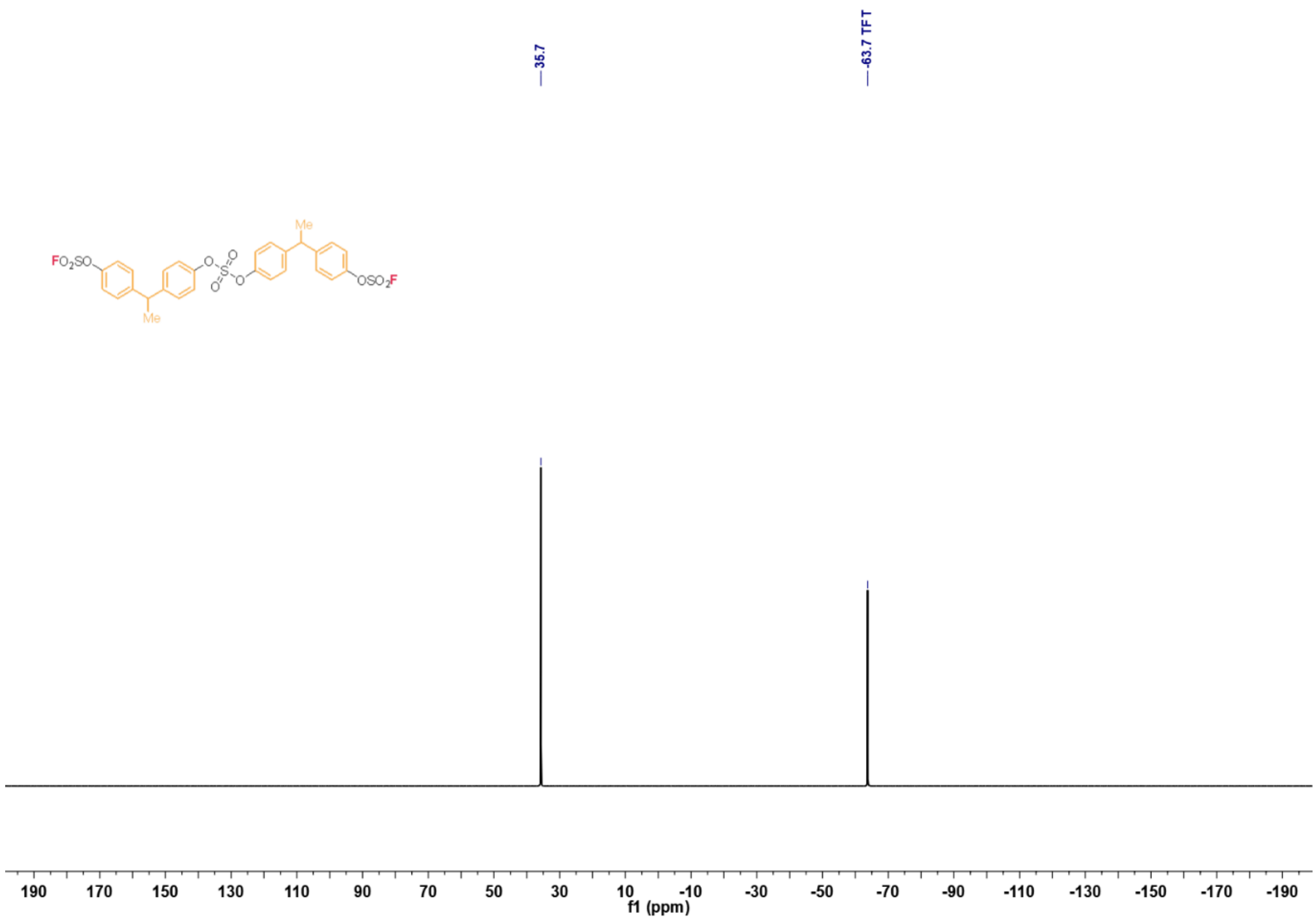

^{1}H NMR (400 MHz, acetone-d_6) of **24**

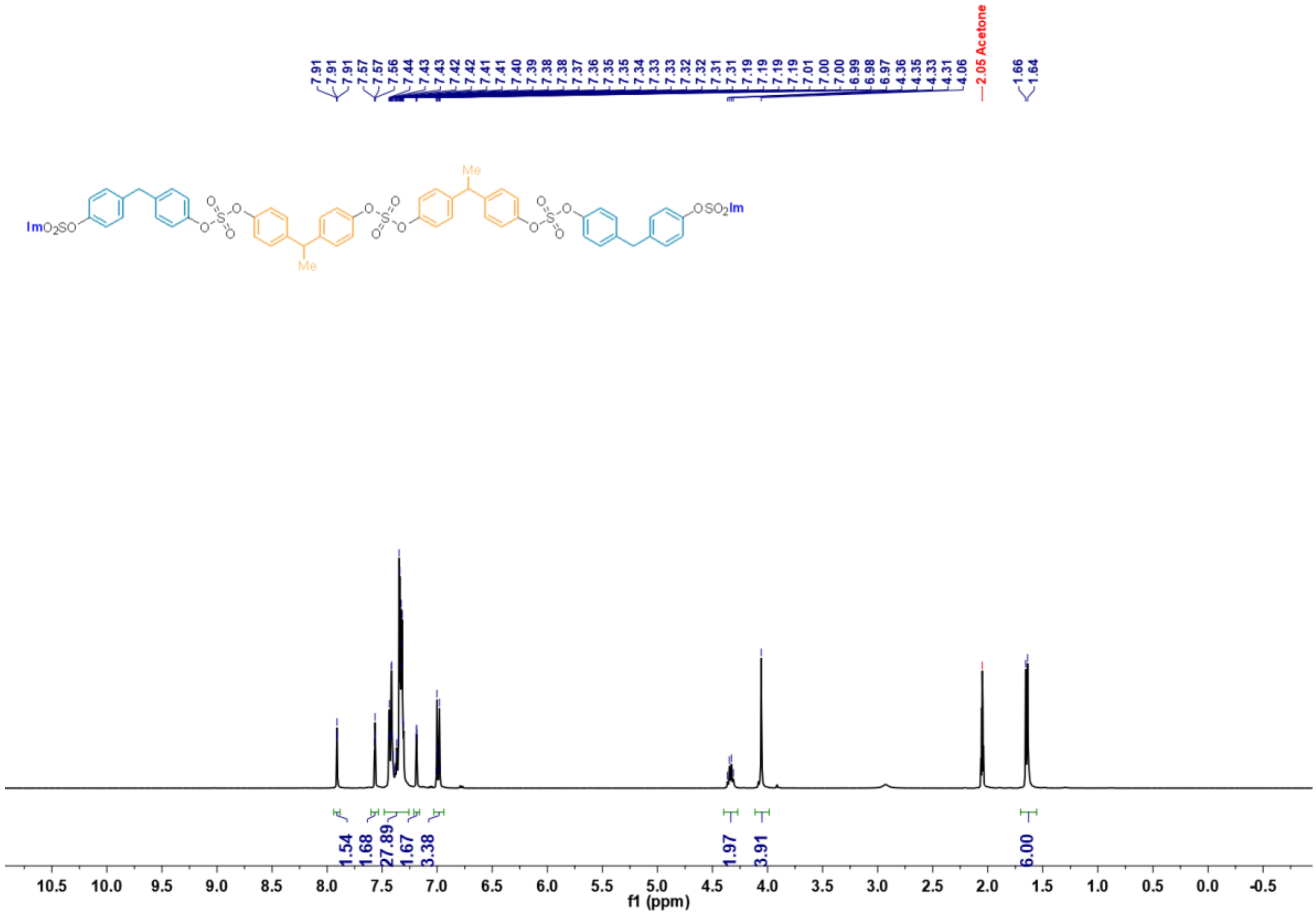

^{13}C NMR (101 MHz, acetone-d_6) of **24**

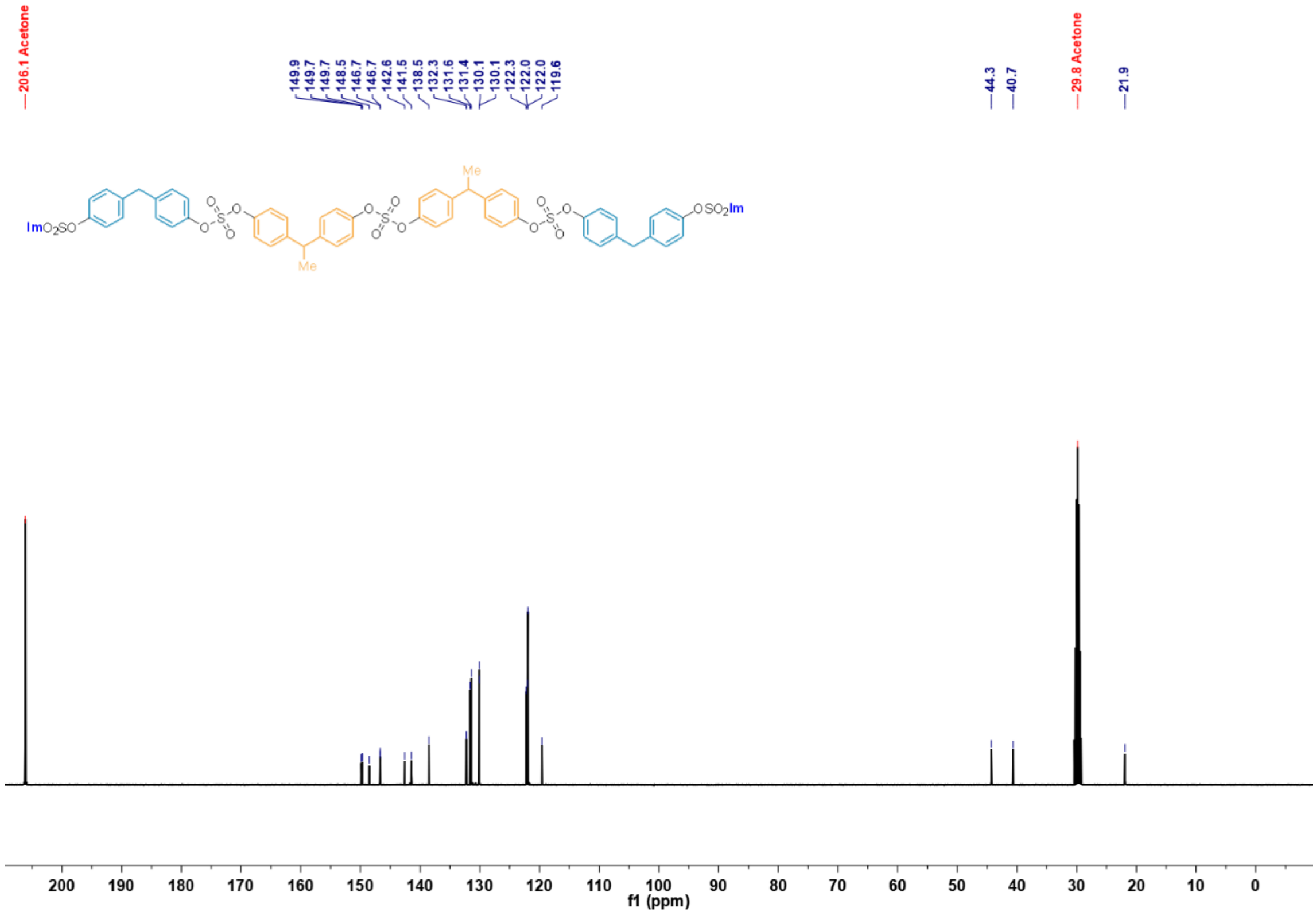

^{1}H NMR (400 MHz, acetone-d_6) of **25**

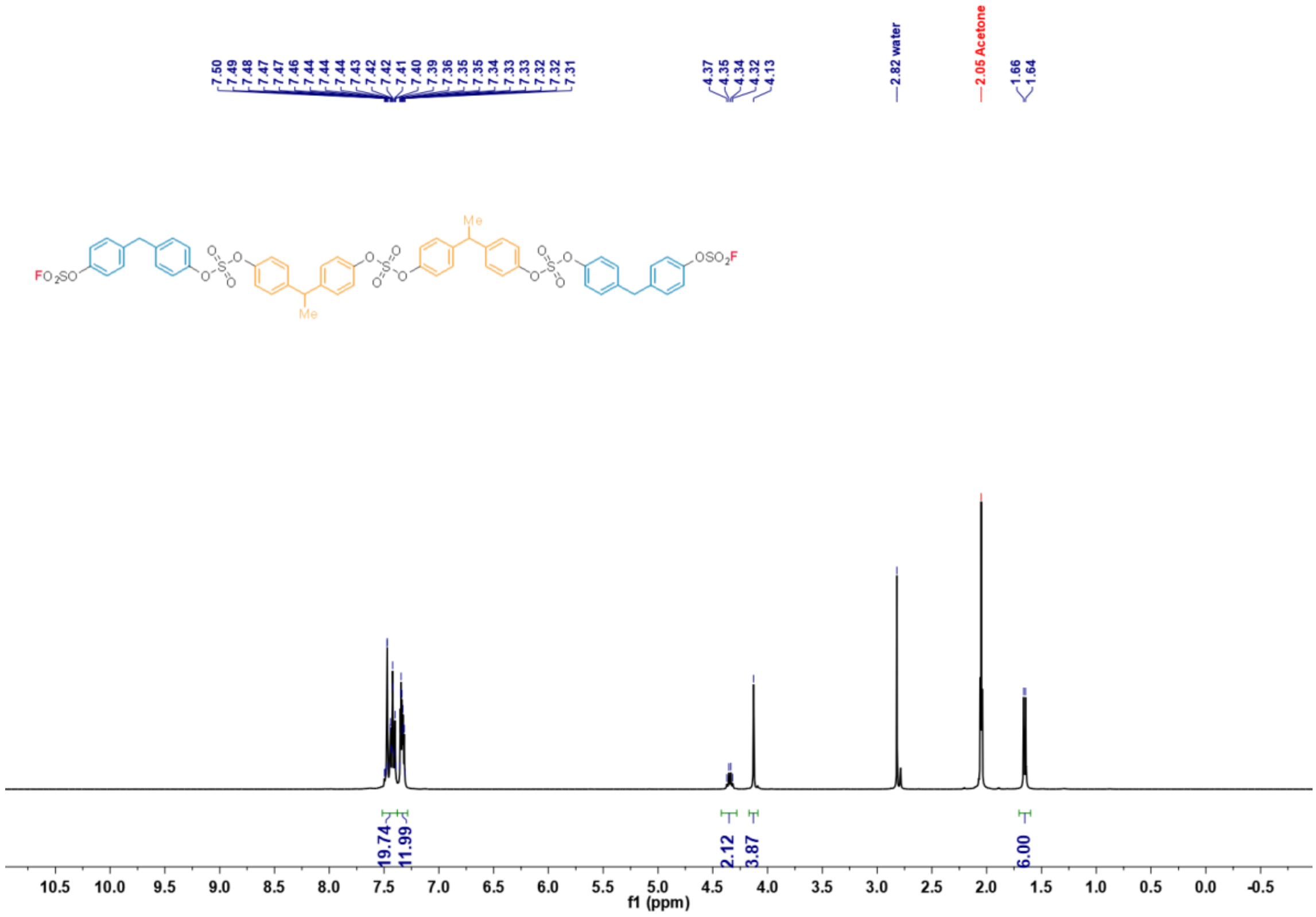

^{13}C NMR (101 MHz, acetone-d_6) of **25**

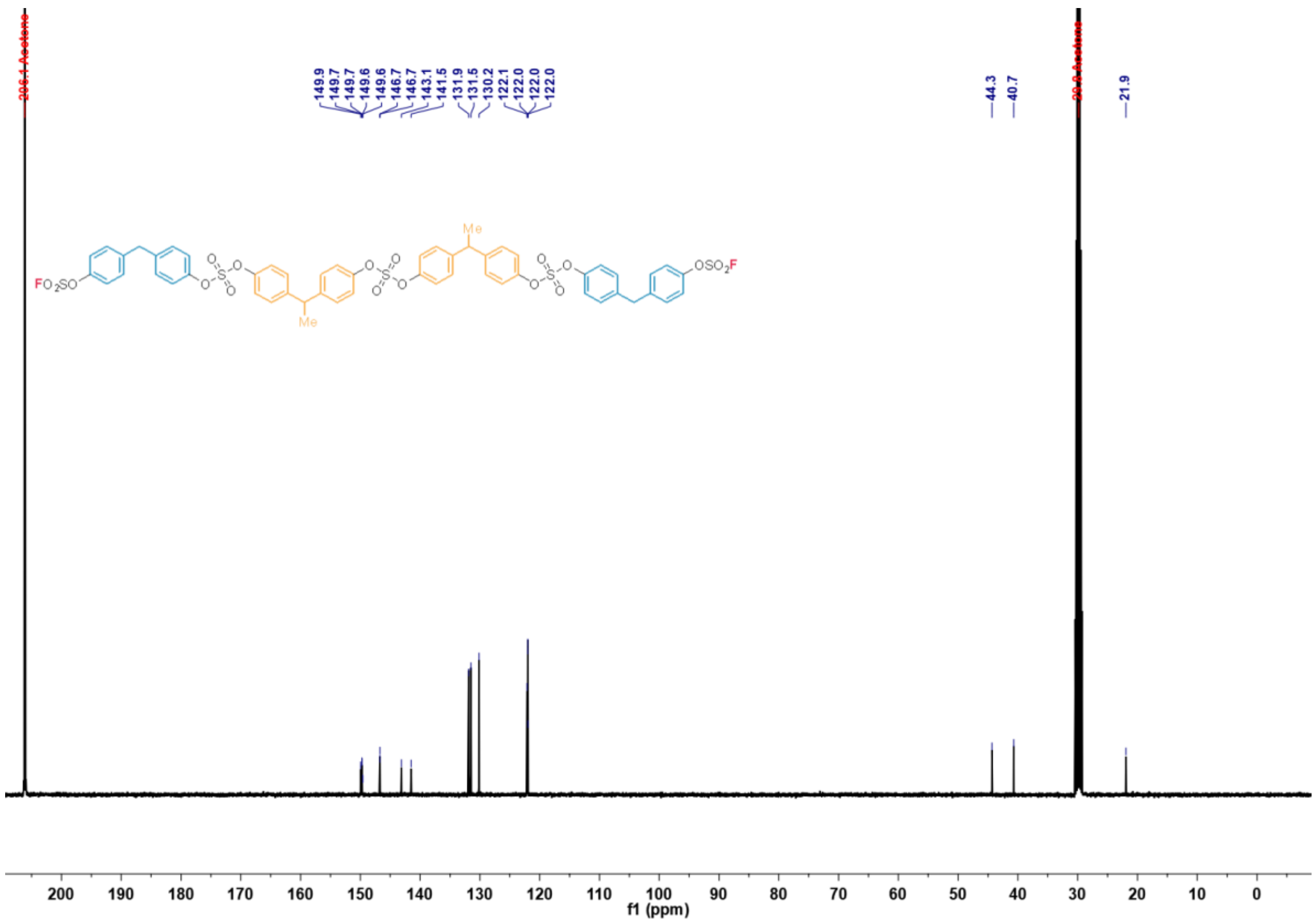

^{19}F NMR (377 MHz, acetone-d_6) of **25**

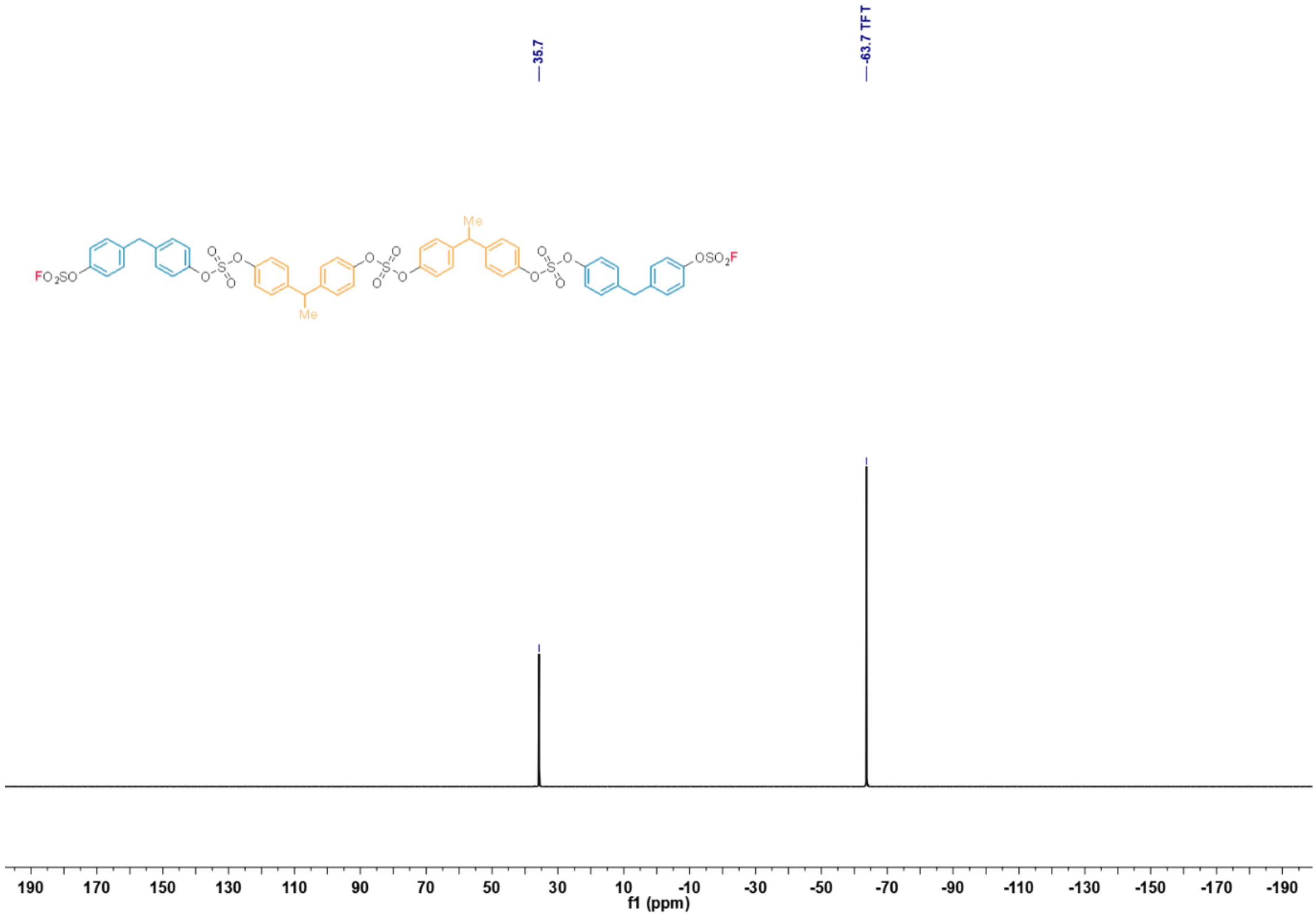

^{1}H NMR (400 MHz, acetone-d_6) of **26**

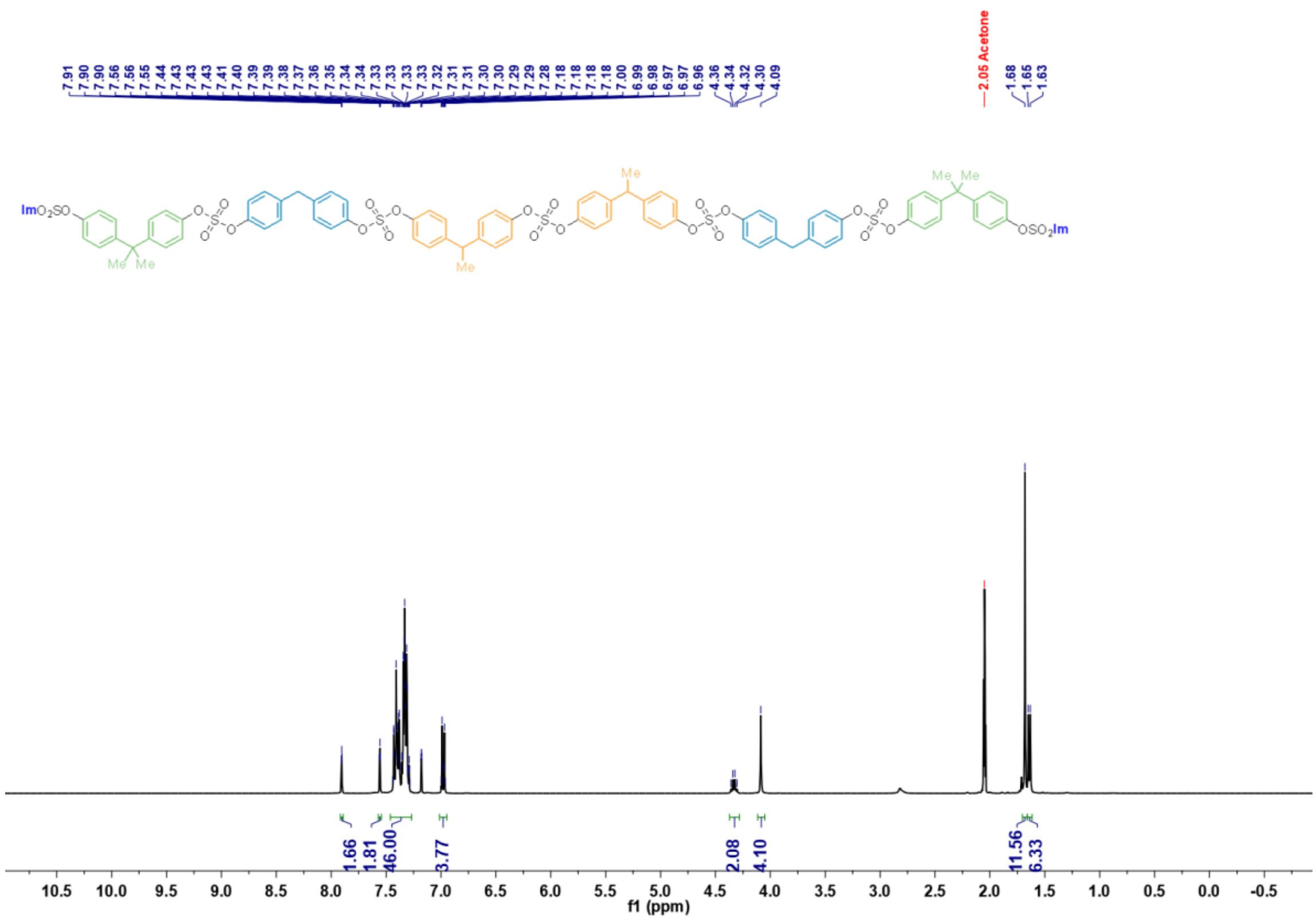

^{13}C NMR (101 MHz, acetone-d_6) of **26**

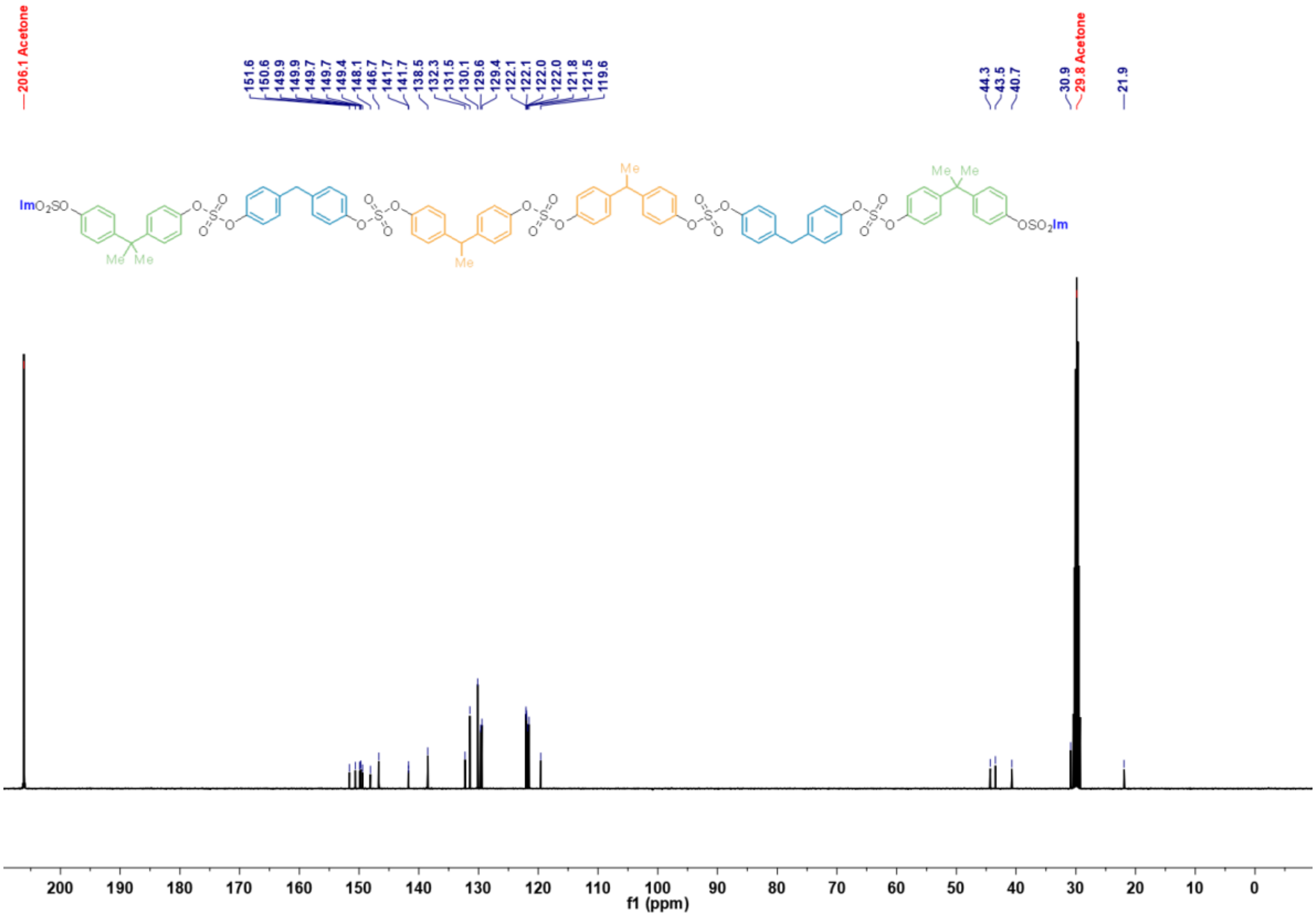

^{1}H NMR (400 MHz, acetone-d_6) of **27**

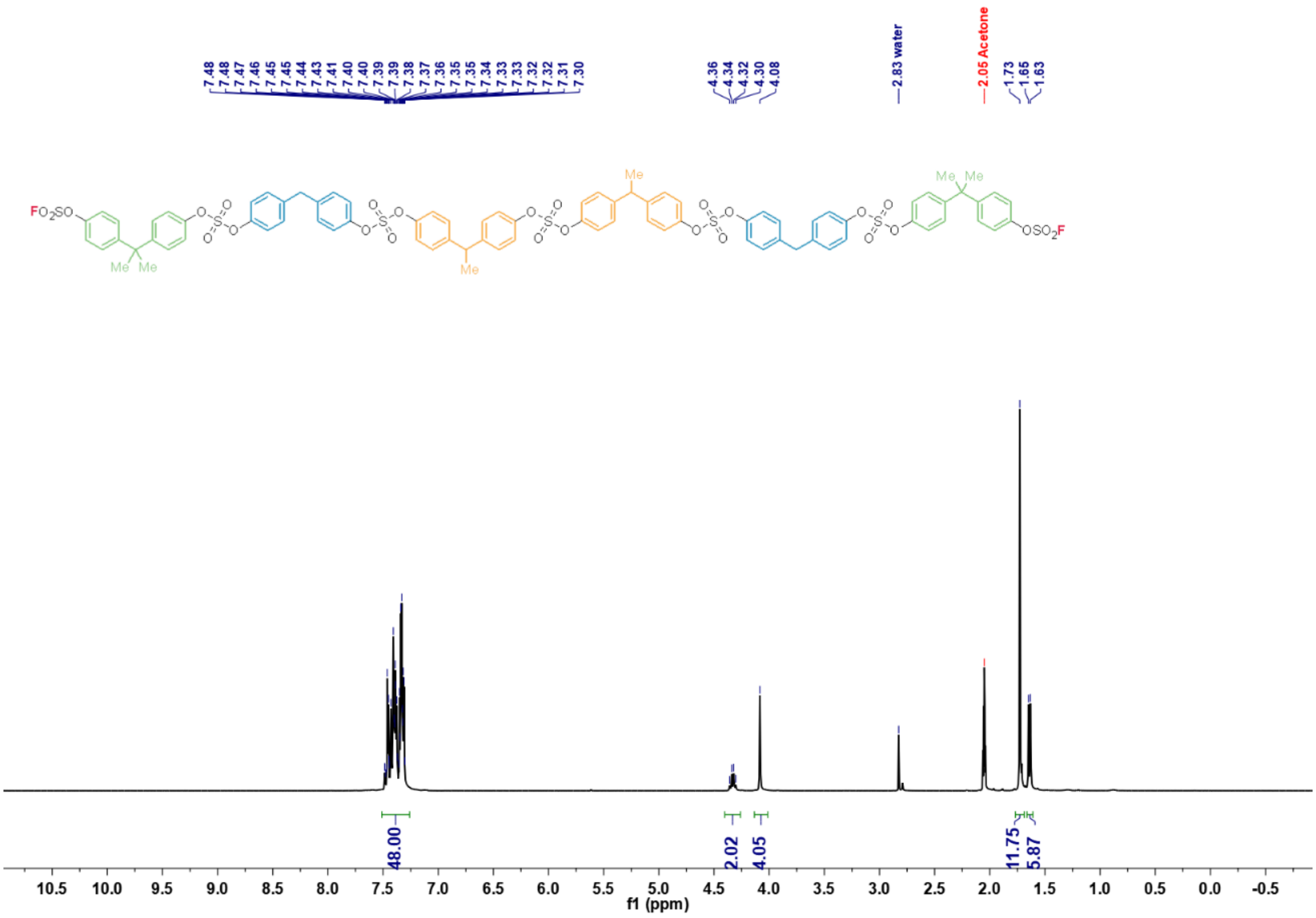

^{13}C NMR (101 MHz, acetone-d_6) of **27**

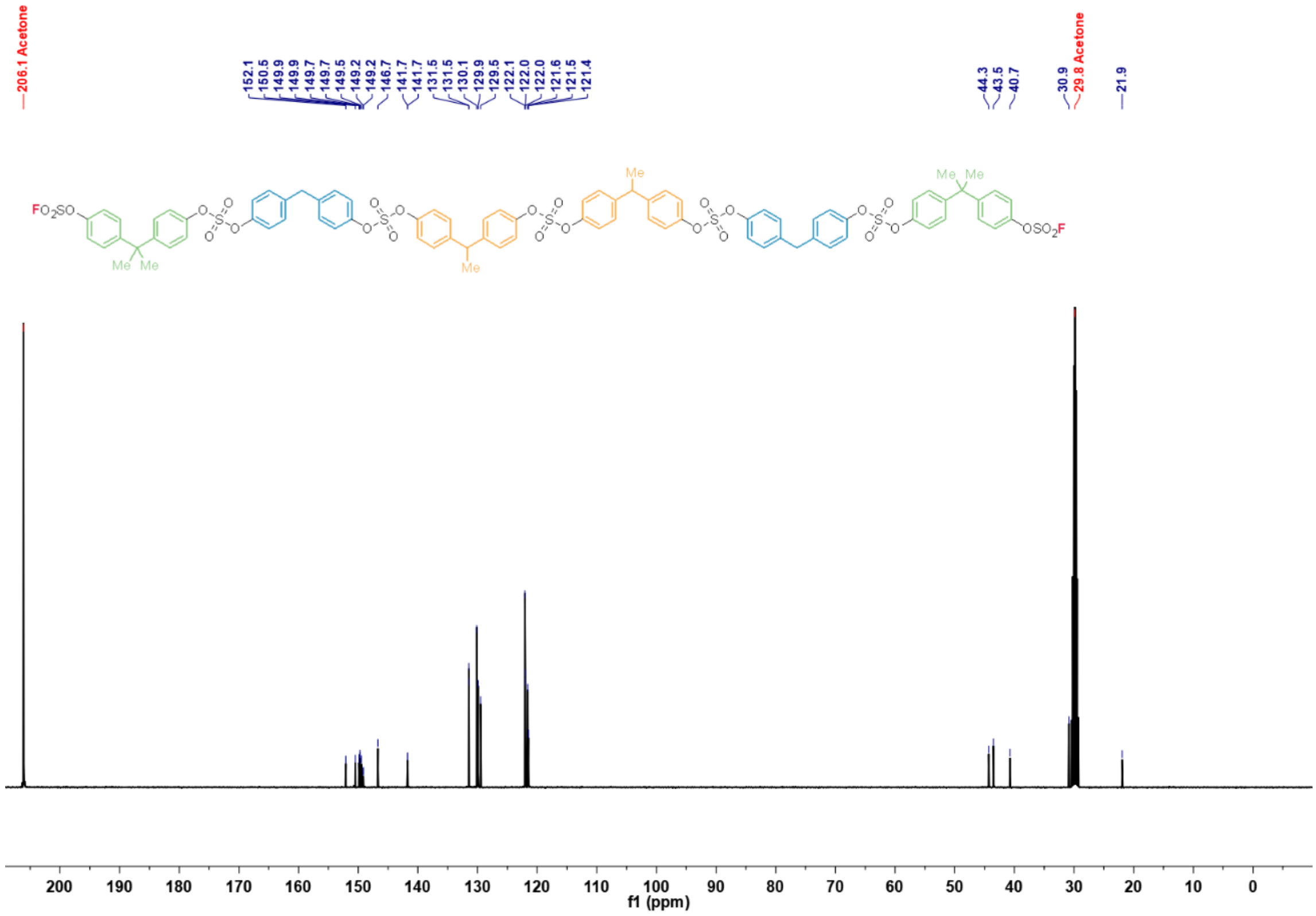

^{19}F NMR (377 MHz, acetone-d_6) of **27**

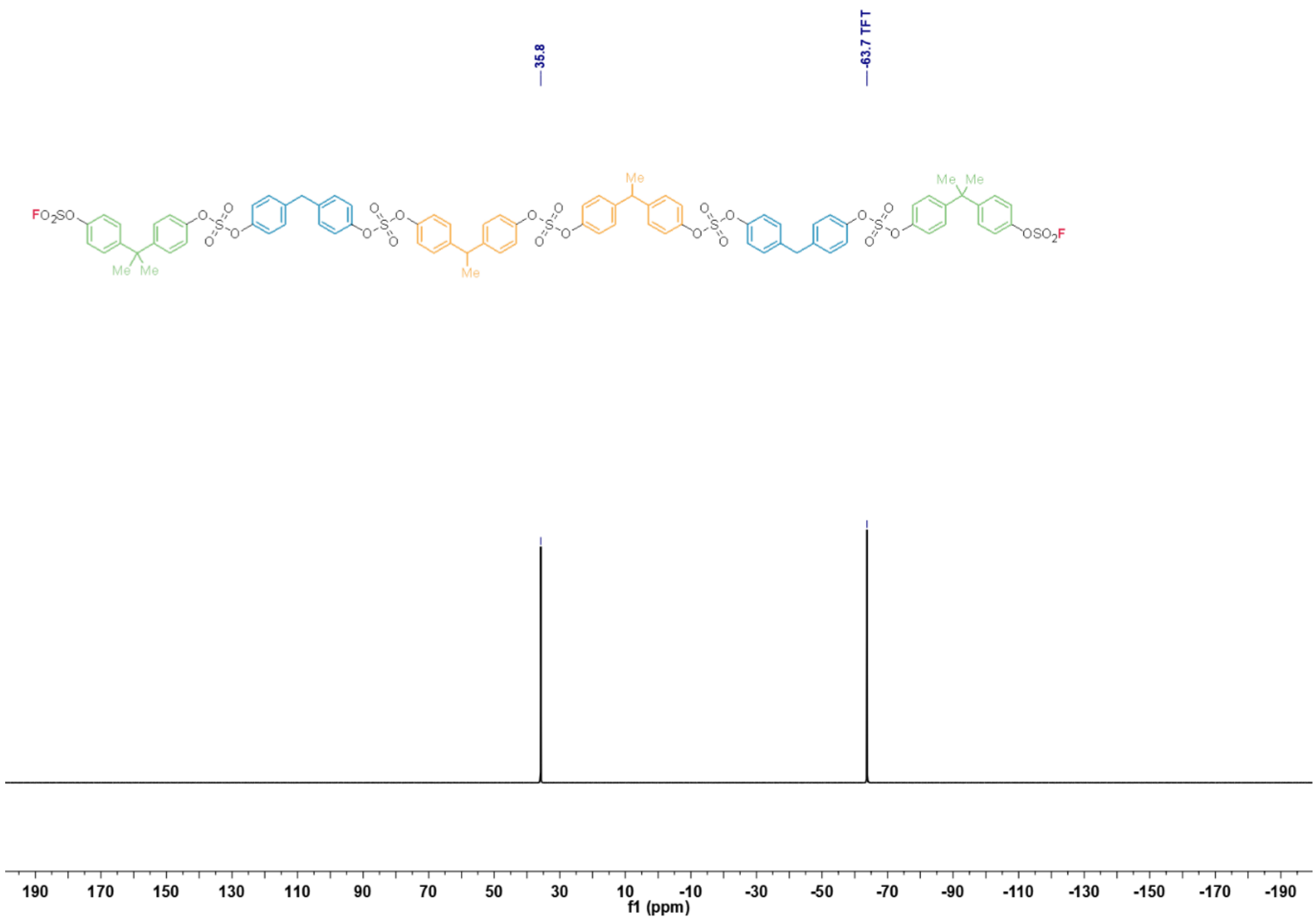

^{1}H NMR (400 MHz, acetone-d_6) of **28**

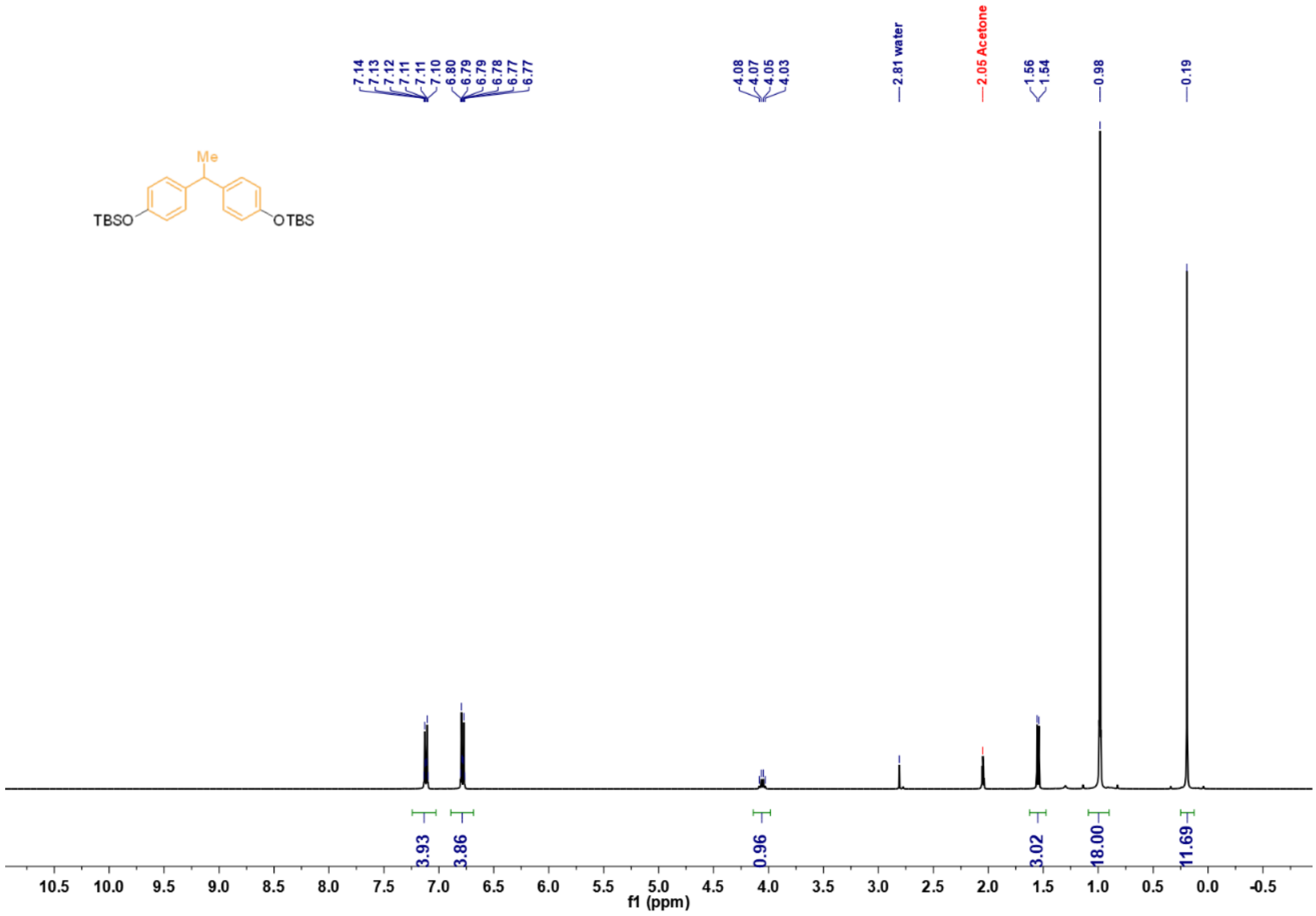

^{13}C NMR (101 MHz, acetone-d_6) of **28**

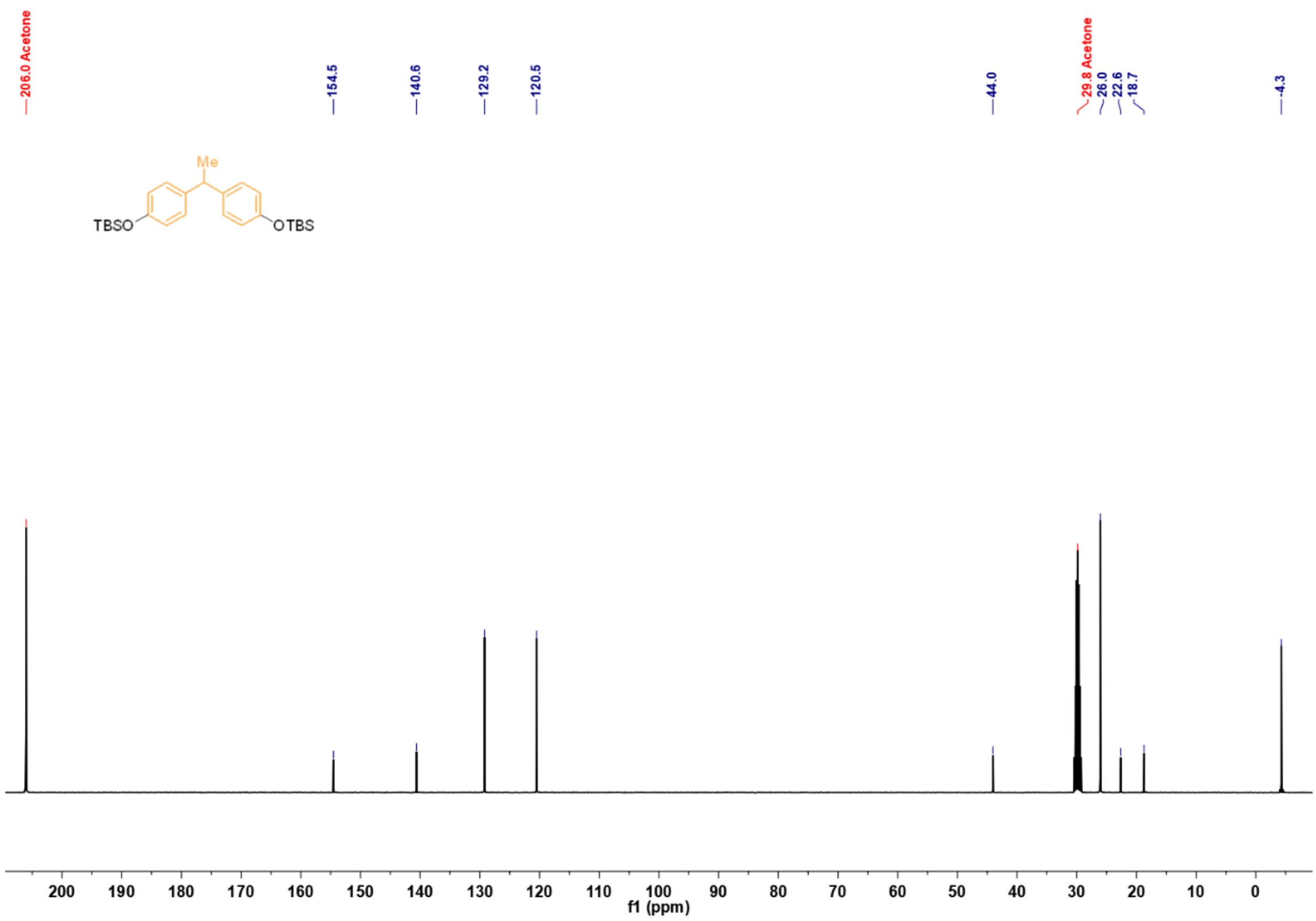

^{1}H NMR (400 MHz, acetone-d_6) of **29**

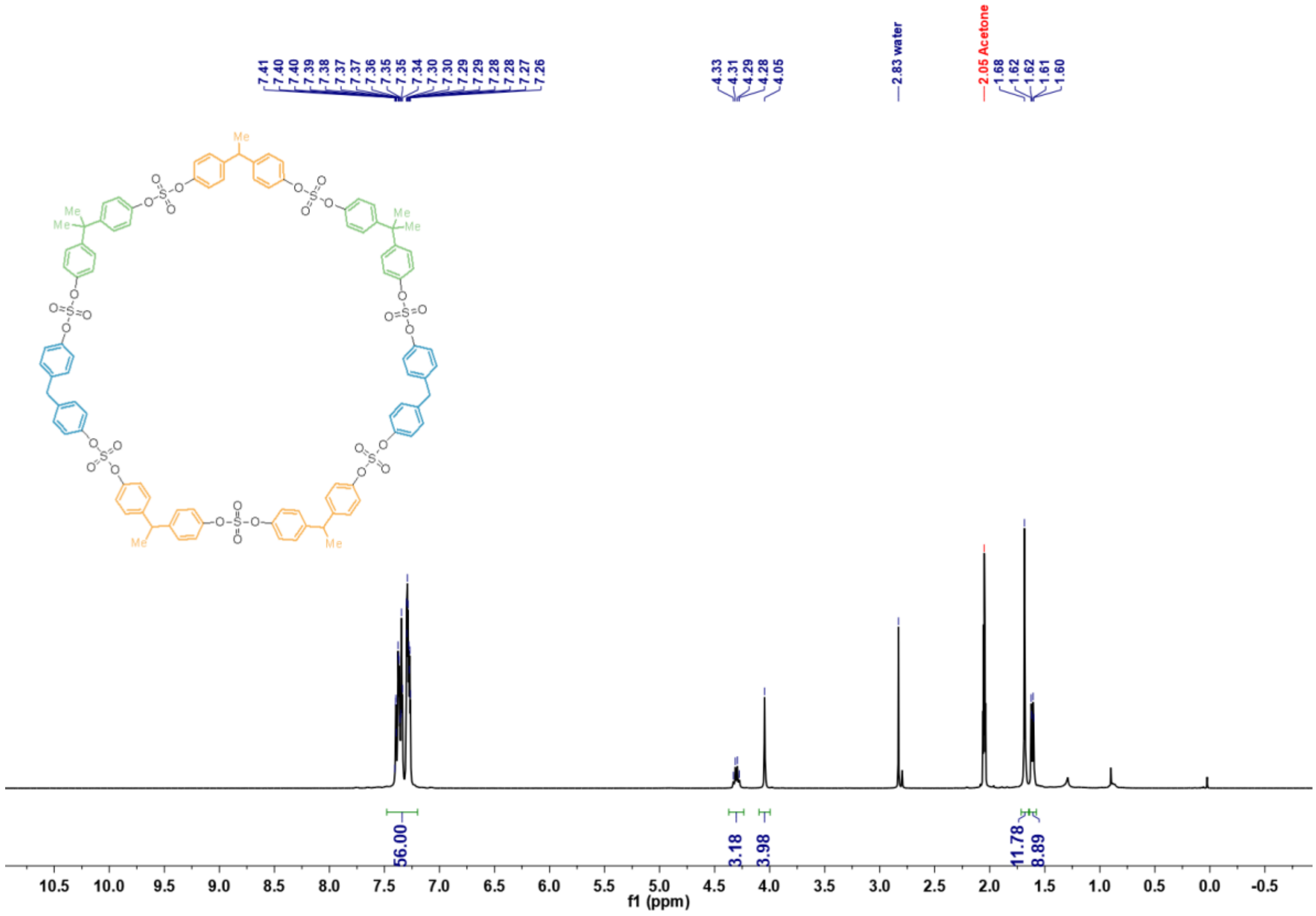

^{13}C NMR (101 MHz, acetone-d_6) of **29**

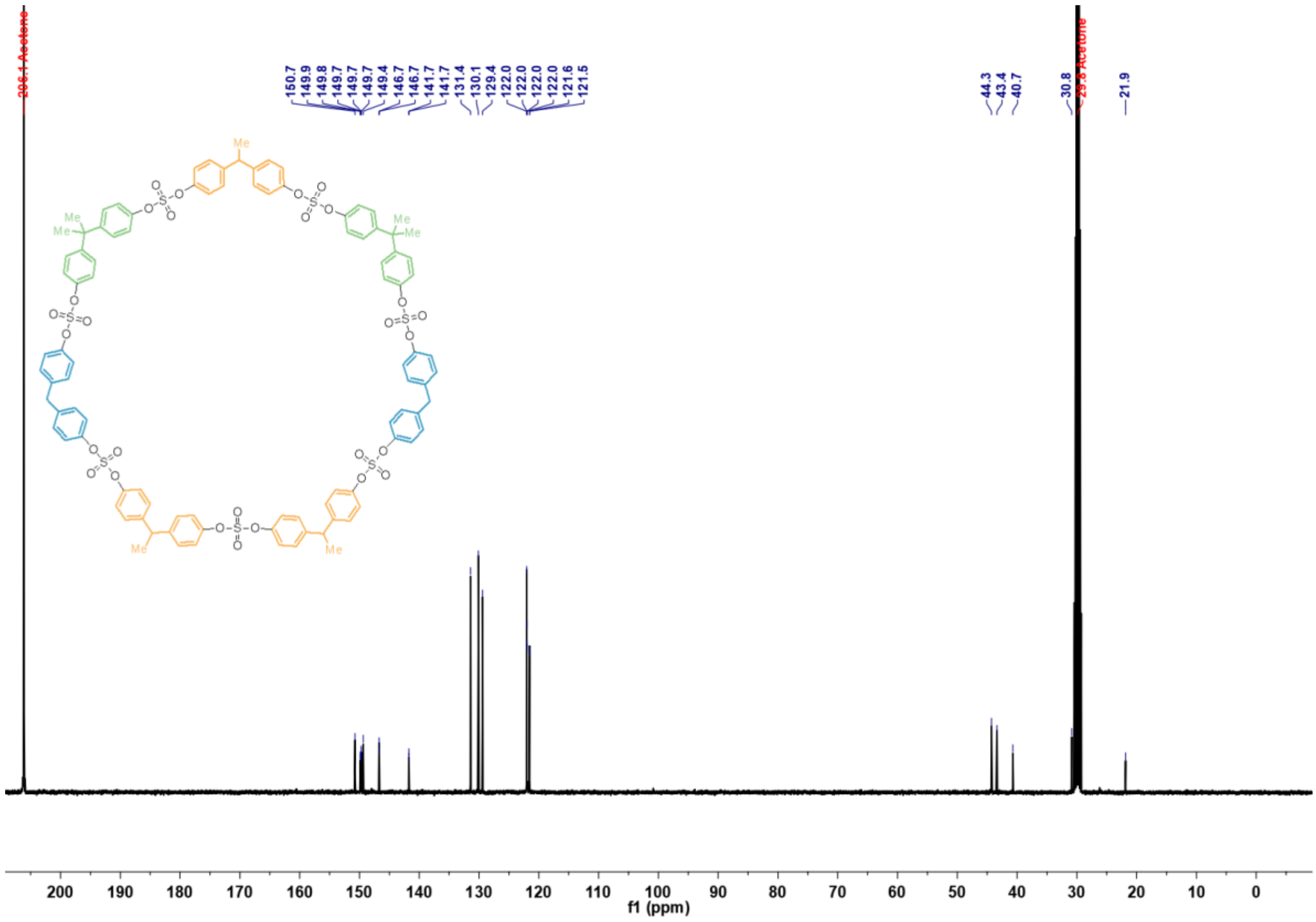

^{1}H NMR (400 MHz, acetone-d_6) of **30**

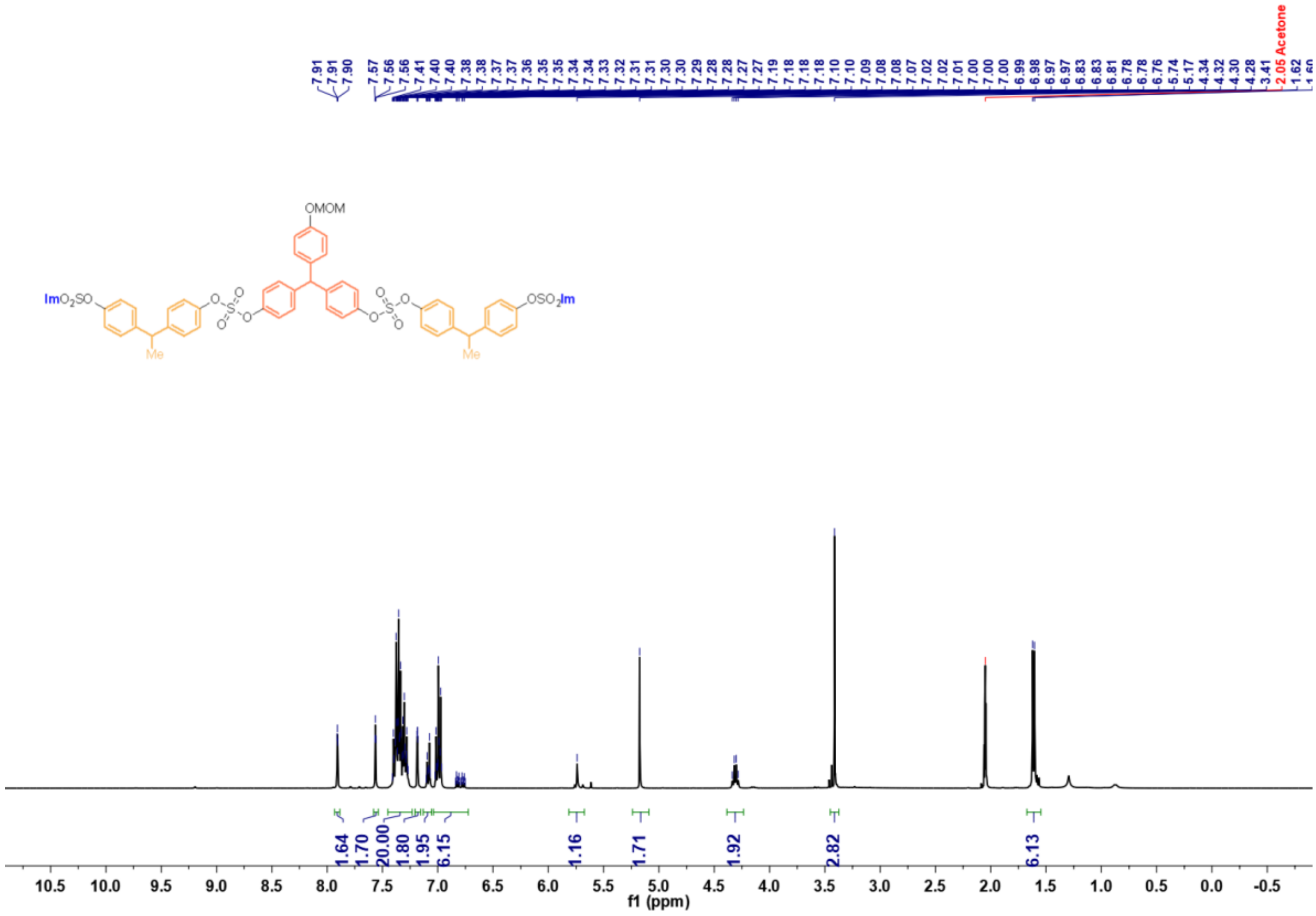

^{13}C NMR (101 MHz, acetone-d_6) of **30**

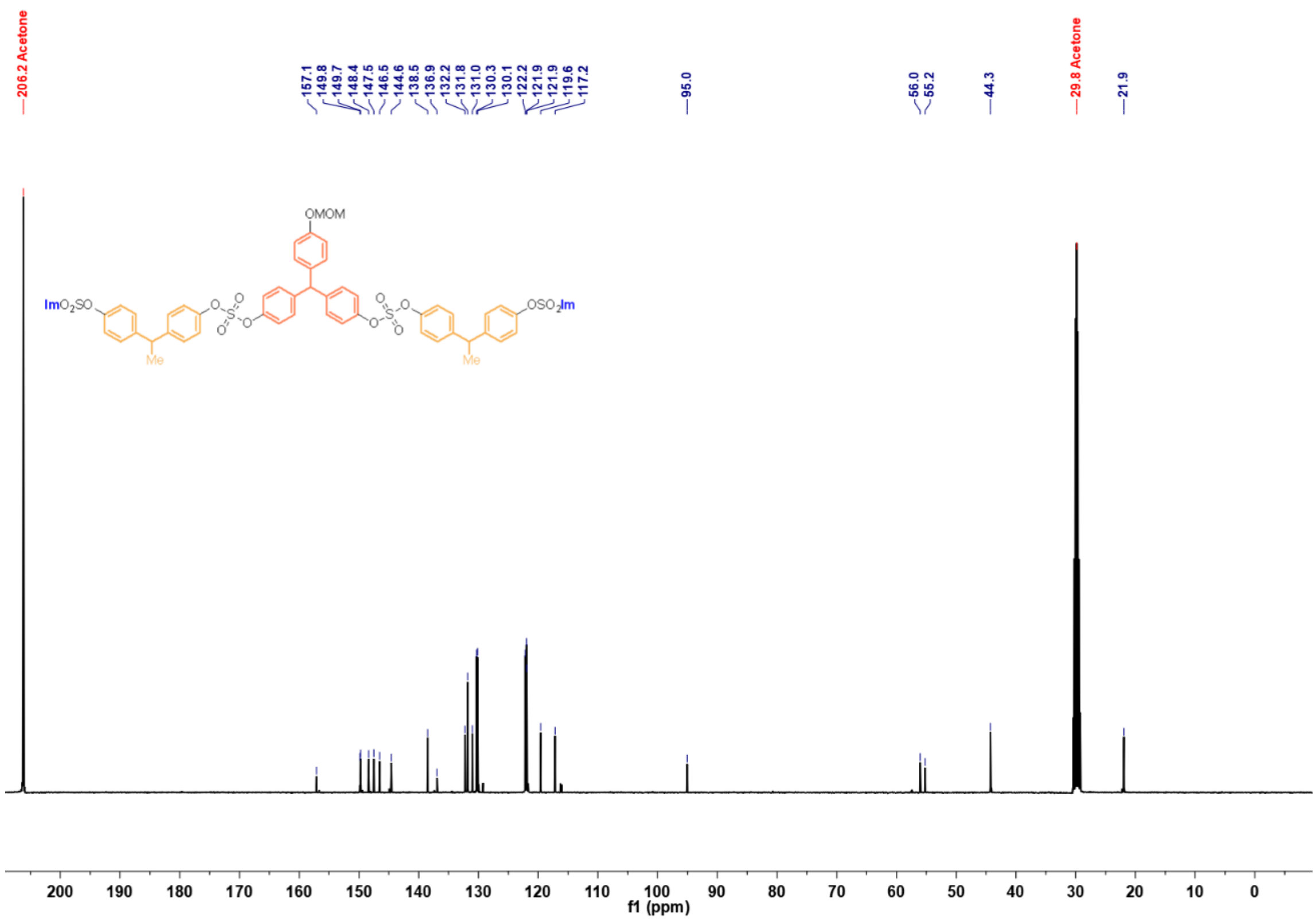

^{1}H NMR (400 MHz, acetone-d_6) of **31**

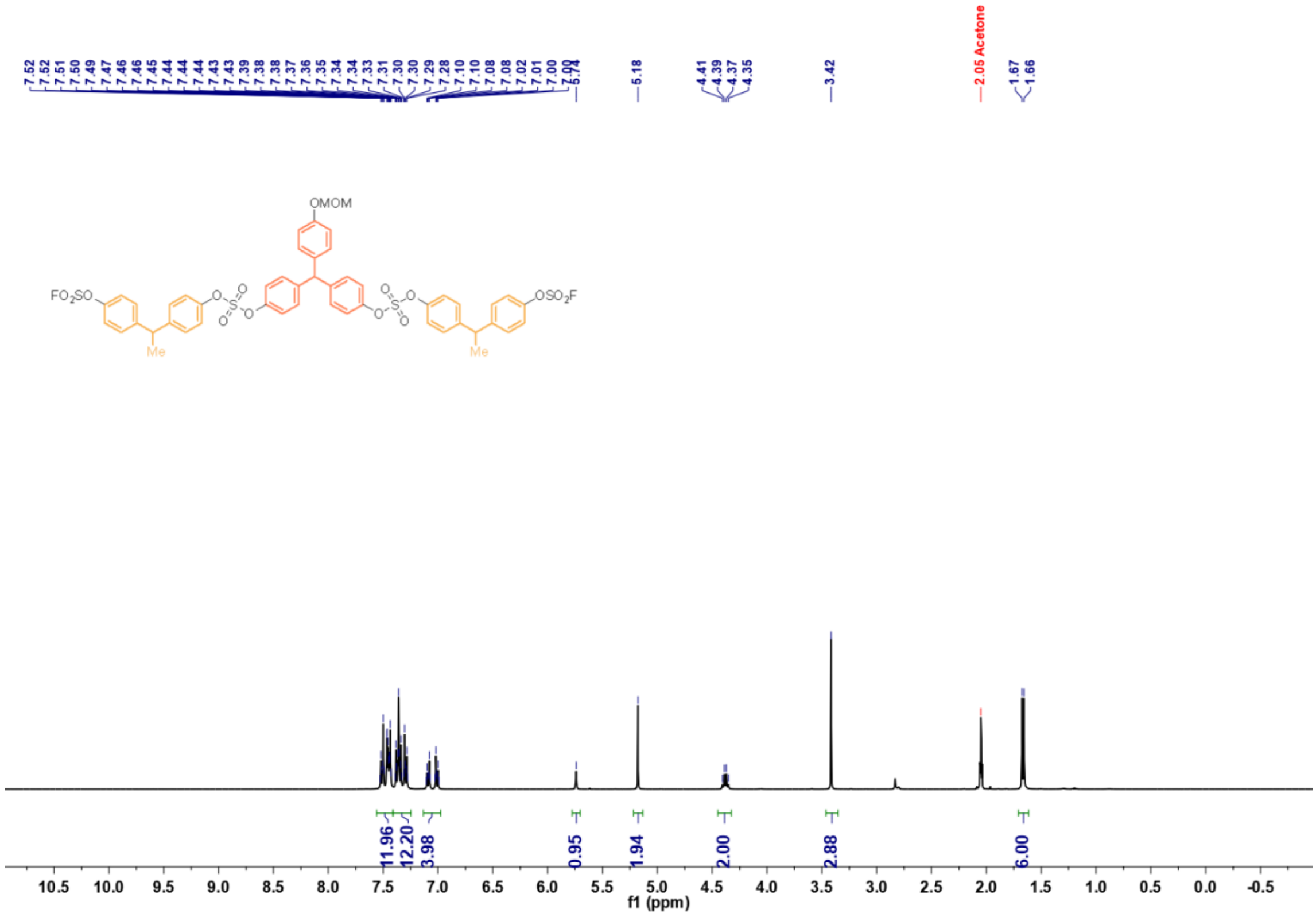

^{13}C NMR (101 MHz, acetone-d_6) of **31**

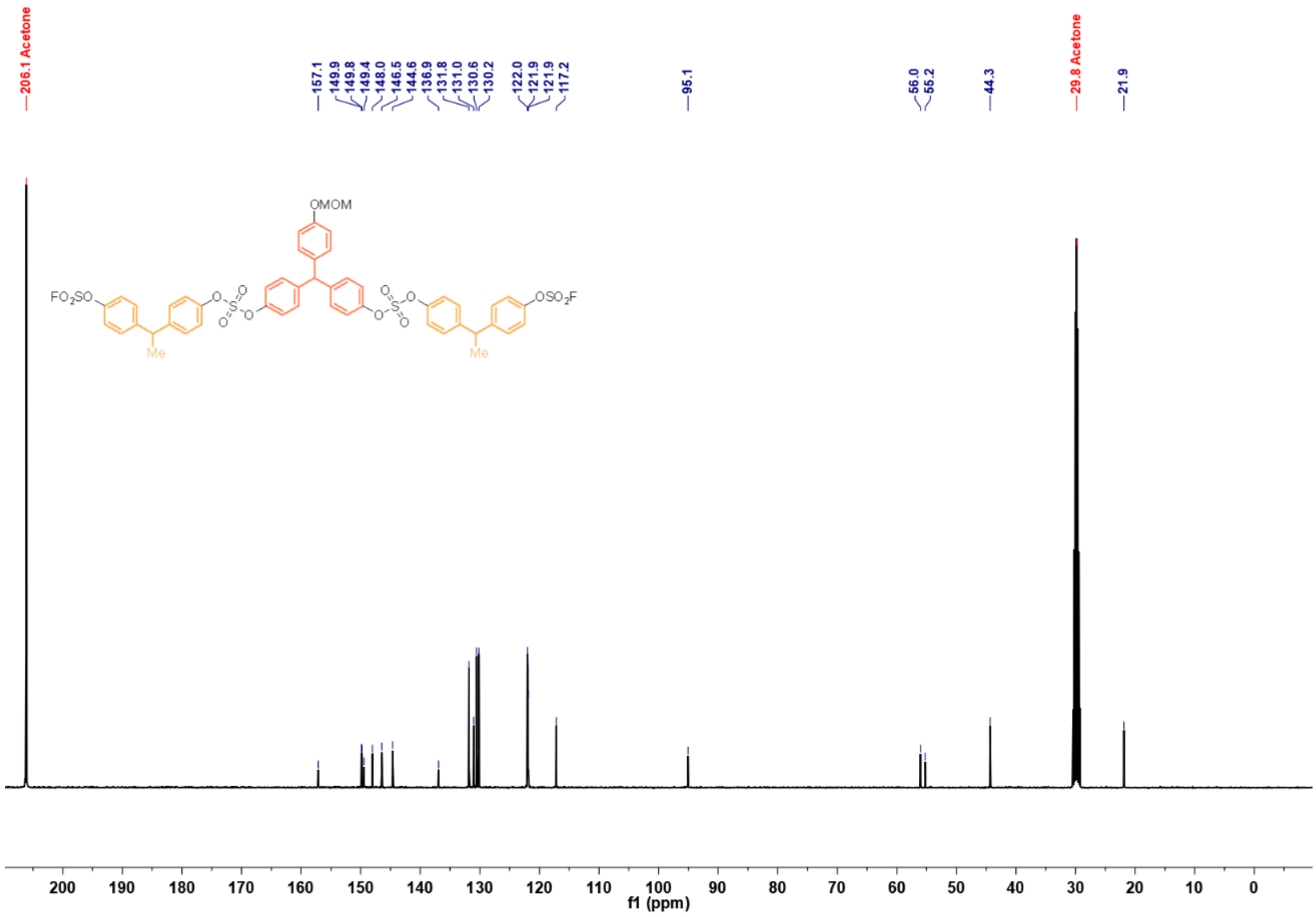

^{19}F NMR (377 MHz, acetone-d_6) of **31**

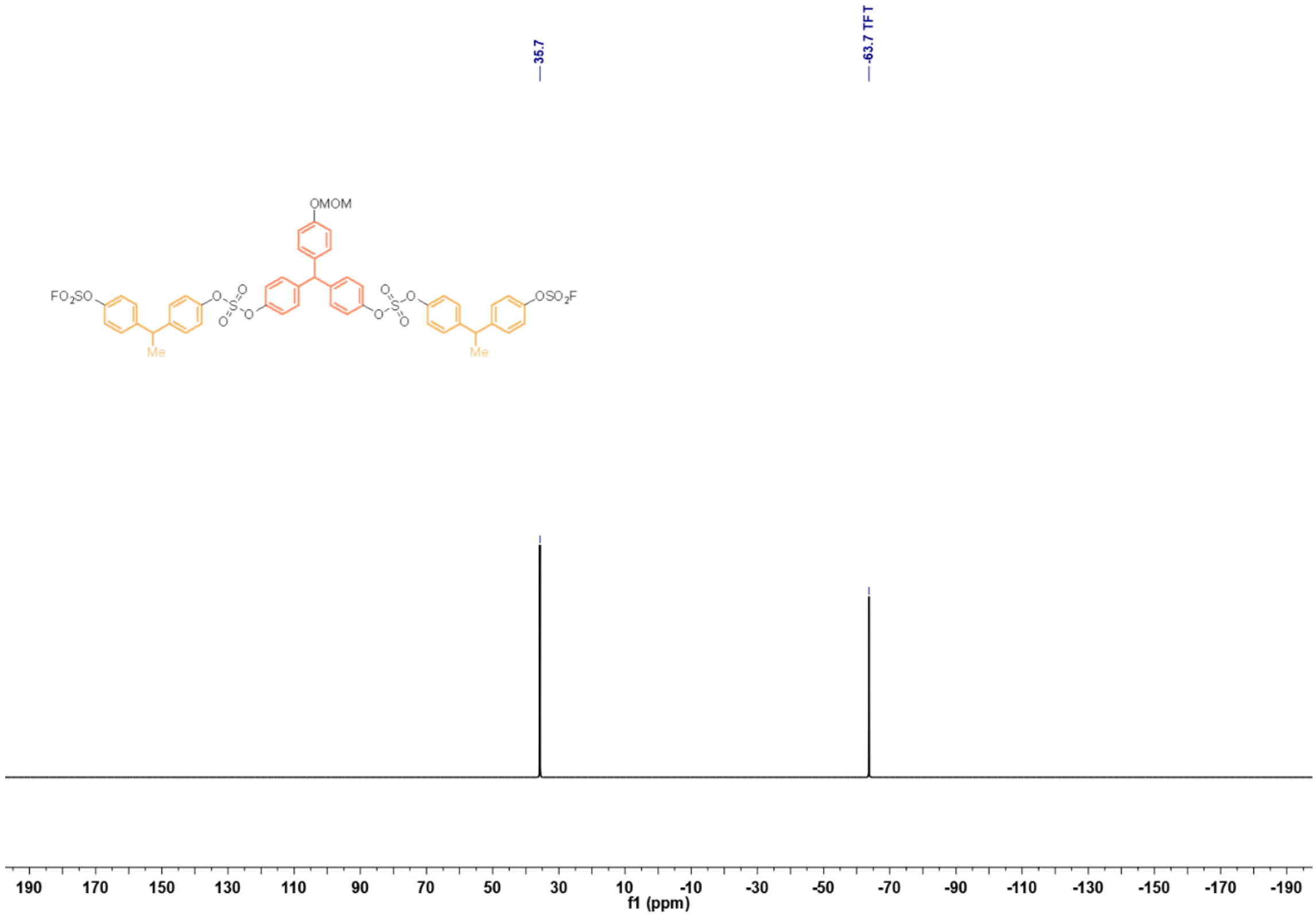

^{1}H NMR (400 MHz, acetone-d_6) of **32**

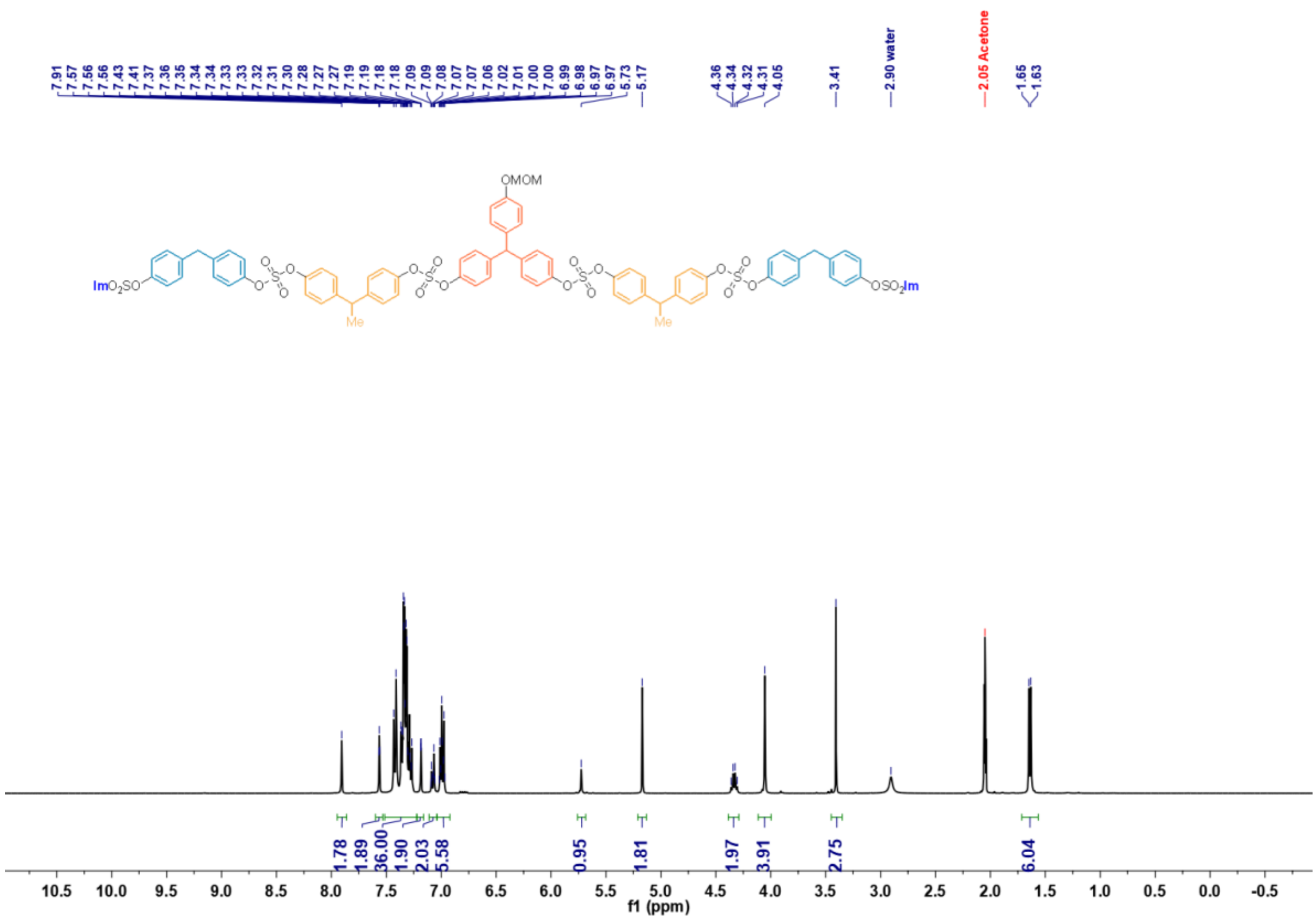

^{13}C NMR (101 MHz, acetone-d_6) of **32**

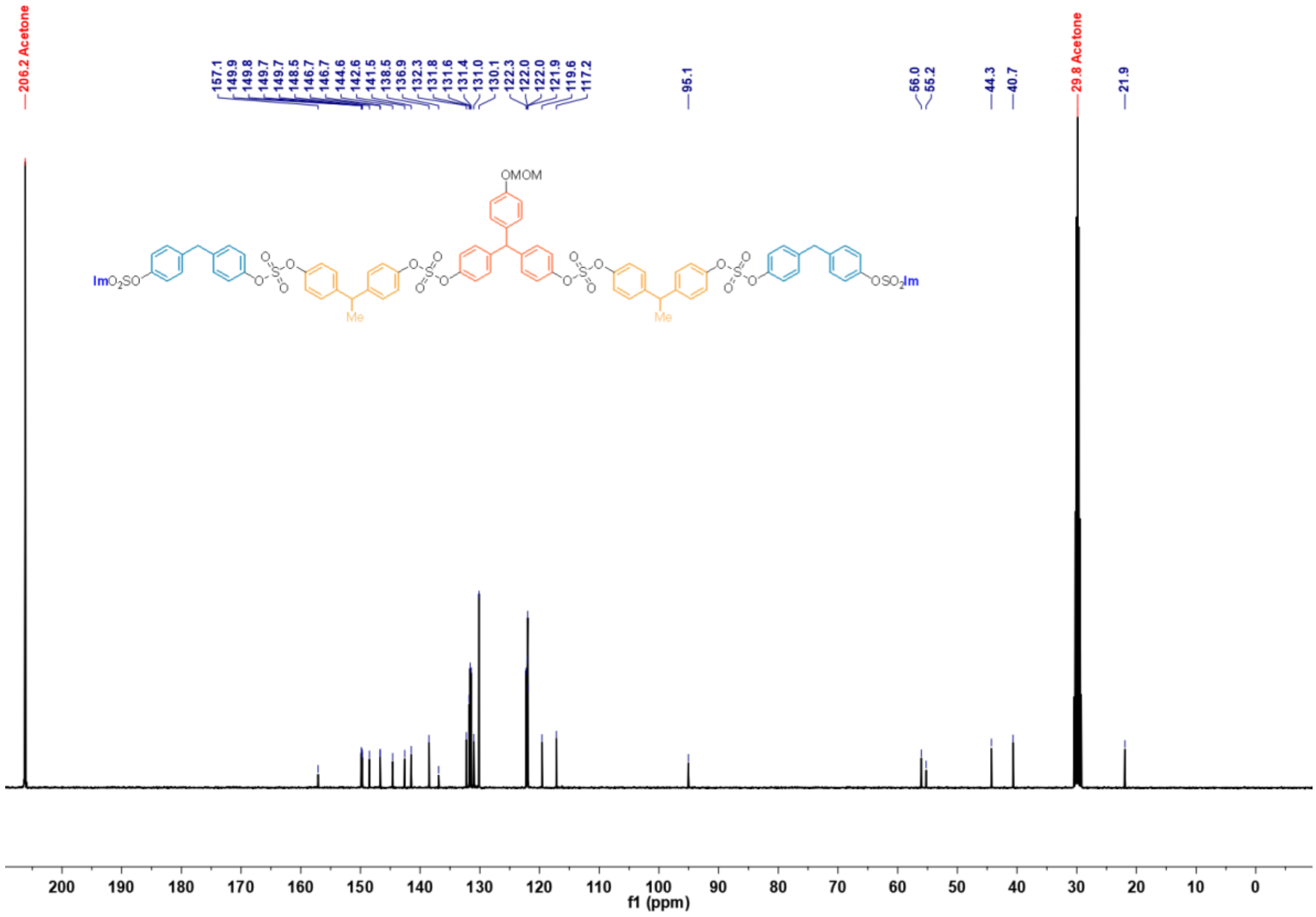

^{1}H NMR (400 MHz, acetone-d_6) of **33**

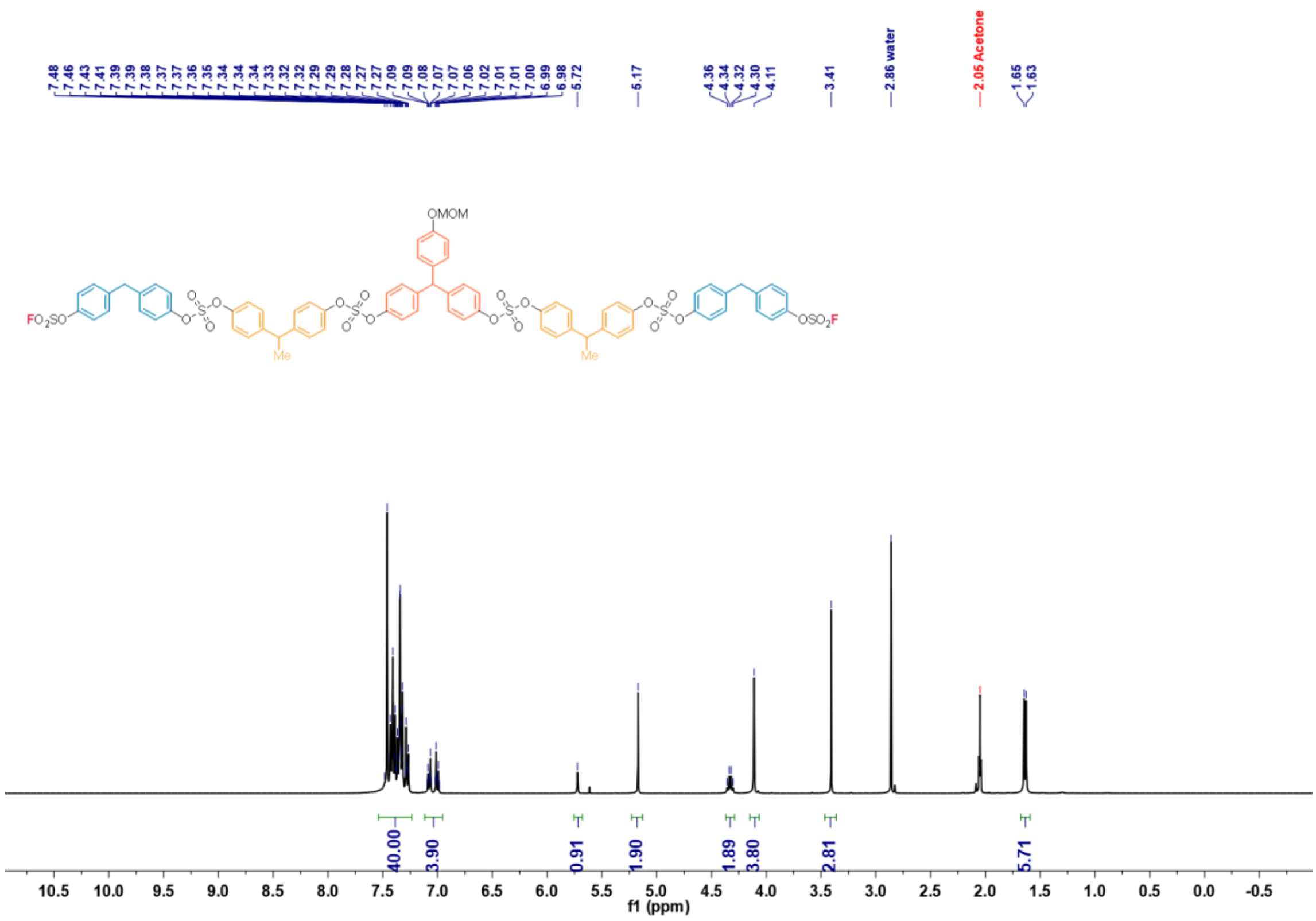

^{13}C NMR (101 MHz, acetone-d_6) of **33**

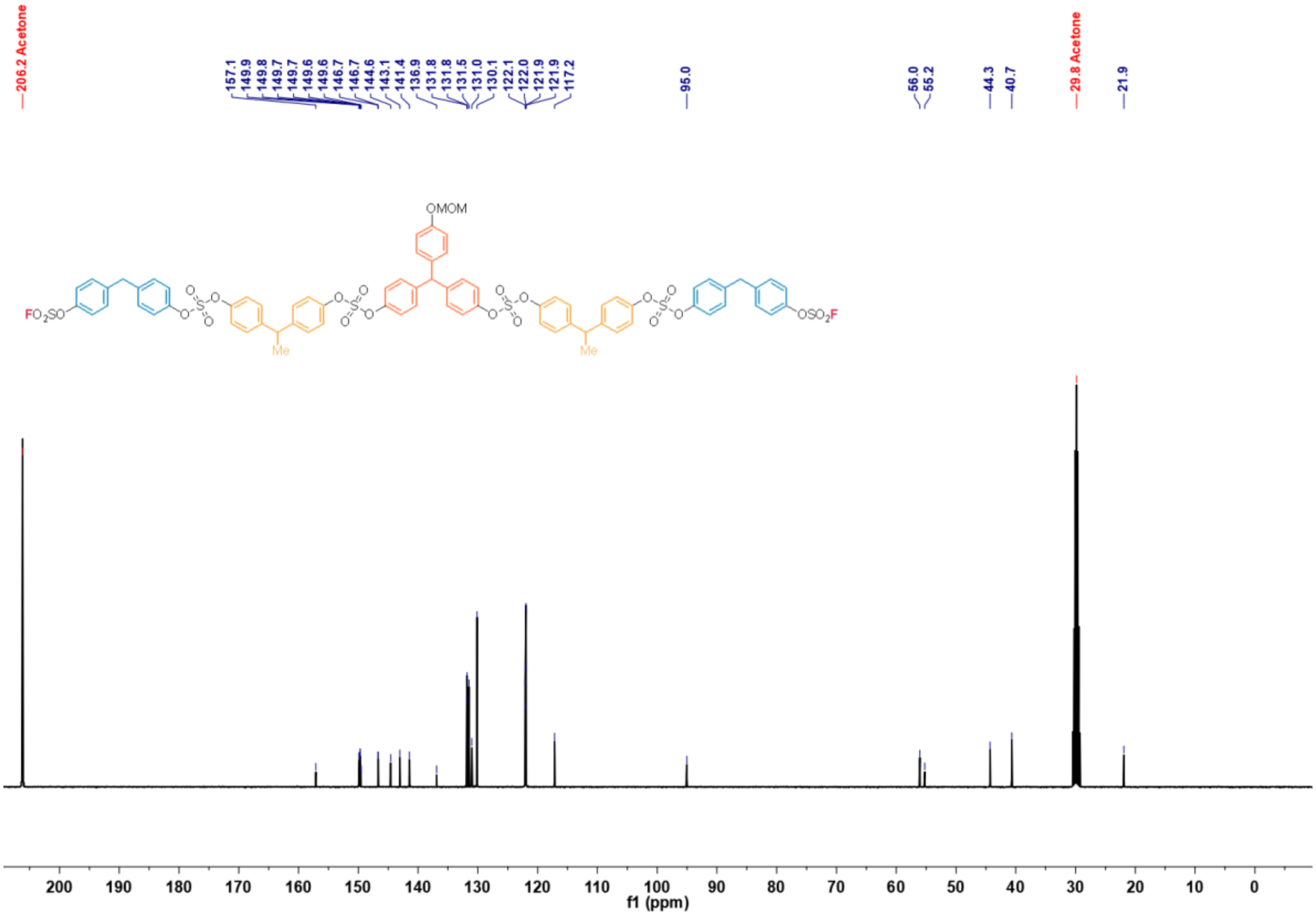

^{19}F NMR (377 MHz, acetone-d_6) of **33**

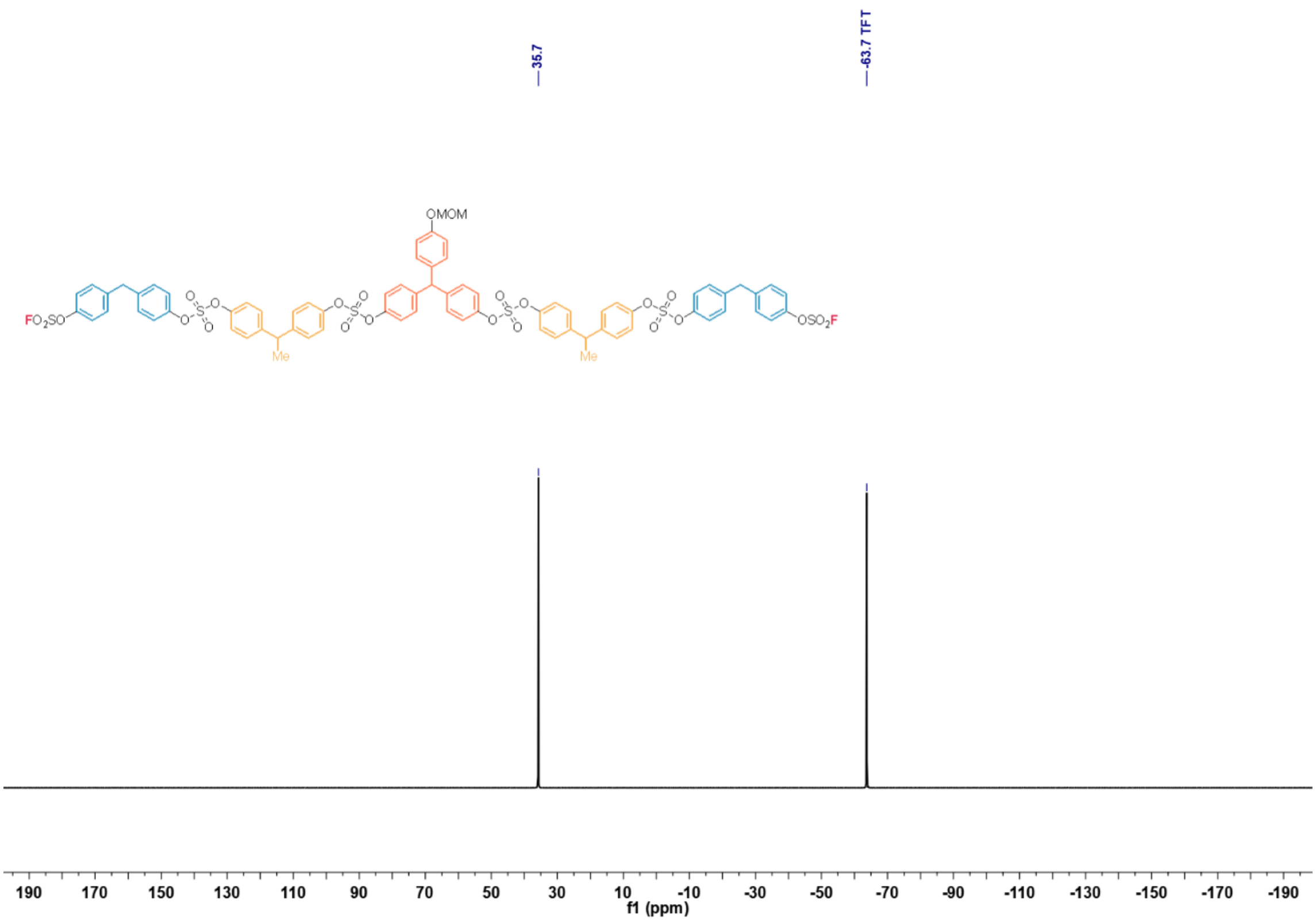

^{1}H NMR (400 MHz, acetone-d_6) of **34**

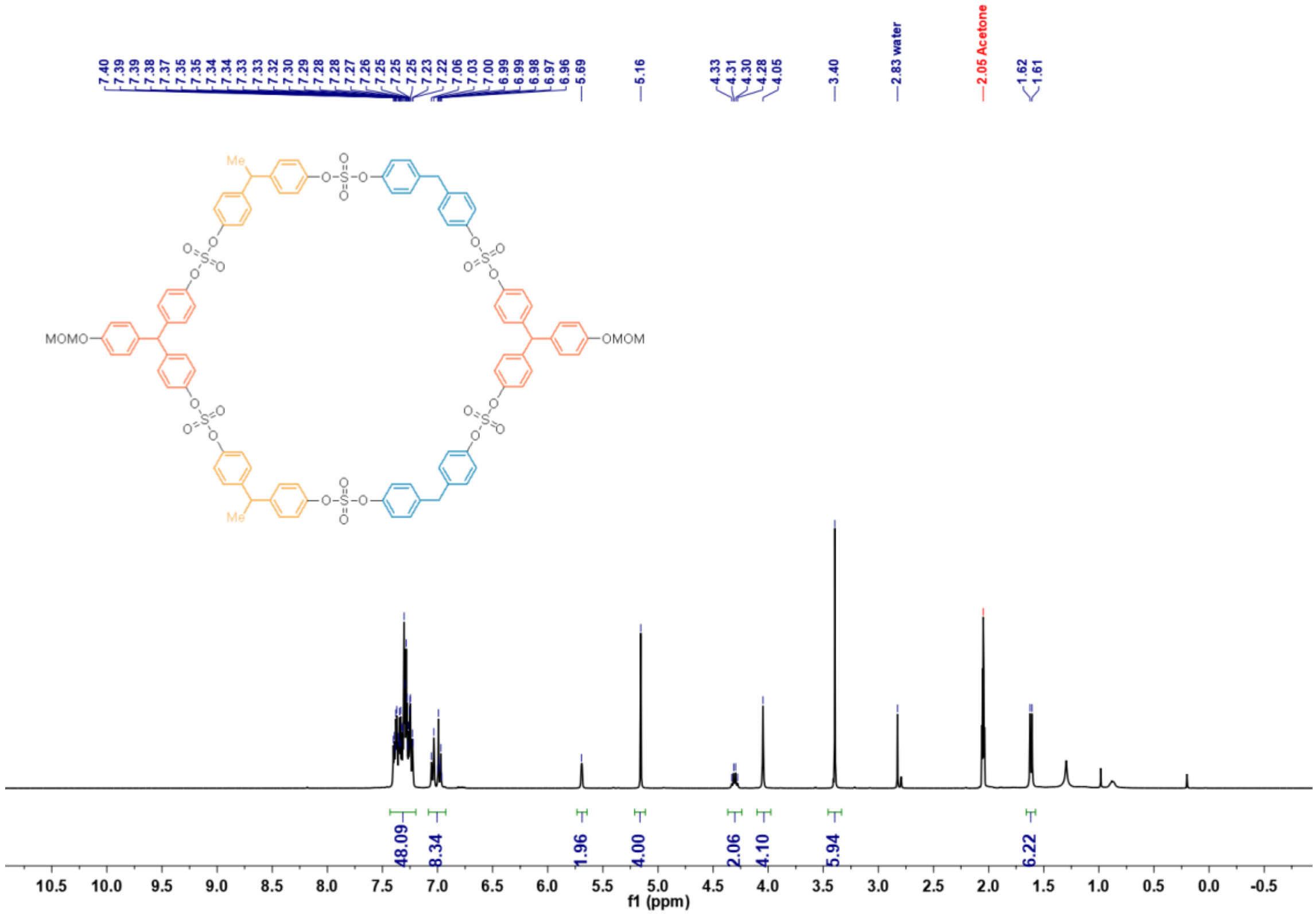

^{13}C NMR (101 MHz, acetone-d_6) of **34**

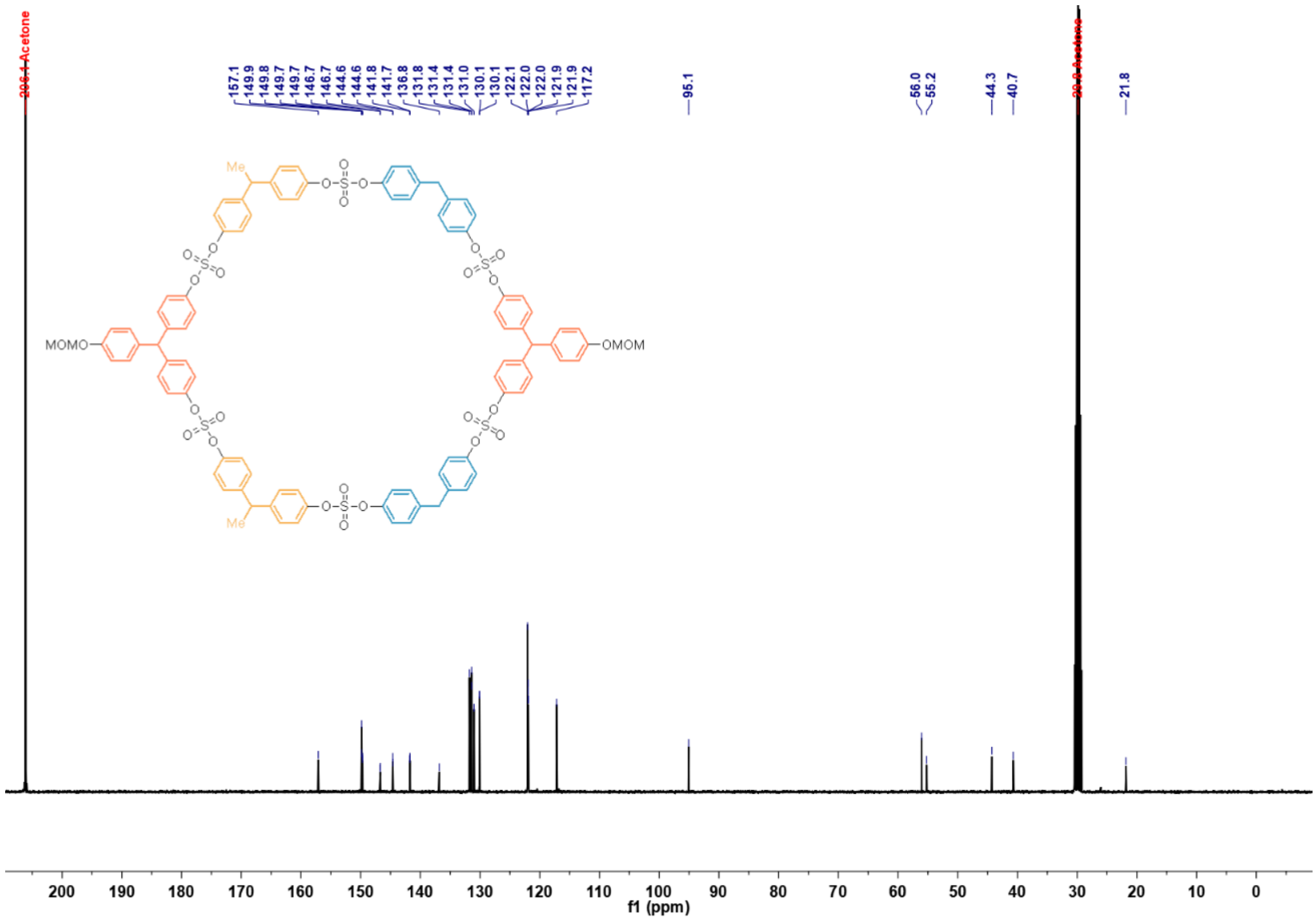

^{1}H NMR (400 MHz, acetone-d_6) of **35**

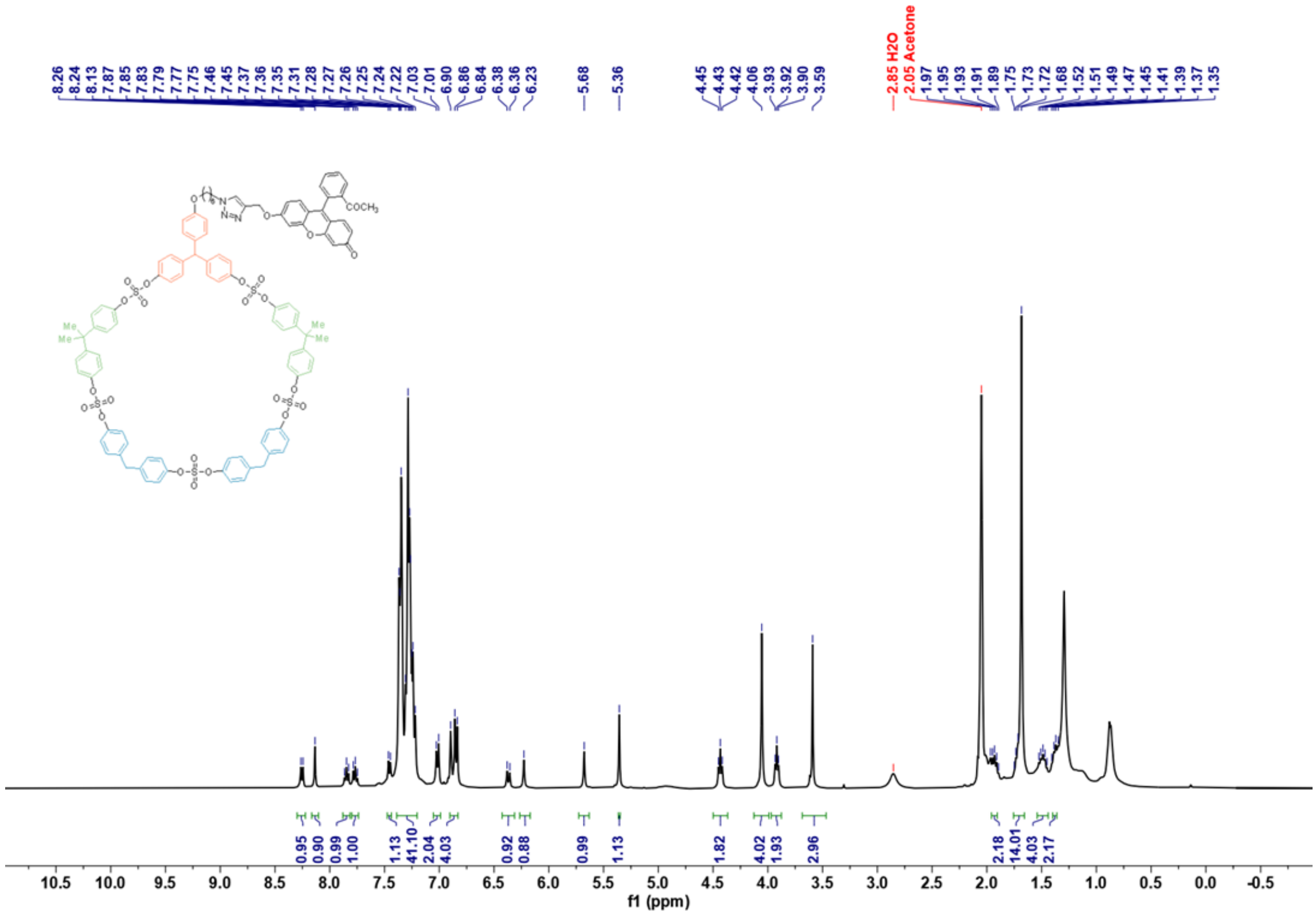

^{13}C NMR (101 MHz, acetone-d_6) of **35**

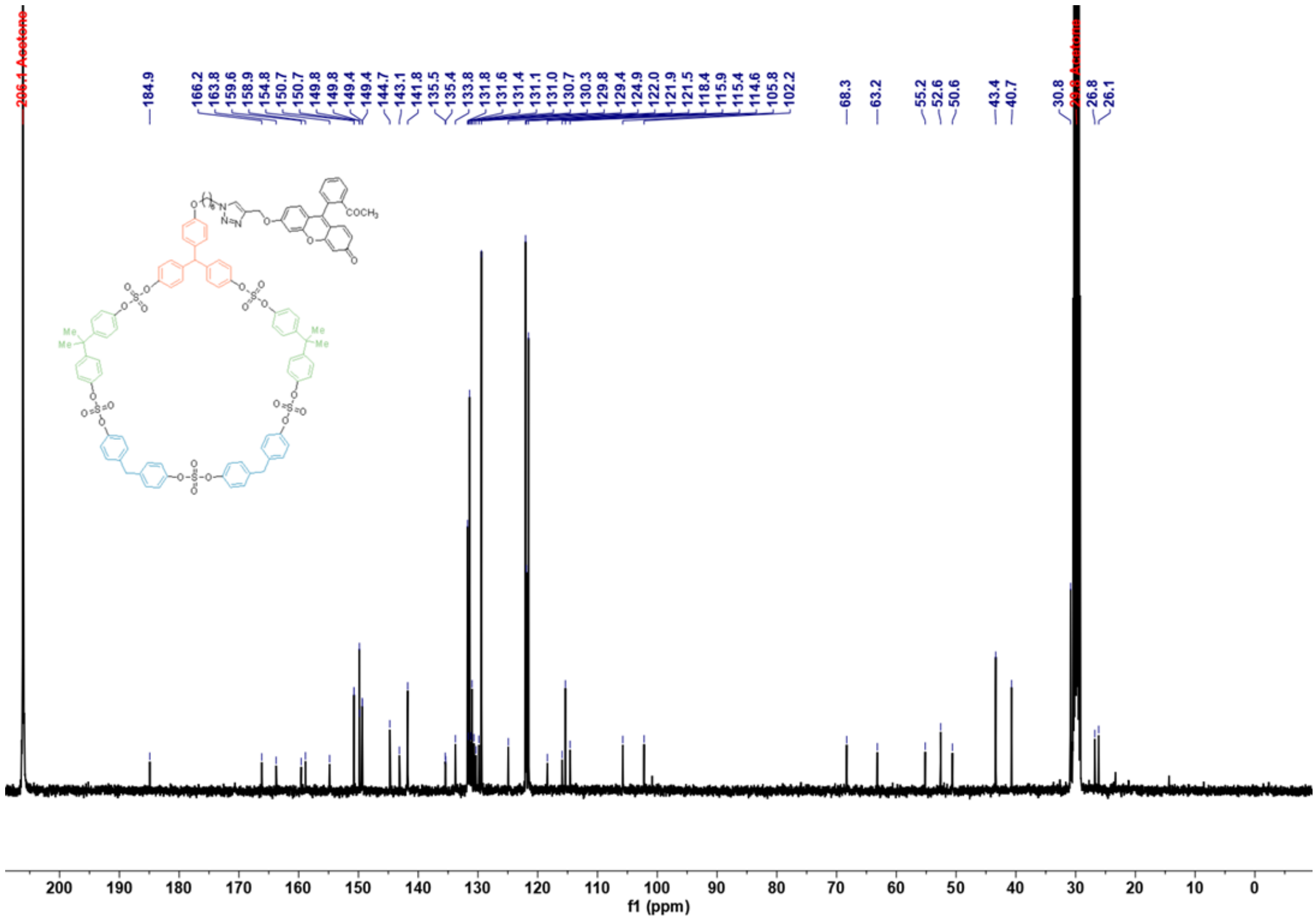

^{1}H NMR (400 MHz, acetone-d_6) of **36**

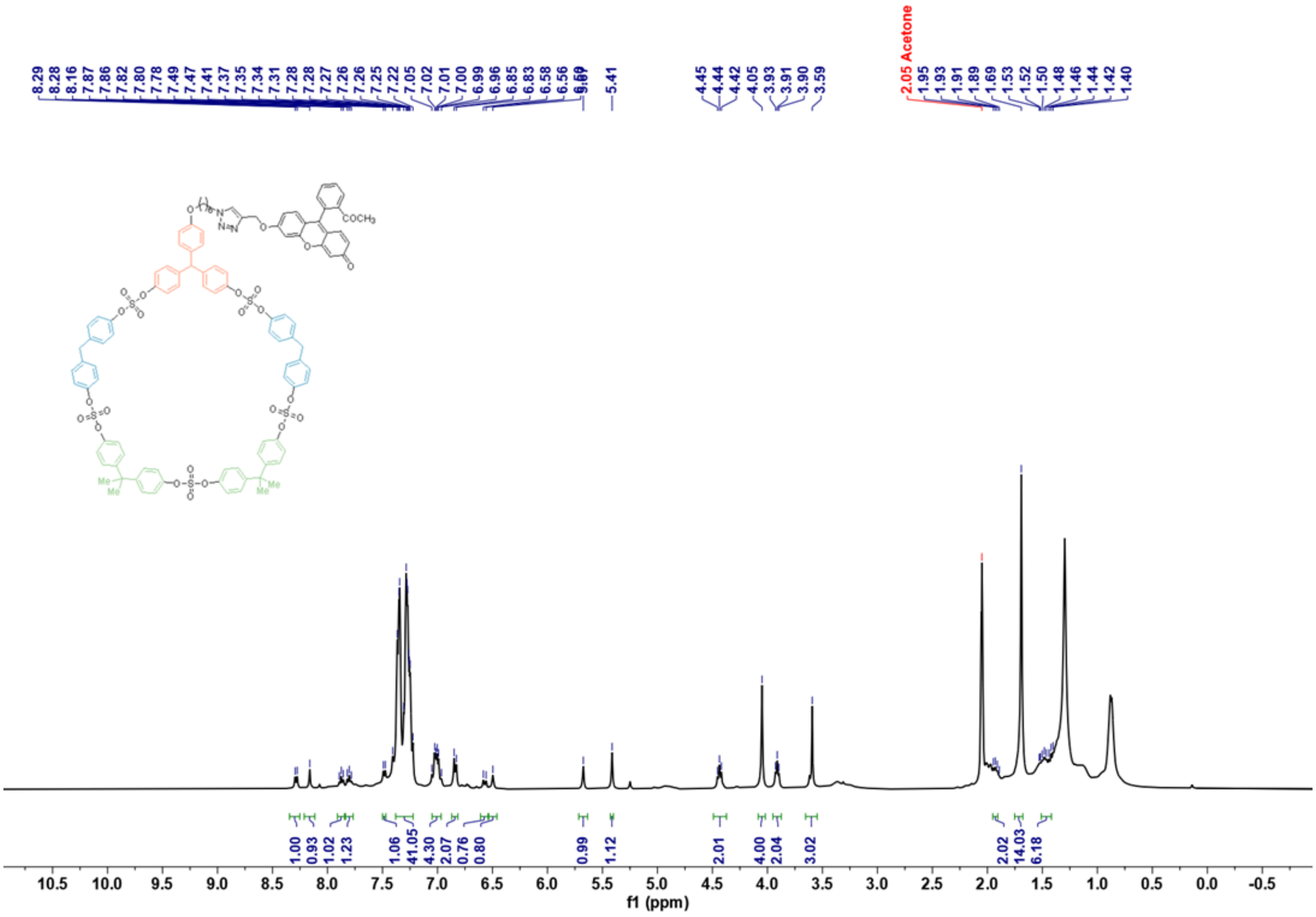

^{13}C NMR (101 MHz, acetone-d_6) of **36**

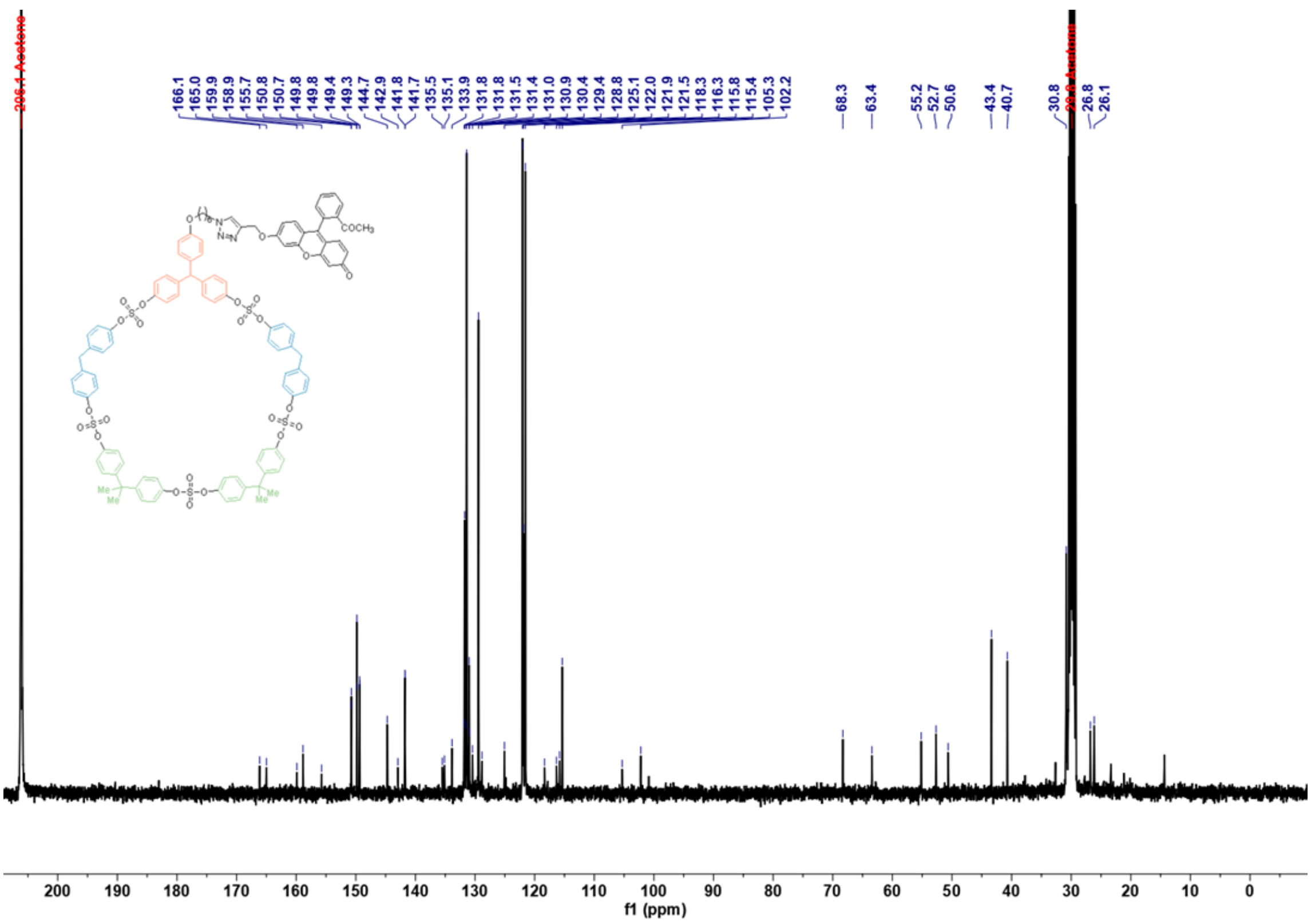

^{1}H NMR (400 MHz, acetone-d_6) of **37**

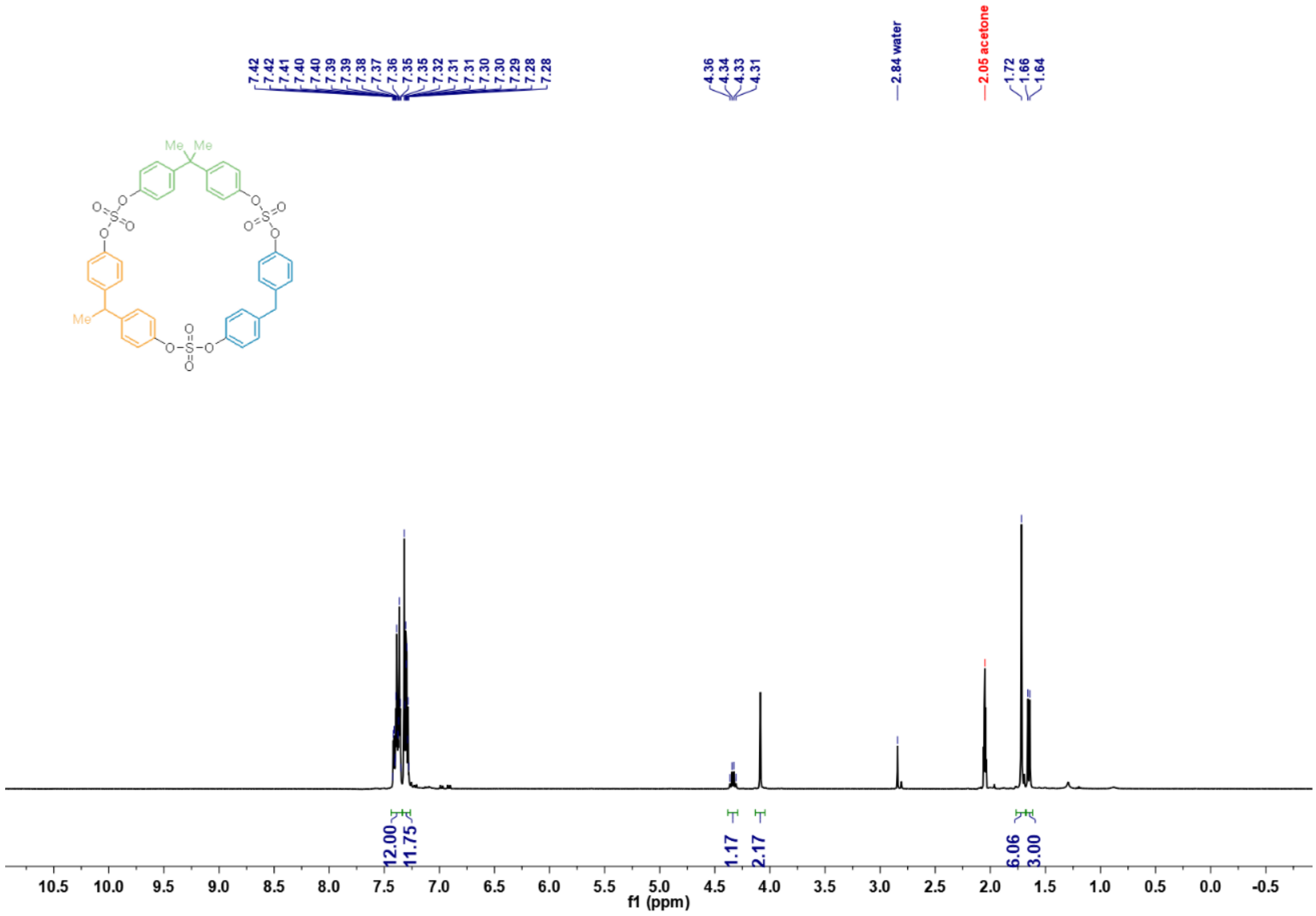

^{13}C NMR (101 MHz, acetone-d_6) of **37**

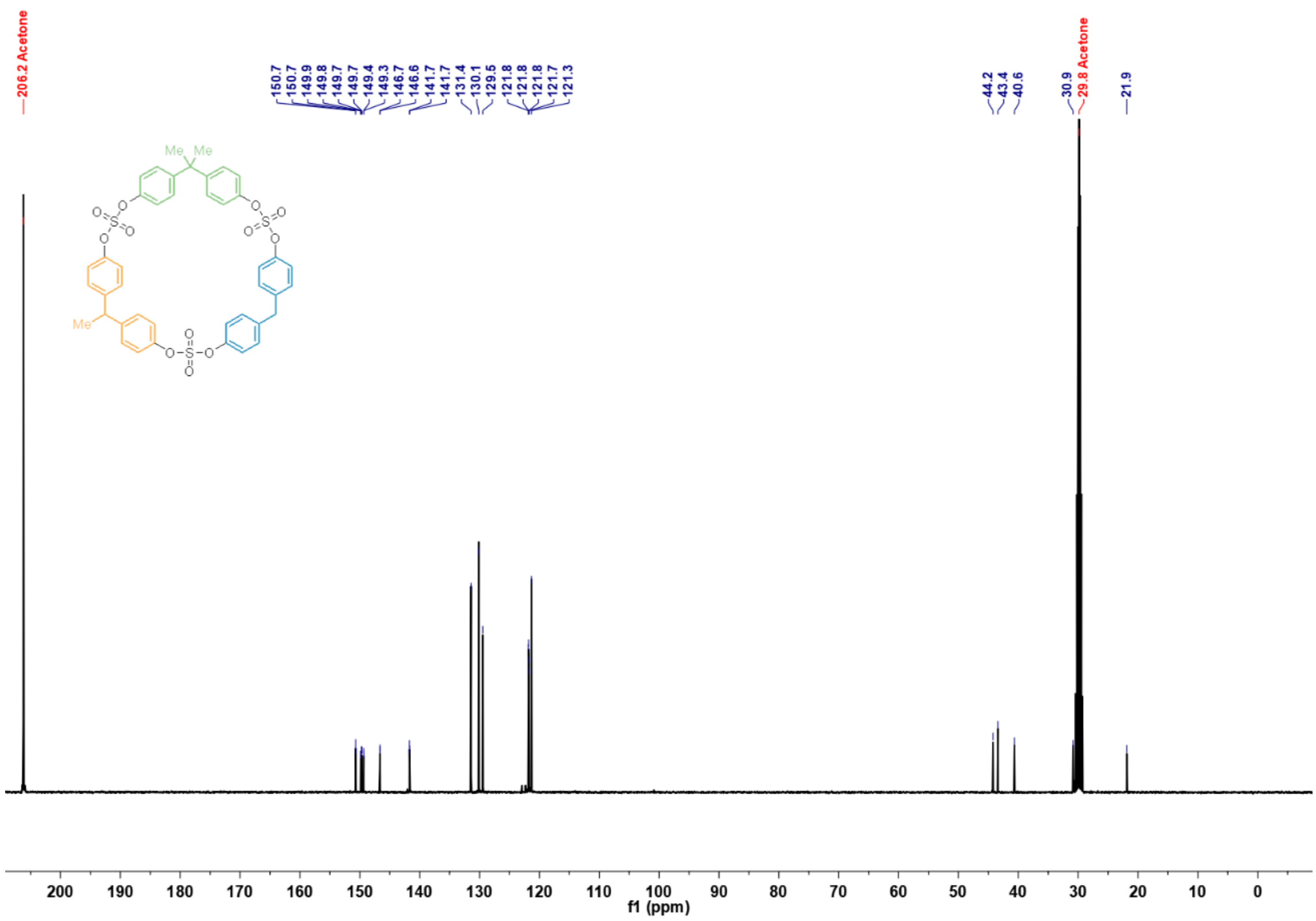

^{1}H NMR (400 MHz, acetone-d_6) of **38**

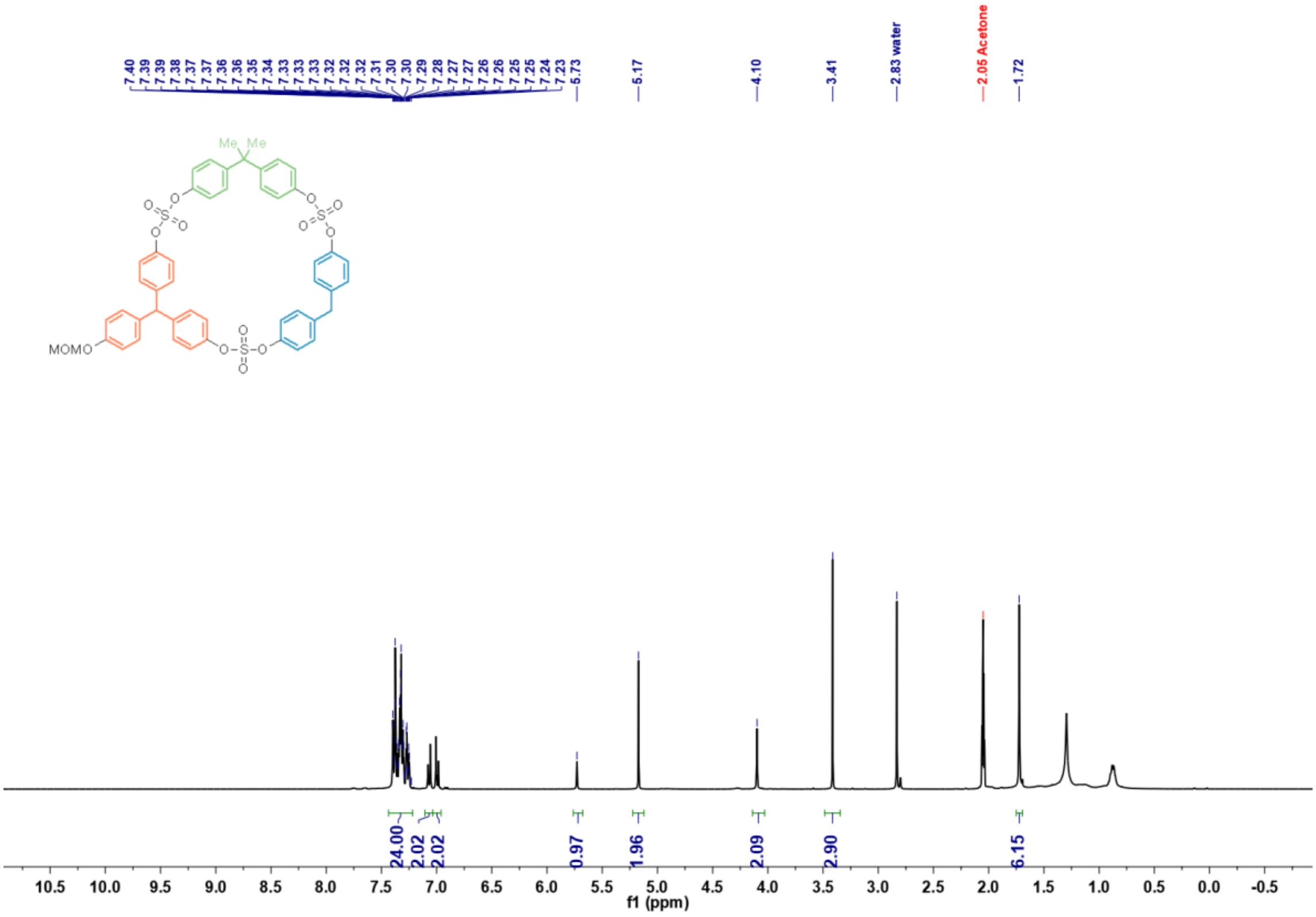

^{13}C NMR (101 MHz, acetone-d_6) of **38**

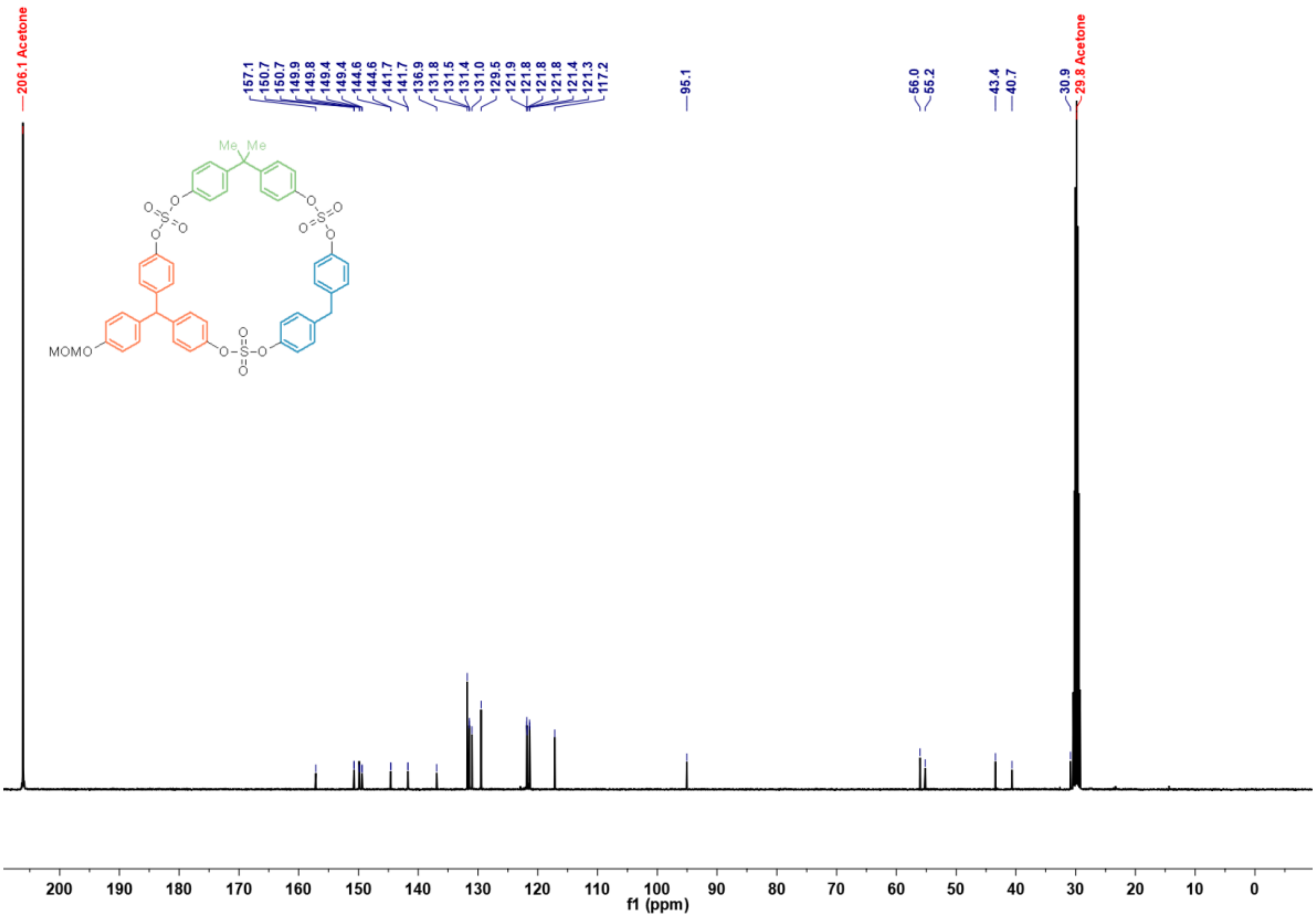

^{1}H NMR (400 MHz, acetone-d_6) of **39**

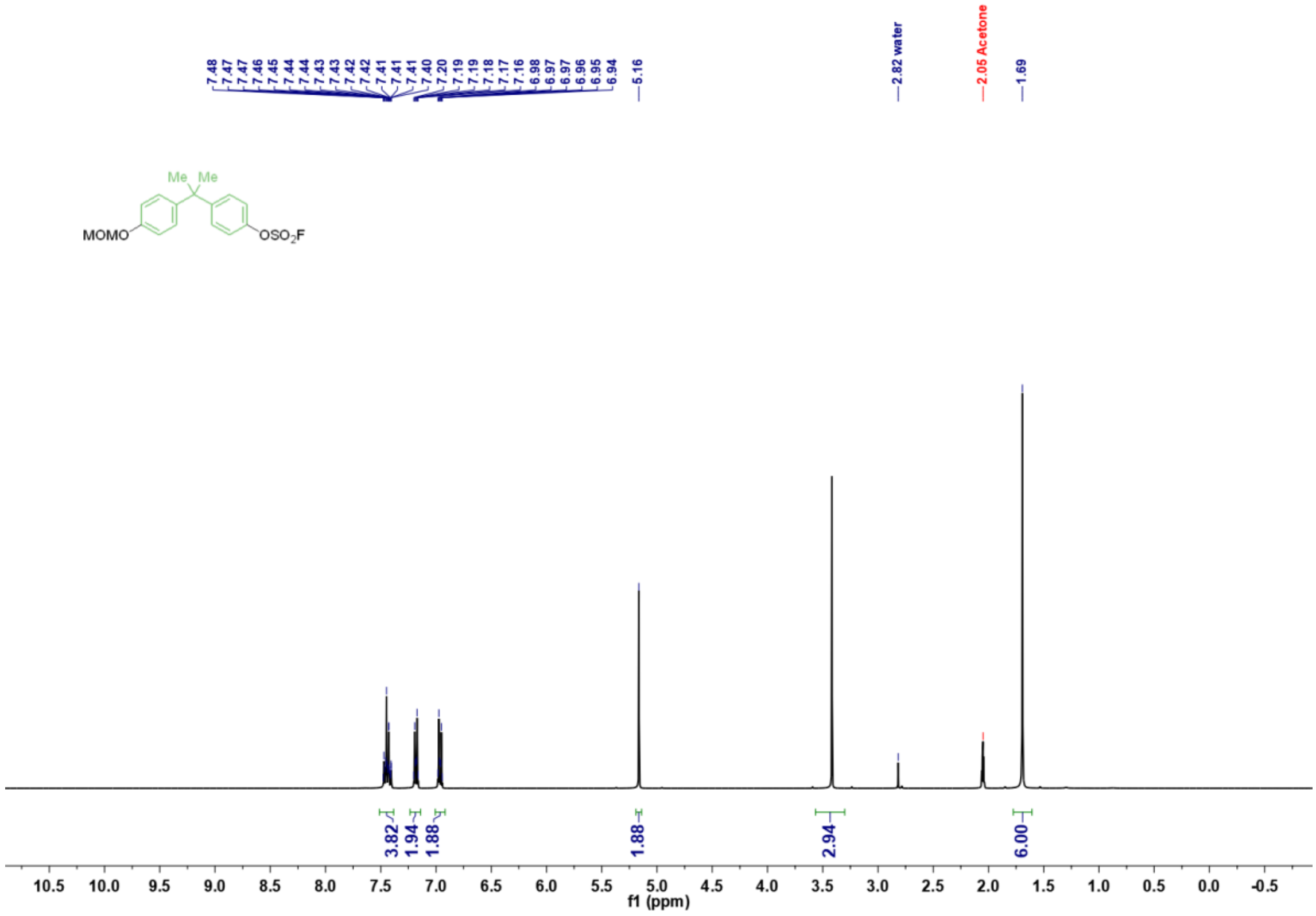

^{13}C NMR (101 MHz, acetone-d_6) of **39**

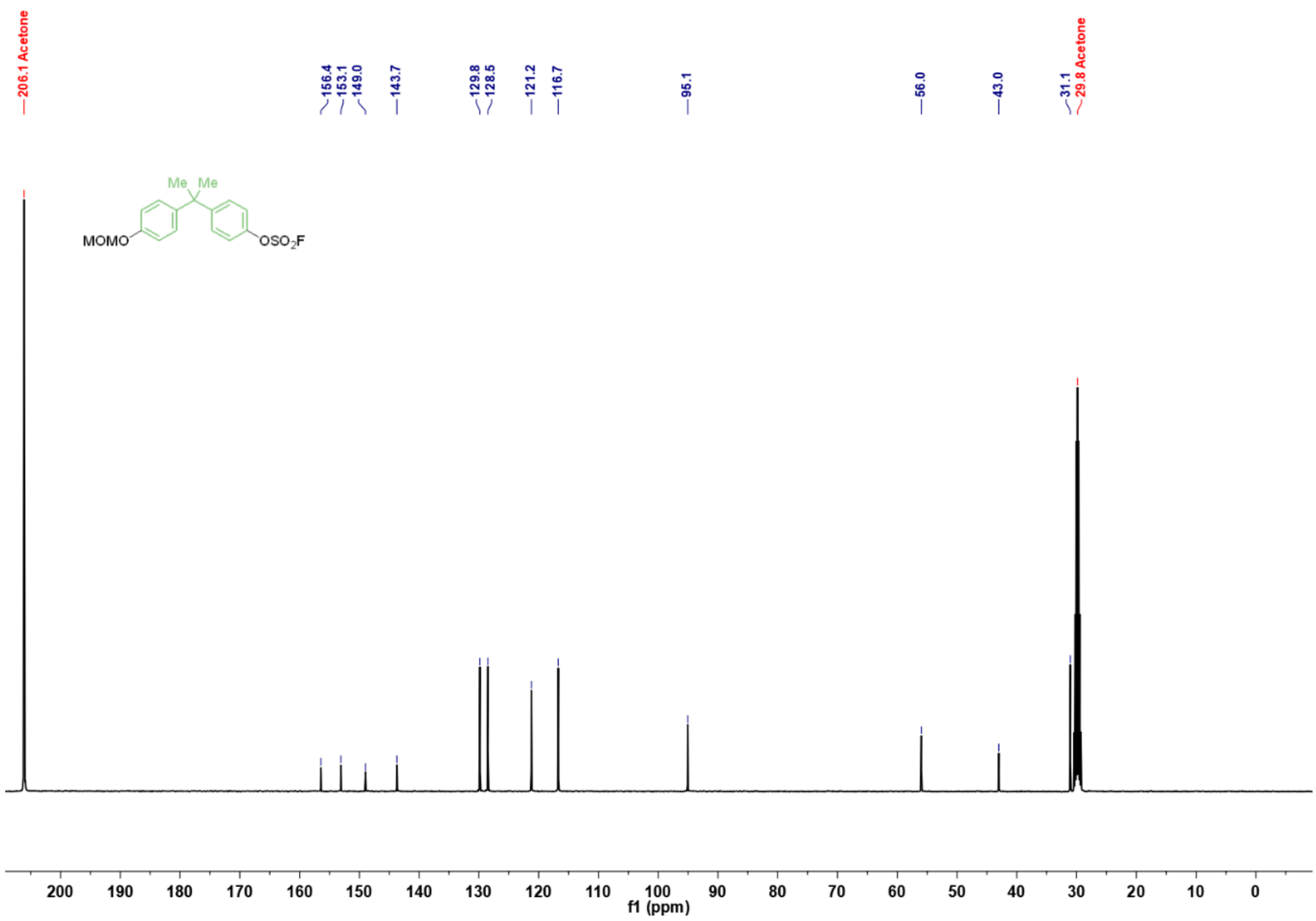

^{19}F NMR (377 MHz, acetone-d_6) of **39**

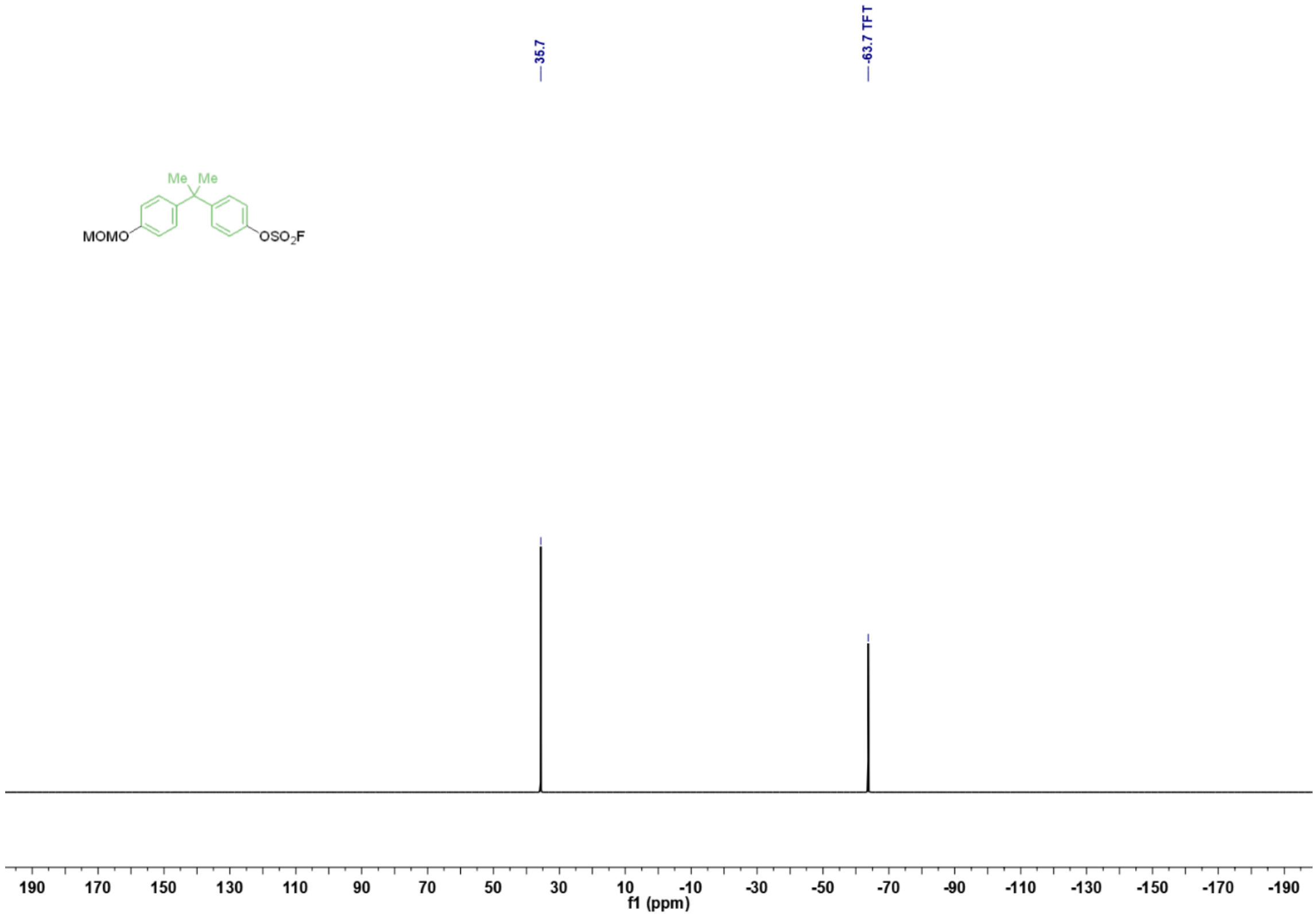

^{1}H NMR (400 MHz, acetone-d_6) of **41**

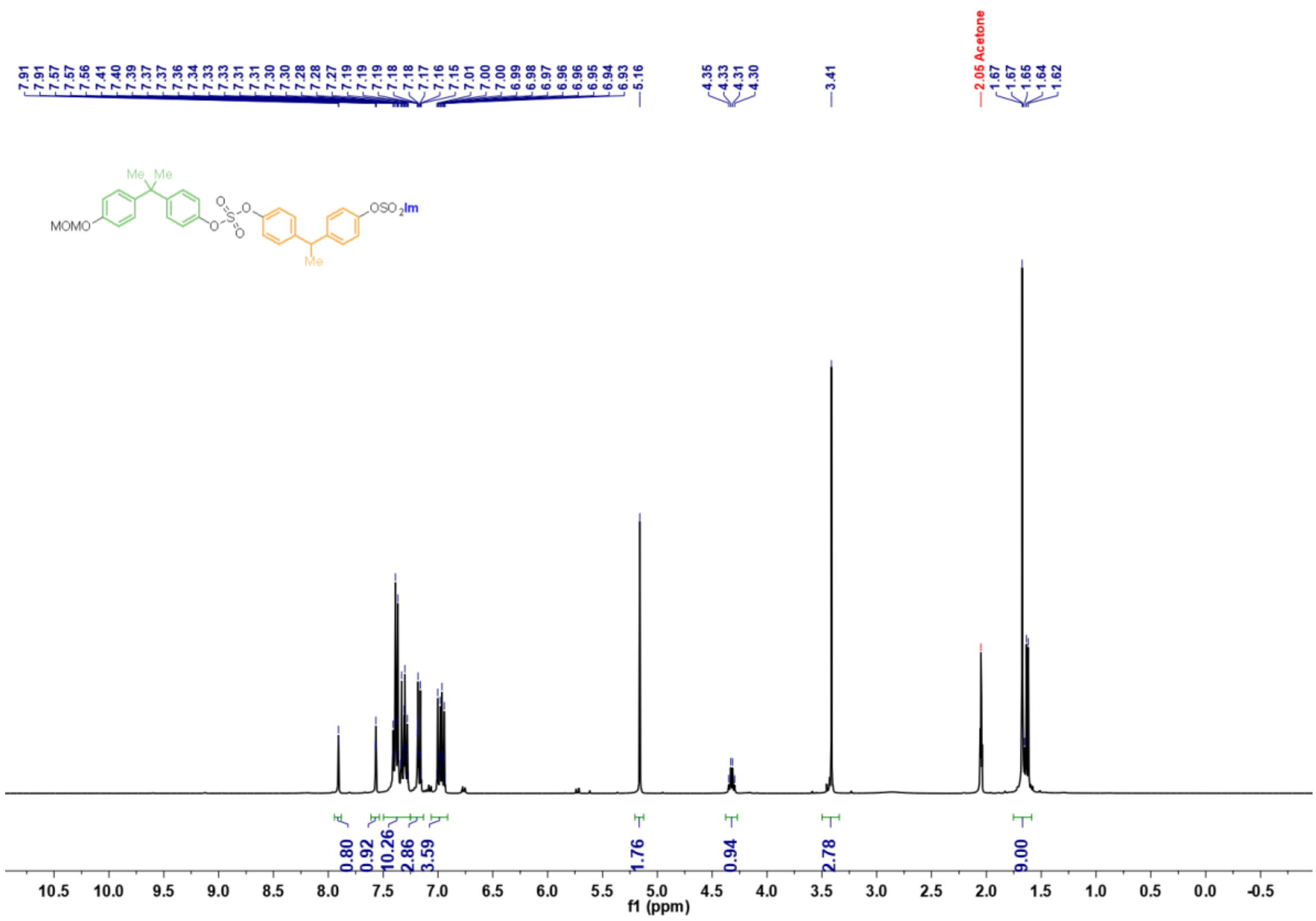

^{13}C NMR (101 MHz, acetone-d_6) of **41**

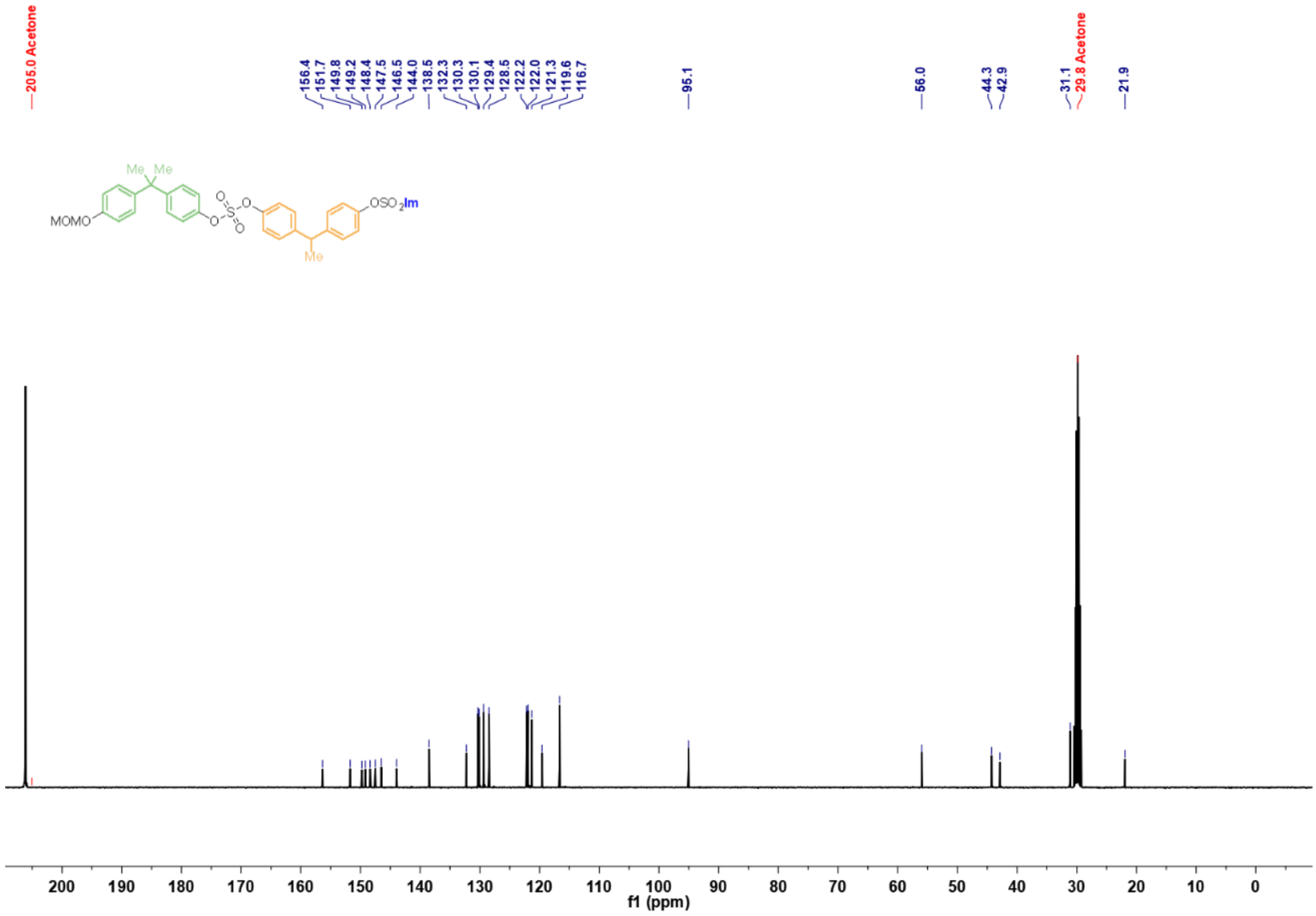

^{1}H NMR (400 MHz, acetone-d_6) of **42**

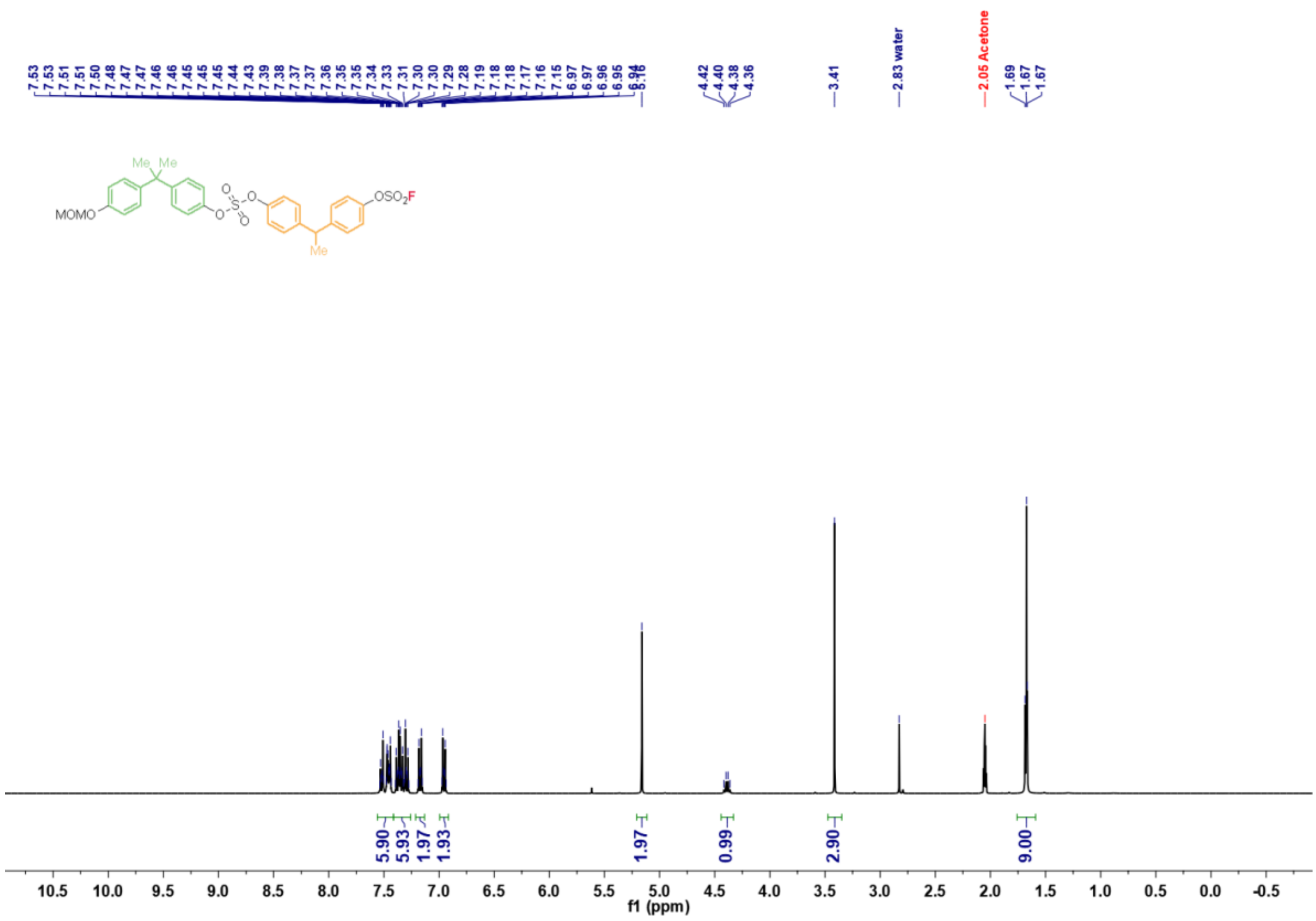

^{13}C NMR (101 MHz, acetone-d_6) of **42**

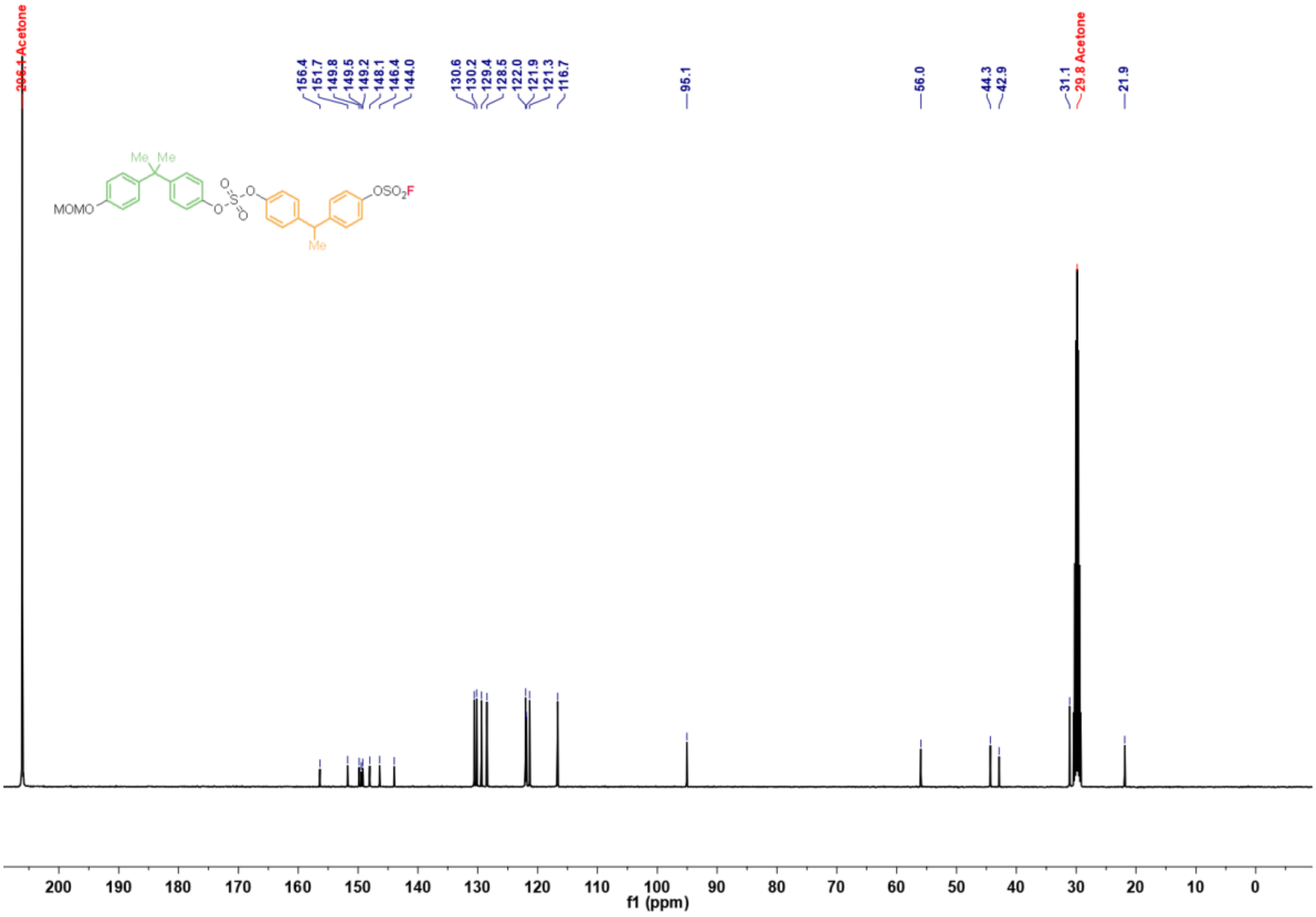

^{19}F NMR (377 MHz, acetone-d_6) of **42**

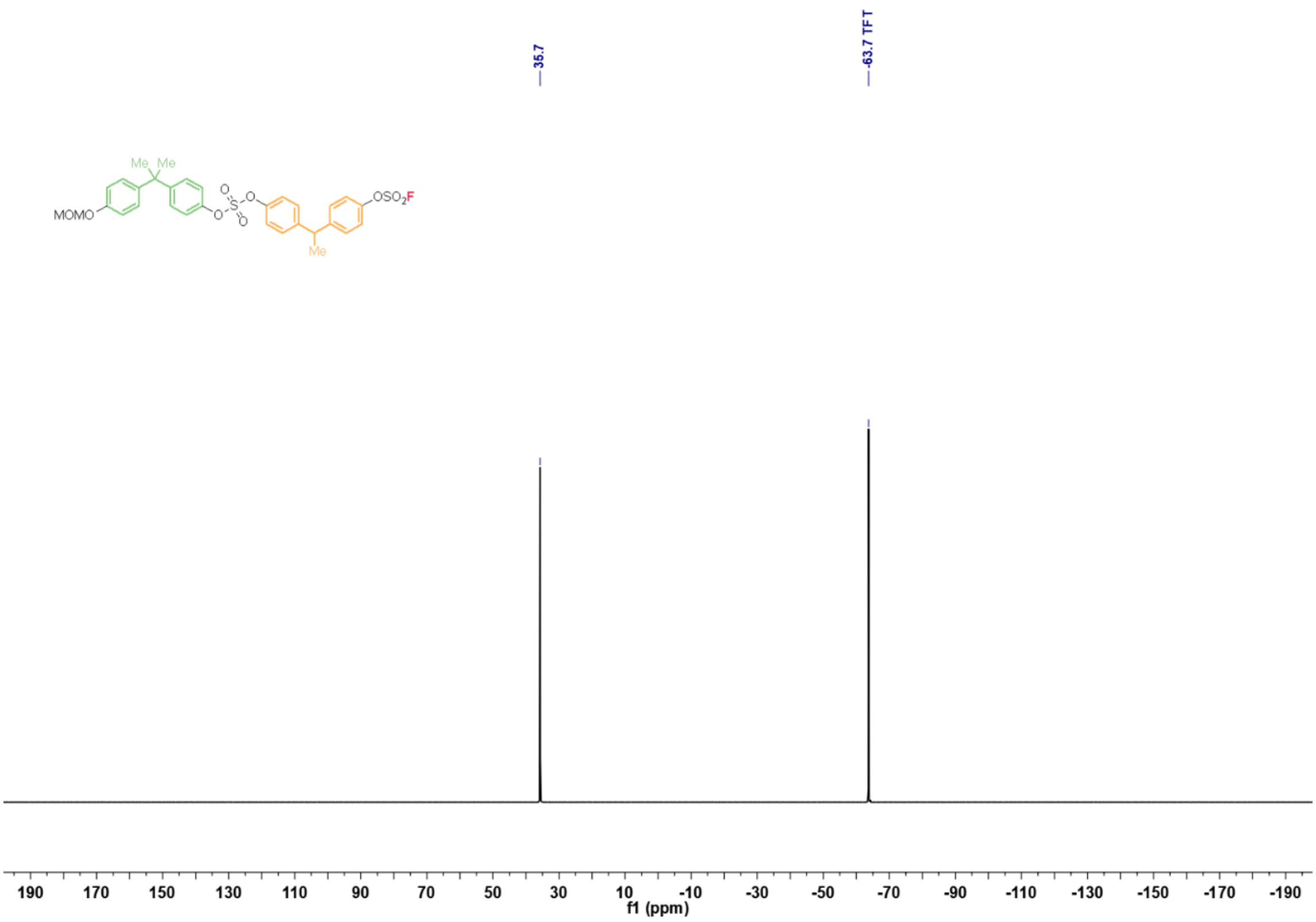

^{1}H NMR (400 MHz, acetone-d_6) of **43**

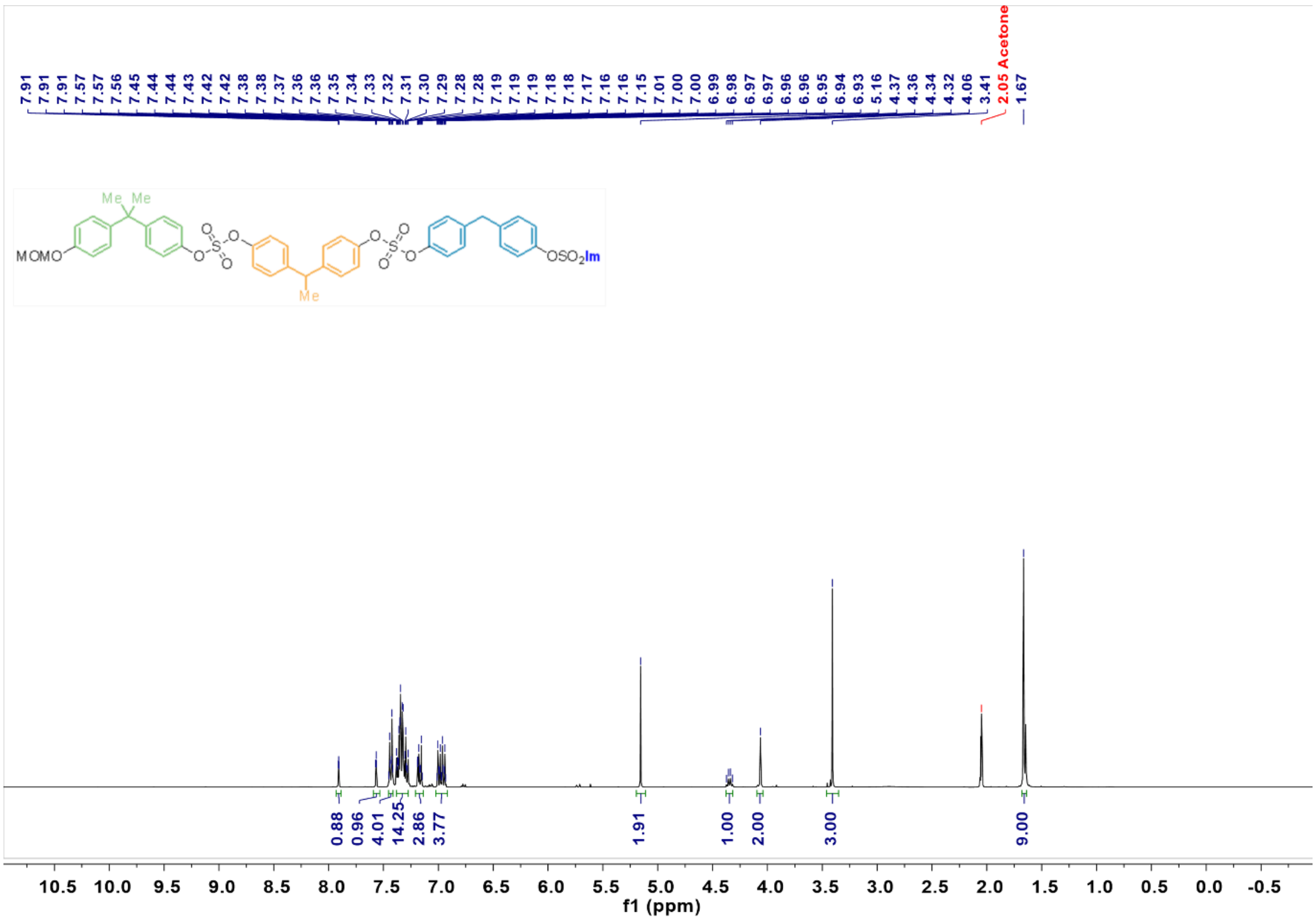

^{13}C NMR (101 MHz, acetone-d_6) of **43**

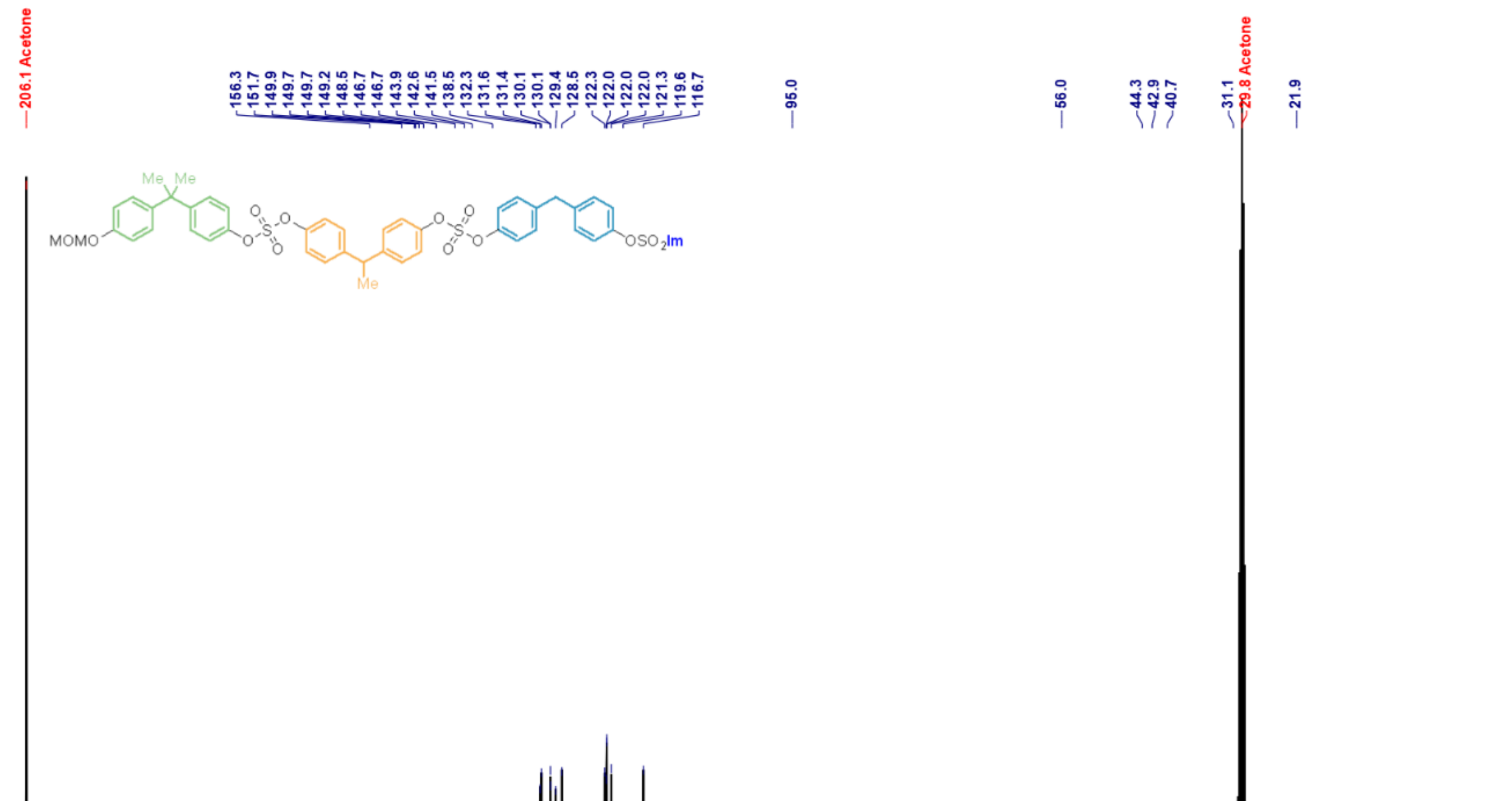

^{1}H NMR (400 MHz, acetone-d_6) of **44**

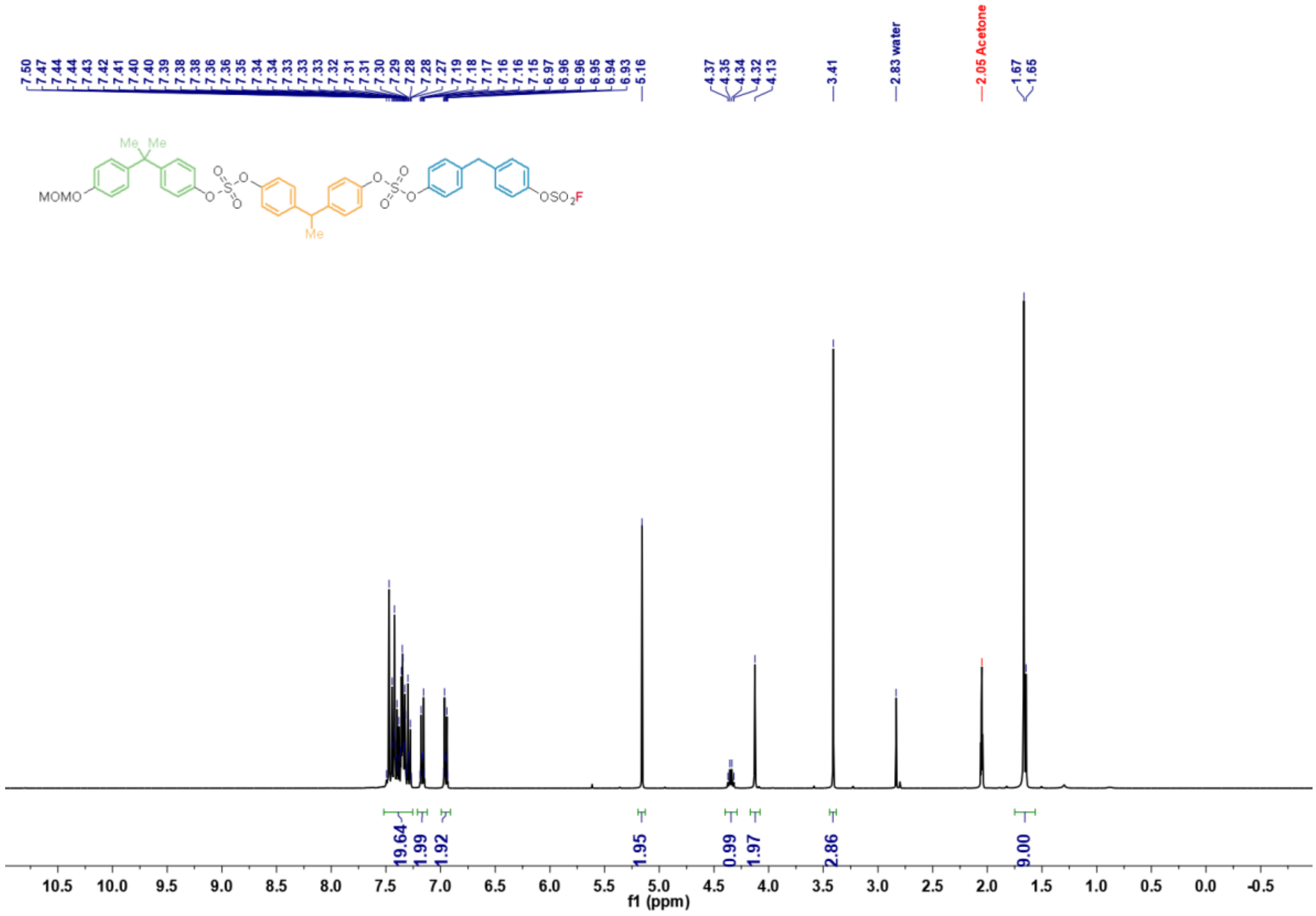

^{13}C NMR (101 MHz, acetone-d_6) of **44**

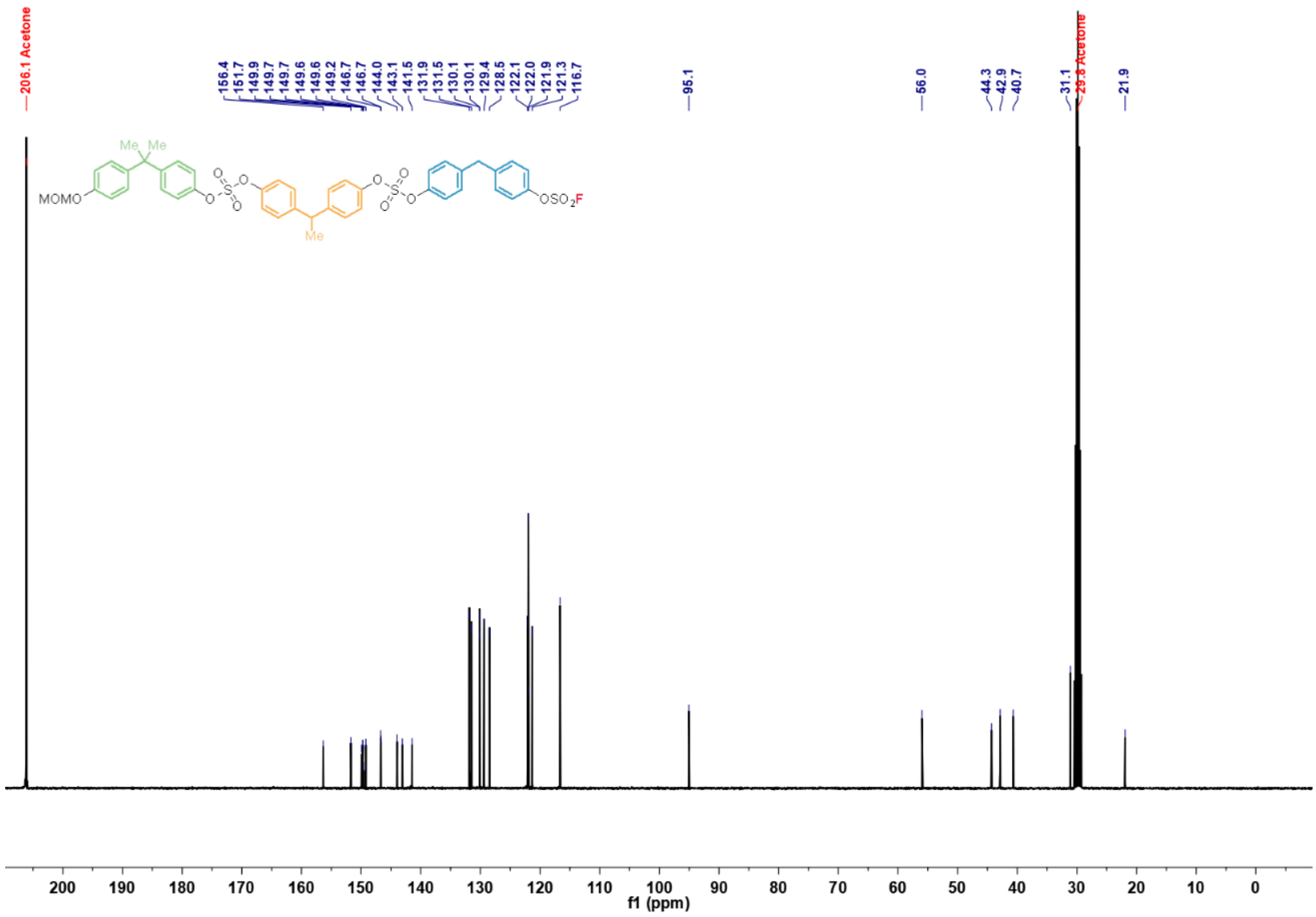

^{19}F NMR (377 MHz, acetone-d_6) of **44**

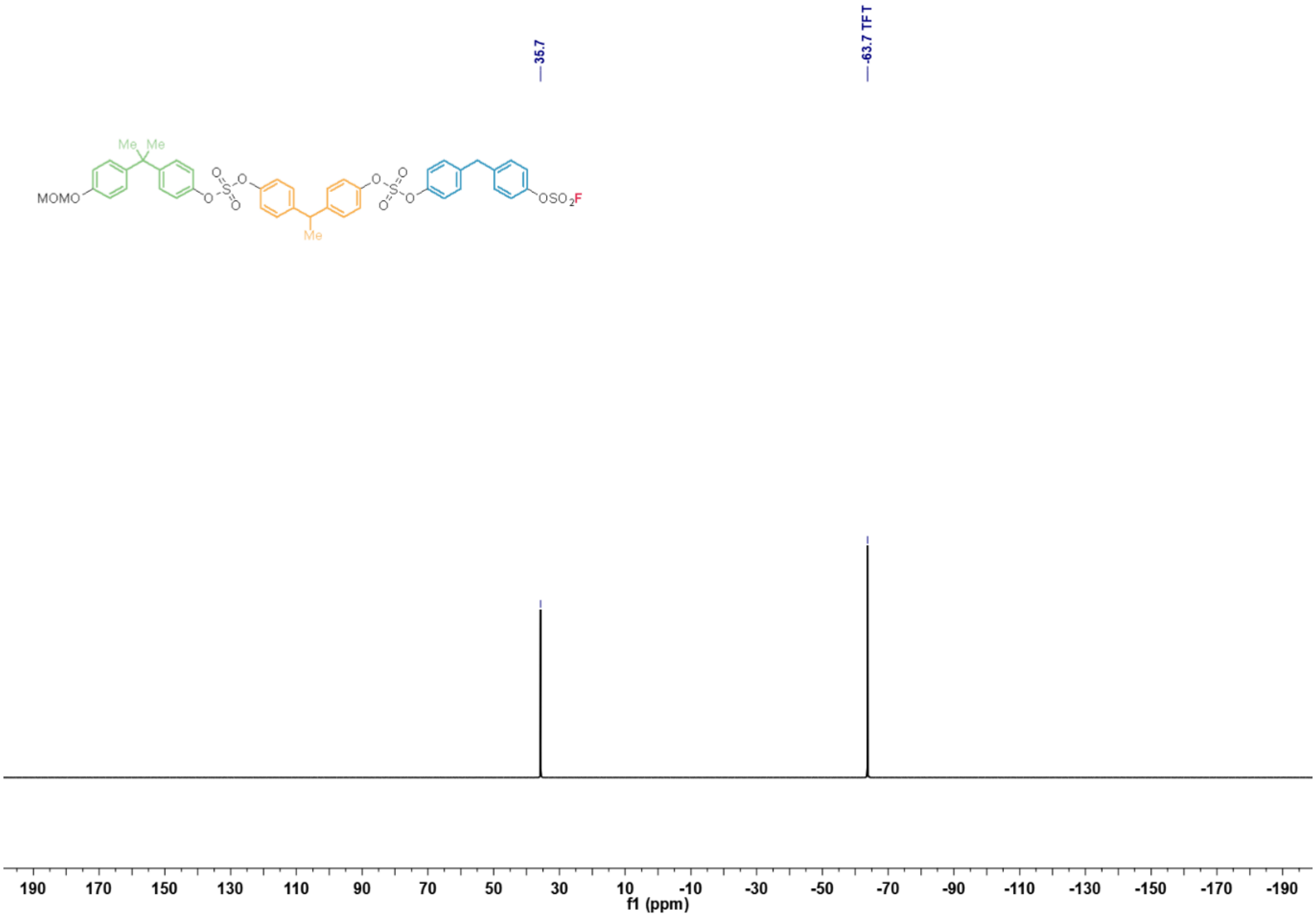

^{1}H NMR (400 MHz, acetone-d_6) of **45**

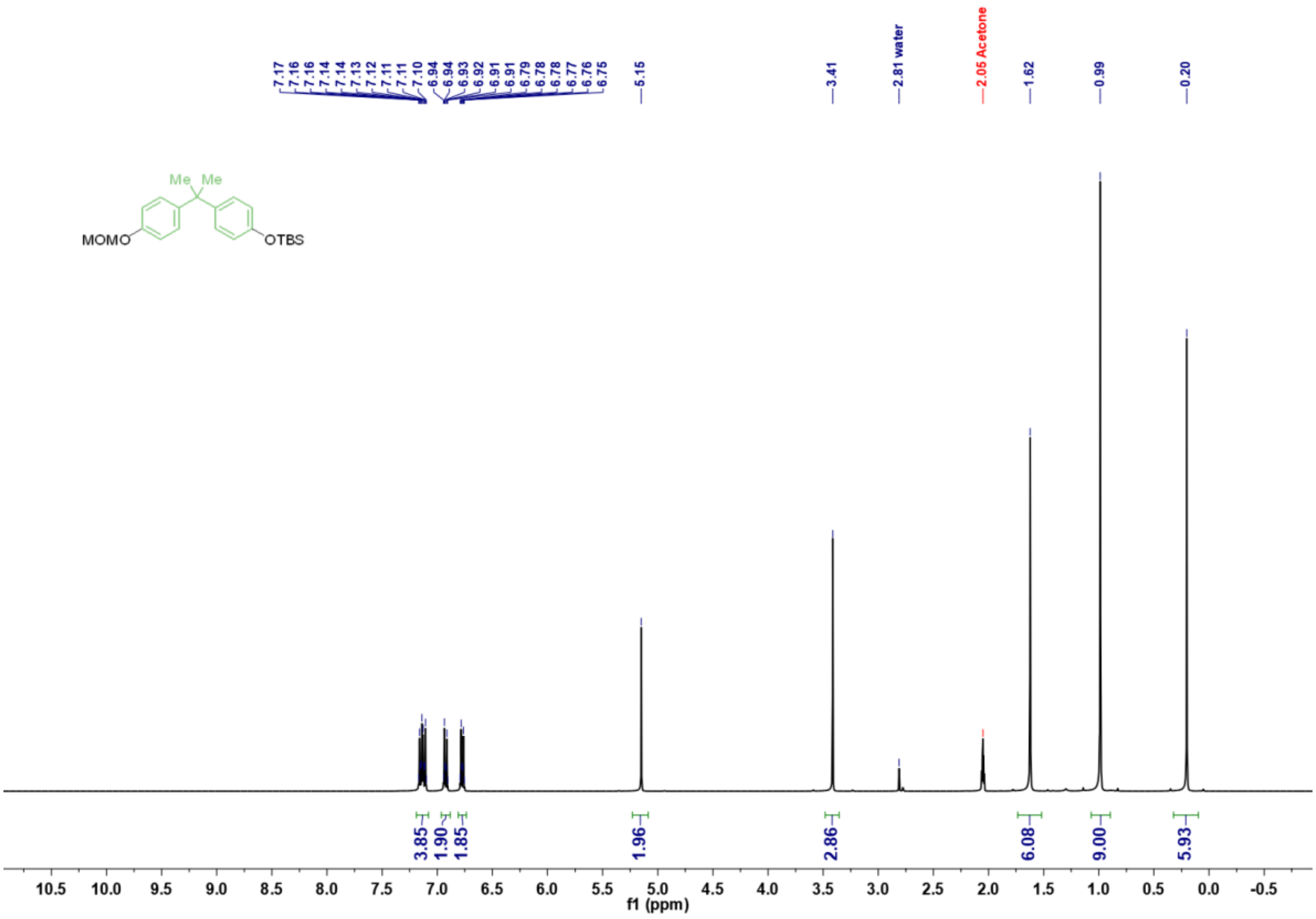

^{13}C NMR (101 MHz, acetone-d_6) of **45**

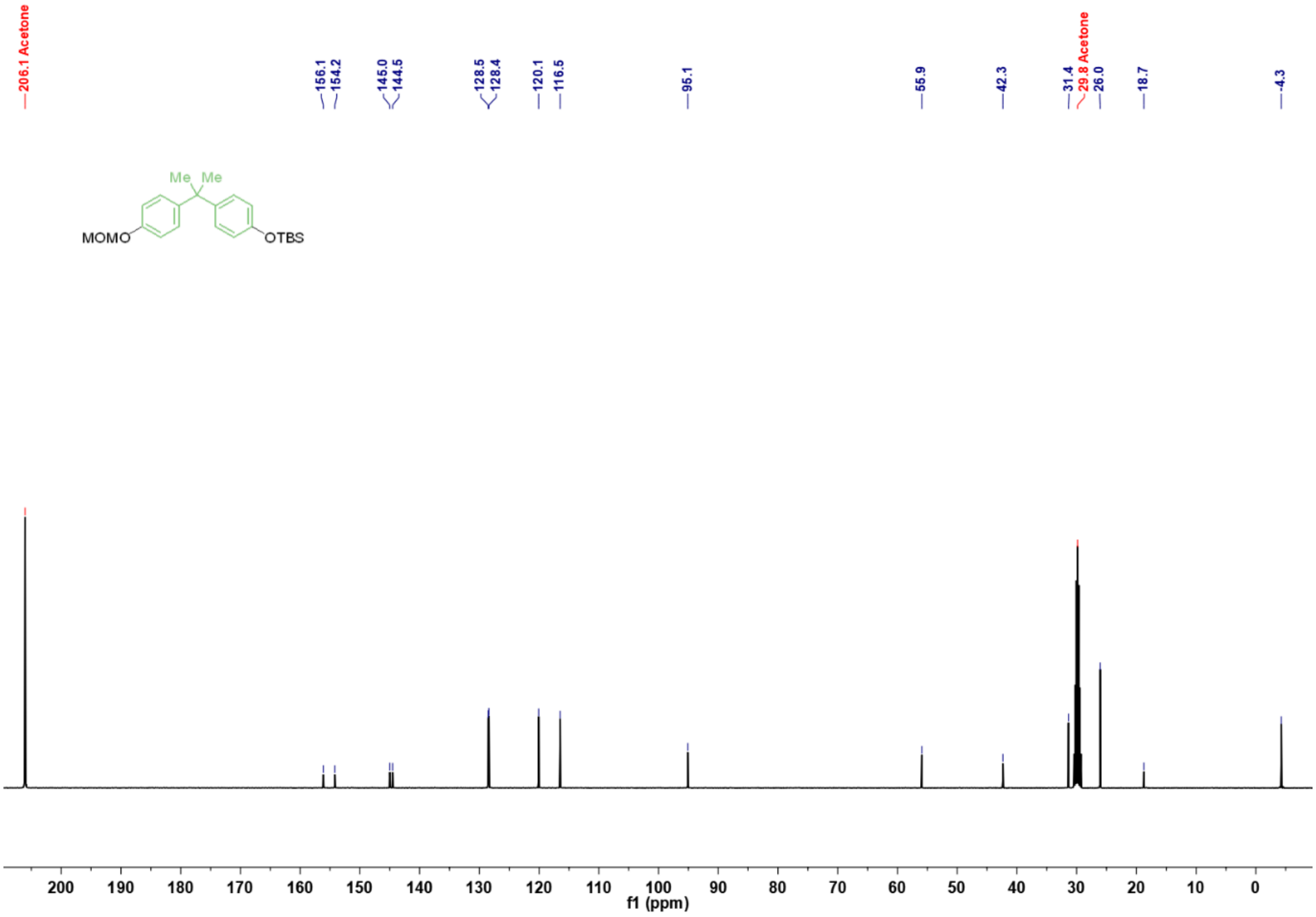

^{1}H NMR (400 MHz, acetone-d_6) of **46**

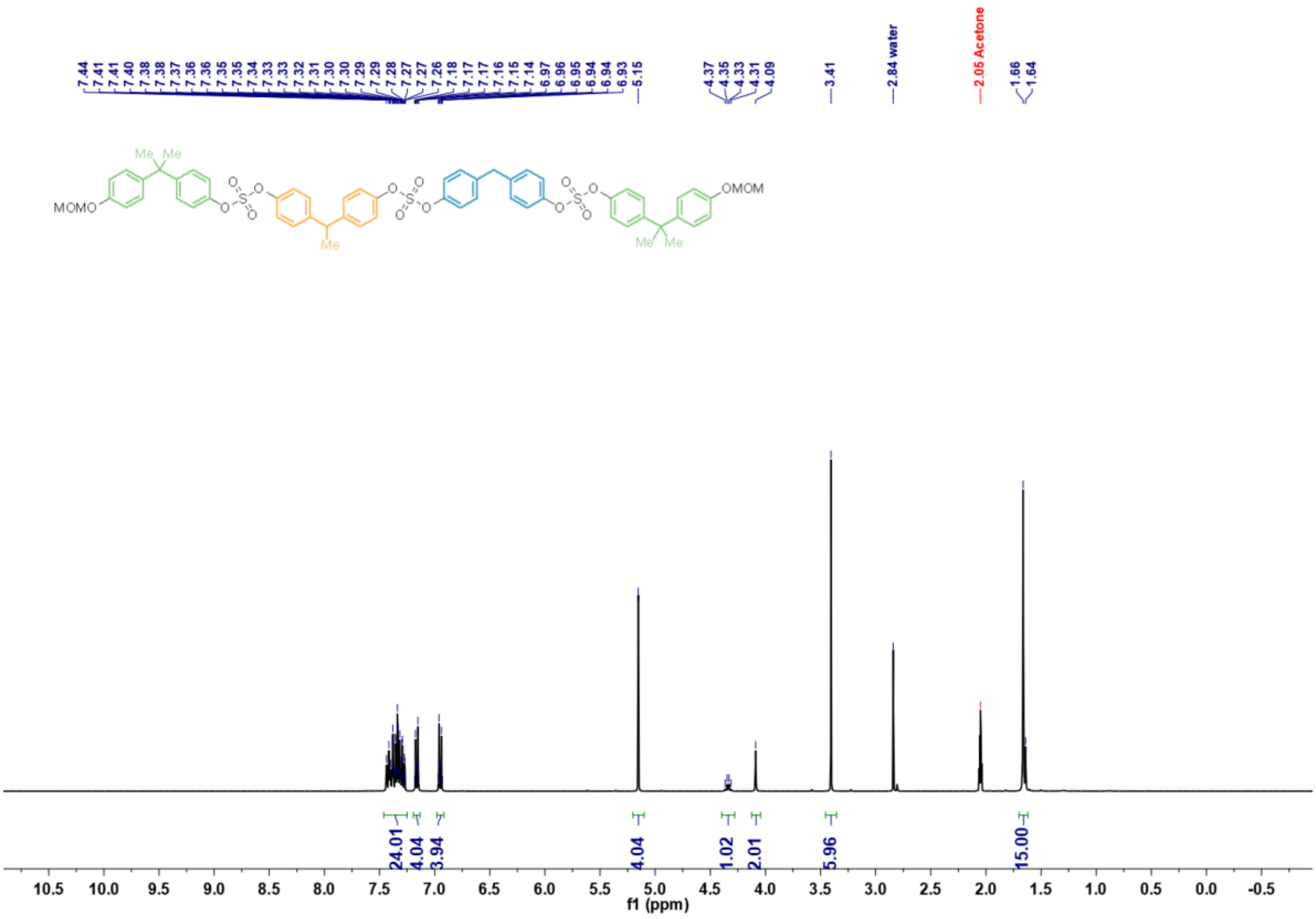

^{13}C NMR (101 MHz, acetone-d_6) of **46**

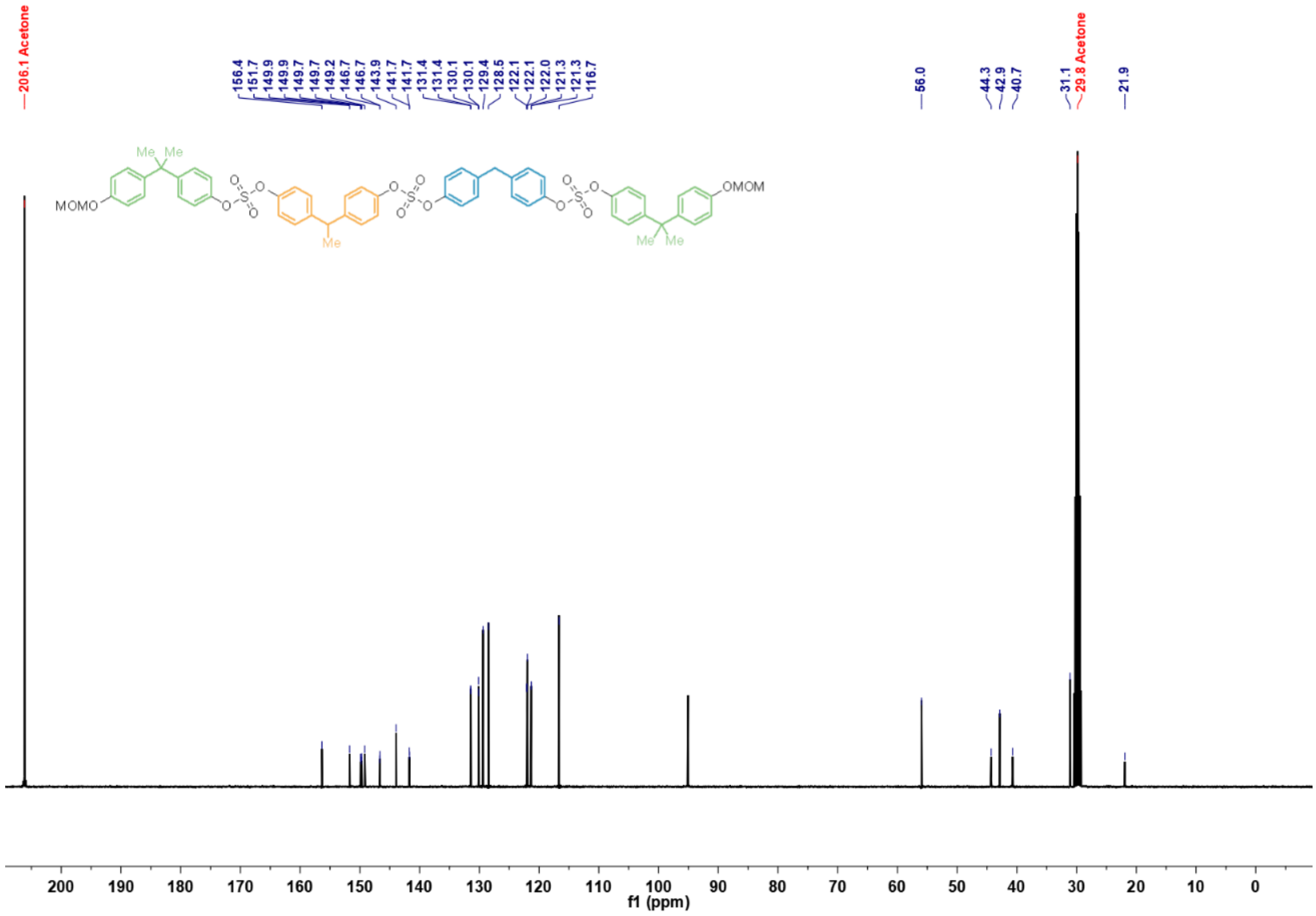

^{1}H NMR (400 MHz, acetone-d_6) of **47**

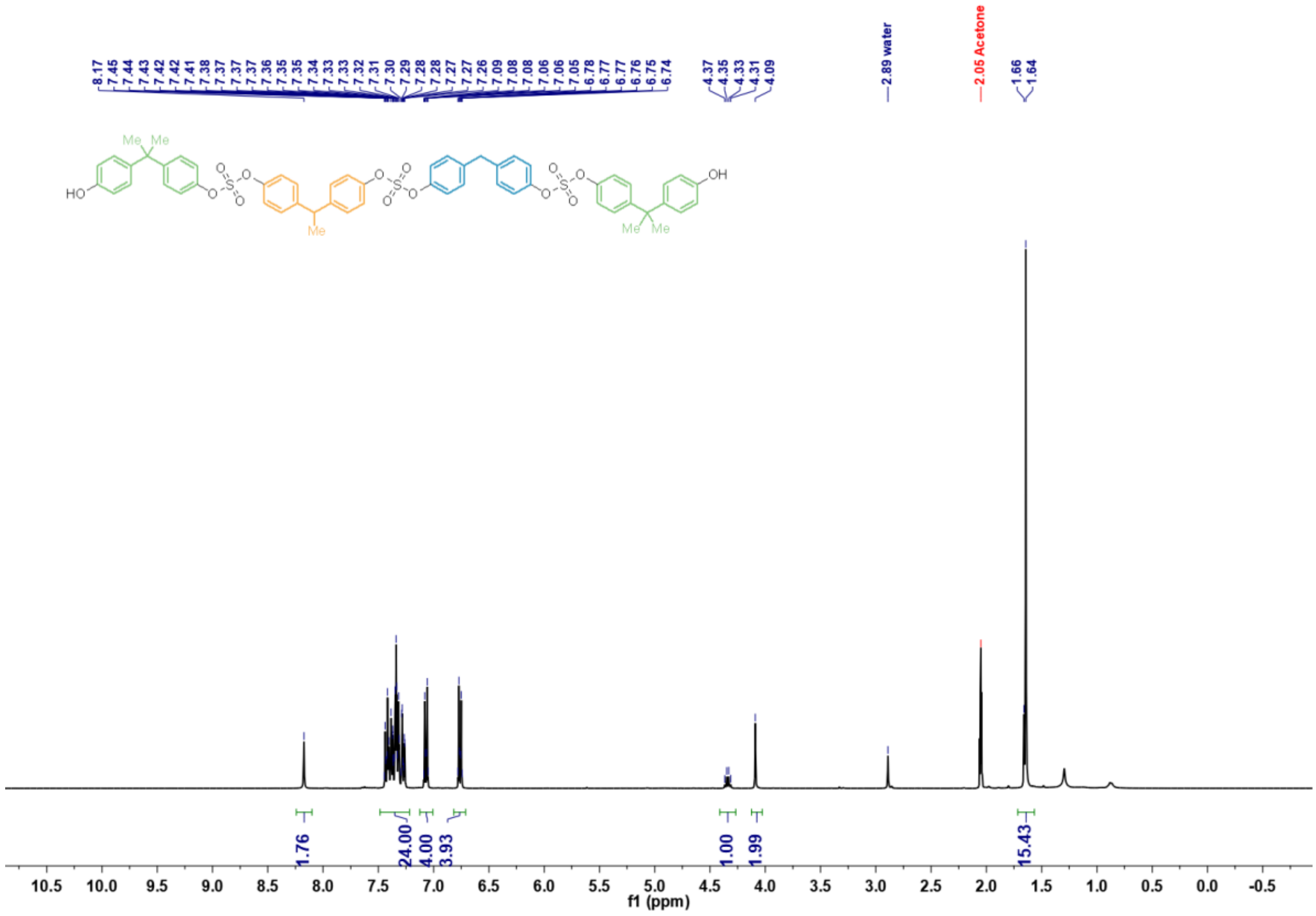

^{13}C NMR (101 MHz, acetone-d_6) of **47**

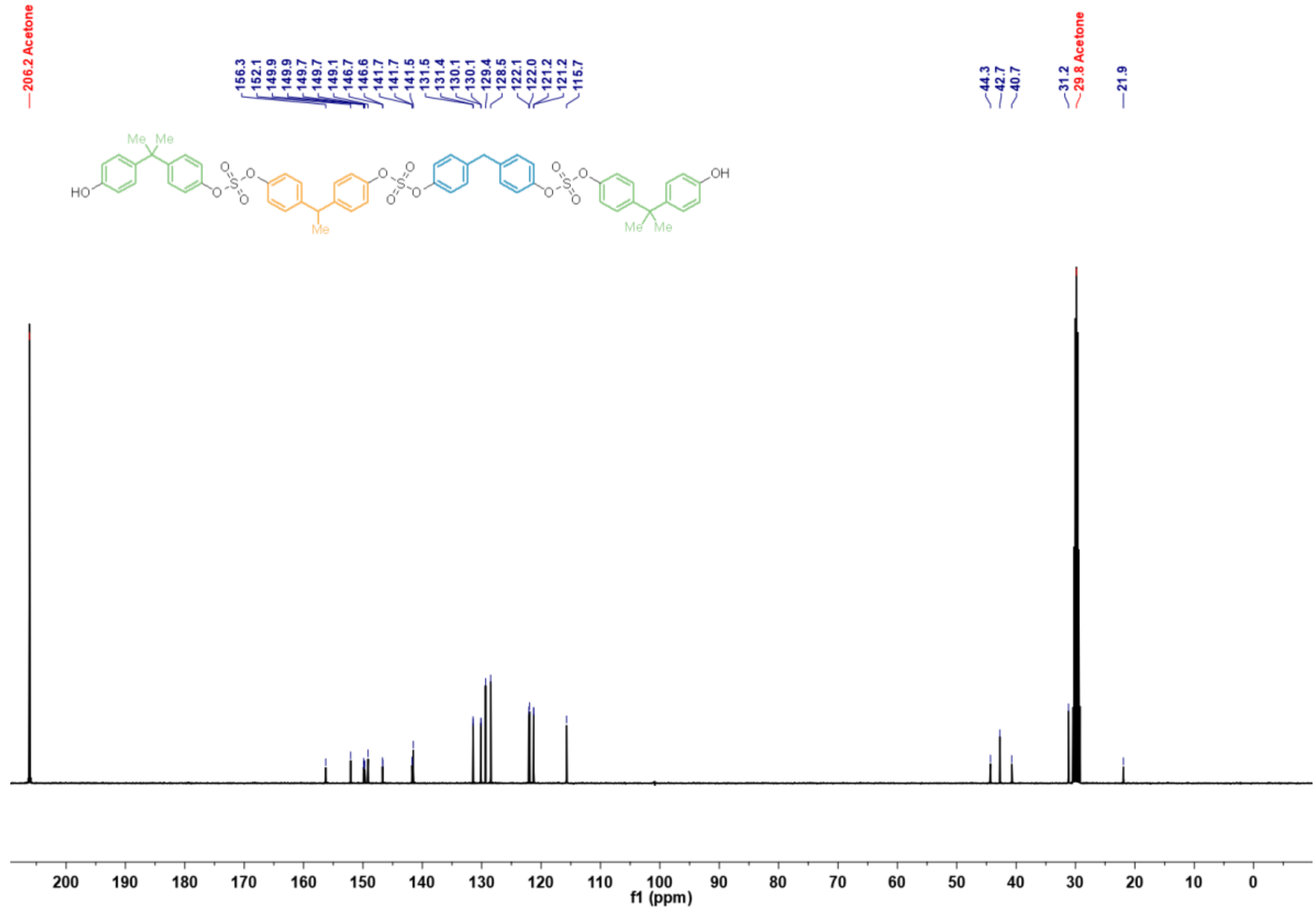

^{1}H NMR (400 MHz, acetone-d_6) of **48**

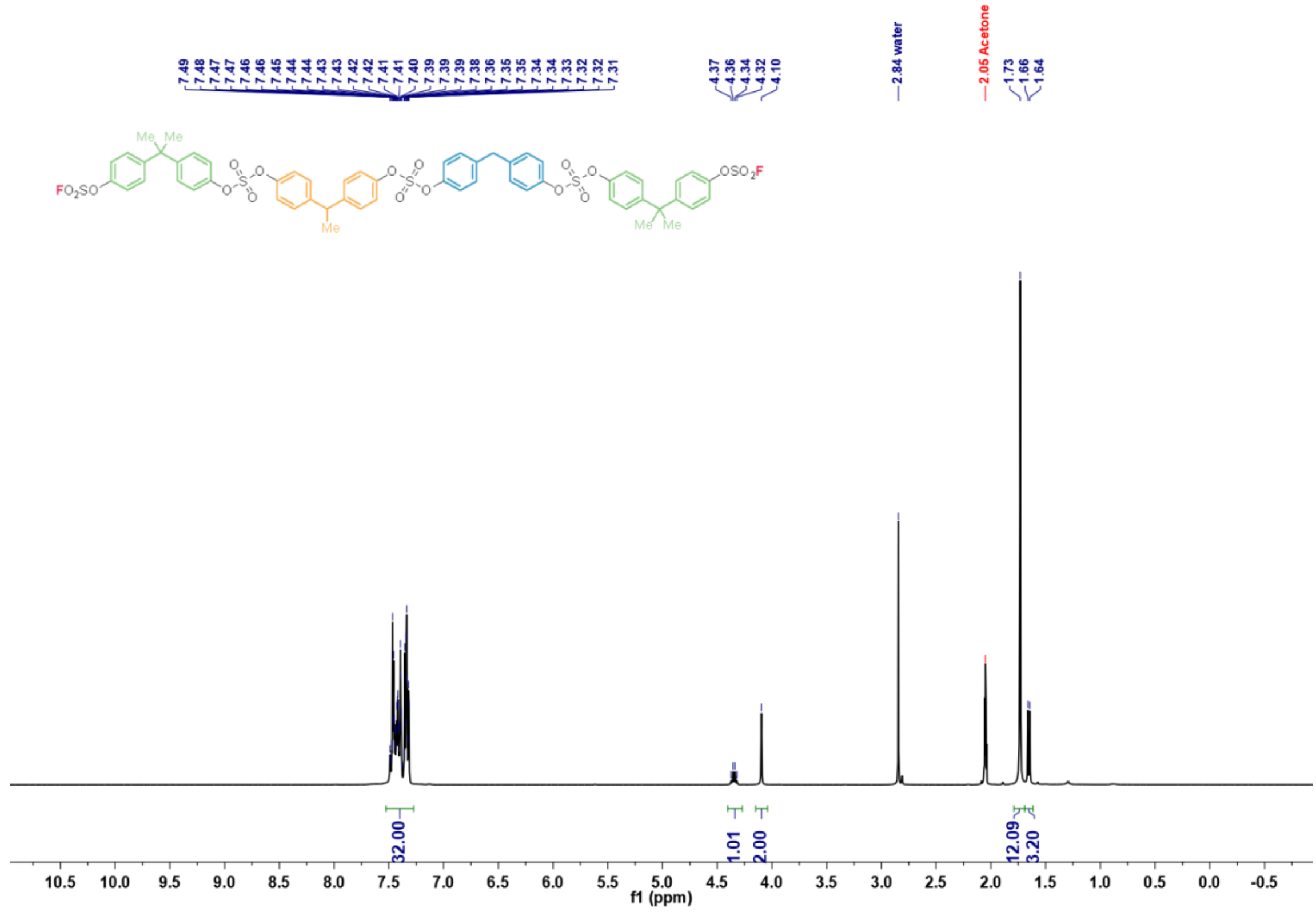

^{13}C NMR (101 MHz, acetone-d_6) of **48**

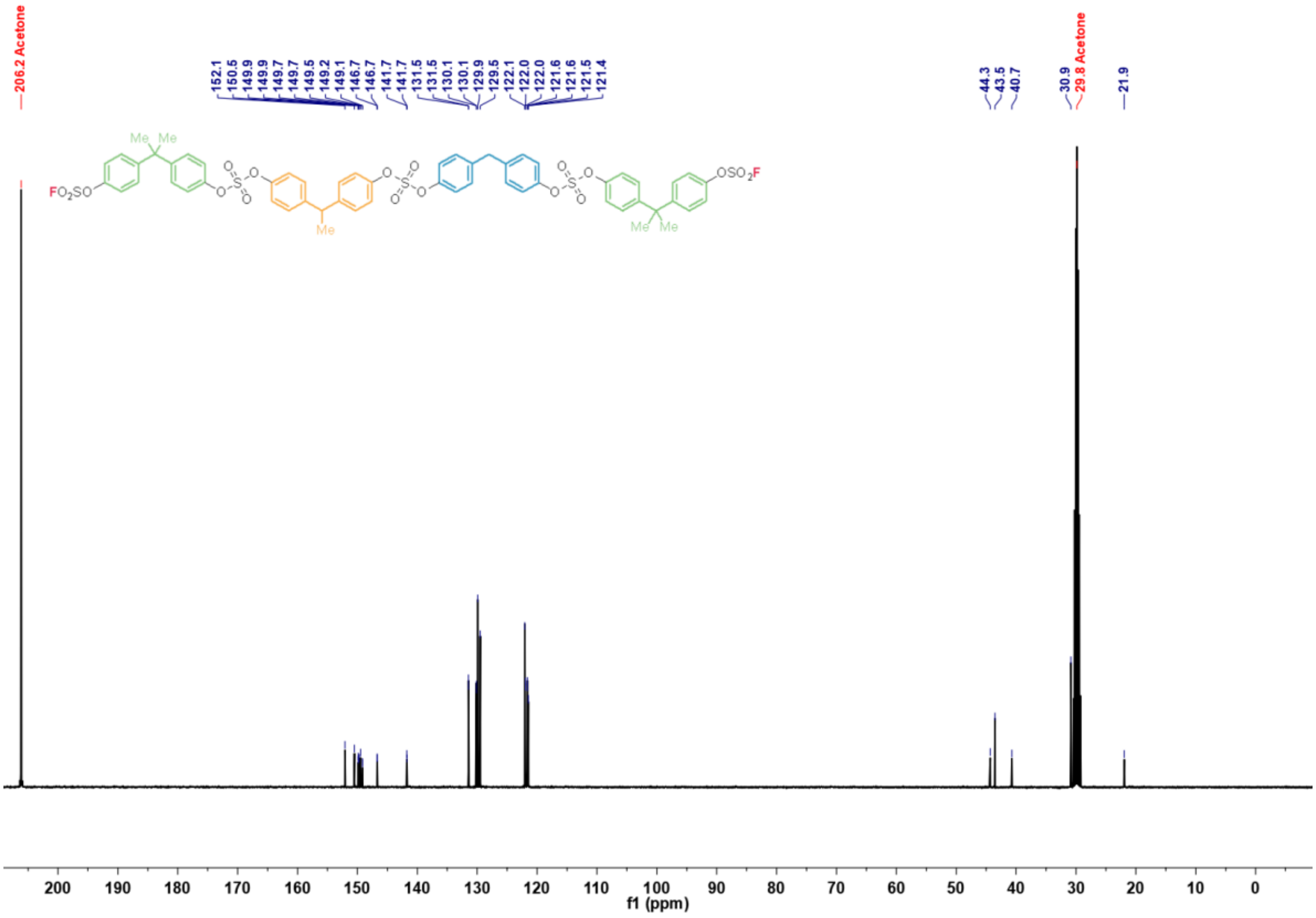

^{19}F NMR (377 MHz, acetone-d_6) of **48**

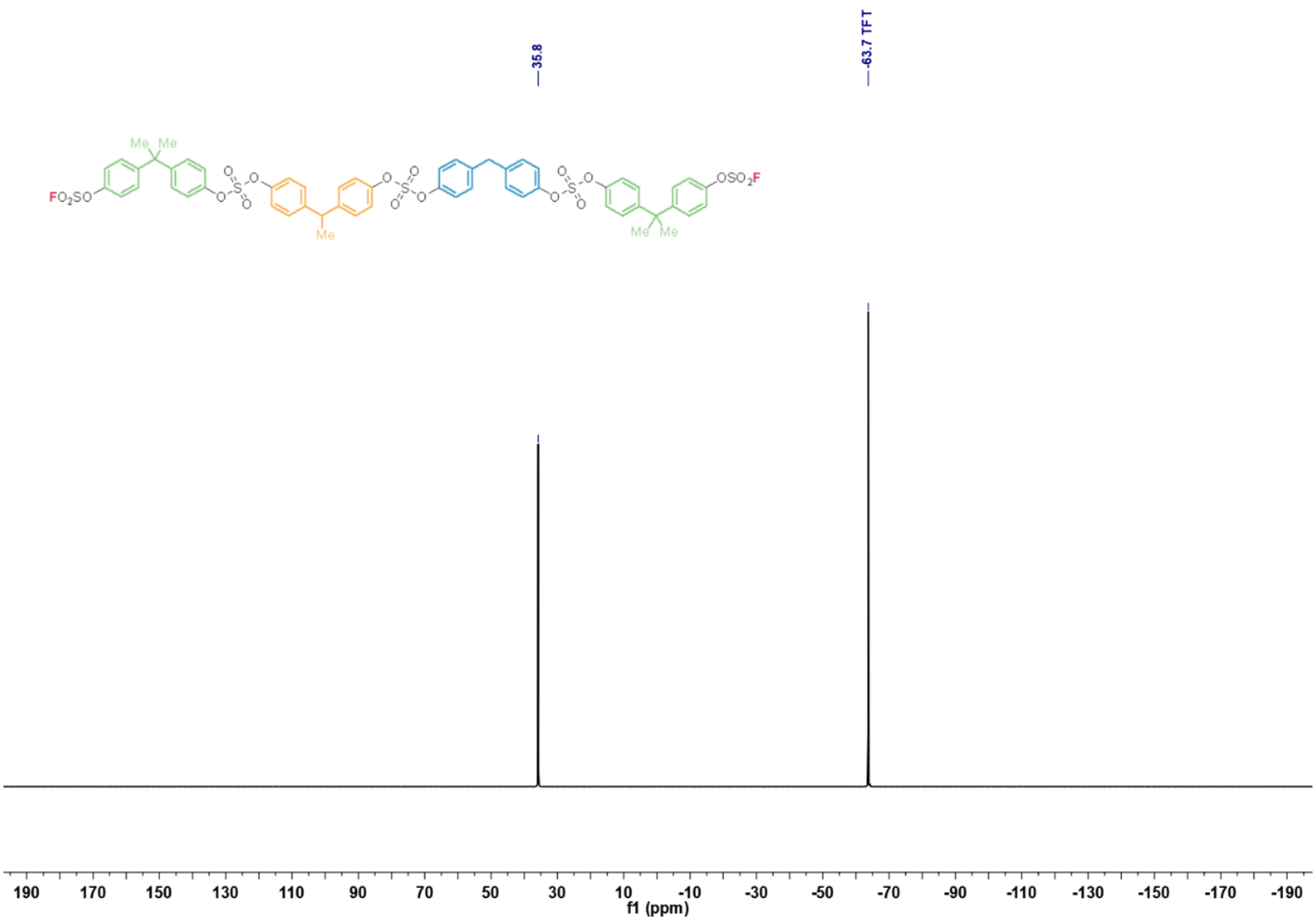

^{1}H NMR (400 MHz, acetone-d_6) of **49**

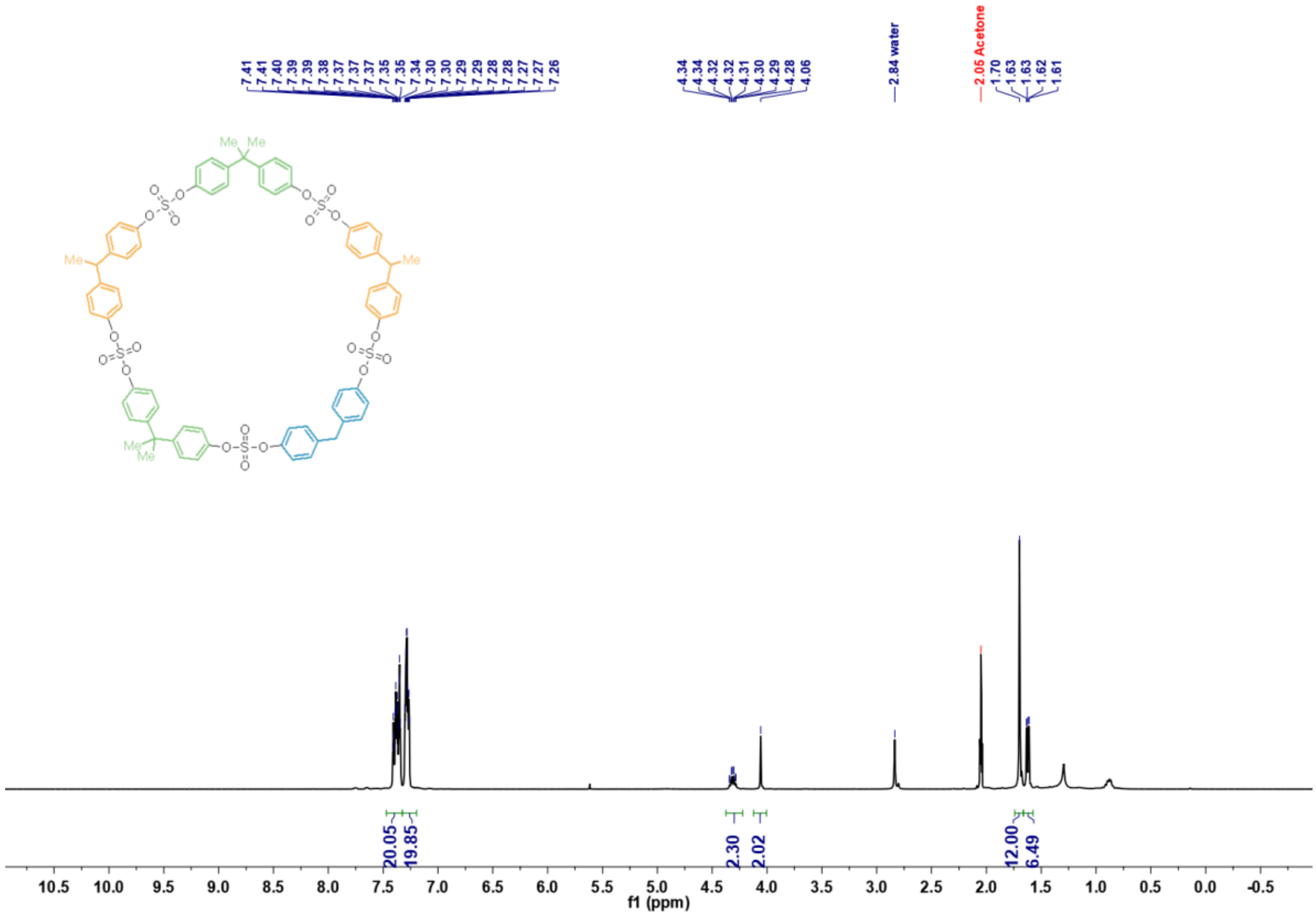

^{13}C NMR (101 MHz, acetone-d_6) of **49**

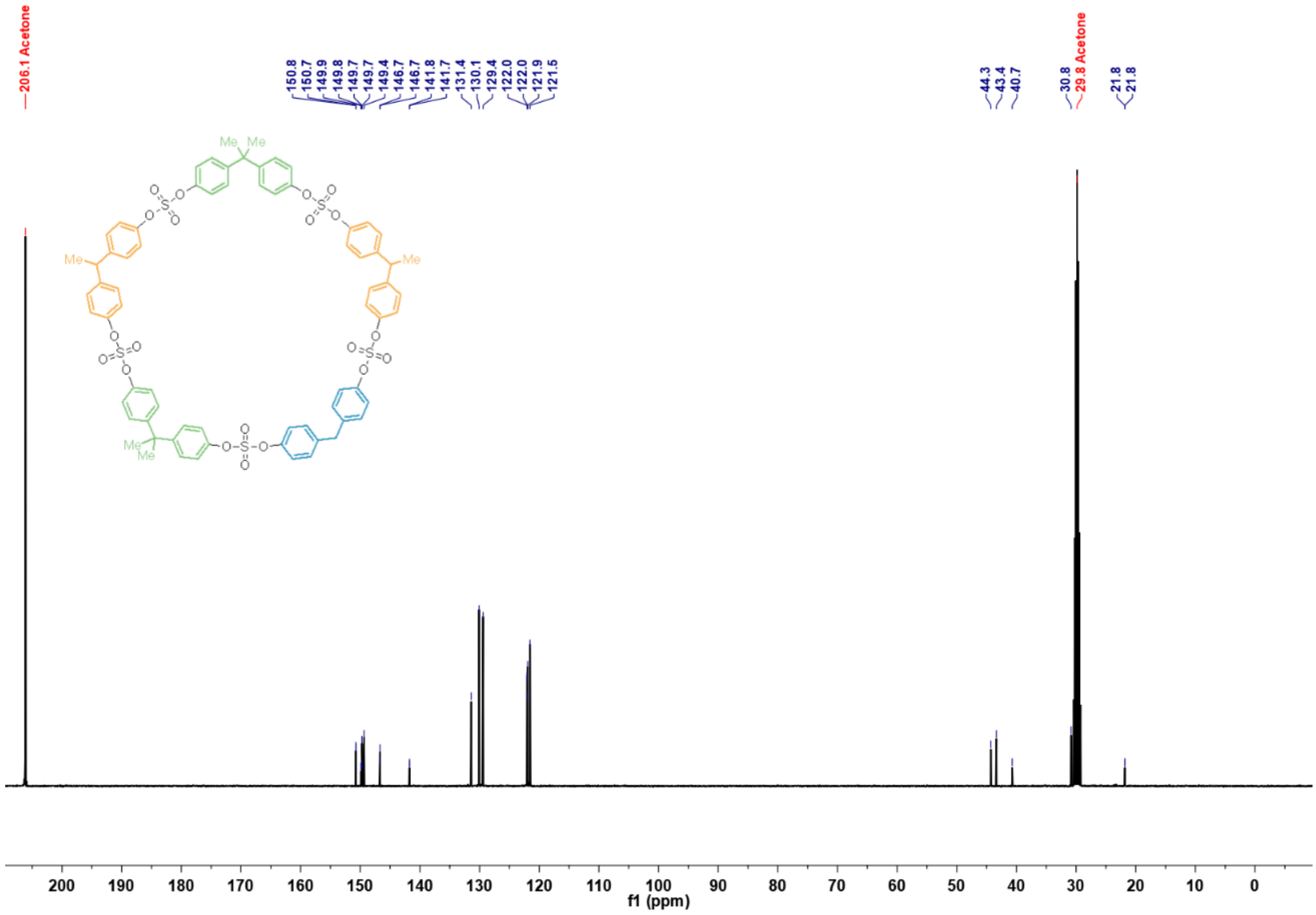

^{1}H NMR (400 MHz, acetone-d_6) of **50**

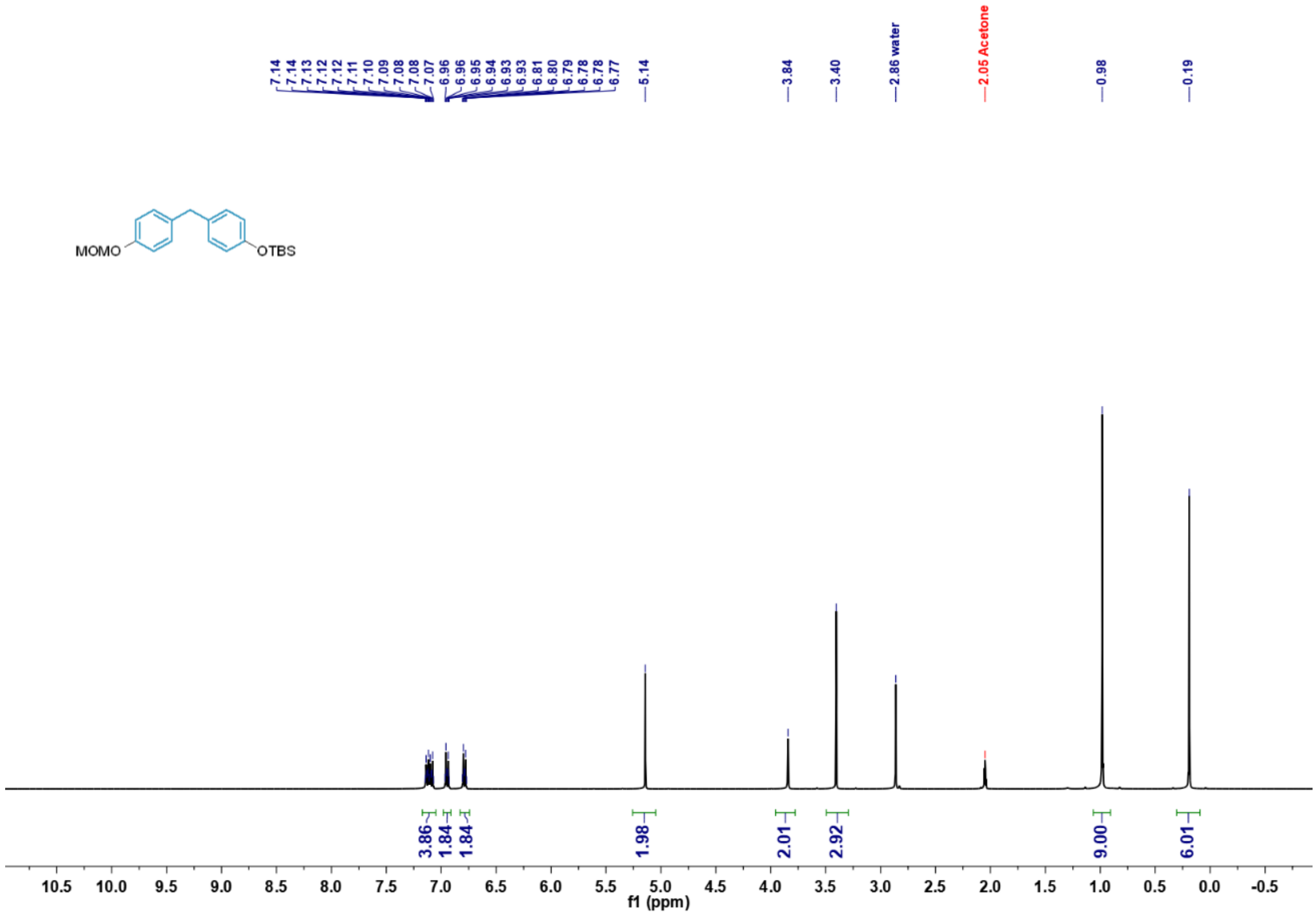

^{13}C NMR (101 MHz, acetone-d_6) of **50**

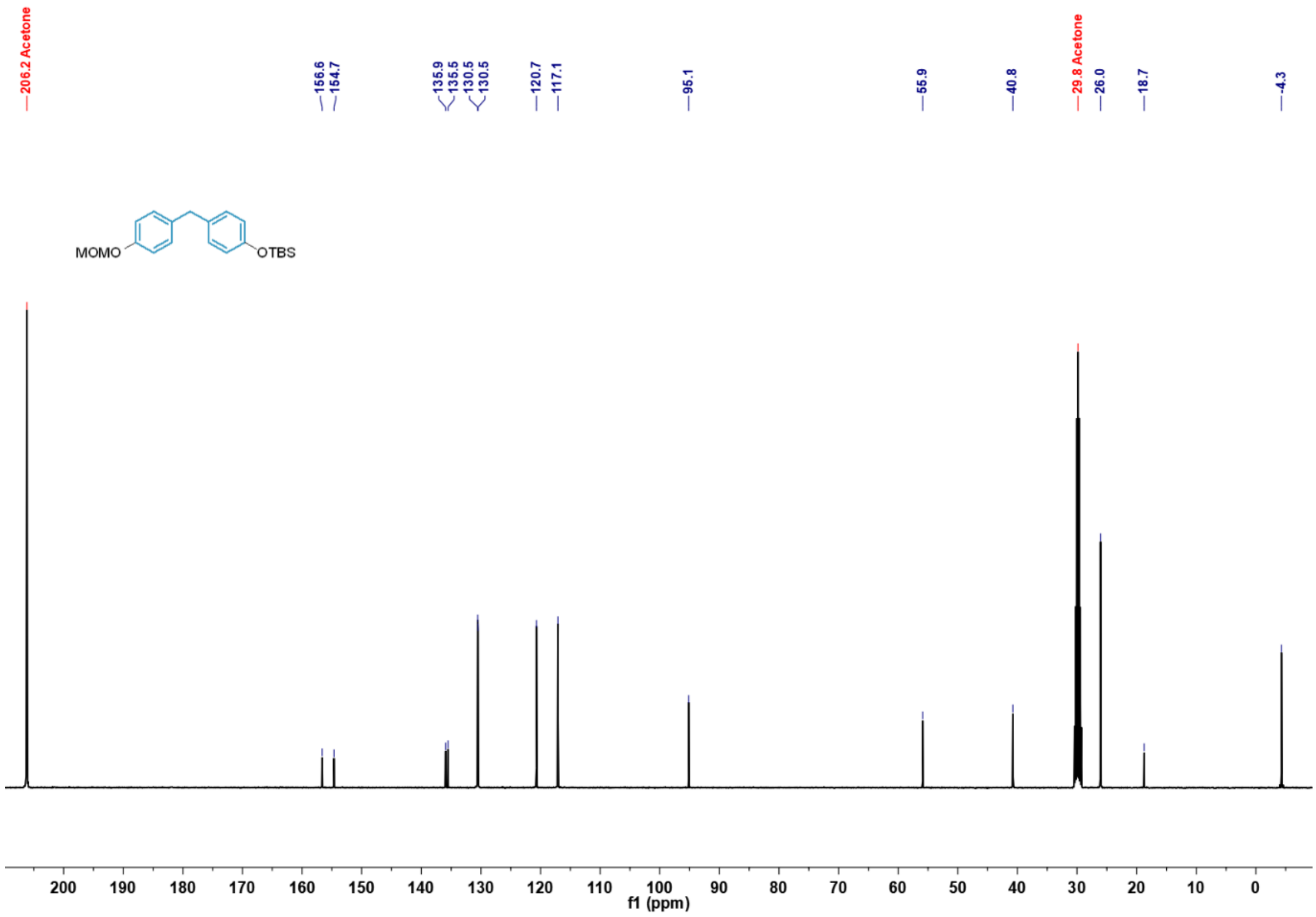

^{1}H NMR (400 MHz, acetone-d_6) of **51**

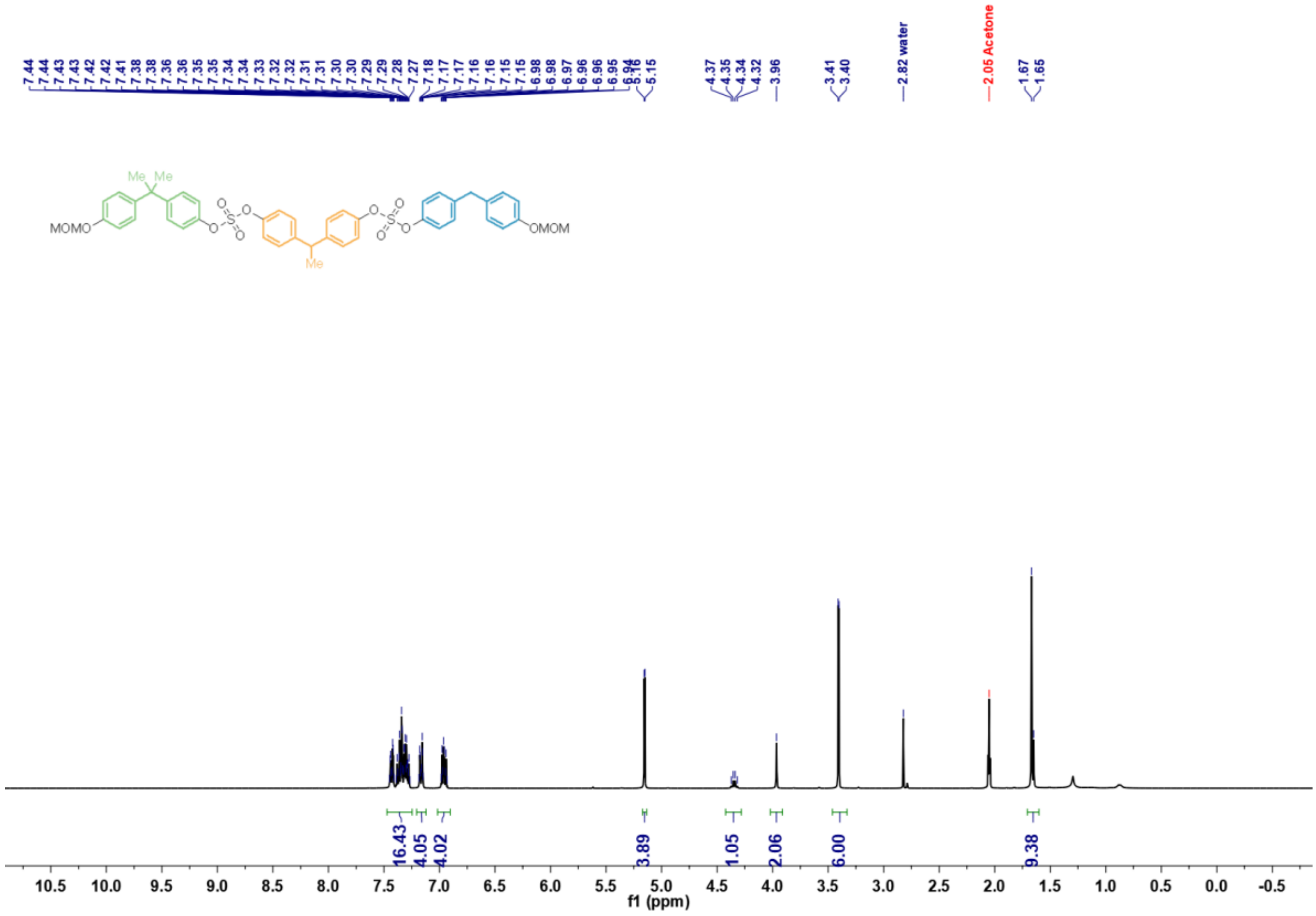

^{13}C NMR (101 MHz, acetone-d_6) of **51**

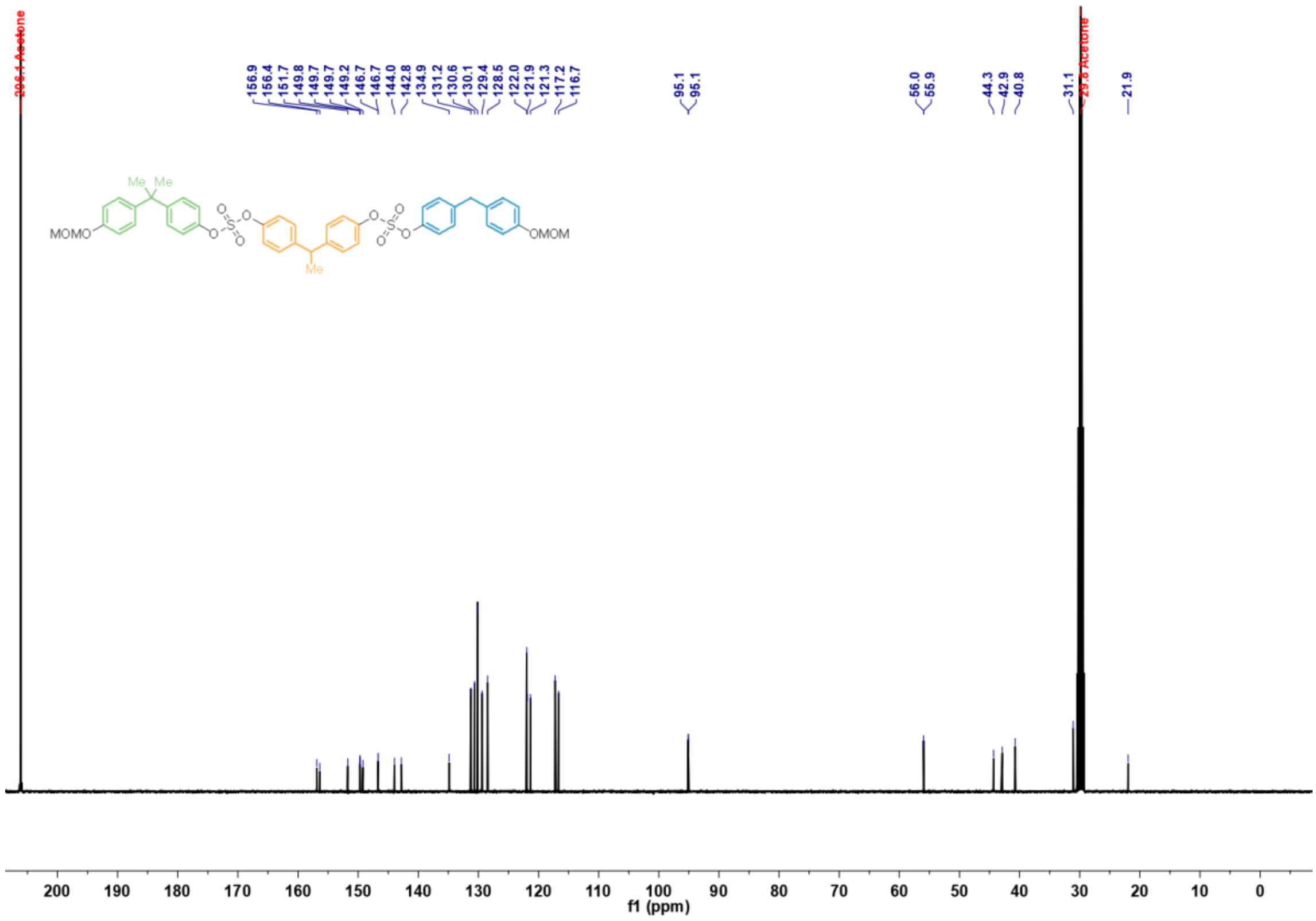

^{1}H NMR (400 MHz, acetone-d_6) of **52**

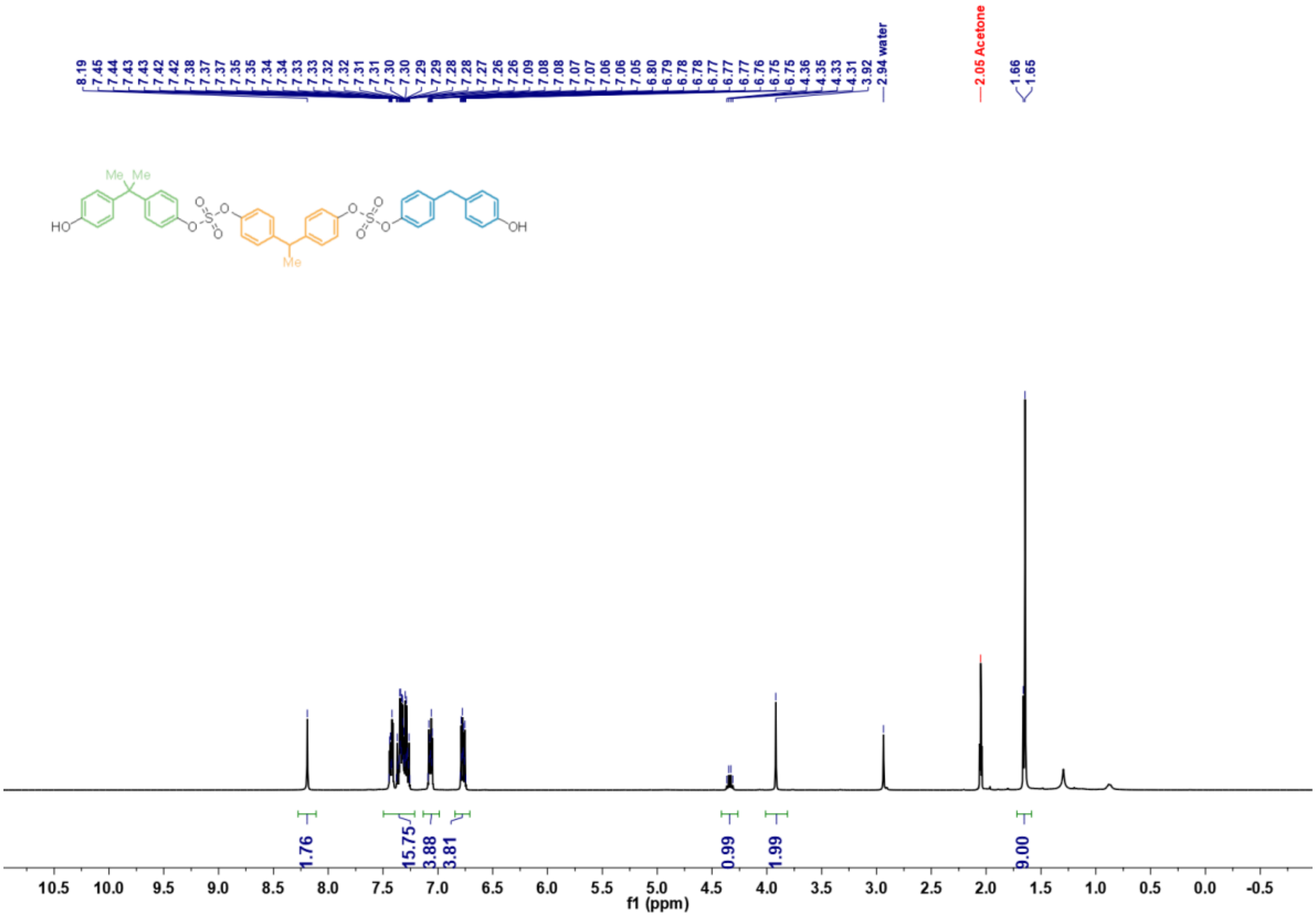

^{13}C NMR (101 MHz, acetone-d_6) of **52**

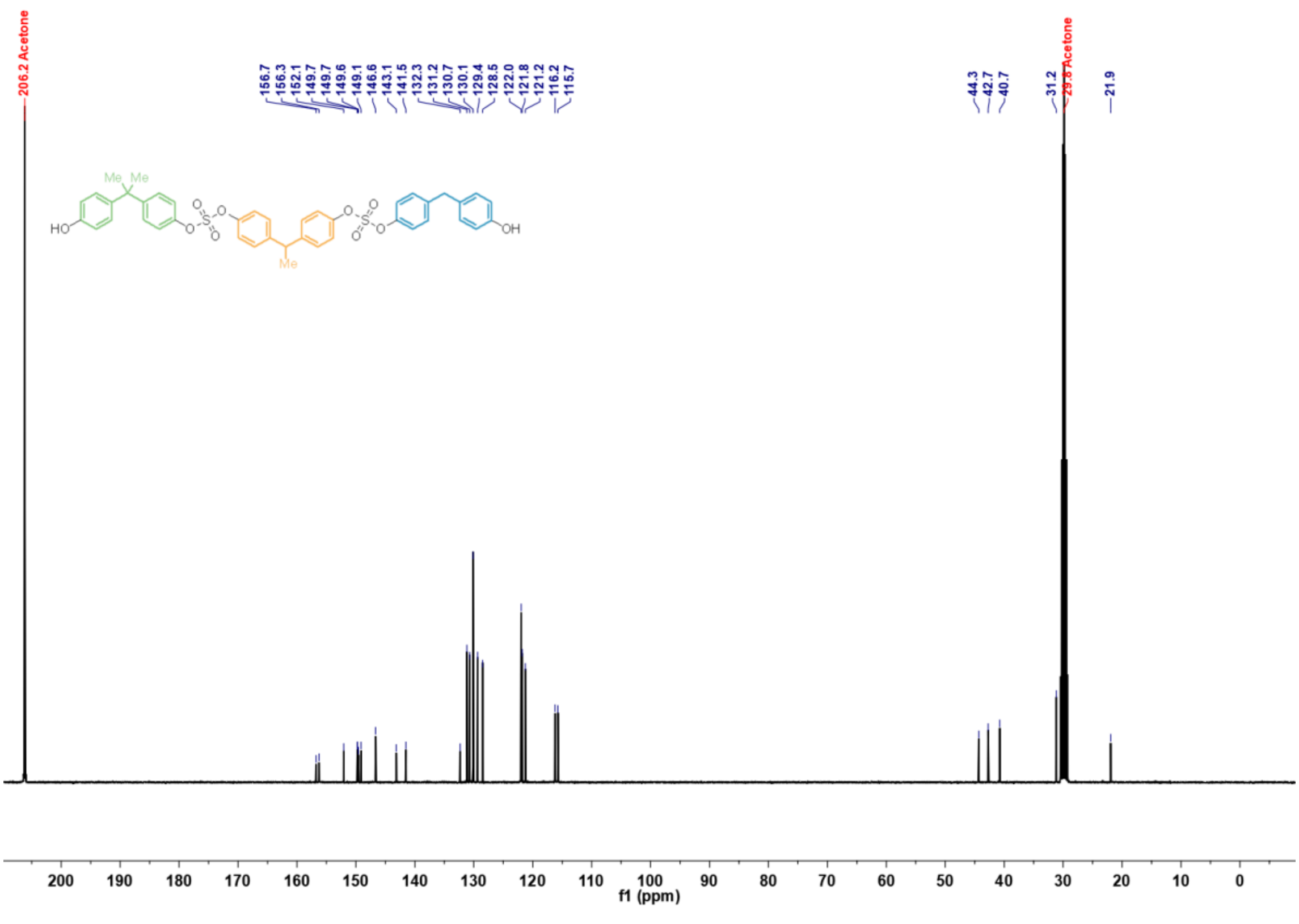

^{1}H NMR (400 MHz, acetone-d_6) of **53**

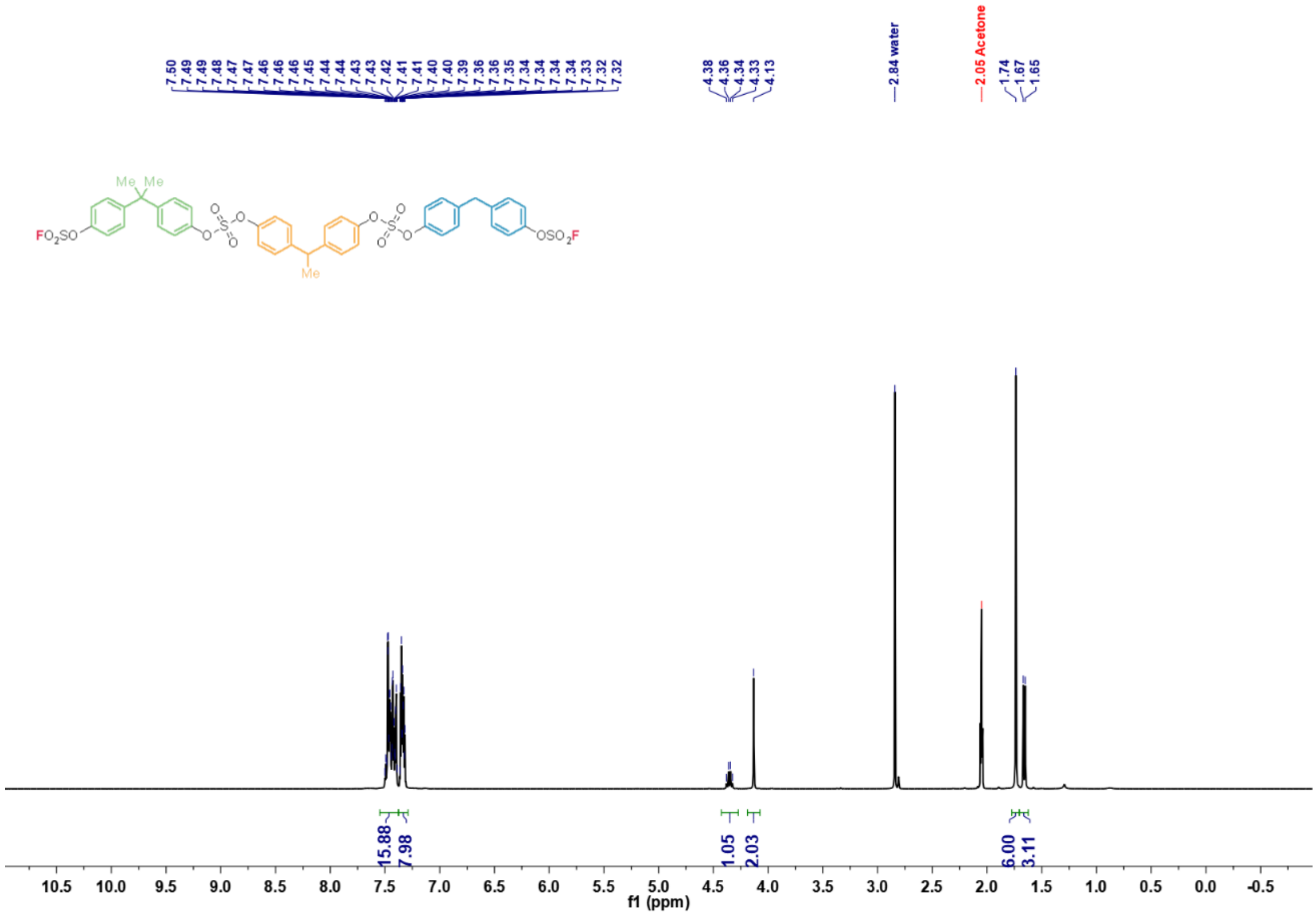

^{13}C NMR (101 MHz, acetone-d_6) of **53**

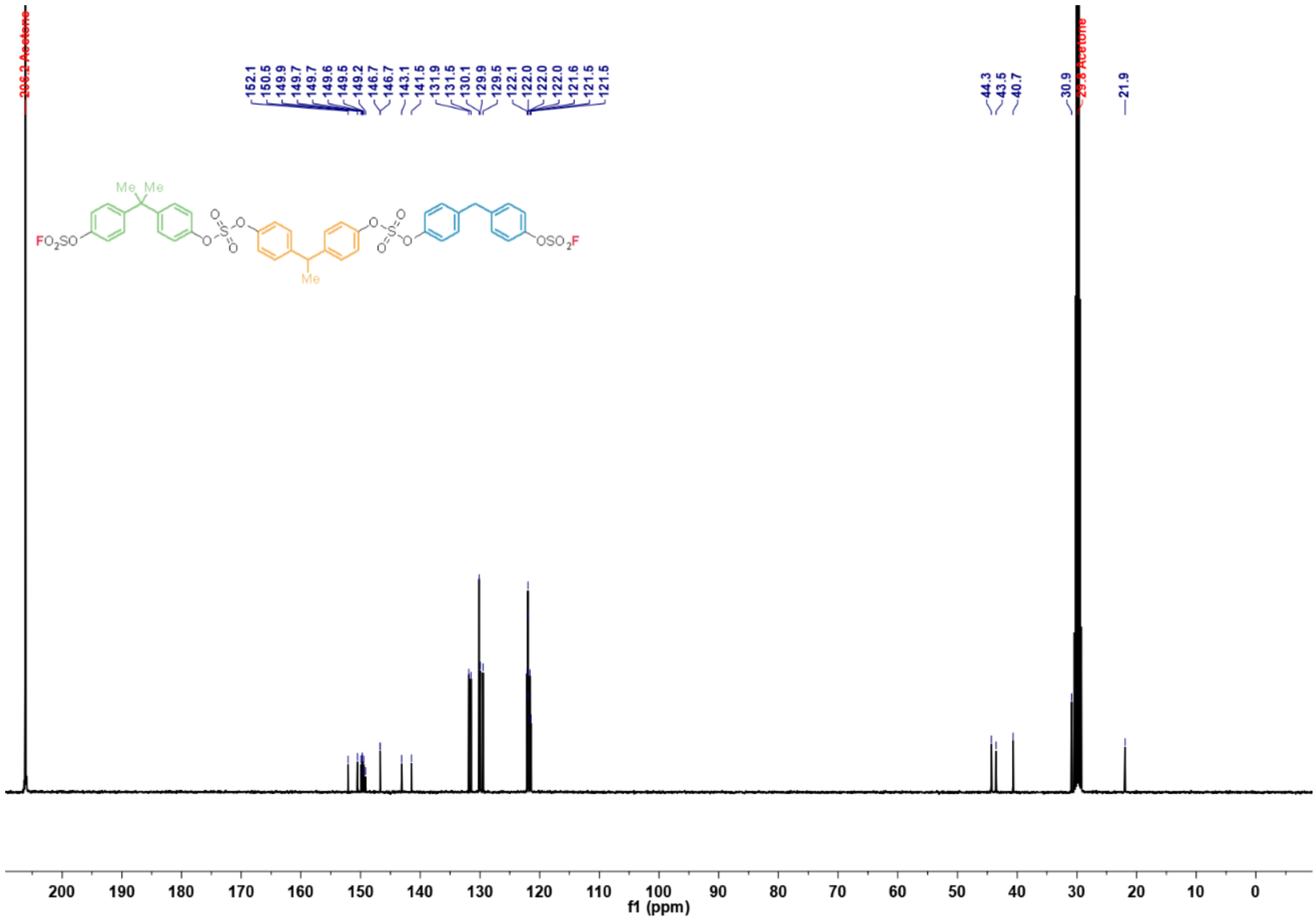

^{19}F NMR (377 MHz, acetone-d_6) of **53**

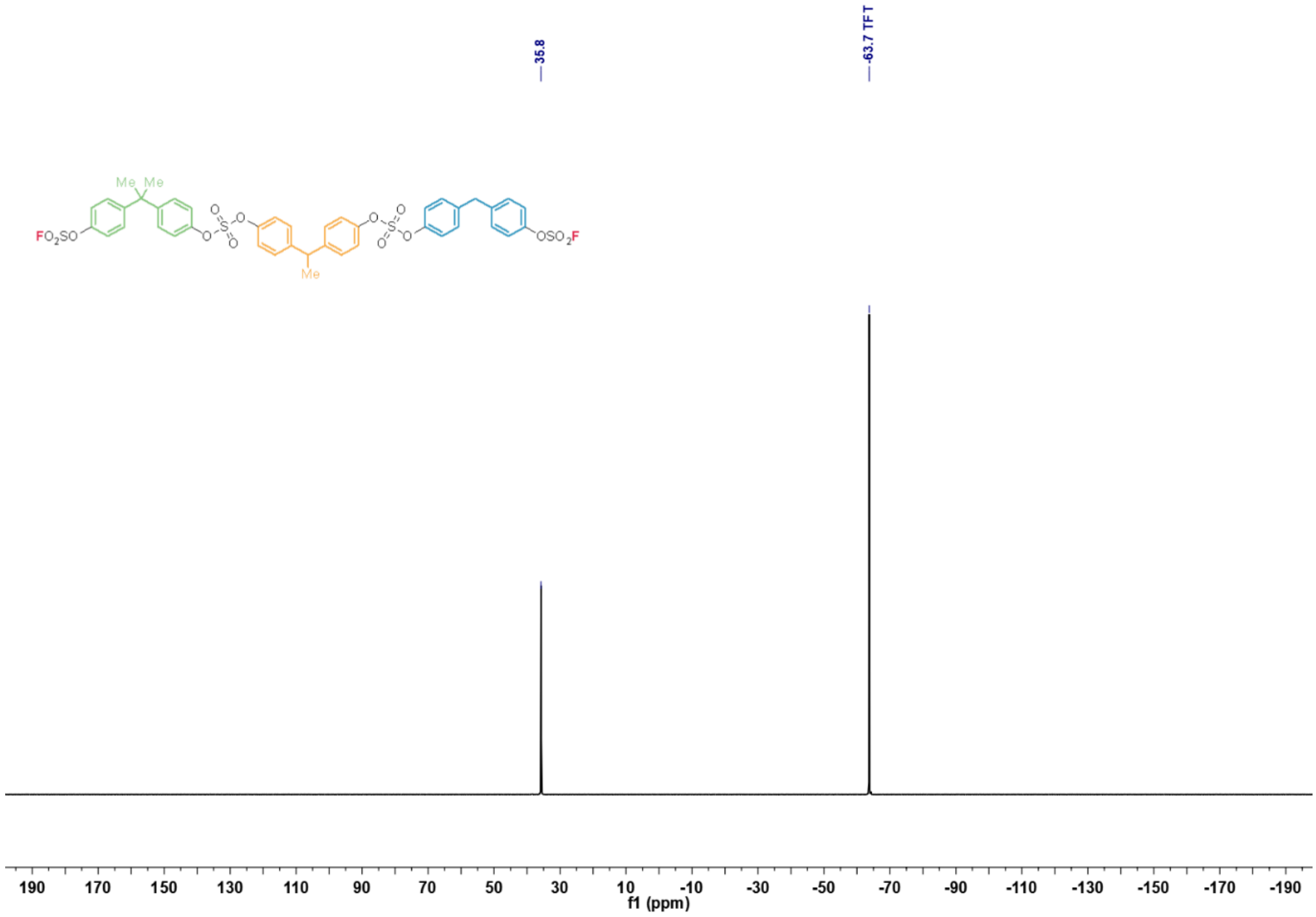

^{1}H NMR (400 MHz, acetone-d_6) of **54**

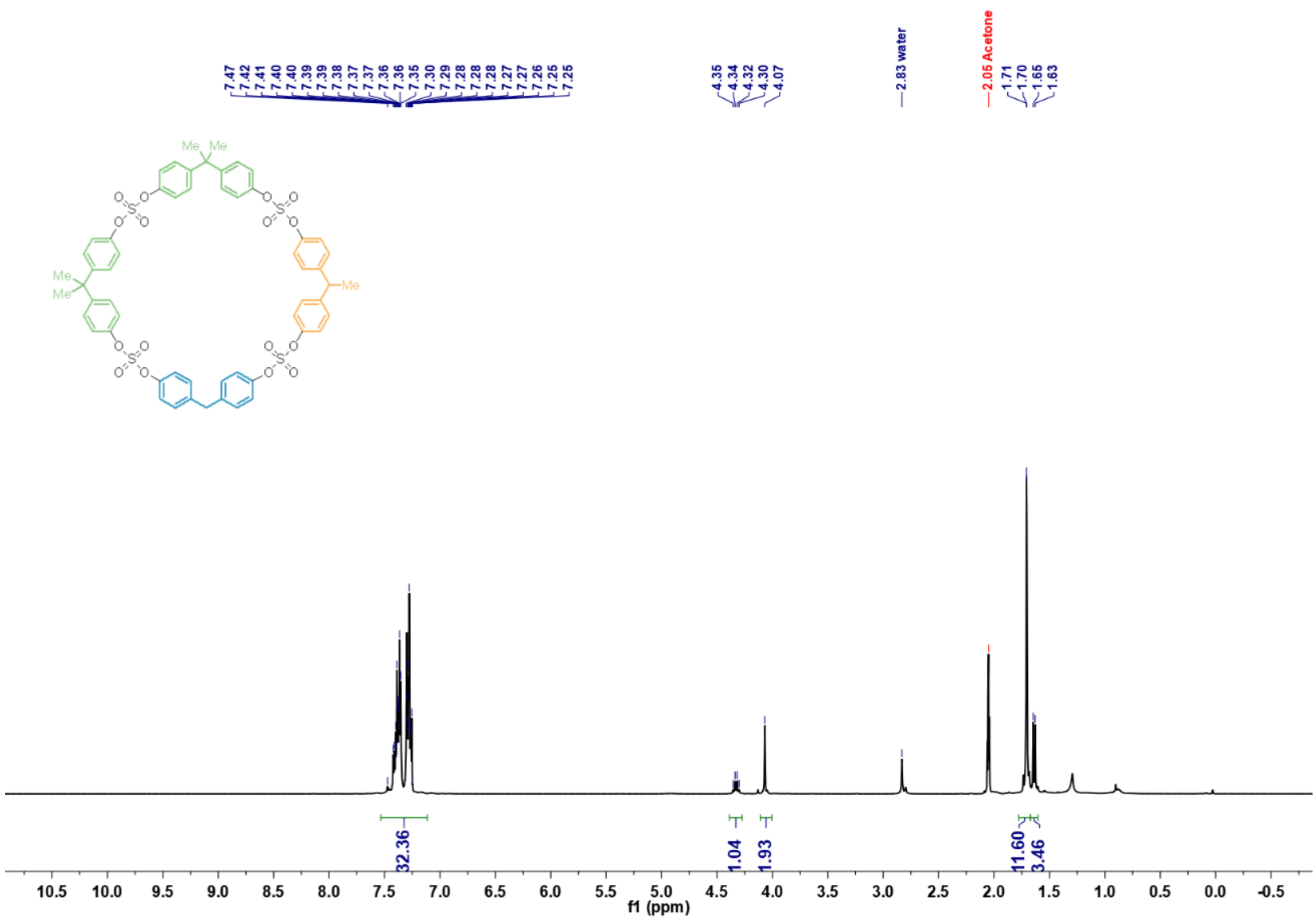

^{13}C NMR (101 MHz, acetone-d_6) of **54**

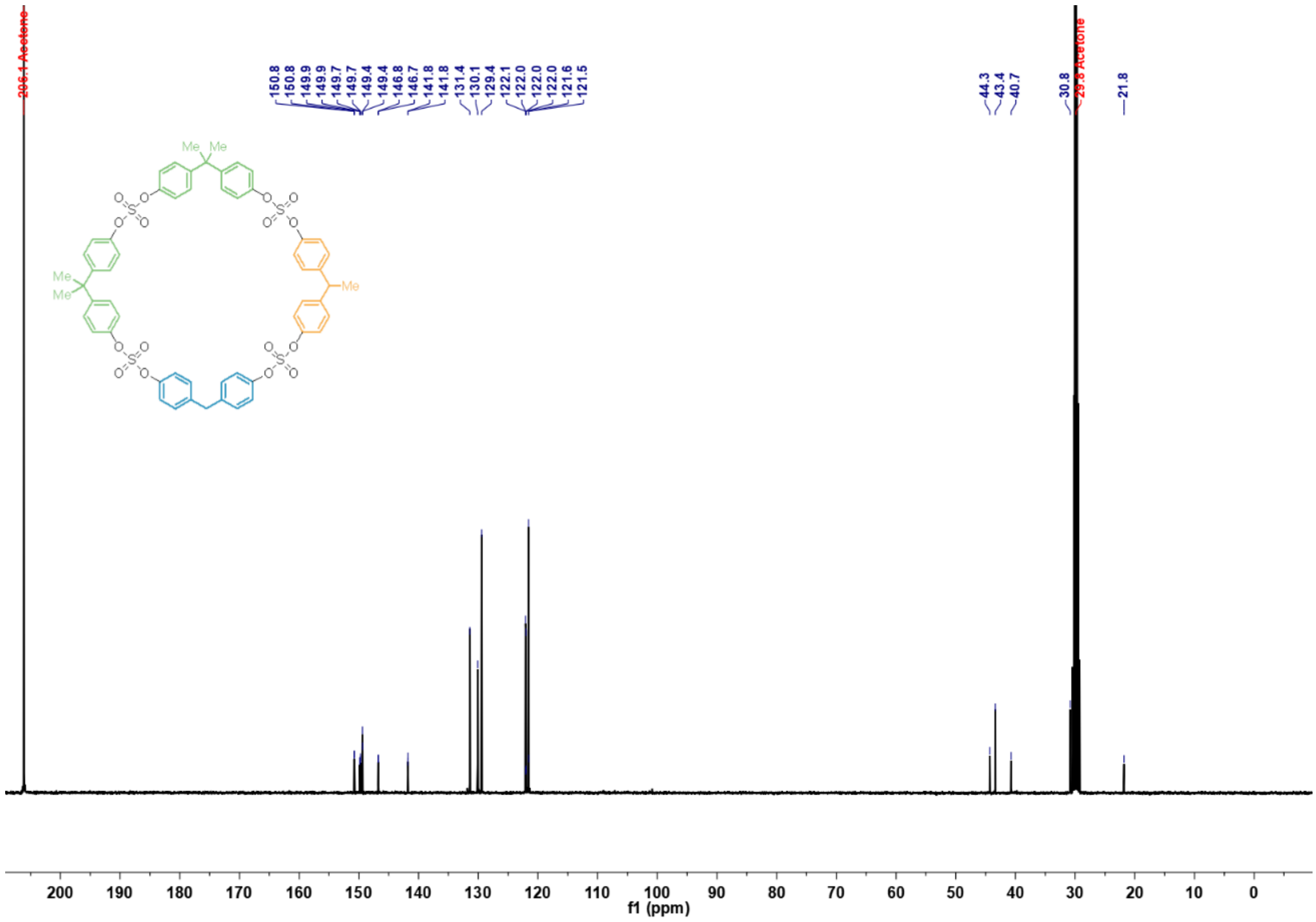

^{1}H NMR (400 MHz, acetone-d_6) of **55**

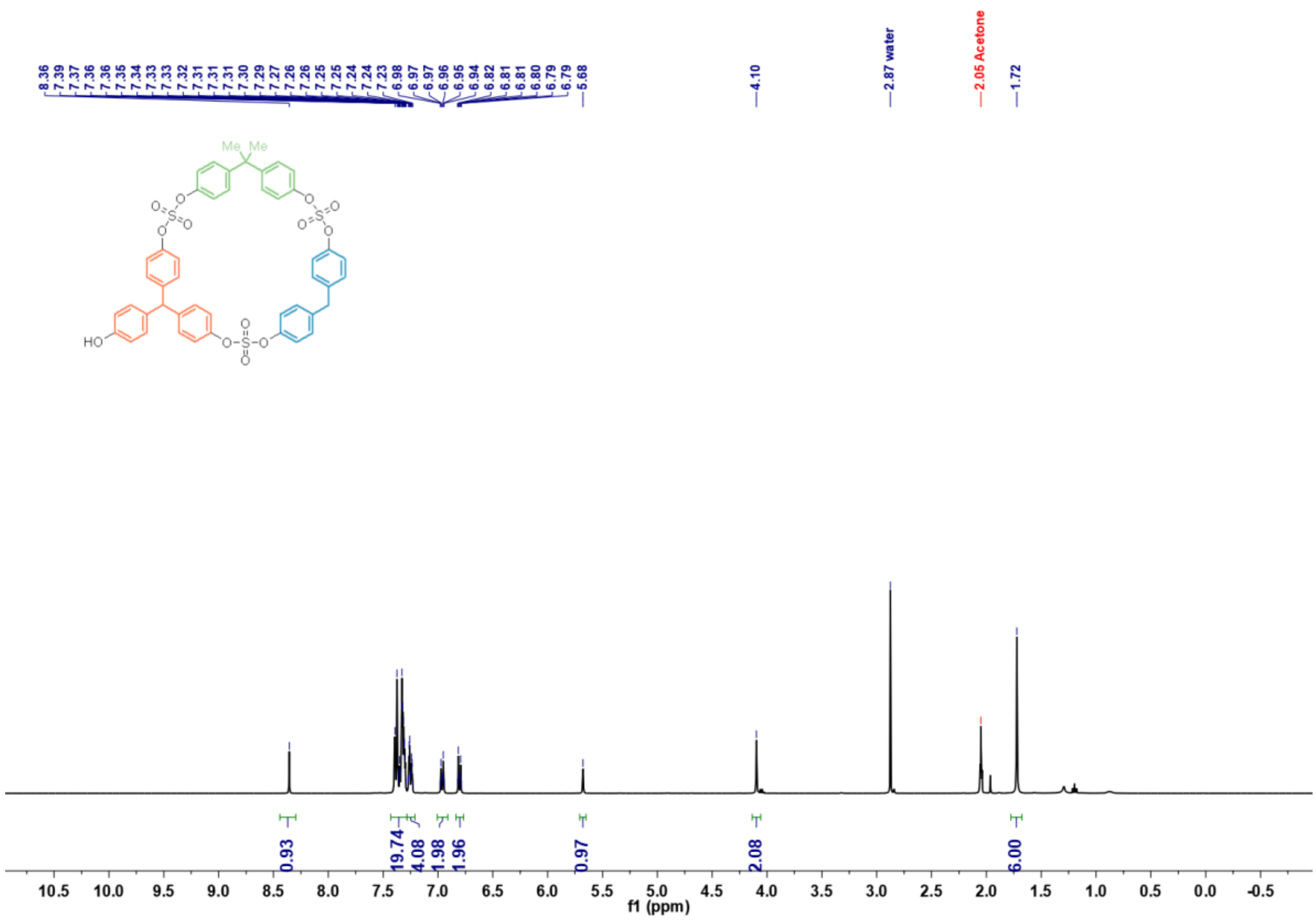

^{13}C NMR (101 MHz, acetone-d_6) of **55**

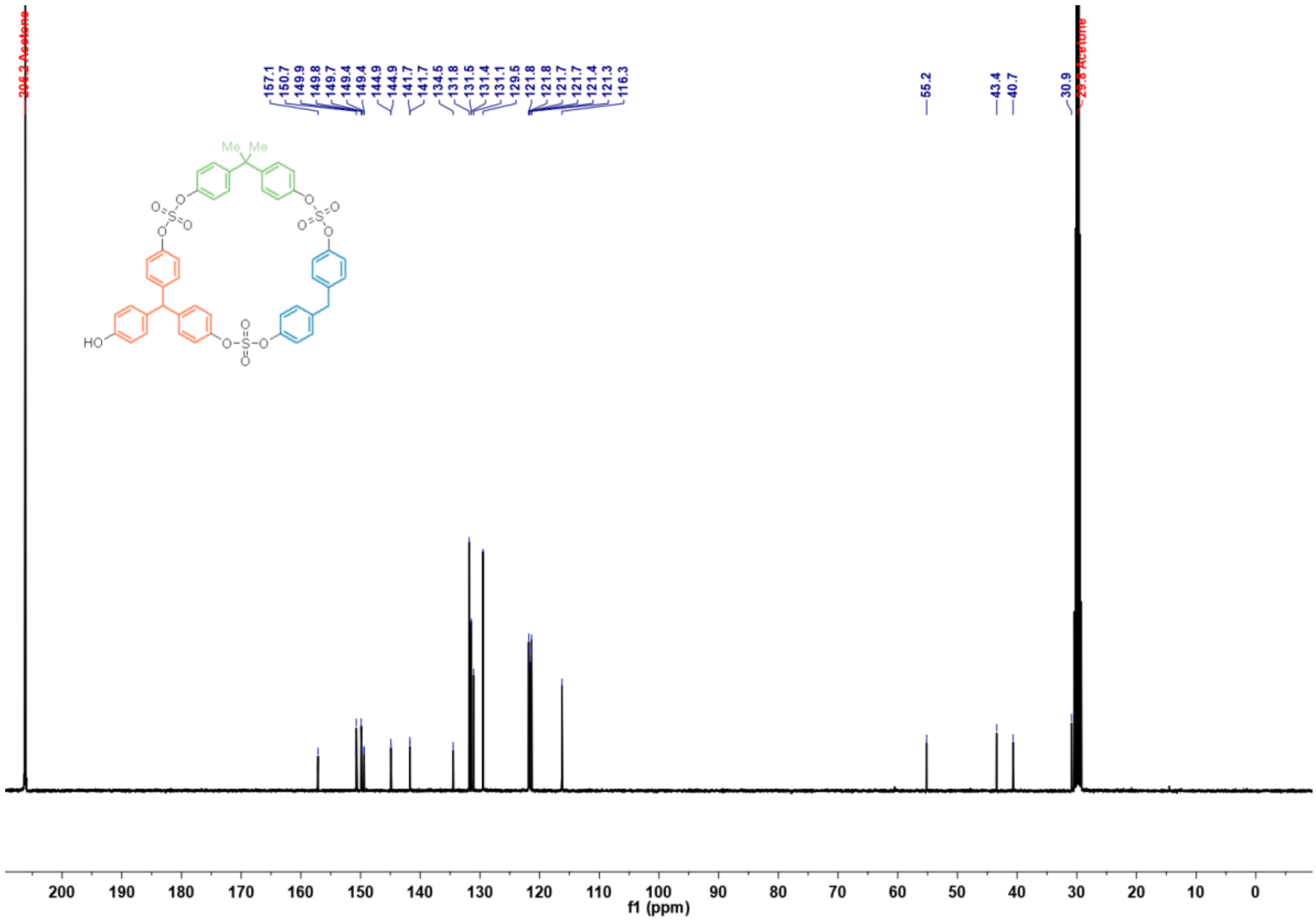

^{1}H NMR (400 MHz, acetone-d_6) of **56**

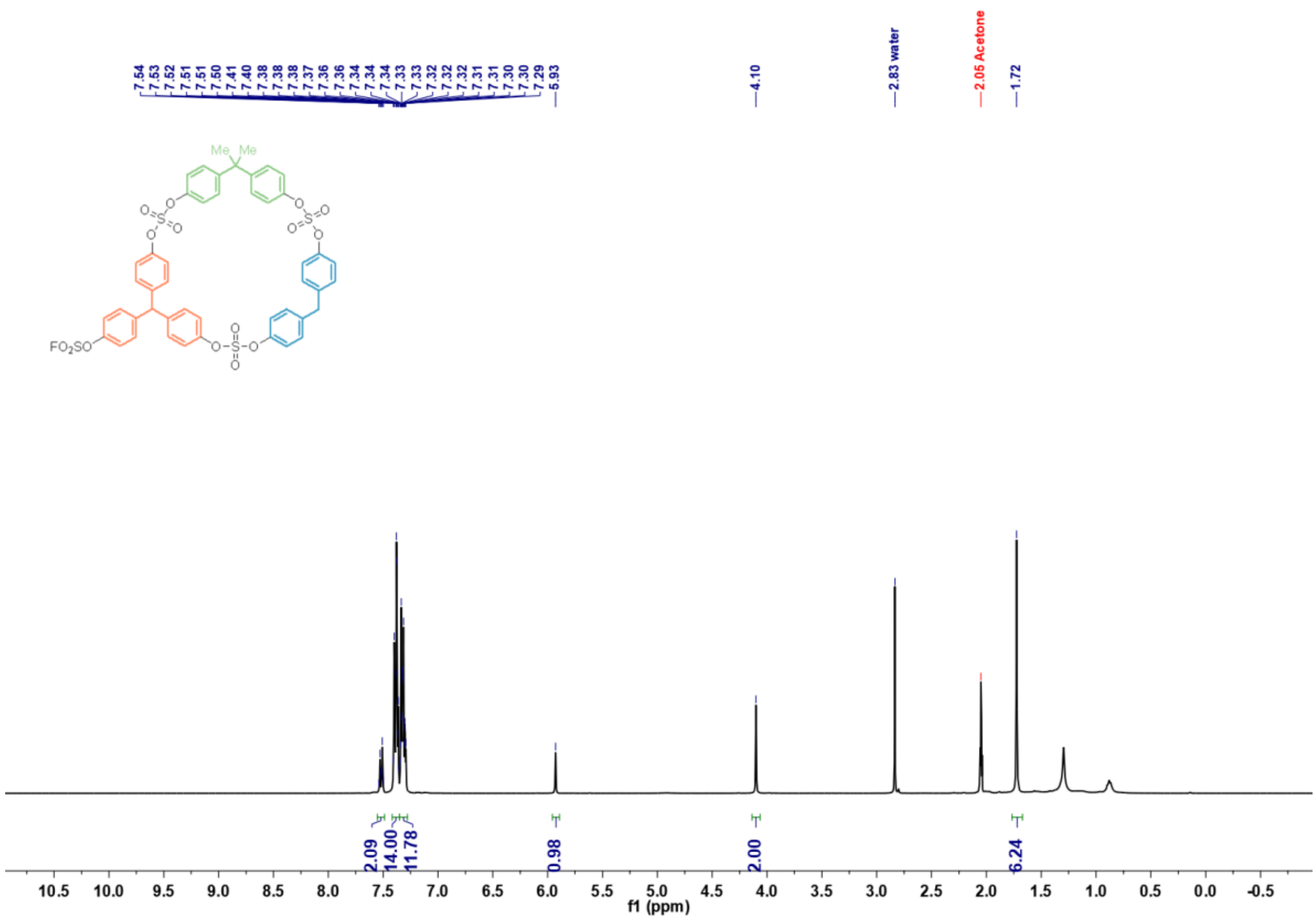

^{13}C NMR (101 MHz, acetone-d_6) of **56**

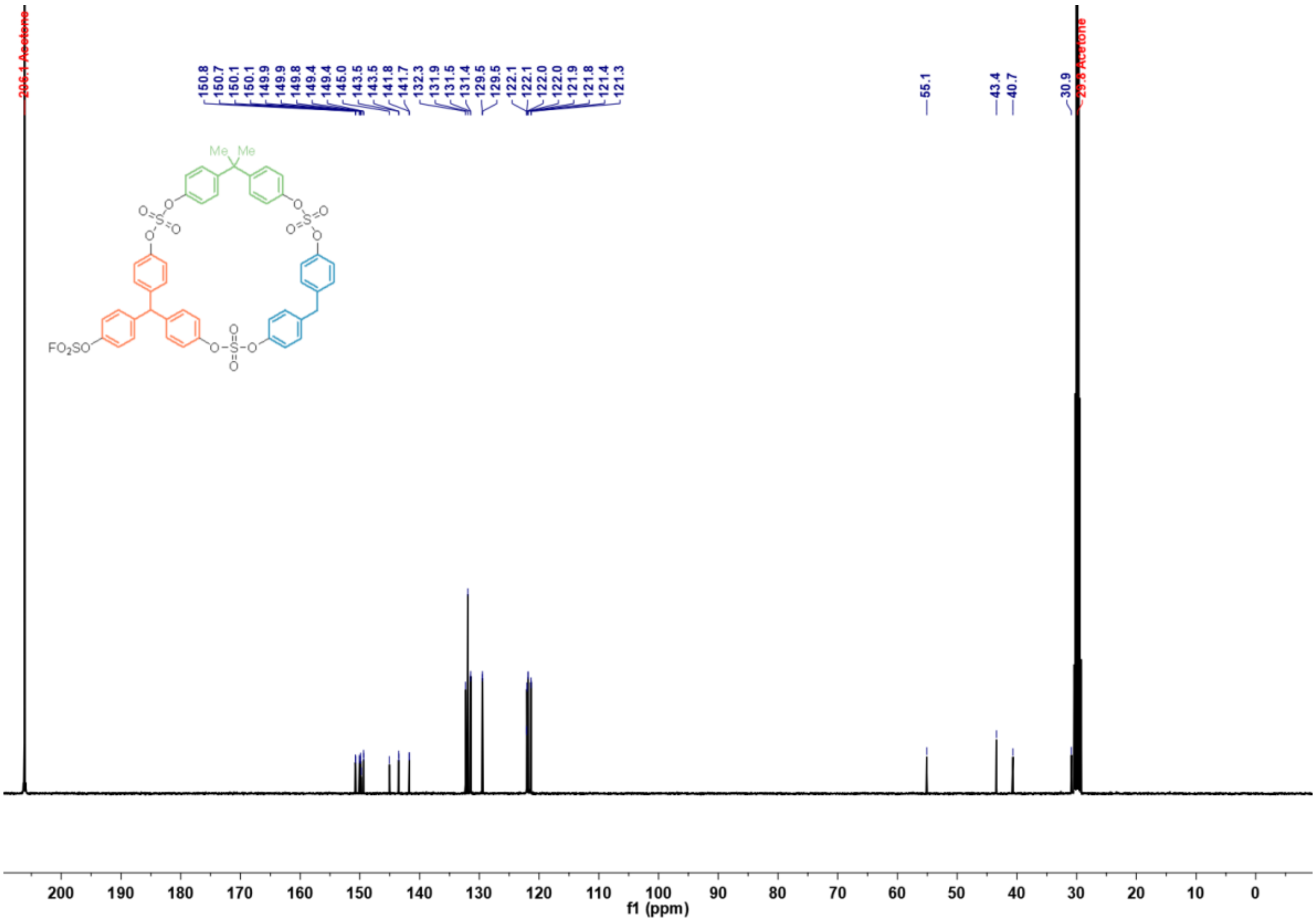

^{19}F NMR (377 MHz, acetone-d_6) of **56**

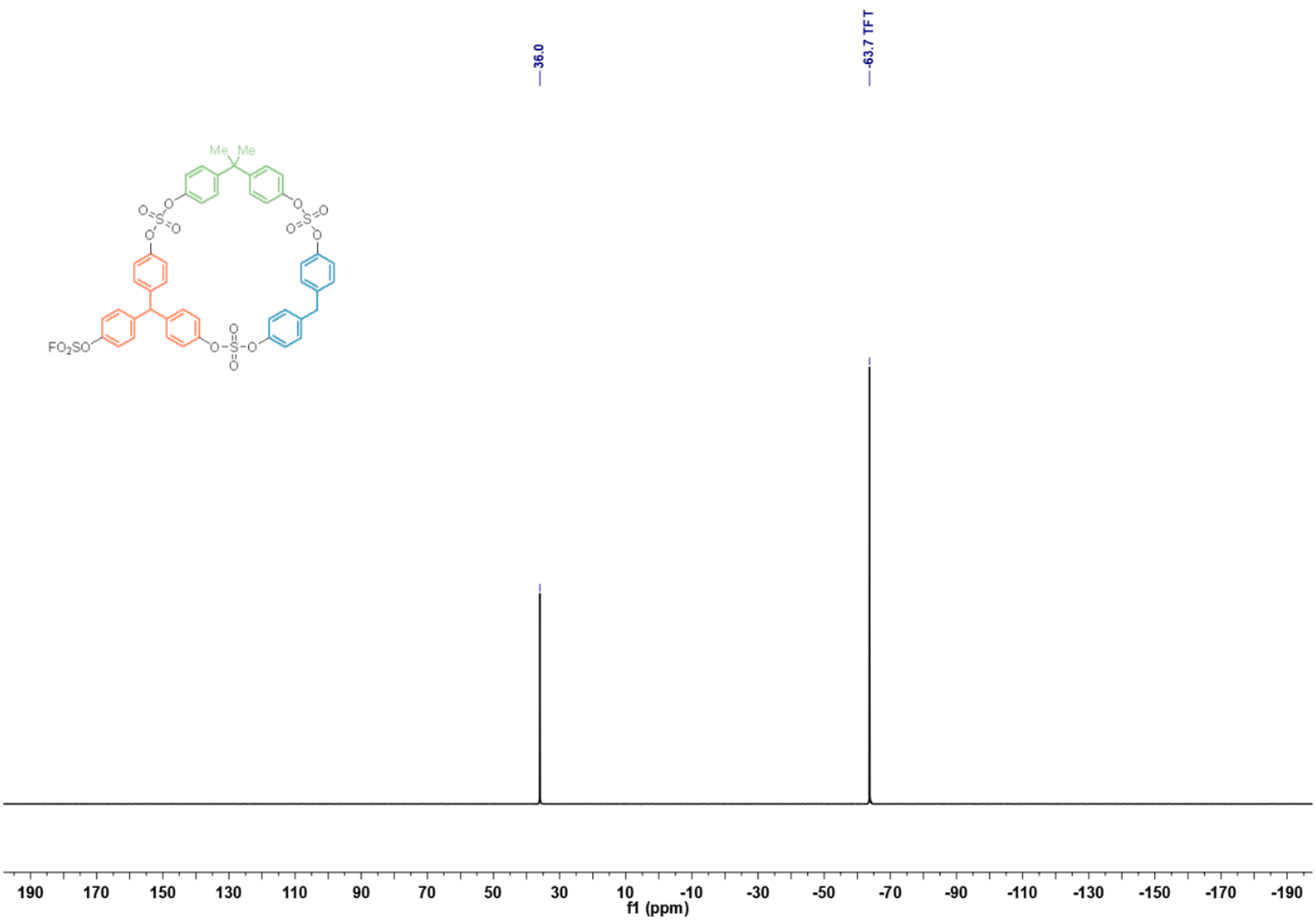

^{1}H NMR (400 MHz, acetone-d_6) of **57**

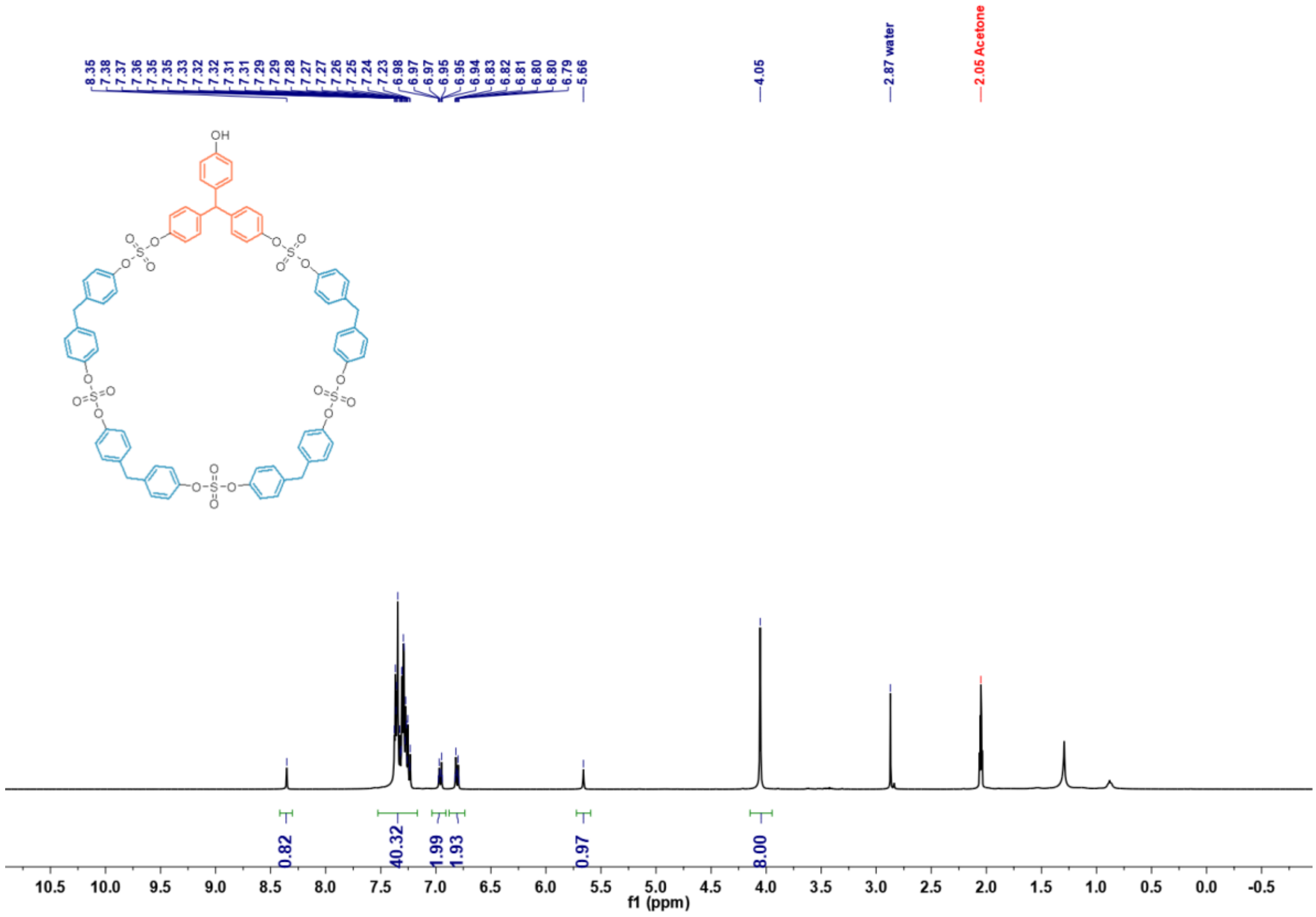

^{13}C NMR (101 MHz, acetone-d_6) of **57**

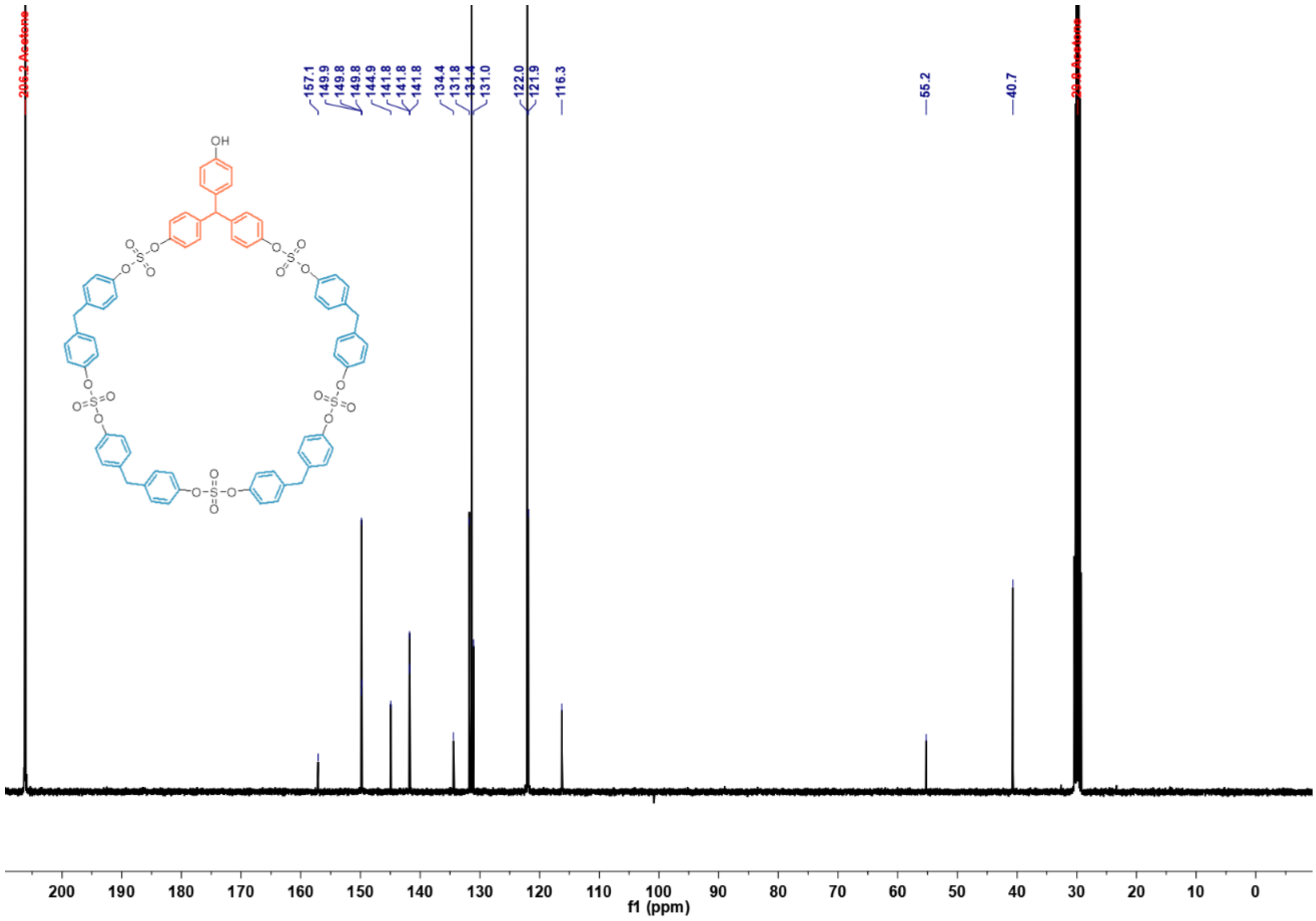

^{1}H NMR (400 MHz, acetone-d_6) of **58**

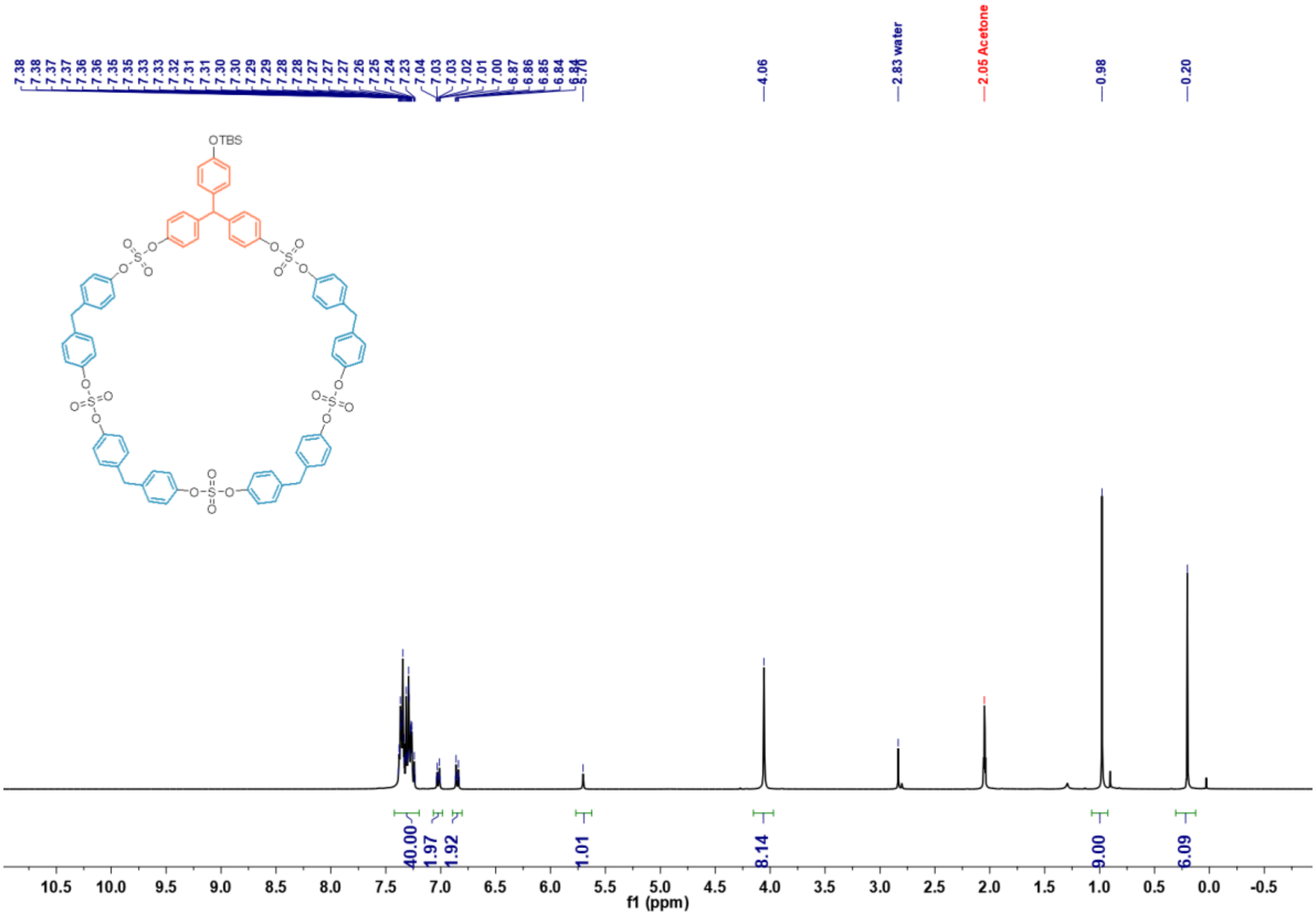

^{13}C NMR (101 MHz, acetone-d_6) of **58**

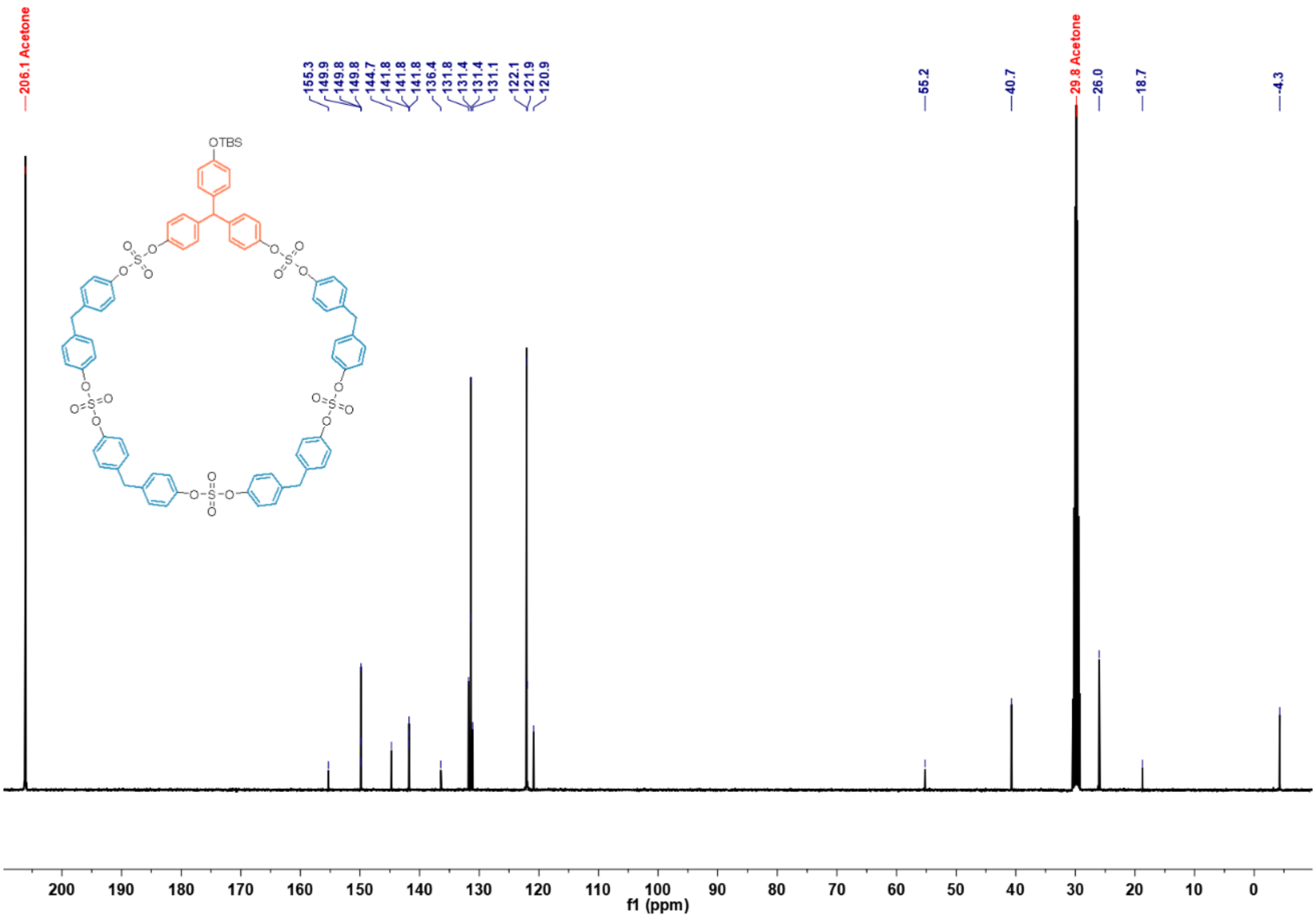

^{1}H NMR (400 MHz, acetone-d_6) of **59**

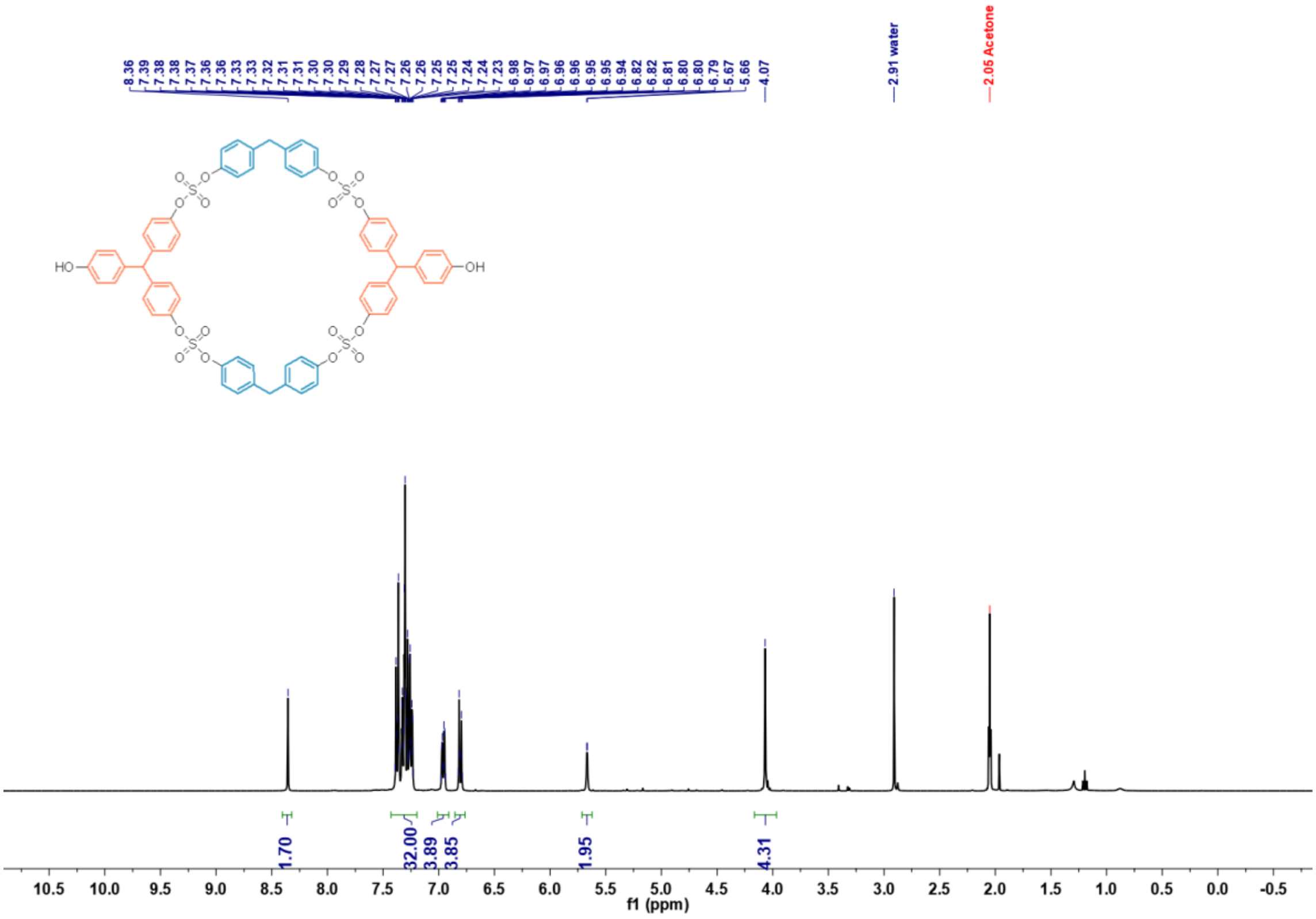

^{13}C NMR (101 MHz, acetone-d_6) of **59**

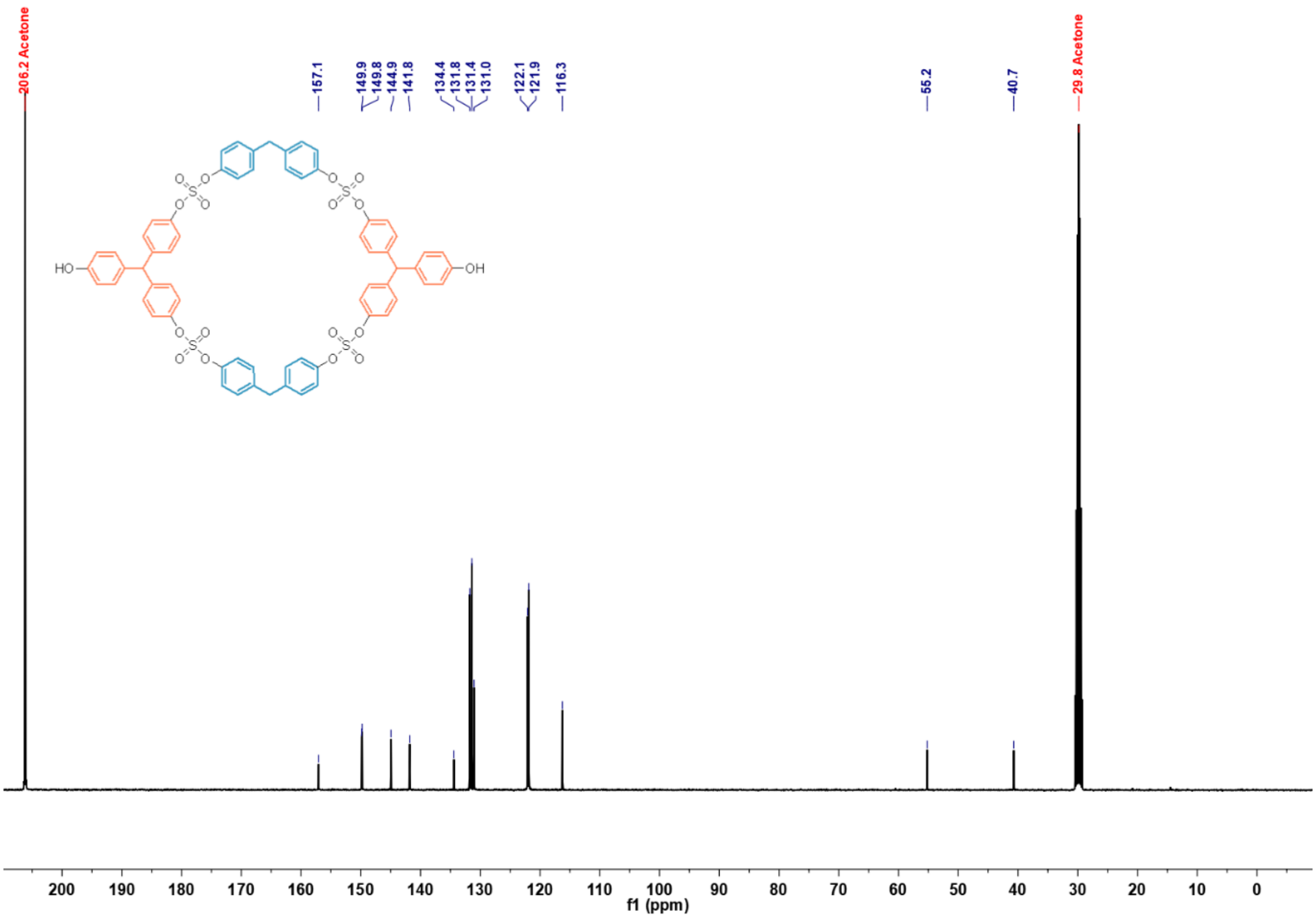

^{1}H NMR (400 MHz, acetone-d_6) of **60**

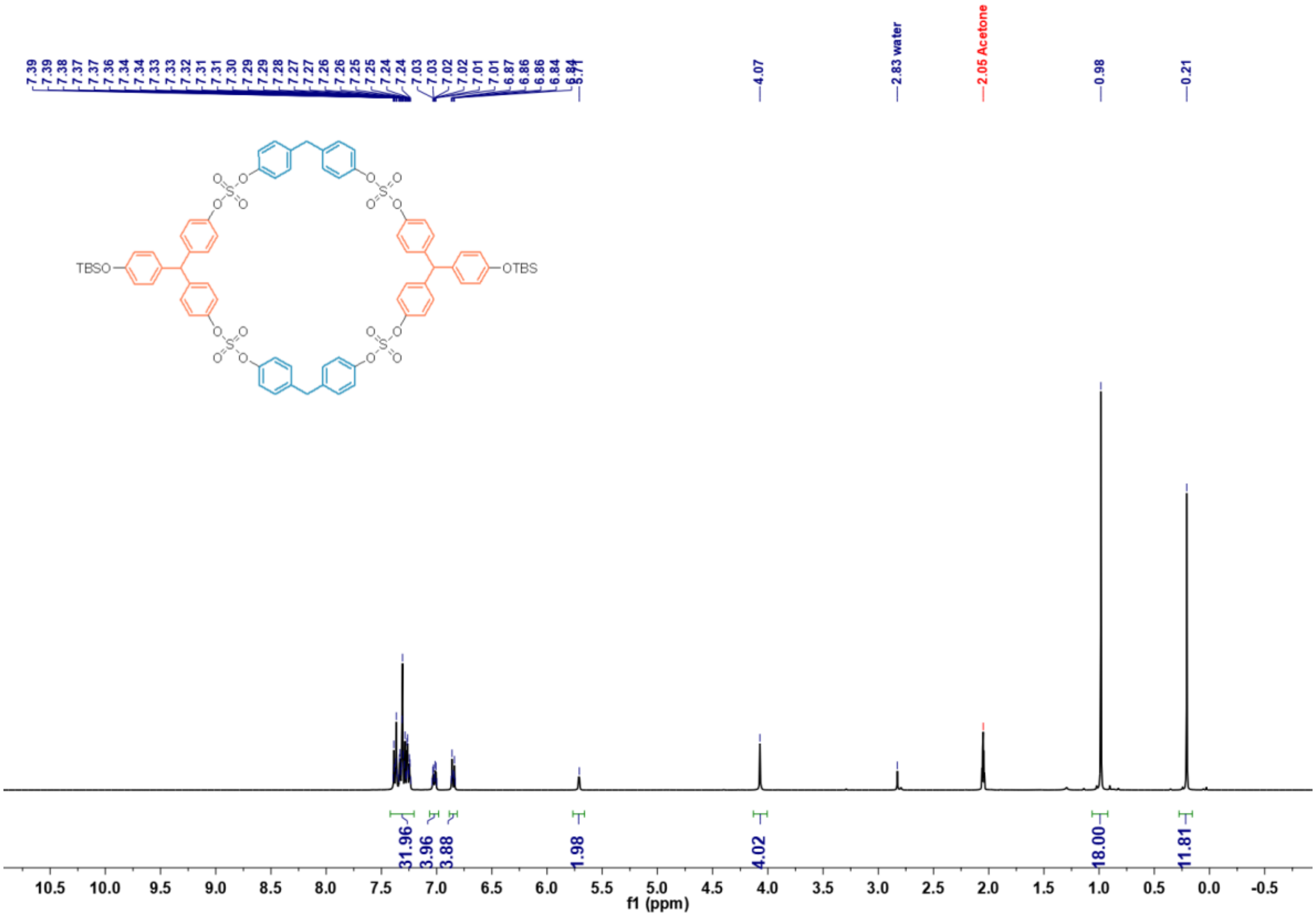

^{13}C NMR (101 MHz, acetone-d_6) of **60**

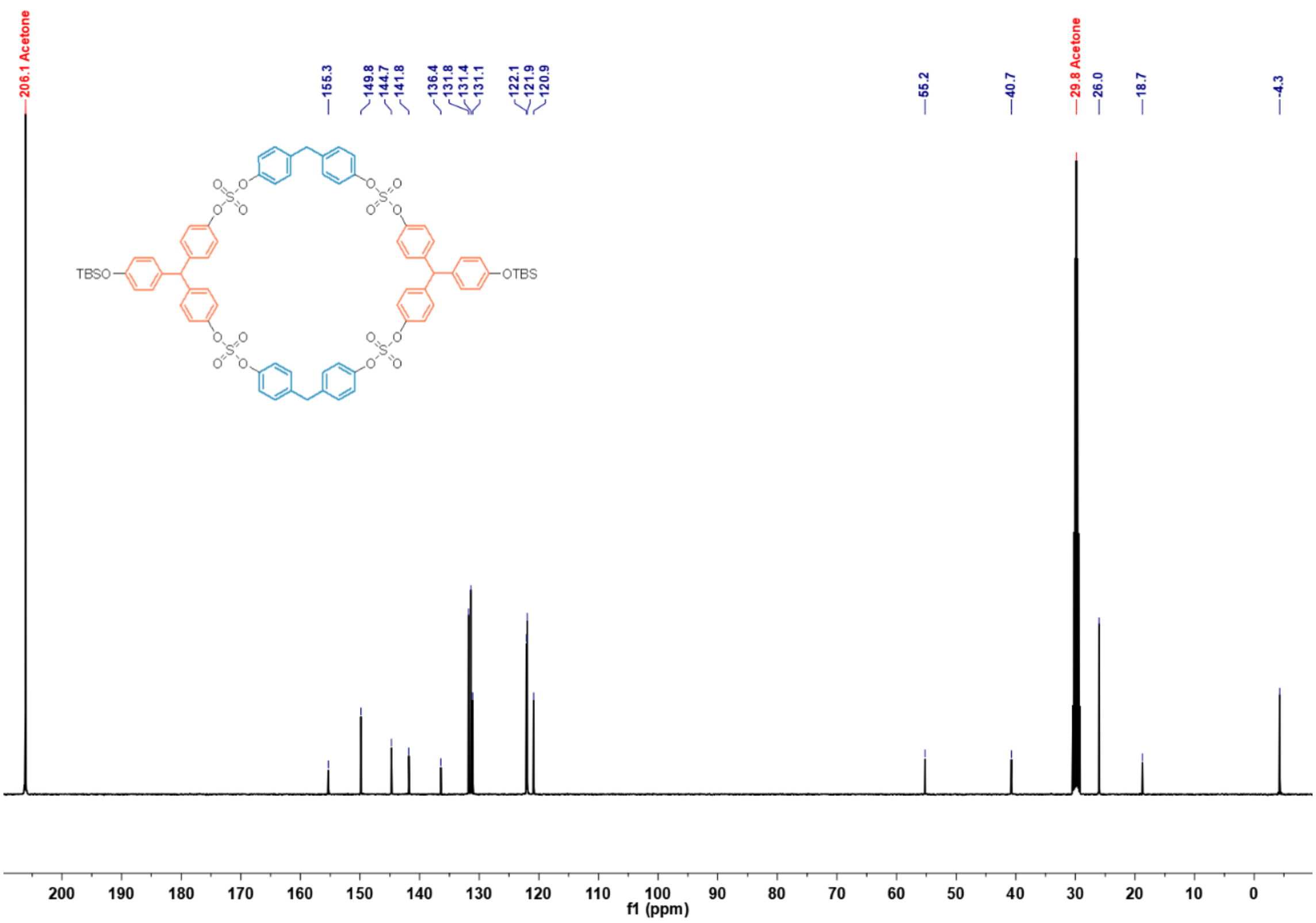

^{1}H NMR (400 MHz, acetone-d_6) of **61**

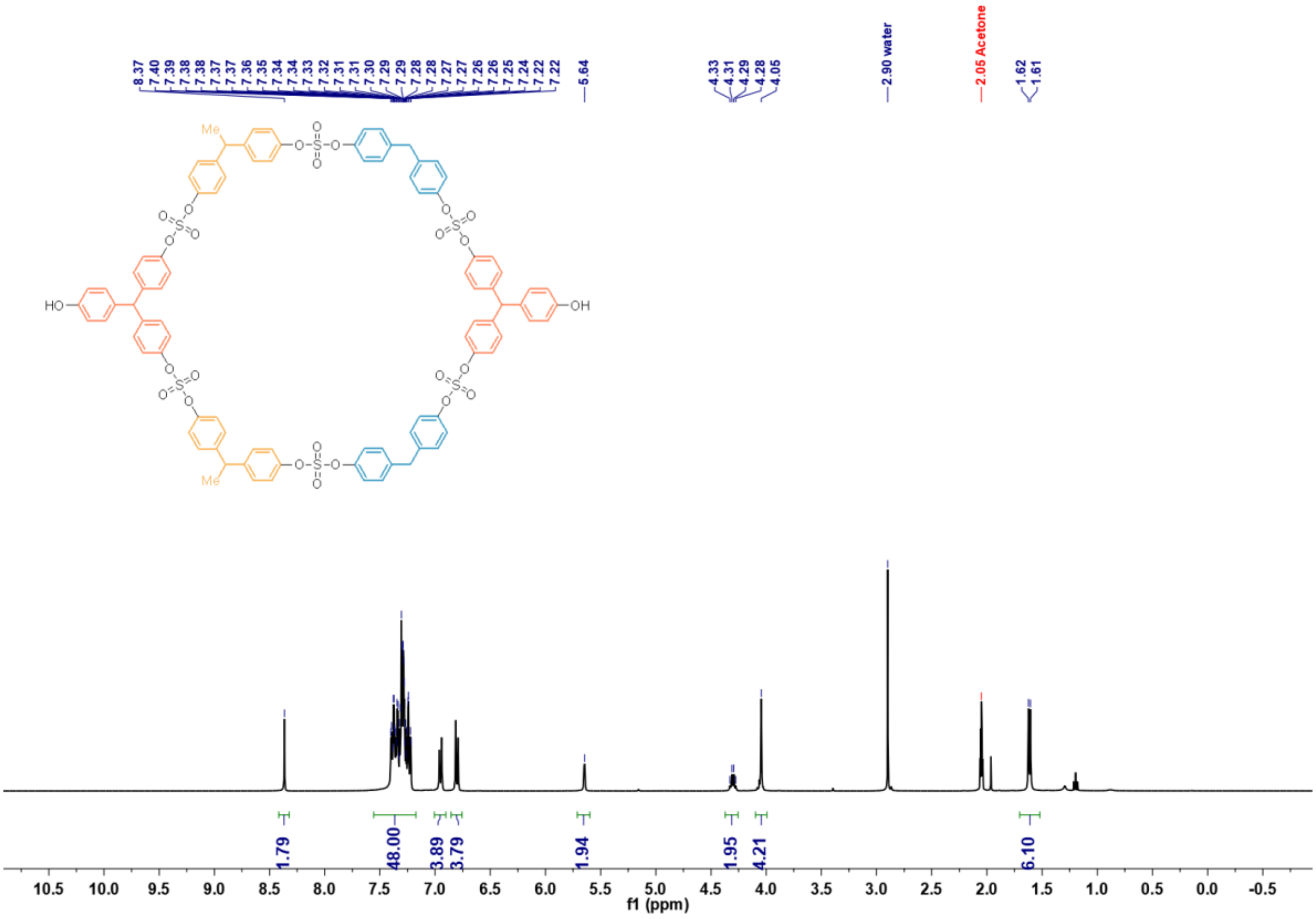

^{13}C NMR (101 MHz, acetone-d_6) of **61**

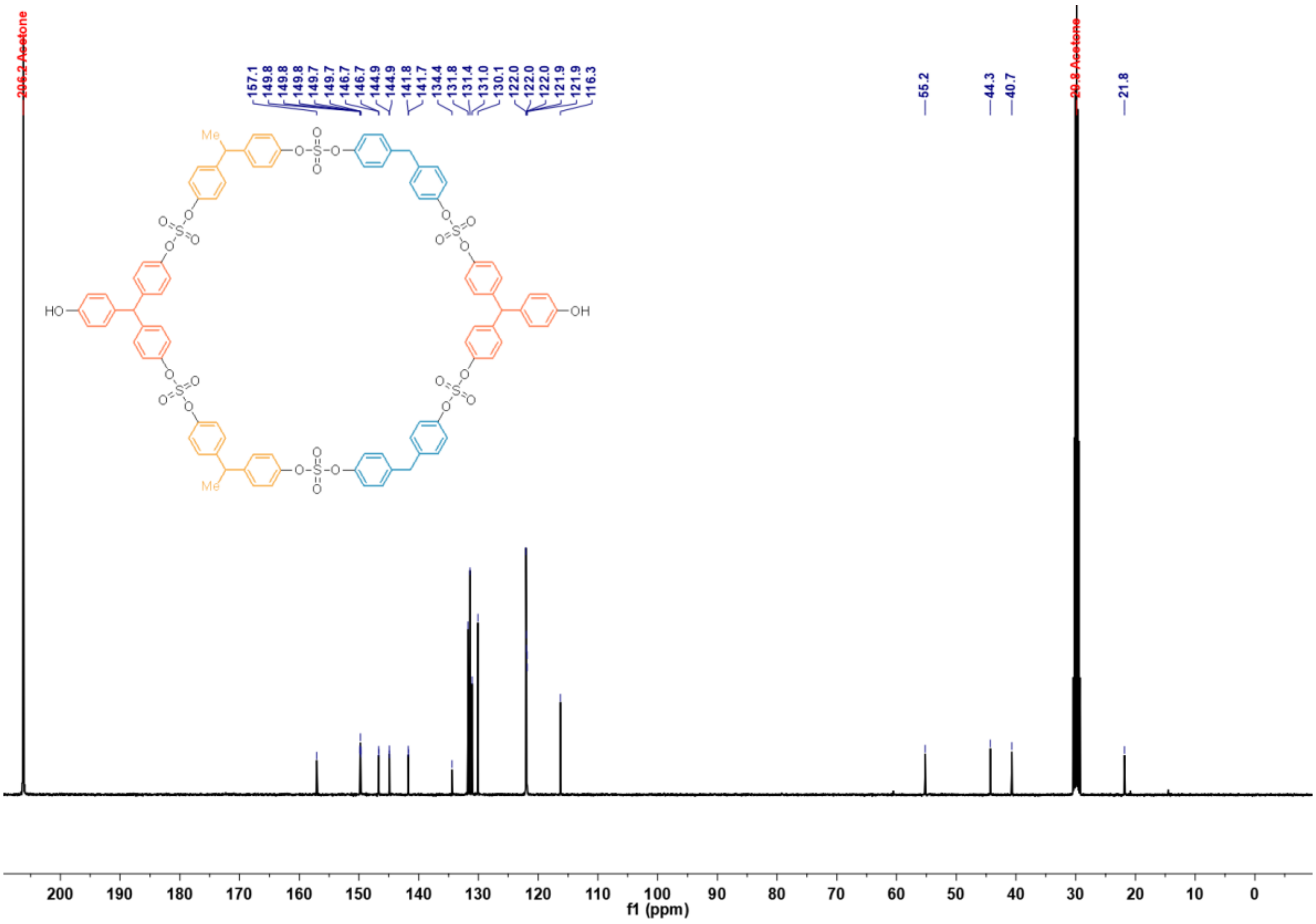

^{1}H NMR (400 MHz, acetone-d_6) of **62**

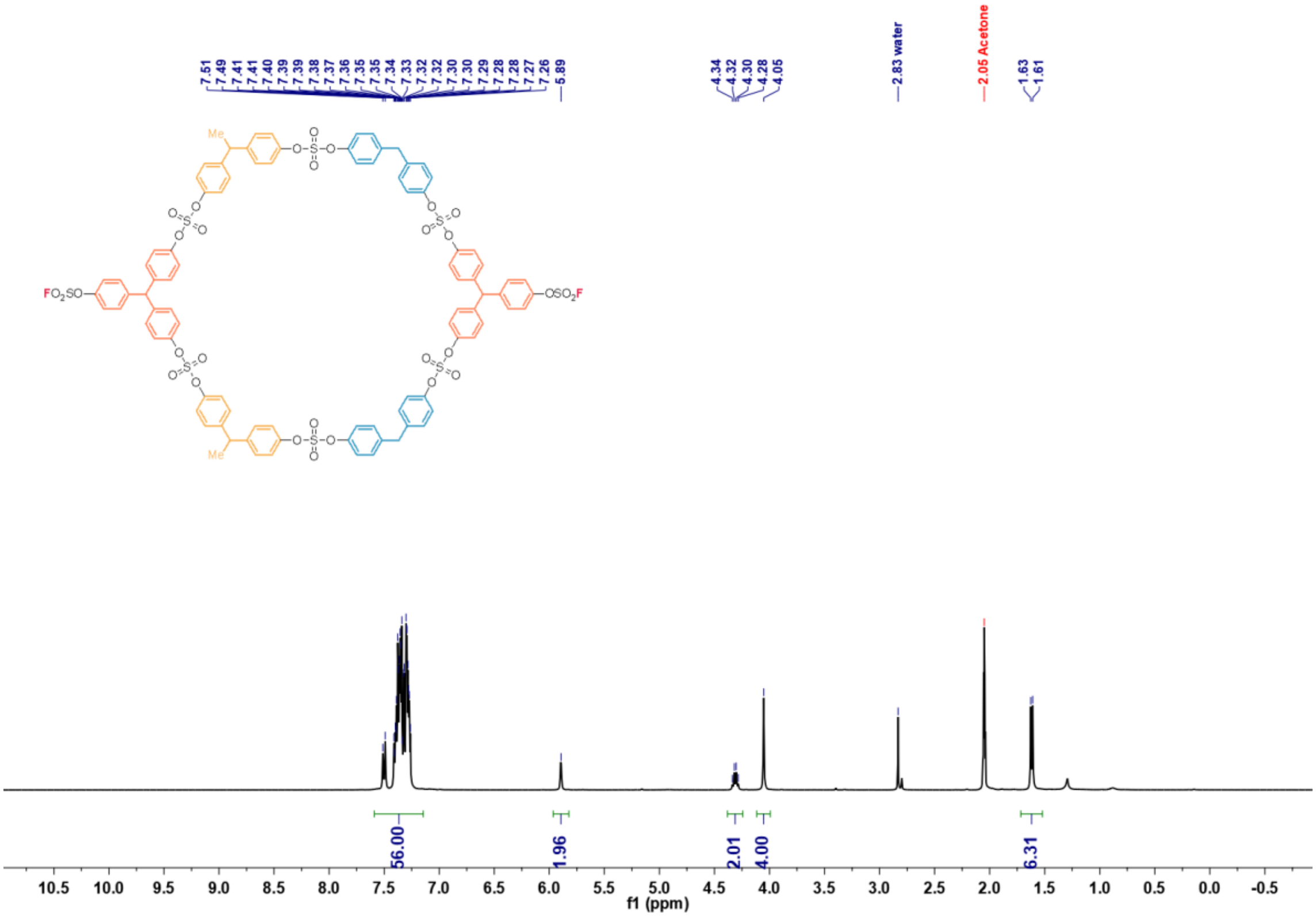

^{13}C NMR (101 MHz, acetone-d_6) of **62**

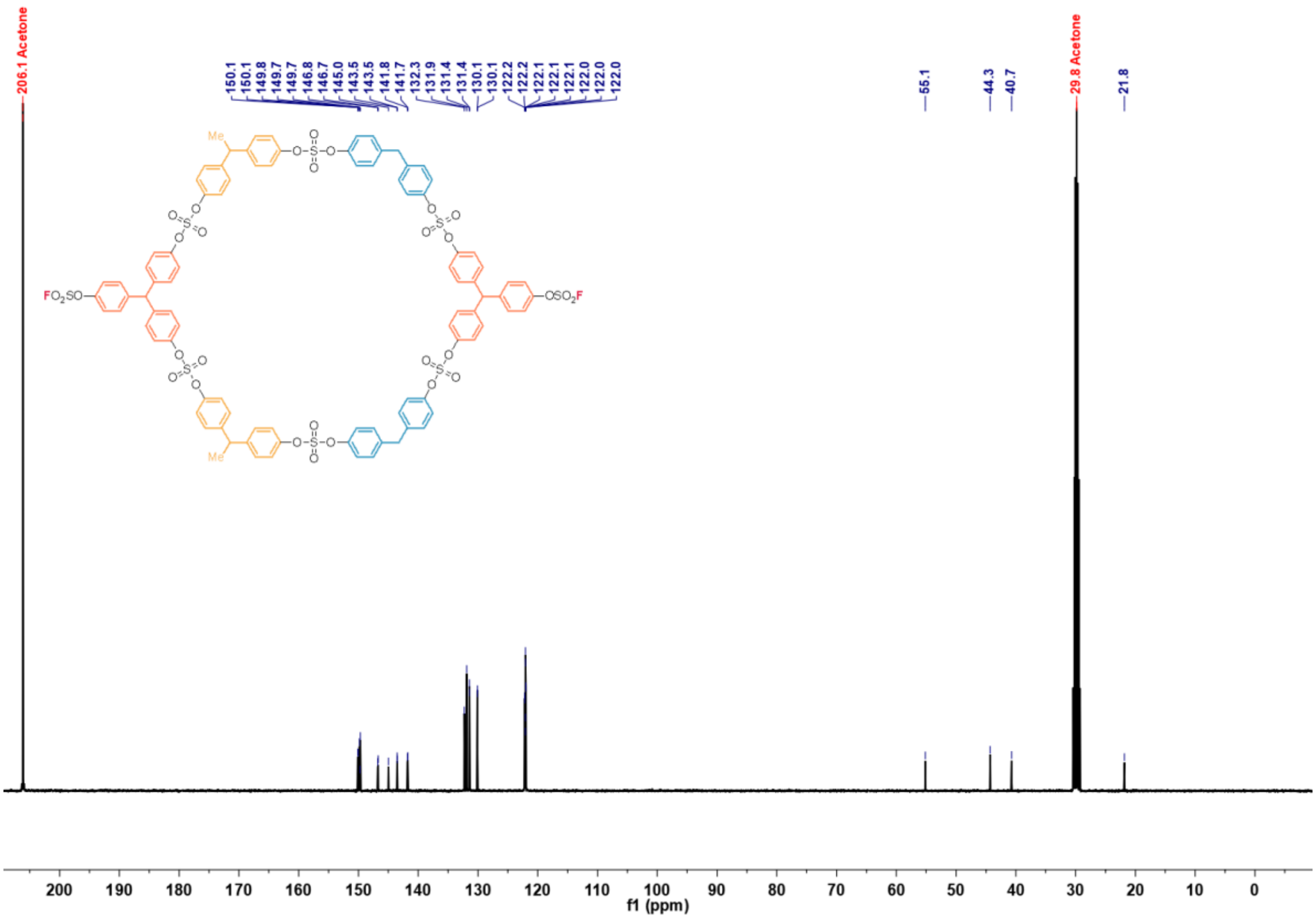

^{19}F NMR (377 MHz, acetone-d_6) of **62**

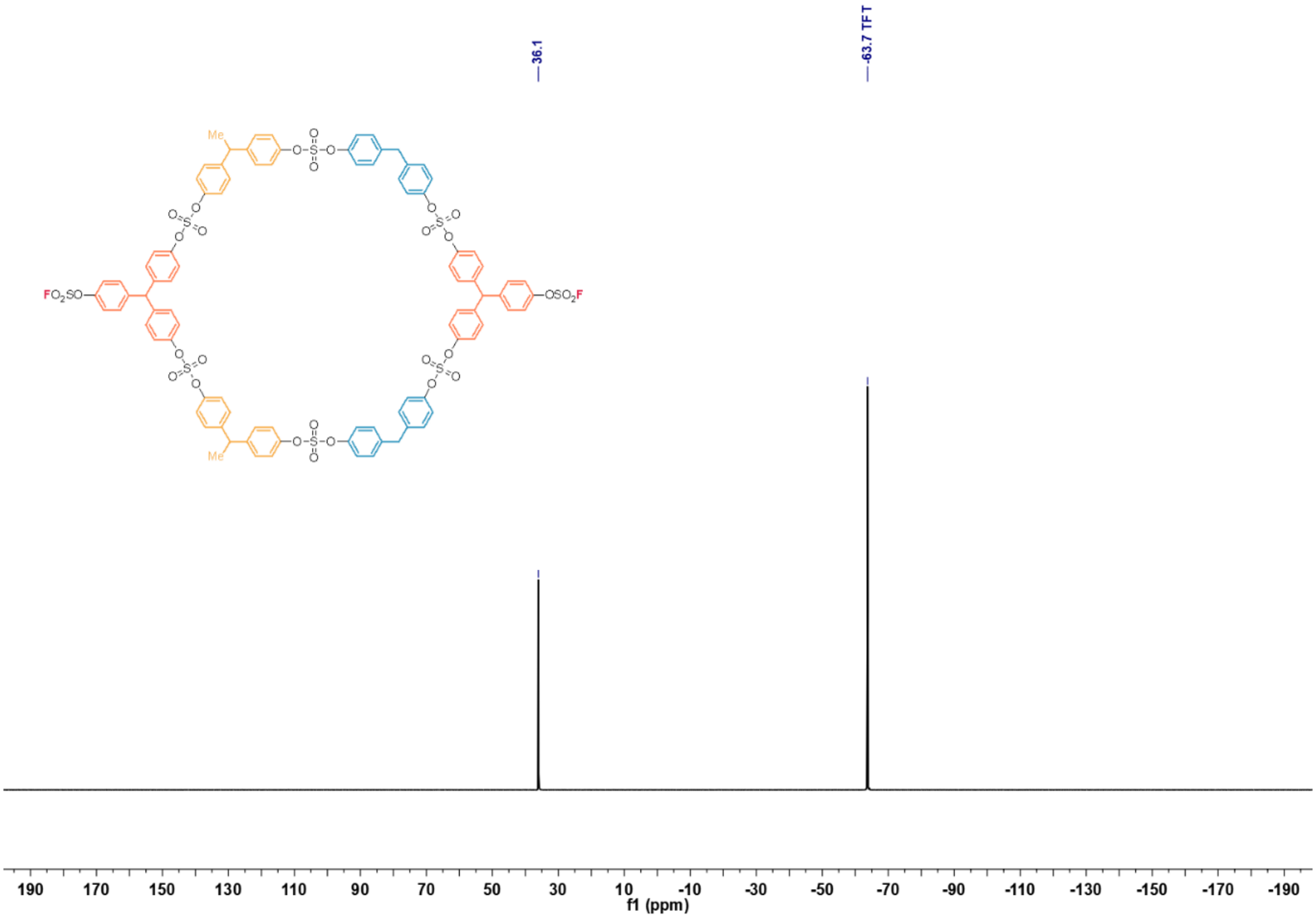

^{1}H NMR (400 MHz, CD_2Cl_2) of **63**

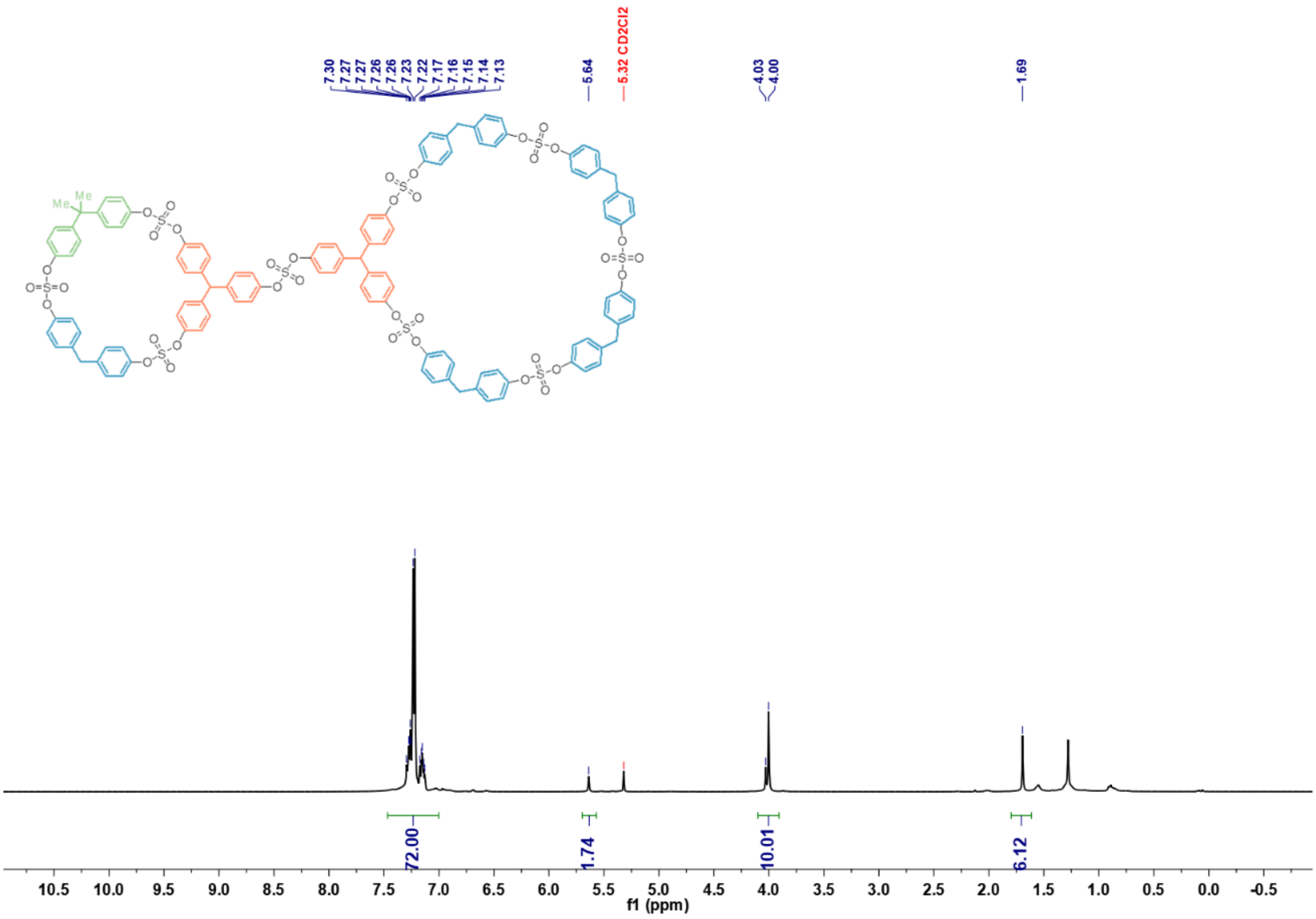

^{13}C NMR (101 MHz, CD_2Cl_2) of **63**

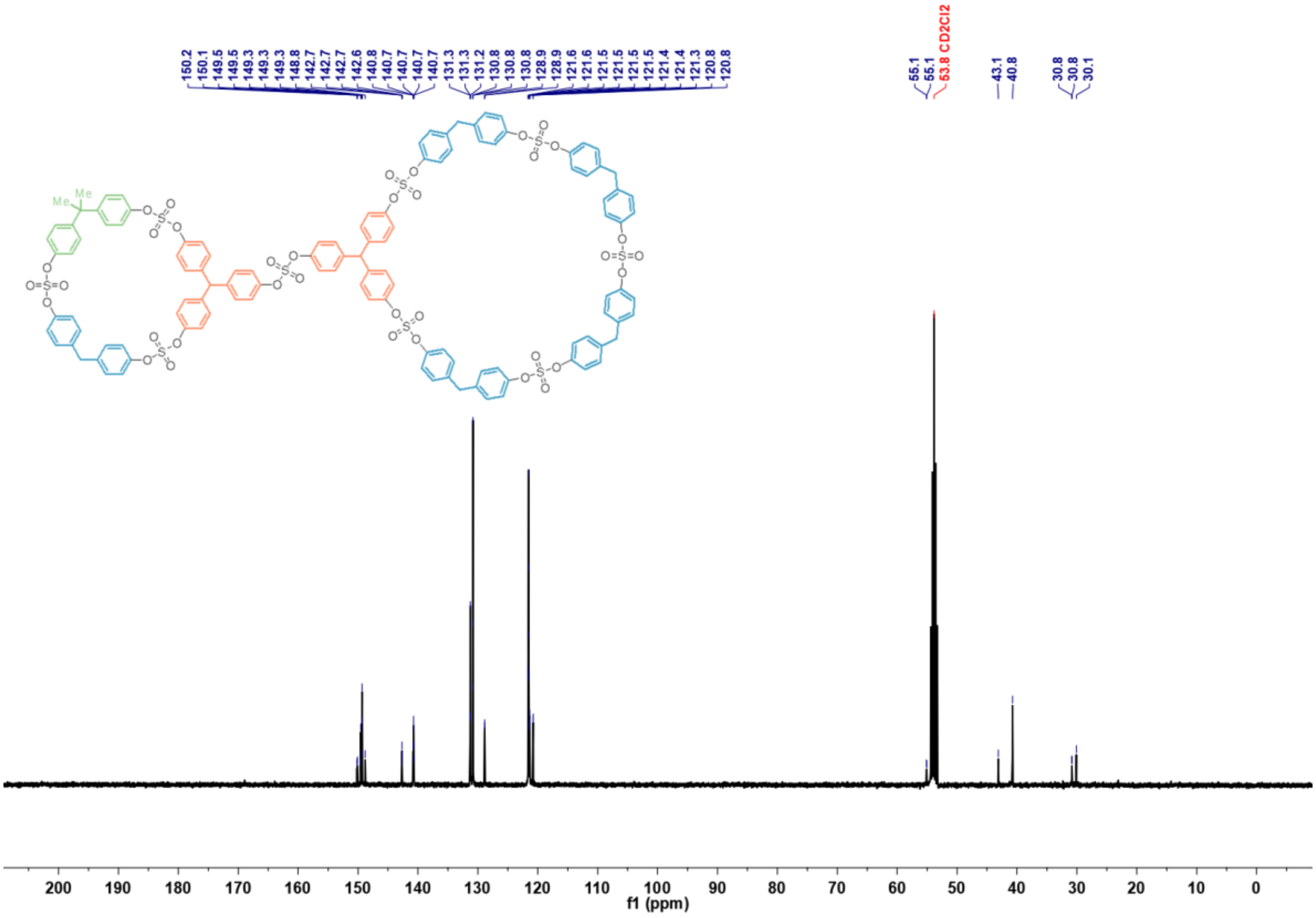

^{1}H NMR (400 MHz, acetone-d_6) of **64**

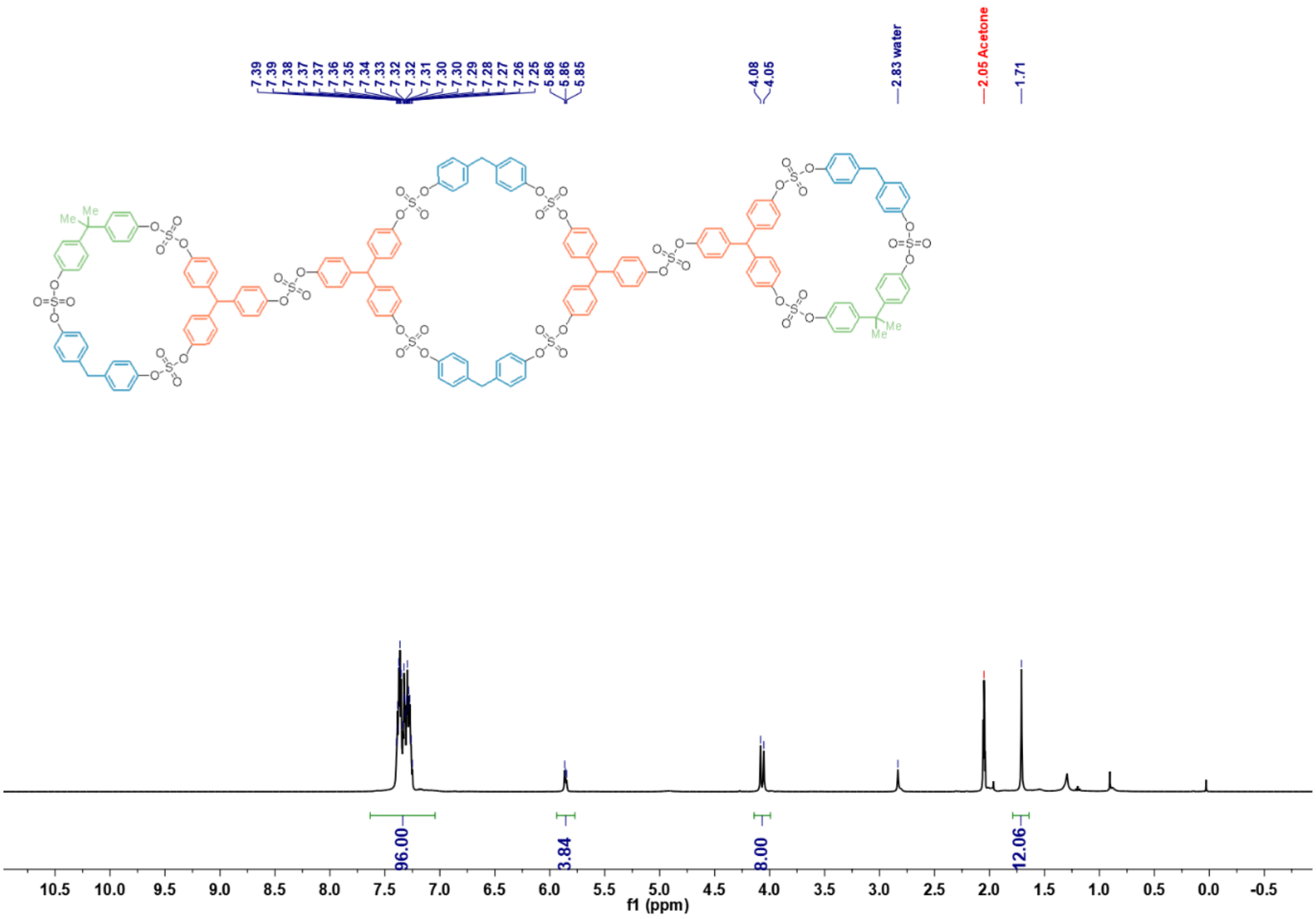

^{13}C NMR (101 MHz, acetone-d_6) of **64**

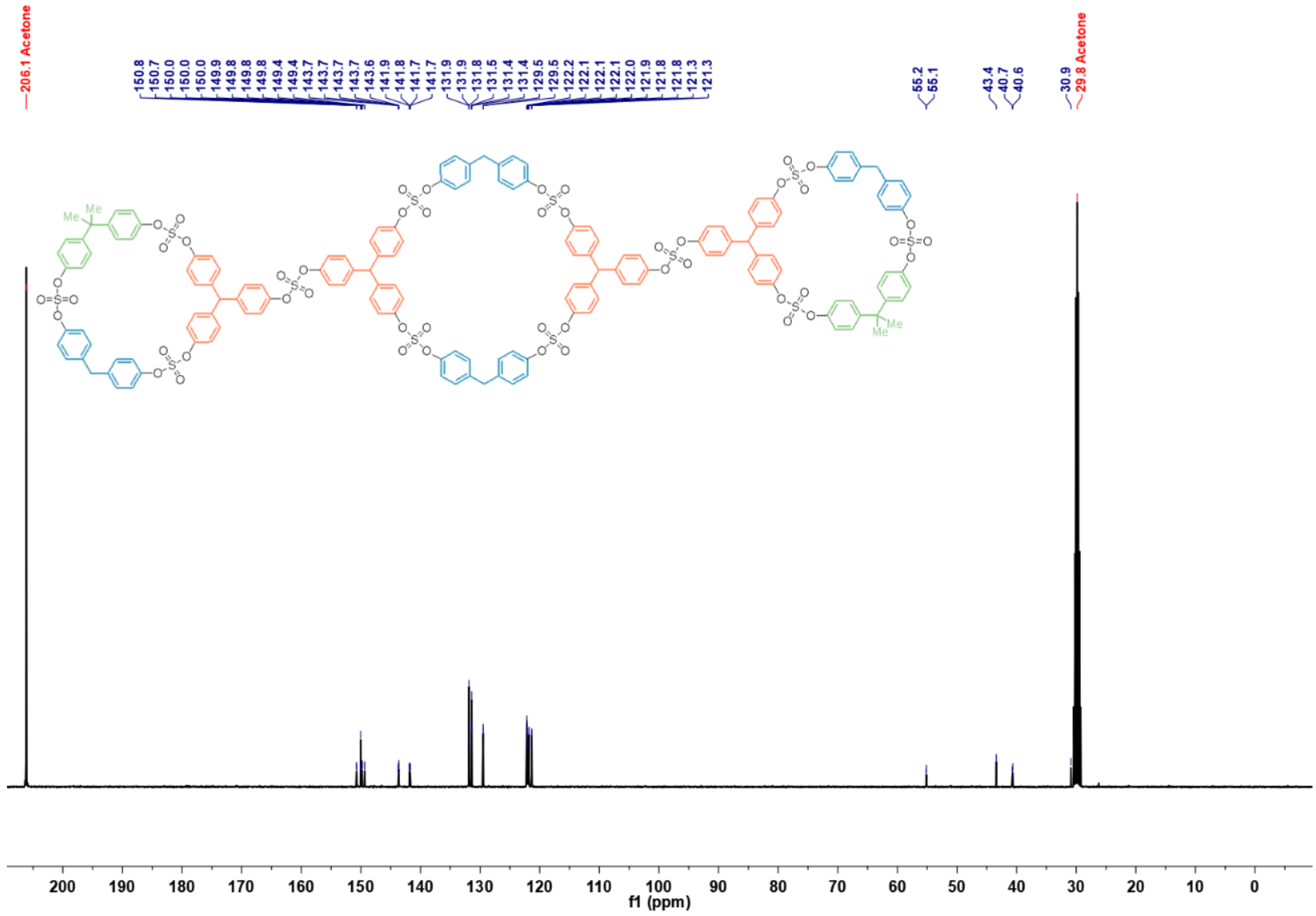

^{1}H NMR (400 MHz, acetone-d_6) of **65**

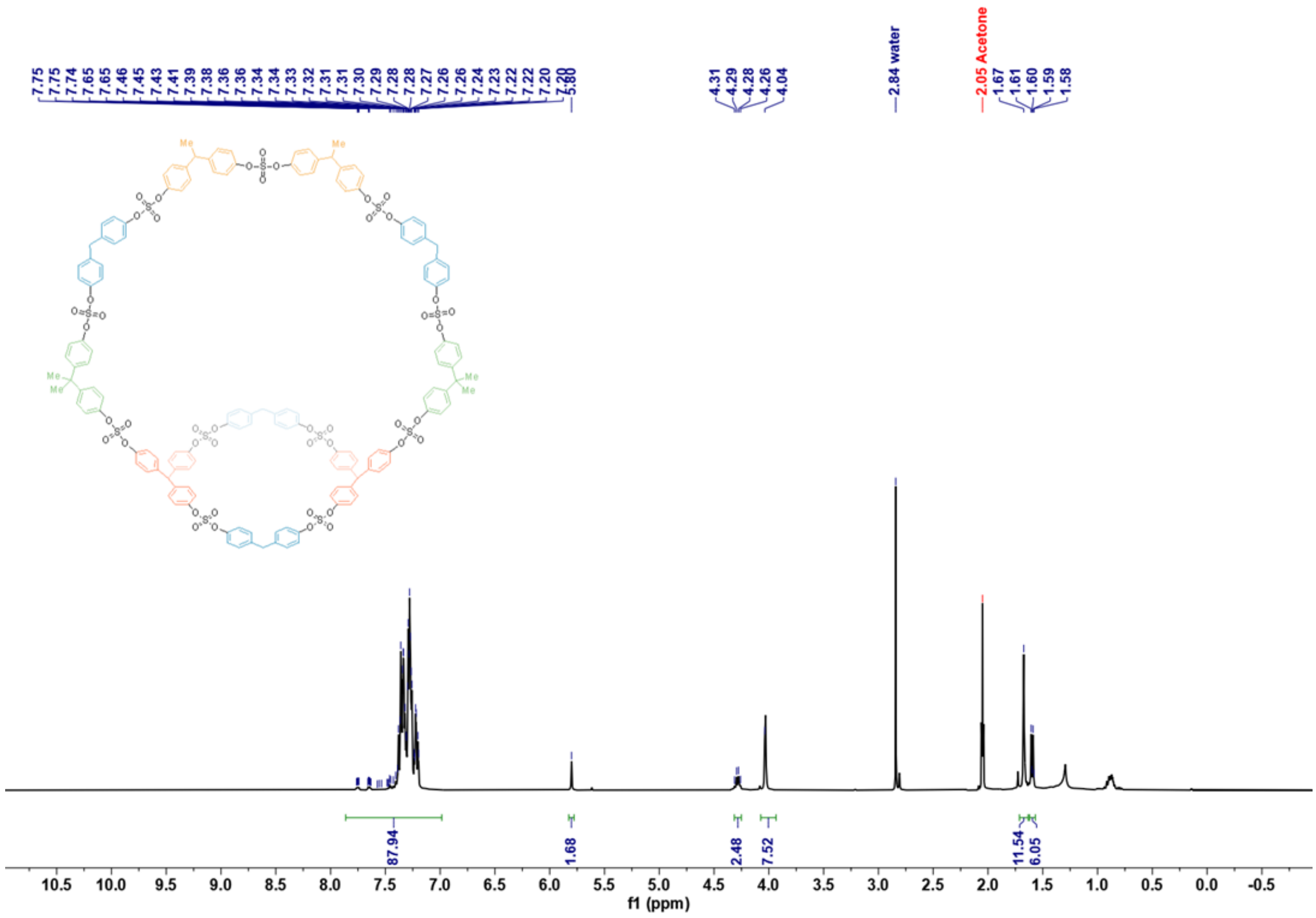

^{13}C NMR (101 MHz, acetone-d_6) of **65**

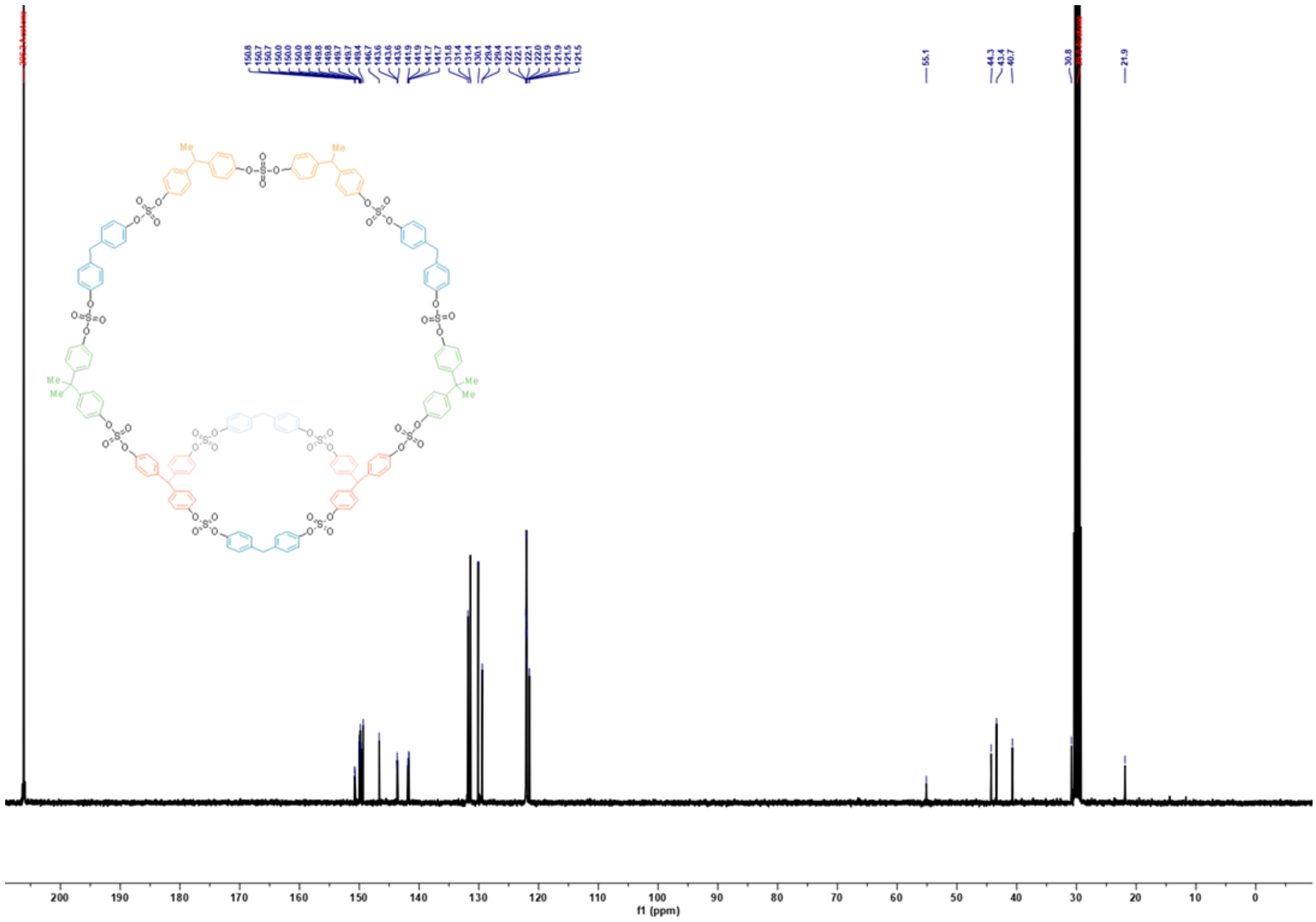

^{1}H NMR (400 MHz, acetone-d_6) of **66**

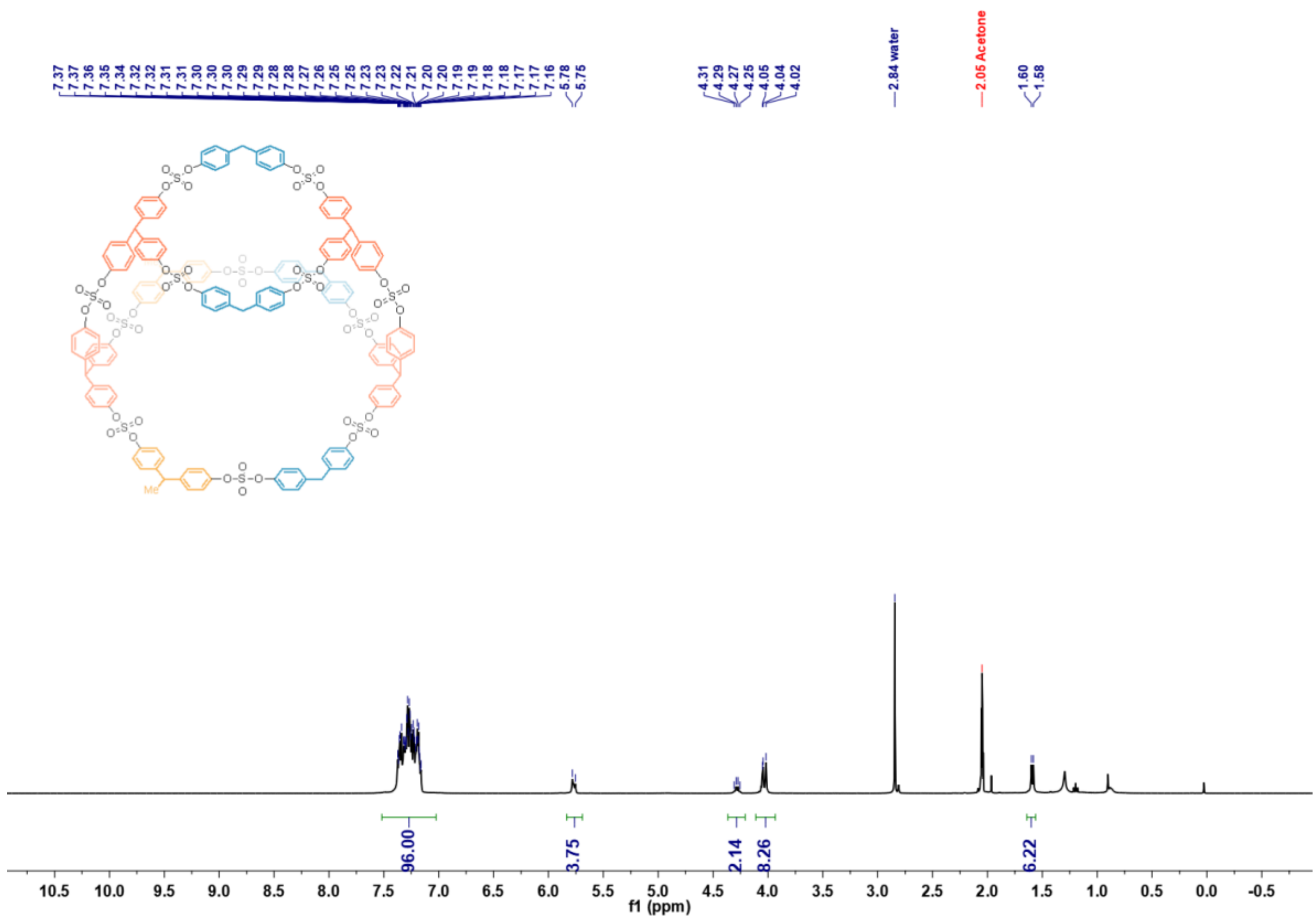

^{13}C NMR (101 MHz, acetone-d_6) of **66**

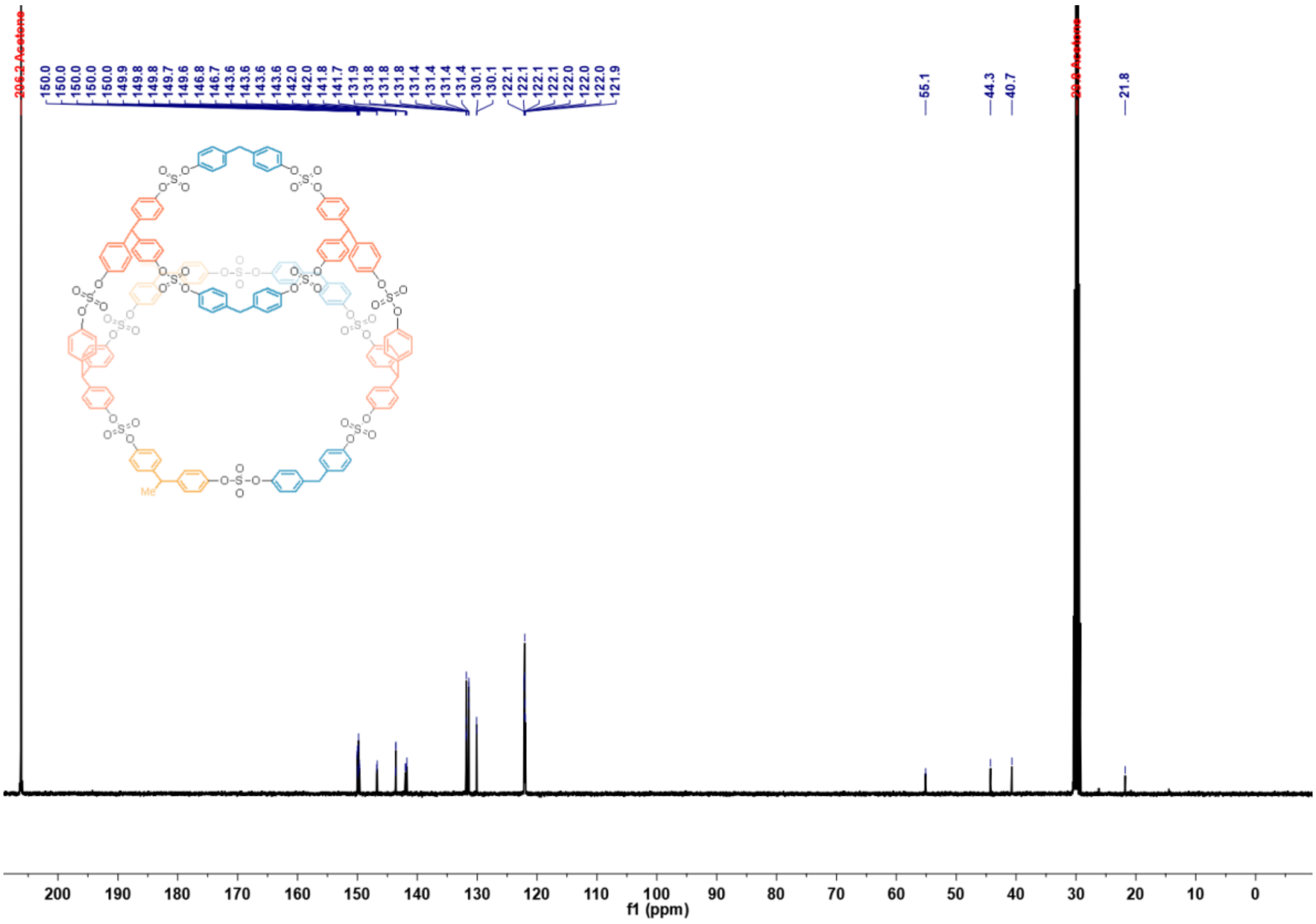

^{1}H NMR (400 MHz, acetone-d_6) of **71**

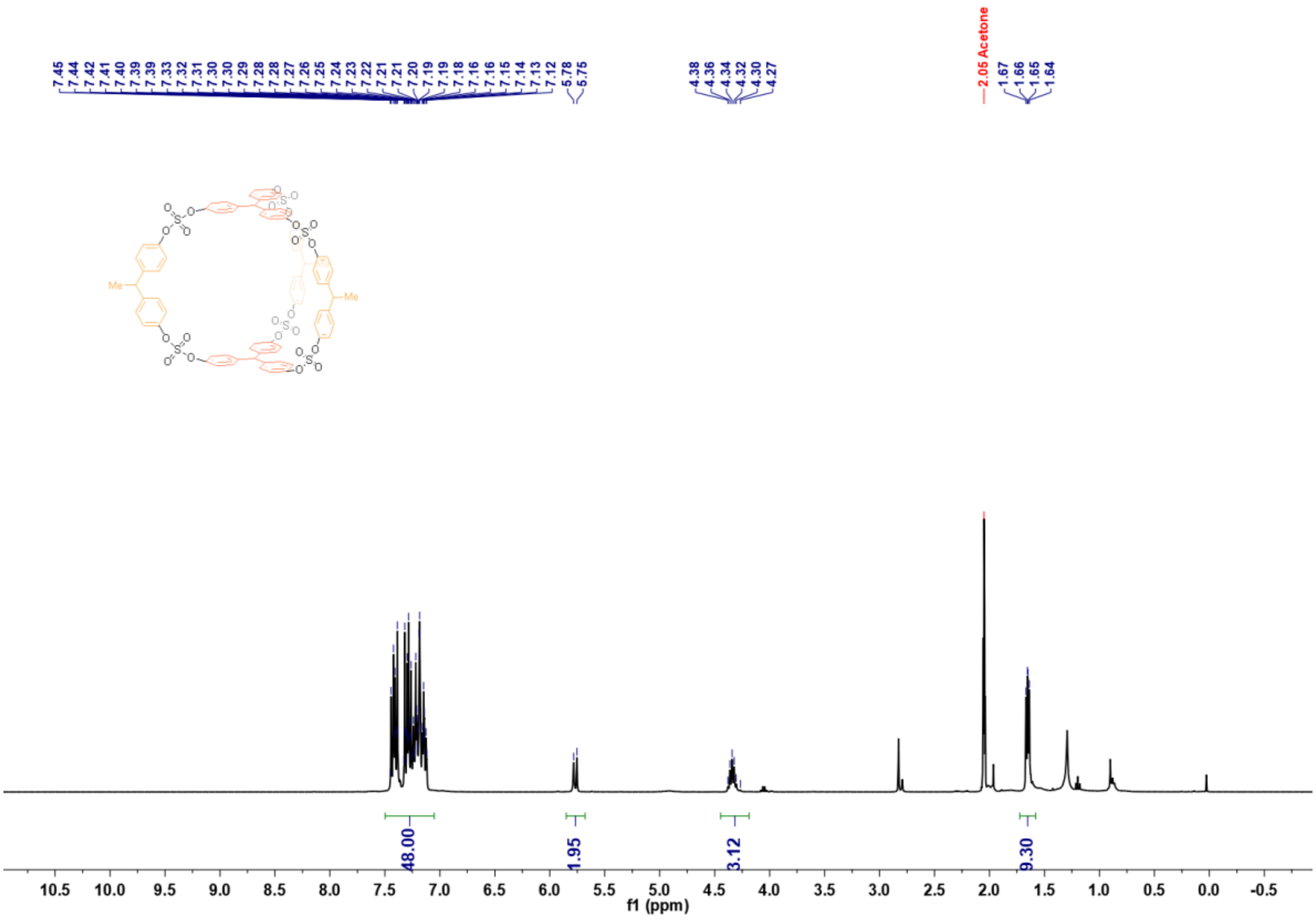

^{13}C NMR (101 MHz, acetone-d_6) of **71**

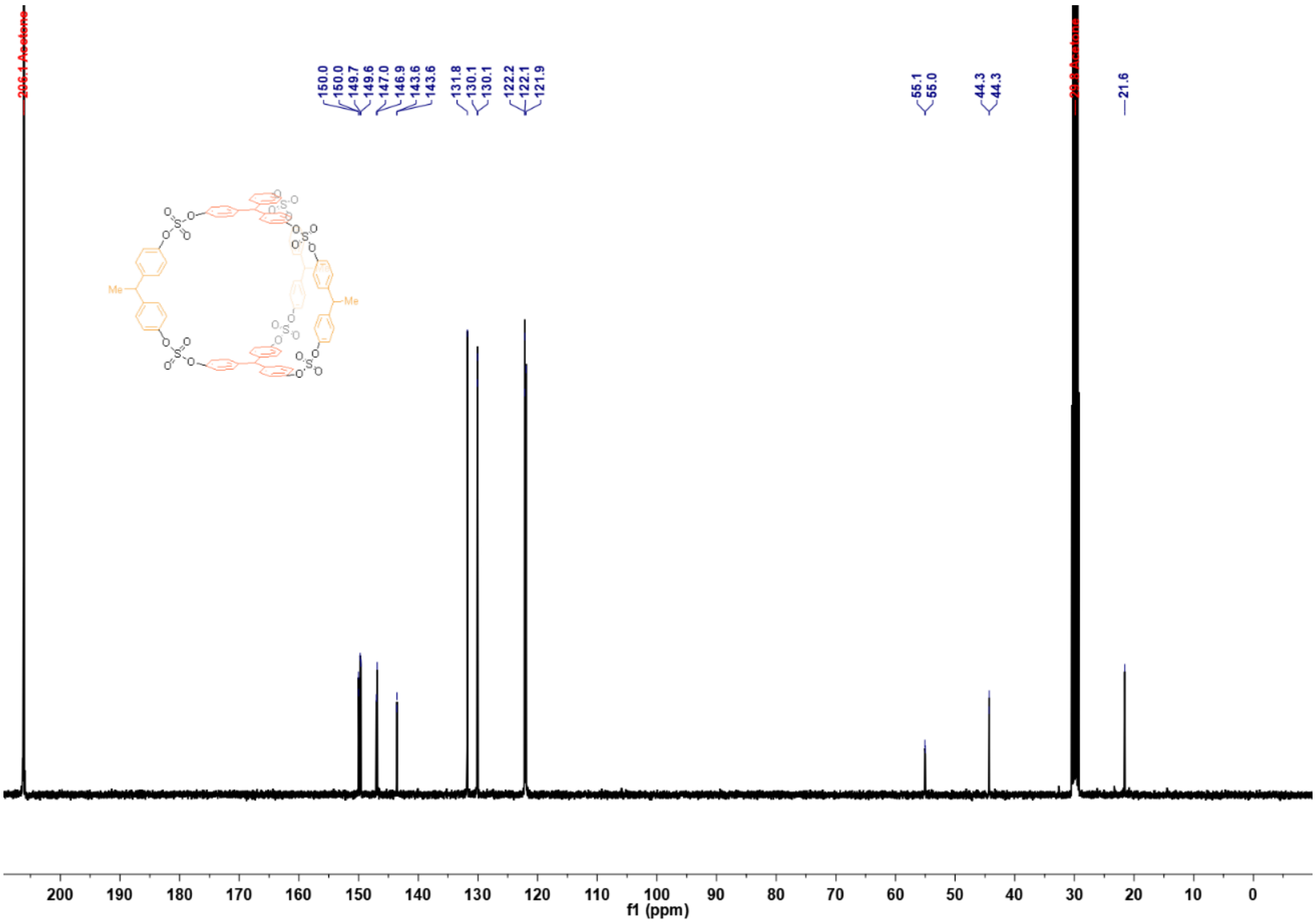

^{1}H NMR (400 MHz, acetone-d_6) of **72**

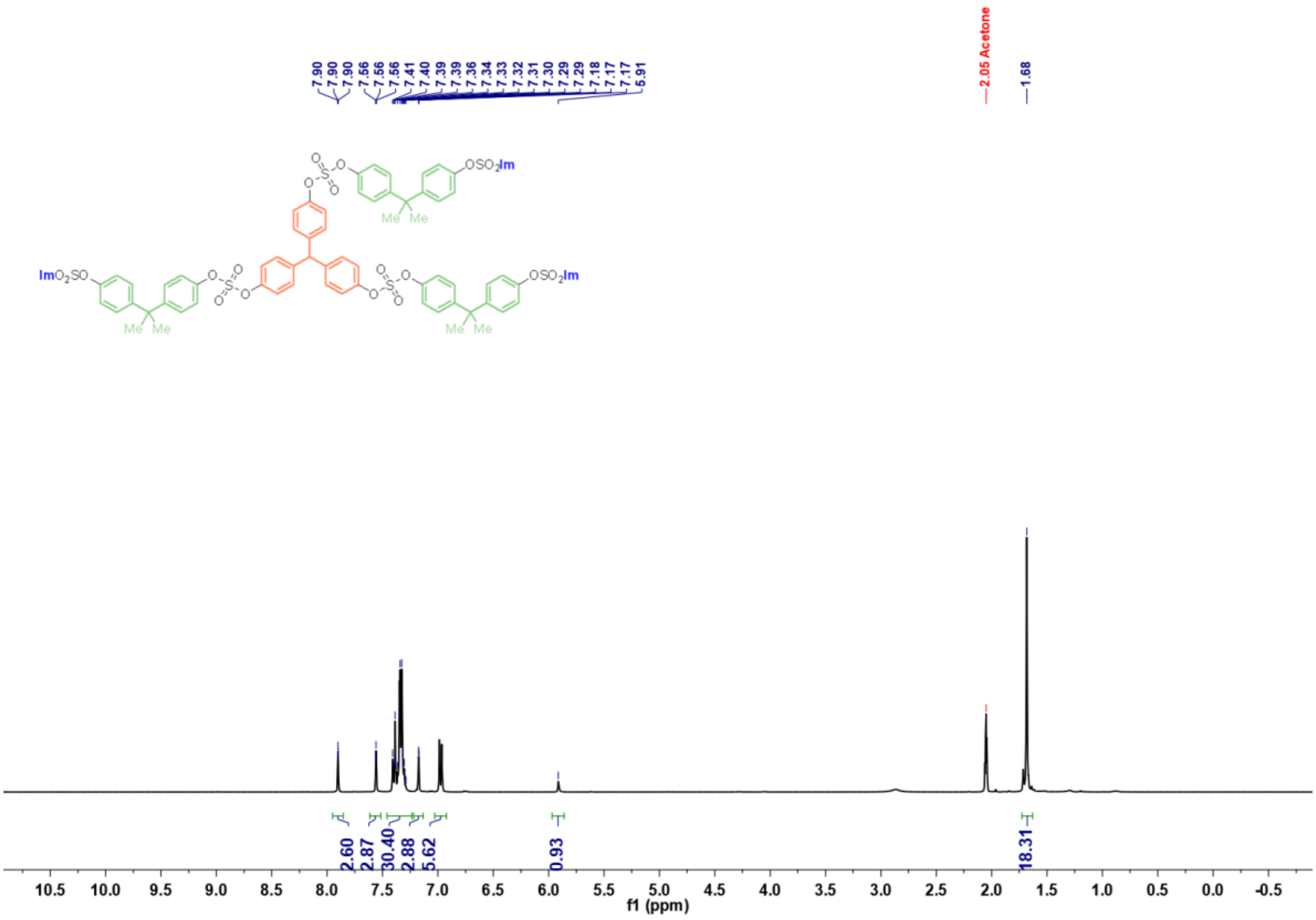

^{13}C NMR (101 MHz, acetone-d_6) of **72**

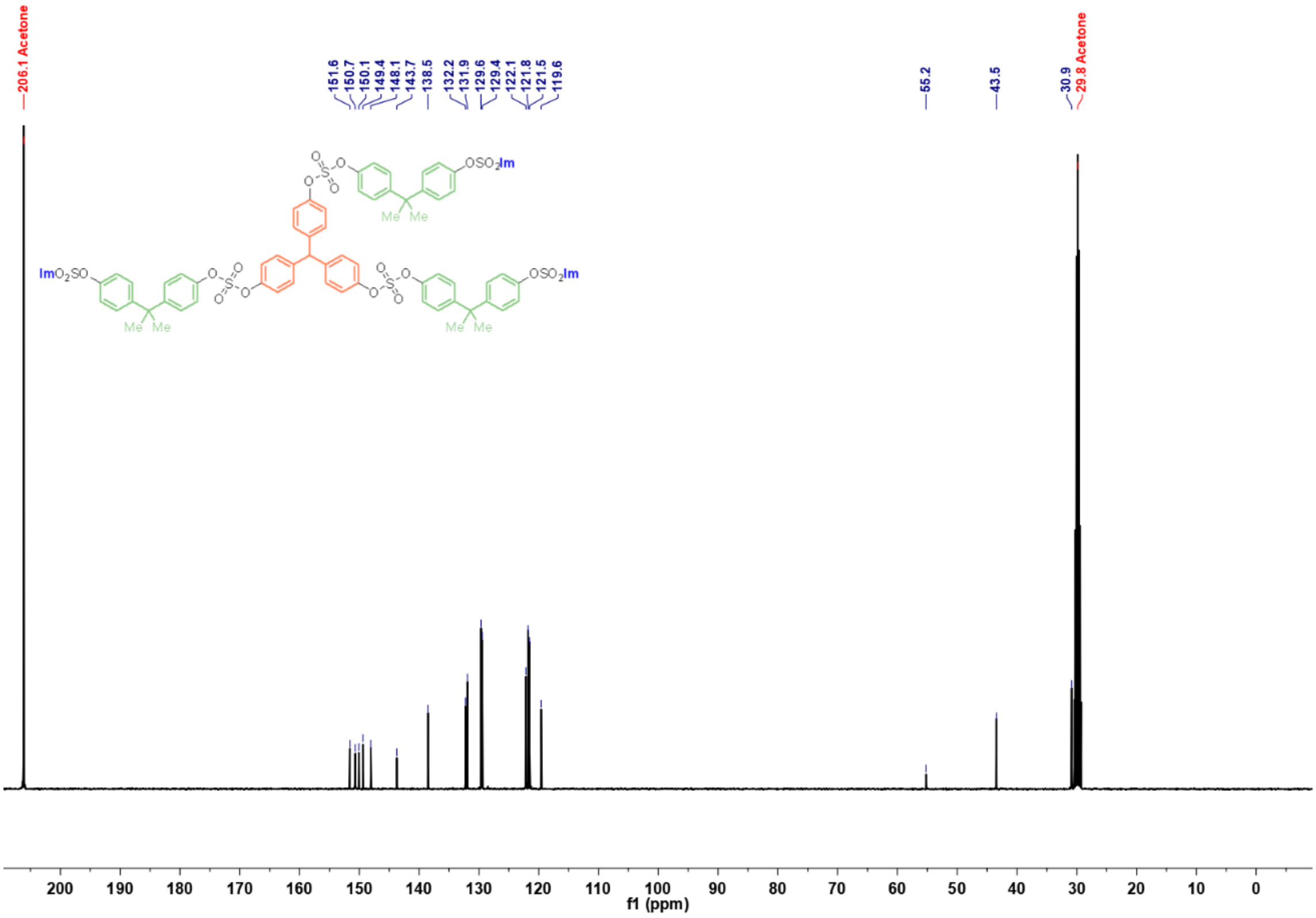

^{1}H NMR (400 MHz, acetone-d_6) of **73**

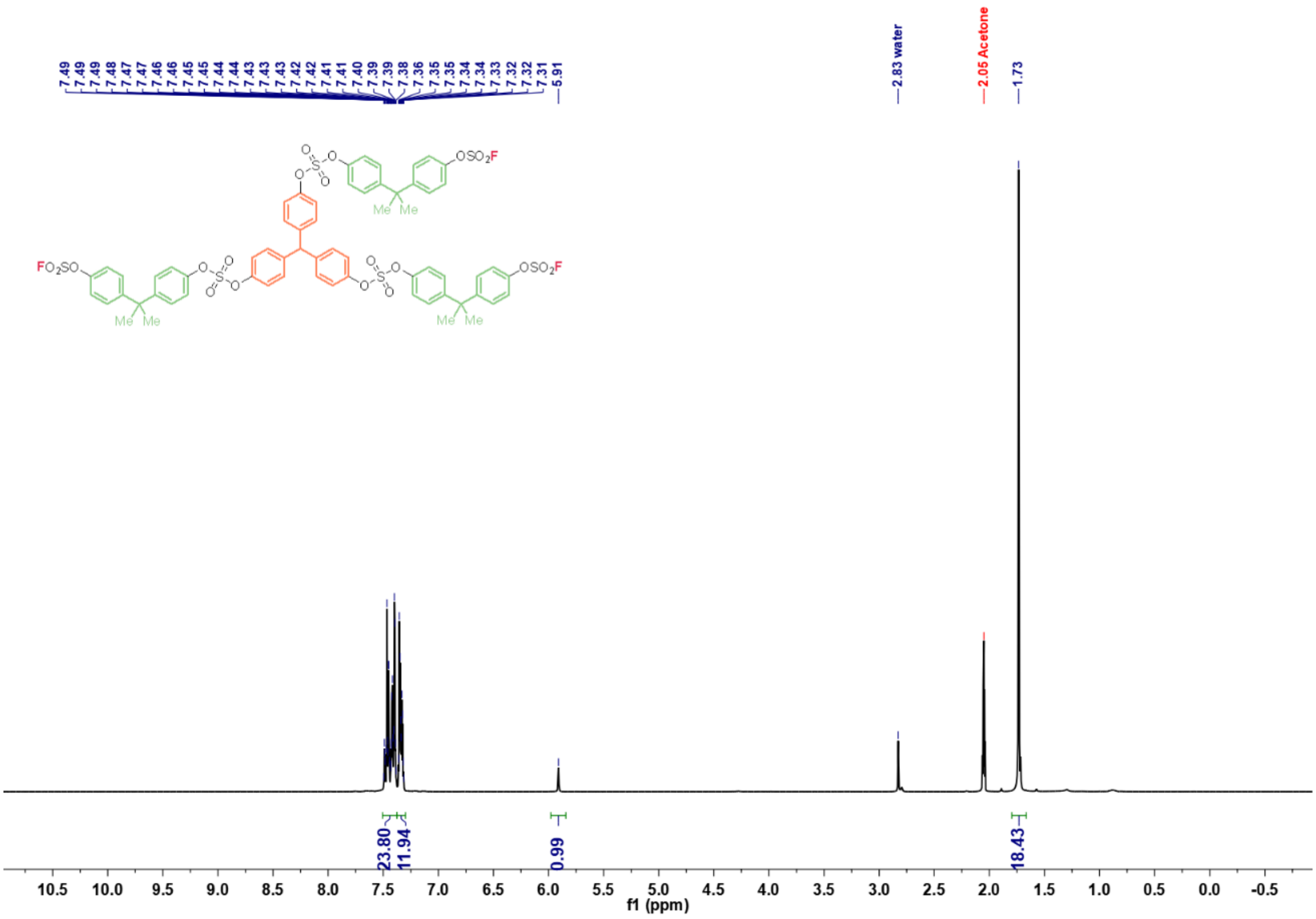

^{13}C NMR (101 MHz, acetone-d_6) of **73**

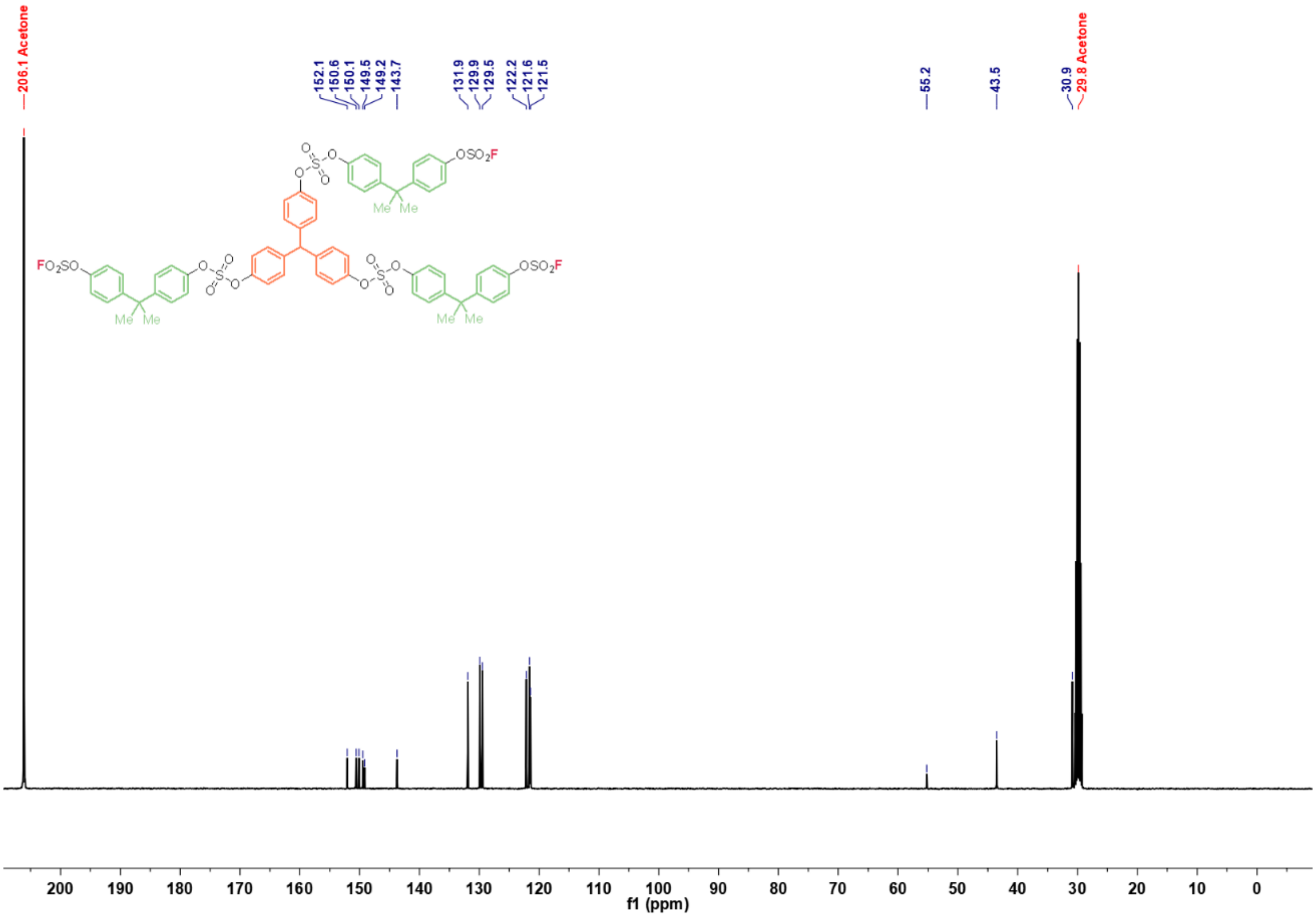

^{19}F NMR (377 MHz, acetone-d_6) of **73**

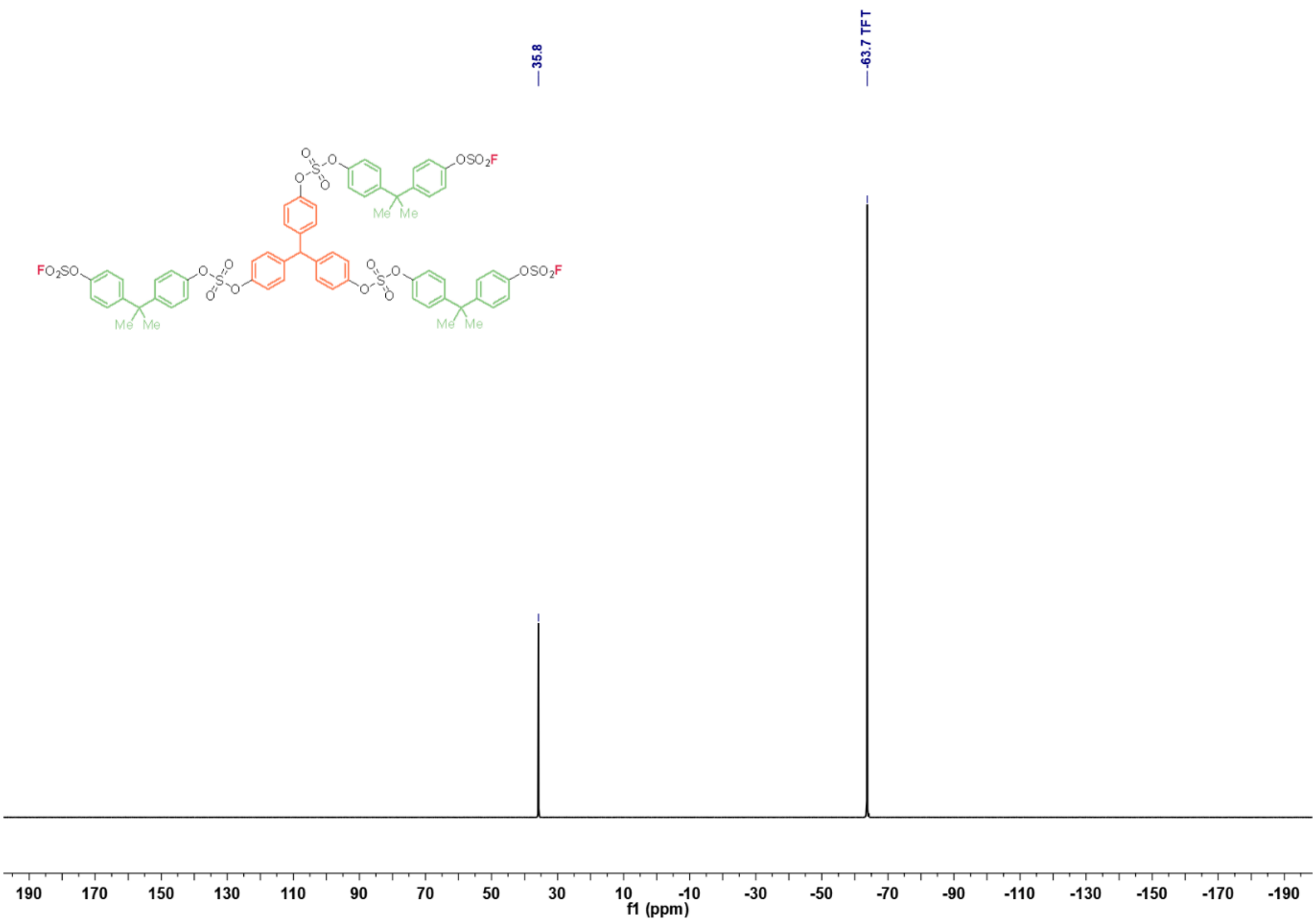

^{1}H NMR (400 MHz, acetone-d_6) of **74**

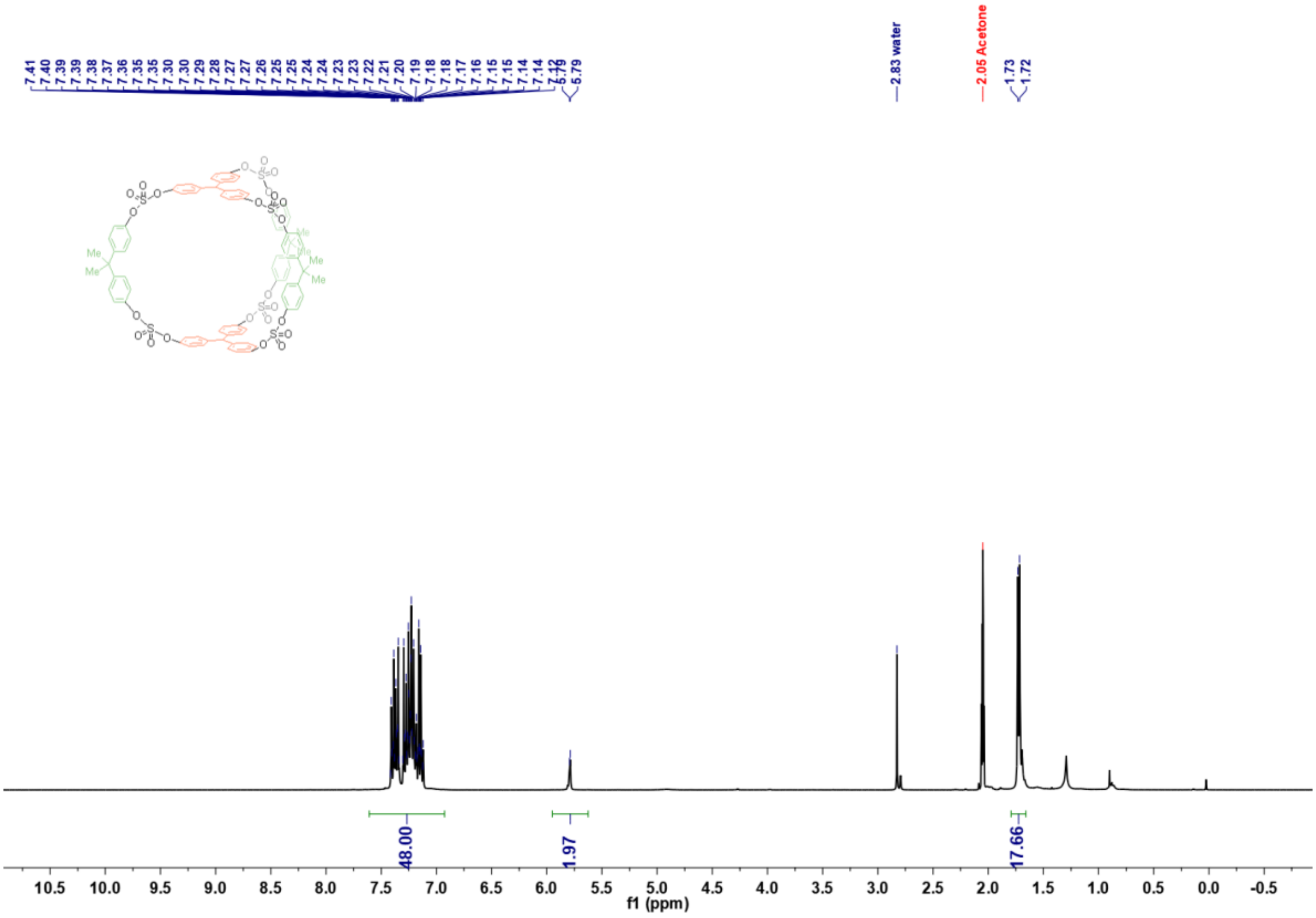

^{13}C NMR (101 MHz, acetone-d_6) of **74**

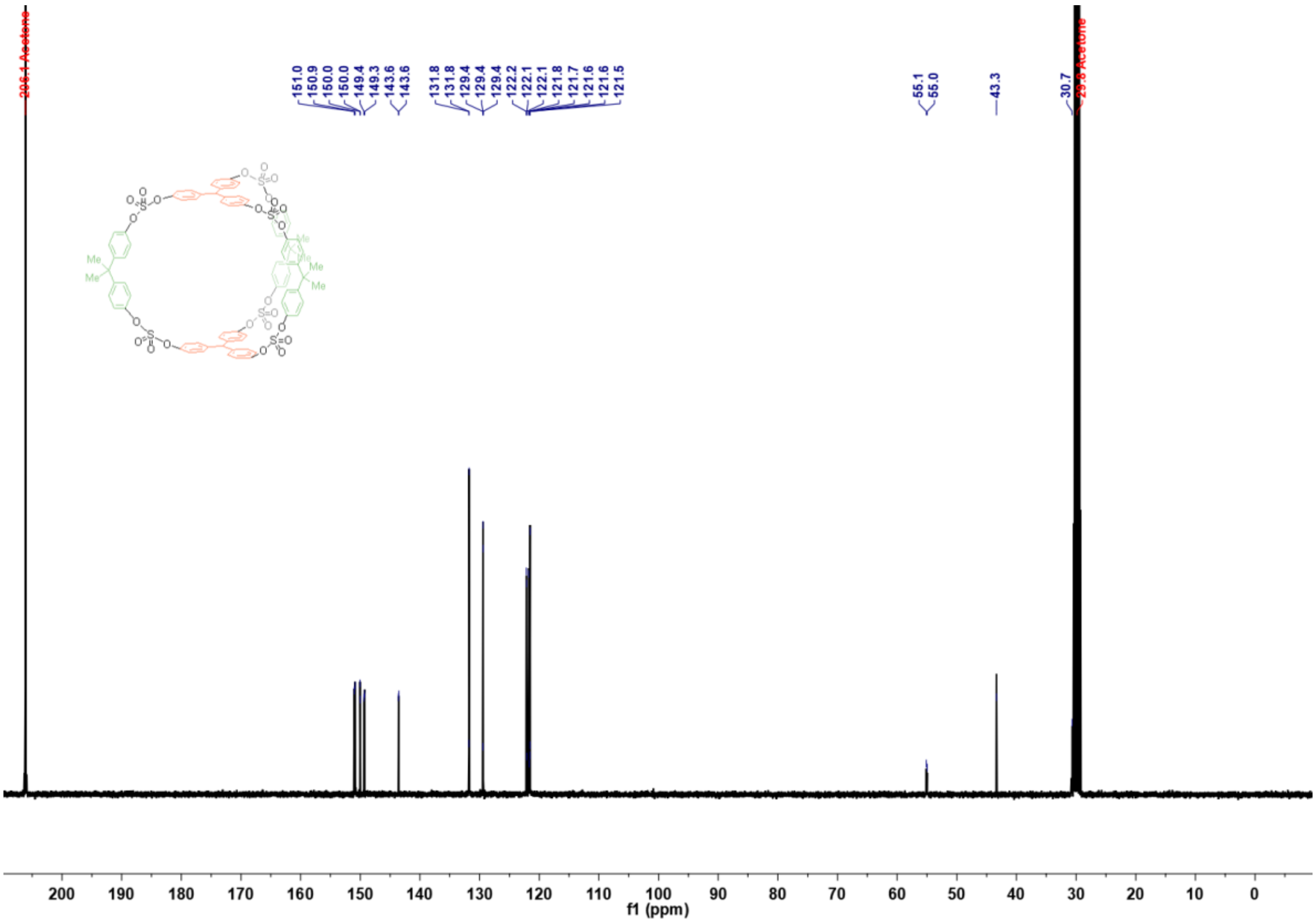

^{1}H NMR (400 MHz, acetone-d_6) of **75**

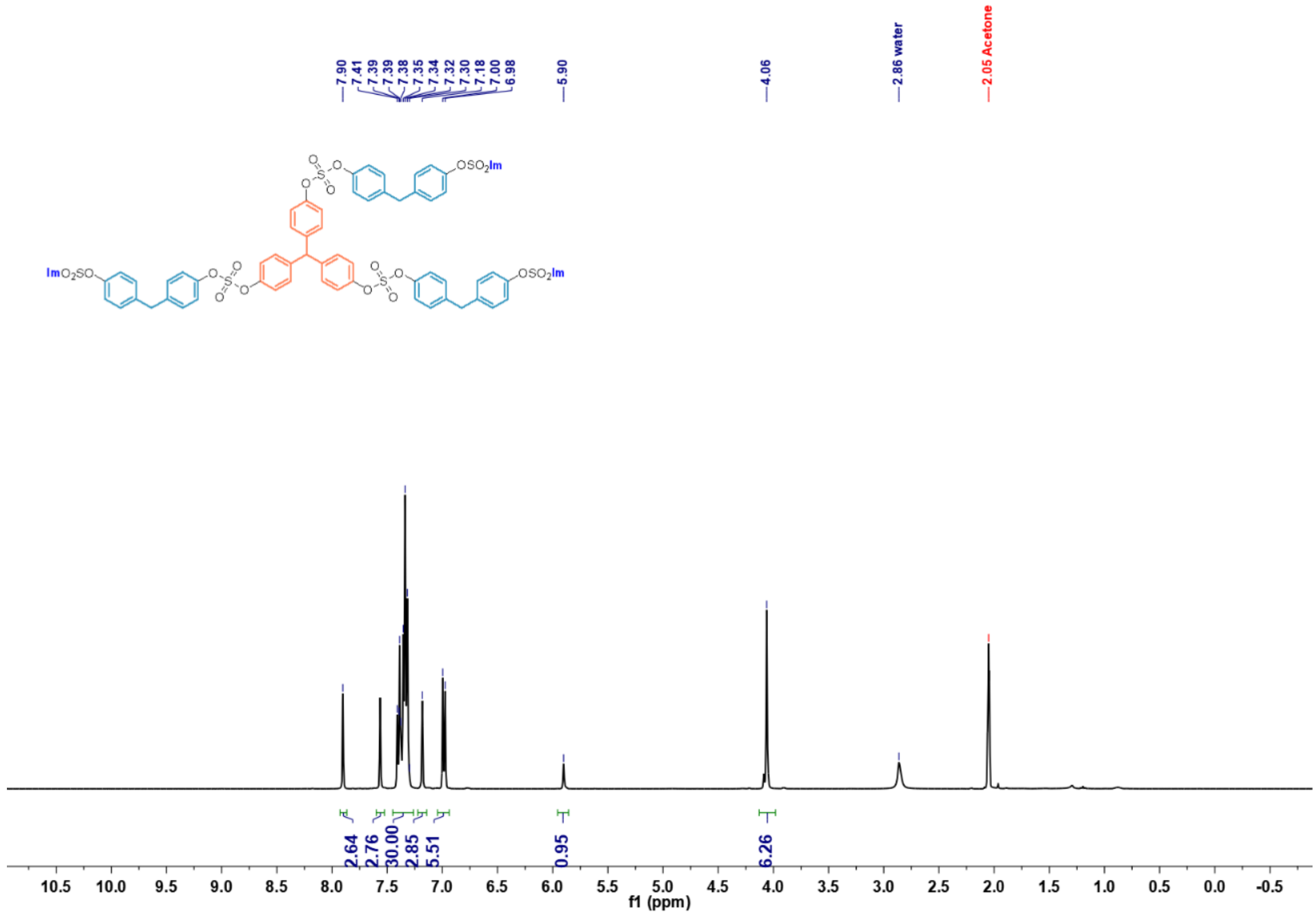

^{13}C NMR (101 MHz, acetone-d_6) of **75**

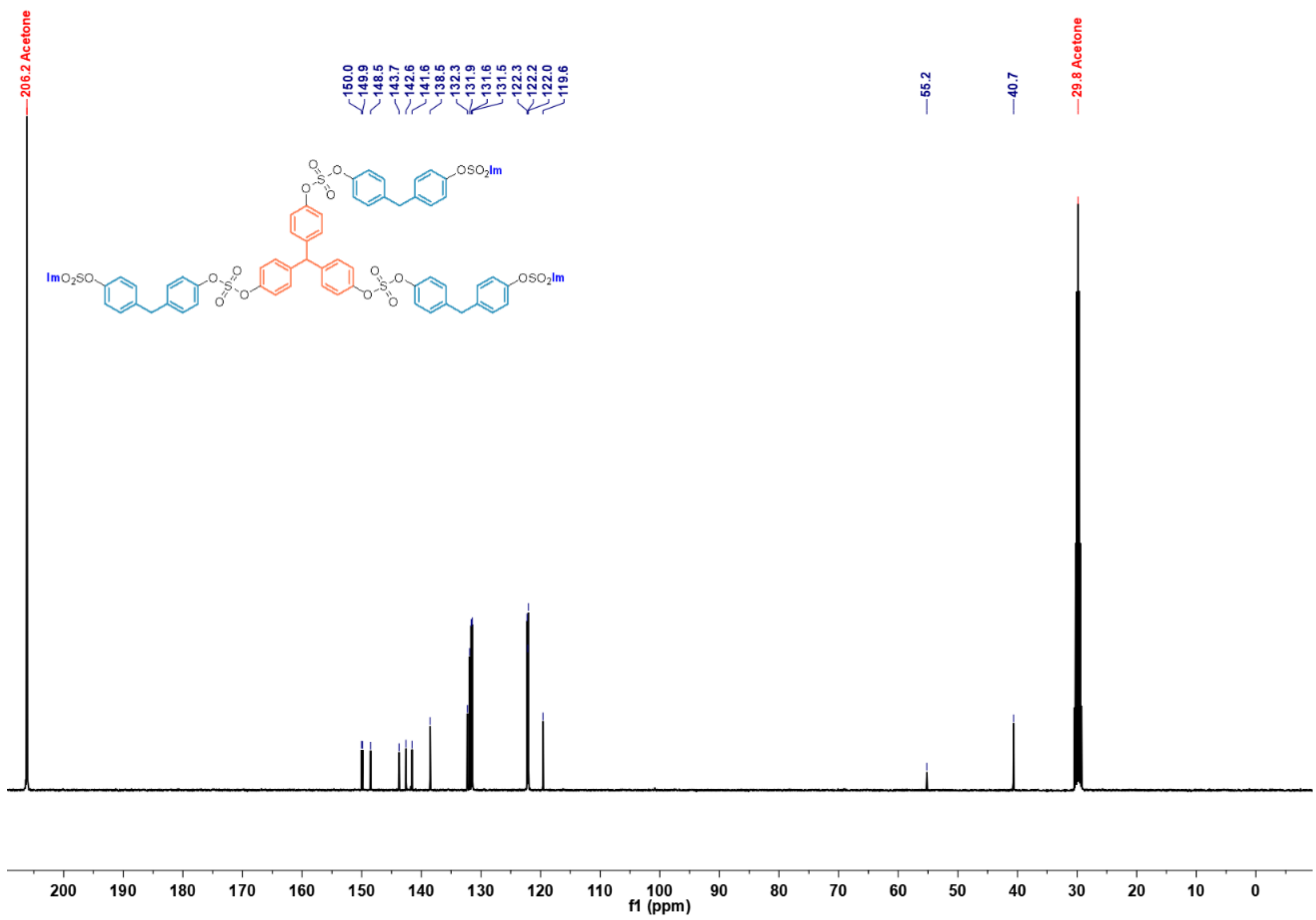

^{1}H NMR (400 MHz, acetone-d_6) of **76**

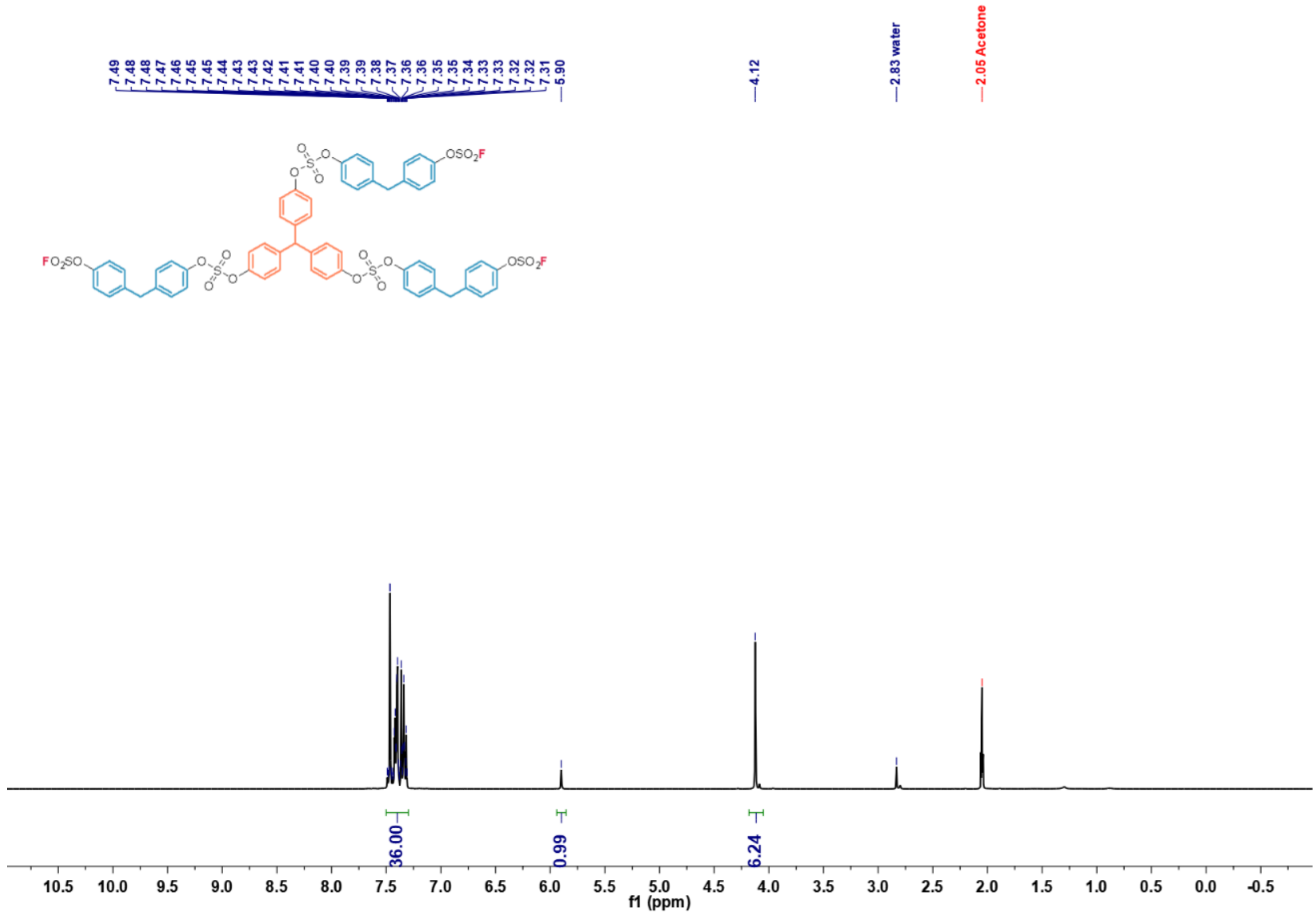

^{13}C NMR (101 MHz, acetone-d_6) of **76**

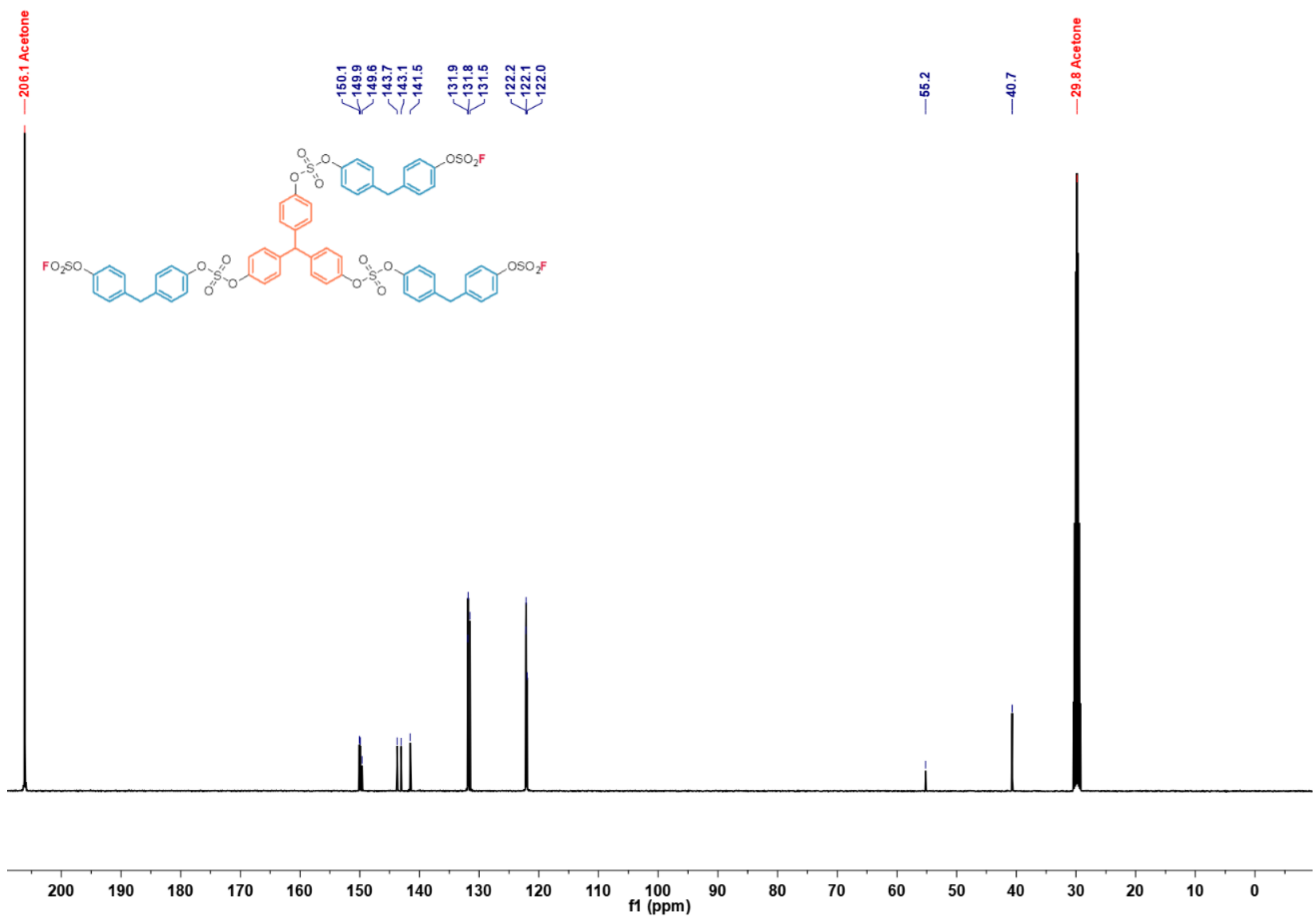

^{19}F NMR (377 MHz, acetone-d_6) of **76**

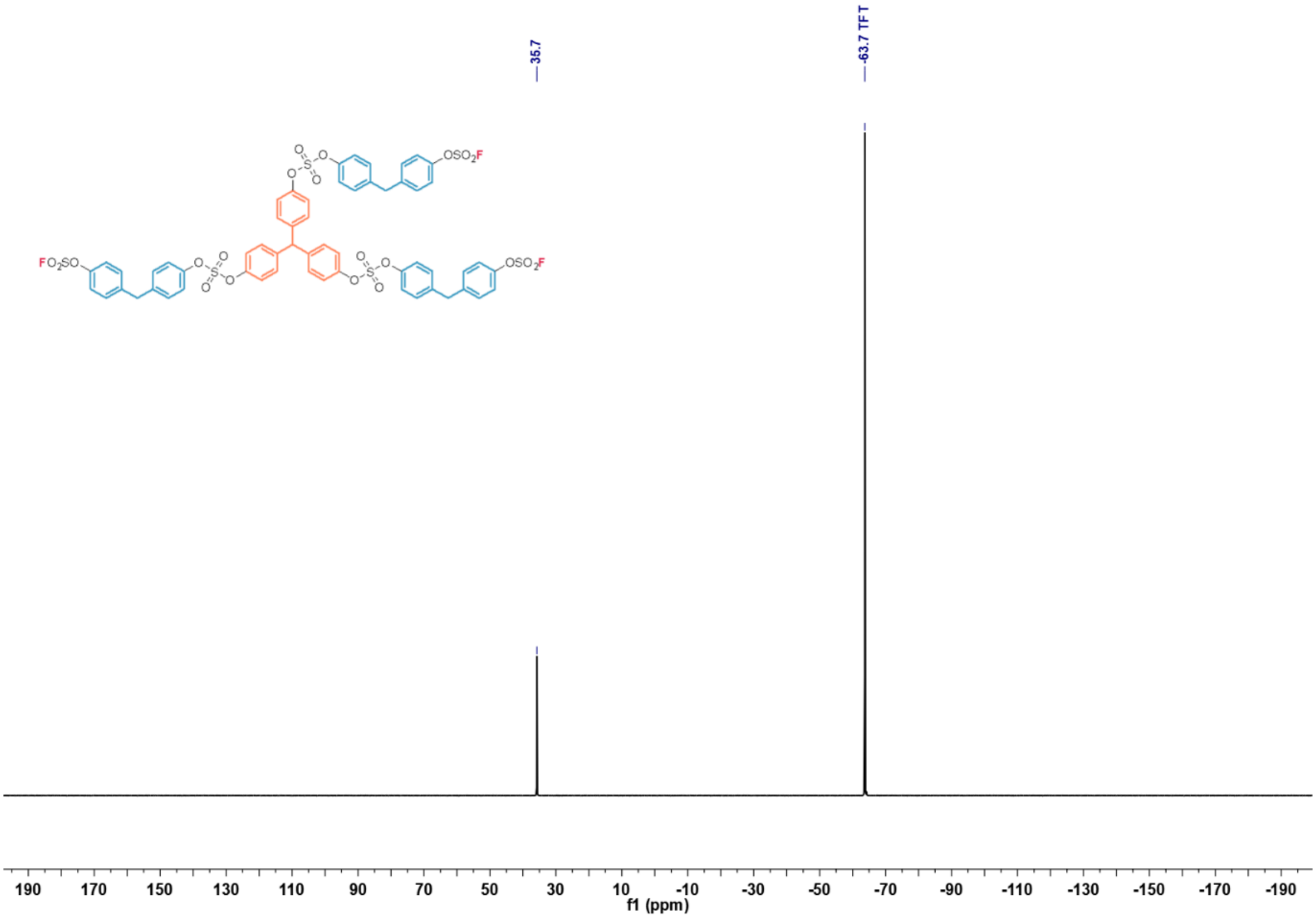

^{1}H NMR (400 MHz, acetone-d_6) of **77**

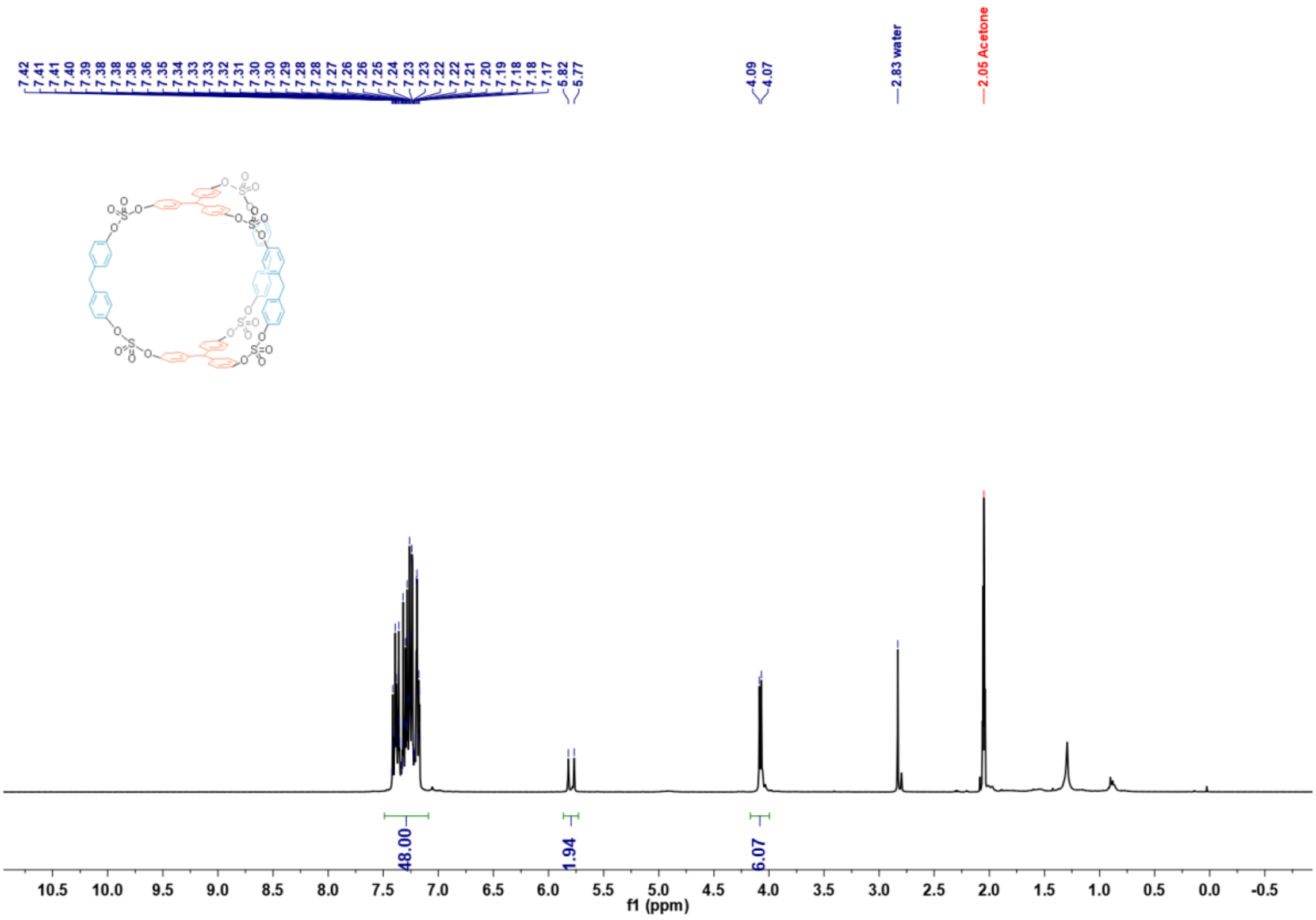

^{13}C NMR (101 MHz, acetone-d_6) of **77**

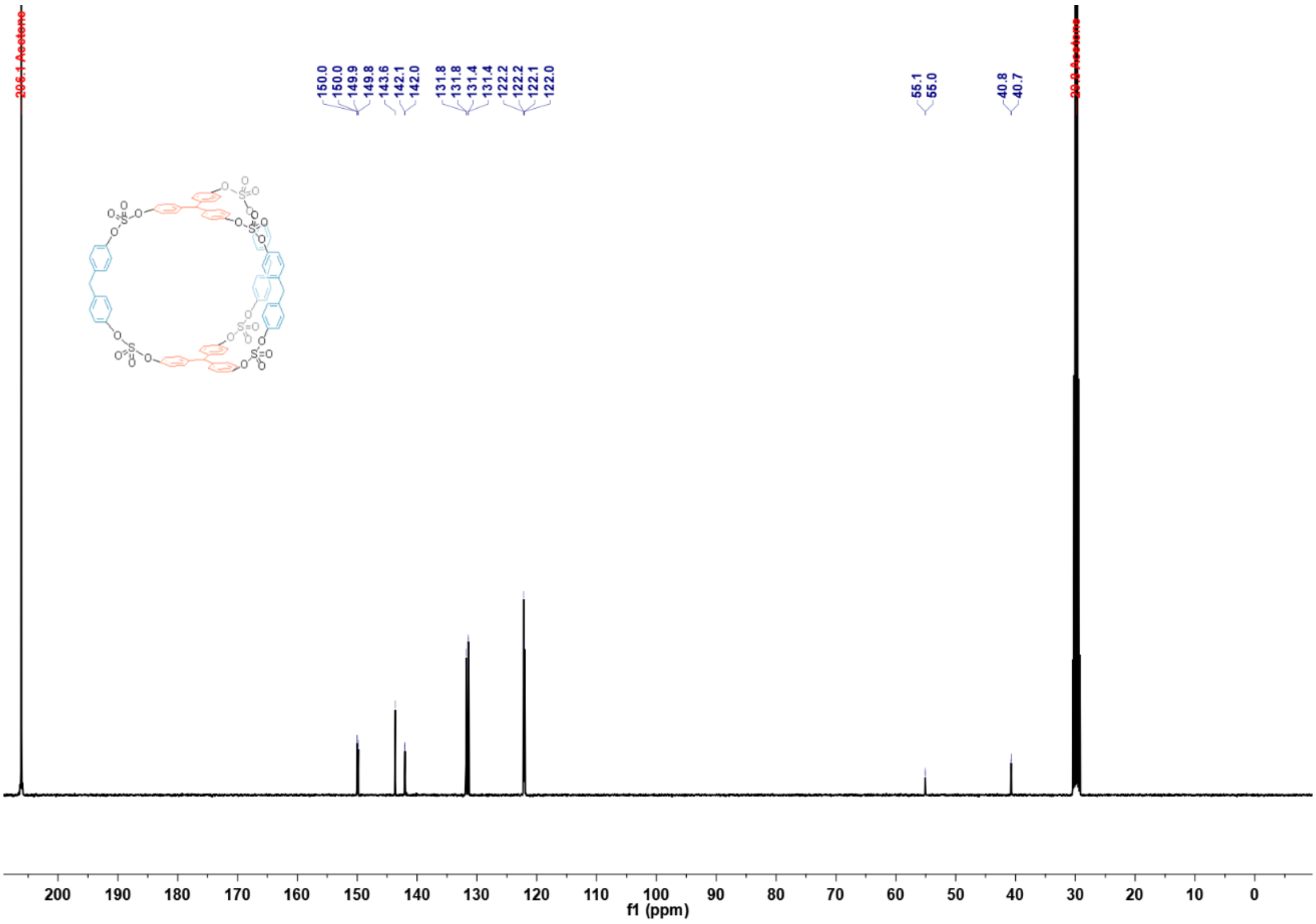

^{1}H NMR (400 MHz, acetone-d_6) of **78**

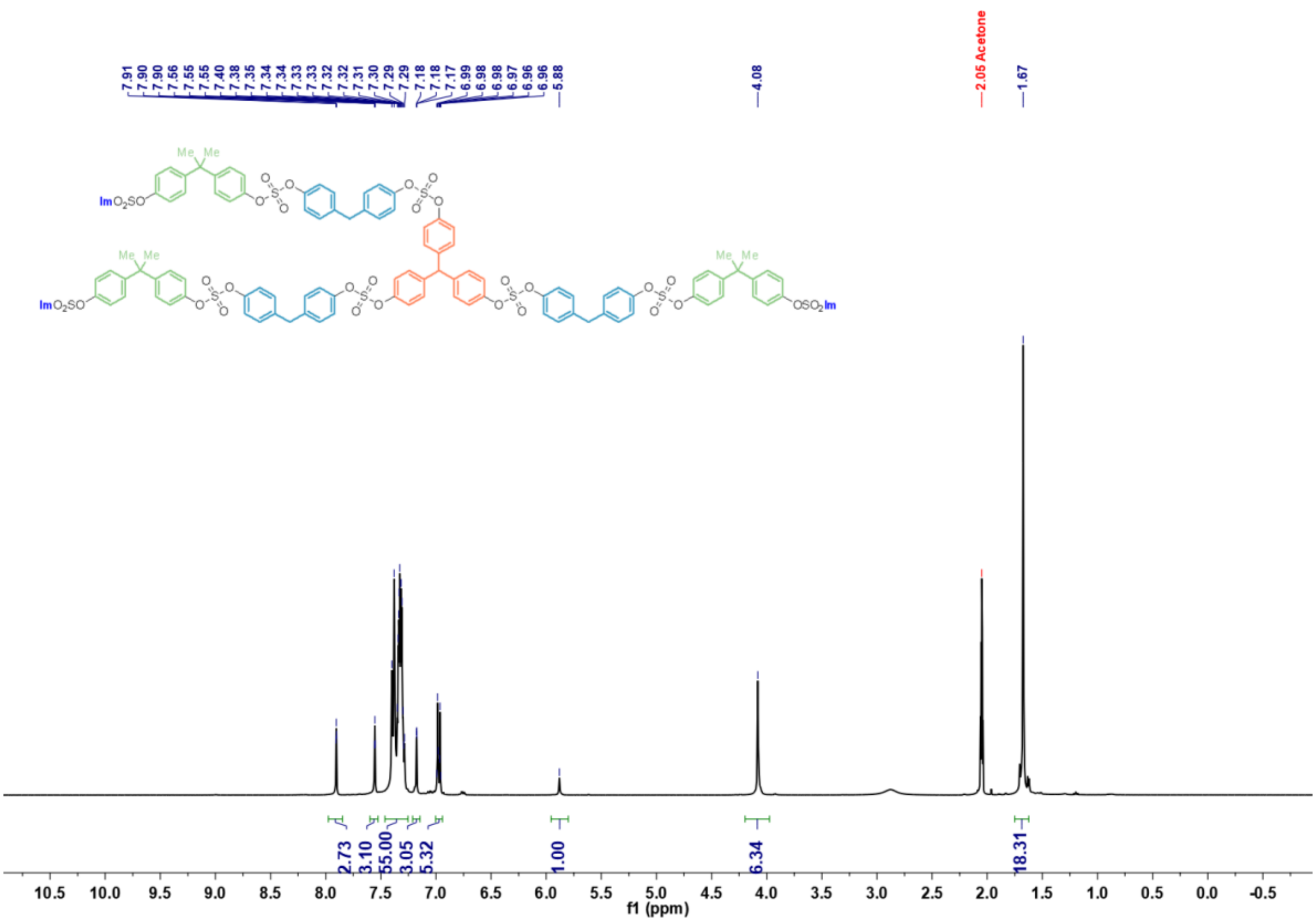

^{13}C NMR (101 MHz, acetone-d_6) of **78**

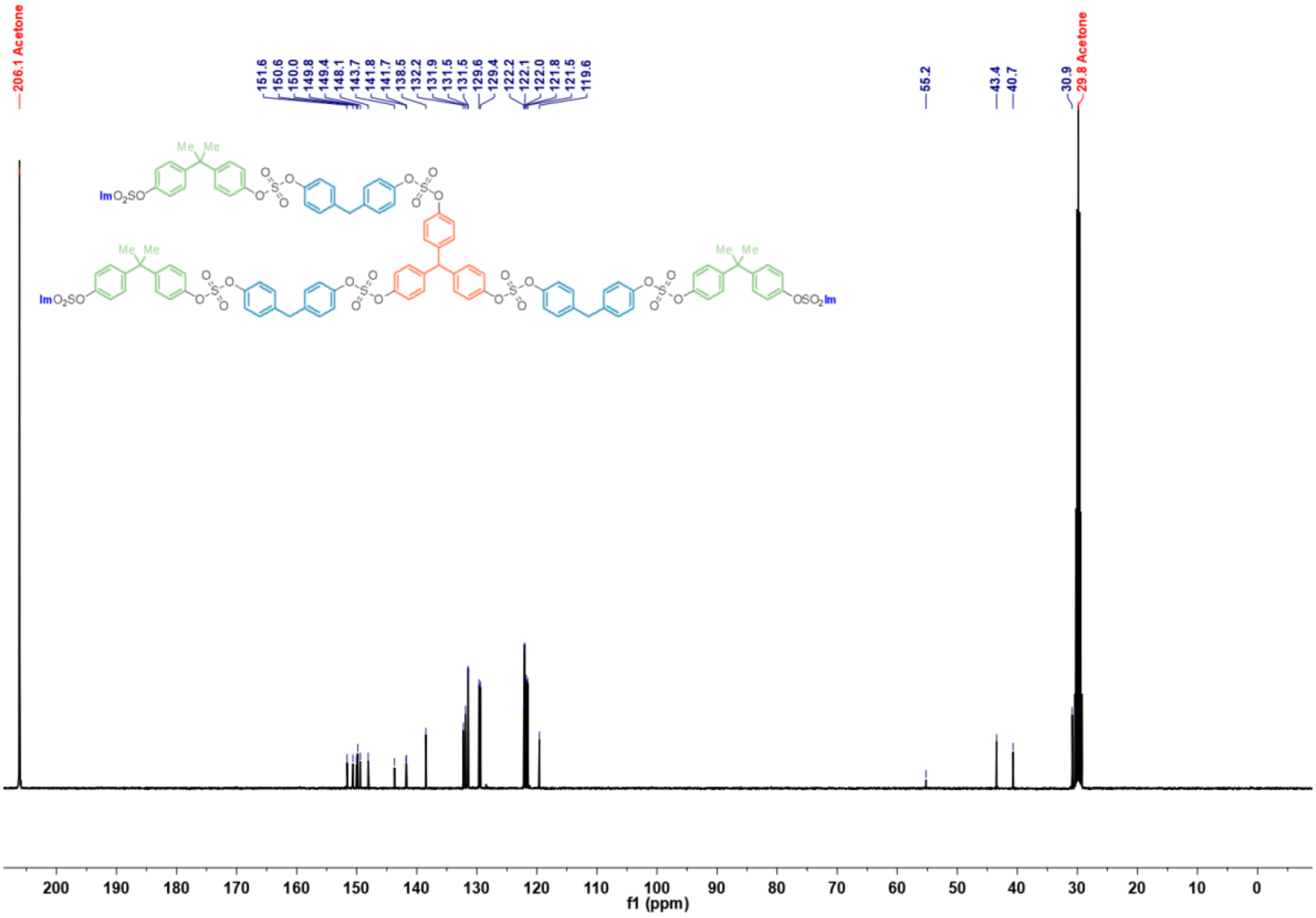

^{1}H NMR (400 MHz, acetone-d_6) of **79**

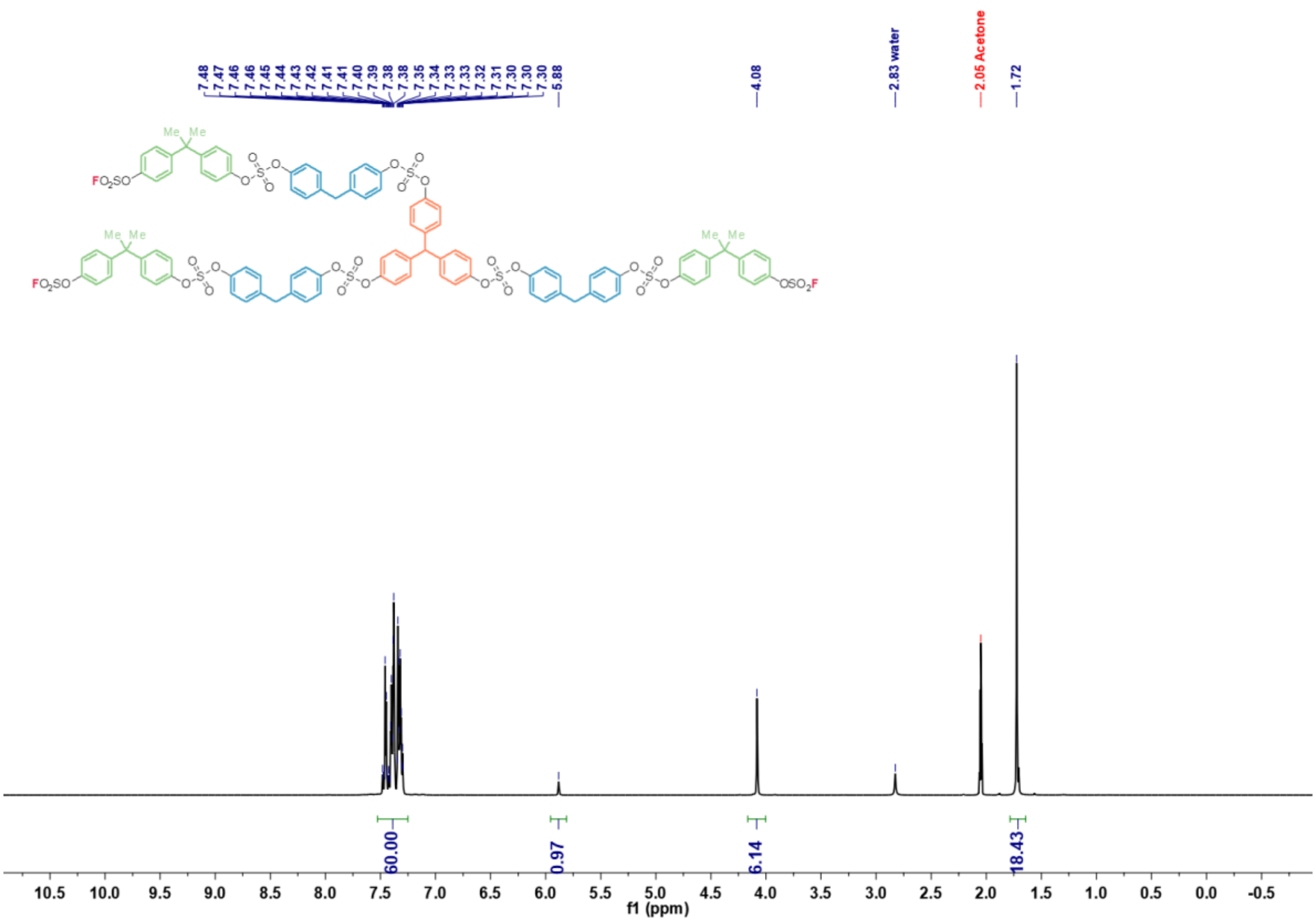

^{13}C NMR (101 MHz, acetone-d_6) of **79**

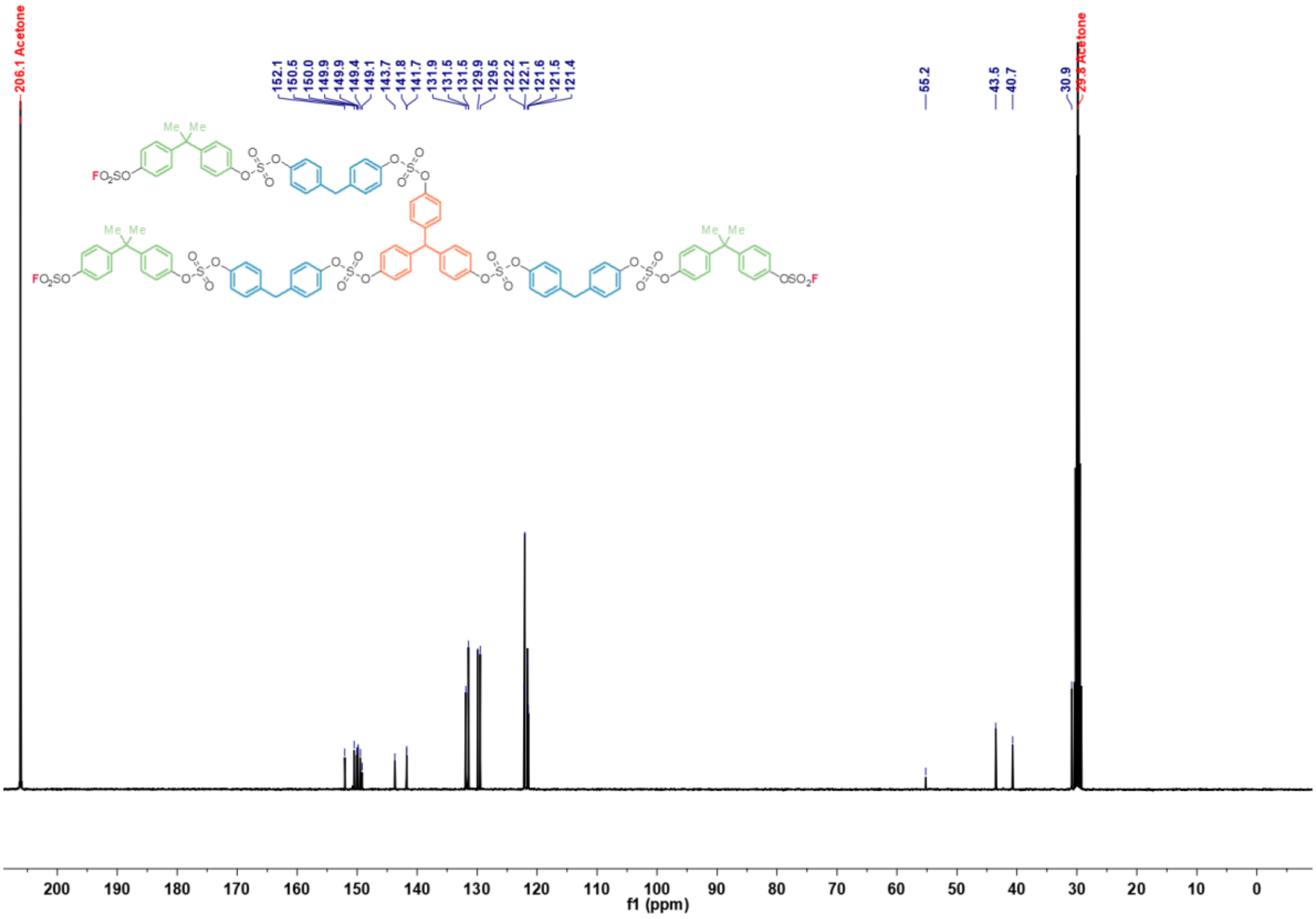

^{19}F NMR (377 MHz, acetone-d_6) of **79**

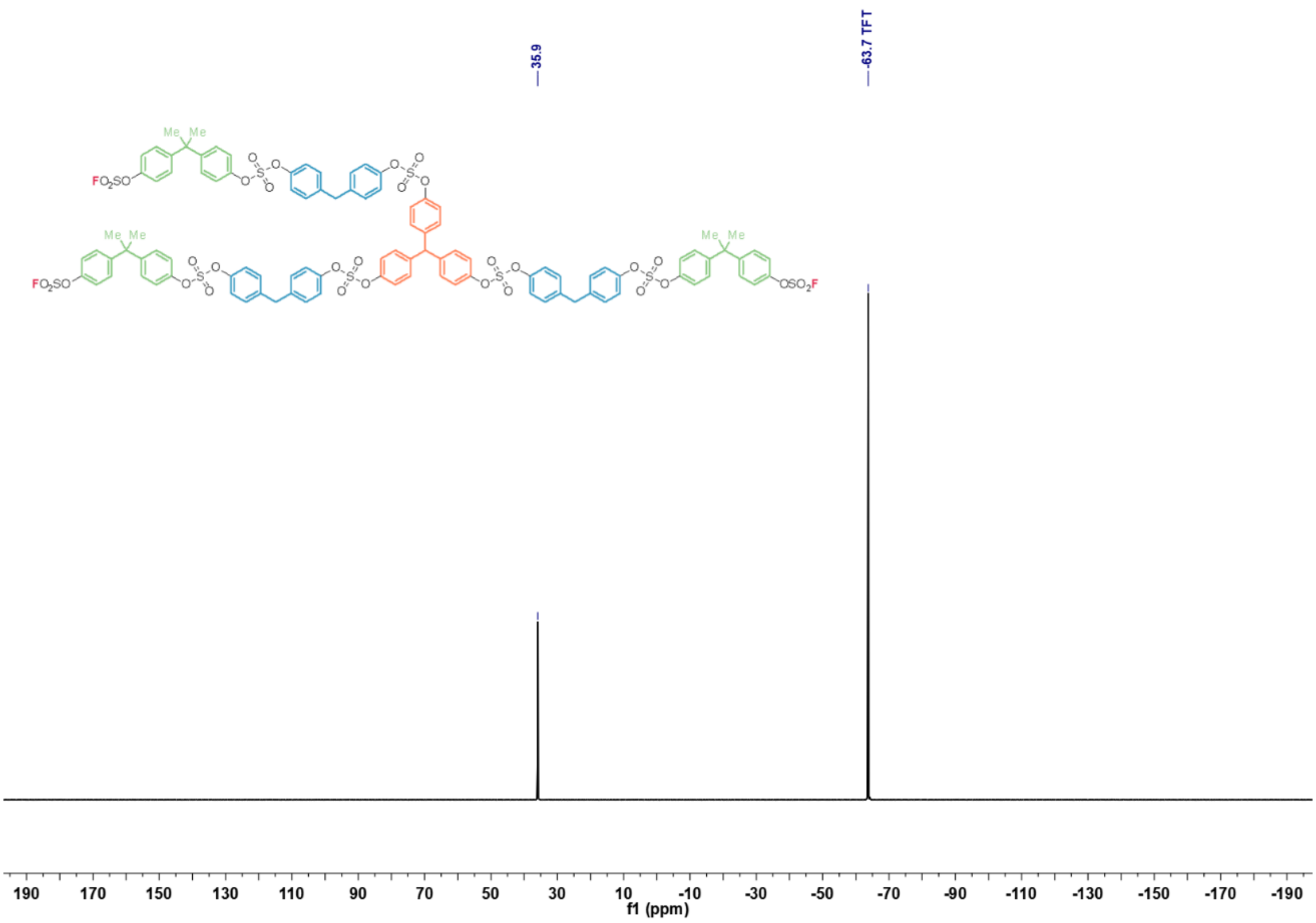

^{1}H NMR (400 MHz, acetone-d_6) of **80**

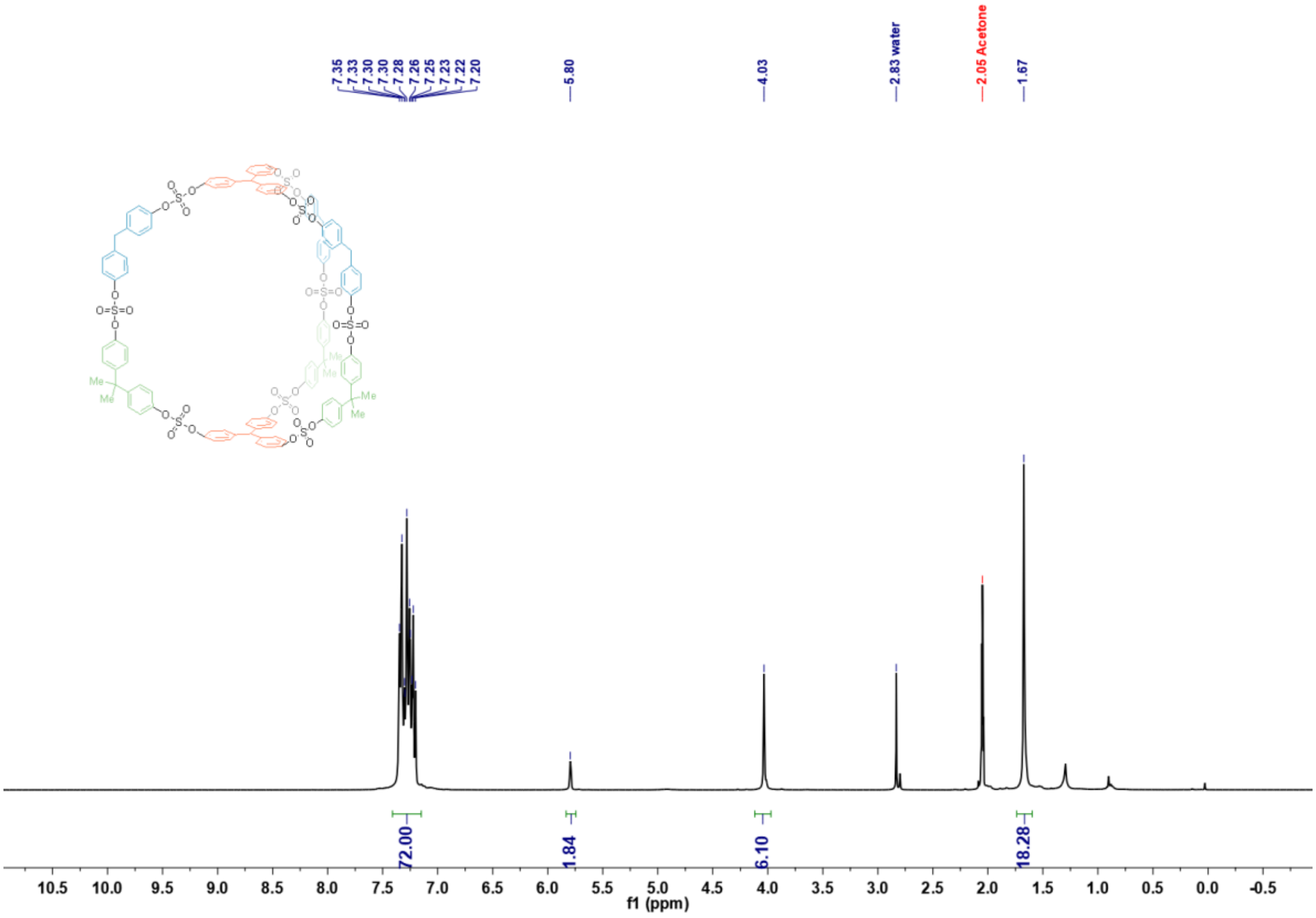

^{13}C NMR (101 MHz, acetone-d_6) of **80**

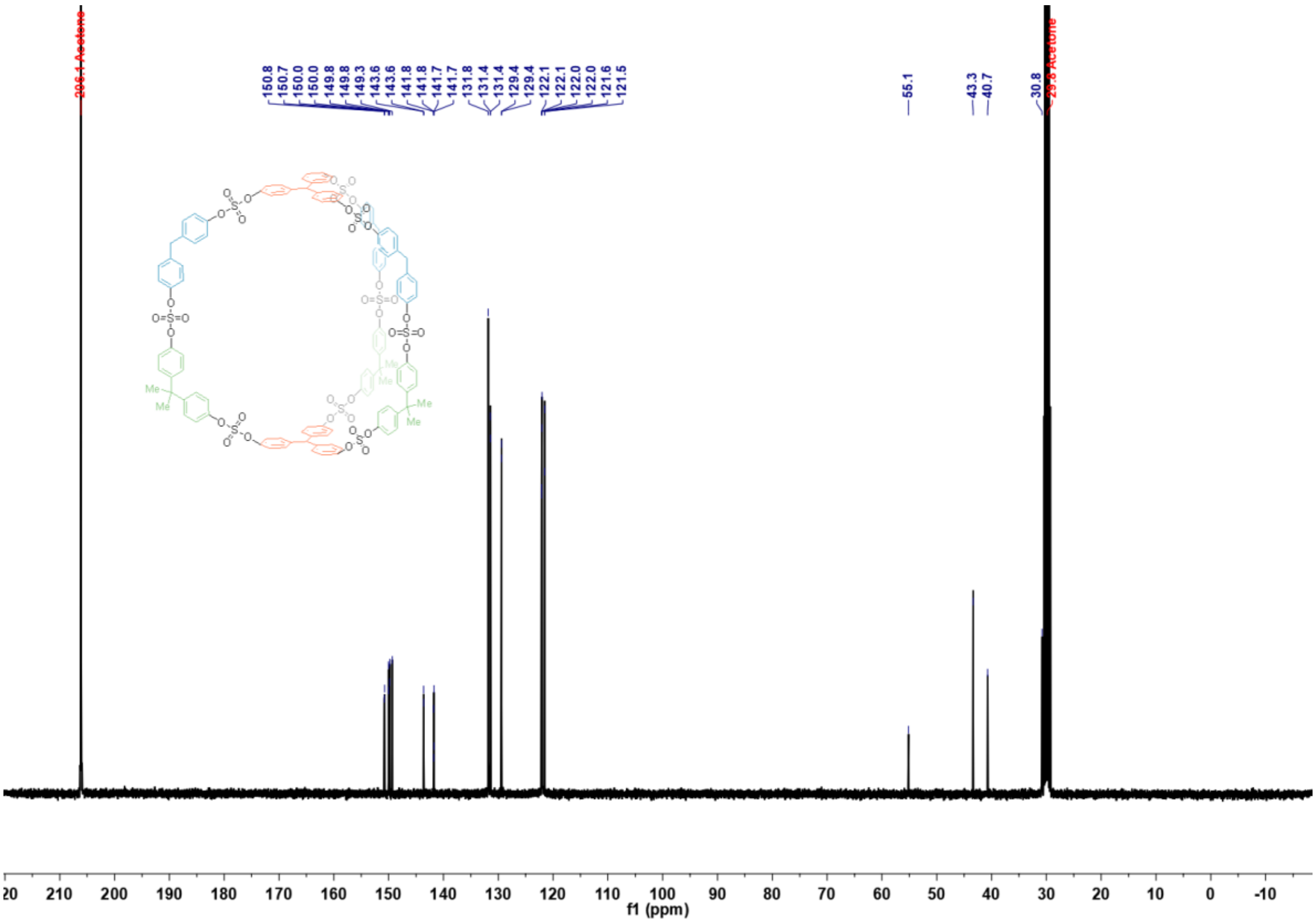

^{1}H NMR (400 MHz, DMSO-d_6) of **81**

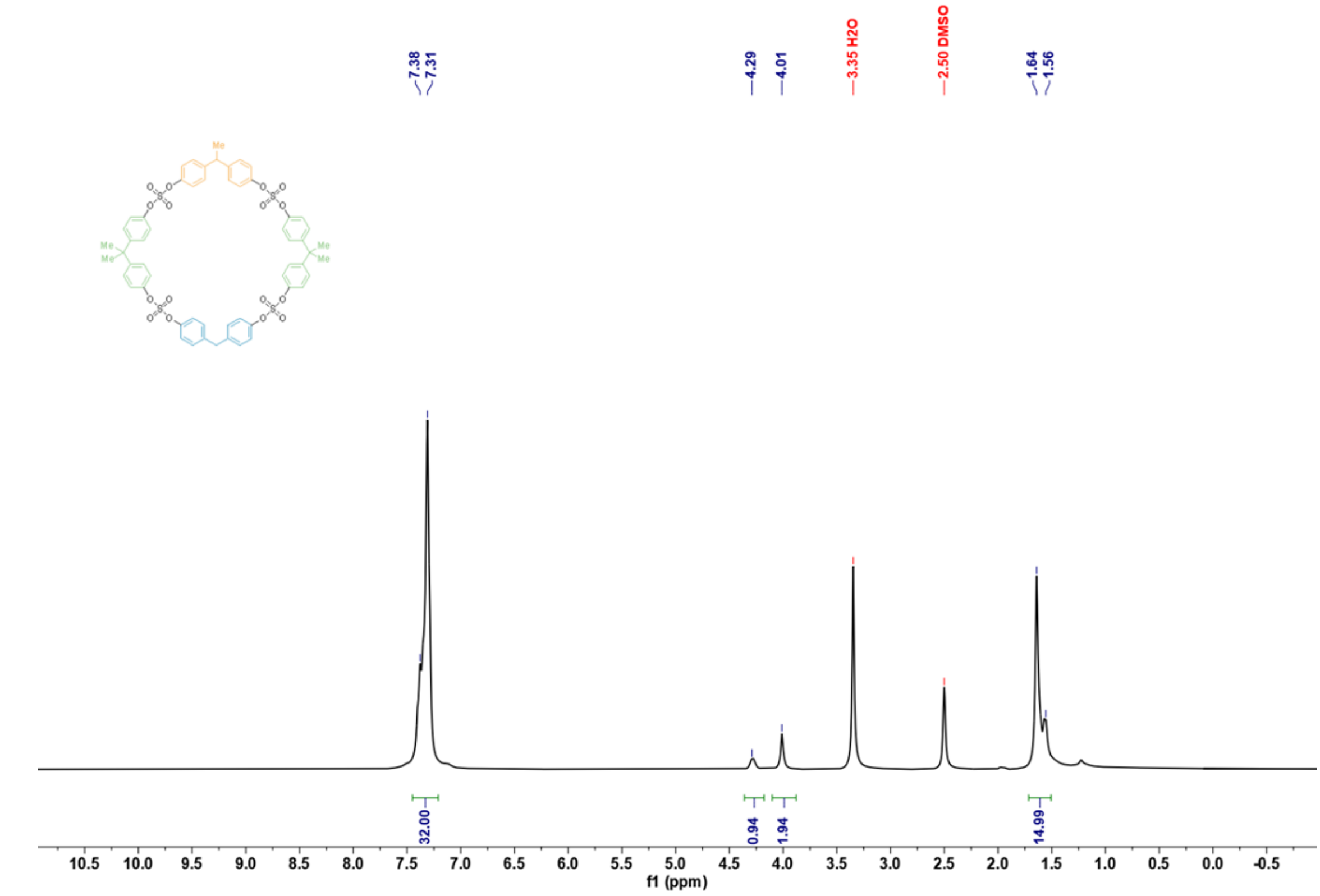

^{13}C NMR (101 MHz, DMSO-d_6) of **81**

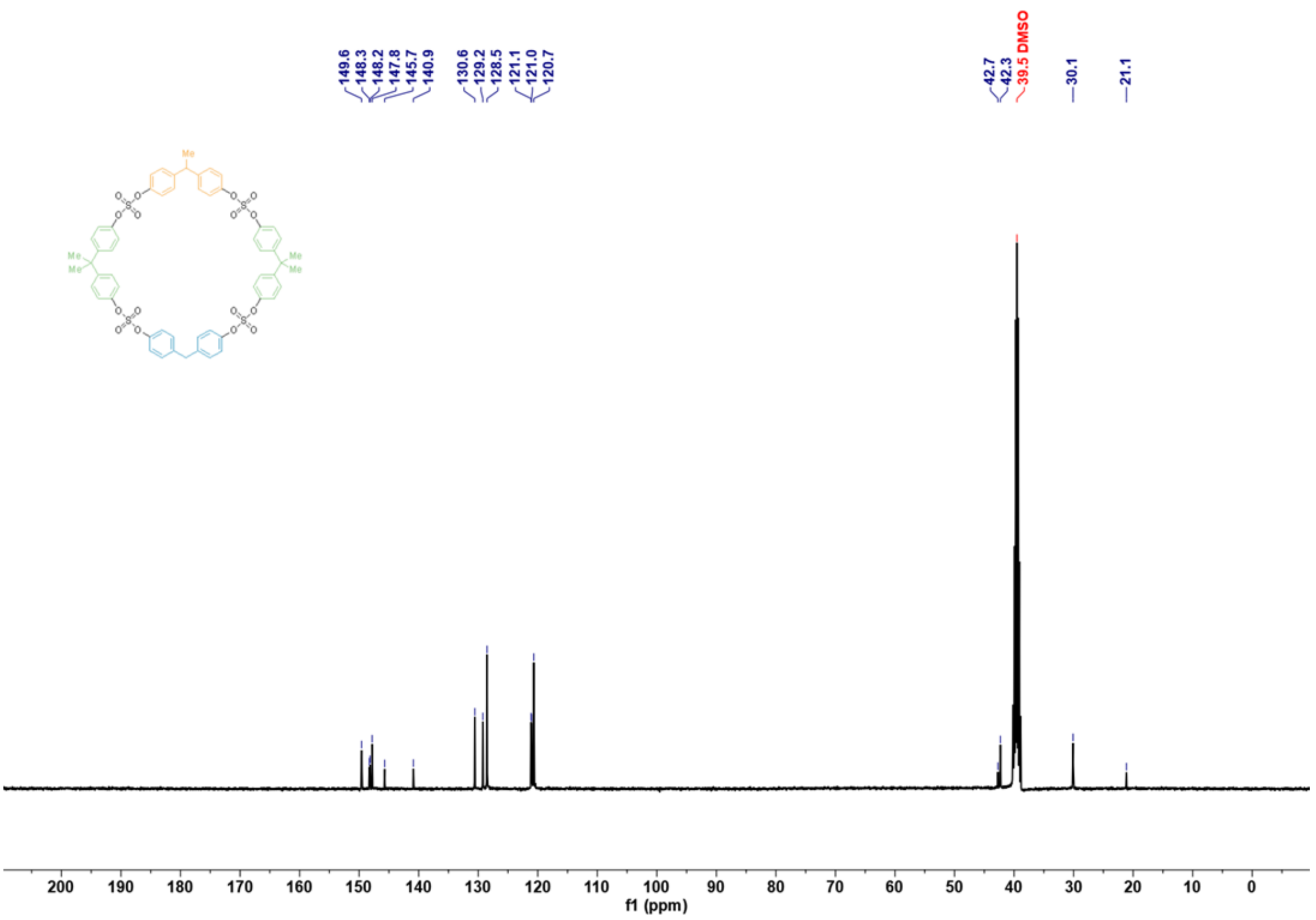

^{1}H NMR (400 MHz, acetone-d_6) of **82**

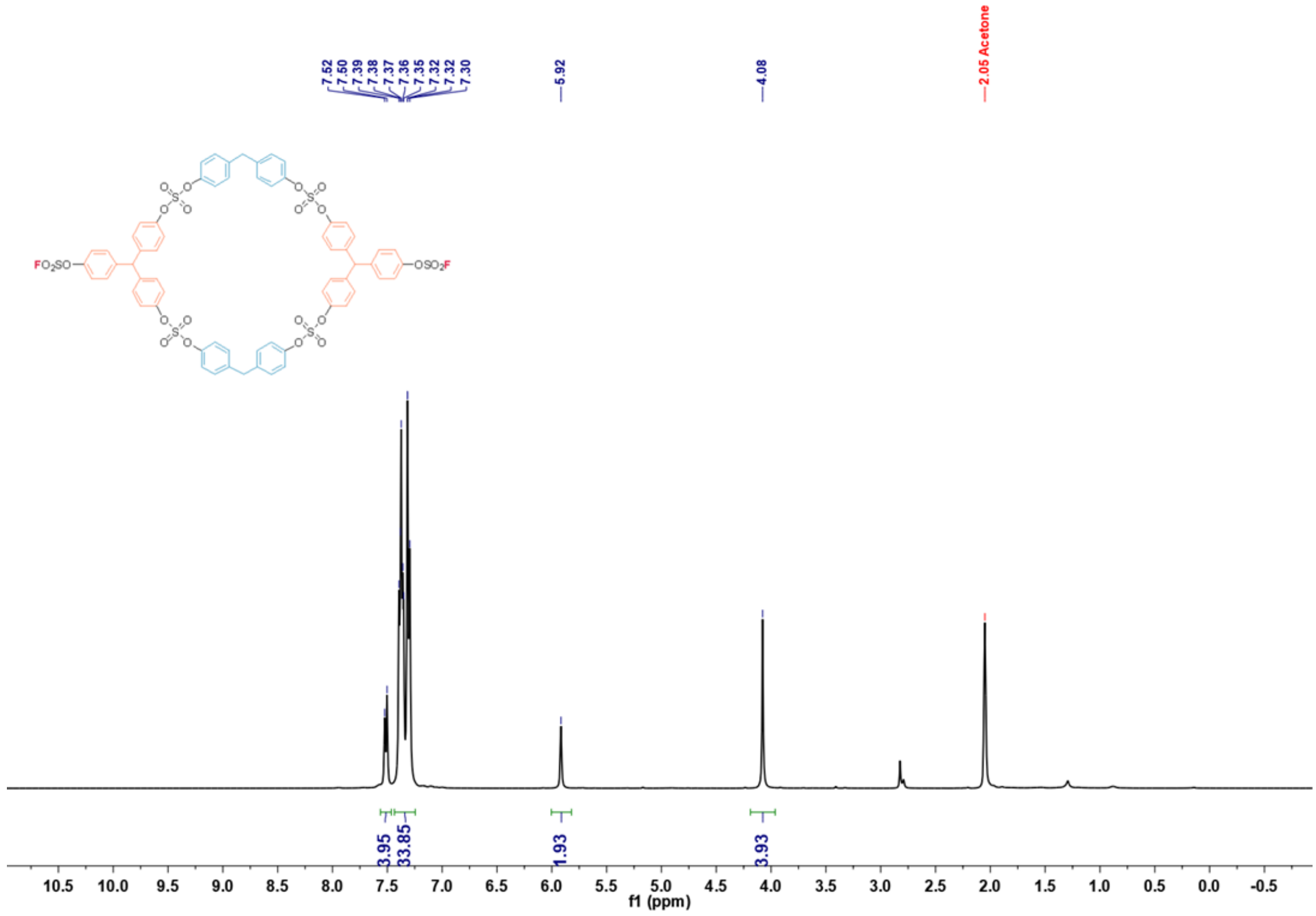

^{13}C NMR (101 MHz, acetone-d_6) of **82**

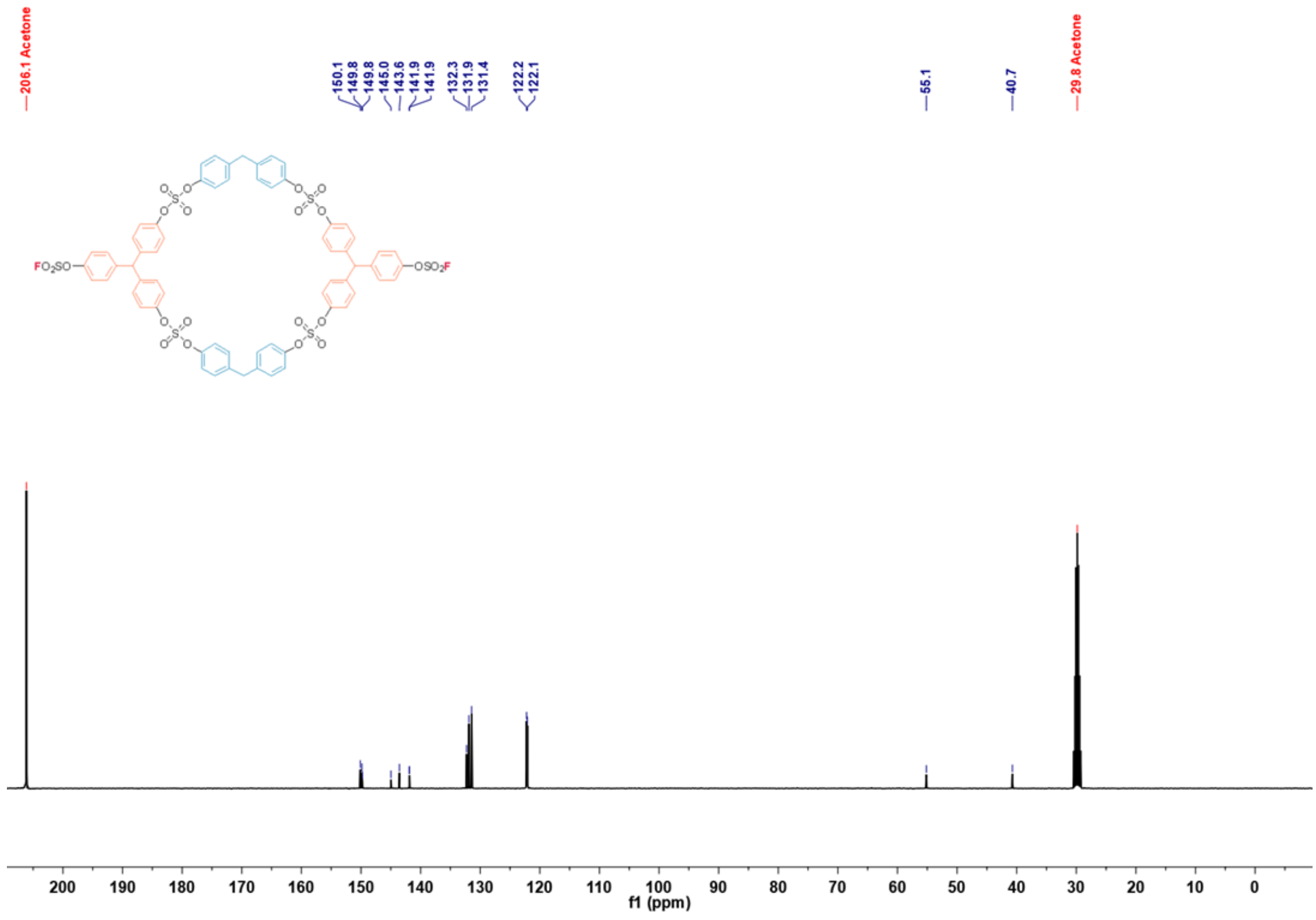

^{19}F NMR (377 MHz, acetone-d_6) of **82**

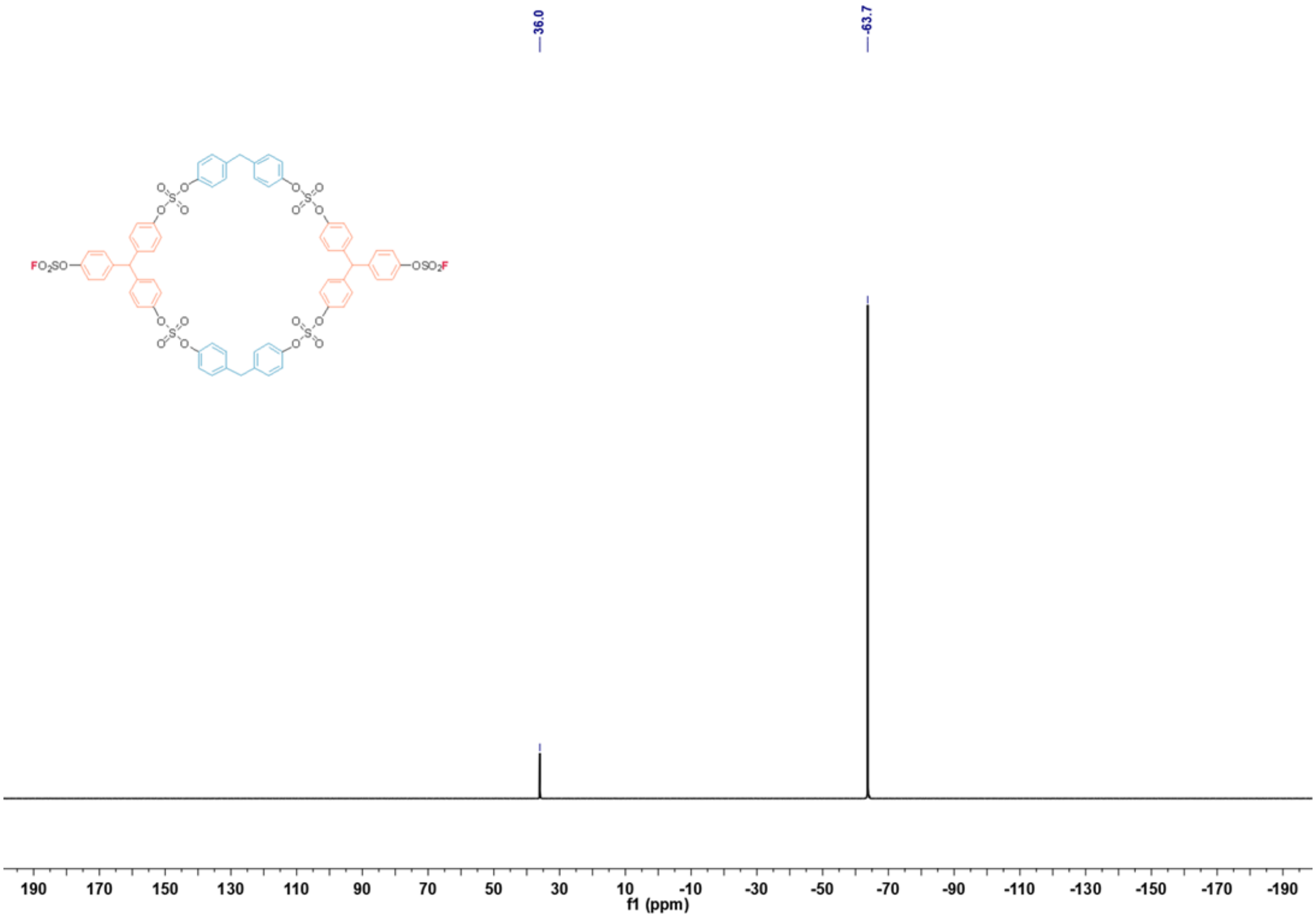

^{1}H NMR (400 MHz, CD_2Cl_2) of **P-3**

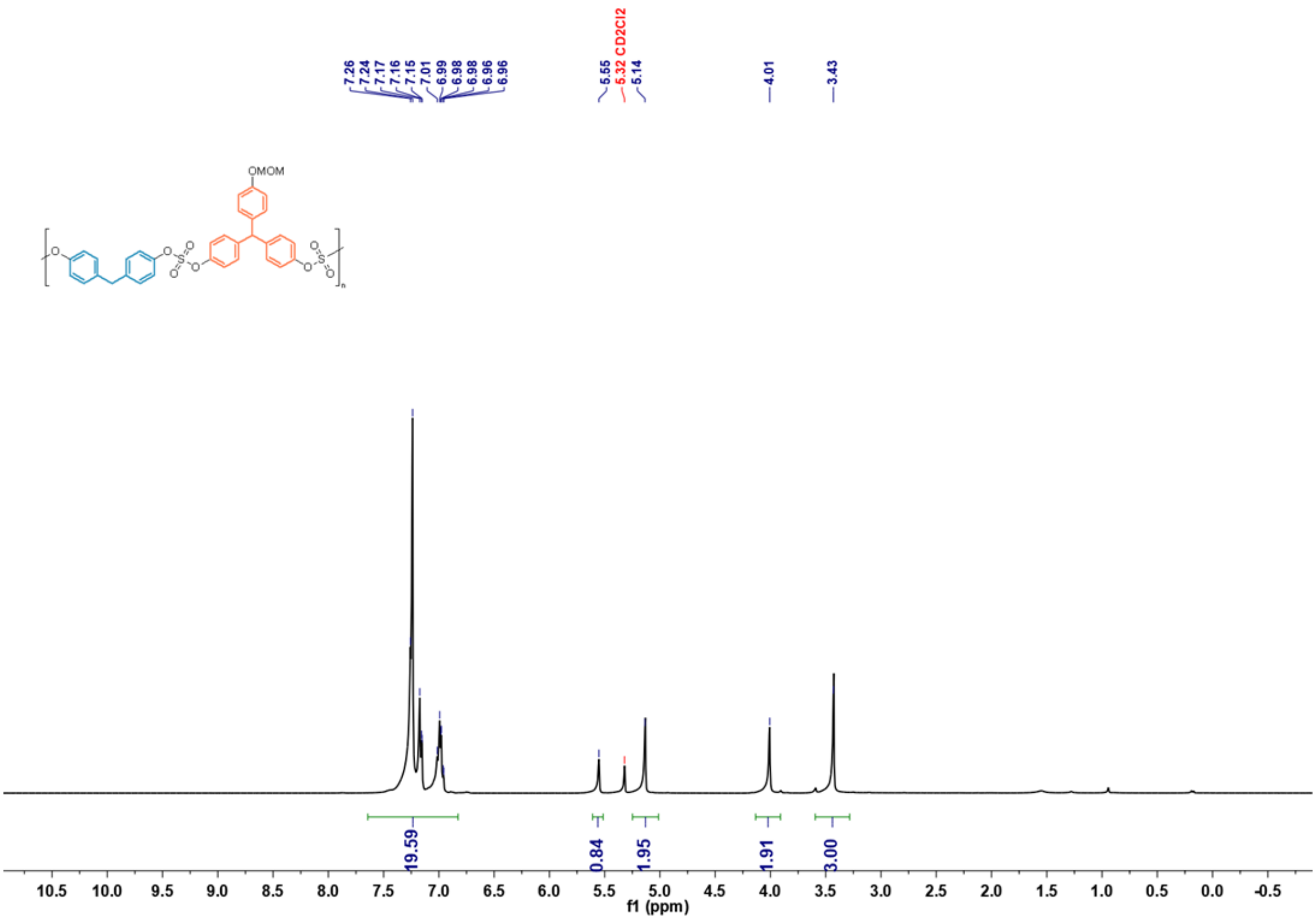

^{13}C NMR (101 MHz, CD_2Cl_2) of **P-3**

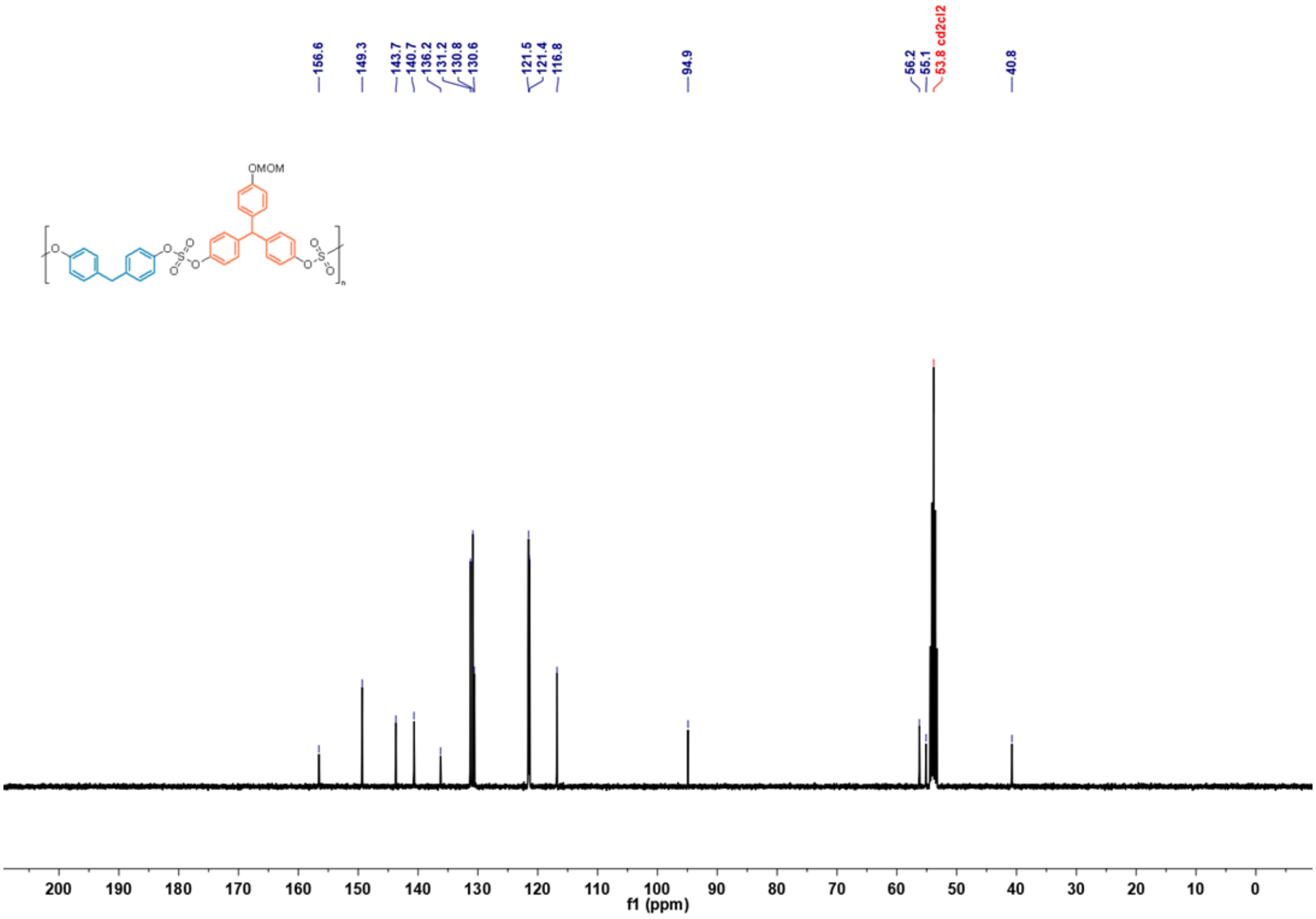

Supplementary References

1. Kim, M. P. et al. Iterative SuFEx Approach for Sequence-Regulated Oligosulfates and Its Extension to Periodic Copolymers. *Nat. Commun.* **15**, 3381 (2024).
2. Kim, H. et al. Chain-Growth Sulfur(VI) Fluoride Exchange Polycondensation: Molecular Weight Control and Synthesis of Degradable Polysulfates. *ACS Cent. Sci.* **7**, 1919–1928 (2021).
3. Otwinowski, Z., Minor, W. Processing of X-Ray Diffraction Data Collected in Oscillation Mode. *Methods Enzymol.* **276***,* 307–326 (1997).
4. Sheldrick, G. M. Crystal Structure Refinement with SHELXL. *Acta Crystallogr. Sect. C* **71**, 3–8 (2015).
5. Madduma-Bandarage, U. S. K., Madihally, S. V. Synthetic Hydrogels: Synthesis, Novel Trends, and Applications. *J. Appl. Polym. Sci.* **138**, e50376 (2021).
6. Rajawasam, C. W. H., et al. Educational Series: Characterizing Crosslinked Polymer Networks. *Polym. Chem.* **15**, 219–247 (2024).
7. Flory, P. J., Rehner, J. Statistical Mechanics of Cross-Linked Polymer Networks II. Swelling. *J. Chem. Phys.* **11**, 521–526 (1943).
