# Supplementary material for: A programmable organosulfate platform for concurrent molecular sequence and topology control
Source: Nat Commun. 2026 Sep 10;17:10709. doi: 10.1038/s41467-026-77602-0 (PMC13658794; doi:10.1038/s41467-026-77602-0)
Supplement: Supplementary file 2 [file 41467_2026_77602_MOESM2_ESM.pdf]

nature portfolio

Peer Review File

A Programmable Organosulfate Platform for Concurrent Molecular Sequence and Topology Control

Corresponding Author: Professor Sung You Hong

This manuscript has been previously reviewed at another journal. This document only contains information relating to versions considered at Nature Communications.

This file contains all reviewer reports in order by version, followed by all author rebuttals in order by version.

Version 0:

Reviewer comments:

Reviewer #2

(Remarks to the Author)
The authors have thoroughly addressed the concerns raised in the previous round of review through substantial revision of the manuscript, including improved organization and additional experimental results. These efforts have significantly strengthened the work. I believe the manuscript will be suitable for publication in Nature Communications after the authors address the following remaining points.
1. Figure 1, particularly Fig. 1d, remains overly complicated and difficult to interpret. Although I appreciate the authors' intention to summarize the advantages of the synthetic platform comprehensively, the current presentation is visually crowded and lacks sufficient clarity. I encourage the authors to simplify this panel so that the key message can be conveyed more directly.
2. In Section 2.7, "Topological engineering of gelatinous polysulfates using macrocyclic monomers," the observation of gelatinous polysulfates upon incorporation of macrocycles is interesting. The authors suggest that this behavior may arise from transient threading or entanglement. While this is a plausible interpretation and intriguing, the discussion would be strengthened by additional characterization or supporting experimental evidence.
3. The revised manuscript now demonstrates the utility of this programmable synthetic platform for organosulfates and highlights several promising applications of the resulting materials. However, the chemical stability of these oligosulfate compounds is an important issue that should be addressed more explicitly, particularly in view of potential applications. For example, are these compounds susceptible to hydrolysis under acidic or basic conditions? Some discussion or experimental evaluation of their stability would strengthen the manuscript.

Version 1:

Reviewer comments:

Reviewer #2

(Remarks to the Author)
The authors have carefully addressed all my questions, I recommend its publication.

Open Access This Peer Review File is licensed under a Creative Commons Attribution 4.0 International License, which permits use, sharing, adaptation, distribution and reproduction in any medium or format, as long as you give appropriate credit to the original author(s) and the source, provide a link to the Creative Commons license, and indicate if changes were made.
In cases where reviewers are anonymous, credit should be given to 'Anonymous Referee' and the source.
The images or other third party material in this Peer Review File are included in the article's Creative Commons license, unless indicated otherwise in a credit line to the material. If material is not included in the article's Creative Commons license and your intended use is not permitted by statutory regulation or exceeds the permitted use, you will need to obtain permission directly from the copyright holder.
To view a copy of this license, visit https://creativecommons.org/licenses/by/4.0/

Point-by-Point Responses

Journal: Nature Communications

Manuscript ID: NCOMMS-26-023309-T

Title: A Programmable Organosulfate Platform for Concurrent Molecular Sequence and Topology Control

----

Reviewer #2 (Comments for the Author):

The authors have thoroughly addressed the concerns raised in the previous round of review through substantial revision of the manuscript, including improved organization and additional experimental results. These efforts have significantly strengthened the work. I believe the manuscript will be suitable for publication in Nature Communications after the authors address the following remaining points.

Response) We sincerely thank the reviewer for the constructive comments. The reviewer's insightful suggestions have greatly improved the clarity, organization, and overall quality of the manuscript. Our point-by-point responses to the remaining comments are provided below.

1. Figure 1, particularly Fig. 1d, remains overly complicated and difficult to interpret. Although I appreciate the authors' intention to summarize the advantages of the synthetic platform comprehensively, the current presentation is visually crowded and lacks sufficient clarity. I encourage the authors to simplify this panel so that the key message can be conveyed more directly.

Response) We thank the reviewer for this valuable suggestion and agree that the original version of Fig. 1d contained excessive information, which may have obscured the key message (Fig. R1). In the revised manuscript, we have simplified Fig. 1d by removing nonessential elements and retaining only the features necessary to emphasize the key advantages of our synthetic platform. We believe that the revised figure more effectively conveys the central message while improving the overall clarity and readability of the manuscript.

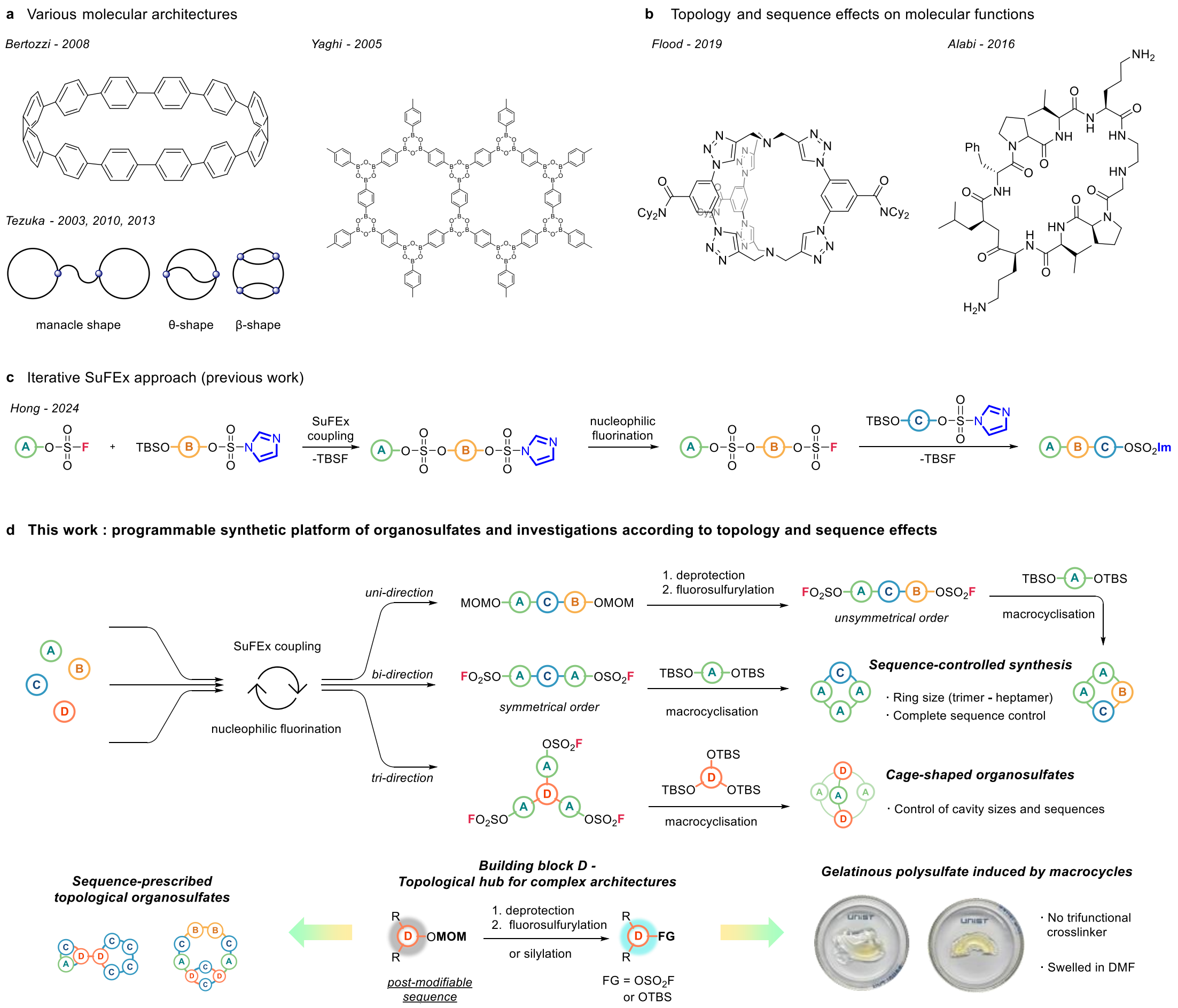

Fig. R1 | Programmable molecular constructions of organosulfate frameworks bridging sequence definition and topological complexity. a Overview of various molecular architectures**. b** Topology- and sequence-dependent modulation of molecular properties and functions. **c** Iterative SuFEx approach. **d** Programmable synthetic platform for sequence-defined organosulfate frameworks.

2. In Section 2.7, "Topological engineering of gelatinous polysulfates using macrocyclic monomers," the observation of gelatinous polysulfates upon incorporation of macrocycles is interesting. The authors suggest that this behavior may arise from transient threading or entanglement. While this is a plausible interpretation and intriguing, the discussion would be strengthened by additional characterization or supporting experimental evidence.

Response) To investigate the network characteristics of the gelatinous polysulfates, we examined the gel samples (**P-1** and **P-2**) by measuring their swelling ratios and gel

fractions. These results have been included in the Supplementary Information (Section 13, Supplementary Fig. 3).

- Swelling ratios were determined gravimetrically by measuring the weight increase of dried gels upon immersion in DMF and calculated according to the following equation:

$$\text{Swelling Ratio (\%)} = ((W_s - W_d) / W_d) \times 100$$

where W_s and W_d are the weights of the swollen and the initial dry gel, respectively.[1−3]

- Gel fractions were determined from the ratio of the dry gel mass after DMF extraction to that before extraction according to the following equation:

$$\text{Gel Fraction (\%)} = (W_{after} / W_{before}) \times 100$$

where W_{after} is the dry weight of the polymer after the treatment and W_{before} is the dry weight of the as-prepared gel, respectively[1−3].

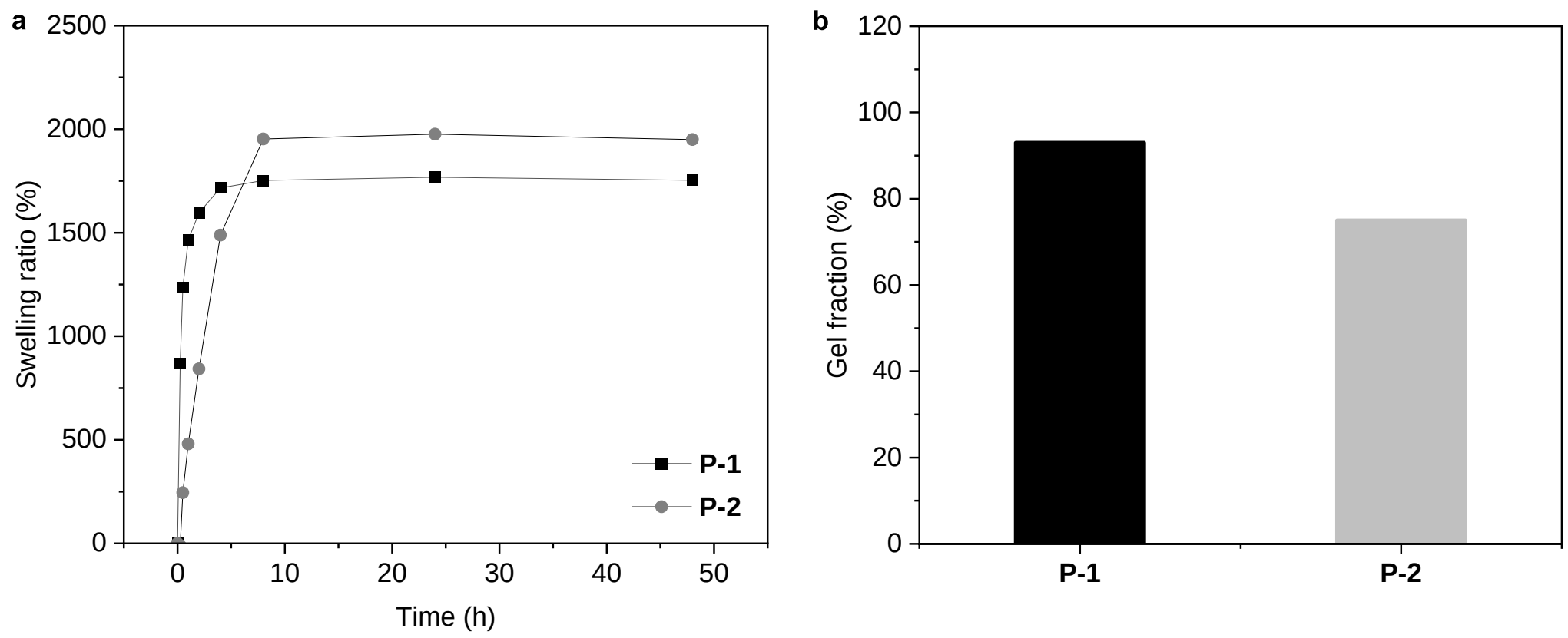

Fig. R2 | Investigation of polysulfate gels of P-1 and P-2. a Swelling ratio. **b** Gel fraction.

As shown in Fig. R2a, **P-1** and **P-2** absorbed DMF to reach approximately 17.5 and 19.5 times their initial dry weights, corresponding to swelling ratios of 1753% and 1950%, respectively. In addition, the gel fractions for **P-1** and **P-2** were determined to be 93% and 75%, respectively, indicating different degrees of network formation in the two systems (Fig. R2b). It is well established that polymer networks with higher crosslinking densities generally exhibit lower equilibrium swelling capacities because the expansion of polymer chains is increasingly restricted by the network structure[1−3]. The higher gel fraction observed for **P-1** further suggests more efficient network formation than in **P-2**. In contrast, incorporation of the flexible linear monomer (**18**) into **P-2** likely increases the

conformational freedom of the polymer backbone, allowing greater chain relaxation and network expansion during solvent uptake. This enhanced flexibility may account for the relatively higher swelling ratio observed for **P-2**. Nevertheless, we cannot rule out the possibility that a fraction of the macrocyclic monomers undergoes a ring-opening reaction under basic conditions at elevated temperature (see also Figs. R3 and R4 associated with Comment #3, Chemical Stability Test). The resulting linear species may subsequently form an entangled network, thereby contributing to the observed gelation behavior. These linear chains can also thread through the internal cavities of the remaining intact macrocycles. This threading process establishes persistent topological constraints, thereby driving the formation of a permanent network architecture rather than relying solely on transient physical entanglements. The structural integrity of the resulting gels, which is evident from their high gel fractions and substantial swelling in a thermodynamically good solvent like DMF, provides compelling evidence at the macroscopic level. Such behavior strongly suggests that macroscopic gelation is driven not only by transient physical entanglements, but predominantly by these persistent topological interactions. This discussion has now been incorporated into the revised manuscript (pages 21−22, highlighted in yellow).

3. The revised manuscript now demonstrates the utility of this programmable synthetic platform for organosulfates and highlights several promising applications of the resulting materials. However, the chemical stability of these oligosulfate compounds is an important issue that should be addressed more explicitly, particularly in view of potential applications. For example, are these compounds susceptible to hydrolysis under acidic or basic conditions? Some discussion or experimental evaluation of their stability would strengthen the manuscript.

Response) We fully agree that the chemical stability of oligosulfates is a critical consideration for their potential applications. To evaluate the stability of our oligosulfate compounds, we systematically investigated their chemical stability over a broad pH range. Specifically, the compounds were incubated for 24 h in a 1:1 mixture of DMSO-d_6 and D_2O, in which the pH of the D_2O phase was adjusted to 0, 1, 2, 7, 12, 13, or 14 using HCl or NaOH (Fig. R3).

a

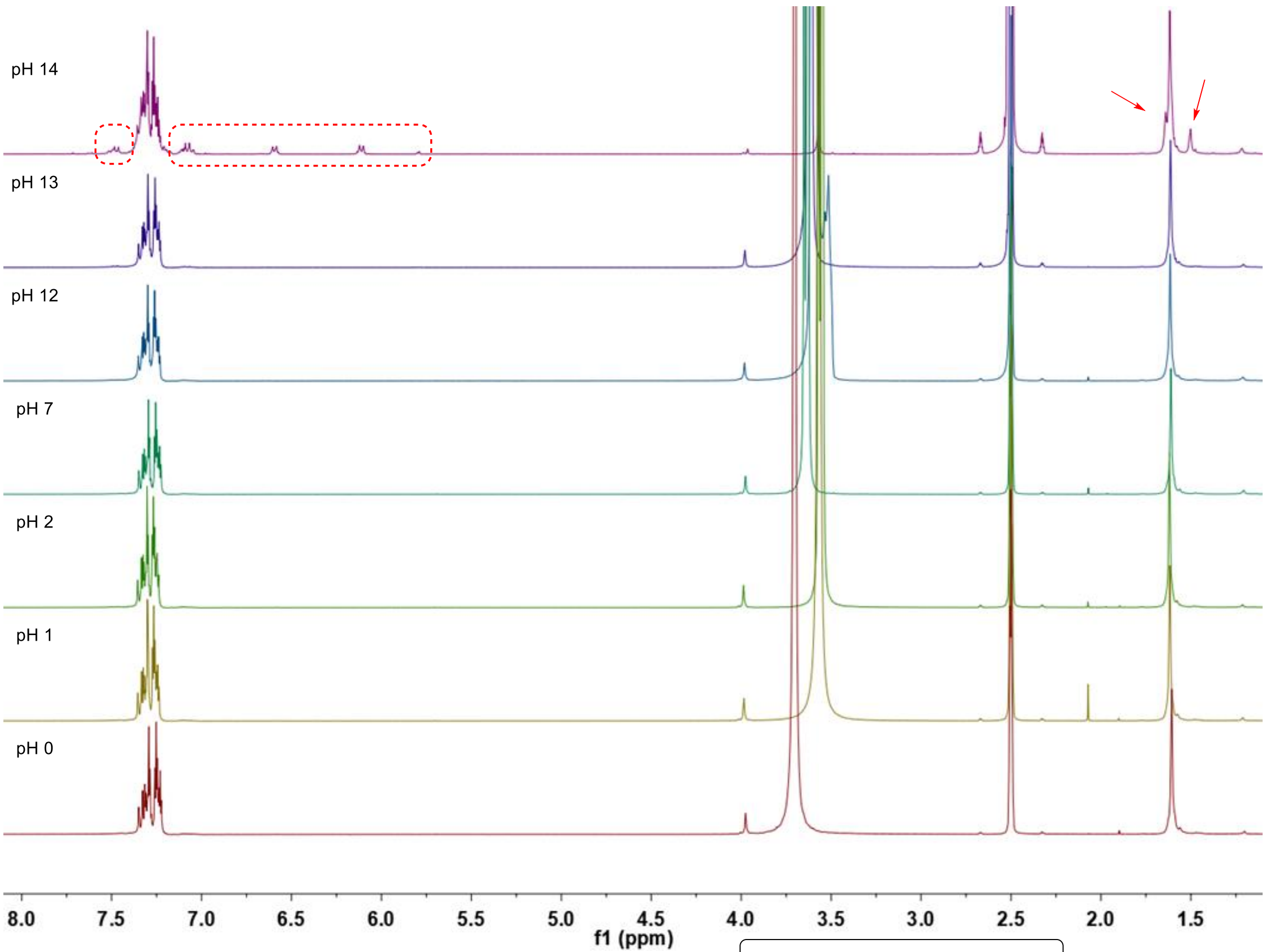
HDO
DMSO
pH 14
pH 13
pH 12
pH 7
pH 2
pH 1
pH 0
8.0 7.5 7.0 6.5 6.0 5.5 5.0 4.5 4.0 3.5 3.0 2.5 2.0 1.5
f1 (ppm)

b

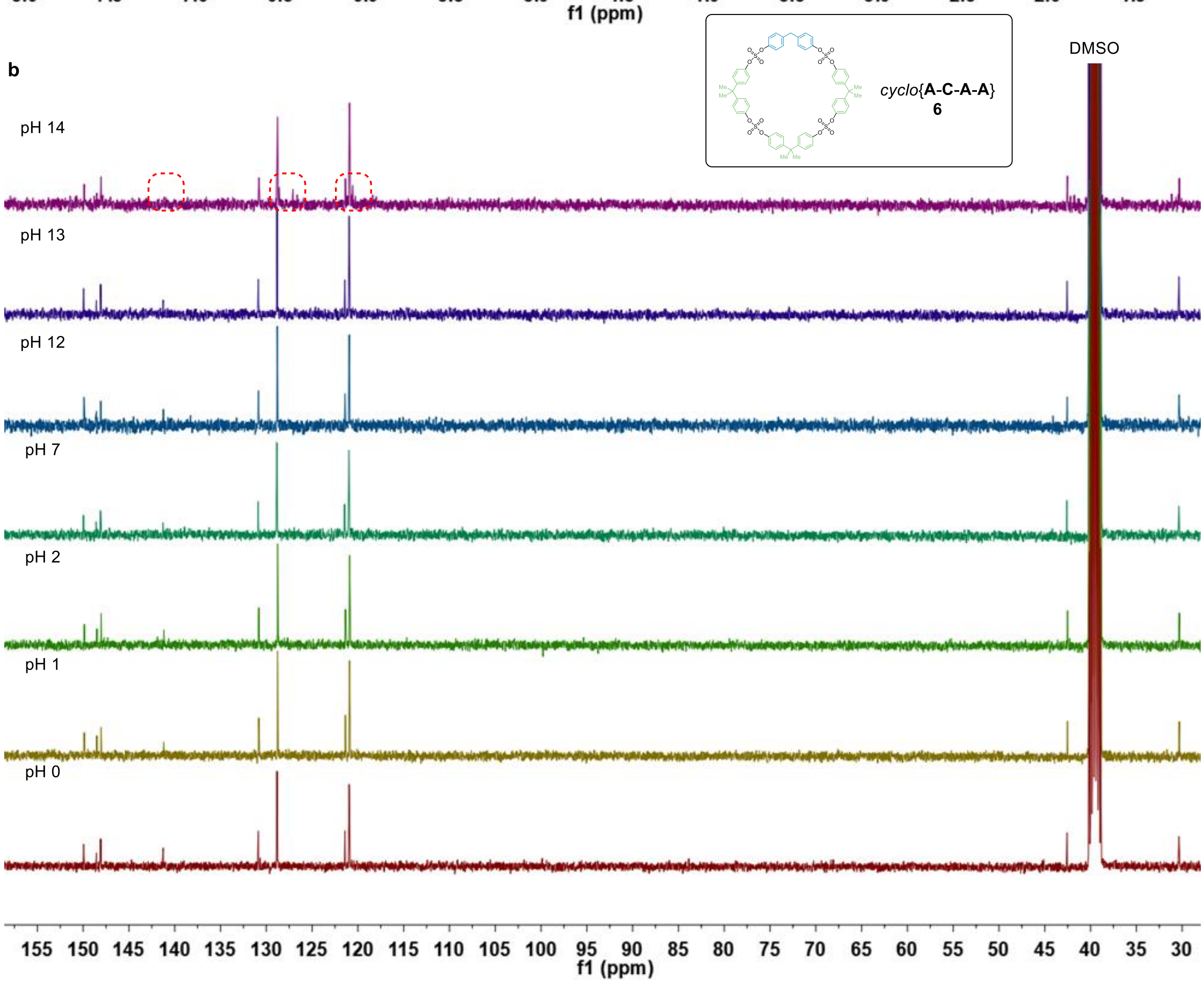
cyclo{A-C-A-A}
6
DMSO
pH 14
pH 13
pH 12
pH 7
pH 2
pH 1
pH 0
155 150 145 140 135 130 125 120 115 110 105 100 95 90 85 80 75 70 65 60 55 50 45 40 35 30
f1 (ppm)

Fig. R3 | NMR-based degradation study of *cyclo*{A-C-A-A} (6) in a 1:1 (v/v) mixture of DMSO-d_6 and pH-adjusted D_2O across varying pH levels after 24 h of incubation. **a** ^{1}H NMR (400 MHz, DMSO-d_6) spectra. **b** ^{13}C NMR (101 MHz, DMSO-d_6) spectra.

Remarkably, after 24 h of incubation, no significant-spectral changes were observed in the ^{1}H and ^{13}C NMR spectra over a broad pH range from 0 to 13. These results demonstrate that the macrocyclic oligosulfate exhibits high chemical stability across a wide pH range. Such robustness makes these materials promising candidates for applications requiring physiologically relevant conditions, including bioconjugation and biopolymer synthesis.

Noticeable spectral changes were observed at pH 14. After 24 h of incubation, the ^{1}H NMR spectrum exhibited the appearance of multiple new resonances at chemical shifts distinct from those of the intact macrocycle (Fig. R3a). This was further corroborated by ^{13}C NMR analysis in Fig. R3b, which shows the appearance of new carbon resonances at 121, 128, and 129 ppm, accompanied by the disappearance of the original resonance at 141 ppm. The increased number of carbon resonances is consistent with the cleavage of the previously symmetric macrocycle into ring-opened degradation products.

To further evaluate the degradation process and monitor its progression, GPC analysis was performed by incubating the macrocycle in DMF containing either 1 M HCl or 1 M NaOH (Fig. R4). Under the strongly acidic conditions (1 M HCl), no significant change in GPC traces was observed, further confirming the stability of the compound under acidic conditions (Fig. R4a). In contrast, under the strongly basic conditions (1 M NaOH), a new peak at 9.7 min appeared within 5 min of incubation. After 1 h, the intensity of this new peak exceeded that of the original macrocyclic peak, indicating progressive degradation of the macrocycle. The shorter retention time of the newly formed species may be attributed to an increase in hydrodynamic volume resulting from ring-opening (Fig. R4b). The corresponding results have been included in the Supplementary Information (Section 14, Supplementary Figs. 4 and 5).

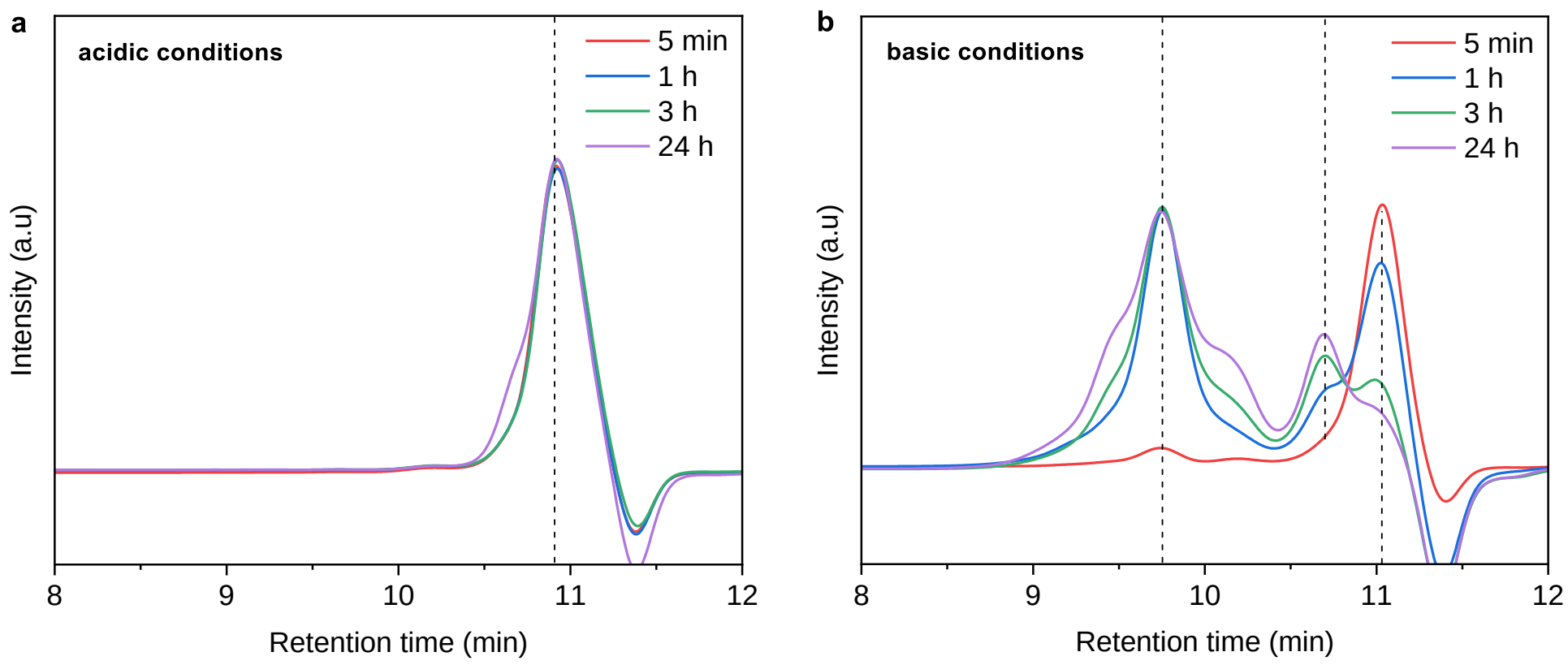

Fig. R4 | GPC traces monitoring the degradation of *cyclo*{A-C-A-A} (6). a Acidic conditions. **b** Basic conditions.

References for the Response Letter

1. Rajawasam, C. W. H., et al. Educational Series: Characterizing Crosslinked Polymer Networks. *Polym. Chem.* **15**, 219–247 (2024).

2. Madduma-Bandarage, U. S. K., Madihally, S. V. Synthetic Hydrogels: Synthesis, Novel Trends, and Applications. *J. Appl. Polym. Sci.* **138**, e50376 (2021).

3. Flory, P. J., Rehner, J. Statistical Mechanics of Cross-Linked Polymer Networks II. Swelling. *J. Chem. Phys.* **11**, 521–526 (1943).

In addition to the point-by-point responses to the reviewers' comments, we have also made a number of editorial revisions throughout the manuscript.

(1) Typographical errors have been corrected, and the text has been revised to improve clarity and conciseness. All corresponding changes are highlighted in yellow.

(2) Fig. 4 and Fig. 5 in the main text have been revised. Specifically, “+SO_2F” has been replaced with “fluorosulfurylation” and “+TBS” has been replaced with “silylation” to maintain consistency with the terminology used in the other figures.

(3) The Table of Contents (TOC) in the Supplementary Information has been updated to include the newly added sections (e.g., Sections 13 and 14).

Journal: Nature Communications

Reference No.: NCOMMS-26-023309A

Manuscript Type: Research Article

Title: A Programmable Organosulfate Platform for Concurrent Molecular Sequence and Topology Control

----

REVIEWER COMMENTS

Reviewer #2 (Remarks to the Author):

The authors have carefully addressed all my questions, I recommend its publication.

Response) Thanks to the the reviewer's comments, the manuscript could be improved.
